# Global, regional, and national prevalence of child and adolescent overweight and obesity, 1990–2021, with forecasts to 2050: a forecasting study for the Global Burden of Disease Study 2021

**DOI:** 10.1016/S0140-6736(25)00397-6

**Published:** 2025-03-08

**Authors:** GBD 2021 Adolescent BMI Collaborators, Jessica A Kerr, George C Patton, Karly I Cini, Yohannes Habtegiorgis Abate, Nasir Abbas, Abdallah H A Abd Al Magied, Samar Abd ElHafeez, Sherief Abd-Elsalam, Arash Abdollahi, Meriem Abdoun, Deldar Morad Abdulah, Rizwan Suliankatchi Abdulkader, Auwal Abdullahi, Hansani Madushika Abeywickrama, Alemwork Abie, Olumide Abiodun, Shady Abohashem, Dariush Abtahi, Hasan Abualruz, Bilyaminu Abubakar, Eman Abu-Gharbieh, Hana J Abukhadijah, Niveen ME Abu-Rmeileh, Salahdein Aburuz, Ahmed Abu-Zaid, Lisa C. Adams, Mesafint Molla Adane, Isaac Yeboah Addo, Kamoru Ademola Adedokun, Nurudeen A Adegoke, Ridwan Olamilekan Adesola, Juliana Bunmi Adetunji, Temitayo Esther Adeyeoluwa, Usha Adiga, Qorinah Estiningtyas Sakilah Adnani, Abdelrahman Yousry Afify, Aanuoluwapo Adeyimika Afolabi, Muhammad Sohail Afzal, Saira Afzal, Suneth Buddhika Agampodi, Shahin Aghamiri, César Agostinis Sobrinho, Williams Agyemang-Duah, Bright Opoku Ahinkorah, Austin J Ahlstrom, Aqeel Ahmad, Danish Ahmad, Fuzail Ahmad, Muayyad M Ahmad, Noah Ahmad, Sajjad Ahmad, Ayman Ahmed, Haroon Ahmed, Luai A Ahmed, Mehrunnisha Sharif Ahmed, Meqdad Saleh Ahmed, Syed Anees Ahmed, Marjan Ajami, Mohammed Ahmed Akkaif, Ashley E Akrami, Hanadi Al Hamad, Syed Mahfuz Al Hasan, Zain Al Ta'ani, Yazan Al Thaher, Tariq A Alalwan, Ziyad Al-Aly, Khurshid Alam, Rasmieh Mustafa Al-amer, Amani Alansari, Fahmi Y. Al-Ashwal, Mohammed Albashtawy, Bezawit Abeje Alemayehu, Abdelazeem M Algammal, Khalid F Alhabib, Dari Alhuwail, Abid Ali, Endale Alemayehu Ali, Mohammad Daud Ali, Mohammed Usman Ali, Rafat Ali, Waad Ali, Sheikh Mohammad Alif, Yousef Alimohamadi, Samah W Al-Jabi, Mohamad Aljofan, Syed Mohamed Aljunid, Ahmad Alkhatib, Wael Almahmeed, Sabah Al-Marwani, Mahmoud A Alomari, Saleh A Alqahtani, Abdullah A Alqarni, Ahmad Alrawashdeh, Intima Alrimawi, Sahel Majed Alrousan, Najim Z Alshahrani, Zaid Altaany, Awais Altaf, Farrukh Jawad Alvi, Nelson Alvis-Guzman, Mohammad Al-Wardat, Yaser Mohammed Al-Worafi, Hany Aly, Safwat Aly, Karem H Alzoubi, Masoud Aman Mohammadi, Tewodros Getnet Amera, Sohrab Amiri, Hubert Amu, Dickson A Amugsi, Ganiyu Adeniyi Amusa, Roshan A Ananda, Robert Ancuceanu, Mohammed Tahir Ansari, Sumbul Ansari, Boluwatife Stephen Anuoluwa, Iyadunni Adesola Anuoluwa, Saeid Anvari, Sumadi Lukman Anwar, Anayochukwu Edward Anyasodor, Juan Pablo Arab, Jalal Arabloo, Mosab Arafat, Aleksandr Y Aravkin, Demelash Areda, Brhane Berhe Aregawi, Hidayat Arifin, Benedetta Armocida, Johan Ärnlöv, Mahwish Arooj, Amit Arora, Anton A Artamonov, Kurnia Dwi Artanti, Ashokan Arumugam, Mohammad Asghari-Jafarabadi, Tahira Ashraf, Bernard Kwadwo Yeboah Asiamah-Asare, Thomas Astell-Burt, Seyyed Shamsadin Athari, Prince Atorkey, Alok Atreya, Zaure Maratovna Aumoldaeva, Mamaru Ayenew Awoke, Adedapo Wasiu Awotidebe, Setognal Birara Aychiluhm, Amirali Azimi, Sadat Abdulla Aziz, Shahkaar Aziz, Ahmed Y. Azzam, Domenico Azzolino, Mina Babashahi, Giridhara Rathnaiah Babu, Alaa Aboelnour Badran, Nasser Bagheri, Ruhai Bai, Atif Amin Baig, Shankar M Bakkannavar, Senthilkumar Balakrishnan, Ovidiu Constantin Baltatu, Kiran Bam, Rajon Banik, Shirin Barati, Mainak Bardhan, Hiba Jawdat Barqawi, Simon Barquera, Amadou Barrow, Lingkan Barua, Mohammad-Mahdi Bastan, Saurav Basu, Reza Bayat, Mulat Tirfie Bayih, Nebiyou Simegnew Bayleyegn, Narasimha M Beeraka, Priyamadhaba Behera, Diana Fernanda Bejarano Ramirez, Umar Muhammad Bello, Luis Belo, Derrick A Bennett, Maria Bergami, Kidanemaryam Berhe, Abiye Assefa Berihun, Ajeet Singh Bhadoria, Neeraj Bhala, Ravi Bharadwaj, Nikha Bhardwaj, Pankaj Bhardwaj, Sonu Bhaskar, Ajay Nagesh Bhat, Priyadarshini Bhattacharjee, Gurjit Kaur Bhatti, Jasvinder Singh Bhatti, Cem Bilgin, Catherine Bisignano, Bijit Biswas, Bruno Bizzozero Peroni, Espen Bjertness, Tone Bjørge, Archith Boloor, Sri Harsha Boppana, Samuel Adolf Bosoka, Souad Bouaoud, Edward J Boyko, Dejana Braithwaite, Javier Brazo-Sayavera, Hermann Brenner, Dana Bryazka, Raffaele Bugiardini, Linh Phuong Bui, Yasser Bustanji, Nadeem Shafique Butt, Zahid A Butt, Mehtap Çakmak Barsbay, Daniela Calina, Luis Alberto Cámera, Luciana Aparecida Campos, Si Cao, Angelo Capodici, Claudia Carletti, Andre F Carvalho, Márcia Carvalho, Monica Cattafesta, Maria Sofia Cattaruzza, Luca Cegolon, Francieli Cembranel, Ester Cerin, Achille Cernigliaro, Joshua Chadwick, Chiranjib Chakraborty, Eeshwar K Chandrasekar, Jung-Chen Chang, Vijay Kumar Chattu, Anis Ahmad Chaudhary, Akhilanand Chaurasia, An-Tian Chen, Haowei Chen, Nicholas WS Chew, Gerald Chi, Ritesh Chimoriya, Patrick R Ching, Abdulaal Chitheer, Dong-Woo Choi, Bryan Chong, Chean Lin Chong, Hitesh Chopra, Shivani Chopra, Hou In Chou, Sonali Gajanan Choudhari, Sheng-Chia Chung, Sunghyun Chung, Muhammad Chutiyami, Rebecca M Cogen, Alyssa Columbus, Nathalie Conrad, Michael H Criqui, Natalia Cruz-Martins, Alanna Gomes da Silva, Omid Dadras, Xiaochen Dai, Mayank Dalakoti, Emanuele D'Amico, Lalit Dandona, Rakhi Dandona, Lucio D'Anna, Pojsakorn Danpanichkul, Samuel Demissie Darcho, Reza Darvishi Cheshmeh Soltani, Nihar Ranjan Dash, Kairat Davletov, Azizallah Dehghan, Edgar Denova-Gutiérrez, Meseret Derbew Molla, Ismail Dergaa, Aragaw Tesfaw Desale, Vinoth Gnana Chellaiyan Devanbu, Devananda Devegowda, Arkadeep Dhali, Bibha Dhungel, Daniel Diaz, Monica Dinu, Thanh Chi Do, Camila Bruneli do Prado, Milad Dodangeh, Phidelia Theresa Doegah, Sushil Dohare, Klara Georgieva Dokova, Paul Narh Doku, Neda Dolatkhah, Mario D'Oria, Fariba - Dorostkar, Ojas Prakashbhai Doshi, Rajkumar Prakashbhai Doshi, Robert Kokou Dowou, Mi Du, Samuel C Dumith, Dorothea Dumuid, Bruce B Duncan, Sulagna Dutta, Alireza Ebrahimi, Kristina Edvardsson, Ashkan Eighaei Sedeh, Michael Ekholuenetale, Rabie Adel El Arab, Ibrahim Farahat El Bayoumy, Mohamed Ahmed Eladl, Said El-Ashker, Iffat Elbarazi, Islam Y Elgendy, Muhammed Elhadi, Ashraf A El-Metwally, Mohamed A Elmonem, Mohamed Hassan Elnaem, Randa Elsheikh, Chadi Eltaha, Theophilus I Emeto, Maysa Eslami, Natalia Fabin, Heidar Fadavian, Adeniyi Francis Fagbamigbe, Ildar Ravisovich Fakhradiyev, Seyed Nooreddin Faraji, Carla Sofia e Sá Farinha, MoezAlIslam Ezzat Mahmoud Faris, Pawan Sirwan Faris, Mohsen Farjoud Kouhanjani, Umar Farooque, Hossein Farrokhpour, Samuel Aanuoluwapo Fasusi, Patrick Fazeli, Timur Fazylov, Alireza Feizkhah, Ginenus Fekadu, Xiaoqi Feng, Rodrigo Fernandez-Jimenez, Nuno Ferreira, Nataliya A Foigt, Morenike Oluwatoyin Folayan, Artem Alekseevich Fomenkov, Roham Foroumadi, Celia Fortuna Rodrigues, Matteo Foschi, Kate Louise Francis, Richard Charles Franklin, Aleš Gába, Muktar A Gadanya, Abhay Motiramji Gaidhane, Yaseen Galali, Silvano Gallus, Balasankar Ganesan, Shivaprakash Gangachannaiah, Miglas Welay Gebregergis, Mesfin Gebrehiwot, Lemma Getacher, Molla Getie, Fataneh Ghadirian, Ramy Mohamed Ghazy, Artyom Urievich Gil, Tiffany K Gill, Richard F Gillum, Alem Abera Girmay, Mahaveer Golechha, Pouya Goleij, Alessandra C Goulart, Ayman Grada, Michal Grivna, Ashna Grover, Zhongyang Guan, Giovanni Guarducci, Mohammed Ibrahim Mohialdeen Gubari, Avirup Guha, Snigdha Gulati, Damitha Asanga Gunawardane, Zheng Guo, Bhawna Gupta, Rahul Gupta, Rajeev Gupta, Vipin Gupta, Roberth Steven Gutiérrez-Murillo, Jose Guzman-Esquivel, Najah R Hadi, Zahra Hadian, Nadia M Hamdy, Samer Hamidi, Asif Hanif, Nasrin Hanifi, Graeme J Hankey, Allie Haq, Josep Maria Haro, Faizul Hasan, Reza Hashempour, Mohammad Hashem Hashempur, Md Saquib Hasnain, Amr Hassan, Nageeb Hassan, Soheil Hassanipour, Afagh Hassanzade Rad, Rasmus J Havmoeller, Simon I Hay, Jeffrey J Hebert, Kamal Hezam, Yuta Hiraike, Mai Hoang, Ramesh Holla, Alamgir Hossain, Hassan Hosseinzadeh, Mihaela Hostiuc, Sorin Hostiuc, Zin Wai Htay, Mengsi Hu, Yifei Hu, Ayesha Humayun, Tsegaye Gebreyes Hundie, Mohamed Ibrahim Husseiny, Foziya Mohammed Hussien, Hong-Han Huynh, Bing-Fang Hwang, Ramzi Ibrahim, Anel Ibrayeva, Nayu Ikeda, Olayinka Stephen Ilesanmi, Irena M Ilic, Milena D Ilic, Leeberk Raja Inbaraj, Arit Inok, Khalid Iqbal, Md Sahidul Islam, Md. Fakrul Islam, Md. Rabiul Islam, Sheikh Mohammed Shariful Islam, Nahlah Elkudssiah Ismail, Hiroyasu Iso, Gaetano Isola, Mosimah Charles Ituka, Masao Iwagami, Chinwe Juliana Iwu-Jaja, Assefa N Iyasu, Louis Jacob, Shabbar Jaffar, Haitham Jahrami, Akhil Jain, Rajesh Jain, Ammar Abdulrahman Jairoun, Mihajlo Jakovljevic, Syed Sarmad Javaid, Sathish Kumar Jayapal, Shubha Jayaram, Felix K Jebasingh, Sun Ha Jee, Alelign Tasew Jema, Bijay Mukesh Jeswani, Jost B Jonas, Nitin Joseph, Charity Ehimwenma Joshua, Jacek Jerzy Jozwiak, Mikk Jürisson, Billingsley Kaambwa, Ali Kabir, Vidya Kadashetti, Ashish Kumar Kakkar, Sanjay Kalra, Saddam Fuad Kanaan, Samuel Berchi Kankam, Arun R Kanmanthareddy, Kehinde Kazeem Kanmodi, Rami S Kantar, Debasish Kar, Mehrdad Karajizadeh, Paschalis Karakasis, Arman Karimi Behnagh, Sahand Karimzadhagh, Nicholas J Kassebaum, Joonas H Kauppila, Gbenga A Kayode, Shemsu Kedir, Dimitrios Kehagias, Ariz Keshwani, Emmanuelle Kesse-Guyot, Mohammad Keykhaei, Himanshu Khajuria, Pantea Khalili, Alireza Khalilian, Mohamed Khalis, Ajmal Khan, Maseer Khan, Md Abdullah Saeed Khan, Mohammad Jobair Khan, Moien AB Khan, Muhammad Shahzeb Khan, Nusrat Khan, Vishnu Khanal, Shaghayegh Khanmohammadi, Moawiah Mohammad Khatatbeh, Masoomeh Kheirkhah, Feriha Fatima Khidri, Manoj Khokhar, Atulya Aman Khosla, Sepehr Khosravi, Mahmood Khosrowjerdi, Helda Khusun, Gyu Ri Kim, Jihee Kim, Jinho Kim, Min Seo Kim, Yun Jin Kim, Ruth W Kimokoti, Adnan Kisa, Ladli Kishore, Shivakumar KM, Michail Kokkorakis, Farzad Kompani, Oleksii Korzh, Karel Kostev, Sindhura Lakshmi Koulmane Laxminarayana, Irene Akwo Kretchy, Chong-Han Kua, Barthelemy Kuate Defo, Mohammed Kuddus, Mukhtar Kulimbet, Vishnutheertha Kulkarni, G Anil Kumar, Vijay Kumar, Satyajit Kundu, Setor K Kunutsor, Om P Kurmi, Maria Dyah Kurniasari, Dian Kusuma, Ville Kytö, Carlo La Vecchia, Ben Lacey, Chandrakant Lahariya, Daphne Teck Ching Lai, Iván Landires, Bagher Larijani, Zohra S Lassi, Huyen Thi Thanh Le, Nhi Huu Hanh Le, Hye Ah Lee, Munjae Lee, Paul H Lee, Seung Won Lee, Wei-Chen Lee, An Li, Ming-Chieh Li, Wei Li, Yongze Li, Stephen S Lim, Jialing Lin, Queran Lin, Daniel Lindholm, Paulina A Lindstedt, Jue Liu, Justin Lo, José Francisco López-Gil, Stefan Lorkowski, Giancarlo Lucchetti, Alessandra Lugo, Angelina M Lutambi, Zheng Feei Ma, Javier A Magaña Gómez, Nastaran Maghbouli, Mehrdad Mahalleh, Nozad H. Mahmood, Azeem Majeed, Konstantinos Christos C. Makris, Elaheh Malakan Rad, Reza Malekzadeh, Kashish Malhotra, Ahmad Azam Malik, Iram Malik, Deborah Carvalho Malta, Abdullah A Mamun, Emmanuel Manu, Hamid Reza Marateb, Mirko Marino, Abdoljalal Marjani, Ramon Martinez-Piedra, Miquel Martorell, Winfried März, Sammer Marzouk, Soroush Masrouri, Yasith Mathangasinghe, Fernanda Penido Matozinhos, Thushara Matthias, Rita Mattiello, Andrea Maugeri, Mohsen Mazidi, Steven M McPhail, Enkeleint A Mechili, María Paz Medel Salas, Asim Mehmood, Kamran Mehrabani-Zeinabad, Tesfahun Mekene Meto, Hadush Negash Meles, Walter Mendoza, Ritesh G Menezes, Emiru Ayalew Mengistie, Sultan Ayoub Meo, Tomislav Mestrovic, Chamila Dinushi Kukulege Mettananda, Sachith Mettananda, Huanhuan Miao, Ted R Miller, Wai-kit Ming, Erkin M Mirrakhimov, Awoke Misganaw, Habtamu Mitiku, Madhukar Mittal, Jama Mohamed, Mona Gamal Mohamed, Nouh Saad Mohamed, Taj Mohammad, Sakineh Mohammad-Alizadeh-Charandabi, Abdollah Mohammadian-Hafshejani, Ibrahim Mohammadzadeh, Shafiu Mohammed, Ali H Mokdad, Lorenzo Monasta, Stefania Mondello, Mohammad Ali Moni, Sara Montazeri Namin, AmirAli Moodi Ghalibaf, Yousef Moradi, Shane Douglas Morrison, Rohith Motappa, Sumaira Mubarik, Francesk Mulita, Erin C Mullany, Yanjinlkham Munkhsaikhan, Efren Murillo-Zamora, Christopher J L Murray, Sani Musa, Ghulam Mustafa, Sathish Muthu, Julius C Mwita, Woojae Myung, Abdulrazaq Bidemi Nafiu, Gabriele Nagel, Ganesh R Naik, Hiten Naik, Gopal Nambi, Vinay Nangia, Shumaila Nargus, Gustavo G Nascimento, Mahmoud Nassar, Javaid Nauman, Zakira Naureen, Samidi Nirasha Kumari Navaratna, Nawsherwan, Biswa Prakash Nayak, Athare Nazri-Panjaki, Masoud Negahdary, Ionut Negoi, Ruxandra Irina Negoi, Soroush Nematollahi, Samata Nepal, Henok Biresaw Netsere, Marie Ng, Josephine W Ngunjiri, Dang Nguyen, Phat Tuan Nguyen, Phuong The Nguyen, Robina Khan Niazi, Luciano Nieddu, Mahdieh Niknam, Taxiarchis Konstantinos Nikolouzakis, Ali Nikoobar, Jan Rene Nkeck, Shuhei Nomura, Syed Toukir Ahmed Noor, Mamoona Noreen, Jean Jacques Noubiap, Mehran Nouri, Chisom Adaobi Nri-Ezedi, Fred Nugen, Virginia Nuñez-Samudio, Aqsha Nur, Felix Kwasi Nyande, Chimezie Igwegbe Nzoputam, Bogdan Oancea, Erin M O'Connell, Ismail A Odetokun, Akinyemi O D Ofakunrin, James Odhiambo Oguta, In-Hwan Oh, Hassan Okati-Aliabad, Sylvester Reuben Okeke, Akinkunmi Paul Okekunle, Osaretin Christabel Okonji, Andrew T Olagunju, Oladotun Victor Olalusi, Tosin Abiola Olasehinde, Arão Belitardo Oliveira, Gláucia Maria Moraes Oliveira, Yinka Doris Oluwafemi, Hany A Omar, Ahmed Omar Bali, Nesredin Ahmed Omer, Sok King Ong, Michal Ordak, Alberto Ortiz, Augustus Osborne, Wael M S Osman, Adrian Otoiu, Abdu Oumer, Amel Ouyahia, Mayowa O Owolabi, Irene Amoakoh Owusu, Kolapo Oyebola, Tope Oyelade, Mahesh Padukudru P A, Alicia Padron-Monedero, Jagadish Rao Padubidri, Tamás Palicz, Sujogya Kumar Panda, Songhomitra Panda-Jonas, Anamika Pandey, Seithikurippu R Pandi-Perumal, Suman Pant, Shahina Pardhan, Utsav Parekh, Pragyan Paramita Parija, Romil R Parikh, Eun-Cheol Park, Roberto Passera, Jay Patel, Dimitrios Patoulias, Susan Paudel, Prince Peprah, Marcos Pereira, Norberto Perico, Simone Perna, Ionela-Roxana Petcu, Fanny Emily Petermann-Rocha, Hoang Nhat Pham, Tung Thanh Pham, Saeed Pirouzpanah, Roman V Polibin, Djordje S Popovic, Isabel Potani, Farzad Pourghazi, Akram Pourshams, Jalandhar Pradhan, Pranil Man Singh Pradhan, Manya Prasad, Akila Prashant, Elton Junio Sady Prates, Jagadeesh Puvvula, Ibrahim Qattea, Yanan Qiao, Venkatraman Radhakrishnan, Maja R Radojˇić, Catalina Raggi, Fryad Majeed Rahman, Md. Mosfequr Rahman, Mohammad Hifz Ur Rahman, Mosiur Rahman, Muhammad Aziz Rahman, Mohammad Rahmanian, Vahid Rahmanian, Masoud Rahmati, Rajesh Kumar Rai, Ivano Raimondo, Jeffrey Pradeep Raj, Prashant Rajput, Mahmoud Mohammed Ramadan, Chitra Ramasamy, Shakthi Kumaran Ramasamy, Sheena Ramazanu, Kritika Rana, Chhabi Lal Ranabhat, Mithun Rao, Sowmya J Rao, Sina Rashedi, Mohammad-Mahdi Rashidi, Ashkan Rasouli-Saravani, Devarajan Rathish, Santosh Kumar Rauniyar, Ilari Rautalin, David Laith Rawaf, Salman Rawaf, Elrashdy M. Moustafa Mohamed Redwan, Sanika Rege, Aqeeb Ur Rehman, Ana Reis-Mendes, Giuseppe Remuzzi, Nazila Rezaei, Mohsen Rezaeian, Taeho Gregory Rhee, João Rocha Rocha-Gomes, Thales Philipe Rodrigues da Silva, Jefferson Antonio Buendia Rodriguez, Leonardo Roever, Peter Rohloff, Debby Syahru Romadlon, Moustaq Karim Khan Rony, Gholamreza Roshandel, Himanshu Sekhar Rout, Nitai Roy, Godfrey M Rwegerera, Aly M A Saad, Maha Mohamed Saber-Ayad, Leila Sabzmakan, Kabir P Sadarangani, Basema Ahmad Saddik, Masoumeh Sadeghi, Umar Saeed, Dominic Sagoe, Fatemeh Saheb Sharif-Askari, Amirhossein Sahebkar, Soumya Swaroop Sahoo, S. Mohammad Sajadi, Mirza Rizwan Sajid, Afeez Abolarinwa Salami, Luciane B Salaroli, Samreen Saleem, Mohamed A Saleh, Marwa Rashad Salem, Dauda Salihu, Sohrab Salimi, Abdallah M Samy, Milena M Santric-Milicevic, Tanmay Sarkar, Mohammad Sarmadi, Gargi Sachin Sarode, Sachin C Sarode, Michele Sassano, Jennifer Saulam, Monika Sawhney, Sonia Saxena, Ganesh Kumar Saya, Christophe Schinckus, Maria Inês Schmidt, Art Schuermans, Siddharthan Selvaraj, Ashenafi Kibret Sendekie, Pallav Sengupta, Yigit Can Senol, Subramanian Senthilkumaran, Sadaf G Sepanlou, Yashendra Sethi, Allen Seylani, Mahan Shafie, Sweni Shah, Samiah Shahid, Moyad Jamal Shahwan, Muhammad Aaqib Shamim, Mehran Shams-Beyranvand, Alfiya Shamsutdinova, Mohd Shanawaz, Mohammed Shannawaz, Medha Sharath, Amin Sharifan, Manoj Sharma, Ujjawal Sharma, Vishal Sharma, Fateme Sheida, Rekha Raghuveer Shenoy, Pavanchand H Shetty, Desalegn Shiferaw, Min-Jeong Shin, Mahsa Shirani Lapari, Rahman Shiri, Aminu Shittu, Sina Shool, Seyed Afshin Shorofi, Gambhir Shrestha, Rajan Shrestha, Kerem Shuval, Yafei Si, Nicole R S Sibuyi, Emmanuel Edwar Siddig, Mario Siervo, Diego Augusto Santos Silva, Luís Manuel Lopes Rodrigues Silva, Amit Singh, Baljinder Singh, Harmanjit Singh, Jasvinder A Singh, Kalpana Singh, Lucky Singh, Mitasha Singh, Poornima Suryanath Singh, Surjit Singh, Anna Aleksandrovna Skryabina, Amanda E Smith, Georgia Smith, Sameh S M Soliman, Soroush Soraneh, Michael Spartalis, Bahadar S Srichawla, Muhammad Haroon Stanikzai, Antonina V Starodubova, Kurt Straif, Pete Stubbs, Vetriselvan Subramaniyan, Muritala Odidi Suleiman Odidi, Aleksander Sulkowski, Anusha Sultan Meo, Zhong Sun, Sumam Sunny, Dev Ram Sunuwar, Chandan Kumar Swain, Lukasz Szarpak, Sree Sudha T Y, Rafael Tabarés-Seisdedos, Seyyed Mohammad Tabatabaei, Ozra Tabatabaei Malazy, Seyed-Amir Tabatabaeizadeh, Shima Tabatabai, Celine Tabche, Mohammad Tabish, Jabeen Taiba, Mircea Tampa, Jacques Lukenze Tamuzi, Ker-Kan Tan, Manoj Tanwar, Saba Tariq, Nathan Y Tat, Mohamad-Hani Temsah, Reem Mohamad Hani Temsah, Masayuki Teramoto, Dufera Rikitu Terefa, Jay Tewari, Pugazhenthan Thangaraju, Rekha Thapar, Aravind Thavamani, Sathish Thirunavukkarasu, Joe Thomas, Sofonyas Abebaw Tiruneh, Tenaw Yimer Tiruye, Mariya Vladimirovna Titova, Krishna Tiwari, Sojit Tomo, Marcello Tonelli, Mathilde Touvier, Marcos Roberto Tovani-Palone, Khaled Trabelsi, Ngoc Ha Tran, Thang Huu Tran, Nguyen Tran Minh Duc, Domenico Trico, Thien Tan Tri Tai Truyen, Guesh Mebrahtom Tsegay, Munkhtuya Tumurkhuu, Sok Cin Tye, Aniefiok John Udoakang, Atta Ullah, Saeed Ullah, Shahid Ullah, Muhammad Umair, Lawan Umar, Umar Muhammad Umar, Brigid Unim, Dinesh Upadhya, Era Upadhyay, Jibrin Sammani Usman, Damla Ustunsoz, Masoud Vaezghasemi, Asokan Govindaraj Vaithinathan, Jef Van den Eynde, Joe Varghese, Tommi Juhani Vasankari, Siavash Vaziri, Balachandar Vellingiri, Narayanaswamy Venketasubramanian, Madhur Verma, Georgios-Ioannis Verras, Victor E Villalobos-Daniel, Sergey Konstantinovitch Vladimirov, Vasily Vlassov, Stein Emil Vollset, Rade Vukovic, Mohammad Wahiduzzaman, Cong Wang, Shu Wang, Xingxin Wang, Yanzhong Wang, Kosala Gayan Weerakoon, Fei-Long Wei, Anggi Lukman Wicaksana, Dakshitha Praneeth Wickramasinghe, Nuwan Darshana Wickramasinghe, Peter Willeit, Marcin W Wojewodzic, Qing Xia, Guangqin Xiao, Wanqing Xie, Suowen Xu, Xiaoyue Xu, Galal Yahya, Kazumasa Yamagishi, Yuichiro Yano, Haiqiang Yao, Amir Yarahmadi, Habib Yaribeygi, Pengpeng Ye, Subah Abderehim Yesuf, Dehui Yin, Dong Keon Yon, Naohiro Yonemoto, Chuanhua Yu, Chun-Wei Yuan, Deniz Yuce, Ismaeel Yunusa, Giulia Zamagni, Michael Zastrozhin, Mohammed G M Zeariya, Casper J P Zhang, Haijun Zhang, Jingya Zhang, Liqun Zhang, Xiaoyi Zhang, Zhiqiang Zhang, Hanqing Zhao, David X Zheng, Anthony Zhong, Claire Chenwen Zhong, Jiayan Zhou, Bin Zhu, Abzal Zhumagaliuly, Magdalena Zielińska, Osama A Zitoun, Ghazal Zoghi, Zhiyong Zou, Sa'ed H Zyoud, Emmanuela Gakidou, Susan M Sawyer, Peter S Azzopardi

## Abstract

**Background:**

Despite the well documented consequences of obesity during childhood and adolescence and future risks of excess body mass on non-communicable diseases in adulthood, coordinated global action on excess body mass in early life is still insufficient. Inconsistent measurement and reporting are a barrier to specific targets, resource allocation, and interventions. In this Article we report current estimates of overweight and obesity across childhood and adolescence, progress over time, and forecasts to inform specific actions.

**Methods:**

Using established methodology from the Global Burden of Diseases, Injuries, and Risk Factors Study 2021, we modelled overweight and obesity across childhood and adolescence from 1990 to 2021, and then forecasted to 2050. Primary data for our models included 1321 unique measured and self-reported anthropometric data sources from 180 countries and territories from survey microdata, reports, and published literature. These data were used to estimate age-standardised global, regional, and national overweight prevalence and obesity prevalence (separately) for children and young adolescents (aged 5–14 years, typically in school and cared for by child health services) and older adolescents (aged 15–24 years, increasingly out of school and cared for by adult services) by sex for 204 countries and territories from 1990 to 2021. Prevalence estimates from 1990 to 2021 were generated using spatiotemporal Gaussian process regression models, which leveraged temporal and spatial correlation in epidemiological trends to ensure comparability of results across time and geography. Prevalence forecasts from 2022 to 2050 were generated using a generalised ensemble modelling approach assuming continuation of current trends. For every age-sex-location population across time (1990–2050), we estimated obesity (*vs* overweight) predominance using the log ratio of obesity percentage to overweight percentage.

**Findings:**

Between 1990 and 2021, the combined prevalence of overweight and obesity in children and adolescents doubled, and that of obesity alone tripled. By 2021, 93·1 million (95% uncertainty interval 89·6–96·6) individuals aged 5–14 years and 80·6 million (78·2–83·3) aged 15–24 years had obesity. At the super-region level in 2021, the prevalence of overweight and of obesity was highest in north Africa and the Middle East (eg, United Arab Emirates and Kuwait), and the greatest increase from 1990 to 2021 was seen in southeast Asia, east Asia, and Oceania (eg, Taiwan [province of China], Maldives, and China). By 2021, for females in both age groups, many countries in Australasia (eg, Australia) and in high-income North America (eg, Canada) had already transitioned to obesity predominance, as had males and females in a number of countries in north Africa and the Middle East (eg, United Arab Emirates and Qatar) and Oceania (eg, Cook Islands and American Samoa). From 2022 to 2050, global increases in overweight (not obesity) prevalence are forecasted to stabilise, yet the increase in the absolute proportion of the global population with obesity is forecasted to be greater than between 1990 and 2021, with substantial increases forecast between 2022 and 2030, which continue between 2031 and 2050. By 2050, super-region obesity prevalence is forecasted to remain highest in north Africa and the Middle East (eg, United Arab Emirates and Kuwait), and forecasted increases in obesity are still expected to be largest across southeast Asia, east Asia, and Oceania (eg, Timor-Leste and North Korea), but also in south Asia (eg, Nepal and Bangladesh). Compared with those aged 15–24 years, in most super-regions (except Latin America and the Caribbean and the high-income super-region) a greater proportion of those aged 5–14 years are forecasted to have obesity than overweight by 2050. Globally, 15·6% (12·7–17·2) of those aged 5–14 years are forecasted to have obesity by 2050 (186 million [141–221]), compared with 14·2% (11·4–15·7) of those aged 15–24 years (175 million [136–203]). We forecasted that by 2050, there will be more young males (aged 5–14 years) living with obesity (16·5% [13·3–18·3]) than overweight (12·9% [12·2–13·6]); while for females (aged 5–24 years) and older males (aged 15–24 years), overweight will remain more prevalent than obesity. At a regional level, the following populations are forecast to have transitioned to obesity (*vs* overweight) predominance before 2041–50: children and adolescents (males and females aged 5–24 years) in north Africa and the Middle East and Tropical Latin America; males aged 5–14 years in east Asia, central and southern sub-Saharan Africa, and central Latin America; females aged 5–14 years in Australasia; females aged 15–24 years in Australasia, high-income North America, and southern sub-Saharan Africa; and males aged 15–24 years in high-income North America.

**Interpretation:**

Both overweight and obesity increased substantially in every world region between 1990 and 2021, suggesting that current approaches to curbing increases in overweight and obesity have failed a generation of children and adolescents. Beyond 2021, overweight during childhood and adolescence is forecast to stabilise due to further increases in the population who have obesity. Increases in obesity are expected to continue for all populations in all world regions. Because substantial change is forecasted to occur between 2022 and 2030, immediate actions are needed to address this public health crisis.

**Funding:**

Bill & Melinda Gates Foundation and Australian National Health and Medical Research Council.

## Introduction

Excess body mass is now well established as a leading modifiable risk factor for death and disability-adjusted life-years globally.^1^, ^2^ Although overweight in early life is often framed as a risk for future health, obesity is increasingly considered a complex chronic disease^3^ that has immediate impacts on child and adolescent physical and mental health and causes serious disease and dysfunction before adulthood (eg, metabolic-associated fatty liver disease, hypertension, diabetes, and ovulatory or endometrial dysfunction).^4^, ^5^, ^6^, ^7^, ^8^, ^9^, ^10^, ^11^ Beyond the substantial disease-related impacts, obesity also has crippling societal impacts, with the total economic impact of overweight and obesity estimated to exceed 3% of the world's gross domestic product by 2060.^12^ Once obesity is established, it is difficult for children and adolescents to return to normal weight.^13^, ^14^, ^15^, ^16^, ^17^ Indeed, obesity rarely resolves after adolescence,^14^, ^16^, ^17^ and further risks develop in adulthood, including infertility, cancer, cardiovascular diseases, and diseases of the liver and kidneys.^1^, ^5^, ^18^, ^19^, ^20^, ^21^, ^22^ Although population-level normal weight rather than overweight should be the ultimate goal, because obesity is associated with greater disease burden than overweight,^16^ it is important to pinpoint population groups that predominantly have overweight and those that predominantly have obesity (ie, the majority of the population has transitioned from overweight to obesity [obesity transition]), as each of these health states signals the need for a different balance of public health and clinical responses.

**Research in context**

**Evidence before this study**

Accurate data on obesity transitions are required to inform effective policy and programming. To review the literature focused on the past and future epidemiology of child and adolescent overweight and obesity, we searched Ovid MEDLINE, PubMed, and the grey literature for articles published to April 30, 2024, with no language or year restrictions. We used the terms “overweight and obese” and “prevalence or epidemiology” and “forecasting or projection” and “children, adolescents, and young adults”. Five global studies reported on overweight or obesity prevalence and forward projections for children and adolescents. One study focused on preschool-aged children (aged 0–5 years), and two focused on school-aged children and adolescents (aged 5–17 years), of which one reported projected prevalence estimates for 5–17-year-olds from 20 countries to 2025, and the other reported estimates for five world regions to 2010: the Americas, eastern Mediterranean, Europe, southeast Asia, and western Pacific. One study published in 2022 included an appendix table that reported global prevalence estimates (161 countries) for children and adolescents younger than 20 years with projections to 2060, but without age-disaggregated prevalence results. A further article from the grey literature (the World Obesity Atlas) used many of the same estimates to report prevalence and economic impact for 187 countries to 2035, with estimates for children and adolescents aged 5–19 years. We also found a limited number of multicountry comparisons (with fewer than ten countries), with estimates that were limited by a narrow age window (or that did not disaggregate by age within a wider period). Only one article reported separate estimates (for two countries) showing the proportion of not overweight, overweight, and obesity across the life course to 2030. Articles focused on single-country prevalence and projections were more likely to present some disaggregation for age, socioeconomic status, or urban or rural location, but separate estimates for overweight and obesity were uncommon. While there are efforts to report contemporary global estimates for overweight or obesity from the NCD Risk Factor Collaboration, WHO's Global Health Observatory, and previous iterations of the Global Burden of Diseases, Injuries, and Risk Factors Study (GBD), there are still important gaps in knowledge across the developmental window from age 5 years to 24 years. Previous global studies have not analysed age-disaggregated results for children and adolescents, nor have they interrogated the transition from overweight to obesity predominance for this age group. Most notably absent from the literature are age-disaggregated country-level forecasts for overweight prevalence and obesity prevalence that are necessary to inform targeted action. Despite limitations, the available literature describes a global epidemic among children and adolescents that requires urgent attention.

**Added value of this study**

Using established methods from GBD 2021, in both 10-year and 5-year age bands between ages 5 years and 24 years, we report the estimated prevalence of overweight and of obesity for 204 countries and territories, from 1990 to 2021, with forecasts to 2050. We do this under a reference scenario representing a probabilistic forecast of the most likely future. To our knowledge, this is the most comprehensive forecasting study on global, regional, and national overweight prevalence and obesity prevalence in individuals aged 5–24 years to 2050. Because public health actions differ significantly between developmental periods, we summarise results for those aged 5–14 years compared with those aged 15–24 years. Unlike other global reports, we pinpoint populations with current and forecasted overweight predominance compared with obesity predominance. This is important because while populations that remain overweight-predominant can be targeted with preventive interventions, adolescent populations that are expected to transition to obesity predominance need to be targeted with both preventive interventions and clinical management strategies. GBD 2021 advances previous GBD estimates for all risk factors including high BMI, with inclusion of 189 new population-representative data sources (1990–2021) for BMI, overweight, or obesity. GBD 2021 included an updated set of model covariates (eg, age-standardised educational attainment) to better capture the relationship between socioeconomic development and overweight and obesity. Compared with other global reports, this study included more primary data sources, including self-reported as well as measured height and weight, and employed a sex-specific and region-specific novel meta-regression approach to reconcile differences between these anthropometric assessments. This study allows us to make novel investigations into the specific trajectories for countries and regions throughout the world and identify priority populations (age group and location) for intervention and prevention across this developmental window.

**Implications of all the available evidence**

Children and adolescents remain a vulnerable population within the obesity epidemic. To effectively set global and national targets for child and adolescent obesity beyond the maturation of the Sustainable Development Goals (SDGs) in 2030, we provide decision makers access to contemporary and future prevalence estimates to understand which regions and population subgroups will require intervention and clinical management strategies and which can still be primarily targeted with prevention strategies. We forecast that by 2050, 360 million children and adolescents (aged 5–24 years) will be living with obesity, a highly complex disease. This magnitude of burden will not only incur substantial costs for health and economic systems, but obesity comorbidities will negatively affect these children and adolescents for decades to come, including intergenerational effects on their offspring. Although these findings indicate monumental failures in the management of overweight and obesity across the entire developmental window, our results also provide optimism that a complete transition to global obesity can be avoided if action comes now, before 2030. We pinpoint several population subgroups that are forecasted to remain overweight-predominant that should be targeted with obesity prevention strategies (eg, much of Europe and central and south Asia, among many other regions), compared with those subgroups forecasted to become obesity-predominant that require urgent interventions and clinical treatment. Multifaceted and multisectoral interventions and treatments are now urgent for many populations in north Africa and the Middle East, Latin America, high-income North America, Australasia, and Oceania. Within these regions, adolescent girls entering their reproductive years with obesity are a priority population if we are to avoid intergenerational transmission of obesity, non-communicable diseases, and the dire financial and societal costs across future generations. 5-year action plans, for 2025–30, are urgently required to shift these forecasts and inform goal setting in the post-SDG era.

The previously set WHO 2025 obesity target—no increase between 2010 and 2025—has already been missed by most countries.^23^ The NCD Risk Factor Collaboration (NCD-RisC) provides the most recent global estimates for individuals aged 5–19 years, reporting that 9·3% of boys and 6·9% of girls had obesity in 2022, equating to 159 million school-aged children and adolescents.^24^ However, despite this and other reports describing the change in obesity epidemiology and obesity transitions over time,^24^, ^25^, ^26^ few reports disaggregate estimates across childhood and adolescence, or routinely disaggregate overweight from obesity. Granular data are essential for targeted advocacy, policy, and service responses. Interventions for children and young adolescents typically need to be targeted at school, at home, and in the community, whereas older adolescents typically need to be targeted outside of school. Disaggregation is also important to effectively target at-risk groups (eg, adolescent females and males entering reproductive age) to prevent intergenerational obesity transmission.^27^, ^28^, ^29^, ^30^, ^31^

Contemporary estimates of child and adolescent overweight and obesity are essential to prioritise immediate resources.^1^, ^24^, ^32^, ^33^, ^34^, ^35^, ^36^ Although obesity has been a part of previous global action plans,^37^, ^38^, ^39^, ^40^ strategies have not been universally supported or adopted across settings.^41^, ^42^ Of note, the UN Sustainable Development Goals (SDGs) for 2015–30 do not include any specific targets for overweight and obesity.^43^ Forecasted estimates can help to inform new goals and targets in the post-SDG era. Important considerations for future target setting include understanding how quickly and when rates of obesity might rise in any given setting in the future, and when populations are forecasted to tip over to obesity predominance. To inform effective policy, it is also important that estimates relate to policy-relevant age groups, such as for schoolchildren and for adolescents entering their reproductive years. While past efforts have been made to forecast child and adolescent obesity,^12^, ^44^, ^45^, ^46^, ^47^ Global Burden of Diseases, Injuries, and Risk Factors Study (GBD) forecasting models are more comprehensive because they forecast estimates for more locations across longer time periods, and use past trends together with the composite covariate, Socio-demographic Index (SDI), which captures the socioeconomic and demographic development of a country, to inform future trend forecasts (appendix 1 pp 11–12).

In this Article, we provide a comprehensive analysis of past and present global, regional, and national shifts in overweight prevalence and obesity prevalence, separately and together, for 204 countries and territories from 1990 to 2021 using data from 180 locations in GBD 2021. We provide estimates for policy-relevant childhood or young adolescent (5–14 years) and older adolescent (15–24 years) age bands. In extending previous global analyses, we have an opportunity to inform the post-SDG era from 2030 to 2050 by characterising the current and the future epidemic through forecasting the prevalence and overweight versus obesity predominance if current trends were to persist. This paper was produced as part of the GBD Collaborator Network and in accordance with the GBD Protocol.^48^

## Methods

### Overview

Leveraging results from GBD 2021,^2^ we estimated the prevalence of overweight, obesity, and overweight and obesity combined in children and adolescents aged 5–24 years^49^ for males and females separately for 204 countries and territories from 1990 to 2021, forecasting prevalence from 2022 to 2050. Prevalence was stratified in 5-year age brackets (5–9, 10–14, 15–19, and 20–24 years) and by sex for all locations. This wide age band for adolescence (10–24 years) aligns with new understandings of adolescent development and reflects changes in biological growth and social expectations that are highly relevant to current societies.^27^, ^49^, ^50^ We defined overweight and obesity using BMI (mass divided by the square of height, in kg/m^2^). For adolescents aged 18–24 years, overweight was defined as a BMI from 25·0 kg/m^2^ to less than 30·0 kg/m^2^, and obesity was defined as a BMI of 30·0 kg/m^2^ or higher. For children and adolescents aged 5–17 years, these classifications were based on the International Obesity Task Force (IOTF) criteria.^51^ Derived from surveys covering multiple countries, races, and ethnicities, these sex-age-specific cut points provide consistent standards for overweight and obesity in children and adolescents younger than 18 years. Specifically, the IOTF cut points were derived from sex-specific BMI-for-age curves that intercept with a BMI of 25 kg/m^2^ for overweight and a BMI of 30 kg/m^2^ for obesity at age 18 years, and therefore correspond to overweight and obesity status in the population of older adolescents and adults, which are known indicators of health risk.^52^ We note that BMI is not a perfect measure of individual-level health or disease risk, which depends on various factors (eg, ethnicity^53^, ^54^), but it is deemed an acceptable measure for large-scale monitoring of population-level risk.^25^ Further details on the definition of overweight and obesity can be found in appendix 1 (pp 3–4).

This study complies with the GATHER statement (appendix 1 p 38).^55^ Analyses were completed with R version 4.4.0 and Python version 3.10. All codes used in the analysis are available upon request.

### Data sources

We identified population-representative data on BMI, overweight, and obesity through a systematic search through the Global Health Data Exchange (GHDx; appendix 1 p 3). We included both self-reported and directly measured heights, weights, and BMI obtained from survey data at the national level, as well as at the subnational (state or province) level for 20 countries (including China, the USA, and Brazil; appendix 1 p 3). We excluded data if based on non-random samples or data limited to specific subpopulations that were not representative of the general population. We also excluded sample sizes of less than 20 per 5-year age-sex group or data based on alternative measures such as waist-to-hip ratio and waist circumference, because conversion to equivalent BMI-based prevalence estimates is unreliable (see appendix 1 p 3 for details on full inclusion and exclusion criteria). In total, exclusions resulted in the removal of data associated with 1 463 615 individuals (8·4%) aged 5–24 years.

We included 1321 data sources from 180 countries and territories that provided relevant data among children and adolescents covering the period from 1990 to 2021. Of these 1321 unique data sources, 743 were measured and 578 were based on self-report. Sources included multicountry survey programmes (eg, Demographic Health Surveys [DHS], the UNICEF Multiple Indicator Cluster Surveys, the WHO STEPwise Approach to Surveillance programme [STEPS], European Health Interview Survey, the EU Eurobarometer Surveys, Health Behaviour in School-aged Children Survey), national surveys (eg, the US National Health and Nutrition Examination Survey, US Behavioural Risk Factor Survellance System, Australian National Health Survey, South Korea National Health and Nutrition Examination Survey), and longitudinal population-representative studies (eg, Living Standards Measurement Study, Understanding the Lives of Adolescents and Young Adults in Bihar and Uttar Pradesh). The complete list of data sources is accessible via the GHDx GBD 2021 Sources Tool. From all sources, we extracted individual-level microdata or tabulated reports, excluding any data with sample sizes less than ten. Finally, we conducted rigorous quality checks to eliminate implausible data entries (eg, BMI >80 kg/m^2^), duplications, or inconsistencies. Additional information on the search strategy, inclusion criteria, and data extraction process is also available in previous GBD publications^1^, ^34^ and in appendix 1 (p 3).

### Data standardisation

Objectively measured height and weight data are considered the gold standard, but self-reported data were still included in this report. Self-report measurements can underestimate height and weight, and biases can vary by race and ethnicity, age, and sex.^56^, ^57^, ^58^ However, because there is reasonable correlation between self-report and measured data, self-report data are still recommended for use in large epidemiological studies, granted that sources of bias are addressed.^59^ Indeed, reasonable convergence has been found in previous studies using models to correct self-report bias.^34^, ^60^, ^61^, ^62^, ^63^ Therefore, in contrast to previous GBD publications, to correct for potential biases, we used an updated and more robust method to adjust the self-reported height and weight data. This novel method was based on meta-regression—Bayesian, regularised, trimmed (MR-BRT), and used datasets that provided both self-reported and measured data.^64^ Although we acknowledge that some bias might remain, we developed sex-specific and region-specific MR-BRT models to estimate bias-correction factors, which were then subsequently applied to self-reported prevalence data from adolescents aged 15–24 years. Corrections were not made for prevalence data from those aged 5–14 years, as these data were all measured data. Details of the bias correction method can be found in appendix 1 (pp 7–8).

Overall adjustments were also made to standardise data that were reported in intervals that differed from GBD conventions (ie, 5–9 years, 10–14 years, 15–19 years, and 20–24 years). We derived an age-sex splitting model^1^, ^34^ using available survey microdata to estimate the underlying age-sex structure, then redistributed aggregated prevalence values into the specific 5-year age and sex categories according to the estimated structure (appendix 1 p 7).

### Estimation of overweight and obesity prevalence 1990 to 2021

As applied in other studies,^1^, ^34^, ^65^ we used spatiotemporal Gaussian process regression (ST-GPR) to generate a retrospective time series for the prevalence of overweight and obesity by age, sex, and year for each location from 1990 to 2021 (appendix 1 p 10). A linear regression model was used to estimate mean function of ST-GPR based on a set of covariates (eg, urbanicity). Compared with previous GBD iterations, we updated the covariate set, including age-standardised educational attainment level and the proportion of the population working in agriculture. This new covariate set helps to capture the relationship between socioeconomic development and overweight and obesity.^66^, ^67^, ^68^ Given the unique magnitude of the obesity epidemic in the USA, and to maximise the use of available data in the USA, a separate self-report bias adjustment was completed for this country. Specifically, prevalence estimation was done separately using national-level and state-level data. The USA-specific results were subsequently corroborated and combined with the global findings.^69^ The 95% uncertainty intervals (UIs) for the final estimates were derived from the 2·5th and 97·5th percentiles of 1000 draws from the posterior distribution of ST-GPR. Further detail relevant to all methods can be found in appendix 1 (pp 3–37) and past GBD publications.^1^, ^34^ Obesity (versus overweight) predominance in a region, country, or population is considered present when there is more obesity than there is overweight, signalled by the log ratio of obesity percentage to overweight percentage exceeding zero. Obesity transition is defined as having shifted to obesity versus overweight predominance.

### Forecasting overweight and obesity prevalence from 2022 to 2050

We forecasted estimates for a reference scenario, a probabilistic forecast based on past trends. Using prevalence estimates from 1990 to 2021 as inputs, we applied a generalised ensemble modelling approach to forecast the future prevalence of overweight and obesity from 2022 to 2050 (appendix 1 pp 11–12). This approach draws on the predictive strength of 12 submodels, six of which were annualised rate-of-change models with distinctive recency weights to put varying emphasis on recent year-over-year trends. The other six submodels employed a two-stage MR-BRT spline model with varying specifications, using SDI as a covariate.^70^, ^71^ Forecasted obesity prevalence was estimated by multiplying forecasted overweight and obesity prevalence by the forecasted proportion of obesity among the population with overweight for each draw. The accuracy of the forecast results and the selection of the best models were based on cross-validation methods across a 10-year holdout period from 2012 to 2021. Full details are described in previous publications^70^, ^71^ and in appendix 1 (pp 11–12).

### Role of the funding source

The funders of the study had no role in study design, data collection, data analysis, data interpretation, or writing of the report.

## Results

### Total prevalence of overweight and obesity across childhood and adolescence

In 2021, 18·1% (95% UI 17·5–18·7) of individuals aged 5–14 years and 20·3% (19·7–21·0) of those aged 15–24 years were living with overweight or obesity across the world, equating to 493 million (483–505) young people (Table 1, Table 2; appendix 1 pp 45–73). This represents a doubling of prevalence since 1990, when it was 8·8% (8·5–9·1) for individuals aged 5–14 years and 9·9% (9·6–10·1) for those aged 15–24 years. Across this same timeframe, the prevalence of obesity tripled from 2·0% (1·9–2·0) in 1990 to 6·8% (6·6–7·0) in 2021 for both age groups combined (244·0% increase [229·5–258·8]), affecting a total of 174 million (169–178) children and adolescents in 2021 (93·1 million [89·6–96·6] aged 5–14 years and 80·6 million [78·2–83·3] aged 15–24 years).

**Table 1:** Age-standardised prevalence, percentage change, and number of children aged 5–14 years with overweight and obesity in 1990, 2021, 2030, 2050, globally and within super-regions

	**Overweight (not obesity) among individuals aged 5–14 years**				**Obesity among individuals aged 5–14 years**			
	Prevalence, %	Estimated mean number	Observed relative change in prevalence, 1990–2021, %	Forecasted relative change in prevalence, 2021–50, %	Prevalence, %	Estimated mean number	Observed relative change in prevalence, 1990–2021, %	Forecasted relative change in prevalence, 2021–50, %
**Global**								
1990	6·7% (6·5 to 7·0)	75 243 389 (72 341 825 to 78 289 161)	..	..	2·0% (1·9 to 2·2)	22 942 921 (21 742 500 to 24 258 536)	..	..
2021	11·2% (10·8 to 11·6)	151 568 150 (146 112 286 to 157 518 737)	66·0% (57·2 to 74·8)	..	6·9% (6·6 to 7·2)	93 086 637 (89 574 689 to 96 614 458)	236·9% (213·5 to 259·3)	..
2030	12·2% (11·7 to 12·8)	158 595 891 (151 005 504 to 167 389 927)	..	..	9·1% (8·4 to 9·7)	115 968 998 (105 692 721 to 124 623 970)	..	..
2050	14·1% (13·4 to 14·8)	169 814 584 (149 896 591 to 191 865 418)	..	26·2% (21·3 to 29·7)	15·6% (12·7 to 17·2)	185 686 624 (141 316 168 to 220 657 573)	..	126·0% (85·6 to 147·3)
**Central Europe, eastern Europe, and central Asia**								
1990	10·1% (9·4 to 10·8)	7 023 174 (6 568 076 to 7 510 363)	..	..	3·4% (3·1 to 3·8)	2 385 177 (2 183 778 to 2 605 458)	..	..
2021	14·7% (13·4 to 16·0)	8 063 313 (7 371 161 to 8 821 422)	45·7% (32·0 to 62·0)	..	7·6% (6·8 to 8·5)	4 149 156 (3 717 097 to 4 634 258)	121·0% (92·2 to 152·7)	..
2030	15·7% (14·4 to 17·1)	7 808 964 (6 993 044 to 8 703 181)	..	..	8·7% (7·8 to 9·9)	4 236 425 (3 680 184 to 4 838 256)	..	..
2050	17·6% (15·6 to 19·5)	7 547 670 (6 409 391 to 8 748 636)	..	20·5% (8·9 to 28·3)	12·1% (9·8 to 14·2)	5 138 219 (3 992 211 to 6 197 182)	..	60·5% (33·8 to 80·6)
**High income**								
1990	12·8% (11·8 to 13·9)	16 090 998 (14 842 130 to 17 508 108)	..	..	5·2% (4·6 to 6·0)	6 552 809 (5 779 504 to 7 517 428)	..	..
2021	18·2% (17·1 to 19·3)	22 368 084 (21 028 360 to 23 726 088)	42·7% (27·8 to 57·6)	..	12·0% (11·1 to 12·9)	14 620 117 (13 529 456 to 15 809 342)	129·7% (93·6 to 168·0)	..
2030	19·0% (17·6 to 20·3)	21 355 633 (19 721 377 to 23 108 751)	..	..	14·2% (13·0 to 15·4)	15 841 741 (14 278 097 to 17 329 138)	..	..
2050	20·2% (18·4 to 21·6)	21 463 442 (18 982 913 to 24 257 458)	..	11·4% (5·5 to 15·1)	19·8% (16·8 to 22·1)	20 824 688 (17 045 368 to 24 145 394)	..	65·1% (43·5 to 78·8)
**Latin America and Caribbean**								
1990	11·5% (10·4 to 12·7)	10 850 890 (9 862 444 to 11 967 425)	..	..	3·2% (2·8 to 3·7)	3 049 113 (2 672 867 to 3 478 577)	..	..
2021	18·2% (16·9 to 19·7)	17 514 858 (16 215 190 to 18 898 194)	59·3% (42·0 to 80·7)	..	11·8% (10·6 to 13·0)	11 225 995 (10 161 933 to 12 393 376)	265·4% (207·8 to 325·3)	..
2030	19·3% (17·9 to 20·8)	17 972 529 (16 381 873 to 19 735 605)	..	..	15·5% (13·4 to 17·5)	14 285 633 (12 265 561 to 16 293 730)	..	..
2050	20·3% (18·5 to 22·3)	15 511 318 (13 234 280 to 18 140 882)	..	11·4% (4·0 to 17·9)	26·5% (20·0 to 30·8)	20 044 342 (14 396 995 to 24 851 629)	..	126·2% (77·6 to 152·6)
**North Africa and Middle East**								
1990	10·2% (9·5 to 11·1)	9 086 281 (8 413 098 to 9 832 428)	..	..	3·0% (2·7 to 3·3)	2 649 424 (2 392 808 to 2 935 100)	..	..
2021	17·6% (16·4 to 18·7)	21 448 748 (19 969 532 to 22 880 779)	72·2% (55·3 to 89·6)	..	16·0% (14·9 to 17·2)	19 577 938 (18 223 444 to 21 013 188)	443·5% (380·6 to 509·7)	..
2030	18·1% (16·7 to 19·4)	21 999 432 (20 171 810 to 23 858 235)	..	..	21·7% (19·2 to 23·8)	26 023 494 (22 753 130 to 28 889 586)	..	..
2050	16·9% (15·0 to 18·8)	20 378 133 (16 837 721 to 24 255 132)	..	−3·8% (−12·7 to 5·2)	36·4% (28·7 to 41·7)	43 413 693 (32 595 960 to 53 092 189)	..	126·9% (80·9 to 155·7)
**South Asia**								
1990	3·3% (2·7 to 4·1)	9 119 534 (7 393 112 to 11 229 221)	..	..	0·9% (0·7 to 1·1)	2 418 918 (1 835 472 to 3 125 246)	..	..
2021	6·3% (5·2 to 7·6)	22 301 783 (18 165 032 to 26 860 632)	92·6% (42·5 to 156·0)	..	3·2% (2·5 to 3·9)	10 993 705 (8 752 745 to 13 360 876)	276·9% (164·0 to 420·0)	..
2030	7·3% (6·0 to 8·7)	22 694 597 (18 014 475 to 27 223 499)	..	..	4·5% (3·6 to 5·4)	13 728 907 (10 715 112 to 17 004 067)	..	..
2050	9·3% (7·7 to 11·1)	21 750 968 (16 264 419 to 27 940 590)	..	48·5 (36·1 to 58·3)	8·3% (6·0 to 10·5)	18 807 225 (12 336 387 to 25 346 436)	..	156·4% (100·4 to 190·4)
**Southeast Asia, east Asia, and Oceania**								
1990	4·6% (4·4 to 4·8)	15 029 811 (14 313 891 to 15 717 392)	..	..	1·2% (1·1 to 1·3)	3 843 897 (3 604 901 to 4 089 638)	..	..
2021	9·7% (9·2 to 10·1)	29 640 569 (28 295 024 to 31 005 348)	111·4% (98·7 to 124·8)	..	5·9% (5·6 to 6·2)	18 137 955 (17 226 690 to 19 127 296)	403·9% (365·1 to 445·8)	..
2030	10·8% (10·1 to 11·4)	28 333 414 (25 952 142 to 30 569 694)	..	..	8·0% (6·8 to 8·8)	20 020 678 (16 860 945 to 22 502 896)	..	..
2050	13·1% (11·6 to 14·3)	25 626 555 (21 842 103 to 29 187 678)	..	35·2% (22·0 to 46·8)	14·9% (9·3 to 17·9)	28 621 580 (17 144 746 to 36 411 483)	..	154·6% (61·2 to 201·6)
**Sub-Saharan Africa**								
1990	6·0% (5·6 to 6·4)	8 042 700 (7 565 817 to 8 571 411)	..	..	1·5% (1·4 to 1·6)	2 043 582 (1 884 720 to 2 217 821)	..	..
2021	10·0% (9·4 to 10·7)	30 230 795 (28 316 592 to 32 325 748)	67·1% (53·1 to 82·4)	..	4·7% (4·4 to 5·1)	14 381 772 (13 411 550 to 15 405 705)	215·8% (185·0 to 248·8)	..
2030	11·1% (10·4 to 11·9)	38 431 321 (35 239 954 to 42 294 721)	..	..	6·3% (5·8 to 6·8)	21 832 118 (19 787 132 to 24 089 710)	..	..
2050	13·3% (12·4 to 14·4)	57 536 497 (48 607 632 to 67 262 601)	..	33·3% (28·1 to 37·8)	11·4% (9·9 to 12·5)	48 836 876 (39 922 662 to 57 534 015)	..	140·3% (115·7 to 155·2)

Values in parentheses are 95% uncertainty intervals. Estimates specific to overweight and obesity, by sex, age group, and region are provided in appendix 1 (pp 45–49, 54-57, 62–65, 70–71).

**Table 2:** Age-standardised prevalence, percentage change, and number of adolescents aged 15–24 years with overweight and obesity in 1990, 2021, 2030, 2050, globally and within super-regions

	**Overweight (not obesity) among individuals aged 15–24 years**				Obesity among individuals aged 15–24 years			
	Prevalence, %	Estimated mean number	Observed relative change in prevalence, 1990–2021, %	Forecasted relative change in prevalence, 2021–50, %	Prevalence, %	Estimated mean number	Observed relative change in prevalence, 1990–2021, %	Forecasted relative change in prevalence, 2021–50, %
**Global**								
1990	8·0% (7·8 to 8·2)	80 796 236 (78 622 682 to 83 125 166)	..	..	1·9% (1·8 to 1·9)	18 888 788 (18 315 960 to 19 538 335)	..	..
2021	13·7% (13·2 to 14·3)	168 015 855 (161 929 465 to 174 237 911)	72·1% (64·1 to 80·1)	..	6·6% (6·4 to 6·8)	80 623 835 (78 209 565 to 83 259 764)	253·3% (236·5 to 269·1)	..
2030	15·2% (14·6 to 15·8)	203 503 516 (194 761 251 to 213 139 082)	..	..	8·7% (7·9 to 9·2)	115 922 532 (106 109 049 to 123 183 105)	..	..
2050	17·5% (16·7 to 18·3)	215 497 572 (194 432 157 to 237 425 261)	..	28·0% (22·6 to 31·4)	14·2% (11·4 to 15·7)	174 535 945 (136 380 887 to 202 753 539)	..	114·4% (74·2 to 135·5)
**Central Europe, eastern Europe, and central Asia**								
1990	10·4% (9·7 to 11·2)	6 393 737 (5 991 318 to 6 875 256)	..	..	2·5% (2·2 to 2·7)	1 520 127 (1 384 756 to 1 662 134)	..	..
2021	16·0% (15·0 to 17·1)	7 414 493 (6 954 748 to 7 896 302)	54·7% (39·7 to 70·0)	..	6·1% (5·6 to 6·8)	2 845 371 (2 605 150 to 3 147 318)	149·4% (118·7 to 184·9)	..
2030	17·0% (15·8 to 18·3)	9 210 192 (8 396 842 to 10 020 044)	..	..	7·3% (6·4 to 8·2)	3 924 467 (3 389 850 to 4 443 520)	..	..
2050	19·3% (17·2 to 21·1)	8 263 011 (7 053 410 to 9 398 791)	..	20·6% (8·7 to 27·2)	10·0% (8·0 to 11·7)	4 301 948 (3 349 052 to 5 152 388)	..	63·0% (35·0 to 82·1)
**High income**								
1990	14·2% (13·7 to 14·7)	20 083 686 (19 397 232 to 20 770 417)		..	5·0% (4·7 to 5·2)	7 049 954 (6 709 222 to 7 401 271)	..	..
2021	20·0% (19·1 to 20·9)	25 792 669 (24 687 914 to 26 950 054)	40·8% (33·2 to 48·9)	..	14·9% (14·1 to 15·7)	19 274 887 (18 262 054 to 20 348 660)	199·3% (179·2 to 221·0)	..
2030	20·8% (19·9 to 21·8)	27 238 880 (25 844 243 to 28 709 740)	..	..	17·2% (15·9 to 18·3)	22 615 280 (20 892 733 to 24 247 776)	..	..
2050	21·9% (20·7 to 23·1)	25 383 514 (23 246 536 to 27 992 060)	..	9·6% (5·2 to 12·5)	23·1% (19·9 to 25·4)	26 985 781 (22 500 298 to 30 647 437)	..	55·4% (36·7 to 67·4)
**Latin America and Caribbean**								
1990	14·5% (13·4 to 15·7)	11 326 254 (10 499 720 to 12 288 247)	..	..	3·7% (3·3 to 4·1)	2 870 352 (2 564 888 to 3 176 054)	..	..
2021	22·4% (21·0 to 23·8)	21 675 403 (20 333 720 to 23 018 983)	54·8% (39·0 to 70·2)	..	13·5% (12·4 to 14·6)	13 074 142 (12 057 092 to 14 232 795)	266·7% (221·5–322·4)	..
2030	23·6% (22·0 to 25·2)	22 314 662 (20 538 915 to 24 146 027)	..	..	17·8% (15·9 to 19·7)	16 844 434 (14 772 145 to 18 921 536)	..	..
2050	23·8% (21·8 to 25·9)	19 971 577 (17 548 665 to 22 737 816)	..	6·4% (−0·4 to 11·7)	29·0% (22·5 to 33·1)	24 391 386 (18 281 469 to 29 614 627)	..	115·3% (70·4–139·6)
**North Africa and Middle East**								
1990	12·9% (12·1 to 13·8)	8 552 833 (8 000 973 to 9 128 104)	..	..	3·3% (3·0 to 3·6)	2 164 546 (1 959 774 to 2 392 515)	..	..
2021	21·9% (20·8 to 22·8)	22 572 310 (21 511 948 to 23 533 301)	69·4% (55·5 to 83·5)	..	15·8% (14·9 to 16·6)	16 269 385 (15 430 773 to 17 110 550)	380·2% (328·2 to 441·6)	..
2030	22·6% (21·5 to 23·7)	27 334 037 (25 499 549 to 29 179 952)	..	..	21·5% (19·3 to 23·2)	25 995 165 (23 293 218 to 28 523 549)	..	..
2050	21·5% (19·4 to 23·3)	25 589 351 (21 710 935 to 29 638 458)	..	−1·9% (−10·2 to 5·6)	35·7% (27·7 to 40·5)	42 630 500 (31 937 376 to 50 644 300)	..	126·0% (78·8 to 155·4)
**South Asia**								
1990	4·4% (3·7 to 5·3)	9 104 094 (7 563 435 to 10 870 550)	..	..	0·5% (0·4 to 0·6)	1 020 030 (817 433 to 1 265 853)	..	..
2021	9·6% (8·2 to 11·4)	33 635 101 (28 631 156 to 39 774 122)	120·9% (70·1 to 177·3)	..	2·3% (1·9 to 2·9)	8 152 635 (6 650 355 to 9 982 873)	379·3% (247·2 to 526·8)	..
2030	11·2% (9·5 to 13·2)	38 694 834 (32 256 880 to 46 076 135)	..	..	3·4% (2·8 to 4·1)	11 855 970 (9 509 024 to 14 469 484)	..	..
2050	14·8% (12·4 to 17·3)	40 301 650 (31 283 034 to 50 465 698)	..	55·5% (41·7 to 65·4)	6·7% (4·7 to 8·4)	18 147 759 (12 495 128 to 23 925 273)	..	175·9% (113·1 to 213·5)
**Southeast Asia, east Asia, and Oceania**								
1990	5·2% (5·0 to 5·5)	19 072 477 (18 211 913 to 20 041 949)	..	..	0·8% (0·8 to 0·9)	3 026 996 (2 840 492 to 3 224 740)	..	..
2021	11·6% (11·1 to 12·1)	31 430 388 (30 161 562 to 32 691 911)	121·4% (108·6 to 134·2)	..	4·7% (4·5 to 5·0)	12 828 373 (12 202 122 to 13 532 828)	469·3% (421·9 to 518·9)	..
2030	13·8% (12·8 to 14·5)	41 381 775 (37 936 417 to 44 629 617)	..	..	6·9% (5·8 to 7·6)	20 839 616 (17 249 508–23 311 346)	..	..
2050	16·4% (14·8 to 17·6)	33 041 788 (28 464 375 to 37 333 495)	..	41·5% (28·4 to 48·8)	12·7% (7·8 to 15·3)	25 622 610 (15 298 911–32 561 956)	..	169·0% (67·2 to 220·2)
**Sub-Saharan Africa**								
1990	6·7% (6·3 to 7·2)	6 263 154 (5 885 966 to 6 718 049)	..	..	1·3% (1·2 to 1·5)	1 236 783 (1 124 067 to 1 354 849)	..	..
2021	11·3% (10·6 to 12·0)	25 495 491 (24 024 159 to 27 065 979)	67·6% (52·8 to 82·7)	..	3·6% (3·4 to 3·9)	8 179 043 (7 590 213 to 8 883 136)	172·3% (139·9 to 207·6)	..
2030	12·8% (12·0 to 13·6)	37 329 135 (34 562 012 to 40 278 288)	..	..	4·8% (4·4 to 5·2)	13 847 602 (12 655 241 to 15 271 729)	..	..
2050	16·1% (14·9 to 17·1)	62 946 680 (55 666 755 to 70 117 792)	..	42·7% (36·9 to 47·8)	8·3% (7·1 to 9·2)	32 455 960 (26 569 446 to 38 062 454)	..	128·5% (103·2 to 144·9)

Values in parentheses are 95% uncertainty intervals. Estimates specific to overweight and obesity, by sex, age group, and region are provided in appendix 1 (pp 45, 50–53, 58–61, 66–69, 72–73).

### Observed transitions between 1990 and 2021

**Figure 1:**
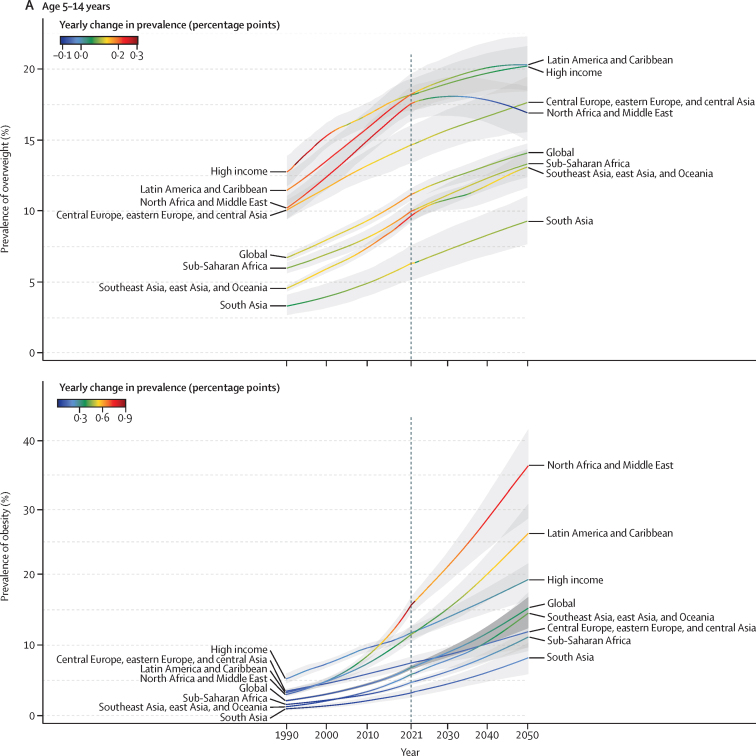

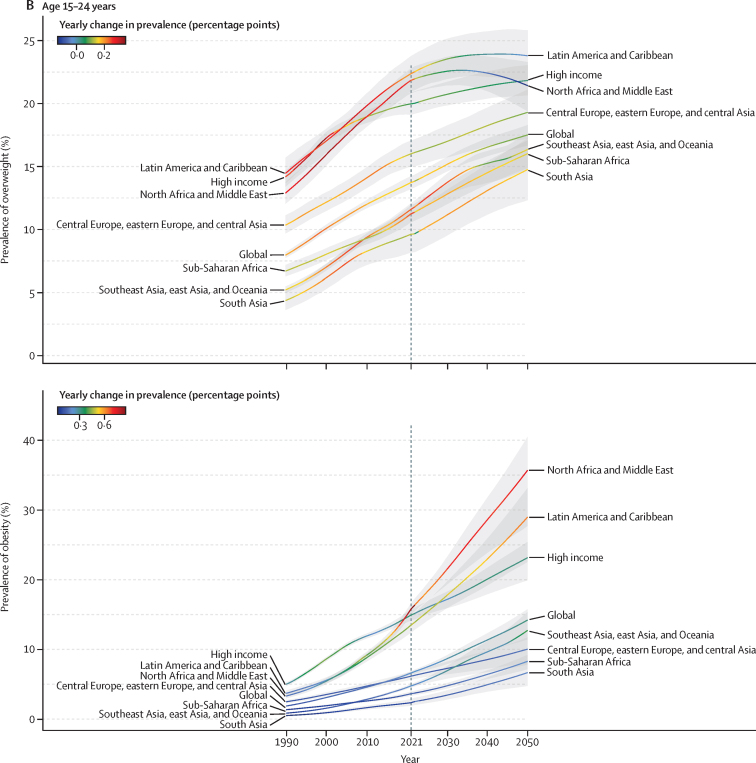
Trajectories of the estimated prevalence of overweight and obesity among children and adolescents, 1990–2050, by super-region (A) Overweight (top) and obesity (bottom) among children and young adolescents aged 5–14 years. (B) Overweight (top) and obesity (bottom) among older adolescents aged 15–24 years. Note: y-axes differ between graphs.

**Figure 2:**
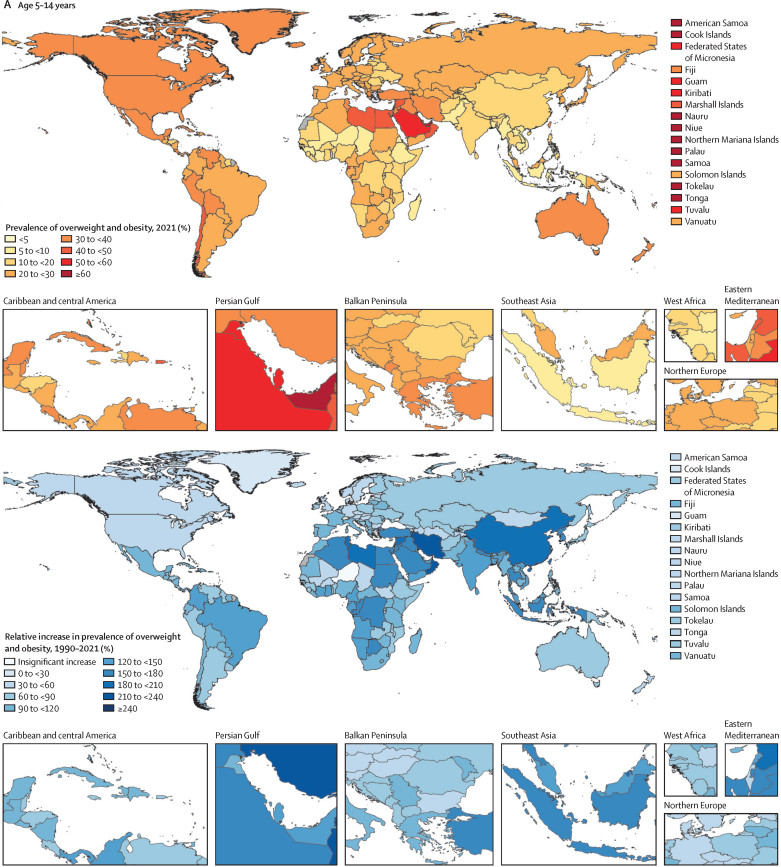

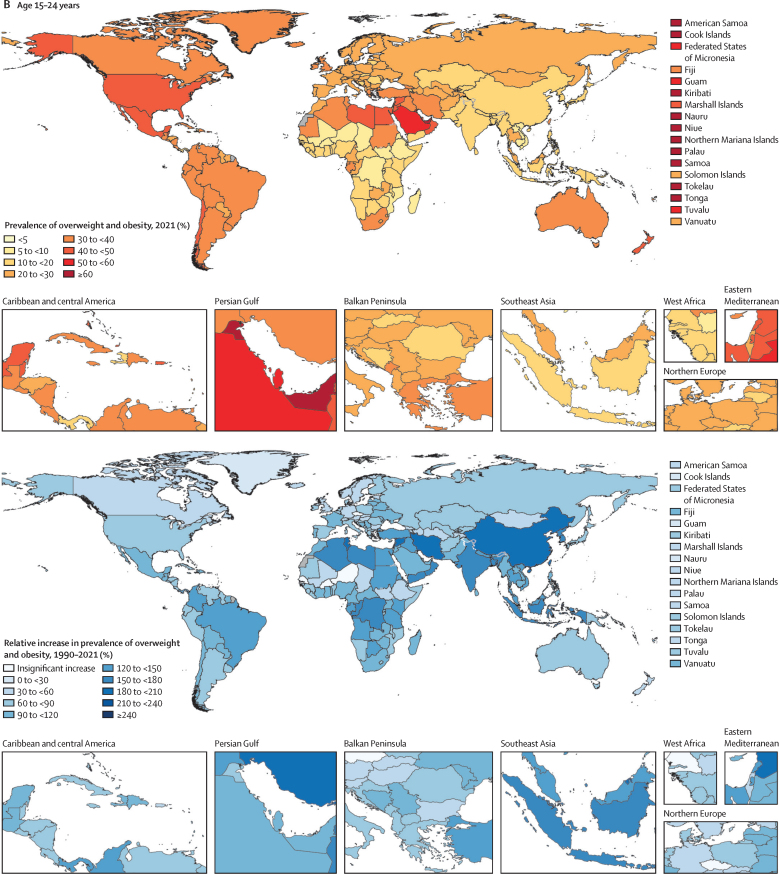
Prevalence of overweight and obesity among children and adolescents, 2021, and percentage change, 1990–2021 (A) Children and young adolescents aged 5–14 years. (B) Older adolescents aged 15–24 years. No estimates are available for Western Sahara, French Guiana, or Svalbard, as these were not modelled locations in the Global Burden of Diseases, Injuries, and Risk Factors Study 2021.

**Figure 3:**
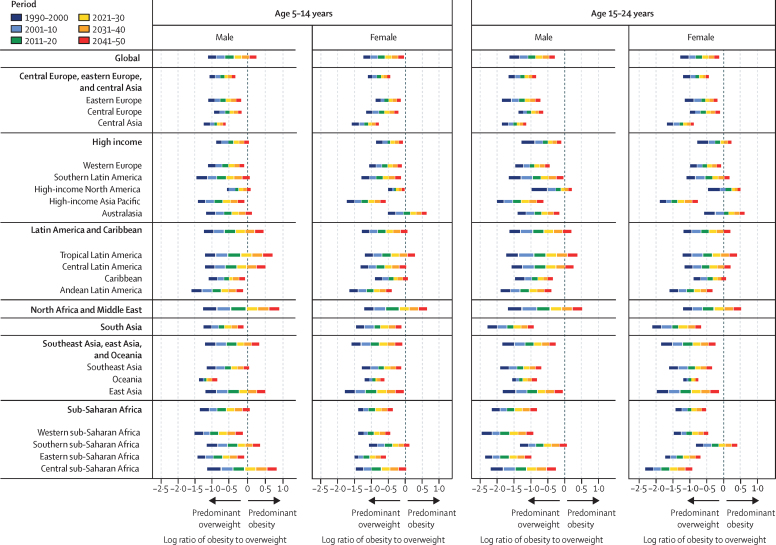
Ratio of obesity to overweight, 1990–2050, by time period, age, sex, super-region, and region

There was substantial geographical variation in weight gain by super-region and age. For all children and adolescents (aged 5–14 years and 15–24 years), increases in overweight and obesity between 1990 and 2021 were fastest in north Africa and the Middle East, Latin America and the Caribbean, and in the high-income super-region from 1990 to 2005 (figure 1). In Latin America and the Caribbean, gains were mainly driven by tropical Latin America (eg, Brazil), and in the high-income super-region by the Asia Pacific region (eg, Singapore), with other high-income regions (eg, North America and Australasia) having already made the majority of their gains before 1990 (figure 2). Around 2021, obesity prevalence remained on a rapid growth trajectory, whereas growth in overweight prevalence was beginning to slow in Latin America and the Caribbean and north Africa and the Middle East. Observed accelerations in obesity prevalence were particularly rapid in north Africa and the Middle East between 2010 and 2021. Due to these rapid increases, the average 2021 prevalence was greatest in north Africa and the Middle East, particularly among adolescent females (appendix 1 pp 66–69). Globally, females aged 15–24 years in this super-region were the only super-region subgroup to exceed 40% prevalence of overweight and obesity combined in 2021 (obesity 18·3% [17·1–19·7], overweight 23·9% [22·4–25·3]). However, at a regional level, unlike any other population subgroups across the world, females in Australasia (aged 5–14 years and 15–24 years) and in high-income North America (aged 15–24 years) transitioned to obesity (*vs* overweight) predominance before 2021 (figure 3; appendix 1 pp 74–235). This transition for female adolescents occurred as early as 2010 in Australia and the USA (appendix 1 pp 236–243). Although no other region made this transition as quickly as female children and adolescents in high-income North America and Australasia, by 2021, female populations in several countries (eg, United Arab Emirates, Qatar, Virgin Islands, and Puerto Rico) in these three super-regions (high income, Latin America and the Caribbean, and north Africa and the Middle East) were already or were close to becoming obesity-predominant (appendix 1 pp 236–243).

Conversely, in all other super-regions (southeast Asia, east Asia, and Oceania; central Europe, eastern Europe, and central Asia; sub-Saharan Africa; and south Asia), the annual percentage changes for increases in both overweight and obesity were steadier between 1990 and 2021, resulting in lower prevalence in 2021 (figure 1). However, beyond 2015, we observed heterogeneity within the super-region of southeast Asia, east Asia, and Oceania. Specifically, because multiple countries in Oceania (eg, Cook Islands and American Samoa) transitioned to obesity predominance before or between 1990 and 2010 (appendix 1 pp 236–243), overall change between 1990 and 2021 was less in Oceania than in east Asia and southeast Asia (appendix 1 pp 62–69). Change in obesity was very pronounced in east Asia between 1990 and 2021 in both those aged 5–14 years (488·5% [95% UI 440·4–539·7]) and aged 15–24 years (597·2% [537·2–657·8]). As a result, obesity prevalence in this super-region in 2021 was highest for young males in east Asia and for females in Oceania (appendix 1 pp 62–69). Although the average obesity prevalence remained below overweight prevalence in all three of these regions by 2021 (appendix 1 pp 74–235), most countries in Oceania (but not in east or southeast Asia) had transitioned to obesity predominance by 2021 (appendix 1 pp 236–243).

### Forecasted transitions between 2022 and 2050

If current trends continue, we forecast that around a third of the world's children and adolescents will have overweight or obesity by 2050 (Table 1, Table 2; appendix 1 pp 45–53), equating to 356 million (95% UI 295–411) young people aged 5–14 years and 390 million (331–440) aged 15–24 years (total 746 million [627–851]). Of those, 360 million (278–422) children and adolescents would have obesity by 2050 (Table 1, Table 2), the result of a 120·7% (80·7–141·6) increase in prevalence. Globally, 9·1% (8·4–9·7) of those aged 5–14 years are forecasted to have obesity by 2030, increasing to 15·6% (12·7–17·2), or 186 million individuals [141–221]), by 2050. Despite being estimated to have rapid increases in obesity during the forecasted period (appendix 1 p 279), obesity prevalence in adolescents aged 15–24 years is forecasted to remain lower than that in younger children and adolescents, at 8·7% (7·9–9·2) in 2030 and 14·2% (11·4–15·7), or 175 million individuals (136–203), by 2050. Largely, this difference between age groups is driven by higher forecast changes in obesity rates among those aged 5–14 years versus those aged 15–24 years in Africa (north Africa and the Middle East and sub-Saharan Africa), central and eastern Europe, and Asia. On average, obesity prevalence in 2050 is forecasted to be greater among those aged 15–24 years than among the younger age groups only in the high-income super-region and in Latin America and the Caribbean (Table 1, Table 2).

At the super-region level, tables 1 and 2 show that the largest forecasted increase in obesity occurs across south Asia (eg, Nepal) and southeast Asia, east Asia, and Oceania (eg, North Korea). The populations that are forecasted to have the most rapid accelerations in obesity during the forecasted period (eg, Latin America and the Caribbean [including Mexico and Barbados] and north Africa and the Middle East [including Egypt and Iran]) are also forecasted to have plateaus or declines in overweight prevalence as greater proportions of their populations shift to obesity status (figure 1; appendix 1 p 245). The prevalence in 2050 is forecasted to be greatest in Latin America and the Caribbean and in north Africa and the Middle East, where around half of all children and adolescents will have overweight or obesity and around a third will have obesity (Table 1, Table 2; appendix 1 p 45). By 2050, in just the Latin America and the Caribbean (44·4 million [95% UI 32·9–53·7]) and north Africa and the Middle East super-regions (86·0 million [65·4–103]), 130 million children and adolescents are forecasted to have obesity, equating to around a third of the world's children and adolescents with obesity (Table 1, Table 2). Comparatively, the southeast Asia, east Asia, and Oceania super-region will be partway through its transition to obesity predominance (figure 3). Central Europe, eastern Europe, and central Asia; south Asia; and sub-Saharan Africa will be earlier in their transition to obesity predominance (figure 3). Despite these super-regions being earlier in their transition, because of a large population size, a quarter of the world's population of children and adolescents with obesity are forecasted to live in sub-Saharan Africa by 2050 (81·3 million [66·7–95·5]).

### Super-regions forecasted to be obesity-predominant

By 2030–50, Latin America and the Caribbean, north Africa and the Middle East, and the high-income super-region will be late in their obesity transition, signalled by many population subgroups having transitioned to obesity predominance during this period (figure 3). Between 2020 and 2040, obesity is forecasted to become predominant for most young people aged 5–24 years in tropical Latin America (eg, Brazil) and north Africa and the Middle East. In addition to other countries and population subgroups (figure 3; appendix 1 pp 236–243), by 2030–40 obesity is also forecasted to become predominant for young males aged 5–14 years in central Latin America (eg, Mexico, Venezuela, and Costa Rica), and for females in Australasia (age 5–24 years; eg Australia) and for adolescent males and females in high-income North America (age 15–24 years; eg, the USA). At the country level, obesity estimates in 2050 within such regions are expected to be highest in north African and Middle Eastern countries, particularly in the United Arab Emirates, Kuwait, Saudi Arabia, and Bahrain (appendix 1 pp 246–251, 264–275). Obesity estimates are also noteworthy in Caribbean countries such as Dominica and the Virgin Islands. Despite these patterns, there are some countries and regions across these three super-regions where this obesity transition is not forecasted to occur—eg, high-income Asia Pacific, Andean Latin America, and some north African and Middle Eastern countries such as Türkiye and Afghanistan (appendix 1 pp 236–243). Figure 3 also shows key differences by age and sex, with more populations of females aged 5–14 years and males aged 15–24 years forecasted to remain overweight-predominant in these obesity-predominant super-regions.

### Super-regions forecasted to be overweight-predominant

Compared with obesity prevalence across the three super-regions outlined above (north Africa and the Middle East, Latin America and the Caribbean, and the high-income super-region), there are several examples of lower forecasted obesity prevalence (appendix 1 pp 246–251, 264–275) and of forecasted overweight predominance in the super-regions that began transitioning to overweight and obesity later in time. Despite some substantial increases in obesity among certain subpopulations (eg, males in South Sudan and Eritrea [eastern sub-Saharan Africa]; appendix 1 pp 62–69), numerous settings are expected to remain overweight-predominant by 2041–50 (figure 3), including the majority of subpopulations in central and eastern Europe, central Asia, south Asia, southeast Asia, and western and eastern sub-Saharan Africa. Figure 3 also shows age and sex differences in east Asia and southern sub-Saharan Africa. Distinct from their surrounding super-region, outlier populations in the following locations are expected to become obesity-predominant by 2041–50: males aged 5–14 years in east Asia and central sub-Saharan Africa, and males aged 5–14 years and females aged 15–24 years in southern sub-Saharan Africa; and males and females aged 5–24 years across several Oceanic countries (appendix 1 238–242). In fact, because most Oceanic countries transitioned to obesity before 2021, by 2050, the Cook Islands, Tonga, Northern Mariana Islands, and Nauru are forecasted to have some of the highest obesity estimates (eg, 60–70%) in the world (appendix 1 pp 246–251). These countries with very high forecasted obesity prevalence are expected to decrease in overweight prevalence during the forecasted period as most of the population shifts from overweight to obesity status. Despite these patterns, it is noteworthy that the average overweight prevalence in Oceania is yet to stabilise at the regional level (appendix 1 pp 54–61, 74–235). This is because the regional trends for Oceania are largely driven by the more populous Papua New Guinea, where child and adolescent obesity prevalence is forecasted to remain very low but where overweight will still increase.

### Overall patterns

**Figure 4:**
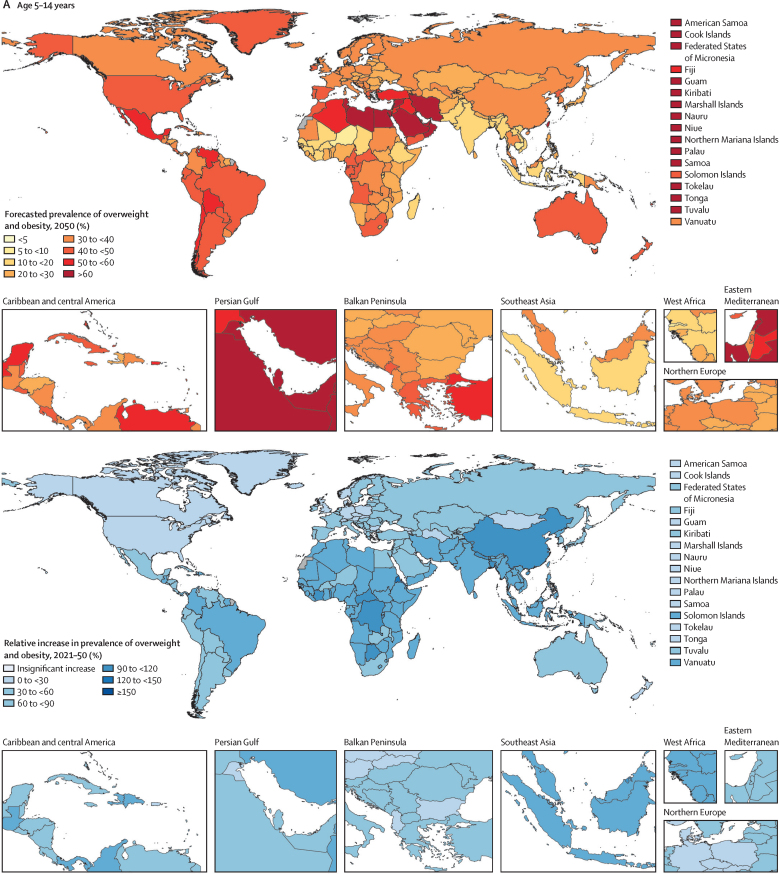

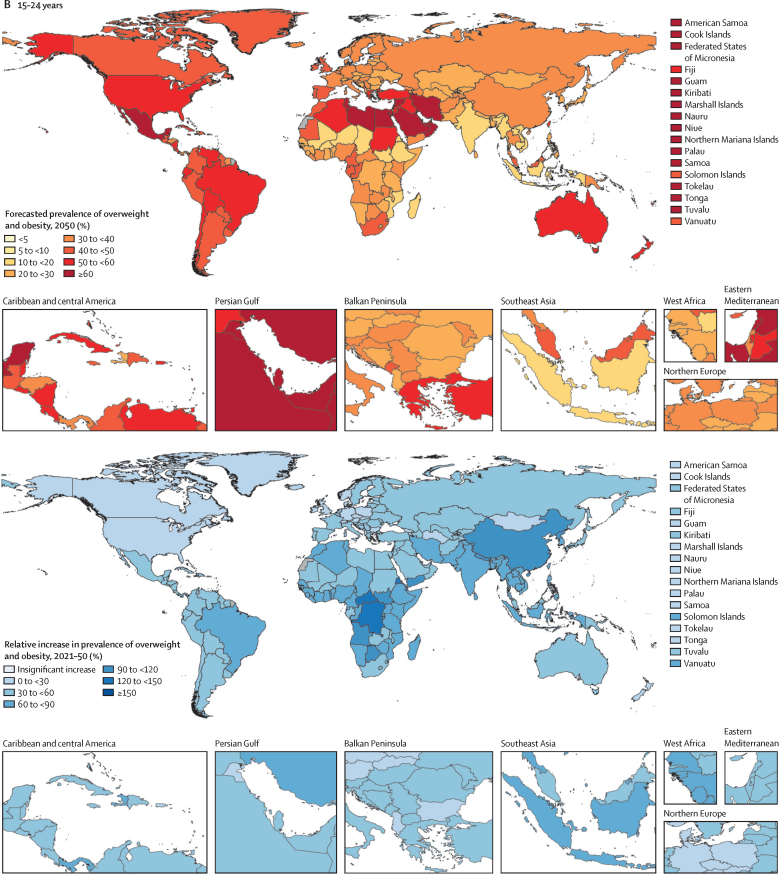
Forecasted prevalence of overweight and obesity among children and adolescents, 2050, and percentage change, 2021–50 (A) Children and young adolescents aged 5–14 years. (B) Older adolescents aged 15–24 years. No estimates are available for Western Sahara, French Guiana, or Svalbard, as these were not modelled locations in the Global Burden of Diseases, Injuries, and Risk Factors Study 2021.

**Figure 5:**
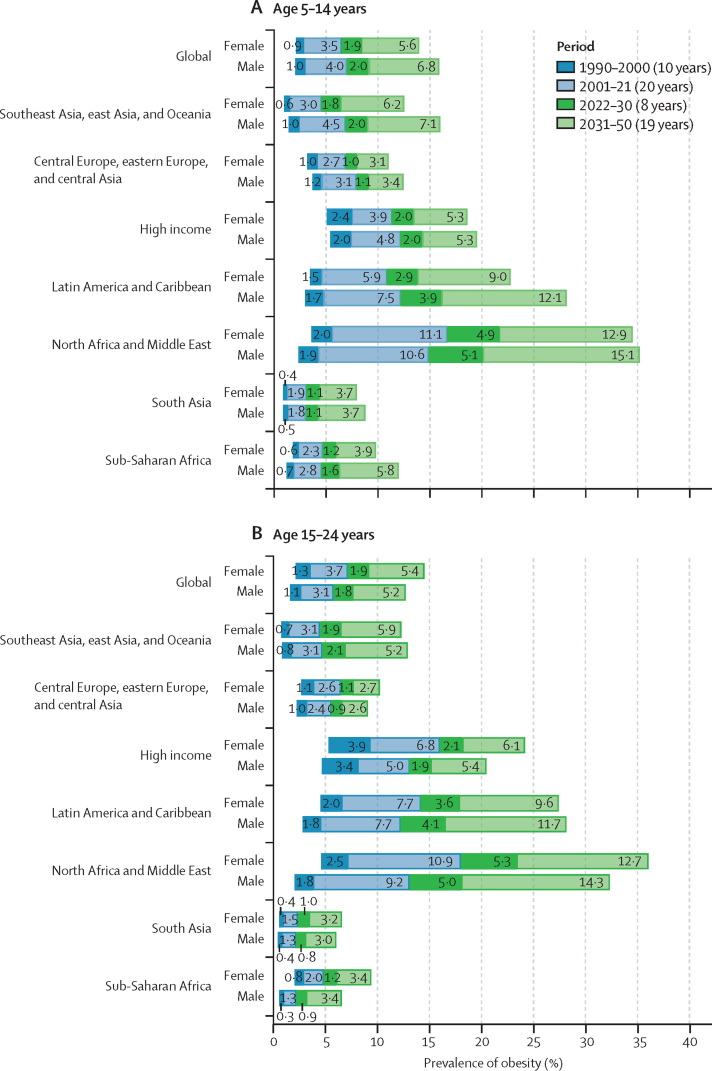
Change in the percentage of children and adolescents with obesity, 1990–2050, by super-region and by sex (A) Children and young adolescents aged 5–14 years. (B) Older adolescents aged 15–24 years. Values shown on each bar represent the percentage increase in obesity prevalence for a given time period.

These forecasted patterns signal sex-related aspects of weight (appendix 1 p 244) in ways that are distinct for individuals aged 5–14 years versus those aged 15–24 years. Globally, males aged 5–14 years are the only population subgroup forecast to become obesity-predominant before 2050, in around 2040 (appendix 1 pp 74–154). Specifically, by 2050 we forecasted that 16·5% (13·3–18·3) of young males will be living with obesity, compared with 12·9% (12·2–13·6) living with overweight. Figure 4 shows the overall changes anticipated in all regions for boys and girls in both the younger and older age groups, regardless of age-related and sex-related effects. If trends continue, at a global level, there will only be a handful of countries with remaining low prevalence of overweight and obesity by 2050: in eastern and western sub-Saharan Africa (eg, Mozambique, Ethiopia, and Niger) and south and southeast Asia (eg, India and Indonesia). Despite the relative percentage change in global overweight and obesity being lower during the future period (2021–50; figure 4) than during the past period (1990–2021; figure 2), the absolute gain in the proportion of the population with obesity alone is forecasted to be greater in the future (figure 5; appendix 1 pp 276–277). Across all regions, sexes, and both child and adolescent age groups, substantial increases are forecast in the absolute gain in obesity prevalence during the 8 years between 2022 and 2030, which continues beyond 2031 to 2050 (figure 5).

## Discussion

### Principal findings

The global prevalence of obesity in children and adolescents increased by 244·0% (95% UI 229·5–258·8) between 1990 and 2021. Obesity is forecasted to increase by a further 120·7% (80·7–141·6) between 2021 and 2050, with this future period marked by greater absolute increases in the proportion of children and adolescents who have obesity. Without immediate action, around a third (746 million) of the world's children and adolescents are expected to be living with overweight or obesity by 2050, of whom approximately half (360 million) will have obesity. Globally and throughout many regions (eg, Africa and Asia), the younger population (aged 5–14 years) is expected to fare the worst, especially young males. Without urgent policy reform and action, the transition to obesity will be particularly rapid in north Africa and the Middle East and in Latin America and the Caribbean, where the rise in prevalence is concurrent with high population numbers. By 2050, a third of the world's children and adolescents with obesity are expected to live in these two super-regions. The ongoing transition to obesity will be particularly devastating for several Oceanic countries, where obesity prevalence is expected to reach 70% by 2050.

The prevalence of overweight is forecasted to plateau in some regions, but this is primarily due to more of the population transitioning to obesity. In contrast to recent commentary,^26^, ^72^ there is no indication of any plateau in the increase of obesity prevalence, which is not expected to stabilise in any region before 2050. If these global forecasts are realised, the impacts will not only be overwhelming for individuals, but the resulting burden will be devastating across health, social, planetary, and economic systems.^12^, ^73^ Obesity in these young populations will impact future societal and economic developments if the forthcoming workforce carries this large disease burden. At the individual level, child and adolescent obesity will immediately affect young lives.^4^, ^5^, ^6^, ^7^, ^8^ There will be even greater effects on their future lives^1^, ^5^, ^19^, ^20^ and on their offspring,^28^, ^30^ as children and adolescents with obesity are highly likely to become adults with obesity.^13^, ^14^, ^15^, ^16^ The lack of reprieve in some high-income regions, in Latin America, and across north Africa, the Middle East, and Oceania will establish obesity across generations. Currently, many populations in these regions (eg, adolescents in Australasia and North America) already require clinical management of obesity.^74^, ^75^, ^76^, ^77^ However, despite obesity predominance in these selected regions, it is not too late to stop most of the world's children and adolescents transitioning from overweight to obesity predominance.

Interruption of this transition is urgently needed to avoid the current and anticipated burden of obesity-specific complications. The next 5 years (2025–30) are particularly crucial for decision makers to address this epidemic. If action is not taken before 2030, a prevalence spike in low-income and middle-income countries (LMICs) will precipitate public health emergencies in the face of high population numbers and limited resources (eg, in Africa).^78^, ^79^ Many regions have historically had to focus on preventing underweight and stunting in children. These forecasts, especially for obesity, indicate the importance of policy makers becoming better prepared to respond to this newer health threat. An immediate imperative is national surveillance of obesity in children and adolescents, as this will enable prioritisation of government investments and health system responses, particularly those that address systemic drivers of overweight and obesity.

### Drivers and intervention targets

Obesity risk varies by age and by sex, suggesting biological drivers. Yet rapid change and geographical variations indicate potentially modifiable determinants.^25^, ^80^ As articulated in the 2019 *Lancet* Commission on obesity, undernutrition, and climate change,^73^ the obesity epidemic has been driven by several interacting factors within high-level societal and environmental systems, which act together with local and individual-level factors.^80^, ^81^ Universal systemic drivers include obesogenic shifts in transport, media, and food systems (eg, commercialisation), and in industry and trade laws that have accompanied globalisation and the epidemiological transition, such as urbanisation and economic development.^82^, ^83^, ^84^ Many of these changes have also triggered wealth and educational inequalities, food insecurity, environmental pollution, nutrition transitions to western-style diets, and disruptions to local agricultural practices and food supply systems,^25^, ^80^, ^84^, ^85^, ^86^, ^87^ many of which interact with climate change, which will continue to exacerbate the current and forecasted obesity crisis.^73^, ^84^ Within this context, obesity has accelerated in children and adolescents, with little clarity around successful and sustainable strategies to address these complex factors that lie at the heart of the epidemic. While families and individuals can work to balance their physical activity, dietary intake, and sleep to uphold a healthy lifestyle, this lifestyle is difficult to maintain while living in obesogenic environments. Indeed, it is increasingly understood that in general, without being coupled with collective policy actions, isolated lifestyle-based individually oriented behaviour-change strategies do not produce meaningful or sustainable change unless they are very intensive with high contact hours—a barrier for most families and health systems, even in high-income countries.^88^, ^89^, ^90^, ^91^ Instead, it is governments rather than individuals that are required to address population-level drivers of obesity, such as its commercial determinants (eg, marketing, pricing, and food industry lobbying). Successful population-level strategies are beginning to emerge,^92^ with multifaceted actions targeting strategies across both public and private sectors (including food industry actors) that are adapted to local contexts.^73^, ^80^, ^92^, ^93^ Programmes and policies cannot be copied across multiple settings; one's local context is unique and strategies need to be tailored accordingly by country, state, community, and neighbourhood.^80^ Such strategies can be informed by our country-specific data on the stage, timing, and speed of current and forecasted transitions in weight.

### Strategies for subgroups with overweight predominance

Population-level normal weight, rather than overweight, should be the future goal. Yet, before we can focus on that goal, we must prevent populations from transitioning from overweight to obesity. If we view overweight as a reversable risk exposure, and obesity as a complex chronic disease that is difficult to reverse,^3^ then government actions can be most efficient if directed by the status of overweight and obesity predominance in particular countries. Prevention is crucial considering how difficult obesity is to reverse once it is established in childhood or adolescence.^13^, ^14^, ^15^, ^16^ Opportunely, there are several regions and population subgroups that remained overweight-predominant in 2021 and are expected to be overweight-predominant in 2040 or 2050. A number of these settings present ideal opportunities to both address overweight and curb future increases in obesity. This includes many regions of Asia, Europe, and sub-Saharan Africa, select regions of Latin America and the Caribbean (eg, the Caribbean and Andean Latin America), and the high-income super-region (eg, western Europe and Asia Pacific), and isolated countries in Oceania (eg, Papua New Guinea) and in north Africa and the Middle East (eg, Türkiye and Afghanistan). Similar interventions should also urgently be directed towards children and adolescents in east Asia and central and southern sub-Saharan Africa who, despite having moderate obesity prevalence in 2021, are at a tipping point and about to enter a rapid transition to obesity. Preventive actions should also be prioritised in heavily populated countries such as Nigeria, India, Pakistan, and China, where we show that the estimated numbers of children and adolescents with obesity are forecast to be devastating.

Most of these preventive prospects exist in LMICs, where there are still opportunities to change population-level drivers (eg, food systems in sub-Saharan Africa)^94^ to make real gains in preventing obesity. In these countries, policies must balance the challenge of overnutrition with ongoing concerns about undernutrition (including micronutrient deficiencies) and stunting.^24^, ^85^, ^95^ Specific to the prevention of overnutrition and obesity, guidance on population-level prevention can be sought from both WHO and UNICEF strategy documents for the prevention of child and adolescent overweight and obesity.^37^, ^96^ Feasible interventions in countries constrained by funding and undernutrition priorities include double-duty maternal and child health programmes that target undernutrition and overnutrition via health education, breastfeeding, and prenatal care, and also regulatory and fiscal interventions that have low implementation cost (eg, subsidising healthy foods and taxing unhealthy foods such as sugar-sweetened beverages) which have shown some success in LMICs.^94^, ^96^, ^97^, ^98^, ^99^ Governments and donors need to coordinate these strategies while also targeting systemic drivers of underweight and overweight at the population level, such as socioeconomic factors and food systems.^73^, ^93^, ^95^ Adolescents have increasing independence from families over their food choices.^92^ For them, rather than family-based or school-based interventions, coordinated actions must work to avoid so-called big food companies overtaking local food systems by maintaining patterns of local agriculture and traditional food preparations, while also modernising food systems and their infrastructure (eg, skills training, marketing reforms, and industry entrepreneurship).^94^ For children and younger adolescents who are at school, government-funded nutritious school meals can be universally scaled^100^ and are also increasingly recognised as a means to achieve multisectoral policy objectives (eg, education, agriculture, social production, and human and planetary health).^101^ This strategy might more quickly be implemented before the necessary wider infrastructure and fiscal policy changes. Recent success in China has been attributed to a series of comprehensive multifaceted preventive interventions (DECIDE-Children) delivered across socioeconomically distinct regions.^102^ Designed for the Chinese context, with the potential for national scaling, these evidence-based interventions engaged families, teachers, and local stakeholders; targeted school policies (eg, no sugary drinks) and health education; and redesigned school lunches and physical education lessons. Such multifaceted initiatives benefit from coordination across sectors and different levels of government.^92^

### Strategies for subgroups with obesity predominance

By contrast, obesity is currently, or is soon to be, the main burden affecting multiple population subgroups in Oceania, north Africa and the Middle East, tropical and central Latin America, Australasia, and high-income North America. These populations with widespread and established obesity already have expansive infrastructure that promotes passive transport and leisure activities, and their traditional local food supply systems have long been replaced by so-called big food distribution, including high-calorie foods with long shelf lives.^82^, ^103^, ^104^ Governments and health systems in these regions need to urgently invest in clinical management and treatment options. The extreme forecasts in these locations have considerable implications for global disease burden, particularly given that adolescent obesity increases risks for multiple cancers, kidney disease, musculoskeletal disorders, cardiovascular diseases, mental disorders, and premature mortality as early as young adulthood.^1^, ^18^, ^19^, ^20^, ^21^ As noted, unless coupled with collective policy action, individual-level management strategies applicable in high-income and low-income countries (eg, intensive behavioural therapy) are burdensome, unsustainable, and commonly only lead to small improvements.^87^, ^88^, ^89^, ^90^ For those with severe obesity, individual-level treatment options (eg, antiobesity medications, bariatric surgery, and very low-energy diets) are available in some middle-income and high-income countries for postpubertal adolescents.^81^, ^105^ This field is moving quickly, and evidence for the efficacy of these treatments is growing in young people^106^, ^107^, ^108^, ^109^, ^110^ and children.^111^ Although antiobesity medications have the ability to positively impact the forecasted trends in obesity, availability and regulatory approval vary across regions and countries, and current evidence in LMICs is scarce.^81^, ^87^, ^105^, ^112^ Care should be taken to ensure that current inequities in obesity and associated disease burden are not further entrenched by inequitable supply of, and access to, these treatment options between high-income and low-income settings (eg, by promoting pharmacoequity).^112^, ^113^, ^114^

To support evidence-based decision making, recent evidence from Australia shows the strength of combining several co-designed intervention strategies to achieve the biggest impact on child and adolescent obesity (eg, financial support for organised sport and sugar-sweetened beverage taxation).^115^ In addition, the Amsterdam Healthy Weight Approach^116^ is an example of a successful health-in-all-policies multifaceted intervention to reduce child and adolescent obesity*.*^117^ This intervention included changes in the community (eg, urban planning, funding, advertising bans, and price discounts), home (eg, home visits), and school (eg, canteen changes). Other examples of multifaceted system-level interventions delivered in high-income countries include the Stanford GOALS trial^118^ and the WHO STOPs trial,^119^ which also show the potential of system-level approaches. Distinct from high-income settings, double-duty actions are again important to guide multisectoral interventions in obesity-predominant LMICs (eg, in Oceania, north Africa and the Middle East, and Latin America and the Caribbean). Exemplar quadruple-duty interventions (for undernutrition, overnutrition, food security, and planetary health) are emerging in Latin America and the Caribbean, including Brazil's intersectoral government-regulated school feeding programme, which supports rural family enterprises.^120^, ^121^ Examples of system-level policies beyond the school system are important for older adolescents and have also been in place throughout Latin America and the Caribbean, including the Mexican Government's multi-institutional and multifaceted prevention strategy that incorporates taxation of sugar-sweetened beverages and unhealthy foods, which are banned from many schools; and Chile's legislation on food labelling and advertising.^103^, ^122^ While the long-term effects of such regulations on obesity remain unknown, combined with education and other policies, such regulatory efforts show promise towards achieving the holistic goal of healthier eating within these countries.^103^

Within obesity-predominant regions, adolescent obesity during childbearing years is forecasted to have striking impacts in many LMICs that are already heavily burdened by communicable, maternal, and nutritional conditions during adolescence.^32^ To address the obesity epidemic, WHO has emphasised the importance of adolescent health via preconception and antenatal care.^41^ Because it is parental obesity that impacts offspring risk, calls are also growing for preconception interventions to include fathers as well as mothers.^29^, ^31^ Adolescent females and males both need to be targeted with preconception interventions. Unless urgent treatment and interventions are provided to these adolescents, high rates of obesity (60–70% by 2050 in several countries in Oceania and north Africa and the Middle East, 20–38% in Australasia and high-income North America) will not only trigger serious epidemics of disease (eg, cancer) during adolescence,^6^ subfertility, and pregnancy and perinatal complications (eg, miscarriage, preeclampsia),^5^ but exposure to an obese intrauterine environment will predetermine the health of the next generation.^28^, ^30^ To prevent future obesity in young children and break the intergenerational cycle of metabolic disturbances in these countries, prevention in these regions must also centre on individual-level and population-level preconception and perinatal interventions focused on adolescent adiposity, nutritional status, and gestational weight gain and diabetes,^30^, ^123^, ^124^, ^125^, ^126^ but should also extend to other important intervention targets, such as education.^28^ The multicountry, multisectoral Healthy Lives Trajectory Initiative provides a good example of a framework that aims to prevent both increasing rates of obesity and non-communicable diseases (NCDs), and subsequent intergenerational transmission.^127^, ^128^, ^129^, ^130^, ^131^

### Strengths and limitations

This study integrates data from 180 countries and territories, enabling the most comprehensive global forecasting of overweight and obesity prevalence among children and adolescents to date. While we acknowledge that normal weight needs to be the goal, given the current state of the obesity epidemic, at this point in time, identifying populations with forecasted overweight predominance pinpoints where preventive interventions should be targeted, while isolating populations with current and forecasted obesity predominance pinpoints where interventions, including clinical interventions, are most urgent. The extension of adolescence to 24 years of age^49^ and the disaggregation of age into the policy-specific age bands of 5–14 years and 15–24 years (including 5-year age bands of 5–9, 10–14, 15–19, and 20–24 years) facilitate targeted advocacy, policy, and service responses. This is important for translation actions because children and young adolescents are typically in school and cared for by parents and child health services, while older adolescents are increasingly out of school and cared for by themselves and adult health services.

Limitations should also be considered. First, due to genetic variation in predisposition to disease,^53^, ^54^ universal BMI cutpoints and IOTF standardisation (for those aged <18 years) might complicate international comparisons and might not capture the optimal risk-related thresholds for all populations, and the same prevalence estimate could be associated with differing NCD risk across countries. Nonetheless, notwithstanding recent recommendations for alternative diagnostic criteria of clinical obesity at the individual level,^133^ BMI is currently considered the most feasible option for large-scale monitoring of the transition to obesity at the population level.^25^ Second, while our inclusion of 578 self-report height and weight sources to increase data volume is a strength (43·8% of all input data), these data are prone to bias, which might vary by sex and location. Despite employing updated bias-correction models to account for variations by country, year, and sex, bias could remain. Third, because of data sparseness, prevalence estimates for some countries and time periods were model driven, determined by the covariates; the accuracy of these estimates is contingent on the quality, and predictive validity, of the covariate inputs. Related, data availability was notably lower among children and adolescents aged 5–14 years compared with those aged 15–24 years. This limitation underscores the existing gap in monitoring overweight and obesity among school-aged young children and adolescents, and emphasises the need to improve population screening coverage. Finally, our forecast scenario is a reference-only scenario that assumes the continuation of past trends without considering the impact of future policy and intervention changes or treatment uptake (eg, alternative scenarios), an opportunity for future research. Although our contemporary prevalence estimates are similar to estimates produced by the NCD-RisC,^24^ despite significant overlaps between key source data (eg, DHS and STEPS), we acknowledge some differences between GBD and NCD-RisC data due to different inclusion criteria (eg, self-report *vs* measured height and weight; and different definitions of population representativeness). We also note that the number of children and adolescents forecasted to have obesity in the future is lower than in previous reports (eg, from the World Obesity Federation).^46^ We attribute this discrepancy to the continuous efforts made by GBD to refine forecasted population estimates and identification of predictive covariates.

### Conclusions

Historically, undernutrition in very young children has been a major priority for governments and donors throughout LMICs. Undernutrition requires continued investments, but global nutritional priorities must expand to include excess weight in children and adolescents. The urgency to address this comes from the timing in the transition of weight patterns from overweight to obesity predominance. Many LMICs have a short window in which investments in the overnutrition agenda will be most effective. Importantly, these interventions do not need to be at the expense of addressing undernutrition. To ensure timely policy responses, these forecasted estimates on both the timing and speed of regional-level and country-level transitions can facilitate priority setting and enable governing bodies to monitor progress. Because the rise in obesity is forecasted to continue throughout the world, political commitment to transform the diets of all children and adolescents within sustainable global food systems is now urgent. Delivered through multisectoral actions, effective multicomponent strategies targeting the multiple drivers of obesity (eg, nutrition, activity, lifestyle, and environment) are needed.

### GBD 2021 Adolescent BMI Collaborators

### Affiliations

### Contributors

### Data sharing

To download GBD data used in these analyses, please visit the GHDx GBD 2021 website. To download estimates produced in these analyses, please visit the GBD Results Tool.

## Declaration of interests

S Afzal reports support for the present manuscript from King Edward Medical University; payment or honoraria for lectures, presentations, speakers bureaus, manuscript writing or educational events from King Edward Medical University and collaborative partners including University of Johns Hopkins, University of California, University of Massachusetts, KEMCAANA, KEMCA_UK international scientific conferences, webinars and meetings; support for attending meetings and/or travel from King Edward Medical University; participation on a data safety monitoring board or advisory board with the National Bioethics Committee Pakistan, King Edward Medical University Ethical Review Board, and Ethical Review Board Fatima Jinnah Medical University and Sir Ganga Ram Hospital; leadership or fiduciary role in other board, society, committee or advocacy group (paid or unpaid) as a member of the Pakistan Association of Medical Editors, Fellow of Faculty of Public Health Royal Colleges UK (FFPH), Society of Prevention, Advocacy And Research, King Edward Medical University (SPARK), Member Pakistan Society of Infectious Diseases, and Member of the Technical Expert Advisory Group of the Government to formulate guidelines on the prevention, surveillance and research on infectious diseases; other financial or non-financial interests as Dean of Public Health and Preventive Medicine at King Edward Medical University, Chief Editor of *Annals of King Edward Medical University* since 2014, Director Quality Enhancement Cell King Edward Medical University, At International level, Fellow of Faculty of Public Health United Kingdom, Advisory Board Member and Chair Scientific Session, KEMCA-UK, Chairperson International Scientific Conference, KEMCAANA, At National level, Member Research and Publications Higher Education Commission, HEC (Pakistan), Member Research and Journals Committee Pakistan Medical and Dental Council (Pakistan), Member National Bioethics Committee (Pakistan), At Punjab Level Member Corona Experts Advisory Group, Member Technical Working Group for Infectious Diseases, Member Dengue Experts Advisory Group, and Chair, Punjab Residency Program Research Committee; outside the submitted work. S M Alif reports payment or honoraria for lectures, presentations, speakers bureaus, manuscript writing or educational events from Victoria University Online; support for attending meetings and/or travel from University of Melbourne; leadership or fiduciary role in other board, society, committee or advocacy group (paid or unpaid) with the Thoracic Society of Australia and New Zealand; outside the submitted work. R Ancuceanu reports consulting fees from AbbVie and Merck Romania; payment or honoraria for lectures, presentations, speakers bureaus, manuscript writing or educational events from AbbVie, Laropharm, Reckitt, and Merck Romania; support for attending meetings and/or travel from Merck Romania and Reckitt; outside the submitted work. J Ärnlöv reports payment or honoraria for lectures from AstraZeneca and Boehringer Ingelheim; participation on a data safety monitoring board or advisory board from AstraZeneca and Astella; outside the submitted work. O C Baltatu reports support for the present manuscript from the National Council for Scientific and Technological Development Fellowship (CNPq, 304224/2022-7), Anima Institute (AI) Research Professor Fellowship, and Alfaisal University; leadership or fiduciary role in other board, society, committee or advocacy group (paid or unpaid) with VividiWise Analytics as Managing Partner and the São José dos Campos Tech Park – CITE as Biotech Advisory Board Member; outside the submitted work. L Belo reports support from FCT in the scope of the project UIDP/04378/2020 and UIDB/04378/2020 of UCIBIO and the project LA/P/0140/2020 of i4HB; outside the submitted work. S Bhaskar reports grants or contracts from the Japan Society for the Promotion of Science (JSPS), Japanese Ministry of Education, Culture, Sports, Science and Technology (MEXT) and from The Australian Academy of Science; leadership or fiduciary roles in board, society, committee or advocacy groups (paid or unpaid) as the visiting director in the department of neurology at the National Cerebral and Cardiovascular Center, Suita (Osaka, Japan), district chair of diversity, equity and inclusion at the Rotary District 9675, chair and manager of the Global Health and Migration Hub Community (Berlin, Germany), an editorial member of *PLOS One, BMC Neurology, Frontiers in Neurology, Frontiers in Stroke, Frontiers in Aging, Frontiers in Public Health*, and *BMC Medical Research Methodology*, a member of the College of Reviewers (Canadian Institutes of Health Research, Government of Canada), a member of the scientific review committee at Cardiff University Biobank (UK), an export advisor and reviewer with the Cariplo Foundation (Milan, Italy), a Visiting Director at the National Cerebral and Cardiovascular Center, Department of Neurology, Division of Cerebrovascular Medicine and Neurology (Suita, Osaka, Japan); outside the submitted work. E J Boyko reports payment or honoraria for lectures, presentations, speakers bureaus, manuscript writing or educational events from the Korean Diabetes Association, Diabetes Association of the R.O.C (Taiwan), The American Diabetes Association, and the International Society for the Diabetic Foot; support for attending meetings/travel from the Korean Diabetes Association, Diabetes Association of the R.O.C. (Taiwan), and the International Society for the Diabetic Foot; outside the submitted work. M Carvalho reports support from FCT/MCTES under the scope of the project UIDP/50006/2020 (DOI 10.54499/UIDP/50006/2020), LAQV/REQUIMTE, University of Porto (Porto, Portugal); outside the submitted work. N Conrad reports grants or contracts from Research Foundation Flanders (grant number 12ZU922N); outside the submitted work. A A Fomenkov reports support for the present manuscript from the Ministry of Science and Higher Education of the Russian Federation (themes number 122042600086-7). R C Franklin reports support for attending meetings and/or travel from the ACTM Conference (2022-2024); leadership or fiduciary role in other board, society, committee or advocacy group (paid or unpaid) as the President of the Australasian College of Tropical Medicine; outside the submitted work. Z Guan reports grants or contracts from Dementia Centre of Excellence, Curtin enAble Institute, Curtin University; outside the submitted work. A Guha reports grants or contracts from the Department of Defense and American Heart Association; leadership or fiduciary role in other board, society, committee or advocacy group (paid or unpaid) with ZERO Cancer health disparities advisory group; outside the submitted work. A Hassan reports consulting fees from Novartis, Sanofi Genzyme, Biologix, Merck, Hikma Pharma, Janssen, Inspire Pharma, Future Pharma, Elixir pharma; payment or honoraria for lectures, presentations, speakers bureaus, manuscript writing or educational events from Novartis, Allergan, Merck, Biologix, Janssen, Roche, Sanofi Genzyme, Bayer, Hikma Pharma, Al Andalus, Chemipharm, Lundbeck, Inspire Pharma, Future Pharma and Habib Scientific Office, and Everpharma; support for attending meetings and/or travel from Novartis, Allergan, Merck, Biologix, Roche, Sanofi Genzyme, Bayer, Hikma Pharma, Chemipharm, and Al Andalus and Clavita pharm; leadership or fiduciary role in other board, society, committee or advocacy group (paid or unpaid) as Vice president of MENA headache society, Board member of Multiple Sclerosis chapter of the Egyptian Society of Neurology, Board member of headache chapter of the Egyptian Society of Neurology, member of committee of Education of the international Headache Society (IHS), membership committee of IHS, and regional committee of HIS; outside the submitted work. I Ilic reports support for the present manuscript from the Ministry of Education, Science and Technological development, Republic of Serbia (project No 175042, 2011-2023). M Ilic reports support for the present manuscript from the Ministry of Education, Science and Technological development, Republic of Serbia (number 451-03-47/2023-01/200111). N E Ismail reports leadership or fiduciary role in other board, society, committee or advocacy group (paid or unpaid) as Council Member and The Bursar of Malaysian Academy of Pharmacy (MAP) (Malaysia) and as Committee Member of Malaysian Pharmacists Society (MPS) Education Chapter Committee (Malaysia); outside the submitted work. J J Jozwiak reports payment or honoraria for lectures, presentations, speakers bureaus, manuscript writing or educational events from Novartis, Adamed, and Amgen; outside the submitted work. M S Khan reports consulting fees from Bayer and Novartis; outside the submitted work. B Lacey reports grants or contracts from UK Biobank, funded largely by the UK Medical Research Council and Wellcome; outside the submitted work. M Lee reports support for the present manuscript from the Ministry of Education of the Republic of Korea and the National Research Foundation of Korea (NRF-2023S1A3A2A05095298). M-C Li reports support for the present manuscript from the National Science and Technology Council, Taiwan (NSTC 113-2314-B-003-002); leadership or fiduciary role in other board, society, committee or advocacy group (paid or unpaid) as the technical editor of the *Journal of the American Heart Association*, outside the submitted work. D Lindholm reports stock options from AstraZeneca; and other financial/non-financial interests as a former employee of AstraZeneca; outside the submitted work. J Liu reports grants or contracts from the National Natural Science Foundation (72474005); outside the submitted work. S Lorkowski reports grants or contracts from dsm-firmenich (formerly DSM Nutritional Products) payments made to the institution; consulting fees from Danone, Novartis Pharma, and Swedish Orphan Biovitrum (SOBI); payment or honoraria for lectures, presentations, speakers bureaus, manuscript writing or educational events from AMARIN Germany, Amedes Holding, AMGEN, Berlin-Chemie, Boehringer Ingelheim Pharma, Daiichi Sankyo Deutschland, Danone, Hubert Burda Media Holding, Janssen-Cilag, Lilly Deutschland, Novartis Pharma, Novo Nordisk Pharma, Roche Pharma, Sanofi-Aventis, Swedish Orphan Biovitrum (SOBI), and SYNLAB Holding Deutschland; support for attending meetings and/or travel from AMGEN; participation on a Data Safety Monitoring Board of Advisory Board from AMGEN, Daiichi Sankyo Deutschland, Novartis Pharma, Sanofi-Aventis; outside the submitted work. H R Marateb reports grants or contracts with the Beatriu de Pinós post-doctoral programme from Agency for Management of University and Research Grants, Government of Catalonia program (#2020 BP 00261) and the Agency for Management of University and Research Grants, Knowledge Industry Grants for 2024- Modality A. LLAVOR (2024 LLAV 00083), payments made to Universitat Politècnica de Catalunya Barcelona Tech; outside the submitted work. S A Meo reports grants or contracts from the Researchers Supporting Project, King Saud University, (Riyadh, Saudi Arabia) (RSP-2025 R47); outside the submitted work. L Monasta reports support for the present manuscript from the Italian Ministry of Health (Ricerca Corrente 34/2017), payments made to the Institute for Maternal and Child Health IRCCS Burlo Garofolo. S Nomura reports support for the present manuscript from the Ministry of Education, Culture, Sports, Science and Technology of Japan (24H00663) grant and the Precursory Research for Embryonic Science and Technology from the Japan Science and Technology Agency (JPMJPR22R8) grant. B Oancea reports grants or contracts from the MRID, project PNRR-I8 no 842027778., contract no 760096; outside the submitted work. A Ortiz reports grants or contracts from Sanofi (Grant to institution: IIS-FJD UAM), and as the Director of the Catedra Mundipharma-UAM of diabetic kidney disease and the Catedra AstraZeneca-UAM of chronic kidney disease and electrolytes (Grants to Universidad Autonoma de Madrid (UAM)); consulting fees, travel fees, or speaker fees from Advicciene, Astellas, AstraZeneca, Amicus, Amgen, Fresenius Medical Care, GSK, Bayer, Sanofi-Genzyme, Menarini, Kyowa Kirin, Alexion, Idorsia, Chiesi, Otsuka, Novo-Nordisk and Vifor Fresenius Medical Care Renal Pharma; payment or honoraria for lectures, presentations, speakers bureaus, manuscript writing or educational events from Advicciene, Astellas, AstraZeneca, Amicus, Amgen, Fresenius Medical Care, GSK, Bayer, Sanofi-Genzyme, Menarini, Kyowa Kirin, Alexion, Idorsia, Chiesi, Otsuka, Novo-Nordisk and Vifor Fresenius Medical Care Renal Pharma; travel support from Advicciene, Astellas, AstraZeneca, Fresenius Medical Care, Boehringer-Ingelheim Bayer, Sanofi-Genzyme, Menarini, Chiesi, Otsuka, Sysmex; consultancy fees from Astellas, AstraZeneca, Boehringer-Ingelheim, Fresenius Medical Care, Bayer, Sanofi-Genzyme, Idorsia, Chiesi, Otsuka, Novo Nordisk, Sysmex; leadership or fiduciary role in other board, society, committee or advocacy group, unpaid from Council ERA. SOMANE; outside the submitted work. S K Panda reports support for the present manuscript from Siksha ‘O’ Anusandhan (Deemed to be University) (salary); grants or contracts from DST-GOVT. OF ODISHA (Letter Number 3444/ST); leadership or fiduciary role in other board, society, committee or advocacy group (paid or unpaid) as the Associate Editor of *Heliyon*; outside the submitted work. R Passera reports participation on a data safety monitoring board or advisory board as a member of the Data Safety Monitoring Board of the clinical trial “Consolidation with ADCT-402 (loncastuximab tesirine) after immunochemotherapy: a phase II study in BTKi-treated/ineligible Relapse/Refractory Mantle Cell Lymphoma (MCL) patients” - FIL, Fondazione Italiana Linfomi, Alessandria; leadership or fiduciary role in other board, society, committee or advocacy group (paid or unpaid) as a Member of the EBMT Statistical Committee, European Society for Blood and Marrow Transplantation, Paris (F) and as a Past member 2020–2023 (biostatistician) of the IRB/IEC Comitato Etico AO SS. Antonio e Biagio Alessandria-ASL AL-VC; outside the submitted work. S Rege reports leadership or fiduciary role in other board, society, committee or advocacy group (paid or unpaid) as Operational Lead of the International Society for Pharmacoeconomics and Outcomes Research (ISPOR) Medication Adherence and Persistence (MAP) Special Interest Group (SIG), Review Editor on the Editorial Board of Pharmacoepidemiology section within *Frontiers in Pharmacology*, and as Academic Editor of *PLoS One* (editorial board); outside the submitted work. V Sharma reports support from DFSS (MHA)'s research project (DFSS28(1)2019/EMR/6) at Institute of Forensic Science & Criminology, Panjab University (Chandigarh, India); outside the submitted work. L M L R D Silva reports grants or contracts from SPRINT, Sport Physical Activity and Health Research e Innovation Center, Polytechnic of Guarda (Portugal), and RISE–UBI Health Sciences Research Centre, University of Beira Interior (6201-506 Covilhã, Portugal); outside the submitted work. J A Singh reports consulting fees from ROMTech, Atheneum, Clearview healthcare partners, American College of Rheumatology, Yale, Hulio, Horizon Pharmaceuticals, DINORA, ANI/Exeltis, USA Inc, Frictionless Solutions, Schipher, Crealta/Horizon, Medisys, Fidia, PK Med, Two labs Inc, Adept Field Solutions, Clinical Care options, Putnam associates, Focus forward, Navigant consulting, Spherix, MedIQ, Jupiter Life Science, UBM LLC, Trio Health, Medscape, WebMD, and Practice Point communications; and the National Institutes of Health]; payment of honoraria for lectures, presentations, speakers bureaus, manuscript writing or education events as a member of the speaker's bureau of Simply Speaking; support for attending meetings as a past steering committee member of OMERACT; participation on a data safety monitoring board or advisory board with the FDA Arthritis Advisory Committee; leadership or fiduciary role in other board, society, committee or advocacy group, paid as a past steering committee member of the OMERACT (an international organization that develops measures for clinical trials and receives arm's length funding from 12 pharmaceutical companies), unpaid as a Co-Chair of the Veterans Affairs Rheumatology Field Advisory Committee, and unpaid as an editor and director of the UAB Cochrane Musculoskeletal Group Satellite Center on Network Meta-analysis; stock of stock options in Atai life sciences, Kintara therapeutics, Intelligent Biosolutions, Acumen pharmaceutical, TPT Global Tech, Vaxart pharmaceuticals, Atyu biopharma, Adaptimmune Therapeutics, GeoVax Labs, Pieris Pharmaceuticals, Enzolytics Inc, Seres Therapeutics, Tonix Pharmaceuticals Holding Corp., and Charlotte's Web Holdings, Inc and previous stock options in Amarin, Viking, and Moderna Pharmaceuticals; outside the submitted work. R Tabares-Seisdedos reports grants or contracts from Valencian Regional Government's Ministry of Education (PROMETEO/CIPROM/2022/58) and the Spanish Ministry of Science, Innovation and Universities (PID2021-129099OB-I00; the funders were not involved in the design of the manuscript or decision to submit the manuscript for publication, nor will they be involved in any aspect of the study's conduct); outside the submitted work. M V Titova reports support for the present manuscripts from the Ministry of Science and Higher Education of the Russian Federation (themes number 122042600086-7). D Trico reports payment or honoraria for lectures, presentations, speakers bureaus, manuscript writing or educational events from AstraZeneca, Eli Lilly, Novo Nordisk; support for attending meetings and/or travel from AstraZeneca; participation on a data safety monitoring board or advisory board from Amarin, Boehringer Ingelheim, Novo Nordisk; leadership or fiduciary role in other board, society, committee or advocacy group (paid or unpaid) with EASD Early Career Academy and EASD Committee on Clinical Affairs; receipt of equipment, materials, drugs, medical writing, gifts or other services from Abbott and PharmaNutra; outside the submitted work. E Upadhyay reports the following published patents: A system and method of reusable filters for anti-pollution mask; a system and method for electricity generation through crop stubble by using microbial fuel cells; a system for disposed personal protection equipment (PPE) into biofuel through pyrolysis and method; a novel herbal pharmaceutical aid for formulation of gel and method thereof; herbal drug formulation for treating lung tissue degenerated by particulate matter exposure; and a file patent for a method to transform cow dung into the wall paint by using natural materials composition thereof. E Upadhyay also reports leadership or fiduciary roles in board, society, committee or advocacy groups (paid or unpaid) with the Indian Meteorological Society, Jaipur Chapter (India) as joint secretary and DSTPURSE Program as member secretary; outside the submitted work. P Willeit reports consulting fees from Novartis Pharmaceuticals; outside the submitted work. G Zamagni reports support for the present manuscript from the Italian Ministry of Health (Ricerca Corrente 34/2017), payments made to the Institute for Maternal and Child Health IRCCS Burlo Garofolo. M Zielińska reports other financial interests as an Alexion, AstraZeneca Rare Disease employee; outside the submitted work.
