# Supplementary material for: Global, regional, and national prevalence of child and adolescent overweight and obesity, 1990–2021, with forecasts to 2050: a forecasting study for the Global Burden of Disease Study 2021
Source: Lancet. 2025 Mar 8;405(10481):785–812. doi: 10.1016/S0140-6736(25)00397-6 (PMC11920006; doi:10.1016/S0140-6736(25)00397-6)

THE LANCET

Supplementary appendix 1

This appendix formed part of the original submission and has been peer reviewed. We post it as supplied by the authors.

Supplement to: GBD 2021 Adolescent BMI Collaborators. Global, regional, and national prevalence of child and adolescent overweight and obesity, 1990–2021, with forecasts to 2050: a forecasting study for the Global Burden of Disease Study 2021. *Lancet* 2025; published online March 3. https://doi.org/10.1016/S0140-6736(25)00397-6.

Supplementary Appendix 1

Supplementary methods to:

Global, regional, and national prevalence of adult overweight and obesity, 1990-2021, with forecasts to 2050: a forecasting study for Global Burden of Disease Study 2021

and

Global, Regional, and National Prevalence of Child and Adolescent Overweight and Obesity, 1990-2021, with Forecasts to 2050: A Forecasting Study for the Global Burden of Disease Study 2021.

Supplementary results to:

Global, Regional, and National Prevalence of Child and Adolescent Overweight and Obesity, 1990-2021, with Forecasts to 2050: A Forecasting Study for the Global Burden of Disease Study 2021.

Table of Contents

Supplementary Appendix 1

Supplementary Methods 3

- Data Sources Used for Overweight and Obesity Prevalence Estimation in GBD 2021 3
  - Inclusion Criteria 3
  - Definition of overweight and obesity 3
- Data Extraction 7
- Data Standardisation and Adjustment 7
  - Age and sex splitting 7
  - Self-report bias adjustment 7
  - Prevalence estimation for overweight and obesity 11
  - Forecast modelling 11
  - Forecast model validation results 13
- GATHER Checklist 38
- References 42

Supplementary Results 43

- Supplementary figures and tables 435

Supplementary Methods

Data Sources Used for Overweight and Obesity Prevalence Estimation in GBD 2021

We searched the Global Health Data Exchange (GHDx) database for individual-level data from major multinational survey series or country-specific surveys and identified 1,873 unique sources meeting the inclusion criteria (1,321 unique sources specific for children and adolescents 5-24 years).

Inclusion Criteria

We included nationally or subnationally representative studies providing data on individual-level height and weight, mean BMI or prevalence of overweight or obesity among adults or children. For individuals ages 19 and above, studies were included if they defined overweight as BMI≥25 kg/m^2 and obesity as BMI≥30 kg/m^2, or if estimates using those cutoffs could be back-calculated from reported categories. For individuals ages 5 to 18, studies were included if they used International Obesity Task Force (IOTF) standards to define overweight and obesity thresholds. We only included studies reporting data collected between 1 January 1990 and 31 December 2021 in one of the 204 countries or territories included in this analysis (we identified data from 180 countries or territories specific for children and adolescents 5-24 years; 184 countries or territories specific to adults 25+ years). We included subnational results for 20 countries: Brazil, China, Ethiopia, Great Britain, India, Indonesia, Iran, Italy, Japan, Kenya, Mexico, New Zealand, Nigeria, Norway, Pakistan, Philippines, Poland, Russia, South Africa, and the United States. Studies were excluded if using non-random samples (e.g., case-control studies or convenience samples); conducted among specific subpopulations (e.g., pregnant females, racial or ethnic minorities, immigrants, or individuals with specific diseases); using alternative methods to assess adiposity (e.g., waist-circumference, skin-fold thickness, or hydrodensitometry); having sample sizes of less than 20 per 5-year age-sex group; or providing inadequate information on any of the inclusion criteria. We also excluded review articles and non-English articles. Figure S1 shows the distribution of data availability across countries. Following exclusions, 222 sources were removed, totalling 2,517,657/99,961,063 individuals among all age groups (2.5%) and 1,463,615/17,500,219 individuals between ages 5-24 years (8.4%).

Definition of overweight and obesity

For individuals aged over 18 years, we considered them to have overweight if their BMI was greater than or equal to 25 kg/m^2, and to have obesity if their BMI was greater than or equal to 30 kg/m^2. For individuals aged 5-18 years, we used monthly IOTF cutoffs [1] to determine overweight and obese status when age in months was available. When only age in years was available, we used the cutoff for the midpoint of that year. We excluded studies using the World Health Organization (WHO) standards or country-specific cutoffs to define childhood overweight and obesity. At the individual level, we considered BMI < 8 kg/m^2 or BMI > 80 kg/m^2 to be biologically implausible and excluded those observations. The rationale for choosing to use the IOTF cutoffs over the WHO standards has been described elsewhere. [1] Briefly, the IOTF cutoffs provide consistent child-specific standards for ages 5-18 derived from surveys covering multiple countries. In contrast, the WHO Child Growth Standards apply to children under age 5, and the WHO growth reference applies to children and adolescents ages 5–19. The WHO growth reference for children and adolescents ages 5–19 was derived using United States data as

the core sample,[2] which are less representative than the multinational data used by IOTF. Additionally, the switch between references at age 5 can produce artificial discontinuities. Given that we estimate global childhood overweight and obesity for ages 5–19 (with ages 19 using standard adult cutoffs), the IOTF cutoffs were preferable. Additionally, we found that IOTF cutoffs were more commonly used in scientific literature covering childhood obesity.

Figure S1: Number of data sources used to estimate (a) children and adolescent and (b) adult overweight and obesity prevalence 1990-2021

(a)

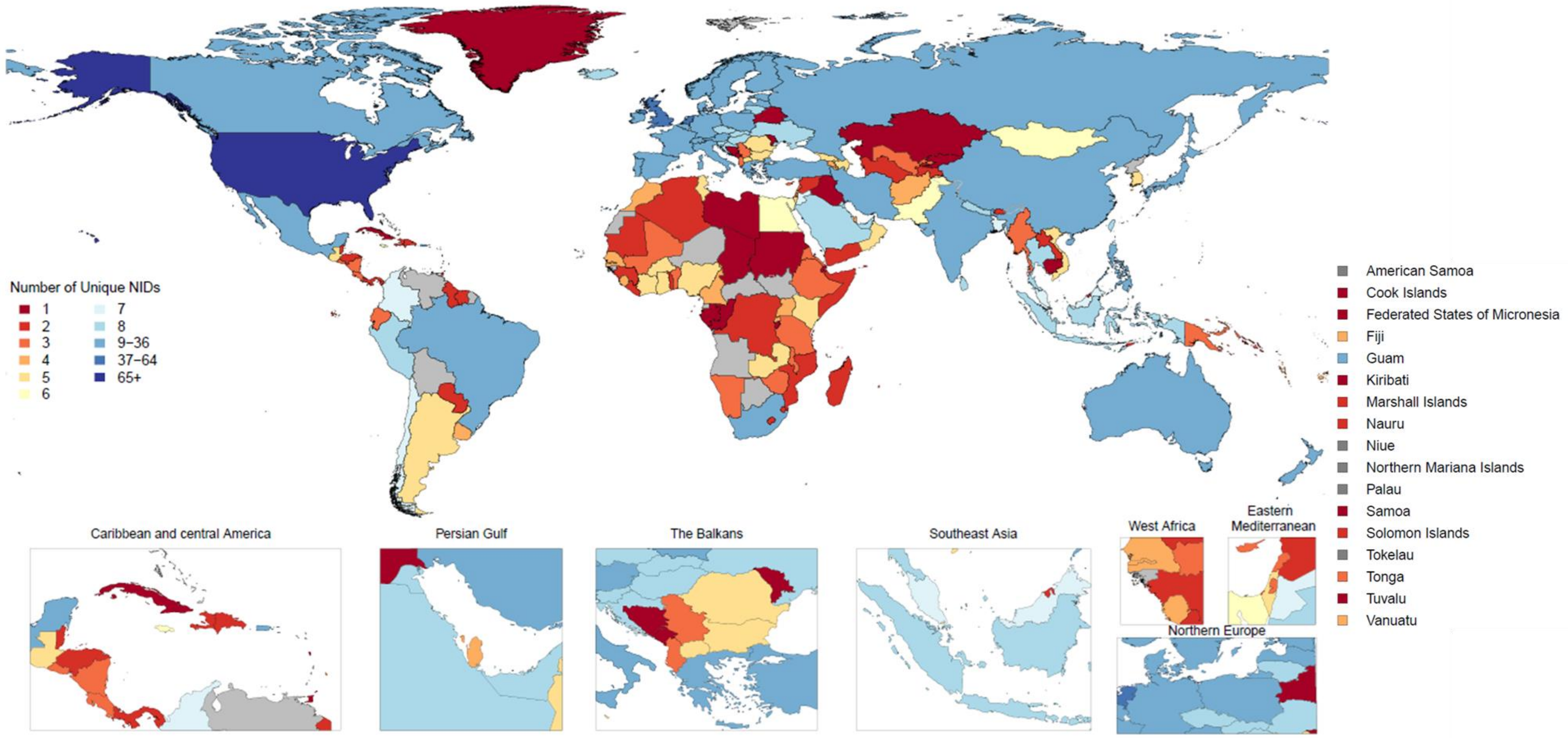

(b)

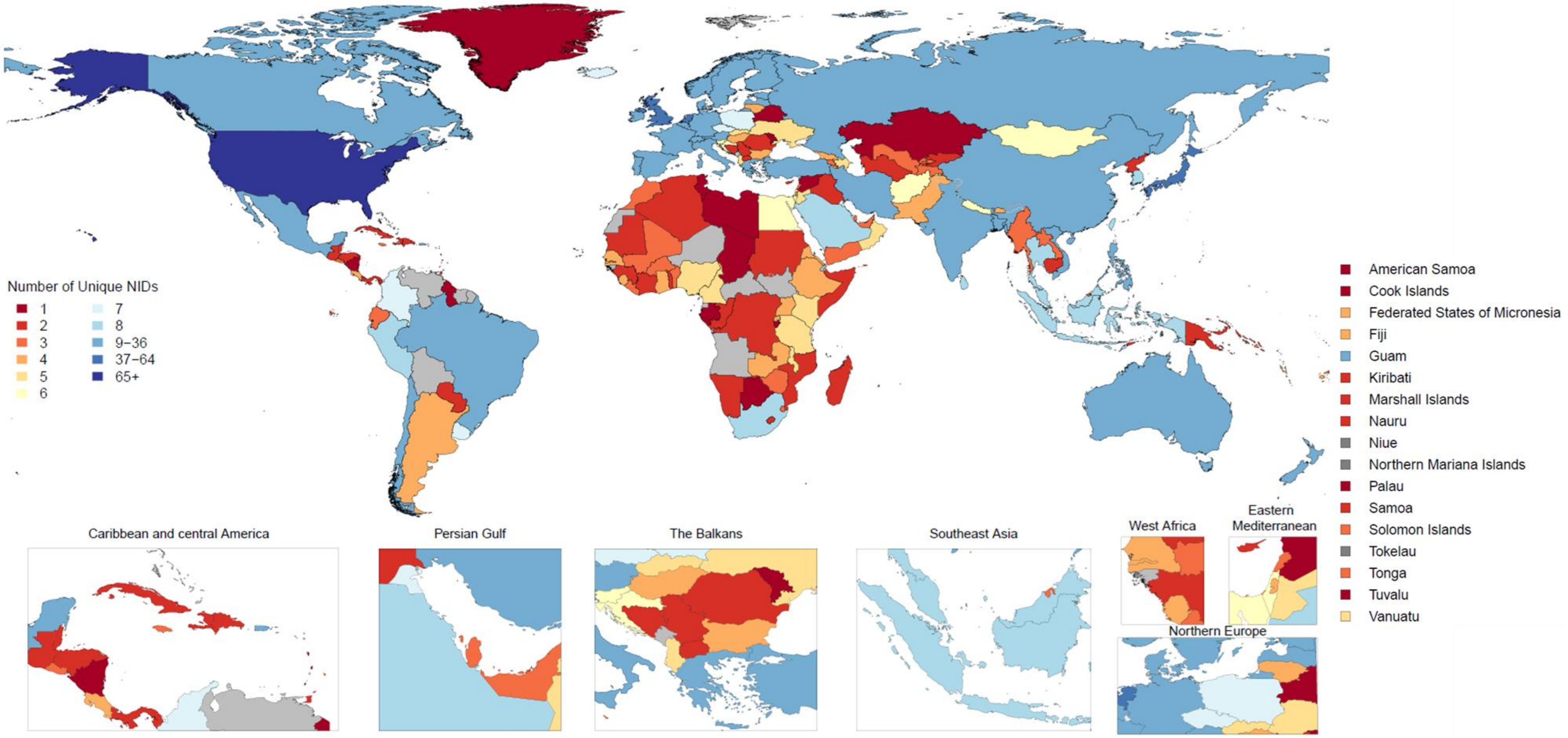

Number of Unique NIDs
1
2
3
4
5
6
7
8
9–36
37–64
65+
Caribbean and central America
Persian Gulf
The Balkans
Southeast Asia
West Africa
Eastern Mediterranean
Northern Europe
American Samoa
Cook Islands
Federated States of Micronesia
Fiji
Guam
Kiribati
Marshall Islands
Nauru
Niue
Northern Mariana Islands
Palau
Samoa
Solomon Islands
Tokelau
Tonga
Tuvalu
Vanuatu

Data Extraction

Where individual-level survey data were available, we computed individual-level BMI using weight and height and then used the BMI to determine whether the individual had overweight or obesity. We then aggregated the individual-level data by age group and sex to produce age- and sex-specific prevalence of overweight and obesity. In addition, we extracted relevant survey-design variables, including primary sampling unit, strata, and survey weights, which were used to tabulate individual-level microdata and produce accurate measures of uncertainty. We extracted three study-level covariates: 1) whether height and weight data were measured or self-reported, 2) whether the study was predominantly conducted in an urban area, rural area, or both, and 3) the level of representativeness of the study (national or subnational). Finally, we extracted relevant demographic indicators, including location, year, age, and sex. We estimated the standard error of the mean from individual-level data where available and used the reported standard error of the mean for published data.

Similarly, from report and literature data, we extracted data on mean BMI, prevalence of overweight, and prevalence of obesity, measures of uncertainty for each, and sample size, by the most granular age and sex groups available. We extracted the same study-level covariates as were extracted from microdata (measurement, urbanicity, and representativeness), as well as all demographic indicators as mentioned previously.

When multiple data sources were available for the same country, we included all of them in our analysis. If data from the same data source were available in multiple formats such as individual-level data and tabulated data, individual-level data were prioritised.

Data Standardisation and Adjustment

Age and sex splitting

Any report or literature data provided in age groups wider than the standard five-year age groups or as both sexes combined were split using the approach used by Ng and colleagues.[3] Briefly, age-sex patterns were modelled with spatiotemporal Gaussian process regression (ST-GPR) leveraging data sources reporting in sex-specific, standard five-year age units. Considering the large heterogeneity in overweight and obesity prevalence across geographical regions, instead of applying a global age pattern upon all countries, we segmented countries into tertiles based on the level of overweight and obesity prevalence. Tertile-specific age-pattern was derived by averaging the modelled age- and sex- patterns of all locations within the tertile. The tertile-specific patterns were subsequently applied to split report and literature data based on the data source's location and its respective tertile of overweight or obesity *prevalence.* We did not propagate the uncertainty in the age pattern and sex pattern used to split the data as they seemed to have small effect.

Self-report bias adjustment

We included both measured and self-reported data. Of 50,310,211 person-years of data, 28,511,589 (56.7%) were self-reported (43.8% for children and adolescents; 578 sources). We tested for bias in self-report data compared to measured data, which is considered to be the gold-standard. There was no clear direction of bias for children ages 5–14, so for these age groups we only included measured data. For individuals ages 15 and above, we adjusted self-reported data for overweight prevalence and obesity

prevalence. We used MR-BRT[4] to determine the level of self-report bias adjustment. For both overweight and obesity, we fit sex-specific MR-BRT models on the logit difference between measured and self-reported with a fixed effect on super-region. The bias coefficients derived from these two models are in Tables S1 and S2.

Given the unique magnitude of the obesity epidemic and to maixmise the use of available data in the USA, a separate self-report bias adjustment was completed for the country. Self-report data was compared to measured data from the NHANES survey series, which were selected as the gold standard for the USA. We used MR-BRT to determine the level of self-report bias adjustment. For both overweight and obesity, we fit sex-specific MR-BRT models on the logit difference between NHANES measured and self-reported with a fixed effect on 5-year age groups and decade when the data was collected. The bias coefficients derived from these two models are in Table S3.

Table S1: MR-BRT self-report crosswalk adjustment factors for overweight prevalence for all countries except the USA

**Model**	**Data input**	**Reference or alternative case definition**	**Gamma**	**Beta coefficient, logit (95% CI)**
Females	Measured data	Ref	0.26	---
	Self-reported data (Southeast Asia, East Asia, and Oceania)	Alt		-0.53 (-1.03, -0.04)
	Self-reported data (Central Europe, Eastern Europe, and Central Asia)	Alt		-0.20 (-0.69, 0.30)
	Self-reported data (High-income)	Alt		-0.25 (-0.75, 0.24)
	Self-reported data (Latin America and Caribbean)	Alt		-0.19 (-0.69, 0.31)
	Self-report data (North Africa and Middle East)	Alt		-0.38 (-0.89,0.11)
	Self-report data (South Asia)	Alt		0.36 (-0.14, 0.85)
	Self-report data (Sub-Saharan Africa)	Alt		-0.26 (-0.76, 0.24)
Men	Measured data	Ref	0.43	---
	Self-reported data (Southeast Asia, East Asia, and Oceania)	Alt		-0.36 (-1.17, 0.50)
	Self-reported data (Central Europe, Eastern Europe, and Central Asia)	Alt		-0.03 (-0.84, 0.82)
	Self-reported data (High-income)	Alt		0.05 (-0.77, 0.87)

	Self-reported data (Latin America and Caribbean)	Alt		-0.02 (-0.84, 0.81)
	Self-report data (North Africa and Middle East)	Alt		-0.21 (-1.04, 0.61)
	Self-report data (South Asia)	Alt		0.53 (-0.28, 1.37)
	Self-report data (Sub-Saharan Africa)	Alt		-0.27 (-1.09, 0.55)

Table S2: MR-BRT self-report crosswalk adjustment factors for obesity prevalence for all countries except the USA

**Model**	**Data input**	**Reference or alternative case definition**	**Gamma**	**Beta coefficient, logit (95% UI) ***
Females	Measured data	Ref	0.38	---
	Self-reported data (Southeast Asia, East Asia, and Oceania)	Alt		-0.11 (-0.86, 0.64)
	Self-reported data (Central Europe, Eastern Europe, and Central Asia)	Alt		-0.95 (-1.70, -0.19)
	Self-reported data (High-income)	Alt		-0.42 (-1.16, 0.34)
	Self-reported data (Latin America and Caribbean)	Alt		-0.41 (-1.16, 0.34)
	Self-report data (North Africa and Middle East)	Alt		-0.48 (-1.23, 0.27)
	Self-report data (South Asia)	Alt		0.50 (-0.25, 1.26)
	Self-report data (Sub-Saharan Africa)	Alt		-0.41 (-1.16, 0.34)
Men	Measured data	Ref	0.74	
	Self-reported data (Southeast Asia, east Asia, and Oceania)	Alt		0.04 (-1.41, 1.53)
	Self-reported data (Central Europe, Eastern Europe, and central Asia)	Alt		-0.79 (-2.25, 0.71)
	Self-reported data (High-income)	Alt		-0.13 (-1.58, 1.40)
	Self-reported data (Latin America and Caribbean)	Alt		-0.26 (-1.70, 1.21)

	Self-report data (North Africa and Middle East)	Alt		-0.33 (-1.77, 1.16)
	Self-report data (South Asia)	Alt		0.66 (-0.78, 2.15)
	Self-report data (Sub-Saharan Africa)	Alt		-0.41 (-1.86, 1.08)

**MR-BRT crosswalk adjustments can be interpreted as the factor the alternative case definition is adjusted by to reflect what it would have been had it been measured using the reference case definition. If the log/logit beta coefficient is negative, then the alternative is adjusted up to the reference. If the log/logit beta coefficient is positive, then the alternative is adjusted down to the reference.*

Table S3: MR-BRT self-report crosswalk adjustment factors for overweight and obesity prevalence for the USA

**Model**	**Data input**	**Reference or alternative case definition**	**Gamma**	**Beta coefficient, logit (95% UI) ***
**Overweight prevalence**				
Females	Measured NHANES data	Ref	0.0052	---
	Self-report (intercept)	Alt		0.08 (0.05, 0.11)
	Self-report (5-year age group)	Alt		–0.02 (–0.02, -0.01)
	Self-report data (decade)	Alt		0 (–0.02, 0.02)
Males	Measured NHANES data	Ref	0.016	---
	Self-report (intercept)	Alt		-0.42 (-0.46, -0.37)
	Self-report (5-year age group)	Alt		-0.003 (–0.005, -0.001)
	Self-report data (decade)	Alt		0 (–0.03, 0.03)
**Obesity prevalence**				
Females	Measured NHANES data	Ref	0.012	---
	Self-report (intercept)	Alt		-0.45 (-0.49, -0.41)
	Self-report (5-year age group)	Alt		0.003 (0.001, 0.004)
	Self-report data (decade)	Alt		0.01 (-0.02, 0.04)
Males	Measured NHANES data	Ref	0.018	---
	Self-report (intercept)	Alt		-0.46 (-0.50, -0.41)
	Self-report (5-year age group)	Alt		0 (–0.002, 0.001)
	Self-report data (decade)	Alt		0.01 (–0.02, 0.04)

**MR-BRT crosswalk adjustments can be interpreted as the factor the alternative case definition is adjusted by to reflect what it would have been had it been measured using the reference case definition. If the log/logit beta coefficient is negative, then the alternative is adjusted up to the reference. If the log/logit beta coefficient is positive, then the alternative is adjusted down to the reference.*

Prevalence estimation for overweight and obesity

After adjusting for self-report bias and splitting aggregated data into five-year age-sex groups, we used ST-GPR to estimate the prevalence of overweight and obesity. This modelling approach has been described in detail elsewhere.[3]

The linear model, which when added to the smoothed residuals forms the mean prior for GPR is as follows:

$$\text{logit}(\text{overweight})_{c,a,t} = \beta_0 + \beta_1 \text{educ}_{c,t} + \beta_2 \text{urban}_{c,t} + \beta_3 \text{agriculture}_{c,t} + \sum_{k=1}^{16} \beta_k I_{A[a]} + \alpha_s + \alpha_r + \alpha_c$$

$$\text{logit}(\text{obesity/overweight})_{c,a,t} = \beta_0 + \beta_1 \text{educ}_{c,t} + \beta_2 \text{urban}_{c,t} + \beta_3 \text{agriculture}_{c,t} + \sum_{k=1}^{16} \beta_k I_{A[a]} + \alpha_s + \alpha_r + \alpha_c$$

where educ *is the age-standardized level of educational attainment;* urban *is the proportion of the population living in an urban area ; and* agriculture *is the proportion of the population working in agriculture.* $I_{A[a]}$ is a dummy variable indicating a specific age group A that the prevalence point captures, and α_s, α_r, and α_c are super-region, region, and country nested random intercepts, respectively. Random effects were used in model fitting but were not used in prediction.

We tested all combinations of the following covariates to see which performed best in terms of in-sample AIC for the overweight linear model and the obesity as a proportion of overweight linear model: ten-year lag-distributed energy per capita, proportion of the population living in urban areas, SDI, lag-distributed income per capita, educational attainment (years) per capita, proportion of the population working in agriculture, grams of sugar adjusted for energy per capita, grams of sugar not adjusted for energy per capita, and the number of two- or four-wheeled vehicles per capita. We selected these candidate covariates based on theory as well as reviewing covariates used in other publications. The final linear model was selected based on 1) if the direction of covariates matched what is expected from theory, 2) all the included covariates were significant, and 3) minimising in-sample AIC. The covariate selection process was performed using the dredge package in R.

Forecast modelling

To forecast the prevalence of overweight and obesity, as well as the proportion of obesity among the overweight, we used a generalised ensemble modelling approach (GenEM) which consisted of 12 different submodels.[5] For the submodels, we employed two approaches: annualised rate of change (ARC) and a two-stage spline model based on the Meta-Regression Bayesian Regularized Trimmed Tool (MR-BRT).[6] Each of these sub-models had one of six different recency-weighting parameters[7] ranging from 0 to 2.5, with higher values giving more weight to recent years.[7]

For the ARC submodels, we calculated the sex-and-location specific annualised rate of change for the logit-transformed age-standardized prevalence of overweight and obesity and the logit-transformed age-standardized proportion of obesity among the overweight. The annual change values are winsorised by replacing outliers with the closest 2.5th and 97.5th percentile values. As for the two-stage MR-BRT submodels, the first stage involved fitting a sex-specific logit spline model separately for each of the metrics of interest (1) the age-standardized prevalence of overweight and obesity and (2) the age-standardized proportion of obesity among the overweight against the socio-demographic index (SDI):

$$logit(\text{overweight}_{s,t,c}) = \beta_0 + \beta_1 spline(SDI_{t,c}) + \varepsilon_{s,t,c}$$

$$logit(\text{obesity/overweight}_{s,t,c}) = \beta_0 + \beta_1 spline(SDI_{t,c}) + \varepsilon_{s,t,c}$$

where $logit(\text{overweight}_{s,t,c})$ is the logit of the overweight and obesity age-standardized prevalence and $logit(\text{obesity/overweight}_{s,t,c})$ is the age-standardized proportion of obesity among the overweight, for sex s, year t, and country c. β_0 is the intercept, β_1 is a coefficient matrix; $spline$ is the piecewise polynomial function with five knots evenly placed across the curve and with the assumption of right and left linear tails; and $\varepsilon_{s,t,c}$ refers to the residuals. In the second stage of the model, the logit of the residuals from each of first-stage model is linearly modeled on time:

$$logit(\varepsilon_{s,t,c}) = \beta'_0 + \beta'_1 t + \zeta_{s,t,c}$$

where β'_0 is the fixed intercept, β'_1 is the coefficient for years and $\zeta_{s,t,c}$ is an error term. We also ran the models separately for adult (aged 25 +) and child (aged 5-24, 5-14, and 15-24) age groups to differentiate the trends between younger and older populations.

To create the ensemble model, each submodel was weighted based on its performance using out of sample validation. Specifically, out-of-sample cross-validation was performed where all submodels were trained using data from 1990-2011. Prediction errors, defined by root mean square errors (RMSE), were calculated based on a 10-year holdout period from 2012 to 2021. Subsequently, model weights based on the inverse of their RMSE were derived, with models demonstrating better out-of-sample predictive performance receiving higher weights.

For the final forecast, submodels were trained using the complete dataset from 1990-2021. For each ARC submodel, we used the calculated annualised rate of change with corresponding recency-weighting parameters to derive prevalence estimates for 2022-2050. For the MR-BRT submodels, forecast SDI values for 2022-2050 and recency weights were used to obtain forecasted age-standardized prevalence values. The forecasted age-standardized prevalence draws from all models were combined using predictive performance weights. The age-specific forecasted prevalence results were obtained by applying the age weights to the forecasted age-standardized prevalence. The forecasted prevalence of obesity was then calculated by multiplying the forecasted prevalence of overweight and obesity by the forecasted proportion of obesity among the overweight. The forecasted prevalence of overweight excluding obesity was calculated by subtracting prevalence of obesity from prevalence of overweight and obesity.

Reference population forecasts from 2022 to 2050 were used to compute the number of individuals with overweight and obesity and to aggregate results by location, age and sex.[5] Population was forecasted from all-cause mortality forecasts, migration forecasts, and fertility forecasts with female education, met need for modern contraceptives, under-5 mortality, and urbanicity as covariates.[8,9] This age and sex-specific forecasted population was produced for 204 countries and accounts for population dynamics such as aging and changes in population size.

All final forecast values were calculated as the mean of the 500 draws from the posterior distribution, and uncertainty estimates were derived from the 2.5th and 97.5th percentiles of the distribution. Additional details on these methods can be found in Vollset et al, 2024.[5]

Forecast model validation results

Table S4 Out-of-sample (OOS) RMSE and draws selected for prevalence of overweight forecast sub-models

	ARC (draws | RMSE)						MR-BRT (draws | RMSE)					
Location	omega 0	omega 0.5	omega 1	omega 1.5	omega 2	omega 2.5	omega 0	omega 0.5	omega 1	omega 1.5	omega 2	omega 2.5
Armenia	49| 0.00838	52| 0.00791	54| 0.00766	54| 0.00757	57| 0.00755	54| 0.00758	34| 0.01218	33| 0.01259	31| 0.0134	29| 0.01428	27| 0.01507	26| 0.01572
Azerbaijan	56| 0.00656	61| 0.00605	65| 0.00565	69| 0.00535	72| 0.00513	76| 0.00496	19| 0.01929	18| 0.01995	17| 0.02109	16| 0.02223	16| 0.02323	15| 0.02404
Georgia	79| 0.00675	67| 0.00767	61| 0.00841	57| 0.009	54| 0.00945	52| 0.00981	20| 0.025	22| 0.02366	22| 0.02331	22| 0.02326	22| 0.02332	22| 0.02339
Kazakhstan	51| 0.00515	54| 0.00489	55| 0.00474	56| 0.00466	57| 0.00462	59| 0.0046	29| 0.00911	29| 0.00906	29| 0.00916	28| 0.00945	27| 0.0098	26| 0.0101
Kyrgyzstan	59| 0.00358	70| 0.00304	78| 0.00274	82| 0.00259	84| 0.00253	87| 0.00251	9| 0.02389	7| 0.02901	6| 0.03289	6| 0.03497	6| 0.03561	6| 0.03545
Mongolia	58| 0.01008	49| 0.01131	44| 0.01242	41| 0.01334	39| 0.01408	37| 0.01466	30| 0.01837	38| 0.01453	42| 0.0131	42| 0.013	41| 0.01348	39| 0.01418
Tajikistan	53| 0.00783	61| 0.00689	66| 0.00631	70| 0.00596	73| 0.00577	78| 0.00565	17| 0.0238	17| 0.0243	17| 0.02478	16| 0.02524	16| 0.02566	16| 0.02602
Turkmenistan	57| 0.00534	57| 0.0053	57| 0.00526	59| 0.00516	60| 0.005	65| 0.00488	28| 0.01092	26| 0.01144	25| 0.01225	23| 0.01302	22| 0.01364	21| 0.01413
Uzbekistan	50| 0.01003	44| 0.01074	42| 0.01132	41| 0.01177	39| 0.01211	39| 0.01236	29| 0.01641	37| 0.01299	42| 0.01136	45| 0.01068	46| 0.01044	46| 0.01041
Albania	45| 0.0105	47| 0.0099	49| 0.00953	51| 0.00921	53| 0.00892	56| 0.00864	38| 0.01242	36| 0.01299	34| 0.01381	32| 0.01468	30| 0.01548	29| 0.01616
Bosnia and Herzegovina	35| 0.01563	43| 0.01246	53| 0.01028	61| 0.00883	69| 0.00786	80| 0.00719	25| 0.02115	26| 0.02069	26| 0.02043	27| 0.02011	27| 0.01965	28| 0.01906
Bulgaria	49| 0.00425	55| 0.00381	59| 0.00352	63| 0.00331	67| 0.00314	73| 0.003	29| 0.00717	25| 0.00845	22| 0.00944	20| 0.01015	19| 0.01063	19| 0.01097
Croatia	34| 0.00733	44| 0.00569	57| 0.00443	70| 0.00361	79| 0.00319	86| 0.00307	22| 0.01135	23| 0.01085	22| 0.01151	21| 0.01201	21| 0.01218	21| 0.01211
Czechia	55| 0.00602	56| 0.00594	57| 0.00586	58| 0.00572	60| 0.00552	68| 0.00535	25| 0.01305	26| 0.01265	26| 0.01258	25| 0.01324	23| 0.01439	21| 0.01567
Hungary	55| 0.00376	60| 0.0034	66| 0.00311	71| 0.00289	76| 0.00272	82| 0.00259	18| 0.01126	17| 0.01208	16| 0.01266	15| 0.01389	13| 0.01569	11| 0.01769
Montenegro	59| 0.00439	62| 0.00416	65| 0.00398	67| 0.00385	69| 0.00375	73| 0.00368	18| 0.01412	19| 0.01311	18| 0.01402	17| 0.01493	17| 0.01542	16| 0.01554
North Macedonia	65| 0.0051	61| 0.00516	60| 0.00529	58| 0.00542	57| 0.00553	56| 0.00562	29| 0.01067	25| 0.01242	23| 0.01373	22| 0.01434	22| 0.01452	22| 0.0145
Poland	45| 0.01108	48| 0.01032	51| 0.00972	54| 0.00926	56| 0.0089	61| 0.00862	38| 0.01297	35| 0.01405	33| 0.01505	30| 0.01666	26| 0.01895	23| 0.02156
Romania	44| 0.00338	57| 0.00263	70| 0.00216	79| 0.0019	85| 0.00177	92| 0.00171	17| 0.00869	13| 0.01117	12| 0.01279	11| 0.01364	10| 0.01404	10| 0.01424
Serbia	55| 0.0057	60| 0.00524	64| 0.00489	68| 0.00463	71| 0.00444	76| 0.0043	18| 0.0177	20| 0.01562	19| 0.01685	17| 0.01831	16| 0.01921	16| 0.01962
Slovakia	68| 0.00509	64| 0.00519	63| 0.00525	62| 0.00528	62| 0.00529	62| 0.00528	22| 0.01484	21| 0.01523	21| 0.01567	20| 0.01671	18| 0.01815	17| 0.01965
Slovenia	60| 0.00361	65| 0.00335	67| 0.00323	71| 0.0032	68| 0.0032	68| 0.00322	15| 0.01416	16| 0.01355	17| 0.01294	17| 0.01243	18| 0.01207	18| 0.0119
Belarus	46| 0.00855	46| 0.00842	47| 0.00826	48| 0.00812	49| 0.00798	54| 0.00787	46| 0.00842	39| 0.00991	34| 0.01131	32| 0.0122	30| 0.01277	29| 0.01318
Estonia	69| 0.00697	56| 0.00813	51| 0.00887	49| 0.00928	48| 0.00949	48| 0.00957	36| 0.01268	34| 0.01352	30| 0.01494	28| 0.01639	26| 0.01752	25| 0.01829

Latvia	67| 0.00572	59| 0.00607	55| 0.00653	52| 0.00689	50| 0.00715	49| 0.00733	30| 0.01208	29| 0.01258	28| 0.01293	27| 0.01316	27| 0.01331	27| 0.01342
Lithuania	52| 0.00691	57| 0.00654	54| 0.00671	52| 0.00696	50| 0.00716	49| 0.00731	31| 0.01164	32| 0.01123	31| 0.01145	31| 0.01162	31| 0.01172	30| 0.01178
Republic of Moldova	51| 0.00925	51| 0.00913	52| 0.00901	53| 0.00889	53| 0.00879	58| 0.0087	22| 0.02156	29| 0.01595	35| 0.01328	35| 0.01335	32| 0.01467	29| 0.0162
Russian Federation	51| 0.01168	50| 0.01189	50| 0.01184	51| 0.01166	52| 0.01141	55| 0.01115	33| 0.01807	32| 0.01815	32| 0.01825	32| 0.01845	31| 0.01871	31| 0.01896
Ukraine	56| 0.00764	52| 0.00765	52| 0.00774	51| 0.00786	50| 0.00797	50| 0.00807	32| 0.01253	36| 0.01102	35| 0.01155	31| 0.01283	28| 0.01407	27| 0.01504
Australia	41| 0.01255	42| 0.0123	44| 0.01173	47| 0.01107	50| 0.01042	56| 0.00984	40| 0.01309	39| 0.01331	38| 0.01378	36| 0.0144	34| 0.01502	33| 0.01553
New Zealand	54| 0.00946	50| 0.00966	50| 0.00983	49| 0.00995	49| 0.01002	48| 0.01006	36| 0.01333	37| 0.01316	35| 0.01381	33| 0.01493	30| 0.01608	29| 0.01698
Brunei Darussalam	36| 0.01645	35| 0.01676	35| 0.01681	36| 0.01658	36| 0.01656	36| 0.01654	44| 0.01345	45| 0.01336	47| 0.01277	49| 0.01225	53| 0.01206	48| 0.0123
Japan	60| 0.0059	55| 0.00597	55| 0.00596	55| 0.00593	55| 0.00591	55| 0.0059	26| 0.01239	27| 0.01191	28| 0.01161	28| 0.01147	28| 0.01142	28| 0.01141
Republic of Korea	36| 0.01058	45| 0.00863	52| 0.00743	57| 0.00676	60| 0.00645	62| 0.00632	30| 0.0129	31| 0.01252	32| 0.01213	32| 0.01199	32| 0.01211	31| 0.01241
Singapore	39| 0.01284	52| 0.00957	61| 0.00818	64| 0.00776	66| 0.00769	64| 0.00777	26| 0.01879	26| 0.01895	26| 0.01911	26| 0.01927	25| 0.01945	25| 0.01965
Canada	44| 0.0066	49| 0.00596	52| 0.00564	53| 0.00551	54| 0.00547	57| 0.00547	31| 0.00942	32| 0.00927	32| 0.00918	32| 0.00914	32| 0.00915	32| 0.00921
Greenland	55| 0.00439	61| 0.00398	64| 0.0038	66| 0.00377	63| 0.00384	61| 0.00395	26| 0.00941	23| 0.01036	21| 0.0113	20| 0.01181	20| 0.01202	20| 0.01208
United States of America	38| 0.02379	44| 0.02035	51| 0.01768	57| 0.01577	62| 0.01442	69| 0.01345	25| 0.03614	28| 0.03191	31| 0.02891	32| 0.02752	32| 0.02752	31| 0.02847
Alabama	39| 0.02114	48| 0.01725	57| 0.01444	66| 0.0125	73| 0.01126	83| 0.01044	19| 0.04235	22| 0.03685	24| 0.03398	24| 0.03363	23| 0.03506	22| 0.03749
Alaska	42| 0.03037	45| 0.02829	48| 0.0264	51| 0.02486	53| 0.02365	57| 0.0227	33| 0.03821	33| 0.03791	34| 0.03741	34| 0.03683	35| 0.03639	35| 0.03623
Arizona	45| 0.02884	48| 0.02734	51| 0.0257	54| 0.02418	57| 0.02288	64| 0.02174	23| 0.05674	29| 0.04578	32| 0.04026	33| 0.03903	33| 0.04016	31| 0.04224
Arkansas	41| 0.02386	45| 0.02191	49| 0.02019	53| 0.01881	56| 0.01778	61| 0.01698	27| 0.03626	30| 0.03278	33| 0.03003	35| 0.02847	35| 0.02802	35| 0.0284
California	35| 0.02591	44| 0.02078	53| 0.01737	59| 0.0154	63| 0.01444	69| 0.01399	24| 0.03855	27| 0.03329	31| 0.02978	32| 0.02817	32| 0.02819	31| 0.02929
Colorado	44| 0.02308	49| 0.02099	54| 0.01897	59| 0.0174	63| 0.0163	69| 0.01551	22| 0.04624	25| 0.04068	28| 0.0367	29| 0.03483	29| 0.03464	29| 0.03545
Connecticut	42| 0.0241	44| 0.02315	45| 0.02243	46| 0.02197	47| 0.02172	50| 0.02162	35| 0.02909	36| 0.02779	38| 0.02668	39| 0.02601	39| 0.02585	39| 0.02613
Delaware	45| 0.02076	47| 0.01985	49| 0.01882	52| 0.01787	54| 0.01708	61| 0.01641	28| 0.03263	31| 0.02976	33| 0.02786	34| 0.02723	34| 0.02758	32| 0.02855
District of Columbia	58| 0.01499	62| 0.01465	58| 0.01498	56| 0.01547	54| 0.01592	53| 0.01626	29| 0.03012	28| 0.03096	27| 0.032	26| 0.03313	25| 0.03427	24| 0.03537
Florida	38| 0.02929	42| 0.02591	47| 0.02324	52| 0.02121	56| 0.0197	62| 0.01851	28| 0.03939	32| 0.03399	36| 0.03064	37| 0.0298	36| 0.03071	34| 0.0325
Georgia	44| 0.02444	51| 0.02083	57| 0.01856	62| 0.01718	65| 0.01637	71| 0.01586	20| 0.05298	24| 0.04492	26| 0.04012	27| 0.03864	27| 0.03927	26| 0.04089
Hawaii	39| 0.03394	44| 0.03019	48| 0.02731	52| 0.02518	56| 0.02362	61| 0.02243	31| 0.04303	32| 0.04173	33| 0.0403	34| 0.03896	35| 0.03793	35| 0.03734
Idaho	35| 0.03003	41| 0.02602	46| 0.02284	52| 0.02043	57| 0.01861	64| 0.01722	29| 0.03672	33| 0.03216	36| 0.02905	38| 0.02814	36| 0.02926	33| 0.03169
Illinois	42| 0.02144	48| 0.01879	52| 0.01737	54| 0.01665	56| 0.01626	59| 0.016	28| 0.03213	31| 0.02933	32| 0.02776	33| 0.02722	33| 0.0274	32| 0.02804
Indiana	47| 0.02087	50| 0.01962	52| 0.01893	53| 0.01856	54| 0.01839	58| 0.01831	29| 0.03415	31| 0.0322	32| 0.03111	32| 0.03091	31| 0.03138	31| 0.03228

Iowa	46| 0.0181	52| 0.01609	57| 0.01454	62| 0.01344	66| 0.01269	71| 0.01218	22| 0.03805	23| 0.03565	25| 0.03365	25| 0.03253	26| 0.03241	25| 0.03308
Kansas	50| 0.02023	52| 0.01927	56| 0.01789	61| 0.01652	65| 0.01539	72| 0.01448	20| 0.05103	21| 0.048	23| 0.04401	25| 0.04016	27| 0.03735	28| 0.03596
Kentucky	37| 0.02619	44| 0.02231	51| 0.01933	58| 0.0171	64| 0.01547	72| 0.01421	27| 0.03677	28| 0.03444	30| 0.03281	30| 0.03221	30| 0.03259	29| 0.03371
Louisiana	40| 0.0244	44| 0.02259	47| 0.02113	49| 0.01997	52| 0.01904	58| 0.01828	32| 0.03057	34| 0.02864	36| 0.02749	36| 0.02708	36| 0.02721	36| 0.0277
Maine	45| 0.02377	48| 0.022	51| 0.02062	54| 0.0196	56| 0.01885	62| 0.01829	30| 0.03525	30| 0.03482	31| 0.03433	31| 0.03407	31| 0.03413	31| 0.03449
Maryland	36| 0.02786	40| 0.02546	42| 0.02374	45| 0.0225	47| 0.0216	51| 0.0209	35| 0.02884	38| 0.02644	41| 0.02471	42| 0.02393	42| 0.02395	41| 0.02451
Massachusetts	41| 0.0241	43| 0.02297	44| 0.02205	46| 0.02133	47| 0.02078	51| 0.02036	38| 0.02562	38| 0.02555	38| 0.02552	38| 0.02554	38| 0.02562	38| 0.02576
Michigan	44| 0.02004	49| 0.01776	54| 0.0164	56| 0.01569	57| 0.01537	61| 0.01525	30| 0.02894	30| 0.02893	30| 0.02897	30| 0.02918	30| 0.02955	29| 0.03005
Minnesota	43| 0.02261	50| 0.01943	54| 0.01785	56| 0.01727	58| 0.0172	56| 0.01735	26| 0.03677	30| 0.03205	33| 0.02963	33| 0.02944	31| 0.03067	30| 0.0326
Mississippi	41| 0.02464	47| 0.02136	54| 0.01874	60| 0.01676	66| 0.01533	72| 0.01426	22| 0.04616	25| 0.04074	27| 0.03647	29| 0.03441	29| 0.03446	28| 0.03596
Missouri	40| 0.02636	45| 0.02312	50| 0.02077	55| 0.01908	59| 0.01785	65| 0.0169	28| 0.0371	30| 0.03444	32| 0.03252	33| 0.03187	32| 0.03244	31| 0.03391
Montana	42| 0.02435	46| 0.02202	50| 0.02051	52| 0.0195	54| 0.01878	60| 0.01823	30| 0.03434	31| 0.033	32| 0.03164	34| 0.03044	34| 0.02964	35| 0.02939
Nebraska	46| 0.01788	52| 0.01608	57| 0.01451	63| 0.01329	67| 0.01237	74| 0.01171	21| 0.03949	22| 0.03672	24| 0.03439	25| 0.03334	25| 0.03359	24| 0.03478
Nevada	46| 0.024	46| 0.02416	47| 0.02354	49| 0.02259	51| 0.0216	58| 0.02065	29| 0.0383	32| 0.03441	35| 0.03123	37| 0.02997	36| 0.03048	34| 0.03208
New Hampshire	42| 0.02539	45| 0.02361	48| 0.02212	51| 0.02097	53| 0.02011	57| 0.01947	32| 0.03317	33| 0.03163	34| 0.03065	35| 0.03019	35| 0.03006	35| 0.03012
New Jersey	42| 0.02687	45| 0.02499	49| 0.02297	54| 0.02118	58| 0.01974	64| 0.01858	29| 0.03895	30| 0.03772	31| 0.03638	32| 0.03536	33| 0.03479	33| 0.03467
New Mexico	41| 0.0239	48| 0.0204	54| 0.01793	60| 0.01623	65| 0.0151	71| 0.01432	19| 0.05039	23| 0.0423	27| 0.03619	30| 0.03257	31| 0.0313	31| 0.03173
New York	36| 0.0317	40| 0.02795	45| 0.02502	50| 0.02279	54| 0.02109	60| 0.01975	32| 0.03469	34| 0.03276	36| 0.03128	37| 0.03032	38| 0.02982	38| 0.02972
North Carolina	41| 0.02171	49| 0.0184	57| 0.01582	64| 0.01399	70| 0.01281	77| 0.01204	20| 0.04547	22| 0.03975	25| 0.03606	26| 0.03487	25| 0.03552	24| 0.03724
North Dakota	46| 0.02153	52| 0.01919	57| 0.01744	62| 0.01617	65| 0.01525	72| 0.01457	25| 0.03982	25| 0.04007	24| 0.04054	24| 0.04102	24| 0.04125	24| 0.0411
Ohio	46| 0.01938	52| 0.01726	57| 0.01578	61| 0.0148	64| 0.01416	68| 0.01374	24| 0.03668	25| 0.03558	26| 0.03487	26| 0.03465	26| 0.03487	25| 0.03545
Oklahoma	45| 0.02266	50| 0.02071	54| 0.01914	57| 0.01789	61| 0.01692	67| 0.01615	22| 0.04542	26| 0.03992	28| 0.03612	30| 0.03419	30| 0.03384	30| 0.0346
Oregon	41| 0.02509	47| 0.02213	52| 0.02015	55| 0.01897	57| 0.01833	61| 0.01799	28| 0.03667	31| 0.03382	32| 0.03216	33| 0.03174	32| 0.03229	31| 0.03349
Pennsylvania	41| 0.02471	44| 0.02281	47| 0.02127	50| 0.02009	52| 0.01923	58| 0.01858	34| 0.02912	35| 0.02877	35| 0.02853	35| 0.02859	35| 0.029	34| 0.02969
Rhode Island	45| 0.02726	49| 0.0254	51| 0.02409	53| 0.02317	55| 0.02254	59| 0.02208	29| 0.04205	31| 0.04015	32| 0.0389	32| 0.0384	32| 0.03846	32| 0.03888
South Carolina	45| 0.0205	50| 0.01837	55| 0.01681	58| 0.0158	60| 0.01521	65| 0.01488	26| 0.0356	27| 0.03414	28| 0.03231	29| 0.03138	29| 0.03156	28| 0.03252
South Dakota	43| 0.02593	48| 0.02346	52| 0.02149	56| 0.01998	60| 0.01885	66| 0.01797	28| 0.03939	28| 0.03923	29| 0.03859	30| 0.0377	30| 0.03707	30| 0.03707
Tennessee	41| 0.02624	47| 0.02305	52| 0.02053	58| 0.01861	62| 0.01722	68| 0.01618	22| 0.04953	25| 0.04313	29| 0.03648	33| 0.03275	33| 0.03292	30| 0.03562
Texas	46| 0.02289	52| 0.02035	56| 0.01885	59| 0.01803	60| 0.01761	65| 0.01741	19| 0.05522	25| 0.04289	29| 0.03584	31| 0.03387	30| 0.03516	28| 0.03789

Utah	40| 0.02277	46| 0.01992	50| 0.01806	54| 0.01681	57| 0.01594	63| 0.01528	26| 0.03532	30| 0.02991	34| 0.02647	35| 0.02562	34| 0.02694	31| 0.02952
Vermont	42| 0.02312	43| 0.02267	44| 0.02205	46| 0.02146	47| 0.02098	50| 0.02059	38| 0.0255	38| 0.02553	38| 0.0256	38| 0.02571	38| 0.02585	38| 0.02601
Virginia	44| 0.02017	50| 0.01801	54| 0.01666	56| 0.01586	58| 0.01537	62| 0.01507	25| 0.03582	29| 0.03076	32| 0.02813	32| 0.02803	30| 0.02957	28| 0.03184
Washington	37| 0.03056	41| 0.02758	45| 0.02516	49| 0.02335	52| 0.02208	56| 0.02116	34| 0.03366	35| 0.03228	37| 0.03111	38| 0.03032	38| 0.03001	38| 0.03021
West Virginia	48| 0.02047	52| 0.0191	54| 0.0184	55| 0.01813	57| 0.01809	54| 0.01816	30| 0.03293	30| 0.03326	30| 0.03327	30| 0.03296	30| 0.0326	30| 0.03252
Wisconsin	48| 0.01908	50| 0.01803	53| 0.01707	56| 0.01624	58| 0.01556	65| 0.01499	29| 0.03154	29| 0.03173	28| 0.0318	28| 0.03182	28| 0.03189	28| 0.03206
Wyoming	50| 0.02281	45| 0.02353	45| 0.02374	45| 0.02369	45| 0.02352	46| 0.02332	37| 0.02911	36| 0.02939	37| 0.02917	37| 0.02861	38| 0.02804	39| 0.02772
Argentina	68| 0.0065	59| 0.00711	55| 0.00761	53| 0.00799	51| 0.00827	50| 0.00848	30| 0.01395	33| 0.01264	31| 0.01365	26| 0.01593	23| 0.01831	21| 0.02029
Chile	52| 0.00711	56| 0.00667	59| 0.00636	61| 0.00615	62| 0.00601	65| 0.00591	28| 0.01332	26| 0.01429	25| 0.01509	23| 0.01583	22| 0.01675	21| 0.01796
Uruguay	60| 0.0069	53| 0.00759	49| 0.00815	47| 0.00859	45| 0.00891	44| 0.00915	35| 0.01155	34| 0.01165	34| 0.01184	33| 0.01202	33| 0.01212	33| 0.01215
Andorra	39| 0.01206	43| 0.01095	47| 0.01009	50| 0.00942	53| 0.00889	59| 0.00851	34| 0.01387	35| 0.01368	35| 0.01362	35| 0.01362	35| 0.01363	35| 0.01364
Austria	56| 0.00402	56| 0.00401	56| 0.00402	56| 0.004	56| 0.00397	59| 0.00393	26| 0.00859	27| 0.00841	27| 0.00828	27| 0.00823	27| 0.00828	27| 0.00841
Belgium	41| 0.00585	53| 0.00458	62| 0.00414	57| 0.00421	54| 0.00444	52| 0.00468	28| 0.00856	29| 0.0082	31| 0.00789	31| 0.00772	31| 0.00772	31| 0.00788
Cyprus	43| 0.01381	47| 0.01264	50| 0.01165	54| 0.01083	58| 0.01014	65| 0.00955	34| 0.01719	34| 0.0174	33| 0.0179	30| 0.01941	27| 0.02139	25| 0.02328
Denmark	45| 0.00954	48| 0.00909	51| 0.00846	55| 0.00783	60| 0.00726	67| 0.00678	31| 0.01389	30| 0.01423	29| 0.01464	29| 0.01508	28| 0.01553	27| 0.01594
Finland	29| 0.01326	34| 0.01132	40| 0.00969	46| 0.00841	53| 0.00743	64| 0.00668	33| 0.01166	37| 0.01049	40| 0.00978	41| 0.00945	42| 0.00936	41| 0.00942
France	49| 0.0095	46| 0.00963	45| 0.00988	44| 0.01008	43| 0.01021	43| 0.01029	45| 0.00978	43| 0.0104	39| 0.01142	36| 0.01235	34| 0.01306	33| 0.0136
Germany	77| 0.00356	72| 0.00366	65| 0.004	60| 0.00439	55| 0.00476	52| 0.00506	20| 0.01292	20| 0.01278	20| 0.01277	20| 0.0129	20| 0.01312	19| 0.01339
Greece	38| 0.01339	46| 0.01108	52| 0.00965	58| 0.00875	62| 0.00814	70| 0.00771	29| 0.01708	29| 0.01713	29| 0.01715	29| 0.01713	29| 0.01711	29| 0.01712
Iceland	35| 0.01139	36| 0.01104	38| 0.01062	39| 0.01019	41| 0.00978	43| 0.00941	43| 0.00937	44| 0.00917	44| 0.00905	45| 0.009	45| 0.00897	47| 0.00895
Ireland	42| 0.00978	50| 0.00823	57| 0.00724	61| 0.00666	65| 0.00634	67| 0.00618	26| 0.01583	26| 0.01596	26| 0.0159	26| 0.01567	27| 0.01534	27| 0.015
Israel	54| 0.00314	56| 0.00301	61| 0.00299	56| 0.003	56| 0.00303	55| 0.00306	26| 0.00644	27| 0.00613	28| 0.00595	28| 0.00598	27| 0.00617	26| 0.0065
Italy	55| 0.01077	57| 0.01046	60| 0.0104	57| 0.01046	57| 0.01053	57| 0.01058	25| 0.02348	26| 0.02321	26| 0.0229	26| 0.02263	27| 0.02242	27| 0.02226
Luxembourg	57| 0.00427	59| 0.00413	60| 0.00403	62| 0.00395	63| 0.00389	68| 0.00383	21| 0.01134	22| 0.01104	22| 0.01089	22| 0.01082	22| 0.01079	22| 0.01077
Malta	22| 0.02145	25| 0.01929	28| 0.0173	31| 0.01556	34| 0.01407	37| 0.01281	54| 0.00884	62| 0.00797	60| 0.00806	54| 0.0088	49| 0.00987	44| 0.01099
Monaco	47| 0.00162	51| 0.00149	55| 0.00138	59| 0.0013	61| 0.00126	66| 0.00124	26| 0.0029	27| 0.00279	27| 0.00277	27| 0.00278	27| 0.00281	27| 0.00284
Netherlands	47| 0.01018	52| 0.0093	57| 0.00846	62| 0.00776	67| 0.00722	75| 0.00679	24| 0.02003	24| 0.02045	23| 0.02072	23| 0.02088	23| 0.02098	23| 0.02104
Norway	72| 0.00501	66| 0.00517	64| 0.00531	64| 0.00533	66| 0.00521	67| 0.00512	18| 0.0192	17| 0.01947	17| 0.01982	17| 0.02024	16| 0.02069	16| 0.02119
Portugal	37| 0.01331	48| 0.01022	55| 0.0088	59| 0.00829	62| 0.00816	59| 0.0082	34| 0.01413	32| 0.01524	30| 0.01622	29| 0.01696	28| 0.01749	27| 0.01785

San Marino	60| 0.00183	69| 0.0016	75| 0.00146	79| 0.00138	82| 0.00134	86| 0.00132	7| 0.01431	8| 0.0131	9| 0.01233	9| 0.01226	8| 0.0129	8| 0.01406
Spain	49| 0.00991	53| 0.00914	58| 0.00882	55| 0.00894	52| 0.00934	50| 0.00972	33| 0.0148	32| 0.01539	30| 0.016	30| 0.01641	29| 0.01669	29| 0.01692
Sweden	52| 0.0077	55| 0.00734	56| 0.00721	59| 0.00719	56| 0.00723	55| 0.00728	29| 0.01387	30| 0.0133	30| 0.01349	28| 0.01436	26| 0.01562	24| 0.01699
Switzerland	48| 0.00679	49| 0.00664	51| 0.00638	53| 0.00613	55| 0.00593	60| 0.00588	27| 0.01213	29| 0.0113	31| 0.01057	32| 0.01009	33| 0.00994	32| 0.01007
United Kingdom	40| 0.00744	45| 0.00666	50| 0.00599	55| 0.00545	60| 0.00501	68| 0.00465	33| 0.00894	33| 0.00916	31| 0.00955	30| 0.01002	28| 0.01049	27| 0.0109
Bolivia (Plurinational State of)	50| 0.00342	54| 0.00317	56| 0.00303	58| 0.00296	58| 0.00293	62| 0.00291	41| 0.00419	38| 0.00447	27| 0.00638	21| 0.00803	18| 0.00923	17| 0.01009
Ecuador	63| 0.00998	59| 0.01006	59| 0.01018	58| 0.01028	57| 0.01037	57| 0.01043	27| 0.02171	26| 0.02308	24| 0.0243	24| 0.02514	23| 0.02563	23| 0.02588
Peru	37| 0.02136	37| 0.02128	37| 0.02123	37| 0.02121	37| 0.02121	37| 0.02123	42| 0.01881	45| 0.01723	47| 0.01658	49| 0.0164	48| 0.01646	47| 0.01667
Antigua and Barbuda	42| 0.00337	54| 0.00262	67| 0.00211	81| 0.00176	94| 0.00151	110| 0.00133	10| 0.01389	9| 0.01523	9| 0.01599	8| 0.01644	8| 0.01672	8| 0.01691
Bahamas	63| 0.0018	73| 0.00155	78| 0.00146	79| 0.00143	80| 0.00142	85| 0.0014	8| 0.01462	8| 0.01374	8| 0.01368	7| 0.01549	6| 0.01798	5| 0.01997
Barbados	87| 0.0029	76| 0.00323	65| 0.00376	57| 0.00426	53| 0.00461	51| 0.00483	23| 0.01065	21| 0.0113	19| 0.01272	17| 0.01411	16| 0.01518	15| 0.0159
Belize	29| 0.01128	33| 0.00991	38| 0.0087	43| 0.0077	48| 0.00689	54| 0.00625	47| 0.00704	45| 0.0073	43| 0.00764	43| 0.00771	41| 0.00809	36| 0.00902
Bermuda	51| 0.00438	55| 0.00434	51| 0.00436	51| 0.00439	50| 0.00444	50| 0.00448	32| 0.00695	33| 0.00677	33| 0.00686	32| 0.00699	31| 0.00714	31| 0.00729
Cuba	74| 0.00373	70| 0.00376	69| 0.00381	68| 0.00386	67| 0.00391	67| 0.00394	17| 0.01548	15| 0.01702	14| 0.01856	13| 0.0196	13| 0.02013	13| 0.02025
Dominica	71| 0.00249	74| 0.00241	75| 0.00236	78| 0.00235	75| 0.00235	75| 0.00237	8| 0.02279	8| 0.02096	9| 0.0193	9| 0.01836	9| 0.01848	9| 0.01955
Dominican Republic	57| 0.0079	52| 0.00795	52| 0.00795	52| 0.00794	52| 0.00794	52| 0.00796	27| 0.01551	31| 0.01354	32| 0.01283	32| 0.01284	31| 0.01317	30| 0.01364
Grenada	90| 0.00206	78| 0.00235	71| 0.00259	66| 0.00277	63| 0.00291	61| 0.00302	6| 0.0275	9| 0.02062	11| 0.01638	13| 0.01378	15| 0.01209	17| 0.01092
Guyana	58| 0.0025	62| 0.00234	64| 0.00229	65| 0.00226	65| 0.00224	68| 0.00223	8| 0.01822	13| 0.01089	21| 0.00687	26| 0.00562	26| 0.00568	24| 0.00616
Haiti	45| 0.00466	59| 0.00352	66| 0.00334	59| 0.00355	54| 0.00385	50| 0.00416	27| 0.00759	28| 0.00755	28| 0.00747	28| 0.0074	28| 0.00738	28| 0.00742
Jamaica	70| 0.00664	60| 0.00741	55| 0.00804	52| 0.00842	51| 0.0086	51| 0.00865	21| 0.02071	26| 0.01719	28| 0.01561	29| 0.01517	29| 0.0153	28| 0.01569
Puerto Rico	39| 0.01084	43| 0.00987	47| 0.00906	51| 0.00839	55| 0.00786	62| 0.00742	34| 0.01254	38| 0.01136	37| 0.01151	35| 0.01237	31| 0.01362	28| 0.01502
Saint Kitts and Nevis	75| 0.00202	69| 0.0022	69| 0.00218	73| 0.00208	78| 0.00195	85| 0.00182	10| 0.01475	9| 0.01671	8| 0.01804	8| 0.01888	8| 0.01937	8| 0.01962
Saint Lucia	91| 0.00259	76| 0.00299	69| 0.00332	64| 0.00357	60| 0.00377	58| 0.00393	9| 0.02538	12| 0.01912	14| 0.0162	15| 0.01491	16| 0.01426	16| 0.01386
Saint Vincent and the Grenadines	40| 0.00483	47| 0.00405	55| 0.00348	63| 0.00306	70| 0.00274	79| 0.00248	10| 0.01878	15| 0.01288	21| 0.0092	27| 0.00697	34| 0.00564	39| 0.00486
Suriname	53| 0.00362	57| 0.00337	61| 0.00312	66| 0.00287	72| 0.00265	80| 0.00246	13| 0.01498	16| 0.0116	19| 0.0099	21| 0.00916	21| 0.00897	21| 0.00911
Trinidad and Tobago	72| 0.00657	64| 0.00711	61| 0.00743	60| 0.00758	59| 0.00764	59| 0.00763	23| 0.0199	21| 0.02156	20| 0.02215	20| 0.02217	20| 0.02201	21| 0.02188
United States Virgin Islands	68| 0.00527	66| 0.00528	65| 0.00533	64| 0.00538	63| 0.00545	63| 0.00551	20| 0.01733	20| 0.01756	19| 0.01815	18| 0.01889	17| 0.01969	17| 0.02052
Colombia	60| 0.00565	67| 0.00538	59| 0.0058	54| 0.00634	50| 0.00682	47| 0.00721	28| 0.01216	31| 0.01104	28| 0.01203	26| 0.01295	25| 0.01347	25| 0.01374
Costa Rica	46| 0.00367	53| 0.00321	58| 0.0029	63| 0.0027	66| 0.00258	70| 0.0025	13| 0.01316	19| 0.00896	24| 0.00689	28| 0.00598	30| 0.00568	30| 0.0057

El Salvador	47| 0.00307	52| 0.00276	58| 0.00249	63| 0.0023	66| 0.0022	69| 0.00216	17| 0.00831	24| 0.00592	26| 0.00553	26| 0.00552	26| 0.00546	26| 0.0056
Guatemala	28| 0.0081	36| 0.00635	46| 0.00505	56| 0.00411	67| 0.00344	82| 0.00297	30| 0.0077	33| 0.00691	33| 0.00707	31| 0.00745	30| 0.00781	28| 0.00815
Honduras	45| 0.00626	47| 0.00599	48| 0.00587	48| 0.00588	47| 0.00597	46| 0.0061	18| 0.01569	24| 0.01183	32| 0.0089	41| 0.00691	49| 0.0058	55| 0.0054
Mexico	54| 0.00812	53| 0.00832	53| 0.00825	54| 0.00804	56| 0.00777	62| 0.00749	21| 0.02111	26| 0.01698	29| 0.01485	31| 0.01395	31| 0.01388	30| 0.01444
Nicaragua	63| 0.00138	66| 0.00137	63| 0.00138	62| 0.00139	62| 0.0014	62| 0.0014	16| 0.00524	21| 0.00414	23| 0.00377	23| 0.00373	21| 0.00401	18| 0.00474
Panama	68| 0.00316	71| 0.00306	72| 0.00301	71| 0.00301	72| 0.00301	71| 0.00303	15| 0.01465	14| 0.01568	13| 0.01695	12| 0.0184	11| 0.02006	10| 0.02191
Venezuela (Bolivarian Republic of)	61| 0.00461	71| 0.004	80| 0.00355	88| 0.00321	96| 0.00296	104| 0.00277	0| Inf	0| Inf	0| Inf	0| Inf	0| Inf	0| Inf
Brazil	49| 0.0163	49| 0.01647	49| 0.01636	50| 0.01612	51| 0.01584	56| 0.01555	29| 0.02764	32| 0.0249	34| 0.02378	34| 0.02353	34| 0.02374	33| 0.02415
Acre	62| 0.00934	57| 0.00959	57| 0.00971	57| 0.00974	57| 0.00972	57| 0.00967	24| 0.02259	27| 0.02051	27| 0.02027	26| 0.02084	25| 0.02165	24| 0.02242
Alagoas	45| 0.0157	45| 0.01551	46| 0.01522	47| 0.0149	48| 0.01459	52| 0.01431	36| 0.01954	38| 0.0183	38| 0.01846	36| 0.01917	35| 0.01995	34| 0.02064
Amapá	43| 0.01708	44| 0.01688	45| 0.01647	46| 0.01602	48| 0.01558	52| 0.01518	32| 0.02306	35| 0.02105	37| 0.01986	39| 0.01917	39| 0.01879	40| 0.0186
Amazonas	46| 0.01868	46| 0.01883	46| 0.01879	46| 0.01867	47| 0.01853	52| 0.01838	38| 0.02255	38| 0.02252	37| 0.0232	36| 0.02414	35| 0.02505	33| 0.02584
Bahia	50| 0.01406	50| 0.01426	50| 0.01426	50| 0.01414	51| 0.01396	53| 0.01375	34| 0.02104	35| 0.02026	34| 0.02103	32| 0.02213	31| 0.02311	30| 0.02388
Ceará	51| 0.01076	51| 0.01071	52| 0.01055	53| 0.01035	54| 0.01014	56| 0.00995	27| 0.01977	31| 0.01764	32| 0.01709	32| 0.01725	31| 0.01768	30| 0.01817
Distrito Federal	65| 0.01664	56| 0.01788	54| 0.01858	53| 0.01896	53| 0.01916	52| 0.01926	27| 0.0374	27| 0.03737	27| 0.0368	28| 0.03593	29| 0.035	29| 0.03418
Espírito Santo	49| 0.01821	49| 0.01819	49| 0.01807	50| 0.01794	50| 0.01781	55| 0.01771	29| 0.03035	32| 0.02746	34| 0.02617	35| 0.02578	34| 0.02585	34| 0.02615
Goiás	48| 0.01983	48| 0.01961	49| 0.01929	50| 0.01895	51| 0.01863	54| 0.01833	30| 0.0314	32| 0.0292	34| 0.02797	34| 0.02731	35| 0.02698	35| 0.02683
Maranhão	49| 0.01357	49| 0.01364	49| 0.01357	50| 0.01344	50| 0.01328	56| 0.01312	35| 0.01897	35| 0.01915	34| 0.01977	32| 0.02061	31| 0.0215	30| 0.02235
Mato Grosso	57| 0.01341	56| 0.01364	56| 0.01367	56| 0.01359	57| 0.01346	59| 0.01332	25| 0.03015	27| 0.02852	27| 0.02797	27| 0.02801	27| 0.02833	26| 0.02877
Mato Grosso do Sul	50| 0.01312	51| 0.01292	52| 0.01255	54| 0.01214	56| 0.01175	61| 0.01138	27| 0.02424	29| 0.02255	30| 0.02179	30| 0.02153	30| 0.02152	30| 0.02164
Minas Gerais	50| 0.01651	50| 0.01678	50| 0.01672	51| 0.01649	51| 0.0162	55| 0.01589	30| 0.02774	32| 0.02596	33| 0.02528	33| 0.02521	33| 0.02543	32| 0.02579
Pará	44| 0.02011	45| 0.01979	46| 0.01937	47| 0.01896	48| 0.0186	50| 0.01828	37| 0.02408	39| 0.02295	38| 0.02327	37| 0.02413	35| 0.02508	34| 0.02593
Paraíba	47| 0.01499	47| 0.01508	47| 0.01508	47| 0.01503	48| 0.01497	51| 0.0149	34| 0.02116	37| 0.01928	37| 0.01908	36| 0.01962	35| 0.02037	34| 0.02108
Paraná	60| 0.01287	55| 0.01334	54| 0.01348	54| 0.01345	55| 0.01333	56| 0.01318	25| 0.02889	27| 0.02666	28| 0.02572	29| 0.02547	29| 0.02553	28| 0.02574
Pernambuco	51| 0.01661	46| 0.01689	46| 0.01697	46| 0.01695	46| 0.01689	47| 0.0168	35| 0.0224	38| 0.02051	38| 0.02051	37| 0.0212	36| 0.02199	34| 0.02272
Piaui	46| 0.01524	47| 0.01515	47| 0.01499	48| 0.0148	48| 0.0146	53| 0.01441	36| 0.01945	37| 0.01919	36| 0.01946	35| 0.02002	34| 0.0207	33| 0.0214
Rio de Janeiro	45| 0.02208	44| 0.02223	45| 0.02207	45| 0.02177	46| 0.02141	51| 0.02105	37| 0.02661	37| 0.02649	37| 0.02644	37| 0.02634	38| 0.02616	38| 0.02593
Rio Grande do Norte	51| 0.01438	50| 0.01465	50| 0.01469	50| 0.01462	50| 0.01448	53| 0.01432	34| 0.02148	35| 0.02083	34| 0.02164	32| 0.0227	31| 0.02361	30| 0.02431
Rio Grande do Sul	47| 0.02002	47| 0.02	48| 0.01971	49| 0.0193	50| 0.01885	53| 0.01842	34| 0.02751	35| 0.02713	35| 0.02718	34| 0.02735	34| 0.0275	34| 0.02758

Rondônia	59| 0.01292	54| 0.01321	53| 0.01334	53| 0.01338	53| 0.01335	54| 0.0133	30| 0.02359	30| 0.02338	30| 0.02393	29| 0.02469	28| 0.02543	27| 0.0261
Roraima	54| 0.01504	51| 0.01547	51| 0.0156	51| 0.01558	51| 0.01548	52| 0.01535	31| 0.02544	31| 0.02512	32| 0.02503	32| 0.02502	32| 0.02504	32| 0.02506
Santa Catarina	48| 0.0189	49| 0.0188	49| 0.01853	50| 0.0182	51| 0.01787	56| 0.01757	32| 0.02857	33| 0.02766	33| 0.02741	33| 0.02743	33| 0.02752	33| 0.02759
São Paulo	47| 0.02051	46| 0.02087	46| 0.02085	47| 0.02064	48| 0.02035	53| 0.02004	34| 0.0284	35| 0.02772	35| 0.02727	36| 0.02691	36| 0.02657	37| 0.02626
Sergipe	53| 0.01498	49| 0.01513	48| 0.01519	48| 0.0152	48| 0.01518	48| 0.01516	35| 0.02083	36| 0.02048	35| 0.02086	34| 0.02144	33| 0.022	33| 0.02248
Tocantins	55| 0.01344	51| 0.01366	51| 0.01372	51| 0.01368	51| 0.01358	52| 0.01346	29| 0.02393	33| 0.02089	34| 0.02067	32| 0.02141	31| 0.02223	30| 0.02286
Paraguay	30| 0.01015	36| 0.00843	41| 0.00733	46| 0.00666	49| 0.00621	52| 0.0059	15| 0.01948	26| 0.01184	39| 0.00776	50| 0.00607	56| 0.00548	60| 0.00528
Afghanistan	32| 0.02293	35| 0.02129	37| 0.02004	39| 0.01907	41| 0.01831	42| 0.01767	42| 0.01779	46| 0.0161	50| 0.01571	47| 0.01596	45| 0.01639	44| 0.01681
Algeria	37| 0.02077	45| 0.01733	52| 0.01481	60| 0.01305	66| 0.01173	76| 0.01079	17| 0.04484	22| 0.03549	26| 0.0293	30| 0.02541	34| 0.02311	35| 0.02191
Bahrain	51| 0.01225	56| 0.01132	59| 0.01063	62| 0.01013	65| 0.00974	71| 0.00944	22| 0.02841	22| 0.02844	22| 0.02813	23| 0.02752	23| 0.02675	24| 0.026
Egypt	70| 0.00825	72| 0.008	74| 0.00785	74| 0.00776	75| 0.0077	77| 0.00766	10| 0.05901	10| 0.05839	10| 0.05809	10| 0.05842	9| 0.05989	9| 0.06386
Iran (Islamic Republic of)	60| 0.0199	55| 0.02049	54| 0.02103	53| 0.02148	52| 0.02181	51| 0.02205	23| 0.04889	27| 0.0418	30| 0.03806	31| 0.03618	32| 0.03524	32| 0.03473
Iraq	41| 0.01011	49| 0.00858	56| 0.00752	61| 0.00684	65| 0.0064	72| 0.00612	25| 0.0167	29| 0.01422	29| 0.01442	26| 0.01573	24| 0.01713	23| 0.01829
Jordan	65| 0.00982	58| 0.01039	56| 0.01086	54| 0.01122	53| 0.0115	52| 0.01171	30| 0.01984	30| 0.02036	28| 0.02153	26| 0.02296	25| 0.0244	23| 0.0257
Kuwait	56| 0.0084	55| 0.00854	55| 0.00853	55| 0.00846	56| 0.00835	59| 0.00823	29| 0.01609	28| 0.01678	27| 0.017	27| 0.01706	27| 0.01736	26| 0.01791
Lebanon	50| 0.01796	52| 0.01732	54| 0.01682	55| 0.01645	56| 0.01616	60| 0.01594	29| 0.03132	30| 0.03012	30| 0.03028	29| 0.03102	28| 0.03196	27| 0.03292
Libya	63| 0.00845	66| 0.00814	68| 0.00784	71| 0.00758	73| 0.00732	78| 0.00709	12| 0.04516	13| 0.04103	14| 0.0387	14| 0.03727	14| 0.03651	14| 0.03642
Morocco	40| 0.01621	43| 0.015	46| 0.01408	48| 0.01336	50| 0.0128	57| 0.01234	27| 0.02345	31| 0.02044	35| 0.01823	39| 0.01669	41| 0.01565	43| 0.01495
Oman	58| 0.01294	65| 0.01143	71| 0.01059	73| 0.01029	75| 0.00999	78| 0.00986	10| 0.0732	12| 0.06348	13| 0.05674	14| 0.05203	15| 0.04897	16| 0.04755
Palestine	45| 0.00811	53| 0.00679	62| 0.00587	68| 0.0053	73| 0.00497	76| 0.0048	19| 0.01903	20| 0.01764	21| 0.01739	21| 0.01746	21| 0.01749	21| 0.0174
Qatar	53| 0.00981	56| 0.00927	59| 0.00881	62| 0.00843	64| 0.00813	71| 0.00787	26| 0.0199	23| 0.02211	23| 0.02253	23| 0.02291	21| 0.02426	19| 0.02668
Saudi Arabia	65| 0.00829	69| 0.00785	72| 0.00784	67| 0.00801	65| 0.00822	64| 0.00843	16| 0.03288	17| 0.03211	17| 0.03225	16| 0.03243	16| 0.0325	16| 0.03252
Sudan	23| 0.02188	25| 0.02014	27| 0.01874	28| 0.01762	30| 0.01671	31| 0.01597	38| 0.01332	51| 0.0099	62| 0.00814	66| 0.00778	62| 0.00814	57| 0.00873
Syrian Arab Republic	56| 0.01258	60| 0.0116	64| 0.01096	66| 0.01065	68| 0.01035	72| 0.01018	19| 0.03585	19| 0.03593	19| 0.03619	19| 0.0365	19| 0.0368	19| 0.03705
Tunisia	44| 0.01347	51| 0.01177	57| 0.01044	63| 0.00944	69| 0.00864	77| 0.008	14| 0.04118	19| 0.03179	22| 0.02633	26| 0.0232	28| 0.0213	30| 0.02009
Turkey	55| 0.0144	59| 0.01429	55| 0.01435	55| 0.01448	54| 0.01463	54| 0.01477	26| 0.03094	28| 0.02791	29| 0.02732	29| 0.02746	28| 0.0278	28| 0.02819
United Arab Emirates	65| 0.0074	69| 0.007	72| 0.00676	72| 0.00672	74| 0.00657	79| 0.00643	12| 0.04074	12| 0.03913	12| 0.0386	12| 0.04006	11| 0.04266	10| 0.04555
Yemen	35| 0.02865	39| 0.02617	42| 0.02417	45| 0.02258	48| 0.02122	54| 0.02011	39| 0.02613	39| 0.0261	39| 0.02595	40| 0.02573	40| 0.02549	40| 0.02527
Bangladesh	58| 0.01351	62| 0.01263	64| 0.01259	61| 0.01284	59| 0.0131	58| 0.01334	25| 0.03112	24| 0.03279	23| 0.03408	22| 0.03476	22| 0.03492	22| 0.03472

Bhutan	39| 0.01201	40| 0.01149	42| 0.01113	43| 0.01083	44| 0.01054	45| 0.01027	32| 0.01444	36| 0.01281	40| 0.01157	43| 0.01071	46| 0.0102	50| 0.01002
India	69| 0.00869	63| 0.00898	63| 0.00907	63| 0.00907	63| 0.00901	64| 0.00893	15| 0.03782	17| 0.03385	18| 0.03057	20| 0.02798	22| 0.02594	23| 0.02433
Andhra Pradesh	67| 0.01297	56| 0.01472	52| 0.01584	50| 0.01652	49| 0.01701	48| 0.01731	24| 0.03449	26| 0.03118	29| 0.02849	31| 0.02637	33| 0.02469	35| 0.02335
Arunachal Pradesh	61| 0.00785	57| 0.00817	56| 0.00836	55| 0.00845	55| 0.00848	55| 0.00846	22| 0.0212	24| 0.01936	26| 0.01781	28| 0.01657	30| 0.01559	31| 0.01481
Assam	74| 0.01059	67| 0.01142	64| 0.01193	63| 0.0122	62| 0.01231	62| 0.01233	15| 0.05095	16| 0.04655	18| 0.04316	19| 0.04065	20| 0.03882	20| 0.03747
Bihar	61| 0.00499	62| 0.00486	64| 0.00475	65| 0.00466	66| 0.00457	70| 0.0045	13| 0.02261	16| 0.01891	18| 0.01645	20| 0.01485	22| 0.01381	23| 0.01311
Chhattisgarh	58| 0.00674	60| 0.00649	62| 0.00629	64| 0.00613	65| 0.006	69| 0.00591	14| 0.02694	17| 0.02317	19| 0.02019	22| 0.01791	24| 0.01617	26| 0.01485
Delhi	61| 0.01059	53| 0.0116	49| 0.0125	46| 0.01321	44| 0.01377	43| 0.01419	29| 0.02122	35| 0.01755	36| 0.01671	36| 0.0169	35| 0.01747	33| 0.01822
Goa	41| 0.01541	41| 0.0153	41| 0.01513	42| 0.01494	43| 0.01476	43| 0.01459	34| 0.01836	40| 0.01586	42| 0.01496	43| 0.01456	44| 0.01435	46| 0.01427
Gujarat	61| 0.00684	65| 0.00677	62| 0.00678	61| 0.00681	61| 0.00683	61| 0.00685	21| 0.01961	21| 0.01958	21| 0.01946	22| 0.01926	22| 0.01899	22| 0.01869
Haryana	66| 0.01146	61| 0.01198	58| 0.01241	57| 0.01272	56| 0.01293	55| 0.01307	23| 0.03086	24| 0.0298	25| 0.0294	25| 0.02928	25| 0.02921	25| 0.02912
Himachal Pradesh	48| 0.01041	49| 0.01026	50| 0.01003	52| 0.00977	53| 0.00952	57| 0.00929	37| 0.01361	36| 0.01395	33| 0.01529	30| 0.01672	28| 0.01794	27| 0.01891
Jammu and Kashmir	48| 0.01594	48| 0.01594	48| 0.01571	49| 0.01536	51| 0.01503	53| 0.01468	33| 0.02323	34| 0.02244	34| 0.02233	34| 0.02242	34| 0.02252	34| 0.02256
Jharkhand	55| 0.00691	55| 0.0069	55| 0.00685	56| 0.00679	56| 0.00673	59| 0.00667	20| 0.01927	23| 0.01663	26| 0.01461	29| 0.01311	32| 0.012	34| 0.01118
Karnataka	63| 0.00619	63| 0.00614	64| 0.00609	64| 0.00606	65| 0.00603	68| 0.00602	15| 0.02487	17| 0.02269	18| 0.02092	20| 0.01955	21| 0.01851	22| 0.0177
Kerala	40| 0.01611	42| 0.01514	45| 0.01439	47| 0.01383	48| 0.01341	51| 0.01307	44| 0.01469	42| 0.01529	39| 0.01663	36| 0.01799	34| 0.01911	32| 0.01999
Madhya Pradesh	66| 0.00562	68| 0.00545	70| 0.00533	71| 0.00526	71| 0.00523	75| 0.00523	10| 0.03636	11| 0.03243	13| 0.02918	14| 0.02657	15| 0.02449	16| 0.02282
Maharashtra	54| 0.01374	54| 0.01365	55| 0.01345	56| 0.01322	57| 0.01298	62| 0.01276	29| 0.0254	28| 0.02678	27| 0.02778	26| 0.02838	26| 0.02867	26| 0.02874
Manipur	49| 0.00786	52| 0.00748	53| 0.00738	53| 0.00735	53| 0.00733	55| 0.00731	33| 0.01186	32| 0.01214	31| 0.01252	30| 0.01286	30| 0.01312	29| 0.01331
Meghalaya	48| 0.00701	48| 0.00702	48| 0.00693	49| 0.00681	50| 0.00666	56| 0.0065	28| 0.01188	31| 0.01069	34| 0.00997	35| 0.00952	36| 0.00921	37| 0.00896
Mizoram	52| 0.0093	53| 0.00901	55| 0.00868	57| 0.00835	60| 0.00805	64| 0.00777	31| 0.01549	29| 0.01661	27| 0.01791	25| 0.01904	24| 0.0199	23| 0.02052
Nagaland	53| 0.00608	47| 0.00633	46| 0.00646	45| 0.00651	45| 0.00653	45| 0.00652	34| 0.00867	37| 0.00792	38| 0.00783	37| 0.00792	37| 0.00804	36| 0.00817
Odisha	54| 0.00695	55| 0.00692	58| 0.0069	55| 0.0069	55| 0.00692	54| 0.00695	20| 0.01904	23| 0.0162	27| 0.01405	30| 0.01249	33| 0.01135	36| 0.01052
Punjab	47| 0.02666	50| 0.02523	52| 0.0242	53| 0.02354	54| 0.02313	58| 0.02288	34| 0.03656	33| 0.03832	31| 0.04009	30| 0.04155	29| 0.0427	29| 0.04358
Rajasthan	67| 0.01009	63| 0.01025	63| 0.01031	63| 0.0103	63| 0.01024	64| 0.01015	15| 0.04367	16| 0.03896	18| 0.03485	20| 0.03139	23| 0.02852	25| 0.02615
Sikkim	53| 0.02272	48| 0.02368	47| 0.02418	47| 0.02437	46| 0.02441	47| 0.02435	36| 0.0317	36| 0.03168	35| 0.03196	35| 0.03217	35| 0.03222	35| 0.03212
Tamil Nadu	47| 0.02041	47| 0.02032	47| 0.02017	48| 0.02001	48| 0.01987	53| 0.01976	39| 0.02464	37| 0.02565	35| 0.02695	34| 0.02809	33| 0.02897	32| 0.0296
Telangana	69| 0.01233	59| 0.01395	55| 0.01498	52| 0.01561	51| 0.01606	50| 0.01632	22| 0.03617	25| 0.03321	27| 0.03074	28| 0.02875	30| 0.02716	32| 0.02587
Tripura	47| 0.00541	50| 0.00518	52| 0.00496	54| 0.00477	56| 0.0046	61| 0.00446	26| 0.00968	29| 0.00887	30| 0.00842	31| 0.00816	32| 0.00803	32| 0.00803

Union Territories other than Delhi	53| 0.00768	49| 0.00797	48| 0.00811	47| 0.00816	47| 0.00815	48| 0.00811	23| 0.01647	32| 0.01222	37| 0.01049	39| 0.00999	39| 0.00994	38| 0.01008
Uttar Pradesh	64| 0.00794	64| 0.00794	64| 0.00789	65| 0.00779	66| 0.00767	69| 0.00754	13| 0.03852	15| 0.03418	17| 0.03035	19| 0.0271	21| 0.02441	23| 0.02222
Uttarakhand	66| 0.00905	57| 0.00978	54| 0.01029	52| 0.01063	51| 0.01086	50| 0.01101	27| 0.02014	29| 0.01889	29| 0.01885	29| 0.01924	28| 0.01968	28| 0.02003
West Bengal	62| 0.00739	59| 0.00764	58| 0.00778	58| 0.00783	58| 0.00783	58| 0.0078	19| 0.02414	21| 0.02124	24| 0.01906	26| 0.01746	28| 0.01629	29| 0.01539
Nepal	35| 0.01127	39| 0.01006	40| 0.00976	39| 0.00999	37| 0.01045	36| 0.01096	40| 0.0098	44| 0.009	46| 0.00849	50| 0.00827	47| 0.00827	47| 0.00842
Pakistan	71| 0.013	53| 0.01672	46| 0.01933	43| 0.02088	41| 0.02195	39| 0.02263	33| 0.02686	35| 0.02561	35| 0.02518	35| 0.02529	35| 0.02568	34| 0.02616
China	61| 0.0055	64| 0.00521	66| 0.00507	67| 0.005	67| 0.00496	70| 0.00496	13| 0.02629	16| 0.02128	18| 0.01872	19| 0.01747	20| 0.01699	19| 0.01702
Anhui	64| 0.00481	61| 0.00499	58| 0.00524	56| 0.00546	54| 0.00564	53| 0.00579	18| 0.0171	22| 0.014	25| 0.01201	28| 0.01082	30| 0.01016	31| 0.00991
Beijing	63| 0.00685	63| 0.00684	64| 0.00683	64| 0.00681	64| 0.00678	66| 0.00676	22| 0.0198	20| 0.02192	19| 0.02248	19| 0.0228	18| 0.02346	18| 0.02448
Chongqing	49| 0.00563	56| 0.00489	61| 0.00454	63| 0.00438	64| 0.00431	66| 0.00429	15| 0.01761	20| 0.01348	24| 0.01132	27| 0.01026	28| 0.00995	27| 0.01022
Fujian	55| 0.0079	59| 0.00743	61| 0.00721	62| 0.00706	63| 0.00693	66| 0.00683	16| 0.02704	20| 0.02194	23| 0.01923	24| 0.01782	25| 0.01712	26| 0.01686
Gansu	52| 0.00701	55| 0.00699	53| 0.00699	53| 0.007	53| 0.00701	52| 0.00701	21| 0.01753	26| 0.01412	30| 0.01205	34| 0.01094	35| 0.01043	36| 0.01026
Guangdong	52| 0.01029	58| 0.00922	62| 0.00857	65| 0.00814	68| 0.00782	73| 0.00757	18| 0.0291	20| 0.0266	21| 0.02572	21| 0.02541	21| 0.02527	21| 0.02518
Guangxi	38| 0.01404	42| 0.01278	46| 0.01172	50| 0.01086	53| 0.01014	60| 0.00955	26| 0.02033	31| 0.01732	35| 0.01532	38| 0.0141	40| 0.01344	41| 0.01318
Guizhou	41| 0.00661	49| 0.00551	56| 0.00481	62| 0.00436	66| 0.00407	72| 0.00387	18| 0.01471	21| 0.01247	25| 0.01078	28| 0.0096	30| 0.00883	32| 0.00837
Hainan	40| 0.01295	43| 0.01202	46| 0.01118	49| 0.01046	53| 0.00983	58| 0.00928	25| 0.02057	31| 0.01684	35| 0.0146	39| 0.01338	40| 0.01282	41| 0.01271
Hebei	90| 0.00548	71| 0.00656	61| 0.00763	55| 0.00847	51| 0.0091	49| 0.00957	15| 0.02988	19| 0.02456	21| 0.02212	22| 0.02094	23| 0.02037	23| 0.0202
Heilongjiang	56| 0.00877	59| 0.00842	60| 0.00819	62| 0.00802	63| 0.00791	65| 0.00782	17| 0.02864	21| 0.0236	23| 0.02136	24| 0.02037	25| 0.01999	25| 0.01998
Henan	64| 0.00489	66| 0.00474	67| 0.00464	69| 0.00457	69| 0.00451	73| 0.00447	12| 0.02592	14| 0.02185	16| 0.01962	17| 0.0186	17| 0.01845	16| 0.01895
Hong Kong Special Administrative Region of China	56| 0.01333	47| 0.01491	45| 0.01545	45| 0.01537	47| 0.01495	48| 0.01436	37| 0.01878	35| 0.01981	35| 0.01994	35| 0.0198	35| 0.0197	35| 0.01968
Hubei	58| 0.00554	62| 0.0052	65| 0.00494	68| 0.00475	70| 0.00461	72| 0.00452	13| 0.02438	16| 0.02014	18| 0.01777	19| 0.0166	20| 0.01629	19| 0.0166
Hunan	42| 0.01099	49| 0.00949	55| 0.00838	61| 0.00762	65| 0.00712	73| 0.00679	19| 0.0239	23| 0.02025	26| 0.01788	28| 0.01646	29| 0.01569	30| 0.0154
Inner Mongolia	73| 0.0069	68| 0.00716	66| 0.00744	63| 0.00772	61| 0.00797	60| 0.00819	16| 0.03012	18| 0.02709	19| 0.02595	19| 0.02572	19| 0.02606	18| 0.02678
Jiangsu	61| 0.00522	67| 0.00476	72| 0.00445	75| 0.00428	77| 0.00416	82| 0.00409	9| 0.03299	11| 0.02877	11| 0.0274	12| 0.02712	12| 0.0273	11| 0.02771
Jiangxi	57| 0.00609	61| 0.00585	59| 0.00585	59| 0.0059	58| 0.00596	58| 0.00601	18| 0.01873	22| 0.01591	24| 0.01403	27| 0.01287	28| 0.01223	29| 0.01199
Jilin	74| 0.00495	77| 0.00489	71| 0.00517	66| 0.0055	63| 0.00579	60| 0.00602	12| 0.02895	15| 0.02455	16| 0.02312	16| 0.0229	15| 0.02329	15| 0.02406
Liaoning	61| 0.00702	76| 0.00586	74| 0.00586	69| 0.00627	64| 0.00677	59| 0.00726	14| 0.02974	16| 0.02691	16| 0.02593	17| 0.02562	17| 0.02558	17| 0.02563
Macao Special Administrative Region of China	48| 0.00495	53| 0.00445	58| 0.00409	62| 0.00383	65| 0.00364	69| 0.00352	23| 0.01036	23| 0.01016	24| 0.0097	25| 0.00941	25| 0.00931	25| 0.00928
Ningxia	54| 0.00795	56| 0.00759	58| 0.00734	60| 0.00715	61| 0.00701	64| 0.0069	18| 0.02333	22| 0.01959	25| 0.01713	27| 0.01578	28| 0.01534	27| 0.01559

Qinghai	52| 0.00464	63| 0.00388	69| 0.00366	66| 0.0037	64| 0.00382	61| 0.00397	14| 0.01674	18| 0.01353	21| 0.01152	23| 0.01042	24| 0.00991	25| 0.00977
Shaanxi	56| 0.00814	58| 0.00789	59| 0.00768	60| 0.00751	61| 0.00739	66| 0.00729	18| 0.02499	21| 0.0212	24| 0.01896	25| 0.01777	26| 0.01734	26| 0.01748
Shandong	75| 0.0063	68| 0.0067	65| 0.00698	63| 0.00716	62| 0.00729	62| 0.00737	14| 0.03149	17| 0.02652	18| 0.02455	19| 0.02397	19| 0.02413	18| 0.02473
Shanghai	56| 0.00752	61| 0.00696	65| 0.0065	69| 0.00614	72| 0.00586	80| 0.00565	17| 0.02411	16| 0.02581	16| 0.02606	16| 0.02588	16| 0.02579	16| 0.02593
Shanxi	79| 0.00485	71| 0.00515	68| 0.00541	65| 0.00561	63| 0.00578	62| 0.0059	13| 0.0282	15| 0.02449	16| 0.02274	16| 0.0222	16| 0.02249	16| 0.02335
Sichuan	46| 0.00753	54| 0.00647	56| 0.00619	58| 0.00616	56| 0.00621	55| 0.0063	19| 0.01846	23| 0.0148	28| 0.01237	32| 0.01081	35| 0.00982	38| 0.00921
Tianjin	78| 0.00487	72| 0.00506	70| 0.00521	69| 0.00531	68| 0.00535	68| 0.00536	14| 0.02531	13| 0.0285	12| 0.02995	12| 0.0304	12| 0.03046	12| 0.03044
Tibet	56| 0.00405	51| 0.00416	48| 0.00443	45| 0.00472	43| 0.00498	41| 0.00518	36| 0.00591	36| 0.00585	36| 0.00585	36| 0.00588	36| 0.00592	36| 0.00596
Xinjiang	55| 0.0072	58| 0.00676	60| 0.00658	61| 0.00651	62| 0.0065	61| 0.00652	16| 0.025	21| 0.01887	25| 0.01593	27| 0.01465	27| 0.01435	27| 0.01479
Yunnan	41| 0.00686	46| 0.00605	51| 0.00554	54| 0.0052	56| 0.00501	60| 0.00491	26| 0.01096	29| 0.00957	32| 0.00875	34| 0.00827	35| 0.00799	36| 0.00782
Zhejiang	59| 0.00932	61| 0.00896	62| 0.00879	63| 0.00867	64| 0.00856	66| 0.00845	18| 0.03072	20| 0.02712	21| 0.0256	22| 0.02497	22| 0.02475	22| 0.02473
Democratic People's Republic of Korea	31| 0.01371	35| 0.01243	38| 0.01125	42| 0.0102	47| 0.00928	51| 0.00847	54| 0.00838	46| 0.00939	42| 0.01032	39| 0.011	38| 0.01145	37| 0.01173
Taiwan (Province of China)	87| 0.00579	73| 0.00678	64| 0.00772	59| 0.00839	55| 0.00887	54| 0.00916	19| 0.02612	19| 0.02623	18| 0.02652	18| 0.02714	17| 0.02796	17| 0.02881
American Samoa	87| 0.00105	74| 0.00122	62| 0.00146	53| 0.00169	48| 0.00188	44| 0.00205	22| 0.00415	22| 0.004	22| 0.00408	22| 0.00414	22| 0.00415	22| 0.00415
Cook Islands	75| 0.00119	79| 0.00113	82| 0.00113	78| 0.00115	76| 0.00118	73| 0.00122	7| 0.01299	6| 0.01384	6| 0.01419	6| 0.0143	6| 0.01445	6| 0.01483
Fiji	63| 0.00485	60| 0.00487	59| 0.00494	58| 0.00501	58| 0.00509	57| 0.00515	16| 0.01819	21| 0.01387	26| 0.01125	29| 0.01021	28| 0.01053	25| 0.01179
Guam	64| 0.00645	58| 0.00666	57| 0.00673	57| 0.00675	57| 0.00673	57| 0.00673	26| 0.01467	24| 0.0157	25| 0.0156	25| 0.01509	26| 0.015	24| 0.01597
Kiribati	62| 0.00545	56| 0.00569	53| 0.00596	51| 0.00622	49| 0.00645	48| 0.00665	27| 0.01154	30| 0.01053	31| 0.01027	31| 0.01027	31| 0.01032	31| 0.01036
Marshall Islands	54| 0.00826	47| 0.00891	45| 0.00938	43| 0.0097	42| 0.00992	42| 0.01007	30| 0.01401	37| 0.01122	41| 0.01016	41| 0.01014	40| 0.01053	38| 0.01101
Micronesia (Federated States of)	70| 0.00388	57| 0.00458	50| 0.0052	46| 0.00566	43| 0.00598	42| 0.00619	29| 0.00891	35| 0.00751	35| 0.00738	33| 0.00783	31| 0.00838	29| 0.00888
Nauru	108| 0.00174	90| 0.00201	79| 0.00229	71| 0.00253	66| 0.00274	62| 0.00291	3| 0.06131	5| 0.03798	4| 0.03828	4| 0.04083	4| 0.04184	4| 0.04194
Niue	58| 0.00145	64| 0.00132	69| 0.00122	73| 0.00115	77| 0.00109	85| 0.00104	11| 0.00765	12| 0.00691	12| 0.00669	13| 0.00664	13| 0.00665	13| 0.00664
Northern Mariana Islands	52| 0.00289	62| 0.00242	72| 0.00208	81| 0.00184	89| 0.00168	95| 0.00158	7| 0.02002	8| 0.01838	9| 0.01708	9| 0.01668	8| 0.01741	8| 0.01925
Palau	74| 0.00264	68| 0.00269	66| 0.00277	64| 0.00285	63| 0.00291	62| 0.00296	17| 0.01083	19| 0.00981	20| 0.00922	18| 0.00987	16| 0.01152	13| 0.01346
Papua New Guinea	45| 0.00947	48| 0.00888	51| 0.00832	54| 0.00782	58| 0.00738	64| 0.00699	32| 0.01324	30| 0.01412	29| 0.01468	29| 0.01477	29| 0.01445	31| 0.01389
Samoa	72| 0.00271	64| 0.00289	60| 0.0031	56| 0.00331	53| 0.00351	50| 0.00369	23| 0.00803	24| 0.00763	25| 0.00751	25| 0.00751	24| 0.00756	24| 0.00763
Solomon Islands	70| 0.00369	61| 0.00395	57| 0.00425	53| 0.00453	51| 0.00478	48| 0.005	28| 0.0086	27| 0.00884	26| 0.00921	26| 0.00936	26| 0.00923	27| 0.00897
Tokelau	52| 0.00399	53| 0.00387	55| 0.00375	57| 0.00365	58| 0.00356	64| 0.00347	19| 0.01063	29| 0.00699	33| 0.00618	31| 0.00658	27| 0.0077	22| 0.00917
Tonga	56| 0.00471	59| 0.00452	60| 0.00439	64| 0.00433	61| 0.00433	61| 0.00437	21| 0.01263	26| 0.01022	27| 0.00994	24| 0.01077	22| 0.01221	19| 0.01394

Tuvalu	60| 0.00171	81| 0.00132	76| 0.00135	69| 0.00148	64| 0.00161	60| 0.00171	17| 0.00594	17| 0.00596	17| 0.00602	15| 0.00655	13| 0.00765	11| 0.00911
Vanuatu	49| 0.0069	56| 0.00601	60| 0.00558	62| 0.00539	63| 0.00532	67| 0.00531	20| 0.01637	22| 0.01515	24| 0.0142	25| 0.0135	26| 0.01304	26| 0.01277
Cambodia	30| 0.00414	35| 0.00348	40| 0.00309	43| 0.00287	45| 0.00273	47| 0.00263	46| 0.00265	49| 0.00263	44| 0.00277	42| 0.00294	40| 0.00307	39| 0.00315
Indonesia	37| 0.0188	37| 0.01887	38| 0.0185	39| 0.01788	40| 0.01732	42| 0.01676	67| 0.01074	52| 0.01353	41| 0.01706	37| 0.01903	35| 0.01974	35| 0.01975
Lao People's Democratic Republic	41| 0.01782	38| 0.01957	36| 0.02043	36| 0.02072	36| 0.02067	36| 0.02044	39| 0.01884	42| 0.01742	45| 0.01637	47| 0.0156	49| 0.01504	55| 0.01461
Malaysia	63| 0.01213	56| 0.01311	52| 0.01402	50| 0.01472	48| 0.01524	47| 0.01561	31| 0.02354	31| 0.02344	31| 0.02359	31| 0.02378	30| 0.02398	30| 0.02421
Maldives	33| 0.01508	29| 0.01678	28| 0.01768	27| 0.01804	27| 0.0183	27| 0.01841	72| 0.00723	63| 0.00778	57| 0.00861	51| 0.00958	46| 0.0108	40| 0.01221
Mauritius	26| 0.01832	27| 0.01765	29| 0.01692	30| 0.01618	31| 0.01547	33| 0.01481	44| 0.01097	55| 0.00875	56| 0.00869	55| 0.00888	55| 0.00885	59| 0.00863
Myanmar	39| 0.00892	41| 0.0085	43| 0.00816	44| 0.0079	45| 0.00771	46| 0.00755	24| 0.01449	31| 0.01107	39| 0.00881	46| 0.00756	49| 0.00704	53| 0.00692
Philippines	43| 0.00952	57| 0.00731	69| 0.00603	76| 0.00546	81| 0.00512	88| 0.0049	18| 0.02294	14| 0.0282	13| 0.03059	13| 0.03091	14| 0.03006	14| 0.02876
Seychelles	37| 0.01417	44| 0.01196	51| 0.01037	57| 0.00927	62| 0.00853	71| 0.008	26| 0.02069	29| 0.01809	32| 0.01642	33| 0.01606	31| 0.01713	27| 0.01928
Sri Lanka	38| 0.01175	43| 0.01046	46| 0.00972	48| 0.00938	51| 0.00928	48| 0.00931	31| 0.01425	38| 0.01178	41| 0.01092	41| 0.01087	39| 0.01136	36| 0.01222
Thailand	47| 0.00775	49| 0.00749	50| 0.00725	52| 0.00704	53| 0.00686	56| 0.00671	19| 0.01901	27| 0.0133	34| 0.01076	37| 0.00992	38| 0.00964	38| 0.0095
Timor-Leste	49| 0.00529	51| 0.00509	52| 0.00491	54| 0.00475	56| 0.00463	60| 0.00451	29| 0.00886	29| 0.00888	29| 0.00887	29| 0.00874	30| 0.00848	32| 0.00814
Viet Nam	47| 0.00654	52| 0.00617	46| 0.00677	41| 0.00751	38| 0.00817	35| 0.00867	26| 0.01191	33| 0.00936	40| 0.00764	46| 0.00673	48| 0.00642	48| 0.00649
Angola	34| 0.01003	36| 0.00948	37| 0.00913	38| 0.00889	39| 0.00871	39| 0.00859	28| 0.01188	38| 0.00884	49| 0.00698	57| 0.00633	54| 0.00633	51| 0.00662
Central African Republic	28| 0.00957	32| 0.00837	36| 0.00745	40| 0.00674	44| 0.0062	51| 0.00578	45| 0.00596	46| 0.00593	45| 0.00595	45| 0.00601	44| 0.0061	44| 0.00621
Congo	61| 0.00626	61| 0.00621	62| 0.00612	63| 0.00601	65| 0.00588	68| 0.00576	16| 0.02399	18| 0.02119	20| 0.0192	21| 0.01783	22| 0.01689	23| 0.01621
Democratic Republic of the Congo	52| 0.01388	56| 0.01289	60| 0.01206	64| 0.01138	67| 0.01081	74| 0.01034	17| 0.0414	22| 0.0332	22| 0.03273	22| 0.03321	22| 0.03331	22| 0.03316
Equatorial Guinea	44| 0.01144	45| 0.0111	47| 0.01061	50| 0.01004	52| 0.00955	56| 0.00905	20| 0.02526	26| 0.01931	32| 0.01556	38| 0.01323	43| 0.01164	47| 0.01049
Gabon	45| 0.00634	51| 0.00554	56| 0.00504	60| 0.00473	63| 0.00453	66| 0.0044	25| 0.01141	29| 0.00976	31| 0.00919	28| 0.01025	24| 0.01176	22| 0.01295
Burundi	27| 0.00827	30| 0.00769	31| 0.00725	33| 0.00692	34| 0.00666	35| 0.00644	38| 0.00601	45| 0.00503	52| 0.00434	57| 0.00401	61| 0.00395	57| 0.00403
Comoros	37| 0.00425	44| 0.00361	48| 0.00331	50| 0.00317	52| 0.00309	55| 0.00305	25| 0.00645	31| 0.00518	36| 0.00438	40| 0.00399	41| 0.00388	41| 0.00393
Djibouti	62| 0.0038	65| 0.00376	62| 0.0038	61| 0.00385	61| 0.00388	60| 0.0039	13| 0.01759	16| 0.01443	20| 0.01194	23| 0.01006	27| 0.00871	30| 0.00776
Eritrea	41| 0.00681	41| 0.00677	41| 0.00678	41| 0.00679	41| 0.00678	41| 0.00677	34| 0.00823	37| 0.00754	41| 0.00684	45| 0.00628	51| 0.00598	46| 0.00604
Ethiopia	49| 0.00711	50| 0.00687	53| 0.00655	56| 0.00623	58| 0.00595	64| 0.00571	21| 0.01608	25| 0.01392	28| 0.01236	31| 0.01132	32| 0.01069	33| 0.01037
Kenya	49| 0.01122	52| 0.01058	55| 0.01002	58| 0.00955	60| 0.00916	65| 0.00884	20| 0.02739	23| 0.02421	25| 0.02153	28| 0.01933	31| 0.01756	34| 0.01616
Madagascar	27| 0.01023	28| 0.0097	30| 0.00919	31| 0.00873	33| 0.00832	35| 0.00795	38| 0.00722	47| 0.00588	53| 0.00516	57| 0.00483	59| 0.0047	62| 0.00468
Malawi	59| 0.00779	50| 0.00847	46| 0.00914	44| 0.00972	42| 0.0102	40| 0.01058	30| 0.01412	37| 0.01136	41| 0.01031	40| 0.01054	37| 0.01136	34| 0.01236

Mozambique	22| 0.00817	24| 0.00772	25| 0.0074	26| 0.00717	26| 0.00699	27| 0.00684	51| 0.00362	56| 0.00327	60| 0.00309	64| 0.00304	60| 0.00305	59| 0.0031
Rwanda	31| 0.01032	33| 0.00997	34| 0.00965	35| 0.00936	36| 0.0091	37| 0.00886	61| 0.00556	55| 0.0059	49| 0.00661	45| 0.00722	43| 0.00765	41| 0.00793
Somalia	35| 0.01194	38| 0.01089	41| 0.0101	44| 0.00951	46| 0.00906	53| 0.00871	30| 0.01366	36| 0.01144	41| 0.01012	44| 0.00944	46| 0.00913	46| 0.00901
South Sudan	44| 0.0052	48| 0.00471	52| 0.00438	55| 0.00416	57| 0.00401	61| 0.00389	31| 0.00737	31| 0.00739	31| 0.00741	30| 0.00743	30| 0.00746	30| 0.00748
Uganda	40| 0.00759	45| 0.00688	46| 0.00662	48| 0.0066	46| 0.00665	46| 0.0067	39| 0.00793	40| 0.0077	39| 0.00786	38| 0.00813	37| 0.00838	36| 0.00859
United Republic of Tanzania	56| 0.00813	49| 0.00898	46| 0.00955	44| 0.00988	44| 0.01005	43| 0.01011	28| 0.01577	33| 0.01332	37| 0.01186	39| 0.01115	40| 0.01086	41| 0.01075
Zambia	58| 0.00882	51| 0.00931	49| 0.0096	49| 0.00975	48| 0.00981	48| 0.00981	18| 0.02581	25| 0.01894	33| 0.01453	39| 0.01223	41| 0.01145	41| 0.01152
Botswana	45| 0.00565	53| 0.00476	61| 0.00419	66| 0.00382	70| 0.0036	77| 0.00348	11| 0.02223	15| 0.01665	19| 0.01294	24| 0.01057	28| 0.00905	31| 0.00806
Eswatini	37| 0.00819	43| 0.00706	49| 0.00625	54| 0.00569	58| 0.00531	65| 0.00507	28| 0.01092	31| 0.00982	33| 0.00929	34| 0.00902	34| 0.00897	34| 0.00908
Lesotho	74| 0.00379	61| 0.00445	53| 0.0051	48| 0.00557	46| 0.00589	44| 0.00609	23| 0.01153	27| 0.01001	29| 0.00913	31| 0.00869	32| 0.00853	32| 0.00849
Namibia	59| 0.00831	50| 0.00939	46| 0.01022	43| 0.01081	42| 0.01123	41| 0.01152	34| 0.01384	41| 0.01148	41| 0.01141	38| 0.01243	34| 0.0137	31| 0.01492
South Africa	61| 0.01185	54| 0.01295	51| 0.01365	50| 0.01409	49| 0.01436	48| 0.01453	29| 0.02444	31| 0.02253	32| 0.02192	32| 0.02188	32| 0.02204	31| 0.02224
Zimbabwe	87| 0.0057	82| 0.00578	78| 0.00614	73| 0.00649	70| 0.00678	68| 0.00699	7| 0.06363	8| 0.05739	8| 0.0601	7| 0.06832	6| 0.0743	6| 0.07732
Benin	47| 0.00912	44| 0.00915	44| 0.00923	44| 0.00932	43| 0.00941	43| 0.00951	35| 0.01143	38| 0.01078	39| 0.01028	41| 0.00999	41| 0.00988	41| 0.00993
Burkina Faso	39| 0.00636	40| 0.00618	40| 0.00609	41| 0.00603	41| 0.00601	41| 0.00599	47| 0.0058	43| 0.0058	42| 0.0058	42| 0.00581	42| 0.00581	42| 0.00581
Cabo Verde	17| 0.01252	20| 0.0109	22| 0.00978	24| 0.00899	25| 0.00841	27| 0.00797	37| 0.00588	75| 0.00286	97| 0.00228	66| 0.00329	49| 0.00438	41| 0.00529
Cameroon	60| 0.01081	53| 0.01155	51| 0.0121	49| 0.01247	48| 0.0127	48| 0.01284	28| 0.02216	30| 0.0203	32| 0.01902	33| 0.01833	34| 0.01807	34| 0.01808
Chad	38| 0.00619	40| 0.00592	41| 0.00572	42| 0.00558	43| 0.0055	43| 0.00541	40| 0.00584	43| 0.00545	47| 0.00535	43| 0.0055	41| 0.00577	39| 0.00604
Côte d'Ivoire	46| 0.00927	50| 0.00856	54| 0.00787	58| 0.0073	62| 0.00683	69| 0.00647	30| 0.01415	30| 0.01425	28| 0.01498	26| 0.01615	24| 0.01746	23| 0.01869
Gambia	30| 0.01885	32| 0.01781	33| 0.017	35| 0.01637	36| 0.01587	37| 0.01547	44| 0.01287	47| 0.01221	49| 0.01168	50| 0.01128	52| 0.011	55| 0.0108
Ghana	40| 0.01651	42| 0.01558	44| 0.01488	46| 0.01431	47| 0.01385	52| 0.01349	35| 0.01865	37| 0.01772	38| 0.01711	39| 0.01671	40| 0.01645	40| 0.01627
Guinea	27| 0.00956	31| 0.00857	34| 0.00781	36| 0.00721	39| 0.00672	42| 0.00631	46| 0.00572	47| 0.00553	49| 0.00539	52| 0.00532	49| 0.00534	48| 0.00544
Guinea-Bissau	29| 0.00705	32| 0.00637	35| 0.00586	37| 0.00548	39| 0.00518	41| 0.00494	50| 0.00408	52| 0.00405	49| 0.00418	47| 0.00437	45| 0.00452	44| 0.0046
Liberia	46| 0.01023	51| 0.00928	55| 0.00864	58| 0.00823	59| 0.00798	64| 0.00781	27| 0.01747	28| 0.01694	28| 0.01687	28| 0.0169	28| 0.01689	28| 0.01684
Mali	33| 0.0134	36| 0.01234	38| 0.01165	40| 0.0112	41| 0.01091	42| 0.01072	52| 0.00947	46| 0.00966	45| 0.00995	44| 0.01029	42| 0.01062	41| 0.01095
Mauritania	58| 0.00794	58| 0.00791	58| 0.0079	63| 0.0079	58| 0.0079	58| 0.00791	20| 0.02293	21| 0.02143	23| 0.01987	25| 0.01824	28| 0.01661	30| 0.01512
Niger	32| 0.00266	35| 0.00239	38| 0.0022	40| 0.0021	41| 0.00206	42| 0.002	51| 0.00186	45| 0.00187	45| 0.00189	44| 0.00191	44| 0.00194	43| 0.00197
Nigeria	60| 0.00842	54| 0.00888	51| 0.0093	49| 0.00963	48| 0.0099	47| 0.01011	24| 0.02011	31| 0.01509	36| 0.01316	36| 0.01328	33| 0.01418	31| 0.01521
Sao Tome and Principe	38| 0.01173	42| 0.01069	46| 0.00981	50| 0.00908	53| 0.00847	61| 0.00797	19| 0.02355	25| 0.01788	32| 0.01392	40| 0.01142	45| 0.00999	49| 0.0093

Senegal	50| 0.01235	44| 0.01289	43| 0.01331	42| 0.01365	41| 0.01394	41| 0.01414	42| 0.01363	41| 0.01399	40| 0.01429	39| 0.01453	39| 0.01476	38| 0.01498
Sierra Leone	22| 0.01773	23| 0.01649	25| 0.01549	26| 0.01467	28| 0.01398	29| 0.0134	51| 0.0076	58| 0.0067	63| 0.00636	60| 0.00641	58| 0.00661	57| 0.00683
Togo	37| 0.01318	40| 0.01217	43| 0.01136	46| 0.0107	48| 0.01016	54| 0.0097	45| 0.01091	42| 0.01165	39| 0.01257	37| 0.01342	35| 0.01409	34| 0.01457

*The number of draws is weighted by the inverse RMSE and sampled proportionally out of a total of 500 draws

Table S5 Out-of-sample (OOS) RMSE and draws selected for proportion of obese among overweight forecast sub-models

	ARC (draws | RMSE)						MR-BRT (draws | RMSE)					
Location	omega 0	omega 0.5	omega 1	omega 1.5	omega 2	omega 2.5	omega 0	omega 0.5	omega 1	omega 1.5	omega 2	omega 2.5
Armenia	50| 0.00532	60| 0.00445	68| 0.00419	63| 0.0042	62| 0.00429	61| 0.00438	30| 0.00876	27| 0.00978	23| 0.01131	21| 0.01289	18| 0.0143	17| 0.01547
Azerbaijan	56| 0.00677	62| 0.00604	66| 0.00569	72| 0.00561	67| 0.00564	66| 0.00569	21| 0.01763	20| 0.01844	19| 0.01964	18| 0.02082	17| 0.02183	16| 0.02265
Georgia	45| 0.01694	48| 0.01592	52| 0.01482	55| 0.01386	59| 0.01309	65| 0.01249	28| 0.02724	29| 0.02605	30| 0.02576	30| 0.02576	30| 0.02589	29| 0.02608
Kazakhstan	26| 0.00733	32| 0.00597	39| 0.00493	47| 0.00415	54| 0.00357	65| 0.00316	46| 0.00424	43| 0.00454	40| 0.00482	38| 0.00508	36| 0.00537	34| 0.00571
Kyrgyzstan	35| 0.00899	47| 0.00663	65| 0.00486	84| 0.00374	100| 0.00314	113| 0.00284	10| 0.03069	9| 0.03399	9| 0.03496	9| 0.03418	9| 0.03266	10| 0.03109
Mongolia	42| 0.00816	43| 0.00799	43| 0.00788	44| 0.00782	44| 0.00778	46| 0.00775	37| 0.00915	41| 0.00835	41| 0.00828	40| 0.00842	40| 0.00859	39| 0.00876
Tajikistan	47| 0.00734	57| 0.00616	66| 0.00529	73| 0.00478	80| 0.00462	74| 0.00468	18| 0.01873	18| 0.01927	17| 0.01979	17| 0.02027	17| 0.0207	16| 0.02108
Turkmenistan	37| 0.008	40| 0.00732	43| 0.00691	44| 0.00665	45| 0.0065	46| 0.0064	46| 0.00646	49| 0.00632	43| 0.00688	39| 0.00762	35| 0.00831	33| 0.00889
Uzbekistan	35| 0.01329	32| 0.01433	31| 0.01498	30| 0.01534	30| 0.01549	30| 0.01551	60| 0.00813	56| 0.0083	52| 0.00897	49| 0.00947	48| 0.00976	47| 0.00993
Albania	41| 0.00789	51| 0.00643	56| 0.00586	61| 0.00571	57| 0.00571	57| 0.00578	34| 0.00947	32| 0.01012	30| 0.0109	28| 0.01163	27| 0.01221	26| 0.01264
Bosnia and Herzegovina	46| 0.0077	58| 0.00607	65| 0.00544	70| 0.00527	67| 0.00529	66| 0.00537	19| 0.0184	20| 0.0177	21| 0.01709	21| 0.0164	23| 0.01558	24| 0.01469
Bulgaria	48| 0.00348	57| 0.00296	62| 0.00269	66| 0.00256	69| 0.00254	66| 0.00256	26| 0.00641	24| 0.00699	22| 0.00753	21| 0.00796	20| 0.0083	19| 0.00856
Croatia	76| 0.00321	69| 0.00342	65| 0.00361	63| 0.00377	61| 0.00389	59| 0.00399	20| 0.01174	18| 0.01331	17| 0.01396	17| 0.01392	17| 0.01353	18| 0.01303
Czechia	54| 0.00669	58| 0.00624	61| 0.00593	63| 0.00575	65| 0.00562	70| 0.00553	20| 0.01815	21| 0.01702	22| 0.01611	23| 0.01589	22| 0.01623	21| 0.01684
Hungary	65| 0.00206	72| 0.00189	75| 0.00181	76| 0.00179	76| 0.00178	78| 0.00178	11| 0.01258	10| 0.01292	10| 0.01311	10| 0.01376	9| 0.01483	8| 0.01607
Montenegro	52| 0.00376	62| 0.00319	69| 0.00285	75| 0.00264	79| 0.00251	85| 0.00243	14| 0.0135	15| 0.01323	13| 0.01452	12| 0.01561	12| 0.01621	12| 0.01643
North Macedonia	60| 0.00354	61| 0.00351	64| 0.00336	67| 0.00318	71| 0.00301	78| 0.00287	21| 0.00994	17| 0.01242	15| 0.01369	15| 0.0141	15| 0.01399	16| 0.01362
Poland	52| 0.00587	55| 0.00552	57| 0.00538	59| 0.00535	57| 0.00539	56| 0.00545	31| 0.00993	28| 0.01106	27| 0.01127	27| 0.01136	26| 0.0117	25| 0.01227
Romania	33| 0.01171	35| 0.01086	37| 0.01029	38| 0.00993	39| 0.00969	40| 0.00952	54| 0.00759	49| 0.00775	46| 0.00828	44| 0.00867	43| 0.00891	42| 0.00908
Serbia	62| 0.00562	62| 0.00569	61| 0.00572	62| 0.00569	63| 0.0056	67| 0.0055	24| 0.01428	23| 0.0152	21| 0.01694	19| 0.01816	18| 0.01879	18| 0.01901

Slovakia	67| 0.00547	60| 0.00575	58| 0.0059	57| 0.00598	57| 0.006	57| 0.00599	25| 0.01371	25| 0.01395	25| 0.01389	24| 0.01412	23| 0.01464	22| 0.01525
Slovenia	50| 0.00713	53| 0.00667	57| 0.00629	59| 0.00602	61| 0.00584	67| 0.00573	24| 0.01448	25| 0.01393	26| 0.01354	26| 0.01339	26| 0.0135	26| 0.01388
Belarus	56| 0.00386	67| 0.00323	77| 0.00299	72| 0.00302	67| 0.00328	60| 0.00362	23| 0.00952	19| 0.01155	16| 0.0133	15| 0.01441	14| 0.01501	14| 0.01529
Estonia	65| 0.00383	73| 0.00359	65| 0.00383	61| 0.00407	59| 0.00421	58| 0.00429	21| 0.01194	21| 0.01173	21| 0.01205	19| 0.01269	19| 0.01329	18| 0.01369
Latvia	49| 0.00744	54| 0.00678	57| 0.00643	60| 0.00635	57| 0.00643	55| 0.00659	30| 0.01214	30| 0.0123	29| 0.01266	28| 0.01319	26| 0.01384	25| 0.01453
Lithuania	73| 0.00406	86| 0.00367	74| 0.00402	65| 0.00457	58| 0.0051	53| 0.00555	17| 0.01704	16| 0.01812	15| 0.01904	15| 0.01974	14| 0.02029	14| 0.02076
Republic of Moldova	80| 0.00339	130| 0.00213	77| 0.00353	53| 0.00511	43| 0.00635	37| 0.00726	14| 0.01862	17| 0.01596	15| 0.01795	13| 0.02118	11| 0.02406	10| 0.0263
Russian Federation	42| 0.01418	41| 0.01457	41| 0.01437	42| 0.01396	44| 0.01352	47| 0.01311	36| 0.01626	40| 0.01487	42| 0.01401	43| 0.01382	42| 0.01414	40| 0.01471
Ukraine	44| 0.00771	49| 0.00681	56| 0.00603	61| 0.00548	65| 0.00514	72| 0.00496	26| 0.01275	31| 0.01068	29| 0.0115	25| 0.01337	22| 0.01517	20| 0.01661
Australia	35| 0.01694	39| 0.01547	42| 0.01417	46| 0.01307	49| 0.01213	56| 0.01134	40| 0.0151	40| 0.01509	39| 0.01523	39| 0.0155	38| 0.01579	37| 0.01603
New Zealand	62| 0.01104	53| 0.01213	50| 0.01297	48| 0.0135	47| 0.01378	46| 0.01391	38| 0.01681	37| 0.01765	33| 0.01927	31| 0.02108	28| 0.02264	27| 0.02372
Brunei Darussalam	43| 0.00897	40| 0.00963	38| 0.01001	38| 0.01023	37| 0.01034	37| 0.0104	40| 0.00958	40| 0.00969	42| 0.0092	45| 0.00858	48| 0.00805	52| 0.00773
Japan	46| 0.00713	50| 0.00711	46| 0.00716	46| 0.00722	45| 0.00726	45| 0.00729	37| 0.00889	37| 0.00885	37| 0.00886	37| 0.0089	37| 0.00895	37| 0.00899
Republic of Korea	28| 0.01172	30| 0.01094	32| 0.01021	34| 0.00957	36| 0.00904	38| 0.0086	50| 0.00652	55| 0.00644	51| 0.00644	50| 0.00654	49| 0.00671	47| 0.00691
Singapore	35| 0.01448	37| 0.01373	38| 0.01309	40| 0.01255	42| 0.01211	43| 0.01176	41| 0.01236	42| 0.01189	44| 0.01148	45| 0.01126	45| 0.01117	48| 0.01115
Canada	48| 0.00578	49| 0.00562	50| 0.00553	50| 0.00548	51| 0.00544	52| 0.0054	32| 0.00844	33| 0.0084	33| 0.00829	34| 0.00817	34| 0.00812	34| 0.00818
Greenland	42| 0.00839	48| 0.00731	53| 0.00656	57| 0.00608	60| 0.0058	65| 0.00564	33| 0.01061	30| 0.01162	28| 0.01222	28| 0.01247	28| 0.01253	28| 0.01253
United States of America	37| 0.02057	42| 0.01827	47| 0.01622	53| 0.01455	58| 0.01322	65| 0.0122	26| 0.02969	28| 0.02692	32| 0.02414	35| 0.02185	38| 0.02038	39| 0.01985
Alabama	43| 0.02126	49| 0.01854	56| 0.01634	61| 0.01484	65| 0.01401	69| 0.01373	21| 0.04224	24| 0.03762	27| 0.03392	28| 0.03179	29| 0.03129	28| 0.0321
Alaska	47| 0.03165	50| 0.02977	53| 0.02834	54| 0.02744	56| 0.02693	58| 0.02666	28| 0.05361	29| 0.05192	30| 0.05007	31| 0.04836	32| 0.04704	32| 0.0462
Arizona	47| 0.02472	46| 0.02527	46| 0.02535	46| 0.02513	47| 0.02475	51| 0.02432	30| 0.03882	34| 0.03441	37| 0.03144	39| 0.03002	39| 0.0298	38| 0.03036
Arkansas	51| 0.0178	54| 0.01679	56| 0.01609	58| 0.01562	59| 0.01532	61| 0.01511	24| 0.03708	25| 0.03525	27| 0.0336	28| 0.03236	28| 0.03159	29| 0.03127
California	37| 0.02804	44| 0.02386	51| 0.02073	56| 0.01863	61| 0.01726	67| 0.01641	26| 0.03992	28| 0.03672	31| 0.03415	32| 0.03239	33| 0.03144	34| 0.03118
Colorado	52| 0.02051	55| 0.01921	58| 0.01813	61| 0.01725	64| 0.01655	70| 0.01601	22| 0.04723	23| 0.04587	23| 0.04476	24| 0.0441	24| 0.04384	24| 0.04387
Connecticut	50| 0.02189	55| 0.02188	50| 0.02205	49| 0.02227	49| 0.02248	49| 0.02266	33| 0.03321	33| 0.03325	33| 0.03319	33| 0.03307	33| 0.03293	33| 0.03281
Delaware	49| 0.02603	49| 0.02604	49| 0.02571	50| 0.02527	51| 0.02478	55| 0.02428	33| 0.03827	33| 0.03807	33| 0.03797	33| 0.03814	33| 0.03857	32| 0.0392
District of Columbia	47| 0.02465	49| 0.02364	51| 0.02271	52| 0.02206	53| 0.02161	56| 0.02128	33| 0.03509	33| 0.03526	32| 0.03556	32| 0.03596	31| 0.03649	31| 0.03714
Florida	41| 0.02687	43| 0.02545	45| 0.02444	46| 0.02368	48| 0.02307	51| 0.02254	33| 0.03336	35| 0.03097	38| 0.02896	40| 0.02773	40| 0.02727	40| 0.0274
Georgia	47| 0.02373	51| 0.02189	57| 0.01977	63| 0.01798	67| 0.01671	74| 0.01593	17| 0.06418	20| 0.05653	23| 0.04849	26| 0.0428	28| 0.04042	27| 0.0407

Hawaii	42| 0.03702	42| 0.03682	42| 0.03664	43| 0.0365	43| 0.03642	45| 0.03637	41| 0.03819	40| 0.03879	40| 0.03902	40| 0.03884	41| 0.03836	41| 0.03777
Idaho	35| 0.04357	37| 0.04135	39| 0.03989	40| 0.03891	40| 0.03825	41| 0.03779	40| 0.03883	42| 0.0367	44| 0.03487	46| 0.03372	50| 0.03345	46| 0.03403
Illinois	48| 0.01986	48| 0.01981	49| 0.01938	51| 0.01884	52| 0.01833	58| 0.01792	32| 0.02976	32| 0.02977	32| 0.02967	32| 0.02949	33| 0.0293	33| 0.02917
Indiana	47| 0.02059	48| 0.02017	49| 0.0199	49| 0.01967	50| 0.01947	55| 0.0193	31| 0.03106	33| 0.02949	34| 0.02829	35| 0.02766	35| 0.02765	34| 0.02819
Iowa	47| 0.02293	52| 0.02263	48| 0.02268	48| 0.02281	47| 0.02293	47| 0.02301	33| 0.03295	34| 0.03153	36| 0.0306	36| 0.03014	36| 0.03007	36| 0.0303
Kansas	54| 0.02332	53| 0.02373	54| 0.02327	56| 0.02266	57| 0.02219	61| 0.02189	24| 0.05218	25| 0.05075	26| 0.04805	28| 0.04482	30| 0.04185	32| 0.03974
Kentucky	43| 0.02106	45| 0.01975	48| 0.01877	50| 0.01802	51| 0.01744	56| 0.01697	34| 0.02659	34| 0.02637	34| 0.02604	35| 0.02572	35| 0.02554	35| 0.02563
Louisiana	44| 0.03133	47| 0.02959	49| 0.02823	51| 0.02707	53| 0.02609	60| 0.02523	32| 0.04329	32| 0.0427	33| 0.0423	33| 0.04206	33| 0.04198	33| 0.04203
Maine	45| 0.02415	46| 0.02357	47| 0.02319	47| 0.02298	47| 0.02289	49| 0.02288	35| 0.03117	35| 0.03092	36| 0.03024	37| 0.0294	38| 0.02864	38| 0.02809
Maryland	42| 0.02317	45| 0.0216	47| 0.02039	50| 0.01942	52| 0.01861	57| 0.01794	32| 0.02996	33| 0.02905	34| 0.02803	35| 0.02712	36| 0.02643	37| 0.026
Massachusetts	38| 0.02958	38| 0.02957	38| 0.02928	39| 0.02884	39| 0.02835	40| 0.02787	47| 0.02416	45| 0.02483	44| 0.02531	44| 0.02552	44| 0.0255	44| 0.02531
Michigan	44| 0.02401	47| 0.02236	50| 0.0211	53| 0.02018	54| 0.01954	58| 0.01911	33| 0.03252	32| 0.03306	32| 0.03329	32| 0.03319	32| 0.03285	33| 0.03239
Minnesota	43| 0.01857	48| 0.01671	53| 0.0151	58| 0.01381	61| 0.01293	65| 0.01248	24| 0.03311	25| 0.03109	28| 0.02865	30| 0.0264	32| 0.02484	33| 0.02428
Mississippi	42| 0.03354	45| 0.03181	47| 0.03038	49| 0.02917	50| 0.02813	55| 0.02723	30| 0.04654	32| 0.04399	35| 0.04093	37| 0.03818	39| 0.03652	39| 0.03621
Missouri	43| 0.02083	48| 0.01857	52| 0.01727	54| 0.01663	54| 0.01635	56| 0.01627	27| 0.03274	30| 0.02984	33| 0.02724	35| 0.02574	35| 0.02562	33| 0.02667
Montana	45| 0.03155	48| 0.03153	45| 0.0317	45| 0.03202	44| 0.0324	44| 0.03283	37| 0.03884	37| 0.03884	37| 0.03852	38| 0.03775	39| 0.03661	41| 0.03534
Nebraska	49| 0.01977	54| 0.01804	58| 0.01674	62| 0.01572	65| 0.01489	71| 0.01422	19| 0.05084	20| 0.04707	22| 0.04278	25| 0.03885	27| 0.03588	28| 0.0341
Nevada	46| 0.02927	46| 0.02934	47| 0.02862	49| 0.02764	51| 0.02667	57| 0.02581	33| 0.04105	34| 0.0395	35| 0.03855	35| 0.03864	34| 0.03967	33| 0.0413
New Hampshire	46| 0.03336	45| 0.03415	46| 0.03403	46| 0.0335	47| 0.03281	52| 0.03212	36| 0.04337	36| 0.04337	36| 0.04305	36| 0.04255	37| 0.04197	37| 0.04135
New Jersey	38| 0.03862	40| 0.03708	41| 0.03535	43| 0.03385	45| 0.03265	51| 0.0317	38| 0.03852	39| 0.03743	40| 0.03626	41| 0.03537	42| 0.03489	42| 0.0348
New Mexico	40| 0.02731	43| 0.02557	46| 0.02403	48| 0.02279	50| 0.02181	56| 0.02102	30| 0.03658	33| 0.03341	36| 0.03075	38| 0.02874	40| 0.02756	40| 0.02725
New York	33| 0.03459	36| 0.03158	39| 0.02921	41| 0.02724	44| 0.02555	50| 0.02407	39| 0.02857	41| 0.02757	42| 0.02666	44| 0.02583	45| 0.02511	46| 0.02452
North Carolina	48| 0.02132	50| 0.0201	52| 0.01935	53| 0.01903	56| 0.019	53| 0.01918	25| 0.04079	28| 0.03649	31| 0.03252	34| 0.02989	35| 0.0288	35| 0.02895
North Dakota	53| 0.0226	56| 0.02164	57| 0.02097	59| 0.02049	60| 0.02014	62| 0.01986	28| 0.04329	27| 0.0448	26| 0.04671	25| 0.04866	24| 0.05021	23| 0.05104
Ohio	46| 0.02164	47| 0.02087	49| 0.02022	50| 0.0197	51| 0.01932	57| 0.01905	30| 0.03268	31| 0.03148	33| 0.03005	34| 0.02871	36| 0.02772	36| 0.02726
Oklahoma	47| 0.02566	49| 0.02476	52| 0.02333	55| 0.0219	58| 0.02066	64| 0.01964	23| 0.05286	25| 0.04703	29| 0.04187	31| 0.03815	33| 0.0361	34| 0.03556
Oregon	41| 0.02697	45| 0.02483	49| 0.02288	52| 0.02128	56| 0.02002	60| 0.019	31| 0.0358	32| 0.03448	33| 0.03354	34| 0.03306	34| 0.03301	33| 0.0333
Pennsylvania	43| 0.02714	44| 0.0264	45| 0.02545	47| 0.02445	49| 0.02348	54| 0.02259	36| 0.03234	35| 0.03255	36| 0.03246	36| 0.032	37| 0.0313	38| 0.03055
Rhode Island	44| 0.02519	44| 0.02527	44| 0.02519	45| 0.025	45| 0.02472	49| 0.02442	37| 0.03022	37| 0.03004	38| 0.02955	38| 0.02892	39| 0.02832	40| 0.02785

South Carolina	47| 0.02289	51| 0.02121	55| 0.01967	58| 0.01837	62| 0.01739	66| 0.0167	24| 0.04537	24| 0.04498	25| 0.04246	27| 0.03907	30| 0.03613	31| 0.03433
South Dakota	49| 0.03297	49| 0.03274	51| 0.03182	53| 0.03066	55| 0.02952	60| 0.02849	31| 0.05264	29| 0.05489	29| 0.05559	30| 0.05437	31| 0.05175	33| 0.04871
Tennessee	44| 0.02891	50| 0.02544	56| 0.02284	60| 0.02099	64| 0.01968	70| 0.01874	21| 0.06123	23| 0.05575	25| 0.04988	28| 0.04539	29| 0.04298	30| 0.04235
Texas	48| 0.02271	50| 0.02151	52| 0.02098	55| 0.02084	52| 0.0209	51| 0.02106	24| 0.04482	29| 0.03718	33| 0.03235	36| 0.03029	36| 0.03025	34| 0.03141
Utah	44| 0.02612	48| 0.02351	53| 0.02159	56| 0.02023	59| 0.01929	64| 0.01864	26| 0.04367	28| 0.04041	30| 0.03797	31| 0.03679	31| 0.03683	30| 0.03779
Vermont	42| 0.02952	42| 0.02914	43| 0.02872	43| 0.02837	44| 0.0281	46| 0.02791	40| 0.03055	40| 0.03068	40| 0.03079	40| 0.03085	40| 0.03084	40| 0.03079
Virginia	44| 0.02018	48| 0.01846	52| 0.01713	54| 0.01623	56| 0.01571	59| 0.01546	30| 0.02955	32| 0.0277	33| 0.02688	32| 0.02726	31| 0.02852	29| 0.03025
Washington	40| 0.03202	42| 0.03003	45| 0.02801	49| 0.02622	52| 0.02473	57| 0.02352	34| 0.03702	35| 0.03671	35| 0.0361	36| 0.03525	37| 0.03429	38| 0.0334
West Virginia	52| 0.01834	55| 0.0172	59| 0.01614	62| 0.01528	65| 0.01464	69| 0.01417	23| 0.04024	23| 0.04145	22| 0.04202	23| 0.04165	23| 0.04047	24| 0.03893
Wisconsin	42| 0.02528	44| 0.02431	45| 0.02367	46| 0.0232	47| 0.02281	49| 0.02247	38| 0.02827	37| 0.02845	37| 0.0285	38| 0.02828	38| 0.02779	39| 0.02712
Wyoming	48| 0.02067	45| 0.02091	44| 0.02113	44| 0.02121	44| 0.0212	44| 0.02116	39| 0.02413	38| 0.02451	38| 0.02461	38| 0.0244	39| 0.02404	39| 0.0237
Argentina	55| 0.01039	50| 0.01064	50| 0.01074	50| 0.01074	50| 0.01069	51| 0.01061	36| 0.01495	36| 0.0148	34| 0.01573	31| 0.01705	29| 0.01823	28| 0.01909
Chile	48| 0.01085	48| 0.01087	48| 0.01087	48| 0.01086	48| 0.01082	49| 0.01078	38| 0.01355	37| 0.01391	36| 0.01436	35| 0.01486	33| 0.01547	32| 0.01622
Uruguay	36| 0.01216	38| 0.01158	39| 0.01118	40| 0.01088	41| 0.01062	42| 0.01038	40| 0.01096	43| 0.01022	44| 0.00983	45| 0.00966	47| 0.00966	45| 0.00978
Andorra	35| 0.00723	45| 0.00562	55| 0.00461	63| 0.00404	68| 0.00374	72| 0.00358	27| 0.00941	27| 0.00934	27| 0.00932	27| 0.00931	27| 0.00931	27| 0.0093
Austria	46| 0.00964	47| 0.0093	49| 0.00894	51| 0.00861	53| 0.00831	57| 0.00804	31| 0.01425	32| 0.01385	33| 0.01346	33| 0.01314	34| 0.0129	34| 0.01272
Belgium	32| 0.00871	37| 0.00742	43| 0.00645	49| 0.00571	54| 0.00513	60| 0.00468	38| 0.00735	37| 0.00754	36| 0.00761	37| 0.00753	38| 0.00736	39| 0.00716
Cyprus	52| 0.00835	53| 0.0081	55| 0.0078	58| 0.0075	60| 0.00721	65| 0.00695	26| 0.01673	26| 0.01632	27| 0.01595	27| 0.01614	26| 0.01673	25| 0.01744
Denmark	73| 0.00356	59| 0.00416	53| 0.00461	50| 0.00491	48| 0.00509	48| 0.00519	29| 0.0086	30| 0.00809	31| 0.00804	29| 0.00849	26| 0.00929	24| 0.01028
Finland	30| 0.00755	39| 0.00588	48| 0.00473	58| 0.00396	66| 0.00346	77| 0.00314	28| 0.00814	30| 0.00759	31| 0.00737	31| 0.00732	31| 0.00733	31| 0.00735
France	38| 0.01253	40| 0.01193	42| 0.01139	43| 0.01099	44| 0.01075	45| 0.01066	49| 0.01039	44| 0.01094	41| 0.01161	39| 0.01216	38| 0.0126	37| 0.01296
Germany	78| 0.00325	66| 0.00367	59| 0.00405	56| 0.00433	53| 0.00451	52| 0.00462	23| 0.01019	23| 0.01037	23| 0.01046	23| 0.01058	22| 0.01071	22| 0.01085
Greece	39| 0.01312	41| 0.01243	44| 0.01159	48| 0.01075	51| 0.00997	60| 0.00928	37| 0.01369	38| 0.01359	37| 0.0137	36| 0.01405	35| 0.0145	34| 0.01492
Iceland	28| 0.00603	30| 0.00557	32| 0.00522	34| 0.00496	35| 0.00478	36| 0.00464	48| 0.00354	50| 0.00339	51| 0.00333	51| 0.00332	51| 0.00331	54| 0.0033
Ireland	31| 0.01675	32| 0.01583	34| 0.01503	36| 0.01436	37| 0.01382	38| 0.01338	55| 0.01011	50| 0.01024	49| 0.01047	48| 0.01079	46| 0.01123	44| 0.0118
Israel	51| 0.00355	52| 0.00346	53| 0.00338	54| 0.00333	55| 0.00329	58| 0.00326	26| 0.00696	28| 0.00638	30| 0.00596	31| 0.00575	31| 0.00572	31| 0.00581
Italy	53| 0.01192	49| 0.01229	47| 0.01278	45| 0.01331	43| 0.01387	42| 0.01441	38| 0.01566	38| 0.01584	37| 0.01605	37| 0.0163	36| 0.01658	35| 0.0169
Luxembourg	49| 0.00493	51| 0.00475	53| 0.00459	55| 0.00442	57| 0.00426	60| 0.00412	30| 0.00807	29| 0.00816	29| 0.00822	29| 0.00827	29| 0.0083	29| 0.00833
Malta	21| 0.01532	23| 0.01406	25| 0.01283	28| 0.01171	30| 0.01074	33| 0.00988	64| 0.00513	68| 0.00506	61| 0.00533	55| 0.00593	49| 0.00673	43| 0.00758

Monaco	43| 0.00193	45| 0.00186	48| 0.00175	51| 0.00163	55| 0.00153	62| 0.00143	29| 0.00283	33| 0.00257	34| 0.00248	34| 0.00247	33| 0.00251	33| 0.00254
Netherlands	50| 0.01135	48| 0.01179	48| 0.01188	48| 0.01177	49| 0.01157	52| 0.01131	36| 0.0155	35| 0.01592	34| 0.01639	34| 0.01679	33| 0.01708	33| 0.01729
Norway	54| 0.00477	44| 0.00561	41| 0.00598	41| 0.00606	41| 0.00599	42| 0.00584	37| 0.00673	37| 0.00667	38| 0.00647	40| 0.00618	42| 0.00592	43| 0.00577
Portugal	45| 0.01031	45| 0.01029	46| 0.0102	46| 0.01009	47| 0.00997	51| 0.00984	38| 0.01215	38| 0.01238	37| 0.01261	36| 0.01286	36| 0.01312	35| 0.01337
San Marino	70| 0.00152	74| 0.00144	76| 0.0014	80| 0.00139	76| 0.00139	76| 0.00141	7| 0.01458	8| 0.01337	8| 0.01248	9| 0.01213	8| 0.01237	8| 0.01308
Spain	24| 0.01909	30| 0.01561	36| 0.01296	43| 0.011	49| 0.00954	58| 0.00844	43| 0.01087	44| 0.01066	44| 0.01063	44| 0.01074	43| 0.01095	42| 0.01123
Sweden	60| 0.00614	51| 0.00685	48| 0.00727	47| 0.00749	46| 0.00759	46| 0.00761	35| 0.01003	38| 0.00929	37| 0.00948	34| 0.01036	30| 0.01149	28| 0.01258
Switzerland	37| 0.01142	40| 0.01058	43| 0.00978	46| 0.00906	50| 0.00845	53| 0.00791	33| 0.01284	35| 0.01192	38| 0.01113	40| 0.0105	42| 0.01004	43| 0.00975
United Kingdom	39| 0.01055	41| 0.00999	44| 0.00943	46| 0.00891	49| 0.00843	54| 0.00801	43| 0.00953	41| 0.00999	39| 0.01063	36| 0.01129	35| 0.0119	33| 0.01241
Bolivia (Plurinational State of)	41| 0.00697	45| 0.00635	50| 0.00571	55| 0.00515	60| 0.00471	69| 0.00438	45| 0.00629	34| 0.00843	28| 0.01002	26| 0.01107	24| 0.01171	23| 0.01207
Ecuador	54| 0.00879	56| 0.00853	57| 0.00838	58| 0.00829	58| 0.00823	62| 0.0082	33| 0.01458	27| 0.01734	25| 0.01906	24| 0.02005	23| 0.02057	23| 0.02082
Peru	33| 0.01403	34| 0.01336	36| 0.01286	37| 0.01249	38| 0.01224	38| 0.01207	41| 0.01133	46| 0.0101	49| 0.00952	51| 0.00936	49| 0.00944	48| 0.00966
Antigua and Barbuda	41| 0.00395	52| 0.00306	65| 0.00247	77| 0.00208	89| 0.0018	103| 0.0016	13| 0.01193	12| 0.01284	12| 0.01332	12| 0.01354	12| 0.0136	12| 0.01358
Bahamas	72| 0.00165	69| 0.00172	71| 0.00168	74| 0.0016	78| 0.00151	85| 0.00144	8| 0.01421	9| 0.01307	9| 0.01227	9| 0.01275	8| 0.01396	8| 0.01505
Barbados	50| 0.00352	66| 0.00268	76| 0.00242	71| 0.00252	66| 0.0027	62| 0.00287	20| 0.00895	20| 0.00864	19| 0.00916	18| 0.00993	16| 0.01065	16| 0.0112
Belize	32| 0.0128	35| 0.01152	39| 0.01029	44| 0.0092	49| 0.00828	57| 0.00753	44| 0.00915	41| 0.00987	39| 0.01045	39| 0.01039	40| 0.01008	41| 0.00997
Bermuda	69| 0.00257	73| 0.00256	67| 0.00266	64| 0.00277	62| 0.00286	61| 0.00293	19| 0.00949	18| 0.00991	17| 0.01025	17| 0.01048	17| 0.01063	16| 0.01074
Cuba	55| 0.00728	57| 0.00705	59| 0.00686	62| 0.00677	59| 0.00678	59| 0.00685	27| 0.01496	27| 0.01506	25| 0.01582	24| 0.01655	23| 0.01703	23| 0.01727
Dominica	59| 0.0034	63| 0.00321	68| 0.00298	73| 0.00276	78| 0.00258	87| 0.00242	10| 0.01952	11| 0.01838	12| 0.01698	13| 0.01571	13| 0.01493	13| 0.01477
Dominican Republic	40| 0.00683	42| 0.00647	45| 0.00609	47| 0.00575	50| 0.00546	55| 0.00522	34| 0.00785	39| 0.00698	40| 0.00686	38| 0.00709	36| 0.00745	34| 0.00785
Grenada	102| 0.00166	80| 0.00205	62| 0.00265	52| 0.00315	47| 0.00354	43| 0.00386	10| 0.01625	14| 0.01177	18| 0.00908	22| 0.00758	24| 0.00679	26| 0.00641
Guyana	43| 0.00241	57| 0.00182	73| 0.00142	84| 0.00131	75| 0.00139	67| 0.00156	12| 0.00839	22| 0.00468	23| 0.00459	17| 0.00596	14| 0.00719	13| 0.00809
Haiti	40| 0.00235	52| 0.00178	62| 0.00151	68| 0.00138	72| 0.00134	70| 0.00135	25| 0.00374	23| 0.00402	22| 0.00418	22| 0.00426	22| 0.00429	22| 0.0043
Jamaica	57| 0.00634	46| 0.0073	43| 0.0078	42| 0.008	42| 0.00802	43| 0.00792	28| 0.01182	36| 0.00945	40| 0.00846	41| 0.00818	41| 0.00818	41| 0.00828
Puerto Rico	45| 0.01009	47| 0.0097	48| 0.00941	49| 0.00918	50| 0.00899	55| 0.00882	36| 0.01246	37| 0.01206	37| 0.01228	35| 0.013	32| 0.01411	29| 0.01539
Saint Kitts and Nevis	97| 0.00138	76| 0.00171	69| 0.00188	68| 0.00191	69| 0.00188	70| 0.00184	10| 0.01232	9| 0.01386	8| 0.01493	8| 0.01561	8| 0.016	8| 0.01617
Saint Lucia	51| 0.00277	83| 0.00169	98| 0.0015	77| 0.00182	63| 0.00224	54| 0.0026	8| 0.01772	10| 0.01345	12| 0.01123	14| 0.01008	15| 0.00945	15| 0.0091
Saint Vincent and the Grenadines	31| 0.00445	38| 0.00363	46| 0.00298	56| 0.00246	68| 0.00204	84| 0.00171	15| 0.00929	22| 0.0061	31| 0.00442	36| 0.0038	37| 0.00371	36| 0.00382
Suriname	58| 0.00265	55| 0.00278	55| 0.0028	56| 0.00276	57| 0.00268	62| 0.00258	21| 0.00723	26| 0.00592	28| 0.00538	29| 0.00534	27| 0.00558	26| 0.00596

Trinidad and Tobago	66| 0.00421	66| 0.00427	66| 0.00426	66| 0.00421	67| 0.00415	72| 0.00408	17| 0.01585	16| 0.01699	16| 0.01741	16| 0.01748	16| 0.01747	16| 0.01752
United States Virgin Islands	64| 0.00372	66| 0.00359	67| 0.00352	71| 0.00351	67| 0.00353	66| 0.00357	18| 0.01306	17| 0.01358	17| 0.01404	16| 0.01445	16| 0.01485	15| 0.01522
Colombia	48| 0.00556	53| 0.00511	55| 0.00487	57| 0.00473	58| 0.00465	62| 0.00462	35| 0.00777	31| 0.00858	27| 0.00989	25| 0.01057	25| 0.01085	24| 0.011
Costa Rica	51| 0.00346	47| 0.00351	46| 0.00355	46| 0.00358	46| 0.0036	46| 0.00361	25| 0.00657	35| 0.00463	41| 0.004	41| 0.00397	39| 0.00417	37| 0.00444
El Salvador	48| 0.00426	57| 0.00356	60| 0.00337	61| 0.00334	61| 0.00333	64| 0.00333	23| 0.00867	24| 0.00828	24| 0.00836	25| 0.00811	26| 0.0077	27| 0.0076
Guatemala	31| 0.00703	41| 0.00537	54| 0.00413	68| 0.00327	82| 0.00271	95| 0.00241	23| 0.00943	23| 0.00954	22| 0.0101	21| 0.01061	20| 0.01097	20| 0.01125
Honduras	47| 0.00316	54| 0.00279	59| 0.00268	55| 0.00273	53| 0.00284	50| 0.00297	20| 0.00737	29| 0.00515	35| 0.00428	36| 0.00416	33| 0.00459	29| 0.00516
Mexico	63| 0.00452	63| 0.00455	65| 0.0044	68| 0.00421	71| 0.00404	75| 0.00389	16| 0.01742	17| 0.01635	17| 0.01665	16| 0.01752	15| 0.01868	14| 0.02007
Nicaragua	58| 0.00297	59| 0.00293	58| 0.00298	57| 0.00303	56| 0.00306	56| 0.00307	30| 0.00579	28| 0.00614	26| 0.00667	25| 0.00695	24| 0.00716	23| 0.00754
Panama	114| 0.00089	97| 0.00103	80| 0.00125	68| 0.00147	59| 0.00169	53| 0.00188	6| 0.01688	5| 0.01795	5| 0.0191	5| 0.02029	4| 0.02158	4| 0.02299
Venezuela (Bolivarian Republic of)	69| 0.00886	78| 0.00781	84| 0.00723	88| 0.00693	90| 0.00679	91| 0.00673	0| Inf	0| Inf	0| Inf	0| Inf	0| Inf	0| Inf
Brazil	44| 0.0099	39| 0.01118	36| 0.012	35| 0.01245	34| 0.01266	34| 0.01271	46| 0.00942	53| 0.00898	47| 0.00916	46| 0.00944	44| 0.00979	42| 0.01022
Acre	38| 0.01449	34| 0.01624	32| 0.01724	31| 0.0177	31| 0.01783	31| 0.01778	39| 0.01426	45| 0.01222	50| 0.011	54| 0.01028	56| 0.00983	59| 0.00955
Alagoas	46| 0.01088	42| 0.01179	41| 0.01221	40| 0.0123	41| 0.01219	41| 0.01199	30| 0.01679	36| 0.01365	42| 0.01177	46| 0.01091	49| 0.01071	46| 0.01087
Amapá	43| 0.01018	41| 0.01056	40| 0.01086	39| 0.01105	39| 0.01111	39| 0.01108	43| 0.01014	47| 0.00981	43| 0.01	42| 0.01024	42| 0.01035	42| 0.01032
Amazonas	55| 0.00998	48| 0.01097	45| 0.01157	44| 0.01183	44| 0.01187	45| 0.01177	32| 0.01649	35| 0.0151	38| 0.01399	39| 0.01358	38| 0.01377	37| 0.01432
Bahia	41| 0.01306	37| 0.0143	36| 0.0149	35| 0.0151	35| 0.01506	36| 0.0149	35| 0.01513	42| 0.01259	47| 0.01122	50| 0.0106	55| 0.01038	51| 0.01039
Ceará	35| 0.01657	33| 0.01729	33| 0.0175	33| 0.0174	34| 0.01713	34| 0.01678	36| 0.01617	42| 0.01369	48| 0.01203	53| 0.0109	57| 0.0101	62| 0.00954
Distrito Federal	66| 0.00722	60| 0.00767	58| 0.00799	56| 0.00818	56| 0.00824	56| 0.00823	21| 0.02206	23| 0.02029	25| 0.01868	26| 0.01763	27| 0.01727	26| 0.01745
Espírito Santo	43| 0.01025	39| 0.01139	36| 0.01224	35| 0.01278	34| 0.0131	33| 0.01327	40| 0.01113	46| 0.00956	48| 0.00912	51| 0.00909	48| 0.00924	47| 0.0095
Goiás	47| 0.00864	41| 0.00988	37| 0.01092	35| 0.01162	34| 0.01206	33| 0.01232	36| 0.01131	43| 0.0095	47| 0.00874	48| 0.00849	48| 0.00843	51| 0.00843
Maranhão	39| 0.0168	36| 0.01829	35| 0.01887	35| 0.01889	35| 0.01863	36| 0.01821	37| 0.01763	41| 0.01594	46| 0.01432	50| 0.01302	54| 0.01221	56| 0.01188
Mato Grosso	52| 0.00957	43| 0.01116	39| 0.01224	37| 0.01289	36| 0.01326	36| 0.01345	36| 0.01348	41| 0.01182	44| 0.01098	45| 0.01062	46| 0.01053	45| 0.01062
Mato Grosso do Sul	44| 0.00986	40| 0.0109	37| 0.01172	35| 0.01227	34| 0.01259	34| 0.01275	46| 0.00948	50| 0.00925	46| 0.00938	45| 0.00956	45| 0.00971	44| 0.00982
Minas Gerais	53| 0.00785	44| 0.00913	40| 0.01006	38| 0.01065	37| 0.01099	36| 0.01117	40| 0.01	43| 0.00939	43| 0.00933	43| 0.00944	42| 0.00964	41| 0.00991
Pará	56| 0.01176	48| 0.01282	46| 0.01341	46| 0.01361	46| 0.01356	46| 0.01338	25| 0.02466	30| 0.02079	35| 0.01779	39| 0.01593	41| 0.01506	42| 0.01491
Paraíba	40| 0.0158	36| 0.01743	34| 0.01835	33| 0.01876	33| 0.01886	33| 0.01878	38| 0.01671	43| 0.01476	47| 0.0134	51| 0.01245	53| 0.01179	59| 0.01136
Paraná	54| 0.00762	47| 0.00834	44| 0.00889	43| 0.00925	42| 0.00946	41| 0.00955	31| 0.01272	36| 0.01081	39| 0.00996	41| 0.00966	41| 0.00962	41| 0.0097
Pernambuco	54| 0.01054	47| 0.01132	45| 0.0118	44| 0.01199	44| 0.01201	45| 0.0119	27| 0.01931	32| 0.01636	37| 0.01447	40| 0.01333	42| 0.01268	43| 0.01237

Piaui	39| 0.01268	35| 0.01414	34| 0.01484	33| 0.01506	33| 0.01502	34| 0.01482	36| 0.01401	41| 0.01224	47| 0.01074	52| 0.00963	56| 0.00898	60| 0.00881
Rio de Janeiro	45| 0.00905	41| 0.01	38| 0.01068	37| 0.01111	36| 0.01135	36| 0.01146	41| 0.01009	43| 0.00963	44| 0.00936	45| 0.00916	46| 0.00899	48| 0.00885
Rio Grande do Norte	43| 0.01153	40| 0.01213	39| 0.01244	39| 0.01254	39| 0.01252	40| 0.01242	33| 0.01476	39| 0.01243	43| 0.01129	46| 0.01073	47| 0.01043	52| 0.01027
Rio Grande do Sul	54| 0.00827	49| 0.00864	47| 0.00904	45| 0.00932	45| 0.0095	44| 0.00959	36| 0.01178	37| 0.01145	37| 0.01147	36| 0.01166	35| 0.01191	35| 0.01218
Rondônia	51| 0.00966	43| 0.01107	40| 0.01187	39| 0.01224	39| 0.01234	39| 0.0123	37| 0.01299	41| 0.01174	43| 0.01123	43| 0.01111	43| 0.01119	42| 0.01135
Roraima	38| 0.01289	35| 0.01387	34| 0.01431	34| 0.0144	34| 0.01431	34| 0.01413	45| 0.01095	47| 0.01043	48| 0.01013	49| 0.00996	49| 0.00988	53| 0.00986
Santa Catarina	64| 0.00714	54| 0.0081	50| 0.0088	47| 0.00927	46| 0.00957	45| 0.00975	31| 0.01412	33| 0.01343	33| 0.01324	33| 0.01329	32| 0.01344	32| 0.01363
São Paulo	38| 0.01453	35| 0.01575	33| 0.01654	32| 0.017	32| 0.01723	32| 0.01729	41| 0.01342	46| 0.01199	49| 0.01114	52| 0.01061	53| 0.01028	57| 0.01007
Sergipe	43| 0.01147	40| 0.01253	38| 0.01305	38| 0.01321	38| 0.01316	38| 0.01299	40| 0.01255	43| 0.01156	45| 0.01109	47| 0.01092	45| 0.01093	45| 0.01106
Tocantins	44| 0.01036	40| 0.01125	39| 0.01159	39| 0.0116	40| 0.01143	41| 0.01119	27| 0.01658	37| 0.01216	45| 0.0102	47| 0.0096	48| 0.00946	53| 0.00942
Paraguay	39| 0.01272	42| 0.0118	45| 0.01088	49| 0.01006	53| 0.00937	58| 0.00881	42| 0.01175	37| 0.0132	35| 0.01422	34| 0.0146	33| 0.01477	33| 0.01499
Afghanistan	43| 0.00904	46| 0.00837	49| 0.00794	50| 0.00766	52| 0.00745	57| 0.00729	28| 0.01351	34| 0.0112	37| 0.01044	36| 0.01056	35| 0.01101	33| 0.01153
Algeria	41| 0.01976	41| 0.01977	41| 0.01978	41| 0.01977	41| 0.01972	41| 0.01963	31| 0.02558	38| 0.02144	42| 0.01901	46| 0.0177	47| 0.01704	50| 0.01677
Bahrain	48| 0.01885	51| 0.01772	54| 0.01679	56| 0.01603	59| 0.0154	63| 0.01488	28| 0.03212	28| 0.03265	27| 0.03271	28| 0.03228	29| 0.03149	29| 0.0305
Egypt	57| 0.01512	64| 0.01351	69| 0.01246	73| 0.01178	76| 0.01132	83| 0.01097	13| 0.06568	13| 0.06495	13| 0.06475	13| 0.06489	13| 0.06542	13| 0.06794
Iran (Islamic Republic of)	50| 0.01862	50| 0.0187	50| 0.01858	51| 0.01832	52| 0.01799	57| 0.01762	26| 0.03562	30| 0.03133	32| 0.02906	33| 0.02789	34| 0.02723	35| 0.02677
Iraq	50| 0.01085	54| 0.01006	56| 0.00974	60| 0.00965	56| 0.00965	56| 0.00968	40| 0.01337	32| 0.01673	27| 0.01992	24| 0.02219	23| 0.02361	22| 0.02436
Jordan	48| 0.01346	49| 0.01313	51| 0.01282	52| 0.01256	52| 0.01235	57| 0.01218	36| 0.01781	35| 0.01839	33| 0.01952	31| 0.02087	29| 0.02223	27| 0.02345
Kuwait	52| 0.01886	46| 0.02004	45| 0.02051	45| 0.0206	45| 0.0205	45| 0.02032	40| 0.02302	37| 0.02528	36| 0.02599	36| 0.02581	36| 0.02537	37| 0.02498
Lebanon	39| 0.02735	42| 0.02566	44| 0.02434	46| 0.02332	48| 0.02255	52| 0.02194	39| 0.0278	40| 0.02728	39| 0.02765	38| 0.0284	37| 0.02928	36| 0.03018
Libya	54| 0.01836	58| 0.01737	60| 0.01674	61| 0.01635	62| 0.01612	66| 0.01594	22| 0.04562	23| 0.04403	23| 0.04314	23| 0.04236	24| 0.04156	24| 0.0408
Morocco	55| 0.00647	62| 0.00608	58| 0.00611	58| 0.0062	57| 0.00627	57| 0.00631	19| 0.01827	22| 0.01598	25| 0.01428	27| 0.01304	29| 0.01214	31| 0.01148
Oman	52| 0.02214	56| 0.02035	60| 0.01927	61| 0.01869	63| 0.01835	68| 0.01815	20| 0.0562	22| 0.05178	23| 0.04896	24| 0.04682	25| 0.04496	26| 0.0433
Palestine	33| 0.01998	35| 0.0191	36| 0.01845	37| 0.01801	37| 0.01775	38| 0.01759	51| 0.01312	54| 0.01263	49| 0.0137	45| 0.01485	43| 0.01559	42| 0.0159
Qatar	45| 0.01837	49| 0.01697	52| 0.0159	55| 0.0151	57| 0.01448	61| 0.01398	31| 0.02643	29| 0.02841	29| 0.02827	30| 0.0274	31| 0.02672	31| 0.02665
Saudi Arabia	50| 0.03141	50| 0.0311	55| 0.03107	50| 0.03114	50| 0.03121	50| 0.03123	34| 0.04602	33| 0.0469	32| 0.04808	32| 0.04883	32| 0.04902	32| 0.04872
Sudan	31| 0.01759	34| 0.01635	36| 0.01541	38| 0.0147	39| 0.01417	40| 0.01375	44| 0.01259	52| 0.01068	56| 0.01046	48| 0.01155	43| 0.01289	39| 0.01402
Syrian Arab Republic	53| 0.02365	54| 0.02311	55| 0.02286	55| 0.02279	55| 0.02275	60| 0.02271	28| 0.0452	28| 0.04486	28| 0.04463	28| 0.0445	28| 0.04444	28| 0.04443
Tunisia	39| 0.01611	44| 0.01427	49| 0.0128	54| 0.01165	59| 0.01073	68| 0.00999	21| 0.02978	26| 0.02386	31| 0.02042	34| 0.01843	37| 0.01721	38| 0.01641

Turkey	48| 0.02367	44| 0.02481	43| 0.02556	42| 0.02601	42| 0.02625	42| 0.02634	43| 0.02558	43| 0.02569	41| 0.0267	39| 0.02798	37| 0.02925	36| 0.03035
United Arab Emirates	57| 0.01463	62| 0.01358	65| 0.01298	66| 0.01265	67| 0.01247	72| 0.01235	18| 0.04725	18| 0.04534	19| 0.04397	19| 0.04384	19| 0.04457	18| 0.04568
Yemen	39| 0.02494	39| 0.02476	39| 0.02442	40| 0.02401	41| 0.02357	42| 0.02312	47| 0.02143	44| 0.02205	43| 0.02251	42| 0.02281	42| 0.02297	42| 0.02303
Bangladesh	64| 0.00723	57| 0.00783	55| 0.0081	55| 0.00813	56| 0.00803	57| 0.00787	23| 0.01976	24| 0.01891	25| 0.01797	26| 0.01696	28| 0.01596	30| 0.01502
Bhutan	32| 0.00821	34| 0.00788	34| 0.00767	35| 0.00753	36| 0.00741	36| 0.00732	49| 0.00538	54| 0.00526	50| 0.00534	48| 0.0055	47| 0.00567	45| 0.00584
India	54| 0.0115	53| 0.01156	54| 0.01146	55| 0.01129	56| 0.0111	59| 0.01091	20| 0.03076	23| 0.02622	27| 0.02278	30| 0.02029	33| 0.0185	36| 0.0172
Andhra Pradesh	48| 0.0174	45| 0.01867	43| 0.01936	42| 0.0197	42| 0.01984	42| 0.01987	28| 0.02953	33| 0.02523	38| 0.02201	42| 0.01972	46| 0.01811	51| 0.01699
Arunachal Pradesh	41| 0.01782	44| 0.01657	47| 0.0155	50| 0.01457	53| 0.01377	59| 0.01306	28| 0.02549	32| 0.02276	35| 0.02101	36| 0.01997	37| 0.01938	38| 0.01905
Assam	70| 0.00847	64| 0.00889	61| 0.00938	59| 0.00975	57| 0.01	56| 0.01017	17| 0.03378	19| 0.02944	22| 0.0263	24| 0.02416	25| 0.02273	26| 0.02178
Bihar	55| 0.00731	58| 0.00695	61| 0.00664	63| 0.00637	66| 0.00615	71| 0.00598	15| 0.02635	18| 0.02199	21| 0.01922	23| 0.01757	24| 0.01662	25| 0.01611
Chhattisgarh	52| 0.01008	52| 0.01011	52| 0.01011	53| 0.01003	53| 0.00992	58| 0.00979	18| 0.02872	22| 0.02332	27| 0.01915	33| 0.01612	38| 0.014	42| 0.01257
Delhi	63| 0.00798	56| 0.00865	52| 0.00938	48| 0.01004	46| 0.01059	44| 0.01104	35| 0.01404	35| 0.01379	34| 0.01427	32| 0.01533	29| 0.0169	26| 0.01876
Goa	40| 0.01053	44| 0.00968	48| 0.00893	52| 0.00827	55| 0.00772	62| 0.00725	30| 0.01436	33| 0.01292	34| 0.01235	35| 0.01225	34| 0.01245	33| 0.01282
Gujarat	53| 0.00986	54| 0.00962	56| 0.00935	57| 0.00908	59| 0.00885	64| 0.00865	25| 0.02076	26| 0.02033	26| 0.02001	26| 0.01969	27| 0.01935	27| 0.01898
Haryana	51| 0.01322	54| 0.01255	57| 0.01195	59| 0.01146	61| 0.01107	65| 0.01076	24| 0.02833	25| 0.0268	26| 0.02619	26| 0.02596	26| 0.02582	26| 0.02568
Himachal Pradesh	51| 0.00707	54| 0.00668	56| 0.00641	58| 0.00622	60| 0.00608	65| 0.00598	36| 0.00993	30| 0.01204	26| 0.014	23| 0.01555	21| 0.01677	20| 0.01773
Jammu and Kashmir	44| 0.00805	47| 0.00756	49| 0.00724	50| 0.00702	51| 0.00689	54| 0.00681	35| 0.01012	36| 0.00985	35| 0.01017	34| 0.01053	33| 0.01081	32| 0.01105
Jharkhand	48| 0.00672	51| 0.00634	53| 0.00616	54| 0.00603	55| 0.00592	59| 0.00582	18| 0.01755	23| 0.01398	28| 0.01137	34| 0.00965	37| 0.00864	40| 0.00815
Karnataka	50| 0.00827	53| 0.00786	56| 0.00743	59| 0.00706	61| 0.00675	66| 0.00649	19| 0.02195	22| 0.01893	25| 0.01666	27| 0.01502	30| 0.01384	32| 0.01298
Kerala	39| 0.01545	43| 0.01413	46| 0.013	50| 0.01206	53| 0.01129	60| 0.01066	48| 0.01251	39| 0.01543	34| 0.01789	31| 0.0196	29| 0.02075	28| 0.02155
Madhya Pradesh	63| 0.01282	59| 0.01304	58| 0.01322	57| 0.01332	57| 0.01335	57| 0.01335	18| 0.04154	21| 0.03633	24| 0.03214	26| 0.0289	29| 0.02642	31| 0.02451
Maharashtra	52| 0.02091	51| 0.02119	51| 0.02118	52| 0.02101	52| 0.02079	55| 0.02054	34| 0.03172	32| 0.03352	31| 0.03489	30| 0.03576	30| 0.03619	30| 0.03631
Manipur	38| 0.00976	45| 0.00818	53| 0.00706	59| 0.00627	65| 0.0057	74| 0.00528	29| 0.01281	28| 0.0131	28| 0.01336	27| 0.01351	27| 0.01356	27| 0.01356
Meghalaya	47| 0.01266	49| 0.01226	51| 0.01185	52| 0.01148	54| 0.01115	57| 0.01087	28| 0.0216	30| 0.01986	32| 0.01888	33| 0.0183	33| 0.01793	34| 0.01764
Mizoram	48| 0.01011	53| 0.00912	58| 0.00838	62| 0.0078	66| 0.00735	72| 0.00699	29| 0.01699	25| 0.01947	23| 0.02128	22| 0.02246	21| 0.02315	21| 0.0235
Nagaland	34| 0.01454	40| 0.01245	46| 0.01086	52| 0.00966	58| 0.00874	66| 0.00802	40| 0.01266	36| 0.01396	34| 0.01494	32| 0.01558	31| 0.01599	31| 0.01626
Odisha	42| 0.01086	42| 0.01089	43| 0.01081	43| 0.01067	44| 0.01052	44| 0.01037	24| 0.01945	30| 0.01526	37| 0.01228	44| 0.01036	50| 0.00924	57| 0.00867
Punjab	46| 0.01489	51| 0.01339	56| 0.0123	60| 0.0115	63| 0.01089	69| 0.01042	30| 0.02308	27| 0.02497	26| 0.02668	25| 0.0279	24| 0.02866	23| 0.02909
Rajasthan	58| 0.00884	57| 0.00891	57| 0.00898	57| 0.00898	57| 0.00892	61| 0.00884	17| 0.03041	20| 0.02564	23| 0.02171	27| 0.01861	31| 0.01623	35| 0.01445

Sikkim	41| 0.02453	44| 0.02287	47| 0.02121	51| 0.01969	55| 0.01833	61| 0.01712	32| 0.03112	33| 0.03013	34| 0.02975	34| 0.02956	34| 0.02937	34| 0.02915
Tamil Nadu	51| 0.02169	47| 0.0222	47| 0.02242	46| 0.02246	47| 0.02242	47| 0.02234	41| 0.02521	38| 0.02723	36| 0.02903	34| 0.0304	33| 0.03137	33| 0.03204
Telangana	45| 0.02383	42| 0.02522	41| 0.02592	41| 0.02621	41| 0.02627	41| 0.02619	31| 0.03486	35| 0.03031	40| 0.02685	44| 0.02434	47| 0.02253	52| 0.02122
Tripura	50| 0.00395	56| 0.0035	61| 0.0032	65| 0.00301	68| 0.0029	73| 0.00283	18| 0.01063	20| 0.00958	21| 0.00916	22| 0.00891	23| 0.00866	23| 0.0084
Union Territories other than Delhi	57| 0.0036	58| 0.00354	59| 0.00348	60| 0.00342	61| 0.00337	65| 0.00332	21| 0.00974	26| 0.00801	26| 0.00797	24| 0.00848	22| 0.00908	21| 0.00968
Uttar Pradesh	57| 0.00945	56| 0.00961	56| 0.00963	56| 0.00957	57| 0.00946	62| 0.00934	17| 0.0313	20| 0.02652	24| 0.02253	28| 0.01938	32| 0.017	35| 0.01526
Uttarakhand	47| 0.01158	50| 0.01083	53| 0.01017	56| 0.00959	59| 0.00911	65| 0.00871	27| 0.02015	29| 0.01857	29| 0.01836	29| 0.0186	28| 0.01885	28| 0.01902
West Bengal	65| 0.0074	56| 0.00782	54| 0.0081	53| 0.00828	52| 0.00841	52| 0.0085	21| 0.02119	24| 0.01812	27| 0.01599	30| 0.01456	32| 0.01359	34| 0.01293
Nepal	35| 0.00873	35| 0.00872	35| 0.00874	35| 0.00872	35| 0.00868	35| 0.00862	45| 0.00669	47| 0.00647	48| 0.00633	49| 0.00624	49| 0.00617	52| 0.00612
Pakistan	54| 0.00777	46| 0.00873	43| 0.00928	42| 0.00956	42| 0.00967	42| 0.00968	32| 0.0126	36| 0.01119	39| 0.01035	41| 0.00991	41| 0.0097	42| 0.00963
China	58| 0.00328	61| 0.00314	63| 0.00304	64| 0.00298	65| 0.00295	68| 0.00295	17| 0.01095	20| 0.00954	21| 0.00887	22| 0.00863	21| 0.00884	20| 0.00949
Anhui	36| 0.00989	38| 0.00943	40| 0.00908	41| 0.0088	42| 0.00859	43| 0.00842	40| 0.00905	43| 0.00834	45| 0.00817	44| 0.00817	44| 0.0082	44| 0.00824
Beijing	62| 0.00562	63| 0.00555	66| 0.00553	63| 0.00554	62| 0.00557	62| 0.00559	22| 0.01563	20| 0.01683	20| 0.01707	20| 0.017	20| 0.01694	20| 0.01698
Chongqing	53| 0.00498	53| 0.0049	58| 0.00489	53| 0.00492	53| 0.00496	52| 0.00502	29| 0.00896	30| 0.0086	30| 0.00863	30| 0.00863	30| 0.0087	29| 0.00898
Fujian	55| 0.00525	56| 0.00511	57| 0.00505	60| 0.00504	57| 0.00506	57| 0.00509	22| 0.01304	26| 0.01115	28| 0.01037	28| 0.01018	28| 0.0104	26| 0.01094
Gansu	40| 0.0077	42| 0.00738	44| 0.0071	45| 0.00688	47| 0.00671	51| 0.00658	28| 0.01114	34| 0.00913	39| 0.00801	42| 0.00744	44| 0.00716	44| 0.00702
Guangdong	58| 0.00852	52| 0.0089	50| 0.00925	49| 0.00957	47| 0.00986	46| 0.01011	35| 0.01324	35| 0.0132	34| 0.01359	33| 0.01412	31| 0.01473	30| 0.01535
Guangxi	51| 0.00646	47| 0.00651	46| 0.00656	46| 0.00662	45| 0.00668	45| 0.00673	30| 0.01004	34| 0.0088	37| 0.00809	39| 0.0077	40| 0.00755	40| 0.00761
Guizhou	43| 0.0064	45| 0.00611	47| 0.00591	48| 0.00577	49| 0.00568	51| 0.00561	25| 0.01118	30| 0.00907	36| 0.00771	40| 0.00692	42| 0.00649	44| 0.00628
Hainan	53| 0.00651	47| 0.0068	45| 0.00705	44| 0.00725	43| 0.00743	42| 0.00758	31| 0.01032	35| 0.00903	39| 0.00831	41| 0.0079	41| 0.00784	39| 0.00817
Hebei	57| 0.00544	58| 0.00532	60| 0.00521	61| 0.00512	61| 0.00506	64| 0.00502	21| 0.01474	23| 0.01338	24| 0.01284	24| 0.01272	24| 0.013	23| 0.01364
Heilongjiang	56| 0.00648	55| 0.00652	55| 0.00653	55| 0.00652	56| 0.00649	59| 0.00645	26| 0.01403	27| 0.01309	28| 0.01269	29| 0.01257	28| 0.01288	26| 0.0136
Henan	44| 0.00945	46| 0.00918	47| 0.00897	48| 0.0088	48| 0.00868	52| 0.00857	31| 0.01341	35| 0.01207	37| 0.01138	38| 0.01109	38| 0.01115	36| 0.01155
Hong Kong Special Administrative Region of China	50| 0.00475	51| 0.00468	52| 0.00459	53| 0.00449	54| 0.0044	57| 0.00431	28| 0.00829	28| 0.00834	30| 0.00791	31| 0.0075	33| 0.00721	33| 0.00705
Hubei	50| 0.00565	52| 0.00549	53| 0.0054	53| 0.00536	56| 0.00535	53| 0.00537	27| 0.01059	30| 0.00933	32| 0.00875	33| 0.00861	32| 0.00895	29| 0.00975
Hunan	44| 0.00707	46| 0.00688	47| 0.00673	48| 0.0066	48| 0.0065	51| 0.00642	30| 0.01048	34| 0.00915	37| 0.00846	38| 0.00816	39| 0.00813	38| 0.00832
Inner Mongolia	55| 0.00566	60| 0.00524	63| 0.005	64| 0.00488	65| 0.00483	68| 0.00482	21| 0.01469	22| 0.01441	22| 0.01426	21| 0.01448	20| 0.01522	19| 0.01637
Jiangsu	60| 0.00458	61| 0.00452	62| 0.00448	65| 0.00448	61| 0.0045	61| 0.00454	22| 0.01235	23| 0.01196	23| 0.01209	22| 0.01252	21| 0.01323	19| 0.01412
Jiangxi	49| 0.006	54| 0.00595	50| 0.00596	49| 0.006	49| 0.00605	49| 0.0061	27| 0.01076	31| 0.00944	34| 0.0087	36| 0.00832	36| 0.00817	36| 0.00818

Jilin	53| 0.00575	54| 0.00564	56| 0.0055	57| 0.00536	58| 0.00523	62| 0.00513	27| 0.01139	28| 0.01096	28| 0.0109	27| 0.01115	26| 0.01183	24| 0.01285
Liaoning	48| 0.01034	47| 0.01039	47| 0.0104	47| 0.01037	48| 0.01033	53| 0.01028	36| 0.01353	36| 0.01346	36| 0.01361	35| 0.01389	34| 0.01427	33| 0.01471
Macao Special Administrative Region of China	48| 0.00345	51| 0.00323	53| 0.00311	54| 0.00304	55| 0.003	58| 0.00298	27| 0.00605	28| 0.0058	30| 0.00547	31| 0.00522	32| 0.00506	33| 0.00496
Ningxia	46| 0.00611	51| 0.00556	54| 0.00524	55| 0.0051	58| 0.00507	55| 0.0051	23| 0.01215	27| 0.01028	31| 0.00915	33| 0.00856	34| 0.00838	33| 0.00853
Qinghai	55| 0.00403	56| 0.00397	58| 0.00396	56| 0.00398	56| 0.004	55| 0.00403	20| 0.01109	24| 0.00914	28| 0.00804	30| 0.00749	31| 0.00723	31| 0.00713
Shaanxi	48| 0.00674	52| 0.00635	54| 0.00606	56| 0.00587	57| 0.00575	61| 0.00568	27| 0.01217	29| 0.01119	30| 0.01074	30| 0.01071	29| 0.01115	27| 0.01205
Shandong	67| 0.00435	64| 0.00437	64| 0.00438	64| 0.00439	64| 0.0044	63| 0.00441	18| 0.01579	19| 0.01439	20| 0.01395	20| 0.01403	19| 0.01456	18| 0.0154
Shanghai	57| 0.00632	61| 0.00599	62| 0.00588	65| 0.00587	61| 0.00591	61| 0.00596	23| 0.01545	22| 0.01633	22| 0.01647	22| 0.01636	22| 0.01628	22| 0.01628
Shanxi	63| 0.00429	64| 0.00424	66| 0.00422	64| 0.00423	63| 0.00425	63| 0.00428	18| 0.01505	19| 0.01373	20| 0.01312	21| 0.01302	20| 0.0134	19| 0.01417
Sichuan	48| 0.0055	48| 0.00547	48| 0.00544	49| 0.00542	49| 0.0054	51| 0.00539	26| 0.01	32| 0.00829	35| 0.00744	37| 0.00707	38| 0.00691	39| 0.00682
Tianjin	65| 0.00449	67| 0.0044	67| 0.00437	70| 0.00437	67| 0.00438	67| 0.00439	19| 0.01532	17| 0.01741	16| 0.01852	15| 0.01894	15| 0.019	15| 0.01888
Tibet	44| 0.0056	45| 0.00552	45| 0.00545	46| 0.00538	46| 0.00531	50| 0.00525	39| 0.00633	39| 0.00633	38| 0.00644	37| 0.00659	36| 0.00677	35| 0.00694
Xinjiang	60| 0.00496	57| 0.00501	56| 0.00508	55| 0.00516	54| 0.00523	54| 0.0053	24| 0.01167	27| 0.01043	28| 0.01003	29| 0.00979	29| 0.0099	27| 0.0105
Yunnan	43| 0.00695	46| 0.00641	49| 0.00607	51| 0.00586	52| 0.00572	54| 0.00564	27| 0.01082	32| 0.00928	35| 0.0085	36| 0.00812	37| 0.00794	38| 0.00785
Zhejiang	61| 0.00628	57| 0.00629	57| 0.00633	56| 0.0064	55| 0.00649	54| 0.00658	26| 0.01355	28| 0.01264	28| 0.01258	27| 0.01296	26| 0.01361	25| 0.01441
Democratic People's Republic of Korea	40| 0.00691	46| 0.00598	51| 0.00542	54| 0.00507	57| 0.00481	62| 0.00461	31| 0.00887	35| 0.00779	35| 0.00794	32| 0.0086	29| 0.00931	28| 0.00994
Taiwan (Province of China)	60| 0.00416	61| 0.00414	62| 0.00407	63| 0.00398	65| 0.0039	69| 0.00383	19| 0.01305	19| 0.01291	20| 0.01255	20| 0.01226	21| 0.01209	21| 0.01201
American Samoa	88| 0.00189	75| 0.0021	65| 0.0024	58| 0.00272	52| 0.00301	48| 0.00326	19| 0.00805	19| 0.00813	19| 0.00813	19| 0.00808	19| 0.00802	19| 0.00798
Cook Islands	53| 0.00353	60| 0.00312	67| 0.00279	74| 0.00252	81| 0.00231	90| 0.00213	13| 0.01394	12| 0.01485	12| 0.0151	12| 0.01492	13| 0.01456	13| 0.01425
Fiji	60| 0.00389	62| 0.00389	58| 0.00404	56| 0.00421	54| 0.00435	53| 0.00445	19| 0.01223	23| 0.00998	27| 0.00872	29| 0.00806	30| 0.00785	29| 0.008
Guam	58| 0.00496	59| 0.00488	61| 0.00471	64| 0.00451	66| 0.00435	72| 0.00422	20| 0.01437	19| 0.01522	19| 0.01495	20| 0.01413	21| 0.0134	21| 0.01331
Kiribati	44| 0.00742	44| 0.00737	45| 0.00724	46| 0.00708	47| 0.00691	50| 0.00675	34| 0.00963	36| 0.00899	37| 0.00868	38| 0.00845	39| 0.00825	40| 0.00811
Marshall Islands	36| 0.01063	36| 0.01055	37| 0.01041	37| 0.01024	38| 0.01004	39| 0.00984	36| 0.01073	43| 0.00898	47| 0.00818	49| 0.00782	50| 0.00764	52| 0.00755
Micronesia (Federated States of)	53| 0.00362	60| 0.00321	63| 0.00304	67| 0.00301	63| 0.00304	62| 0.0031	21| 0.00907	23| 0.00829	23| 0.00824	22| 0.0085	22| 0.00879	21| 0.00902
Nauru	70| 0.00455	78| 0.00408	86| 0.00391	80| 0.00398	76| 0.00421	71| 0.00449	4| 0.06881	7| 0.04353	7| 0.04335	7| 0.04591	7| 0.04686	7| 0.04681
Niue	69| 0.00281	61| 0.00309	57| 0.00329	55| 0.00343	53| 0.00351	53| 0.00355	28| 0.00671	28| 0.00669	26| 0.00706	25| 0.00756	23| 0.00803	22| 0.00839
Northern Mariana Islands	64| 0.00318	69| 0.00294	73| 0.00276	77| 0.00263	80| 0.00252	86| 0.00244	8| 0.02582	8| 0.02388	9| 0.02229	9| 0.02155	9| 0.02189	8| 0.02335
Palau	64| 0.0043	59| 0.00456	56| 0.00476	55| 0.00489	54| 0.00496	54| 0.00498	25| 0.0108	27| 0.00985	29| 0.00914	28| 0.00938	26| 0.01044	23| 0.01181
Papua New Guinea	48| 0.00351	52| 0.00324	56| 0.00303	59| 0.00288	61| 0.00276	66| 0.00267	27| 0.0062	26| 0.00655	25| 0.0067	25| 0.00658	27| 0.00628	28| 0.00589

Samoa	56| 0.00385	77| 0.00289	74| 0.0029	64| 0.00336	56| 0.00383	51| 0.00425	19| 0.01137	20| 0.01051	21| 0.01008	21| 0.01007	21| 0.01034	20| 0.01074
Solomon Islands	54| 0.00455	57| 0.00441	55| 0.00442	54| 0.00454	52| 0.00471	50| 0.0049	31| 0.00789	29| 0.00839	28| 0.00867	28| 0.00856	30| 0.00815	32| 0.00767
Tokelau	96| 0.00192	66| 0.00266	55| 0.00322	49| 0.00359	46| 0.00383	44| 0.00398	26| 0.00662	31| 0.00563	28| 0.00615	24| 0.00738	19| 0.00905	16| 0.01087
Tonga	61| 0.0032	65| 0.00307	63| 0.00307	62| 0.00313	61| 0.00321	59| 0.00328	17| 0.01109	23| 0.00848	25| 0.00775	24| 0.0082	21| 0.00918	19| 0.01038
Tuvalu	54| 0.00499	61| 0.00479	56| 0.00483	56| 0.00487	55| 0.00489	56| 0.00487	28| 0.00962	29| 0.00926	30| 0.00907	28| 0.00952	25| 0.01058	22| 0.012
Vanuatu	59| 0.00375	54| 0.00391	53| 0.00401	52| 0.00408	51| 0.00413	50| 0.00417	28| 0.0074	30| 0.00705	31| 0.00684	31| 0.00675	31| 0.00677	30| 0.00688
Cambodia	40| 0.00358	39| 0.0037	38| 0.00378	38| 0.00384	37| 0.00388	37| 0.00391	58| 0.00262	52| 0.00279	46| 0.00313	41| 0.00349	38| 0.0038	36| 0.00405
Indonesia	41| 0.01441	44| 0.01341	48| 0.01237	52| 0.01139	56| 0.01054	61| 0.00981	41| 0.01427	33| 0.01789	30| 0.01964	30| 0.01977	31| 0.01904	33| 0.01806
Lao People's Democratic Republic	34| 0.00867	36| 0.00829	37| 0.00792	39| 0.00757	41| 0.00726	42| 0.00698	33| 0.00884	40| 0.00734	46| 0.00649	49| 0.00609	50| 0.00593	53| 0.00589
Malaysia	40| 0.01275	38| 0.0134	37| 0.01384	36| 0.01409	36| 0.01422	36| 0.01426	40| 0.01274	45| 0.01138	48| 0.01066	51| 0.01048	48| 0.01072	45| 0.01124
Maldives	82| 0.00297	56| 0.00416	44| 0.00534	39| 0.00604	36| 0.00643	35| 0.00664	35| 0.00668	37| 0.00626	38| 0.0062	36| 0.00648	33| 0.00718	29| 0.00817
Mauritius	43| 0.00818	45| 0.00777	48| 0.00732	51| 0.0069	54| 0.00652	59| 0.00619	28| 0.01233	32| 0.01094	34| 0.01038	35| 0.01009	35| 0.00991	36| 0.00981
Myanmar	35| 0.00892	39| 0.00786	44| 0.00711	47| 0.00657	50| 0.00617	57| 0.00587	27| 0.01134	34| 0.00908	39| 0.00789	42| 0.00735	43| 0.00718	43| 0.00721
Philippines	79| 0.00721	74| 0.00768	68| 0.00829	63| 0.00898	59| 0.00962	56| 0.01014	26| 0.02196	19| 0.02912	16| 0.0353	14| 0.03981	13| 0.04268	13| 0.04421
Seychelles	54| 0.00934	55| 0.00913	57| 0.0089	58| 0.00869	59| 0.0085	63| 0.00833	21| 0.02412	23| 0.02157	26| 0.01938	28| 0.01792	29| 0.01754	27| 0.01835
Sri Lanka	46| 0.00532	52| 0.00478	54| 0.00472	51| 0.00487	49| 0.00508	47| 0.00528	30| 0.00816	36| 0.00675	36| 0.00685	34| 0.00727	33| 0.00756	32| 0.00771
Thailand	32| 0.00828	38| 0.00713	43| 0.00629	47| 0.00567	51| 0.00521	58| 0.00488	24| 0.01095	32| 0.00842	38| 0.00702	43| 0.00627	46| 0.00583	48| 0.00557
Timor-Leste	37| 0.00389	38| 0.00386	39| 0.00372	41| 0.00352	44| 0.00328	52| 0.00305	37| 0.00392	40| 0.00364	42| 0.00346	43| 0.00337	44| 0.00335	43| 0.00336
Viet Nam	51| 0.00429	47| 0.00433	46| 0.0044	46| 0.00446	45| 0.00451	45| 0.00455	41| 0.00502	41| 0.00499	38| 0.00533	35| 0.00575	33| 0.0061	32| 0.00639
Angola	32| 0.00836	33| 0.00822	33| 0.00819	33| 0.0082	33| 0.00822	33| 0.00825	36| 0.00746	50| 0.00549	61| 0.00478	57| 0.00481	52| 0.00521	47| 0.00576
Central African Republic	35| 0.00655	40| 0.00581	42| 0.00549	43| 0.00534	44| 0.00527	47| 0.00524	39| 0.00583	41| 0.00561	42| 0.00543	43| 0.00536	43| 0.00542	41| 0.00561
Congo	68| 0.00372	74| 0.00358	70| 0.00362	68| 0.00372	66| 0.00383	64| 0.00395	11| 0.02174	13| 0.01892	15| 0.01691	16| 0.01548	17| 0.01443	18| 0.01363
Democratic Republic of the Congo	67| 0.00849	67| 0.00855	68| 0.0084	70| 0.00808	74| 0.00772	81| 0.00735	11| 0.05048	13| 0.04314	13| 0.04439	12| 0.0456	12| 0.04578	12| 0.04541
Equatorial Guinea	35| 0.02052	34| 0.02092	35| 0.02045	37| 0.01956	39| 0.01855	41| 0.01754	30| 0.02396	37| 0.01962	44| 0.01653	50| 0.01431	56| 0.01289	62| 0.01239
Gabon	46| 0.00688	50| 0.00638	53| 0.00602	55| 0.00574	57| 0.00553	61| 0.00537	55| 0.00577	36| 0.00875	26| 0.01209	22| 0.01441	20| 0.01585	19| 0.01668
Burundi	25| 0.00825	26| 0.00803	26| 0.0079	26| 0.0078	27| 0.00774	27| 0.00768	37| 0.00559	49| 0.00424	62| 0.00335	71| 0.00306	65| 0.00319	59| 0.00351
Comoros	56| 0.00236	54| 0.00238	48| 0.00263	44| 0.00289	41| 0.0031	39| 0.00326	38| 0.00339	43| 0.00296	42| 0.00303	36| 0.00355	31| 0.00414	28| 0.00462
Djibouti	107| 0.00125	69| 0.00189	50| 0.0026	42| 0.00315	37| 0.00356	34| 0.00387	14| 0.00952	21| 0.00621	31| 0.00427	35| 0.00374	32| 0.00403	28| 0.00459
Eritrea	67| 0.00297	63| 0.00303	61| 0.00312	59| 0.0032	58| 0.00326	57| 0.00331	20| 0.00956	22| 0.00862	24| 0.00788	25| 0.00764	23| 0.00803	21| 0.00893

Ethiopia	50| 0.00481	51| 0.00481	50| 0.00484	49| 0.00493	48| 0.00505	46| 0.00517	36| 0.00669	36| 0.00668	35| 0.00688	34| 0.00713	33| 0.00735	32| 0.00754
Kenya	57| 0.00611	53| 0.00631	52| 0.00644	51| 0.00652	51| 0.00658	51| 0.00662	18| 0.01814	23| 0.01467	28| 0.01192	34| 0.0099	39| 0.00855	43| 0.00775
Madagascar	26| 0.01031	26| 0.01016	27| 0.00993	28| 0.00966	28| 0.00939	29| 0.00912	37| 0.00721	49| 0.00544	59| 0.00452	68| 0.00424	63| 0.00428	60| 0.00445
Malawi	34| 0.01246	33| 0.01288	32| 0.01319	32| 0.01341	32| 0.01355	31| 0.01364	47| 0.00916	56| 0.00768	61| 0.00754	52| 0.00818	47| 0.00904	43| 0.00988
Mozambique	42| 0.00699	51| 0.00578	58| 0.00516	63| 0.00499	58| 0.00508	56| 0.00529	29| 0.01018	29| 0.0102	29| 0.01025	29| 0.01034	28| 0.01046	28| 0.0106
Rwanda	33| 0.00954	32| 0.00966	32| 0.0097	32| 0.00967	32| 0.0096	33| 0.00951	63| 0.00513	56| 0.00556	51| 0.00613	47| 0.00659	45| 0.00691	44| 0.00713
Somalia	48| 0.00627	45| 0.00637	45| 0.00644	45| 0.00647	45| 0.00648	45| 0.00648	25| 0.01176	33| 0.00888	39| 0.00733	43| 0.00672	44| 0.00663	43| 0.00678
South Sudan	47| 0.00503	48| 0.00489	49| 0.00479	53| 0.00473	50| 0.00473	49| 0.00476	34| 0.00681	34| 0.00681	34| 0.00684	34| 0.00687	34| 0.0069	34| 0.00694
Uganda	47| 0.00428	47| 0.00424	48| 0.00419	48| 0.00415	49| 0.00411	53| 0.00409	31| 0.00637	34| 0.0059	35| 0.00571	36| 0.00563	36| 0.00558	36| 0.00555
United Republic of Tanzania	48| 0.00662	57| 0.00559	61| 0.00549	55| 0.00579	52| 0.0061	50| 0.00632	26| 0.01239	27| 0.01169	29| 0.01108	30| 0.01053	32| 0.01003	33| 0.00959
Zambia	49| 0.00786	63| 0.00614	69| 0.00555	72| 0.00551	67| 0.00573	64| 0.00604	11| 0.03296	16| 0.02384	21| 0.01859	23| 0.01659	23| 0.0166	22| 0.01755
Botswana	55| 0.00401	40| 0.00511	37| 0.00557	37| 0.00563	38| 0.00549	39| 0.00525	20| 0.01013	34| 0.00604	48| 0.00435	51| 0.00404	51| 0.00405	50| 0.00414
Eswatini	49| 0.005	57| 0.00469	51| 0.00482	48| 0.00513	45| 0.00545	43| 0.00574	29| 0.00852	34| 0.00725	36| 0.00688	36| 0.00681	36| 0.0068	36| 0.00678
Lesotho	41| 0.00724	40| 0.0073	41| 0.00721	42| 0.00697	45| 0.00662	50| 0.00619	41| 0.00719	45| 0.00657	43| 0.00684	40| 0.00746	37| 0.00803	35| 0.00843
Namibia	53| 0.0065	54| 0.00637	56| 0.00615	59| 0.00584	63| 0.00548	71| 0.00509	35| 0.00989	26| 0.01308	22| 0.01529	21| 0.01648	20| 0.01698	20| 0.01711
South Africa	51| 0.00664	50| 0.00674	51| 0.0067	51| 0.00659	52| 0.00647	58| 0.00637	26| 0.01289	30| 0.01127	32| 0.01053	33| 0.01022	33| 0.01011	33| 0.01012
Zimbabwe	64| 0.01395	66| 0.01336	69| 0.01277	73| 0.01222	76| 0.01172	82| 0.01128	12| 0.07129	13| 0.06475	13| 0.06741	11| 0.07575	11| 0.08184	10| 0.08488
Benin	40| 0.00768	40| 0.0077	40| 0.00775	39| 0.00783	39| 0.00797	38| 0.00812	41| 0.00754	42| 0.00734	43| 0.00716	44| 0.00701	45| 0.0069	49| 0.00684
Burkina Faso	38| 0.00524	41| 0.00492	43| 0.00469	45| 0.00451	46| 0.00436	50| 0.00423	39| 0.00522	39| 0.00514	39| 0.0051	40| 0.00508	40| 0.00508	40| 0.00508
Cabo Verde	34| 0.01204	34| 0.01201	34| 0.01195	34| 0.01186	35| 0.01173	35| 0.0116	66| 0.00619	67| 0.00618	49| 0.00821	41| 0.00992	37| 0.01108	34| 0.01185
Cameroon	58| 0.00567	61| 0.00538	63| 0.00521	64| 0.00514	68| 0.00513	64| 0.00515	15| 0.02147	17| 0.01904	19| 0.01682	22| 0.01504	24| 0.01381	25| 0.01307
Chad	51| 0.00386	46| 0.00401	44| 0.00417	43| 0.00433	41| 0.0045	40| 0.00467	32| 0.00571	39| 0.00479	42| 0.00436	43| 0.00432	41| 0.00453	38| 0.00483
Côte d'Ivoire	58| 0.00345	61| 0.00326	64| 0.00313	66| 0.00303	68| 0.00295	72| 0.00289	22| 0.00902	21| 0.00942	19| 0.01023	18| 0.01124	16| 0.01227	15| 0.01321
Gambia	39| 0.00597	36| 0.00644	34| 0.00691	32| 0.00731	31| 0.00765	29| 0.00794	47| 0.00501	50| 0.00467	55| 0.00454	51| 0.00461	49| 0.0048	47| 0.00503
Ghana	55| 0.00912	70| 0.0071	75| 0.00693	61| 0.00815	52| 0.00966	45| 0.01102	16| 0.03159	19| 0.02543	24| 0.02104	27| 0.01854	28| 0.01762	28| 0.01774
Guinea	42| 0.00485	44| 0.00462	45| 0.00446	47| 0.00433	48| 0.00421	53| 0.00411	36| 0.00567	36| 0.00556	37| 0.00549	37| 0.00544	37| 0.00541	38| 0.0054
Guinea-Bissau	46| 0.00358	50| 0.0033	55| 0.00318	51| 0.00321	50| 0.0033	48| 0.0034	32| 0.00513	32| 0.00513	32| 0.00506	33| 0.00492	35| 0.00473	36| 0.00452
Liberia	52| 0.00828	55| 0.00822	52| 0.00835	51| 0.00844	51| 0.00846	51| 0.00842	31| 0.01389	32| 0.01344	32| 0.01351	31| 0.01367	31| 0.01377	31| 0.01378
Mali	35| 0.00564	37| 0.00542	38| 0.00521	40| 0.00504	41| 0.00492	42| 0.00481	43| 0.00468	43| 0.0047	43| 0.00465	44| 0.00454	45| 0.00442	49| 0.00431

Mauritania	55| 0.00735	48| 0.00793	45| 0.00845	43| 0.00888	41| 0.0092	40| 0.00945	26| 0.01442	32| 0.01202	37| 0.01015	42| 0.00897	45| 0.0084	46| 0.00833
Niger	52| 0.00167	50| 0.00167	49| 0.00171	47| 0.00178	43| 0.00193	39| 0.00212	33| 0.00253	36| 0.00233	37| 0.00221	38| 0.00216	38| 0.00216	38| 0.00219
Nigeria	67| 0.00696	50| 0.00905	43| 0.01058	39| 0.01167	36| 0.01245	34| 0.01303	33| 0.01347	46| 0.00988	46| 0.00975	40| 0.01125	35| 0.01296	31| 0.01445
Sao Tome and Principe	22| 0.01472	23| 0.01401	24| 0.01317	26| 0.01233	28| 0.01155	30| 0.01084	40| 0.00804	68| 0.00473	81| 0.00416	62| 0.00519	51| 0.00634	45| 0.00725
Senegal	34| 0.00934	37| 0.00852	41| 0.00779	44| 0.00716	48| 0.00664	53| 0.00622	41| 0.00771	41| 0.0077	41| 0.00777	40| 0.00786	40| 0.00793	40| 0.008
Sierra Leone	51| 0.0065	55| 0.00646	52| 0.00647	51| 0.00651	51| 0.00662	50| 0.00675	25| 0.0135	29| 0.01155	32| 0.01035	34| 0.00973	35| 0.00949	35| 0.00948
Togo	56| 0.00547	61| 0.00543	56| 0.0055	55| 0.00561	54| 0.00571	53| 0.0058	28| 0.01095	27| 0.01121	27| 0.01136	27| 0.01134	28| 0.01116	28| 0.01087

*The number of draws is weighted by the inverse RMSE and sampled proportionally out of a total of 500 draws

GATHER Checklist

For *Global, Regional, and National Prevalence of Child and Adolescent Overweight and Obesity, 1990-2021, with Forecasts to 2050: A Forecasting Study for the Global Burden of Disease Study 2021*.

Item #	Checklist item	Reporting location
Objectives and funding		
1	Define the indicator(s), populations (including age, sex, and geographic entities), and time period(s) for which estimates were made.	Main text methods overview, paragraph 1
2	List the funding sources for the work.	Main text method section "role of the funders"
Data Inputs		
For all data inputs from multiple sources that are synthesized as part of the study:		
3	Describe how the data were identified and how the data were accessed.	Main text methods section: "Data Sources"
4	Specify the inclusion and exclusion criteria. Identify all ad-hoc exclusions.	Inclusion/exclusion criteria summarized in Methods section section "Data Sources" and Supplementary Methods "Inclusion Criteria"
5	Provide information on all included data sources and their main characteristics. For each data source used, report reference information or contact name/institution, population represented, data collection method, year(s) of data collection, sex and age range, diagnostic criteria or measurement method, and sample size, as relevant.	As noted in "Data Sources" the complete list of sources is available via the Global Health Exchange Sources tool: https://ghdx.healthdata.org/gbd-2021/sources
6	Identify and describe any categories of input data that have potentially important biases (e.g., based on characteristics listed in item 5).	Data inputs in excel format available on the GHDx (https://ghdx.healthdata.org/)
For data inputs that contribute to the analysis but were not synthesized as part of the study:		
7	Describe and give sources for any other data inputs.	N/A
For all data inputs:		
8	Provide all data inputs in a file format from which data can be efficiently extracted (e.g., a spreadsheet rather than a PDF), including all relevant meta-data listed in item 5. For any data inputs that cannot be shared because of ethical or legal reasons, such as third-party ownership, provide a contact name or the name of the institution that retains the right to the data.	Data inputs in excel format available on the GHDx (https://ghdx.healthdata.org/)
Data analysis		
9	Provide a conceptual overview of the data analysis method. A diagram may be helpful.	Main text methods
10	Provide a detailed description of all steps of the analysis, including mathematical formulae. This description should cover, as relevant, data cleaning, data pre-processing, data adjustments and weighting of data sources, and mathematical or statistical model(s).	Supplementary methods (appendix 1)
11	Describe how candidate models were evaluated and how the final model(s) were selected.	Main text methods; supplementary methods: "Prevalence estimation for overweight and obesity" and "Forecast modeling"
12	Provide the results of an evaluation of model performance, if done, as well as the results of any relevant sensitivity analysis.	N/A
13	Describe methods for calculating uncertainty of the estimates. State which sources of uncertainty were, and were not, accounted for in the uncertainty analysis.	Main text methods section "Estimation of overweight and obesity prevalence 1990 to 2021" and "Forecating overweight and obexity prevalence from 2022 to 2050"
14	State how analytic or statistical source code used to generate estimates can be accessed.	GitHub URL will be provided at resubmission
Results and Discussion		
15	Provide published estimates in a file format from which data can be efficiently extracted.	The results can be efficiently extracted at https://vizhub.healthdata.org/gbd-results/

16	Report a quantitative measure of the uncertainty of the estimates (e.g. uncertainty intervals).	UIs given for all findings, including in the text, figures, and tables in the main text and SM; online viz tools (see information above)
17	Interpret results in light of existing evidence. If updating a previous set of estimates, describe the reasons for changes in estimates.	Main text discussion
18	Discuss limitations of the estimates. Include a discussion of any modelling assumptions or data limitations that affect interpretation of the estimates.	Main text discussion

For *Global, Regional, and National Prevalence of Adult Overweight and Obesity, 1990-2021, with Forecasts to 2050: A Forecasting Study for the Global Burden of Disease Study 2021*.

Item #	Checklist item	Reporting location
Objectives and funding		
1	Define the indicator(s), populations (including age, sex, and geographic entities), and time period(s) for which estimates were made.	Main text methods overview, paragraph 1
2	List the funding sources for the work.	Main text method section "role of the funders"
Data Inputs		
For all data inputs from multiple sources that are synthesized as part of the study:		
3	Describe how the data were identified and how the data were accessed.	Main text methods section: "Data Sources"
4	Specify the inclusion and exclusion criteria. Identify all ad-hoc exclusions.	Inclusion/exclusion criteria summarized in Methods section section "Data Sources" and Supplementary Methods "Inclusion Criteria"
5	Provide information on all included data sources and their main characteristics. For each data source used, report reference information or contact name/institution, population represented, data collection method, year(s) of data collection, sex and age range, diagnostic criteria or measurement method, and sample size, as relevant.	As noted in "Data Sources" the complete list of sources is available via the Global Health Exchange Sources tool: https://ghdx.healthdata.org/
6	Identify and describe any categories of input data that have potentially important biases (e.g., based on characteristics listed in item 5).	Data inputs in excel format available on the GHDx (https://ghdx.healthdata.org/)
For data inputs that contribute to the analysis but were not synthesized as part of the study:		
7	Describe and give sources for any other data inputs.	N/A
For all data inputs:		
8	Provide all data inputs in a file format from which data can be efficiently extracted (e.g., a spreadsheet rather than a PDF), including all relevant meta-data listed in item 5. For any data inputs that cannot be shared because of ethical or legal reasons, such as third-party ownership, provide a contact name or the name of the institution that retains the right to the data.	Data inputs in excel format available on the GHDx (https://ghdx.healthdata.org/)
Data analysis		
9	Provide a conceptual overview of the data analysis method. A diagram may be helpful.	Main text methods
10	Provide a detailed description of all steps of the analysis, including mathematical formulae. This description should cover, as relevant, data cleaning, data pre-processing, data adjustments and weighting of data sources, and mathematical or statistical model(s).	Supplementary methods
11	Describe how candidate models were evaluated and how the final model(s) were selected.	Main text methods; supplementary methods: "Prevalence estimation for overweight and obesity" and "Forecast modeling"
12	Provide the results of an evaluation of model performance, if done, as well as the results of any relevant sensitivity analysis.	N/A
13	Describe methods for calculating uncertainty of the estimates. State which sources of uncertainty were, and were not, accounted for in the uncertainty analysis.	Main text methods section "Estimation of overweight and obesity prevalence from 1990 to 2021" and "Projection of overweigh and obexity prevalence from 2022 to 2050"
14	State how analytic or statistical source code used to generate estimates can be accessed.	GitHub URL will be provided at resubmission
Results and Discussion		
15	Provide published estimates in a file format from which data can be efficiently extracted.	The results can be efficiently extracted at https://vizhub.healthdata.org/gbd-results/
16	Report a quantitative measure of the uncertainty of the estimates (e.g. uncertainty intervals).	UIs given for all findings, including in the text, figures, and tables in the main text and SM; online viz tools (see information above)

17	Interpret results in light of existing evidence. If updating a previous set of estimates, describe the reasons for changes in estimates.	Main text discussion
18	Discuss limitations of the estimates. Include a discussion of any modelling assumptions or data limitations that affect interpretation of the estimates.	Main text discussion

References

1 Cole TJ, Lobstein T. Extended international (IOTF) body mass index cut-offs for thinness, overweight and obesity. *Pediatr Obes* 2012; **7**: 284–94.

2 Onis M de, Onyango AW, Borghi E, Siyam A, Nishida C, Siekmann J. Development of a WHO growth reference for school-aged children and adolescents. *Bulletin of the World Health Organization* 2007; **85**: 660.

3 Ng M, Fleming T, Robinson M, *et al.* Global, regional, and national prevalence of overweight and obesity in children and adults during 1980–2013: a systematic analysis for the Global Burden of Disease Study 2013. *The Lancet* 2014; **384**: 766–81.

4 Zheng P, Afshin A, Biryukov S, *et al.* The Burden of Proof studies: assessing the evidence of risk. *Nat Med* 2022; **28**: 2038–44.

5 Vollset SE, Ababneh HS, Abate YH, *et al.* Burden of disease scenarios for 204 countries and territories, 2022–2050: a forecasting analysis for the Global Burden of Disease Study 2021. *The Lancet* 2024; **403**: 2204–56.

6 Zheng P, Barber R, Sorensen RJD, Murray CJL, Aravkin AY. Trimmed Constrained Mixed Effects Models: Formulations and Algorithms. *Journal of Computational and Graphical Statistics* 2021; **30**: 544–56.

7 Foreman KJ, Marquez N, Dolgert A, *et al.* Forecasting life expectancy, years of life lost, and all-cause and cause-specific mortality for 250 causes of death: reference and alternative scenarios for 2016–40 for 195 countries and territories. *The Lancet* 2018; **392**: 2052–90.

8 Vollset SE, Goren E, Yuan C-W, *et al.* Fertility, mortality, migration, and population scenarios for 195 countries and territories from 2017 to 2100: a forecasting analysis for the Global Burden of Disease Study. *Lancet* 2020; **396**: 1285–306.

9 Bhattacharjee NV, Schumacher AE, Aali A, *et al.* Global fertility in 204 countries and territories, 1950–2021, with forecasts to 2100: a comprehensive demographic analysis for the Global Burden of Disease Study 2021. *The Lancet* 2024; **403**: 2057–99.

Supplementary Results

Supplementary figures and tables

Table S6: Age-standardised prevalence, percent change, and number with overweight or obesity in 1990, 2021, 2030, 2050, globally and within super-region (p 45)

Table S7: Age-standardised prevalence and number of children and young adolescents (5-14 years) with overweight or obesity, globally and within each super-region and region, by sex (pp 46-49)

Table S8: Age-standardised prevalence and number of older adolescents (15-24 years) with overweight or obesity, globally and within each super-region and region, by sex (pp 50-53)

Table S9: Age-standardised prevalence and number of older adolescents (5-14 years) with overweight (not obesity), globally and within each super-region and region, by sex (pp 54-57)

Table S10: Age-standardised prevalence and number of older adolescents (15-24 years) with overweight (not obesity), globally and within each super-region and region, by sex (pp 58-61)

Table S11: Age-standardised prevalence and number of older adolescents (5-14 years) with obesity (not overweight), globally and within each super-region and region, by sex (pp 62-65)

Table S12: Age-standardised prevalence and number of older adolescents (15-24 years) with obesity (not overweight), globally and within each super-region and region, by sex (pp 66-69)

Table S13: Age-standardised prevalence of children (5-9 years) with overweight or obesity in 1990, 2021, 2030, 2050, globally and within each super-region, by sex (p 70)

Table S14: Age-standardised prevalence of adolescents (10-14 years) with overweight or obesity in 1990, 2021, 2030, 2050, globally and within each super-region, by sex (p 71)

Table S15: Age-standardised prevalence of adolescents (15-19 years) with overweight or obesity in 1990, 2021, 2030, 2050, globally and within each super-region, by sex (p 72)

Table S16: Age-standardised prevalence of adolescents (20-24 years) with overweight or obesity in 1990, 2021, 2030, 2050, globally and within each super-region, by sex (p 73)

Figure S2: Age-standardised prevalence estimates from 1990 to 2021 and forecasts to 2050 for children and young adolescents aged 5 to 14 years old, by sex (pp 74-154)

Figure S3: Age-standardised prevalence estimates from 1990 to 2021 and forecasts to 2050 for older adolescents aged 15 to 24 years old, by sex (pp 155-235)

Figure S4: Ratio of overweight to obesity 1990-2050 for children and young adolescents 5-14 years old by sex and country (pp 236-239)

Figure S5: Ratio of overweight to obesity 1990-2050 for older adolescents 15-24 years old by sex and country (pp 240-243)

Figure S6: Ternary diagram of global change (1990-2050) in obesity, overweight, non-overweight prevalence, by age and sex (p 244)

Figure S7: Ternary diagram of change (1990, 2021, 2030, and 2050) in obesity, overweight, non-overweight/obesity prevalence, by age, sex, and super-region (p 245)

Figure S8: Country-level heatmaps of estimated age-standardised prevalence of obesity, 1990, 2021, 2030, 2050 by sex, super-region, and age group 5-14 vs. 15-24 years (pp 246-251)

Figure S9: Country-level heatmaps of estimated age-standardised prevalence of overweight and obesity, 1990, 2021, 2030, 2050 by sex, super-region, and age group (5-year age bands) (pp 252-263)

Figure S10: Country-level heatmaps of estimated age-standardised prevalence of obesity, 1990, 2021, 2030, 2050 by sex, super-region, and age group (5-year age bands) (pp 264-275)

Figure S11: Change in the percentage of 5–24-year-olds with overweight or obesity 1990-2050, by super-region and by sex (p 276)

Figure S12: Change in the percentage of 5–24-year-olds with obesity 1990-2050, by super-region and by sex (p 277)

Figure S13: Overweight and obesity prevalence over time by age group and super-region (p 278)

Figure S14: Obesity prevalence over time by age group and super-region (p 279)

Figure S15: Mean counts of children and young adolescents (5-14 years) with obesity (p 280)

Figure S16: Mean counts of children and young adolescents (5-14 years) with overweight (p 281)

Figure S17: Mean counts of children and young adolescents (5-14 years) with overweight or obesity (p 282)

Figure S18: Mean counts of older adolescents (15-24 years) with obesity (p 283)

Figure S19: Mean counts of older adolescents (15-24 years) with overweight (p 284)

Figure S20: Mean counts of older adolescents (15-24 years) with overweight or obesity (p 285)

Table S6: Age-standardised prevalence, number and percent change with overweight or obesity in 1990, 2021, 2030, 2050, globally and within super-region

		5-14-year-olds				15-24-year-olds			
		Prevalence % (95% UI)	Mean number overweight or obese (95% UI)	Observed percent change of OVOB 1990-2021 (95% UI)	Forecasted percent change of OVOB 2021-2050 (95% UI)	Prevalence % (95% UI)	Mean number overweight or obese (95% UI)	Observed percent change of OVOB 1990-2021 (95% UI)	Forecasted percent change of OVOB 2021-2050 (95% UI)
Global	1990	8.8 (8.5–9.1)	98,186,310 (94,608,962–102,004,681)	106 (95–116)		9.9(9.6–10.1)	99,685,024(97,216,295–102,423,659)	106 (98–115)	
	2021	18.1 (17.5–18.7)	244,654,786 (236,532,921–252,938,891)			20.3(19.7–21.0)	248,639,690(241,094,839–256,530,243)		
	2030	21.3 (20.2–22.2)	274,564,889 (257,955,821–290,035,529)		64 (46–74)	23.9(22.7–24.9)	319,426,048(303,629,583–334,776,407)		56 (40–65)
	2050	29.7 (26.1–31.8)	355,501,208 (295,450,629–410,830,223)			31.7(28.2–33.9)	390,033,516(331,214,302–440,188,699)		
High-income	1990	18.0 (16.6–19.5)	22,643,808 (20,942,640–24,511,866)	68 (52–83)		19.2 (18.6–19.8)	27,133,640 (26,268,279–28,039,056)	82 (73–90)	
	2021	30.2 (28.6–31.7)	36,988,201 (35,037,111–38,852,386)			34.9 (33.8–36.1)	45,067,556 (43,666,249–46,614,599)		
	2030	33.2 (31.1–35.2)	37,197,374 (34,423,956–40,008,365)		33 (21–40)	37.9 (36.2–39.4)	49,854,160 (47,298,104–52,253,665)		29 (19–35)
	2050	40.0 (35.6–43.2)	42,288,130 (36,256,242–48,159,792)			45.0 (41.1–47.7)	52,369,295 (46,120,853–58,094,784)		
Latin America and Caribbean	1990	14.7 (13.4–16.1)	13,900,003 (12,639,926–15,273,779)	104 (82–130)		18.2 (16.8–19.6)	14,196,606 (13,142,626–15,331,500)	98 (80–117)	
	2021	30.0 (28.0–32.1)	28,740,853 (26,824,448–30,798,338)			35.9 (33.8–37.8)	34,749,545 (32,739,971–36,664,504)		
	2030	34.8 (31.8–37.5)	32,258,163 (28,932,208–35,633,451)		56 (35–69)	41.3 (38.8–44.1)	39,159,096 (35,891,488–42,558,849)		47 (29–58)
	2050	46.8 (39.6–51.7)	35,555,660 (28,288,620–42,156,769)			52.8 (45.9–57.7)	44,362,963 (37,219,936–51,641,315)		
North Africa and Middle East	1990	13.2 (12.2–14.2)	11,735,705 (10,873,897–12,662,011)	155 (133–179)		16.2 (15.2–17.3)	10,717,379 (10,018,913–11,425,363)	132 (115–150)	
	2021	33.6 (31.7–35.5)	41,026,686 (38,725,871–43,308,624)			37.6 (36.2–38.9)	38,841,695 (37,415,585–40,193,495)		
	2030	39.7 (36.9–42.4)	48,022,926 (43,627,793–52,065,643)		59 (38–73)	44.1 (41.6–46.5)	53,329,202 (49,591,690–56,787,118)		52 (34–65)
	2050	53.3 (46.2–59.0)	63,791,826 (51,972,387–75,880,130)			57.1 (50.2–62.5)	68,219,852 (57,093,102–79,233,409)		
Central Europe, Eastern Europe, and Central Asia	1990	13.5 (12.7–14.4)	9,408,351 (8,816,407–10,053,706)	65 (49–82)		12.8 (12.0–13.7)	7,913,864 (7,419,741–8,463,811)	73 (57–90)	
	2021	22.2 (20.5–24.1)	12,212,469 (11,266,018–13,241,765)			22.2 (20.8–23.7)	10,259,864 (9,648,628–10,938,044)		
	2030	24.5 (22.5–26.7)	12,045,390 (10,864,188–13,388,404)		34 (20–44)	24.3 (22.4–26.2)	13,134,659 (11,880,390–14,307,348)		32 (19–41)
	2050	29.8 (25.8–33.4)	12,685,889 (10,500,402–14,690,140)			29.4 (25.6–32.3)	12,564,960 (10,635,018–14,402,417)		
Sub-Saharan Africa	1990	7.5 (7.0–8.0)	10,086,283 (9,499,958–10,717,728)	97 (81–114)		8.1 (7.6–8.6)	7,499,937 (7,058,670–7,992,101)	85 (70–101)	
	2021	14.7 (13.9–15.6)	44,612,566 (41,959,478–47,318,836)			14.9 (14.1–15.7)	33,674,534 (31,870,919–35,619,585)		
	2030	17.4 (16.4–18.5)	60,263,440 (55,122,745–66,142,650)		68 (57–75)	17.6 (16.6–18.6)	51,176,737 (47,649,895–55,047,394)		64 (54–71)
	2050	24.7 (22.7–26.5)	106,373,373 (89,911,315–124,968,128)			24.3 (22.3–26.0)	95,402,640 (82,645,102–108,246,641)		
Southeast Asia, East Asia, and Oceania	1990	5.8 (5.5–6.0)	18,873,708 (17,986,582–19,721,268)	171 (155–188)		6.1 (5.8–6.4)	22,099,473 (21,101,783–23,219,974)	169 (153–185)	
	2021	15.6 (14.9–16.3)	47,778,524 (45,797,513–49,891,476)			16.4 (15.7–17.0)	44,258,761 (42,623,615–45,984,449)		
	2030	18.8 (16.9–20.0)	48,354,092 (42,975,780–52,762,140)		80 (37–100)	20.7 (18.7–21.9)	62,221,391 (55,345,026–67,302,537)		78 (40–97)
	2050	28.0 (21.0–31.6)	54,248,135 (39,088,211–64,744,080)			29.1 (22.7–32.5)	58,664,397 (43,835,953–69,457,446)		
South Asia	1990	4.2 (3.4–5.1)	11,538,452 (9,408,905–14,149,156)	130 (74–203)		4.9 (4.1–5.9)	10,124,124 (8,448,967–12,140,493)	147 (92–208)	
	2021	9.5 (7.8–11.3)	33,295,488 (27,201,339–39,550,103)			12.0 (10.3–14.1)	41,787,736 (35,852,670–49,160,069)		
	2030	11.8 (9.7–13.8)	36,423,505 (29,266,648–43,262,447)		85 (60–101)	14.6 (12.6–17.1)	50,550,803 (42,138,520–59,966,611)		80 (56–94)
	2050	17.6 (14.1–20.9)	40,558,194 (29,574,204–52,256,854)			21.5 (17.3–25.3)	58,449,409 (44,927,814–73,274,724)		

Table S7: Age-standardised prevalence of children and young adolescents (5-14 years) with overweight or obesity, globally and within each super-region and region, by sex

		Females			Males		
		Prevalence % (95% UI)	Percent change of OVOB 1990-2021 (95% UI)	Percent change of OVOB 2021-2050 (95% UI)	Prevalence % (95% UI)	Percent change of OVOB 1990-2021 (95% UI)	Percent change of OVOB 2021-2050 (95% UI)
**GLOBAL**	**1990**	9.2 (8.7–9.7)	103 (88–118)	61 (43–71)	8.4 (8.0–8.8)	109 (96–123)	68 (48–78)
	**2021**	18.6 (17.8–19.6)			17.6 (16.8–18.3)		
	**2030**	22.0 (20.6–23.3)			20.7 (19.4–21.9)		
	**2050**	29.9 (26.3–32.4)			29.4 (25.7–31.7)		
**HIGH-INCOME**	**1990**	**17.1 (15.2–19.2)**	**73 (49–100)**	**36 (23–44)**	**18.8 (17.1–20.7)**	**64 (46–83)**	**30 (19–36)**
	**2021**	**29.5 (27.0–31.9)**			**30.8 (28.8–32.9)**		
	**2030**	**32.8 (29.9–35.6)**			**33.6 (31.0–36.1)**		
	**2050**	**40.1 (35.1–44.0)**			**39.9 (35.2–43.3)**		
High-income Asia Pacific	1990	10.3 (8.4–12.5)	91 (43–161)	51 (28–70)	11.8 (10.0–13.8)	84 (43–133)	42 (24–57)
	2021	19.5 (15.4–24.3)			21.6 (17.5–26.0)		
	2030	22.3 (17.6–28.1)			24.3 (19.5–28.6)		
	2050	29.2 (21.8–36.9)			30.6 (23.9–36.8)		
High-income Australasia	1990	19.3 (14.7–24.7)	69 (19–131)	41 (25–55)	22.0 (17.9–27.5)	71 (29–123)	38 (23–50)
	2021	32.0 (25.5–39.3)			37.4 (31.0–44.8)		
	2030	35.8 (28.8–43.9)			41.8 (35.0–49.5)		
	2050	44.8 (34.8–54.8)			51.3 (42.2–60.6)		
High-income Western Europe	1990	15.2 (13.7–16.9)	71 (49–92)	39 (32–46)	17.0 (15.5–18.6)	62 (43–80)	32 (26–37)
	2021	25.9 (23.8–28.1)			27.4 (25.4–29.4)		
	2030	29.1 (26.8–31.5)			30.1 (27.9–32.3)		
	2050	36.1 (33.0–39.1)			36.2 (33.0–38.9)		
High-income Southern Latin America	1990	17.2 (13.3–21.6)	78 (31–134)	48 (24–67)	18.9 (15.4–22.9)	84 (42–128)	41 (14–60)
	2021	30.2 (24.3–36.6)			34.6 (29.2–40.4)		
	2030	34.7 (28.1–41.5)			38.9 (32.8–45.7)		
	2050	44.9 (34.0–54.2)			48.7 (36.5–58.6)		
High-income North America	1990	23.5 (17.9–29.4)	58 (16–110)	26 (10–40)	25.1 (20.3–30.7)	46 (13–81)	20 (7–29)
	2021	36.4 (31.1–42.2)			36.2 (31.4–41.0)		
	2030	39.2 (32.7–45.5)			38.3 (33.1–44.0)		
	2050	45.8 (35.5–53.8)			43.3 (35.2–50.5)		
**LATIN AMERICA AND CARIBBEAN**	**1990**	**15.9 (13.8–18.3)**	**92 (61–128)**	**51 (31–64)**	**13.5 (11.8–15.3)**	**120 (88–156)**	**62 (38–76)**
	**2021**	**30.5 (27.3–33.9)**			**29.5 (26.8–32.2)**		

	**2030**	**34.9 (31.4–38.5)**			**34.7 (30.8–38.5)**		
	**2050**	**46.0 (38.5–51.7)**			**47.7 (39.6–53.3)**		
Caribbean	1990	15.5 (13.5–17.7)	61 (35–92)	42 (20–53)	10.8 (9.6–12.2)	73 (46–103)	58 (31–72)
	2021	24.9 (22.0–28.1)			18.6 (16.5–21.0)		
	2030	27.3 (23.7–31.2)			20.9 (18.1–23.8)		
	2050	35.3 (28.6–41.0)			29.5 (23.5–34.3)		
Andean Latin America	1990	20.4 (16.5–25.2)	77 (36–121)	38 (16–58)	16.4 (13.4–19.9)	91 (51–141)	47 (23–69)
	2021	35.8 (30.6–41.2)			30.9 (26.7–35.3)		
	2030	40.0 (33.6–46.5)			35.2 (29.7–40.0)		
	2050	49.4 (38.8–58.9)			45.3 (35.2–53.6)		
Central Latin America	1990	17.1 (14.2–20.7)	87 (46–132)	49 (30–63)	14.3 (11.8–16.8)	115 (73–161)	58 (34–75)
	2021	31.7 (27.7–35.9)			30.4 (27.3–33.6)		
	2030	36.2 (32.1–40.7)			35.5 (31.9–39.3)		
	2050	47.2 (39.5–53.6)			47.9 (39.8–54.4)		
Tropical Latin America	1990	13.5 (9.5–18.2)	115 (39–219)	62 (39–82)	12.3 (9.2–16.1)	148 (77–246)	74 (48–96)
	2021	28.3 (21.3–36.5)			30.0 (23.7–36.8)		
	2030	33.2 (26.0–42.2)			36.5 (29.1–44.6)		
	2050	45.6 (35.0–56.2)			52.1 (40.2–62.0)		
**NORTH AFRICA AND MIDDLE EAST**	**1990**	**15.8 (14.1–17.7)**	**134 (105–165)**	**49 (32–64)**	**10.7 (9.7–11.8)**	**187 (152–223)**	**69 (46–86)**
	**2021**	**36.8 (33.7–39.6)**			**30.6 (28.3–32.9)**		
	**2030**	**42.9 (39.1–46.2)**			**36.7 (33.6–39.7)**		
	**2050**	**55.1 (47.8–61.2)**			**51.6 (44.3–57.5)**		
**CENTRAL EUROPE, EASTERN EUROPE, AND CENTRAL ASIA**	**1990**	**12.9 (11.6–14.3)**	**67 (42–93)**	**37 (23–48)**	**14.1 (13.0–15.4)**	**63 (44–85)**	**32 (16–43)**
	**2021**	**21.4 (18.9–24.2)**			**23.0 (20.8–25.7)**		
	**2030**	**23.8 (21.0–26.3)**			**25.1 (22.7–28.0)**		
	**2050**	**29.2 (24.9–32.9)**			**30.3 (25.7–34.7)**		
Central Asia	1990	14.8 (12.6–17.4)	58 (29–92)	34 (23–44)	13.6 (12.0–15.6)	51 (27–77)	31 (20–42)
	2021	23.3 (20.2–26.9)			20.5 (18.0–23.1)		
	2030	25.7 (22.3–29.9)			22.3 (19.5–25.4)		
	2050	31.2 (26.8–36.2)			26.7 (22.8–31.3)		
Central Europe	1990	12.0 (10.5–13.9)	75 (43–109)	35 (28–43)	15.6 (14.0–17.4)	60 (38–83)	27 (22–37)
	2021	21.0 (18.3–23.7)			24.9 (22.4–27.6)		
	2030	23.2 (20.6–26.1)			27.1 (24.4–30.0)		
	2050	28.2 (25.1–31.9)			31.7 (28.6–35.7)		
Eastern Europe	1990	12.4 (10.4–14.7)	64 (20–114)	35 (11–51)	13.5 (11.5–15.6)	78 (38–126)	42 (13–60)
	2021	20.2 (15.2–25.6)			23.9 (19.6–29.0)		
	2030	22.2 (16.5–28.6)			26.8 (21.3–32.9)		
	2050	27.2 (18.5–36.1)			34.0 (24.0–43.1)		

**SUB-SAHARAN AFRICA**	**1990**	**9.1 (8.4–10.0)**	**79 (59–100)**	**54 (44–61)**	**5.8 (5.4–6.3)**	**126 (102–152)**	**85 (73–93)**
	**2021**	**16.3 (15.0–17.8)**			**13.2 (12.1–14.3)**		
	**2030**	**18.9 (17.2–20.6)**			**16.1 (14.7–17.3)**		
	**2050**	**25.1 (22.6–27.7)**			**24.3 (22.0–26.4)**		
Central sub-Saharan Africa	1990	8.7 (7.1–11.0)	145 (79–215)	88 (58–104)	5.6 (4.4–6.8)	182 (117–260)	109 (78–124)
	2021	21.2 (17.1–25.9)			15.5 (12.9–18.6)		
	2030	26.4 (21.5–32.1)			20.0 (16.5–24.0)		
	2050	39.9 (30.8–48.1)			32.5 (25.6–38.6)		
Eastern sub-Saharan Africa	1990	9.5 (8.3–10.8)	72 (46–101)	54 (47–63)	5.7 (5.1–6.3)	121 (93–153)	88 (77–105)
	2021	16.3 (14.5–18.2)			12.5 (11.3–13.8)		
	2030	18.8 (16.8–20.8)			15.2 (13.7–17.0)		
	2050	25.2 (22.6–28.0)			23.3 (20.9–26.4)		
Southern sub-Saharan Africa	1990	13.7 (10.8–17.3)	94 (43–155)	53 (28–72)	7.9 (6.1–10.0)	125 (63–201)	58 (27–80)
	2021	26.1 (21.7–30.9)			17.5 (14.4–21.0)		
	2030	30.0 (24.7–35.2)			20.3 (16.6–24.5)		
	2050	39.8 (31.4–46.8)			27.6 (20.7–34.0)		
Western sub-Saharan Africa	1990	7.7 (6.5–9.2)	81 (40–127)	49 (39–57)	5.6 (4.8–6.5)	126 (82–179)	82 (68–93)
	2021	13.8 (11.5–16.5)			12.6 (10.8–14.9)		
	2030	15.8 (13.4–18.9)			15.3 (13.1–18.0)		
	2050	20.5 (17.0–24.5)			22.8 (19.6–26.5)		
**SOUTHEAST ASIA, EAST ASIA, AND OCEANIA**	**1990**	**5.5 (5.1–5.9)**	**170 (145–197)**	**87 (44–108)**	**6.0 (5.7–6.4)**	**172 (152–193)**	**74 (31–94)**
	**2021**	**14.7 (13.8–15.8)**			**16.4 (15.5–17.3)**		
	**2030**	**18.1 (16.1–19.7)**			**19.4 (17.5–20.8)**		
	**2050**	**27.5 (20.6–31.4)**			**28.4 (21.1–32.3)**		
East Asia	1990	5.5 (5.1–5.9)	201 (173–236)	114 (39–143)	7.0 (6.5–7.5)	193 (169–220)	97 (30–121)
	2021	16.6 (15.3–17.8)			20.4 (19.3–21.7)		
	2030	21.6 (18.3–23.6)			25.7 (22.4–27.7)		
	2050	35.3 (22.7–40.9)			39.9 (26.1–45.4)		
Southeast Asia	1990	5.2 (4.4–6.0)	124 (81–174)	69 (50–87)	4.1 (3.5–4.7)	133 (92–178)	69 (47–89)
	2021	11.6 (9.9–13.5)			9.4 (8.3–10.8)		
	2030	13.6 (11.7–15.9)			11.2 (9.8–12.9)		
	2050	19.4 (16.0–23.0)			16.0 (13.3–19.0)		
Oceania	1990	19.9 (16.6–23.7)	39 (7–77)	32 (25–54)	13.2 (11.4–15.4)	62 (30–100)	58 (47–89)
	2021	27.4 (22.5–33.1)			21.3 (17.9–25.2)		
	2030	29.6 (23.8–36.0)			24.2 (20.0–29.0)		
	2050	36.4 (29.0–45.9)			33.5 (26.7–42.7)		
**SOUTH ASIA**	**1990**	**4.5 (3.4–6.1)**	**126 (48–232)**	**82 (54–100)**	**3.9 (3.0–5.0)**	**139 (66–233)**	**90 (63–106)**
	**2021**	**9.9 (7.5–13.1)**			**9.2 (7.1–11.4)**		

**2030**	**12.2 (9.2–15.7)**	**11.3 (8.8–14.0)**
**2050**	**17.9 (13.3–23.6)**	**17.3 (13.0–21.4)**

Table S8: Age-standardised prevalence of older adolescents (15-24 years) with overweight or obesity, globally and within each super-region and region, by sex

		Females			Males		
		Prevalence % (95% UI)	Percent change of OVOB 1990-2021 (95% UI)	Percent change of OVOB 2021-2050 (95% UI)	Prevalence % (95% UI)	Percent change of OVOB 1990-2021 (95% UI)	Percent change of OVOB 2021-2050 (95% UI)
**GLOBAL**	**1990**	**9.9 (9.5–10.4)**	**111 (99–124)**	**55 (39–64)**	**9.8 (9.5–10.2)**	**102 (90–114)**	**58 (40–67)**
	**2021**	**21.0 (20.1–21.9)**			**19.8 (18.9–20.7)**		
	**2030**	**24.5 (23.1–25.7)**			**23.2 (21.9–24.5)**		
	**2050**	**32.4 (28.9–34.8)**			**31.1 (27.4–33.6)**		
**HIGH-INCOME**	**1990**	**16.8 (16.0–17.7)**	**104 (90–118)**	**32 (21–38)**	**21.5 (20.6–22.4)**	**65 (55–76)**	**27 (18–33)**
	**2021**	**34.3 (32.6–36.0)**			**35.5 (34.0–37.1)**		
	**2030**	**37.4 (35.3–39.5)**			**38.4 (36.5–40.3)**		
	**2050**	**45.0 (40.5–48.1)**			**45.0 (41.0–47.9)**		
High-income Asia Pacific	1990	5.7 (4.5–7.0)	111 (51–193)	63 (35–87)	10.5 (9.1–12.3)	85 (45–129)	47 (28–63)
	2021	11.8 (9.1–15.0)			19.4 (16.1–22.8)		
	2030	14.0 (11.2–17.5)			22.1 (18.5–26.1)		
	2050	19.3 (14.3–25.1)			28.5 (22.9–34.3)		
High-income Australasia	1990	20.5 (17.1–24.5)	75 (34–119)	37 (23–48)	25.7 (21.7–29.7)	65 (33–104)	33 (21–43)
	2021	35.6 (31.0–40.8)			42.2 (36.9–47.7)		
	2030	39.7 (34.5–45.1)			46.6 (41.0–52.3)		
	2050	48.6 (40.6–56.1)			56.0 (48.0–63.3)		
High-income Western Europe	1990	15.7 (14.5–17.1)	75 (57–96)	38 (31–44)	19.8 (18.5–21.3)	52 (37–66)	30 (24–35)
	2021	27.3 (25.6–29.4)			30.1 (28.1–32.0)		
	2030	30.5 (28.5–32.7)			33.1 (31.1–35.0)		
	2050	37.6 (34.8–40.7)			39.1 (36.0–41.4)		
High-income Southern Latin America	1990	18.8 (15.2–23.3)	92 (49–142)	42 (21–55)	20.5 (16.8–24.5)	86 (47–129)	37 (13–53)
	2021	35.7 (31.1–40.7)			37.7 (33.2–42.6)		
	2030	40.0 (34.4–46.2)			42.0 (35.7–47.6)		
	2050	50.3 (40.5–59.0)			51.6 (39.7–60.1)		
High-income North America	1990	25.4 (23.4–27.4)	94 (75–116)	19 (7–27)	31.1 (29.6–32.7)	48 (36–62)	16 (6–22)
	2021	49.2 (45.4–52.9)			46.1 (43.0–49.3)		
	2030	51.9 (47.7–56.0)			48.2 (44.4–52.0)		
	2050	58.3 (50.6–64.1)			53.3 (46.5–58.5)		
**LATIN AMERICA AND CARIBBEAN**	**1990**	**19.4 (17.5–21.6)**	**88 (64–112)**	**43 (27–54)**	**16.9 (15.2–18.8)**	**110 (83–137)**	**52 (32–65)**
	**2021**	**36.4 (33.6–39.4)**			**35.3 (32.8–38.1)**		

	**2030**	**41.5 (38.0–45.2)**			**41.2 (38.0–44.6)**		
	**2050**	**52.0 (45.1–57.7)**			**53.6 (46.3–59.2)**		
Caribbean	1990	20.0 (17.7–22.6)	59 (36–84)	35 (17–45)	15.4 (13.7–17.3)	61 (39–85)	44 (22–56)
	2021	31.7 (28.7–34.8)			24.8 (22.6–27.0)		
	2030	35.0 (31.2–38.6)			27.7 (25.0–30.5)		
	2050	42.9 (36.3–48.0)			35.5 (29.4–39.9)		
Andean Latin America	1990	20.0 (16.3–24.2)	84 (46–129)	37 (16–55)	16.7 (13.9–20.0)	95 (58–138)	44 (22–62)
	2021	36.4 (32.2–40.9)			32.3 (29.4–35.5)		
	2030	40.5 (35.2–45.9)			36.5 (32.8–40.7)		
	2050	50.0 (40.2–58.7)			46.4 (38.2–54.1)		
Central Latin America	1990	23.2 (20.5–26.3)	82 (56–111)	38 (23–49)	20.1 (17.3–23.0)	98 (65–132)	45 (28–58)
	2021	41.9 (38.7–45.6)			39.6 (36.2–43.0)		
	2030	47.3 (43.1–51.3)			45.8 (41.6–49.3)		
	2050	57.7 (50.2–63.2)			57.4 (50.0–62.7)		
Tropical Latin America	1990	14.8 (11.1–19.1)	109 (43–188)	59 (37–78)	13.7 (10.6–17.6)	146 (74–232)	68 (44–88)
	2021	30.3 (23.9–37.5)			33.3 (27.3–39.3)		
	2030	35.6 (28.2–43.4)			39.9 (33.4–46.6)		
	2050	48.1 (37.7–57.9)			55.5 (45.0–64.2)		
**NORTH AFRICA AND MIDDLE EAST**	**1990**	**19.8 (18.0–21.8)**	**114 (91–139)**	**43 (27–55)**	**12.8 (11.6–13.9)**	**162 (134–190)**	**63 (42–79)**
	**2021**	**42.2 (40.1–44.3)**			**33.3 (31.5–35.2)**		
	**2030**	**48.6 (45.4–51.5)**			**39.9 (37.0–42.6)**		
	**2050**	**60.2 (53.5–65.6)**			**54.3 (46.9–59.9)**		
**CENTRAL EUROPE, EASTERN EUROPE, AND CENTRAL ASIA**	**1990**	**11.7 (10.5–12.9)**	**72 (47–100)**	**35 (21–43)**	**14.0 (12.8–15.3)**	**73 (53–94)**	**30 (17–40)**
	**2021**	**20.1 (18.2–22.1)**			**24.2 (22.5–26.2)**		
	**2030**	**22.1 (19.7–24.7)**			**26.3 (23.9–28.7)**		
	**2050**	**27.0 (23.1–30.5)**			**31.5 (27.3–35.3)**		
Central Asia	1990	12.1 (10.6–13.8)	69 (40–102)	35 (23–45)	14.5 (12.9–16.1)	55 (32–81)	29 (19–39)
	2021	20.2 (18.1–22.7)			22.4 (20.3–24.5)		
	2030	22.2 (19.8–24.8)			24.3 (22.1–26.9)		
	2050	27.3 (23.6–30.9)			28.9 (25.5–32.8)		
Central Europe	1990	10.9 (9.4–12.5)	82 (53–116)	36 (30–45)	16.6 (14.9–18.3)	65 (43–88)	26 (21–35)
	2021	19.8 (17.6–22.3)			27.3 (25.0–29.7)		
	2030	22.1 (19.7–24.7)			29.5 (27.2–32.1)		
	2050	27.0 (24.1–30.2)			34.3 (31.5–37.8)		
Eastern Europe	1990	11.9 (9.9–14.3)	70 (28–123)	33 (9–47)	12.2 (10.2–14.6)	96 (50–148)	39 (11–56)
	2021	20.1 (16.4–24.4)			23.7 (19.9–28.1)		
	2030	22.1 (17.5–27.4)			26.2 (21.2–30.9)		
	2050	26.9 (19.1–34.5)			32.8 (23.2–40.8)		

**SUB-SAHARAN AFRICA**	**1990**	**10.5 (9.6–11.3)**	**69 (52–88)**	**49 (40–55)**	**5.5 (5.0–6.1)**	**120 (92–148)**	**87 (74–95)**
	**2021**	**17.6 (16.5–18.9)**			**12.0 (11.0–13.2)**		
	**2030**	**20.3 (19.0–21.9)**			**14.9 (13.7–16.3)**		
	**2050**	**26.3 (23.9–28.7)**			**22.4 (20.3–24.6)**		
Central sub-Saharan Africa	1990	7.1 (5.7–8.9)	139 (76–208)	96 (61–113)	3.1 (2.5–3.8)	186 (122–263)	128 (92–146)
	2021	16.8 (13.6–20.3)			8.7 (7.2–10.4)		
	2030	21.1 (17.2–25.4)			11.6 (9.4–14.2)		
	2050	32.8 (25.0–39.3)			20.1 (15.2–24.7)		
Eastern sub-Saharan Africa	1990	8.7 (7.7–9.8)	72 (48–99)	53 (46–62)	4.4 (4.0–5.0)	120 (89–154)	90 (78–106)
	2021	15.0 (13.6–16.4)			9.7 (8.7–10.8)		
	2030	17.4 (15.8–19.0)			12.1 (10.9–13.4)		
	2050	22.9 (20.7–25.4)			18.5 (16.6–20.9)		
Southern sub-Saharan Africa	1990	20.3 (16.8–23.7)	90 (50–136)	38 (22–51)	7.6 (6.0–9.4)	98 (41–165)	59 (27–82)
	2021	38.3 (34.0–43.1)			14.8 (11.7–18.2)		
	2030	42.9 (37.5–47.9)			17.3 (13.5–20.9)		
	2050	52.8 (44.5–58.8)			23.6 (16.8–29.0)		
Western sub-Saharan Africa	1990	10.2 (8.8–12.0)	71 (37–107)	46 (38–52)	6.7 (5.6–8.0)	123 (74–179)	77 (62–89)
	2021	17.4 (15.2–19.8)			14.7 (12.5–17.2)		
	2030	19.9 (17.4–22.7)			18.0 (15.4–21.0)		
	2050	25.3 (22.1–28.8)			26.0 (22.3–30.0)		
**SOUTHEAST ASIA, EAST ASIA, AND OCEANIA**	**1990**	**5.7 (5.3–6.2)**	**178 (152–206)**	**84 (48–104)**	**6.4 (6.0–6.8)**	**161 (142–182)**	**73 (32–93)**
	**2021**	**15.9 (14.9–16.9)**			**16.7 (15.9–17.6)**		
	**2030**	**20.0 (18.0–21.6)**			**21.3 (19.1–22.8)**		
	**2050**	**29.3 (23.1–33.0)**			**29.0 (22.0–32.6)**		
East Asia	1990	5.4 (5.0–5.9)	210 (177–244)	113 (40–142)	6.9 (6.4–7.4)	202 (177–228)	95 (29–118)
	2021	16.8 (15.7–17.8)			20.8 (19.7–21.9)		
	2030	21.8 (18.9–23.7)			26.4 (23.0–28.5)		
	2050	35.5 (23.1–40.8)			40.4 (26.6–46.0)		
Southeast Asia	1990	6.5 (5.5–7.5)	127 (84–178))	61 (44–78)	4.9 (4.3–5.5)	124 (87–165)	64 (42–82)
	2021	14.6 (12.8–16.6)			10.9 (9.7–12.3)		
	2030	17.0 (14.8–19.2)			12.8 (11.4–14.5)		
	2050	23.5 (20.0–27.4)			17.8 (15.0–21.0)		
Oceania	1990	20.0 (17.1–23.5)	35 (4–71)	30 (23–52)	14.2 (12.2–16.5)	53 (21–90)	55 (44–88)
	2021	26.9 (22.4–32.4)			21.6 (18.0–25.4)		
	2030	29.4 (24.5–35.7)			24.9 (20.8–29.0)		
	2050	35.0 (28.3–43.8)			33.5 (26.7–41.9)		
**SOUTH ASIA**	**1990**	**4.9 (3.8–6.3)**	**142 (67–234)**	**78 (53–95)**	**4.9 (3.8–6.1)**	**156 (84–251)**	**82 (59–97)**
	**2021**	**11.6 (9.3–14.5)**			**12.3 (9.9–15.3)**		

**2030**	**14.2 (11.5–17.3)**	**15.0 (12.3–18.3)**
**2050**	**20.6 (16.2–25.5)**	**22.2 (17.5–27.3)**

Table S9: Age-standardised prevalence of children and younger adolescents (5-14 years) with overweight (not obesity), globally and within each super-region and region, by sex

		Females			Male		
		Prevalence % (95% UI)	Percent change of OVOB 1990-2021 (95% UI)	Percent change of OVOB 2021-2050 (95% UI)	Prevalence % (95% UI)	Percent change of OVOB 1990-2021 (95% UI)	Percent change of OVOB 2021-2050 (95% UI)
**GLOBAL**	**1990**	**7.1 (6.7–7.6)**	**68 (56–82)**	**28 (22–33)**	**6.4 (6.0–6.7)**	**64 (53–75)**	**24 (20–28)**
	**2021**	**12.0 (11.4–12.7)**			**10.4 (9.9–11.0)**		
	**2030**	**13.2 (12.5–14.0)**			**11.3 (10.7–11.9)**		
	**2050**	**15.3 (14.3–16.3)**			**12.9 (12.2–13.6)**		
**HIGH-INCOME**	**1990**	**12.1 (10.7–13.7)**	**49 (27–74)**	**16 (9–21)**	**13.4 (12.1–14.9)**	**38 (20–56)**	**7 (2–11)**
	**2021**	**17.9 (16.1–19.7)**			**18.4 (17.0–19.9)**		
	**2030**	**19.0 (17.0–21.0)**			**18.9 (17.4–20.5)**		
	**2050**	**20.8 (18.5–23.2)**			**19.6 (17.8–21.4)**		
High-income Asia Pacific	1990	8.8 (7.1–10.6)	68 (23–129)	29 (11–46)	9.4 (7.9–11.1)	52 (15–93)	11 (-2–26)
	2021	14.6 (11.4–18.3)			14.3 (11.5–17.4)		
	2030	16.0 (12.2–20.1)			14.9 (11.9–18.0)		
	2050	18.7 (14.1–23.9)			15.8 (12.1–19.6)		
High-income Australasia	1990	12.1 (9.2–15.7)	25 (-14–75)	6 (-10–23)	16.5 (13.1–21.1)	37 (0–83)	8 (-8–26)
	2021	14.8 (11.4–18.5)			22.5 (18.0–27.7)		
	2030	15.2 (11.8–19.3)			23.4 (19.3–29.0)		
	2050	15.6 (11.9–20.6)			24.3 (18.9–31.2)		
High-income Western Europe	1990	11.3 (10.2–12.6)	44 (26–64)	17 (12–22)	12.7 (11.6–13.9)	34 (19–51)	10 (5–13)
	2021	16.2 (14.8–17.7)			17.0 (15.6–18.3)		
	2030	17.3 (15.7–18.8)			17.6 (16.1–19.1)		
	2050	19.0 (17.2–20.7)			18.6 (17.0–20.1)		
High-income Southern Latin America	1990	13.5 (10.4–17.3)	49 (6–101)	20 (10–30)	15.3 (12.4–18.8)	47 (12–88)	5 (-7–15)
	2021	19.9 (15.5–24.7)			22.3 (18.4–26.6)		
	2030	21.6 (16.9–26.6)			23.2 (19.2–27.6)		
	2050	24.0 (18.9–29.1)			23.5 (18.7–28.1)		
High-income North America	1990	14.7 (10.9–19.2)	44 (0–104)	12 (0–23)	16.0 (12.3–20.1)	28 (-8–69)	3 (-6–10)
	2021	20.7 (16.6–25.1)			20.1 (16.5–23.8)		
	2030	21.6 (17.0–26.1)			20.3 (16.6–24.1)		
	2050	23.2 (17.3–28.2)			20.5 (16.1–25.0)		
**LATIN AMERICA AND CARIBBEAN**	**1990**	**12.5 (10.8–14.3)**	**57 (32–87)**	**15 (6–24)**	**10.5 (9.1–12.0)**	**63 (38–94)**	**8 (0–15)**
	**2021**	**19.5 (17.3–21.7)**			**17.0 (15.2–18.9)**		

	**2030**	**20.7 (18.7–23.0)**			**17.9 (16.0–20.1)**		
	**2050**	**22.3 (19.7–24.9)**			**18.3 (16.3–20.7)**		
Caribbean	1990	11.0 (9.5–12.7)	33 (9–63)	17 (9–26)	8.1 (7.1–9.2)	44 (19–73)	30 (19–41)
	2021	14.6 (12.6–16.9)			11.6 (10.1–13.3)		
	2030	15.3 (12.9–17.8)			12.5 (10.7–14.5)		
	2050	17.1 (14.2–20.2)			15.1 (12.9–17.7)		
Andean Latin America	1990	17.2 (13.7–21.1)	51 (16–90)	16 (-6–34)	13.5 (11.0–16.5)	55 (21–96)	15 (-9–35)
	2021	25.6 (21.6–29.8)			20.8 (17.6–24.0)		
	2030	27.3 (22.5–32.6)			22.2 (18.0–26.2)		
	2050	29.7 (22.4–36.8)			23.9 (17.3–30.2)		
Central Latin America	1990	13.5 (11.2–16.5)	53 (19–92)	14 (1–25)	11.0 (9.1–13.0)	60 (27–97)	4 (-8–16)
	2021	20.4 (17.9–23.3)			17.4 (15.2–19.7)		
	2030	21.8 (19.3–24.7)			18.1 (16.0–20.5)		
	2050	23.4 (20.2–27.0)			18.1 (15.1–21.1)		
Tropical Latin America	1990	10.4 (7.1–14.1)	69 (4–156)	16 (4–28)	9.5 (7.1–12.5)	76 (18–155)	6 (-7–18)
	2021	17.0 (12.4–22.4)			16.4 (12.3–21.1)		
	2030	18.3 (13.8–23.7)			17.4 (13.3–21.8)		
	2050	19.6 (15.1–24.8)			17.3 (13.3–21.8)		
**NORTH AFRICA AND MIDDLE EAST**	**1990**	**12.2 (10.8–13.7)**	**63 (41–87)**	**-4 (-13–6)**	**8.3 (7.5–9.3)**	**85 (60–112)**	**-3 (-15–7)**
	**2021**	**19.9 (18.1–21.7)**			**15.4 (14.0–16.9)**		
	**2030**	**20.4 (18.7–22.1)**			**15.9 (14.3–17.5)**		
	**2050**	**19.1 (17.0–21.5)**			**14.9 (12.7–16.8)**		
**CENTRAL EUROPE, EASTERN EUROPE, AND CENTRAL ASIA**	**1990**	**9.7 (8.8–10.7)**	**49 (27–74)**	**24 (13–34)**	**10.5 (9.5–11.4)**	**43 (24–65)**	**17 (3–26)**
	**2021**	**14.4 (12.7–16.4)**			**14.9 (13.4–16.8)**		
	**2030**	**15.6 (13.9–17.5)**			**15.8 (14.2–17.7)**		
	**2050**	**17.8 (15.4–20.2)**			**17.5 (15.2–20.0)**		
Central Asia	1990	12.3 (10.5–14.5)	43 (16–74)	23 (10–34)	10.4 (9.2–12.1)	37 (14–61)	20 (8–32)
	2021	17.5 (15.0–20.2)			14.2 (12.4–16.3)		
	2030	18.8 (16.2–21.8)			15.2 (13.1–17.5)		
	2050	21.5 (17.9–25.4)			17.1 (14.3–20.2)		
Central Europe	1990	9.2 (7.9–10.8)	50 (20–83)	14 (6–29)	11.1 (9.9–12.5)	37 (18–58)	10 (3–23)
	2021	13.6 (11.8–15.7)			15.2 (13.4–17.3)		
	2030	14.4 (12.5–16.6)			15.9 (14.1–17.9)		
	2050	15.6 (13.3–18.4)			16.8 (14.7–19.7)		
Eastern Europe	1990	8.7 (7.4–10.4)	45 (3–96)	17 (-7–28)	10.1 (8.6–11.7)	53 (16–97)	19 (-7–31)
	2021	12.5 (9.4–16.5)			15.3 (12.2–18.8)		
	2030	13.2 (9.6–17.5)			16.3 (12.9–20.5)		
	2050	14.6 (10.3–19.6)			18.1 (13.6–23.1)		

**SUB-SAHARAN AFRICA**	**1990**	**7.3 (6.7–8.1)**	**58 (40–77)**	**29 (23–35)**	**4.7 (4.3–5.1)**	**83 (62–106)**	**39 (33–45)**
	**2021**	**11.5 (10.5–12.6)**			**8.5 (7.7–9.3)**		
	**2030**	**12.7 (11.6–14.0)**			**9.6 (8.7–10.5)**		
	**2050**	**14.9 (13.5–16.6)**			**11.8 (10.6–13.0)**		
Central sub-Saharan Africa	1990	7.1 (5.7–9.0)	102 (45–165)	40 (18–55)	4.2 (3.3–5.1)	92 (45–150)	24 (2–40)
	2021	14.1 (11.2–17.5)			7.9 (6.5–9.7)		
	2030	16.3 (13.0–20.1)			8.8 (7.0–10.8)		
	2050	19.8 (15.5–24.2)			9.8 (7.5–12.6)		
Eastern sub-Saharan Africa	1990	7.8 (6.8–8.8)	55 (32–83)	34 (26–43)	4.5 (4.1–5.0)	82 (57–109)	47 (36–62)
	2021	12.1 (10.7–13.5)			8.3 (7.4–9.2)		
	2030	13.4 (11.9–15.0)			9.4 (8.5–10.7)		
	2050	16.2 (14.4–18.3)			12.1 (10.7–13.9)		
Southern sub-Saharan Africa	1990	10.2 (8.0–13.0)	54 (12–104)	22 (9–33)	6.0 (4.6–7.6)	67 (20–127)	15 (1–25)
	2021	15.5 (12.6–18.4)			9.9 (7.9–12.1)		
	2030	16.7 (13.7–19.8)			10.6 (8.5–12.6)		
	2050	18.9 (15.5–22.2)			11.4 (9.2–13.7)		
Western sub-Saharan Africa	1990	6.2 (5.2–7.4)	61 (24–104)	28 (20–35)	4.6 (3.9–5.4)	91 (51–140)	40 (27–48)
	2021	9.9 (8.2–11.8)			8.7 (7.3–10.5)		
	2030	10.8 (9.0–13.0)			9.8 (8.3–11.8)		
	2050	12.6 (10.4–15.0)			12.1 (10.0–14.3)		
**SOUTHEAST ASIA, EAST ASIA, AND OCEANIA**	**1990**	**4.5 (4.2–4.9)**	**122 (102–145)**	**44 (28–54)**	**4.6 (4.3–4.9)**	**102 (87–119)**	**27 (17–40)**
	**2021**	**10.1 (9.4–10.8)**			**9.3 (8.8–9.9)**		
	**2030**	**11.4 (10.4–12.3)**			**10.2 (9.6–10.9)**		
	**2050**	**14.4 (12.5–15.9)**			**11.9 (10.8–13.2)**		
East Asia	1990	4.7 (4.3–5.1)	145 (121–173)	58 (25–75)	5.4 (5.0–5.8)	112 (93–133)	32 (12–57)
	2021	11.5 (10.7–12.4)			11.3 (10.6–12.1)		
	2030	13.8 (12.2–15.0)			12.9 (11.8–13.9)		
	2050	18.1 (14.1–20.3)			15.1 (12.6–17.9)		
Southeast Asia	1990	4.1 (3.5–4.7)	86 (50–131)	38 (27–51)	3.1 (2.7–3.6)	88 (55–127)	36 (24–48)
	2021	7.5 (6.4–8.9)			5.8 (5.0–6.6)		
	2030	8.4 (7.2–9.9)			6.5 (5.6–7.4)		
	2050	10.4 (8.6–12.4)			7.9 (6.6–9.4)		
Oceania	1990	15.3 (12.5–18.6)	28 (-6–69)	21 (14–41)	10.5 (9.0–12.5)	55 (21–95)	45 (36–76)
	2021	19.3 (15.4–24.1)			16.2 (13.4–19.5)		
	2030	20.7 (16.2–26.2)			18.2 (14.8–22.2)		
	2050	23.7 (18.4–30.3)			23.5 (18.7–30.1)		
**SOUTH ASIA**	**1990**	**3.6 (2.7–4.9)**	**88 (21–178)**	**45 (30–57)**	**3.0 (2.3–3.9)**	**102 (39–186)**	**52 (41–65)**
	**2021**	**6.7 (5.0–8.9)**			**6.0 (4.6–7.6)**		

**2030**	**7.6 (5.7–9.8)**	**6.9 (5.4–8.8)**
**2050**	**9.5 (7.1–12.4)**	**9.1 (7.0–11.4)**

Table S10: Age-standardised prevalence of older adolescents (15-24 years) with overweight (not obesity), globally and within each super-region and region, by sex

		Females			Males		
		Prevalence % (95% UI)	Percent change of OVOB 1990-2021 (95% UI)	Percent change of OVOB 2021-2050 (95% UI)	Prevalence % (95% UI)	Percent change of OVOB 1990-2021 (95% UI)	Percent change of OVOB 2021-2050 (95% UI)
**GLOBAL**	**1990**	**7.8 (7.4–8.1)**	**75 (64–88)**	**27 (21–31)**	**8.2 (7.9–8.5)**	**69 (59–81)**	**29 (23–33)**
	**2021**	**13.6 (13.0–14.4)**			**13.9 (13.2–14.7)**		
	**2030**	**15.0 (14.3–15.8)**			**15.4 (14.6–16.2)**		
	**2050**	**17.2 (16.2–18.2)**			**17.8 (16.8–18.8)**		
**HIGH-INCOME**	**1990**	**11.5 (10.9–12.2)**	**55 (42, 70)**	**13 (8, 16)**	**16.8 (16.1–17.5)**	**31 (22–41)**	**8 (4–11)**
	**2021**	**17.9 (16.7–19.2)**			**22.0 (20.9–23.3)**		
	**2030**	**18.6 (17.4–20.0)**			**22.8 (21.6–24.1)**		
	**2050**	**19.9 (18.5–21.5)**			**23.7 (22.2–25.1)**		
High-income Asia Pacific	1990	4.9 (3.9–6.1)	93 (39–168)	40 (18–60)	9.3 (8.0–10.7)	64 (27–105)	23 (7–37)
	2021	9.4 (7.2–12.1)			15.1 (12.3–18.1)		
	2030	10.6 (8.4–13.4)			16.5 (13.6–19.6)		
	2050	13.1 (9.7–16.9)			18.6 (14.8–23.0)		
High-income Australasia	1990	13.1 (10.6–16.2)	26 (-7–66)	3 (-13–17)	20.6 (17.1–24.0)	37 (9–74)	8 (-5–23)
	2021	16.4 (13.6–19.7)			28.0 (23.9–32.6)		
	2030	16.8 (13.9–20.4)			29.2 (24.9–34.0)		
	2050	17.0 (13.4–21.0)			30.3 (25.2–36.0)		
High-income Western Europe	1990	11.4 (10.5–12.5)	48 (32–66)	15 (10–20)	16.1 (15.0–17.3)	33 (19–46)	12 (7–15)
	2021	16.9 (15.7–18.3)			21.3 (19.9–22.8)		
	2030	17.9 (16.6–19.4)			22.4 (21.0–23.9)		
	2050	19.5 (17.8–21.1)			23.8 (22.2–25.4)		
High-income Southern Latin America	1990	14.1 (11.3–17.4)	49 (11–92)	11 (3–18)	17.2 (14.0–20.6)	48 (16–87)	5 (-6–13)
	2021	20.7 (17.6–24.3)			25.2 (21.6–28.9)		
	2030	21.7 (18.6–25.9)			26.1 (22.4–30.0)		
	2050	22.9 (19.7–26.8)			26.4 (22.3–30.5)		
High-income North America	1990	15.6 (14.2–16.9)	38 (18–63)	3 (-5–11)	22.5 (21.3–23.9)	8 (-5–21)	-1 (-7–5)
	2021	21.5 (18.8–24.4)			24.2 (21.8–26.6)		
	2030	21.7 (18.9–24.8)			24.1 (21.5–26.5)		
	2050	22.1 (18.6–25.6)			23.9 (21.0–26.6)		
**LATIN AMERICA AND CARIBBEAN**	**1990**	**14.8 (13.3–16.6)**	**48 (28, 68)**	**8**	**14.1 (12.7–15.7)**	**62 (38–85)**	**6 (-1–12)**
	**2021**	**22.0 (20.0–24.1)**			**22.8 (20.8–24.9)**		

	**2030**	**23.0 (20.9–25.4)**		**(1, 15)**	**24.1 (21.9–26.2)**		
	**2050**	**23.5 (21.0–26.1)**			**24.2 (21.5–26.7)**		
Caribbean	1990	14.2 (12.4–16.2)	30 (8–54)	13 (5–22)	12.5 (11.0–14.1)	36 (16–59)	23 (13–31)
	2021	18.3 (16.2–20.5)			17.0 (15.3–18.8)		
	2030	19.1 (16.8–21.5)			18.2 (16.3–20.1)		
	2050	20.7 (18.0–23.7)			20.9 (18.3–23.4)		
Andean Latin America	1990	16.6 (13.4–20.2)	56 (21–97)	14 (-7–30)	14.5 (12.1–17.4)	65 (33–104)	17 (-5–33)
	2021	25.6 (22.3–29.2)			23.8 (21.4–26.4)		
	2030	27.0 (23.0–31.2)			25.4 (22.6–28.4)		
	2050	29.1 (22.2–35.4)			27.7 (21.7–33.0)		
Central Latin America	1990	17.6 (15.4–20.1)	43 (20–70)	3 (-7–11)	16.6 (14.3–19.0)	52 (25–81)	0 (-10–11)
	2021	25.1 (22.5–27.9)			25.1 (22.4–27.8)		
	2030	26.0 (23.2–28.9)			26.0 (23.3–28.9)		
	2050	25.9 (22.6–29.2)			25.1 (21.7–29.2)		
Tropical Latin America	1990	11.4 (8.4–14.7)	57 (5–121)	11 (2–20)	11.6 (9.0–14.9)	84 (27–154)	8 (-5–19)
	2021	17.6 (13.3–22.4)			21.0 (16.9–25.9)		
	2030	18.6 (14.0–23.7)			22.5 (18.2–27.5)		
	2050	19.4 (14.7–24.8)			22.7 (18.0–28.1)		
**NORTH AFRICA AND MIDDLE EAST**	**1990**	**15.2 (13.8–16.8)**	**57 (39, 76)**	**-5 (-13, 2)**	**10.7 (9.8–11.8)**	**87 (66–108)**	**2 (-8–11)**
	**2021**	**23.9 (22.4–25.3)**			**20.0 (18.6–21.4)**		
	**2030**	**24.3 (22.7–25.8)**			**21.0 (19.5–22.6)**		
	**2050**	**22.5 (20.2–25.0)**			**20.5 (18.1–22.6)**		
**CENTRAL EUROPE, EASTERN EUROPE, AND CENTRAL ASIA**	**1990**	**9.0 (8.0–10.0)**	**51 (27, 75)**	**22 (9, 30)**	**11.8 (10.7–12.9)**	**58 (39–78)**	**20 (8–27)**
	**2021**	**13.4 (12.1–14.9)**			**18.5 (17.0–20.1)**		
	**2030**	**14.3 (12.6–16.0)**			**19.6 (17.9–21.5)**		
	**2050**	**16.4 (14.1–18.4)**			**22.1 (19.6–24.6)**		
Central Asia	1990	10.2 (8.9–11.7)	54 (28–85)	24 (11–35)	12.5 (11.2–14.1)	44 (23–68)	22 (11–32)
	2021	15.5 (13.8–17.6)			18.0 (16.3–19.7)		
	2030	16.6 (14.8–18.6)			19.2 (17.4–21.1)		
	2050	19.3 (16.2–22.3)			21.9 (19.1–25.2)		
Central Europe	1990	8.0 (6.9–9.2)	56 (29–88)	15 (5–30)	13.2 (11.9–14.7)	51 (30–72)	13 (7–24)
	2021	12.4 (10.8–14.0)			19.9 (18.0–21.6)		
	2030	13.1 (11.4–14.9)			20.9 (19.0–22.8)		
	2050	14.2 (12.2–16.9)			22.5 (20.2–25.5)		
Eastern Europe	1990	9.0 (7.4–10.8)	41 (4–89)	15 (-7–25)	10.6 (8.8–12.7)	73 (32–120)	23 (-2–36)
	2021	12.7 (10.2–15.7)			18.1 (15.0–21.6)		
	2030	13.4 (10.4–16.7)			19.3 (15.8–23.1)		
	2050	14.7 (10.6–18.7)			22.1 (17.0–27.4)		

**SUB-SAHARAN AFRICA**	**1990**	**8.4 (7.7–9.1)**	**51 (35, 69)**	**29 (22, 34)**	**4.9 (4.4–5.5)**	**100 (73–127)**	**60 (53–67)**
	**2021**	**12.7 (11.8–13.7)**			**9.8 (8.9–10.8)**		
	**2030**	**14.1 (13.1–15.2)**			**11.6 (10.5–12.8)**		
	**2050**	**16.5 (15.1–17.9)**			**15.6 (14.2–17.2)**		
Central sub-Saharan Africa	1990	6.5 (5.1–8.2)	118 (59–187)	69 (40–85)	2.8 (2.3–3.4)	139 (85–203)	72 (45–91)
	2021	13.9 (11.2–17.0)			6.5 (5.4–7.8)		
	2030	16.8 (13.5–20.4)			8.2 (6.5–10.1)		
	2050	23.5 (17.7–28.7)			11.4 (8.5–14.4)		
Eastern sub-Saharan Africa	1990	7.4 (6.5–8.4)	55 (33–81)	33 (26–42)	4.1 (3.6–4.5)	102 (72–135)	66 (56–81)
	2021	11.4 (10.4–12.6)			8.1 (7.3–9.1)		
	2030	12.7 (11.6–14.1)			9.8 (8.8–10.8)		
	2050	15.2 (13.6–16.9)			13.5 (12.0–15.3)		
Southern sub-Saharan Africa	1990	14.0 (11.5–16.6)	46 (13–85)	4 (-5–13)	6.0 (4.7–7.5)	63 (15–116)	19 (0–32)
	2021	20.4 (17.4–23.7)			9.6 (7.6–11.9)		
	2030	21.0 (17.9–24.2)			10.4 (8.1–12.8)		
	2050	21.2 (18.1–24.4)			11.5 (8.7–14.0)		
Western sub-Saharan Africa	1990	8.3 (7.1–9.8)	50 (20–81)	25 (19–30)	6.1 (5.1–7.4)	103 (57–153)	52 (40–62)
	2021	12.4 (10.8–14.1)			12.3 (10.4–14.5)		
	2030	13.6 (11.9–15.5)			14.4 (12.1–16.8)		
	2050	15.5 (13.6–17.6)			18.6 (15.8–21.4)		
**SOUTHEAST ASIA, EAST ASIA, AND OCEANIA**	**1990**	**5.0 (4.6–5.3)**	**128 (107, 152)**	**49 (34, 57)**	**5.5 (5.2–5.9)**	**115 (99–133)**	**38 (23–46)**
	**2021**	**11.3 (10.6–12.1)**			**11.9 (11.3–12.5)**		
	**2030**	**13.3 (12.2–14.2)**			**14.2 (13.2–15.0)**		
	**2050**	**16.4 (14.6–17.9)**			**16.4 (14.5–17.7)**		
East Asia	1990	4.8 (4.4–5.2)	153 (127–183)	59 (26–76)	5.9 (5.5–6.3)	141 (120–163)	45 (17–64)
	2021	12.0 (11.3–12.8)			14.3 (13.5–15.2)		
	2030	14.5 (13.0–15.6)			17.0 (15.4–18.1)		
	2050	19.0 (14.9–21.2)			20.8 (16.5–23.7)		
Southeast Asia	1990	5.4 (4.6–6.3)	90 (52–133)	35 (23–50)	4.3 (3.7–4.8)	98 (64–137)	42 (28–54)
	2021	10.2 (8.8–11.7)			8.4 (7.4–9.4)		
	2030	11.3 (9.7–12.9)			9.5 (8.4–10.7)		
	2050	13.8 (11.6–16.2)			11.9 (10.2–13.9)		
Oceania	1990	15.4 (12.8–18.3)	27 (-7–68)	23 (15–43)	11.7 (10.0–13.8)	44 (11–82)	39 (30–64)
	2021	19.4 (15.7–24.2)			16.7 (13.7–19.9)		
	2030	20.7 (16.6–25.8)			18.7 (15.7–22.3)		
	2050	23.7 (18.2–30.2)			23.3 (18.7–29.6)		
**SOUTH ASIA**	**1990**	**4.4 (3.4–5.6)**	**114 (47, 200)**	**52**	**4.4 (3.4–5.6)**	**131 (64–220)**	**59 (46–71)**
	**2021**	**9.2 (7.3–11.5)**			**10.1 (8.1–12.6)**		

2030	10.6 (8.4–13.0)	(36, 66)	11.7 (9.6–14.4)
2050	13.6 (10.7–16.9)		15.9 (12.9–19.5)

Table S11: Age-standardised prevalence of children and young adolescents (5-14 years) with obesity (not overweight), globally and within each super-region and region, by sex

		Females			Males		
		Prevalence % (95% UI)	Percent change of OVOB 1990-2021 (95% UI)	Percent change of OVOB 2021-2050 (95% UI)	Prevalence % (95% UI)	Percent change of OVOB 1990-2021 (95% UI)	Percent change of OVOB 2021-2050 (95% UI)
**GLOBAL**	**1990**	**2.1 (1.9–2.2)**	**223 (192–255)**	**120 (82–141)**	**2.0 (1.9–2.2)**	**251 (220–281)**	**131 (89–154)**
	**2021**	**6.6 (6.2–7.0)**			**7.2 (6.8–7.5)**		
	**2030**	**8.8 (8.0–9.4)**			**9.4 (8.6–10.1)**		
	**2050**	**14.6 (11.8–16.3)**			**16.5 (13.3–18.3)**		
**HIGH-INCOME**	**1990**	**5.1 (4.1–6.2)**	**131 (78–186)**	**67 (44–83)**	**5.4 (4.5–6.5)**	**131 (86–180)**	**63 (42–77)**
	**2021**	**11.6 (10.3–13.1)**			**12.4 (11.2–13.6)**		
	**2030**	**13.8 (12.1–15.5)**			**14.6 (13.0–16.2)**		
	**2050**	**19.3 (15.9–22.2)**			**20.2 (16.8–22.8)**		
High-income Asia Pacific	1990	1.6 (1.2–2.0)	222 (112–388)	115 (63–161)	2.3 (1.9–2.9)	215 (120–325)	102 (61–136)
	2021	4.9 (3.6–6.6)			7.3 (5.7–9.3)		
	2030	6.4 (4.8–8.5)			9.3 (7.2–11.7)		
	2050	10.4 (7.3–14.5)			14.7 (10.4–19.2)		
High-income Australasia	1990	7.2 (5.2–9.7)	145 (61–258)	71 (44–95)	5.5 (4.2–7.1)	176 (91–284)	83 (51–111)
	2021	17.2 (12.7–22.2)			14.9 (11.7–19.0)		
	2030	20.6 (15.6–26.6)			18.3 (14.1–23.1)		
	2050	29.1 (21.0–37.7)			27.1 (19.4–34.3)		
High-income Western Europe	1990	3.9 (3.4–4.5)	150 (108–195)	77 (60–88)	4.3 (3.7–5.0)	143 (103–183)	70 (56–80)
	2021	9.6 (8.7–10.8)			10.4 (9.4–11.5)		
	2030	11.8 (10.7–12.9)			12.5 (11.3–13.8)		
	2050	17.1 (14.8–19.0)			17.6 (15.4–19.7)		
High-income Southern Latin America	1990	3.7 (2.7–4.9)	185 (99–291)	103 (45–146)	3.6 (2.7–4.6)	249 (148–367)	105 (39–151)
	2021	10.3 (8.1–12.9)			12.3 (9.9–15.1)		
	2030	13.1 (10.1–16.4)			15.7 (11.9–19.6)		
	2050	20.9 (13.4–27.5)			25.2 (15.7–33.3)		
High-income North America	1990	8.8 (6.1–12.3)	84 (17–168)	44 (21–64)	9.1 (6.4–12.5)	82 (23–167)	42 (21–56)
	2021	15.7 (12.5–19.5)			16.1 (13.0–19.2)		
	2030	17.6 (14.0–21.7)			18.0 (14.5–21.7)		
	2050	22.6 (17.0–28.7)			22.8 (17.5–28.0)		
**LATIN AMERICA AND CARIBBEAN**	**1990**	**3.5 (2.8–4.1)**	**223 (154–308)**	**114 (70–140)**	**3.0 (2.5–3.6)**	**317 (232–415)**	**136 (84–168)**
	**2021**	**11.1 (9.6–12.9)**			**12.4 (10.8–14.2)**		

	**2030**	**14.2 (11.9–16.4)**			**16.7 (13.9–19.6)**		
	**2050**	**23.6 (17.5–27.9)**			**29.3 (21.4–34.8)**		
Caribbean	1990	4.5 (3.8–5.3)	132 (86–182)	76 (30–100)	2.7 (2.3–3.2)	159 (110–219)	104 (47–136)
	2021	10.3 (8.9–11.9)			7.1 (6.1–8.0)		
	2030	12.0 (9.9–14.3)			8.4 (7.0–9.8)		
	2050	18.3 (13.0–22.5)			14.4 (10.1–17.5)		
Andean Latin America	1990	3.3 (2.4–4.4)	217 (113–347)	94 (61–122)	2.8 (2.1–3.8)	266 (157–395)	112 (71–146)
	2021	10.2 (8.2–12.6)			10.1 (8.2–12.4)		
	2030	12.7 (10.2–15.7)			13.0 (10.4–15.7)		
	2050	19.7 (14.9–24.5)			21.3 (15.8–26.2)		
Central Latin America	1990	3.6 (2.8–4.8)	217 (121–326)	113 (67–142)	3.3 (2.5–4.2)	305 (200–436)	130 (74–166)
	2021	11.2 (9.4–13.3)			13.0 (11.1–15.1)		
	2030	14.4 (12.1–17.0)			17.3 (14.5–20.0)		
	2050	23.8 (17.8–28.4)			29.8 (21.6–35.4)		
Tropical Latin America	1990	3.1 (2.0–4.6)	275 (118–516)	134 (82–178)	2.8 (1.9–3.9)	403 (223–672)	156 (96–204)
	2021	11.3 (7.8–15.7)			13.6 (9.9–18.1)		
	2030	15.0 (10.4–20.6)			19.1 (14.0–24.7)		
	2050	25.9 (17.2–34.3)			34.8 (23.4–44.2)		
**NORTH AFRICA AND MIDDLE EAST**	**1990**	**3.6 (3.1–4.2)**	**371 (295–451)**	**113 (69–142)**	**2.3 (2.0–2.7)**	**553 (460–662)**	**142 (93–174)**
	**2021**	**17.0 (15.2–18.6)**			**15.2 (13.8–16.7)**		
	**2030**	**22.5 (19.4–25.2)**			**20.9 (18.6–23.1)**		
	**2050**	**36.0 (28.0–41.9)**			**36.7 (29.1–42.0)**		
**CENTRAL EUROPE, EASTERN EUROPE, AND CENTRAL ASIA**	**1990**	**3.2 (2.8–3.7)**	**122 (78–175)**	**63 (36–82)**	**3.7 (3.3–4.1)**	**121 (86–164)**	**59 (30–81)**
	**2021**	**7.0 (5.9–8.3)**			**8.1 (7.0–9.4)**		
	**2030**	**8.1 (6.9–9.6)**			**9.3 (8.0–10.8)**		
	**2050**	**11.4 (9.0–13.6)**			**12.9 (9.9–15.4)**		
Central Asia	1990	2.5 (2.0–3.1)	134 (76–209)	67 (58–89)	3.1 (2.7–3.7)	100 (61–147)	54 (44–76)
	2021	5.8 (4.9–7.0)			6.2 (5.3–7.3)		
	2030	6.9 (5.7–8.3)			7.2 (6.0–8.5)		
	2050	9.7 (8.1–11.7)			9.6 (8.1–11.7)		
Central Europe	1990	2.9 (2.4–3.4)	157 (109–212)	72 (56–87)	4.5 (3.9–5.2)	116 (80–160)	54 (46–66)
	2021	7.3 (6.3–8.5)			9.7 (8.5–11.1)		
	2030	8.8 (7.6–10.1)			11.2 (9.9–12.8)		
	2050	12.6 (10.7–14.6)			14.9 (13.0–17.0)		
Eastern Europe	1990	3.6 (2.9–4.5)	114 (45–206)	66 (20–93)	3.4 (2.7–4.2)	156 (82–248)	83 (24–117)
	2021	7.7 (5.4–10.3)			8.7 (6.5–11.2)		
	2030	9.0 (6.3–12.3)			10.5 (7.7–13.6)		
	2050	12.6 (7.5–18.0)			15.8 (9.3–21.5)		

**SUB-SAHARAN AFRICA**	**1990**	**1.8 (1.6–2.0)**	**165 (128–208)**	**112 (91–125)**	**1.2 (1.1–1.3)**	**295 (244–350)**	**168 (139–185)**
	**2021**	**4.8 (4.3–5.3)**			**4.7 (4.3–5.1)**		
	**2030**	**6.2 (5.5–6.9)**			**6.5 (5.8–7.2)**		
	**2050**	**10.2 (8.8–11.6)**			**12.5 (10.8–14.0)**		
Central sub-Saharan Africa	1990	1.6 (1.2–2.2)	338 (200–512)	185 (131–219)	1.4 (1.0–1.8)	460 (294–659)	198 (146–234)
	2021	7.1 (5.4–9.1)			7.6 (6.0–9.4)		
	2030	10.2 (7.8–12.9)			11.1 (9.0–13.9)		
	2050	20.1 (14.6–25.1)			22.6 (17.5–27.6)		
Eastern sub-Saharan Africa	1990	1.7 (1.5–2.0)	146 (98–199)	112 (100–125)	1.1 (1.0–1.3)	278 (220–346)	168 (149–195)
	2021	4.2 (3.7–4.9)			4.2 (3.7–4.7)		
	2030	5.4 (4.8–6.2)			5.7 (5.1–6.5)		
	2050	9.0 (7.9–10.3)			11.2 (9.8–12.9)		
Southern sub-Saharan Africa	1990	3.5 (2.5–4.7)	216 (101–376)	99 (46–140)	1.9 (1.3–2.7)	310 (163–505)	114 (51–159)
	2021	10.6 (8.2–13.3)			7.6 (5.9–9.7)		
	2030	13.3 (10.2–16.7)			9.8 (7.4–12.4)		
	2050	21.0 (14.6–27.0)			16.2 (10.7–21.5)		
Western sub-Saharan Africa	1990	1.5 (1.3–1.9)	160 (94–242)	102 (83–117)	1.0 (0.9–1.3)	288 (196–395)	177 (141–201)
	2021	3.9 (3.2–4.9)			3.9 (3.2–4.8)		
	2030	5.0 (4.1–6.2)			5.5 (4.6–6.7)		
	2050	8.0 (6.4–10.0)			10.8 (8.8–13.0)		
**SOUTHEAST ASIA, EAST ASIA, AND OCEANIA**	**1990**	**0.9 (0.8–1.0)**	**407 (346–472)**	**180 (78–236)**	**1.4 (1.3–1.5)**	**399 (349–448)**	**139 (50–183)**
	**2021**	**4.7 (4.3–5.1)**			**7.0 (6.6–7.5)**		
	**2030**	**6.6 (5.6–7.5)**			**9.2 (7.7–10.1)**		
	**2050**	**13.1 (8.1–15.9)**			**16.5 (10.2–19.9)**		
East Asia	1990	0.8 (0.7–0.9)	539 (461–631)	243 (71–322)	1.6 (1.5–1.8)	460 (403–523)	180 (53–234)
	2021	5.0 (4.6–5.5)			9.1 (8.5–9.8)		
	2030	7.8 (6.0–8.9)			12.8 (10.4–14.3)		
	2050	17.2 (8.4–21.7)			24.8 (13.4–30.2)		
Southeast Asia	1990	1.1 (0.9–1.4)	263 (176–363)	126 (84–170)	1.0 (0.8–1.2)	277 (191–375)	122 (80–164)
	2021	4.0 (3.4–4.8)			3.7 (3.1–4.4)		
	2030	5.3 (4.3–6.4)			4.8 (4.0–5.6)		
	2050	9.1 (6.7–11.6)			8.1 (6.1–10.3)		
Oceania	1990	4.6 (3.9–5.5)	76 (38–123)	59 (43–93)	2.7 (2.4–3.0)	91 (60–128)	97 (69–154)
	2021	8.0 (6.7–9.7)			5.1 (4.4–5.9)		
	2030	8.9 (7.3–10.8)			5.9 (4.9–7.2)		
	2050	12.7 (9.9–16.1)			10.0 (7.6–13.9)		
**SOUTH ASIA**	**1990**	0.9 (0.6–1.2)	**292 (142–511)**	**154 (97–194)**	**0.9 (0.6–1.3)**	**274 (132–470)**	**159 (102–195)**
	**2021**	3.2 (2.3–4.4)			**3.2 (2.4–4.2)**		

**2030**	4.6 (3.4–6.1)	**4.4 (3.3–5.7)**
**2050**	8.4 (5.6–11.6)	**8.3 (5.7–10.9)**

Table S12: Age-standardised prevalence of older adolescents (15-24 years) with obesity (not overweight), globally and within each super-region and region, by sex

		Females			Males		
		Prevalence % (95% UI)	Percent change of OVOB 1990-2021 (95% UI)	Percent change of OVOB 2021-2050 (95% UI)	Prevalence % (95% UI)	Percent change of OVOB 1990-2021 (95% UI)	Percent change of OVOB 2021-2050 (95% UI)
**GLOBAL**	**1990**	**2.1 (2.0–2.3)**	**241 (220–263)**	**106 (71–125)**	**1.6 (1.5–1.7)**	**270 (246–295)**	**125 (78–150)**
	**2021**	**7.3 (7.0–7.6)**			**5.9 (5.6–6.2)**		
	**2030**	**9.5 (8.7–10.1)**			**7.9 (7.1–8.5)**		
	**2050**	**15.2 (12.4–16.8)**			**13.3 (10.4–14.9)**		
**HIGH-INCOME**	**1990**	**5.3 (4.9–5.7)**	**210 (177–244)**	**53 (35–66)**	**4.7 (4.4–4.9)**	**189 (162–216)**	**58 (39–71)**
	**2021**	**16.4 (15.2–17.7)**			**13.4 (12.5–14.4)**		
	**2030**	**18.8 (17.1–20.4)**			**15.6 (14.4–16.8)**		
	**2050**	**25.1 (21.4–27.9)**			**21.3 (18.1–23.6)**		
High-income Asia Pacific	1990	0.7 (0.6–1.0)	232 (115–395)	153 (82–219)	1.3 (1.0–1.6)	240 (139–377)	133 (78–178)
	2021	2.4 (1.8–3.2)			4.2 (3.3–5.3)		
	2030	3.3 (2.5–4.3)			5.7 (4.4–7.1)		
	2050	6.1 (4.0–8.6)			9.9 (6.9–13.1)		
High-income Australasia	1990	7.4 (5.8–9.2)	163 (92–244)	65 (41–87)	5.1 (4.1–6.4)	180 (105–269)	80 (50–106)
	2021	19.2 (15.9–22.8)			14.2 (11.6–17.3)		
	2030	22.9 (19.3–27.1)			17.5 (14.3–20.7)		
	2050	31.7 (24.8–38.2)			25.6 (19.4–31.7)		
High-income Western Europe	1990	4.3 (3.8–4.8)	146 (112–184)	74 (58–85)	3.7 (3.3–4.2)	134 (101–170)	75 (58–86)
	2021	10.5 (9.6–11.4)			8.7 (8.0–9.6)		
	2030	12.6 (11.6–13.8)			10.6 (9.7–11.6)		
	2050	18.2 (15.9–20.1)			15.2 (13.5–16.8)		
High-income Southern Latin America	1990	4.7 (3.5–6.4)	225 (126–341)	83 (37–116)	3.3 (2.5–4.3)	286 (180–429)	101 (37–146)
	2021	15.0 (12.4–17.9)			12.5 (10.5–14.9)		
	2030	18.4 (14.7–21.8)			15.9 (12.4–19.2)		
	2050	27.4 (19.2–33.8)			25.2 (15.9–32.2)		
High-income North America	1990	9.9 (8.9–11.0)	181 (139–229)	31 (15–44)	8.5 (7.8–9.3)	157 (125–195)	34 (18–46)
	2021	27.7 (24.7–30.9)			21.9 (19.7–24.1)		
	2030	30.2 (26.6–34.2)			24.1 (21.4–26.7)		
	2050	36.2 (29.8–42.0)			29.4 (24.3–33.3)		
**LATIN AMERICA AND CARIBBEAN**	**1990**	**4.6 (3.9–5.2)**	**219 (165–275)**	**98 (60–119)**	**2.8 (2.3–3.3)**	**354 (265–461)**	**136 (81–168)**
	**2021**	**14.4 (12.9–16.0)**			**12.5 (11.0–14.0)**		

	**2030**	**18.5 (16.1–21.0)**			**17.1 (14.7–19.6)**		
	**2050**	**28.6 (22.4–33.2)**			**29.4 (21.9–34.5)**		
Caribbean	1990	5.9 (5.0–6.9)	131 (86–181)	65 (28–86)	2.9 (2.4–3.5)	170 (113–235)	89 (34–119)
	2021	13.5 (12.0–15.2)			7.8 (6.8–8.9)		
	2030	15.9 (13.5–18.2)			9.6 (8.1–11.1)		
	2050	22.3 (16.7–26.4)			14.6 (10.1–17.7)		
Andean Latin America	1990	3.4 (2.5–4.4)	229 (140–334)	92 (60–116)	2.2 (1.7–2.8)	293 (188–420)	120 (78–149)
	2021	10.8 (9.2–12.6)			8.5 (7.4–9.7)		
	2030	13.6 (11.4–15.9)			11.1 (9.4–12.7)		
	2050	20.9 (16.2–24.9)			18.7 (14.2–22.2)		
Central Latin America	1990	5.6 (4.6–6.7)	205 (142–275)	90 (55–113)	3.5 (2.8–4.4)	323 (220–444)	122 (70–156)
	2021	16.8 (14.7–19.1)			14.6 (12.5–16.7)		
	2030	21.3 (18.3–24.1)			19.8 (16.5–22.8)		
	2050	31.9 (24.8–36.9)			32.4 (24.2–37.9)		
Tropical Latin America	1990	3.4 (2.3–4.8)	287 (134–479)	125 (77–168)	2.1 (1.4–3.0)	509 (265–853)	170 (102–226)
	2021	12.8 (9.5–16.5)			12.2 (9.3–15.8)		
	2030	17.0 (12.7–21.7)			17.4 (13.1–22.4)		
	2050	28.7 (20.1–36.7)			32.8 (22.6–41.7)		
**NORTH AFRICA AND MIDDLE EAST**	**1990**	**4.6 (4.0–5.2)**	**300 (241–366)**	**105 (65–132)**	**2.0 (1.8–2.3)**	**562 (472–658)**	**153 (97–188)**
	**2021**	**18.3 (17.1–19.7)**			**13.3 (12.4–14.3)**		
	**2030**	**24.3 (21.6–26.5)**			**18.9 (16.6–20.7)**		
	**2050**	**37.7 (29.8–42.7)**			**33.8 (25.9–38.9)**		
**CENTRAL EUROPE, EASTERN EUROPE, AND CENTRAL ASIA**	**1990**	**2.7 (2.3–3.2)**	**146 (101–200)**	**61 (34–77)**	**2.2 (2.0–2.5)**	**156 (117–200)**	**66 (35–87)**
	**2021**	**6.6 (5.8–7.6)**			**5.7 (5.1–6.4)**		
	**2030**	**7.8 (6.7–9.1)**			**6.7 (5.8–7.8)**		
	**2050**	**10.7 (8.4–12.6)**			**9.4 (7.3–11.3)**		
Central Asia	1990	1.9 (1.6–2.3)	149 (96–217)	69 (58–91)	2.0 (1.7–2.3)	123 (79–175)	60 (52–82)
	2021	4.7 (4.0–5.6)			4.4 (3.8–5.1)		
	2030	5.6 (4.8–6.6)			5.1 (4.4–5.9)		
	2050	8.0 (6.8–9.5)			7.0 (6.0–8.4)		
Central Europe	1990	3.0 (2.5–3.5)	153 (103–209)	72 (56–88)	3.4 (2.9–3.9)	120 (81–166)	60 (50–75)
	2021	7.4 (6.4–8.5)			7.4 (6.6–8.4)		
	2030	9.0 (7.7–10.1)			8.6 (7.7–9.7)		
	2050	12.8 (10.7–14.5)			11.8 (10.5–13.4)		
Eastern Europe	1990	2.9 (2.3–3.7)	163 (80–278)	64 (17–89)	1.7 (1.3–2.1)	245 (139–381)	90 (23–127)
	2021	7.5 (5.7–9.5)			5.6 (4.3–7.3)		
	2030	8.7 (6.6–11.3)			6.9 (5.0–9.0)		
	2050	12.2 (7.6–16.6)			10.7 (5.8–14.9)		

**SUB-SAHARAN AFRICA**	**1990**	2.0 (1.8–2.3)	**143 (109–185)**	**99 (79–112)**	**0.6 (0.5–0.7)**	**294 (231–367)**	**203 (161–228)**
	**2021**	4.9 (4.5–5.4)			**2.3 (2.0–2.5)**		
	**2030**	6.3 (5.6–6.9)			**3.3 (2.9–3.7)**		
	**2050**	9.8 (8.4–11.1)			**6.8 (5.7–7.9)**		
Central sub-Saharan Africa	1990	0.6 (0.5–0.8)	356 (237–506)	222 (151–259)	0.3 (0.2–0.4)	606 (358–901)	302 (215–354)
	2021	2.9 (2.3–3.5)			2.2 (1.7–2.8)		
	2030	4.3 (3.3–5.4)			3.5 (2.6–4.5)		
	2050	9.3 (6.8–11.6)			8.7 (6.3–11.4)		
Eastern sub-Saharan Africa	1990	1.3 (1.1–1.6)	168 (117–228)	118 (104–131)	0.4 (0.3–0.5)	314 (241–405)	213 (181–245)
	2021	3.5 (3.1–4.0)			1.6 (1.4–1.8)		
	2030	4.6 (4.0–5.3)			2.4 (2.1–2.7)		
	2050	7.7 (6.7–8.7)			5.0 (4.3–5.8)		
Southern sub-Saharan Africa	1990	6.2 (4.7–7.8)	192 (114–295)	77 (42–104)	1.6 (1.2–2.2)	234 (117–397)	132 (58–184)
	2021	17.9 (15.0–21.0)			5.2 (4.0–7.0)		
	2030	21.9 (18.0–25.9)			6.9 (5.0–9.2)		
	2050	31.7 (24.1–37.7)			12.1 (7.3–16.5)		
Western sub-Saharan Africa	1990	1.9 (1.6–2.3)	162 (98–240)	99 (82–112)	0.5 (0.4–0.7)	366 (239–517)	202 (155–230)
	2021	4.9 (4.1–5.9)			2.5 (2.0–3.1)		
	2030	6.3 (5.2–7.6)			3.6 (2.9–4.4)		
	2050	9.8 (8.0–11.9)			7.4 (5.8–9.1)		
**SOUTHEAST ASIA, EAST ASIA, AND OCEANIA**	**1990**	**0.8 (0.7–0.9)**	**491 (417–568)**	**177 (79–232)**	**0.9 (0.8–1.0)**	**451 (394–512)**	**162 (56–215)**
	**2021**	**4.6 (4.3–5.0)**			**4.8 (4.5–5.2)**		
	**2030**	**6.7 (5.6–7.5)**			**7.1 (5.8–7.9)**		
	**2050**	**12.9 (8.1–15.8)**			**12.6 (7.4–15.5)**		
East Asia	1990	0.7 (0.6–0.7)	615 (525–716)	248 (73–329)	1.0 (0.9–1.1)	579 (501–665)	206 (57–272)
	2021	4.7 (4.4–5.1)			6.5 (6.0–6.9)		
	2030	7.3 (5.8–8.3)			9.4 (7.5–10.6)		
	2050	16.5 (8.1–20.8)			19.6 (9.8–24.3)		
Southeast Asia	1990	1.1 (0.9–1.3)	316 (225–435)	119 (78–163)	0.6 (0.5–0.8)	298 (207–405)	136 (87–185)
	2021	4.4 (3.8–5.2)			2.5 (2.1–3.0)		
	2030	5.7 (4.8–6.7)			3.3 (2.8–4.0)		
	2050	9.8 (7.4–12.6)			5.9 (4.4–7.7)		
Oceania	1990	4.7 (4.1–5.3)	61 (28–98)	50 (34–80)	2.5 (2.2–2.8)	96 (58–143)	109 (84–173)
	2021	7.5 (6.3–8.9)			4.9 (4.1–5.8)		
	2030	8.7 (7.3–10.4)			6.2 (5.2–7.4)		
	2050	11.2 (8.7–14.9)			10.2 (7.9–14.1)		
**SOUTH ASIA**	**1990**	**0.5 (0.4–0.7)**	**369 (205–588)**	**174 (114–214)**	**0.5 (0.3–0.6)**	**402 (221–653)**	**179 (111–225)**
	**2021**	**2.4 (1.9–3.2)**			**2.2 (1.7–3.0)**		

**2030**	**3.6 (2.9–4.7)**	**3.2 (2.5–4.2)**
**2050**	**7.0 (5.0–9.3)**	**6.3 (4.3–8.6)**

Table S13: Age-standardised prevalence of children (5-9 years) with overweight or obesity in 1990, 2021, 2030, 2050, globally and within each super-region, by sex

		Both Sexes		Females		Males	
		Prevalence	Percent change in prevalence 1990-2021 2021-2050	Prevalence	Percent change in prevalence 1990-2021 2021-2050	Prevalence	Percent change in prevalence 1990-2021 2021-2050
Global	1990	8.5 (8.1–9.1)	103 (89–117)	9.1 (8.5–10.0)	99 (78–120)	8.0 (7.5–8.6)	108 (90–129)
	2021	17.4 (16.6–18.2)		18.1 (17.0–19.4)		16.7 (15.7–17.7)	
	2030	20.4 (19.1–21.6)	66 (47–76)	21.3 (19.6–23.0)	62 (43–72)	19.5 (18.0–20.9)	70 (50–82)
	2050	28.8 (25.2–31.2)		29.3 (25.4–32.2)		28.4 (24.5–30.9)	
High-income	1990	18.2 (16.2–20.4)	54 (33–77)	17.6 (14.7–21.1)	60 (30–96)	18.7 (16.0–21.8)	50 (24–79)
	2021	27.9 (25.8–30.1)		27.9 (24.5–31.5)		27.9 (25.2–30.7)	
	2030	30.9 (28.3–33.4)	35 (22–42)	31.2 (27.4–34.9)	38 (24–47)	30.6 (27.4–33.8)	32 (20–39)
	2050	37.5 (32.7–41.3)		38.4 (32.5–43.4)		36.7 (31.5–40.8)	
Latin America and Caribbean	1990	14.0 (12.1–16.0)	104 (70–143)	15.5 (12.6–18.9)	91 (47–147)	12.5 (10.3–15.1)	123 (76–178)
	2021	28.4 (25.6–31.5)		29.2 (25.0–33.9)		27.6 (24.0–31.6)	
	2030	33.2 (29.6–36.9)	60 (37–74)	33.6 (28.7–38.8)	53 (32–68)	32.8 (28.3–37.4)	66 (41–83)
	2050	45.3 (37.6–51.1)		44.7 (36.2–52.2)		45.9 (36.8–52.8)	
North Africa and Middle East	1990	12.4 (11.2–13.9)	157 (126–192)	15.0 (12.8–17.4)	135 (95–181)	10.0 (8.7–11.7)	190 (141–241)
	2021	31.9 (29.4–34.5)		35.1 (31.0–38.8)		28.9 (25.9–32.3)	
	2030	37.6 (34.3–40.9)	61 (39–77)	40.6 (36.2–45.5)	52 (33–68)	34.8 (31.2–38.9)	71 (45–91)
	2050	51.2 (43.6–57.6)		53.2 (44.9–60.2)		49.4 (41.7–56.0)	
Central Europe, Eastern Europe, and Central Asia	1990	14.6 (13.4–16.1)	58 (37–82)	14.7 (12.8–16.9)	59 (28–93)	14.6 (12.9–16.4)	59 (32–89)
	2021	23.1 (20.5–25.9)		23.2 (19.5–27.3)		23.1 (19.8–26.8)	
	2030	25.4 (22.7–28.4)	34 (19–44)	25.8 (21.7–29.9)	36 (21–48)	25.1 (21.5–29.0)	32 (16–44)
	2050	30.9 (26.4–35.4)		31.5 (25.8–36.9)		30.4 (24.6–36.0)	
Sub-Saharan Africa	1990	7.5 (6.9–8.1)	95 (73–118)	9.0 (8.0–10.2)	78 (50–110)	6.0 (5.4–6.7)	121 (91–155)
	2021	14.5 (13.4–15.9)		15.9 (14.1–17.9)		13.2 (11.8–14.8)	
	2030	17.1 (15.8–18.8)	68 (57–75)	18.3 (16.3–21.0)	54 (44–62)	16.0 (14.4–17.9)	84 (73–93)
	2050	24.4 (22.0–26.7)		24.5 (21.6–27.9)		24.2 (21.5–27.0)	
Southeast Asia, East Asia, and Oceania	1990	5.5 (5.1–5.9)	175 (151–199)	5.3 (4.7–5.9)	173 (138–216)	5.7 (5.2–6.2)	176 (146–207)
	2021	15.1 (14.2–16.0)		14.4 (13.1–15.8)		15.8 (14.6–16.9)	
	2030	17.6 (15.8–19.1)	81 (37–102)	17.3 (15.0–19.3)	87 (43–110)	18.0 (16.0–19.6)	75 (31–96)
	2050	27.2 (20.2–30.9)		26.9 (19.9–31.3)		27.5 (20.1–31.6)	
South Asia	1990	4.0 (3.0–5.4)	129 (52–233)	4.5 (2.9–7.0)	126 (21–280)	3.5 (2.4–5.1)	142 (41–284)
	2021	8.9 (6.8–11.7)		9.7 (6.5–14.2)		8.2 (5.7–11.7)	
	2030	11.1 (8.7–14.4)	86 (58–103)	11.9 (8.1–17.2)	81 (51–102)	10.2 (7.1–14.3)	92 (64–111)
	2050	16.7 (12.3–21.6)		17.6 (11.8–25.2)		15.8 (10.7–21.6)	

Table S14: Age-standardised prevalence of adolescents (10-14 years) with overweight or obesity in 1990, 2021, 2030, 2050, globally and within each super-region, by sex

		Both Sexes		Female		Male	
		Prevalence	Percent change in prevalence 1990-2021 2021-2050	Prevalence	Percent change in prevalence 1990-2021 2021-2050	Prevalence	Percent change in prevalence 1990-2021 2021-2050
Global	1990	9.0 (8.6–9.5)	109 (95–123)	9.3 (8.6–9.9)	107 (89–129)	8.8 (8.3–9.5)	110 (91–128)
	2021	18.8 (18.0–19.8)		19.2 (17.9–20.4)		18.5 (17.5–19.7)	
	2030	22.4 (21.0–23.6)	63 (45–73)	22.7 (21.0–24.5)	60 (43–71)	22.0 (20.6–23.5)	65 (46–76)
	2050	30.6 (26.8–33.1)		30.7 (26.7–33.7)		30.5 (26.6–33.3)	
High-income	1990	17.8 (16.3–19.6)	83 (61–105)	16.6 (14.4–19.4)	89 (55–127)	19.0 (16.9–21.4)	79 (54–106)
	2021	32.6 (30.5–34.9)		31.2 (27.7–34.7)		33.9 (31.1–36.8)	
	2030	35.6 (33.1–38.1)	31 (20–38)	34.5 (30.7–38.1)	35 (22–43)	36.7 (33.3–40.2)	28 (18–35)
	2050	42.6 (37.9–46.2)		42.0 (36.0–46.6)		43.3 (37.8–47.5)	
Latin America and Caribbean	1990	15.5 (13.6–17.6)	106 (73–139)	16.5 (13.6–19.8)	96 (56–145)	14.5 (12.1–17.2)	118 (74–169)
	2021	31.7 (28.7–34.7)		31.9 (27.5–36.5)		31.4 (27.5–35.4)	
	2030	36.5 (32.6–40.1)	53 (33–66)	36.3 (31.6–41.4)	49 (30–63)	36.6 (31.8–41.5)	58 (36–73)
	2050	48.5 (40.7–54.4)		47.3 (39.1–54.5)		49.6 (40.6–56.4)	
North Africa and Middle East	1990	14.0 (12.5–15.6)	154 (124–187)	16.7 (14.3–19.4)	133 (93–178)	11.4 (9.9–13.0)	185 (142–234)
	2021	35.4 (32.9–38.0)		38.8 (34.6–42.9)		32.3 (29.1–35.7)	
	2030	42.0 (38.5–45.4)	57 (38–72)	45.3 (40.3–50.0)	47 (31–63)	38.8 (34.9–43.0)	67 (46–84)
	2050	55.5 (48.1–61.4)		57.2 (49.5–63.8)		53.8 (45.7–60.5)	
Central Europe, Eastern Europe, and Central Asia	1990	12.3 (11.3–13.4)	73 (50–101)	10.9 (9.5–12.4)	80 (44–127)	13.7 (12.2–15.4)	69 (40–101)
	2021	21.3 (19.1–24.0)		19.5 (16.5–23.4)		23.0 (20.0–26.4)	
	2030	23.4 (21.1–26.2)	34 (19–44)	21.7 (18.5–25.6)	38 (21–49)	25.1 (22.0–29.1)	32 (16–43)
	2050	28.6 (24.6–32.6)		26.8 (22.2–31.8)		30.3 (25.5–35.5)	
Sub-Saharan Africa	1990	7.5 (6.9–8.2)	99 (77–125)	9.3 (8.1–10.5)	81 (54–114)	5.7 (5.0–6.5)	131 (97–171)
	2021	15.0 (13.7–16.5)		16.8 (14.8–19.2)		13.1 (11.7–15.0)	
	2030	17.7 (16.3–19.6)	67 (57–75)	19.4 (17.3–22.3)	53 (44–60)	16.1 (14.3–18.4)	86 (73–95)
	2050	25.0 (22.7–27.6)		25.7 (22.6–29.6)		24.4 (21.4–27.7)	
Southeast Asia, East Asia, and Oceania	1990	6.0 (5.6–6.4)	168 (146–192)	5.7 (5.1–6.2)	168 (133–205)	6.4 (5.8–6.9)	168 (140–198)
	2021	16.1 (15.2–17.1)		15.1 (13.7–16.6)		17.0 (15.7–18.3)	
	2030	20.0 (17.8–21.6)	79 (37–99)	18.9 (16.5–21.0)	87 (43–108)	21.0 (18.6–22.8)	74 (31–94)
	2050	28.8 (21.5–32.9)		28.1 (20.8–32.7)		29.4 (21.9–33.6)	
South Asia	1990	4.4 (3.3–5.7)	135 (62–227)	4.5 (3.0–6.5)	134 (31–287)	4.3 (2.9–6.2)	145 (45–286)
	2021	10.1 (7.7–13.0)		10.1 (6.9–14.4)		10.2 (7.1–14.0)	
	2030	12.5 (9.8–15.6)	85 (59–102)	12.5 (8.6–17.3)	83 (54–103)	12.5 (9.0–16.9)	89 (61–107)
	2050	18.7 (14.5–23.7)		18.3 (12.4–25.5)		19.0 (13.4–25.1)	

Table S15: Age-standardised prevalence of adolescents (15-19 years) with overweight or obesity in 1990, 2021, 2030, 2050, globally and within each super-region, by sex

		Both Sexes		Female		Male	
		Prevalence	Percent change in prevalence 1990-2021 2021-2050	Prevalence	Percent change in prevalence 1990-2021 2021-2050	Prevalence	Percent change in prevalence 1990-2021 2021-2050
Global	1990	7.7 (7.4–8.0)	114 (101–128)	8.0 (7.5–8.5)	119 (100–140)	7.3 (6.9–7.8)	109 (92–128)
	2021	16.4 (15.6–17.2)		17.5 (16.5–18.8)		15.3 (14.4–16.4)	
	2030	19.5 (18.3–20.6)	62 (42–74)	20.7 (19.3–22.2)	60 (41–71)	18.4 (17.1–19.7)	65 (44–78)
	2050	26.6 (23.2–28.7)		27.9 (24.2–30.6)		25.3 (21.7–28.0)	
High-income	1990	15.8 (15.0–16.6)	89 (75–103)	14.3 (13.2–15.4)	110 (86–134)	17.2 (16.2–18.3)	73 (57–90)
	2021	29.8 (28.1–31.4)		30.0 (27.6–32.4)		29.6 (27.7–31.8)	
	2030	32.3 (30.4–34.1)	32 (20–39)	32.5 (29.9–35.1)	34 (22–43)	32.1 (30.0–34.4)	30 (19–37)
	2050	39.2 (35.0–42.2)		40.1 (34.9–43.9)		38.5 (34.4–41.9)	
Latin America and Caribbean	1990	13.8 (12.2–15.5)	115 (84–150)	15.2 (12.9–18.0)	102 (62–147)	12.4 (10.4–14.7)	134 (86–191))
	2021	29.6 (26.8–32.6)		30.4 (26.7–34.4)		28.8 (25.1–32.8)	
	2030	34.6 (31.1–38.5)	55 (33–70)	35.0 (30.8–39.6)	50 (30–64)	34.1 (29.6–38.6)	61 (37–78)
	2050	45.9 (38.3–52.1)		45.4 (37.5–52.2)		46.4 (37.7–53.2)	
North Africa and Middle East	1990	11.6 (10.4–12.8)	162 (132–194)	14.4 (12.4–16.7)	144 (104–187)	8.9 (7.8–10.1)	192 (149–238)
	2021	30.2 (28.3–32.2)		34.9 (31.7–37.7)		25.9 (23.5–28.3)	
	2030	36.5 (33.8–39.4)	64 (40–82)	41.4 (37.6–45.0)	53 (32–69)	31.9 (28.4–35.4)	78 (51–100)
	2050	49.7 (42.4–55.9)		53.4 (45.8–59.5)		46.2 (38.3–53.0)	
Central Europe, Eastern Europe, and Central Asia	1990	9.2 (8.4–10.1)	82 (57–109)	8.6 (7.4–10.0)	85 (46–133)	9.8 (8.7–11.1)	80 (49–113)
	2021	16.8 (15.1–18.6)		15.9 (13.5–18.9)		17.6 (15.4–20.2)	
	2030	18.5 (16.4–20.6)	36 (20–46)	17.6 (15.0–21.2)	37 (21–48)	19.3 (16.8–22.2)	35 (18–46)
	2050	22.8 (19.3–26.0)		21.9 (17.9–26.4)		23.6 (19.4–27.8)	
Sub-Saharan Africa	1990	6.0 (5.5–6.6)	92 (69–116)	8.1 (7.2–9.1)	74 (47–102)	3.9 (3.4–4.4)	133 (90–178)
	2021	11.5 (10.6–12.6)		14.0 (12.6–15.6)		9.0 (7.8–10.2)	
	2030	13.8 (12.7–15.0)	70 (58–78)	16.4 (14.7–18.3)	54 (44–62)	11.2 (9.9–12.9)	95 (81–105)
	2050	19.5 (17.6–21.6)		21.6 (19.0–24.4)		17.5 (15.3–20.1)	
Southeast Asia, East Asia, and Oceania	1990	4.9 (4.5–5.2)	178 (154–204)	4.7 (4.2–5.3)	181 (143–225)	5.0 (4.6–5.4)	175 (147–210)
	2021	13.5 (12.7–14.2)		13.2 (12.1–14.5)		13.7 (12.7–14.8)	
	2030	17.3 (15.4–18.6)	85 (40–106)	16.8 (14.8–18.6)	91 (48–114)	17.7 (15.5–19.3)	80 (33–102)
	2050	24.9 (18.6–28.3)		25.2 (18.9–29.1)		24.6 (17.8–28.4)	
South Asia	1990	3.9 (3.0–5.1)	150 (71–249)	4.3 (2.9–6.1)	151 (46–308)	3.5 (2.4–5.1)	158 (53–298)
	2021	9.5 (7.5–12.1)		10.3 (7.2–14.1)		8.8 (6.2–12.3)	
	2030	11.7 (9.3–14.5)	84 (59–101)	12.7 (9.4–16.9)	80 (52–101)	10.9 (7.8–14.9)	90 (62–108)
	2050	17.5 (13.1–21.8)		18.3 (12.8–24.5)		16.7 (12.0–22.3)	

Table S16: Age-standardised prevalence of adolescents (20-24 years) with overweight or obesity in 1990, 2021, 2030, 2050, globally and within each super-region, by sex

		Both Sexes		Female		Male	
		**Prevalence**	**Percent change in prevalence** 1990-2021 2021-2050	**Prevalence**	**Percent change in prevalence** 1990-2021 2021-2050	**Prevalence**	**Percent change in prevalence** 1990-2021 2021-2050
Global	1990	12.2 (11.8–12.6)	102 (91–113)	12.0 (11.4–12.6)	106 (91–121)	12.4 (11.8–13.0)	98 (84–113)
	2021	24.5 (23.7–25.6)		24.6 (23.4–26.0)		24.5 (23.1–26.1)	
	2030	28.4 (27.0–29.9)	52 (37–60)	28.5 (26.8–30.2)	51 (37–60)	28.4 (26.6–30.3)	52 (37–62)
	2050	37.2 (33.4–39.9)		37.2 (33.2–40.2)		37.3 (33.1–40.4)	
High-income	1990	22.8 (21.8–23.8)	77 (66–87)	19.6 (18.2–20.9)	99 (83–118)	26.0 (24.7–27.5)	60 (48–73)
	2021	40.3 (38.7–41.9)		38.9 (36.6–41.2)		41.7 (39.5–43.8)	
	2030	43.9 (41.8–45.7)	27 (18–32)	42.5 (39.8–45.1)	30 (20–36)	45.1 (42.3–47.5)	25 (17–30)
	2050	51.1 (46.7–54.1)		50.3 (45.4–54.1)		51.9 (47.2–55.4)	
Latin America and Caribbean	1990	22.8 (20.8–25.0)	87 (64–111)	23.9 (20.9–27.2)	80 (50–110)	21.7 (19.0–24.7)	96 (66–132)
	2021	42.5 (39.4–45.6)		42.7 (38.5–47.4)		42.2 (38.4–46.3)	
	2030	48.5 (45.1–52.1)	41 (26–51)	48.3 (43.3–53.7)	38 (24–49)	48.7 (44.4–53.2)	45 (28–57)
	2050	60.1 (53.0–65.3)		59.0 (51.3–65.7)		61.1 (53.3–67.1)	
North Africa and Middle East	1990	21.1 (19.3–23.1)	116 (96–138)	25.6 (22.3–28.8)	96 (70–126)	16.8 (15.1–18.9)	146 (113–178)
	2021	45.5 (43.6–47.5)		50.0 (47.2–53.0)		41.2 (38.6–43.9)	
	2030	52.2 (49.2–54.8)	43 (29–54)	56.3 (52.3–59.9)	35 (23–46)	48.4 (44.7–51.6)	52 (36–66
	2050	65.0 (58.0–69.9)		67.5 (60.6–72.7)		62.8 (55.3–68.0)	
Central Europe, Eastern Europe, and Central Asia	1990	16.7 (15.4–18.2)	68 (48–87)	14.9 (12.9–17.2)	65 (35–98)	18.4 (16.3–20.6)	70 (45–98)
	2021	27.9 (25.9–30.1)		24.5 (21.6–27.9)		31.2 (28.3–34.5)	
	2030	30.4 (27.6–33.4)	30 (17–38)	26.8 (23.5–31.0)	33 (19–41)	33.8 (30.1–37.8)	28 (15–37)
	2050	36.3 (31.5–40.3)		32.5 (27.6–37.6)		39.9 (34.5–44.9)	
Sub-Saharan Africa	1990	10.2 (9.3–11.2)	81 (62–102)	13.0 (11.6–14.5)	66 (43–90)	7.2 (6.4–8.3)	113 (77–151)
	2021	18.4 (17.2–19.8)		21.4 (19.7–23.3)		15.3 (13.5–17.2)	
	2030	21.6 (20.1–23.2)	60 (51–66)	24.5 (22.4–26.9)	46 (38–52)	18.8 (16.7–21.0)	81 (70–90)
	2050	29.4 (26.8–31.8)		31.3 (28.2–34.1)		27.6 (24.8–30.6)	
Southeast Asia, East Asia, and Oceania	1990	7.4 (6.9–7.9)	163 (143–185)	6.8 (6.2–7.5)	176 (142–212)	7.9 (7.3–8.6)	153 (128–180)
	2021	19.4 (18.5–20.5)		18.8 (17.2–20.5)		20.0 (18.7–21.4)	
	2030	24.3 (22.0–25.9)	73 (39–91)	23.4 (20.7–25.7)	80 (46–99)	25.1 (22.6–27.0)	68 (32–87)
	2050	33.6 (26.7–37.6)		33.6 (26.5–38.5)		33.6 (26.1–37.8)	
South Asia	1990	6.0 (4.7–7.6)	148 (80–229)	5.6 (4.0–7.8)	141 (48–261)	6.3 (4.5–8.5)	160 (65–284)
	2021	14.6 (12.0–17.7)		13.1 (9.8–17.1)		16.0 (12.1–20.7)	
	2030	17.6 (14.7–21.5)	77 (54–91)	15.9 (11.9–20.6)	77 (52–94)	19.3 (14.5–24.4)	78 (55–95)
	2050	25.7 (20.6–31.6)		23.0 (17.2–30.2)		28.1 (20.8–35.1)	

Figures S2: Age-standardised prevalence estimates from 1990 to 2021 and forecasts to 2050 for children and young adolescents aged 5 to 14 years old, by sex

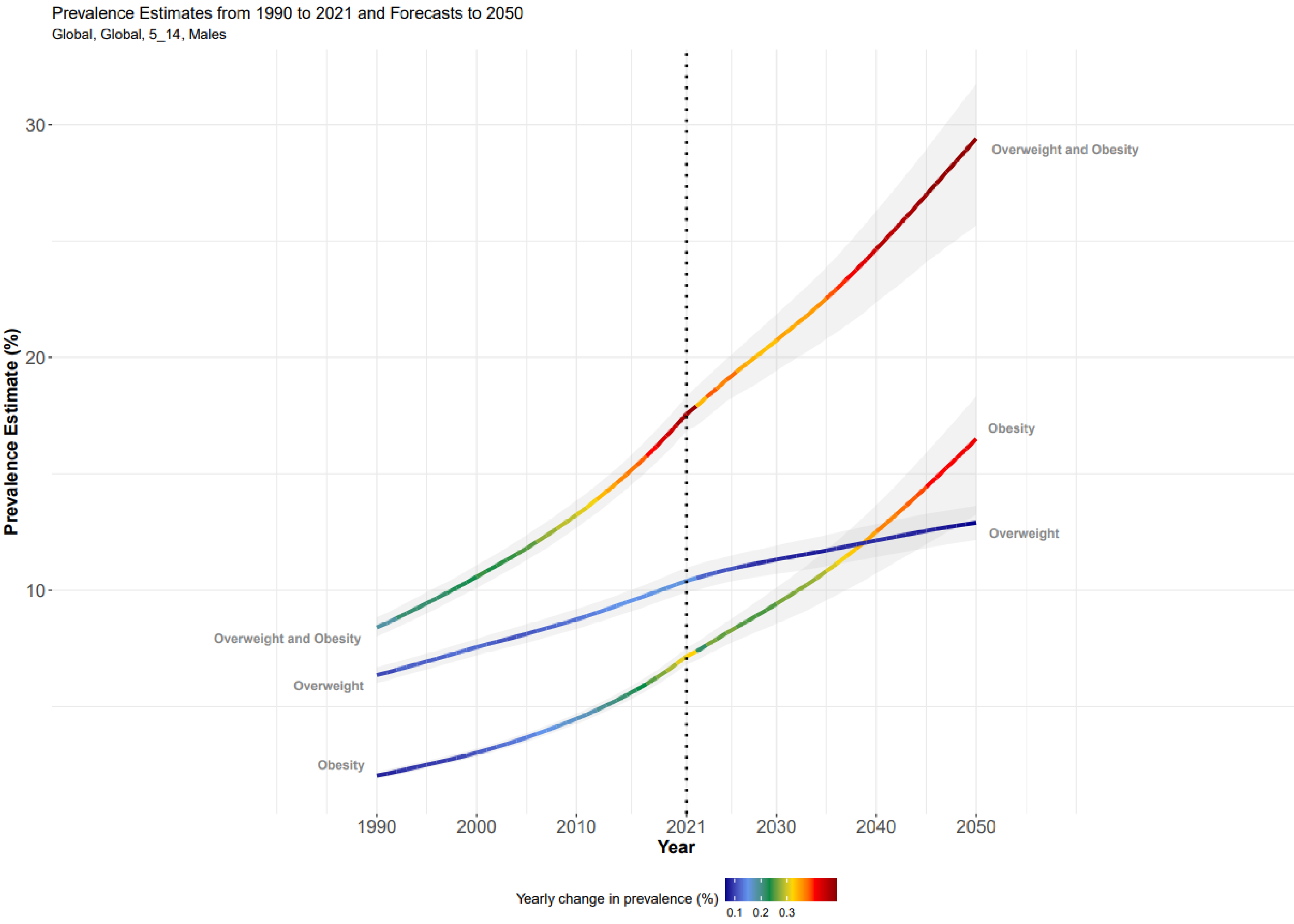

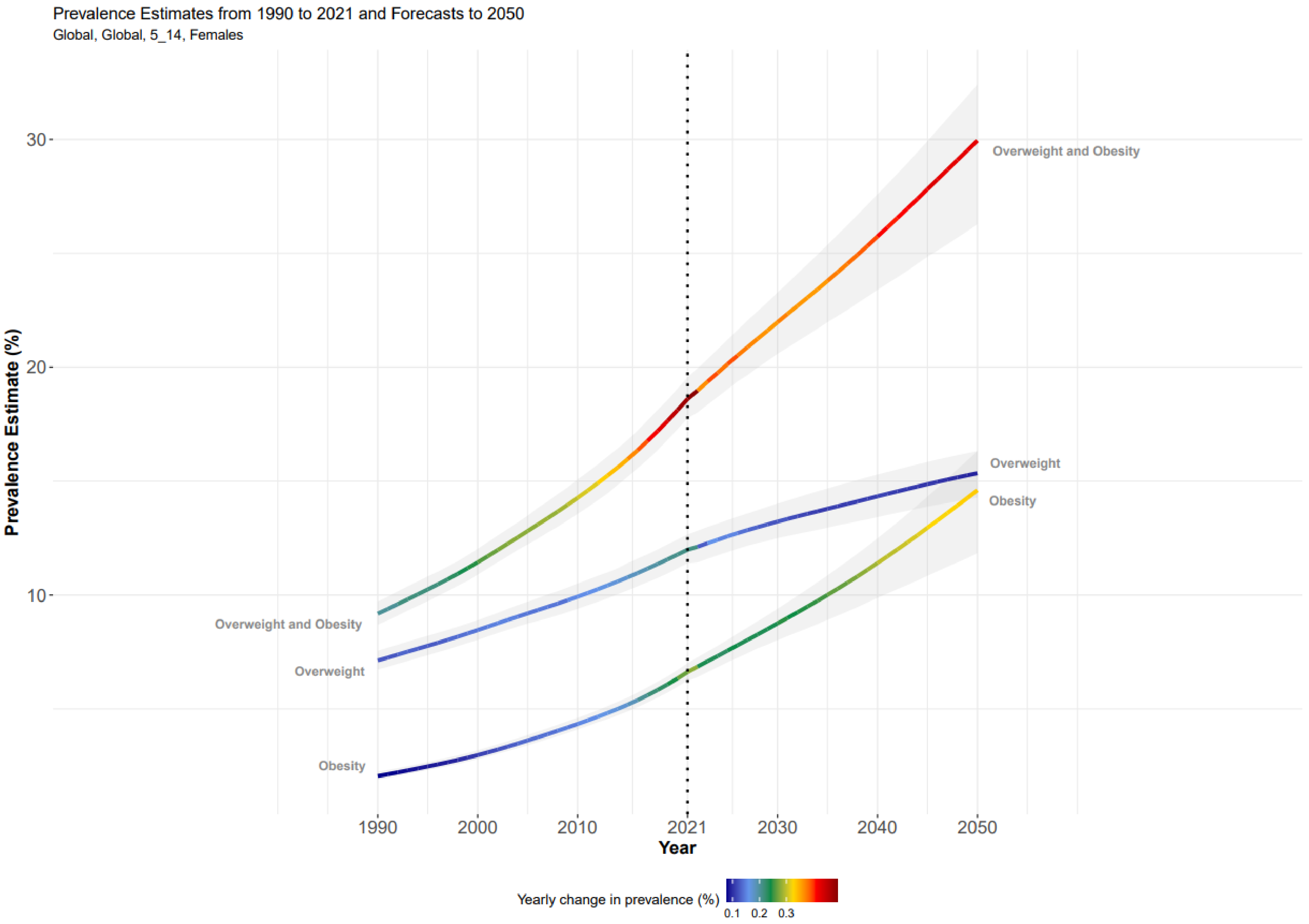

Prevalence Estimates from 1990 to 2021 and Forecasts to 2050
Global, Global, 5_14, Females
Prevalence Estimate (%)
30
20
10
Overweight and Obesity
Overweight
Obesity
Overweight and Obesity
Overweight
Obesity
1990
2000
2010
2021
2030
2040
2050
Year
Yearly change in prevalence (%)
0.1
0.2
0.3

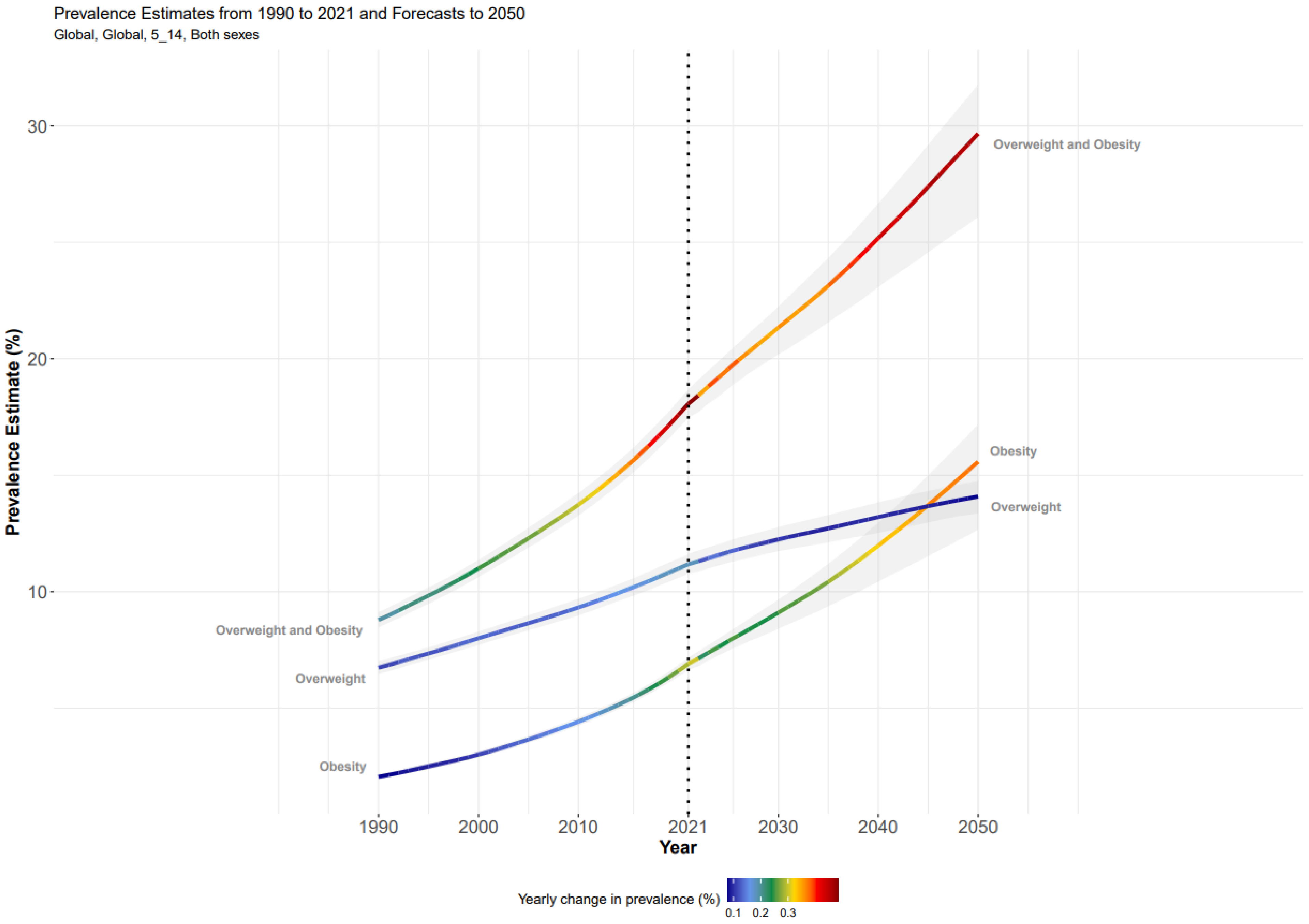

Prevalence Estimates from 1990 to 2021 and Forecasts to 2050
Global, Global, 5_14, Both sexes
Prevalence Estimate (%)
30
20
10
1990
2000
2010
2021
2030
2040
2050
Year
Overweight and Obesity
Obesity
Overweight
Overweight and Obesity
Overweight
Obesity
Yearly change in prevalence (%)
0.1
0.2
0.3

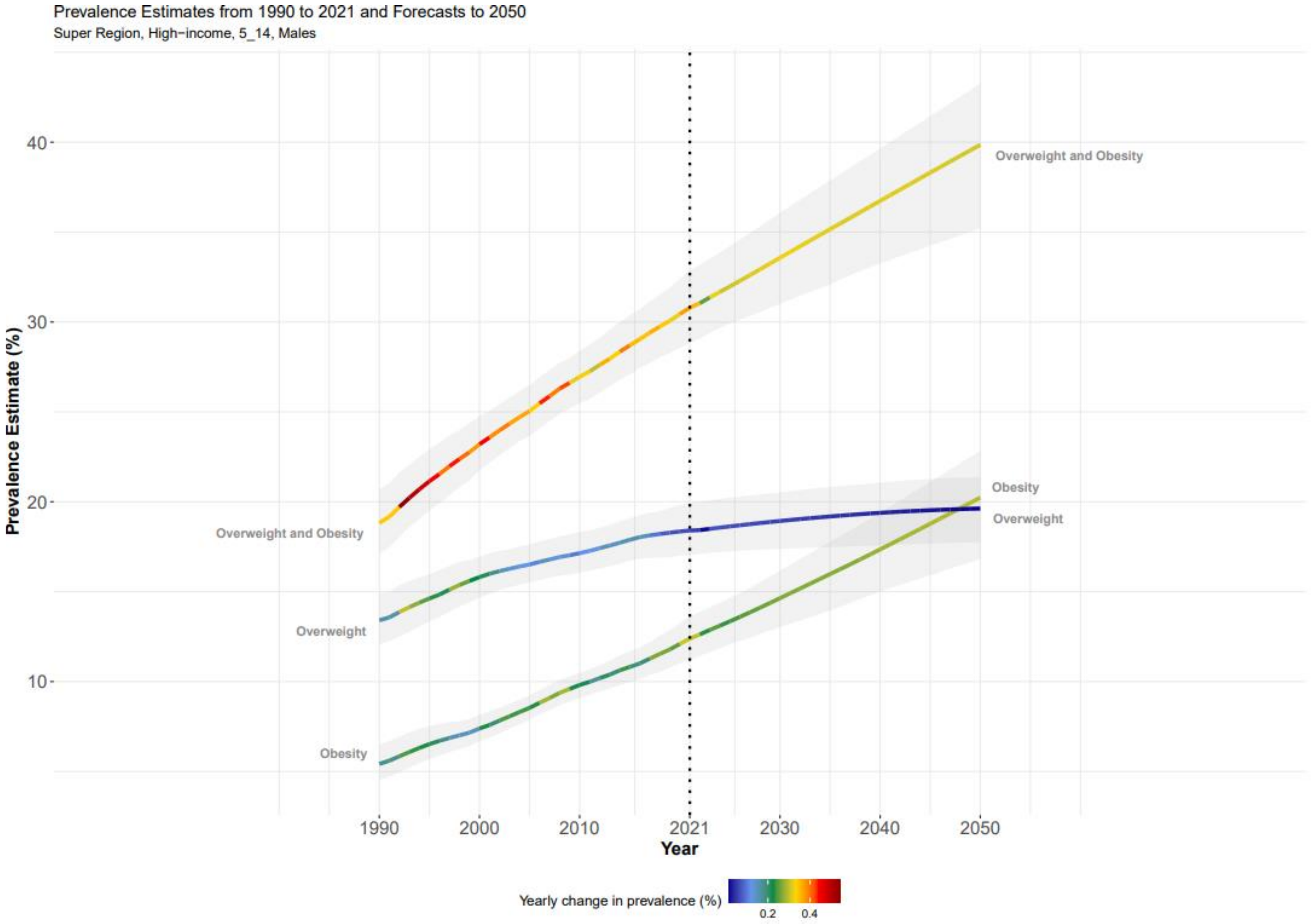

Prevalence Estimates from 1990 to 2021 and Forecasts to 2050
Super Region, High−income, 5_14, Males
Prevalence Estimate (%)
40
30
20
10
Overweight and Obesity
Overweight
Obesity
Overweight and Obesity
Obesity
Overweight
1990
2000
2010
2021
2030
2040
2050
Year
Yearly change in prevalence (%)
0.2
0.4

Prevalence Estimates from 1990 to 2021 and Forecasts to 2050
Super Region, High−income, 5_14, Females

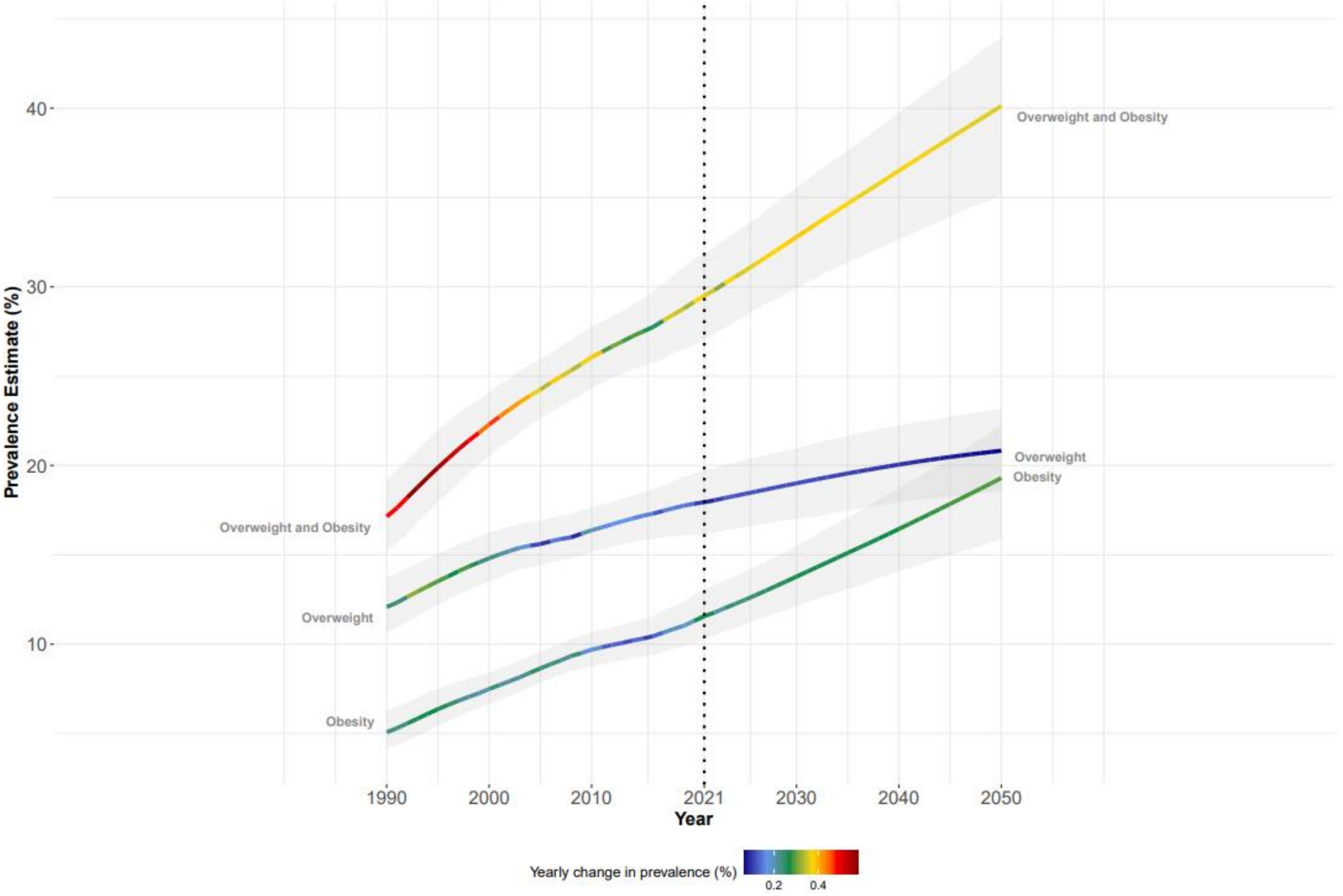

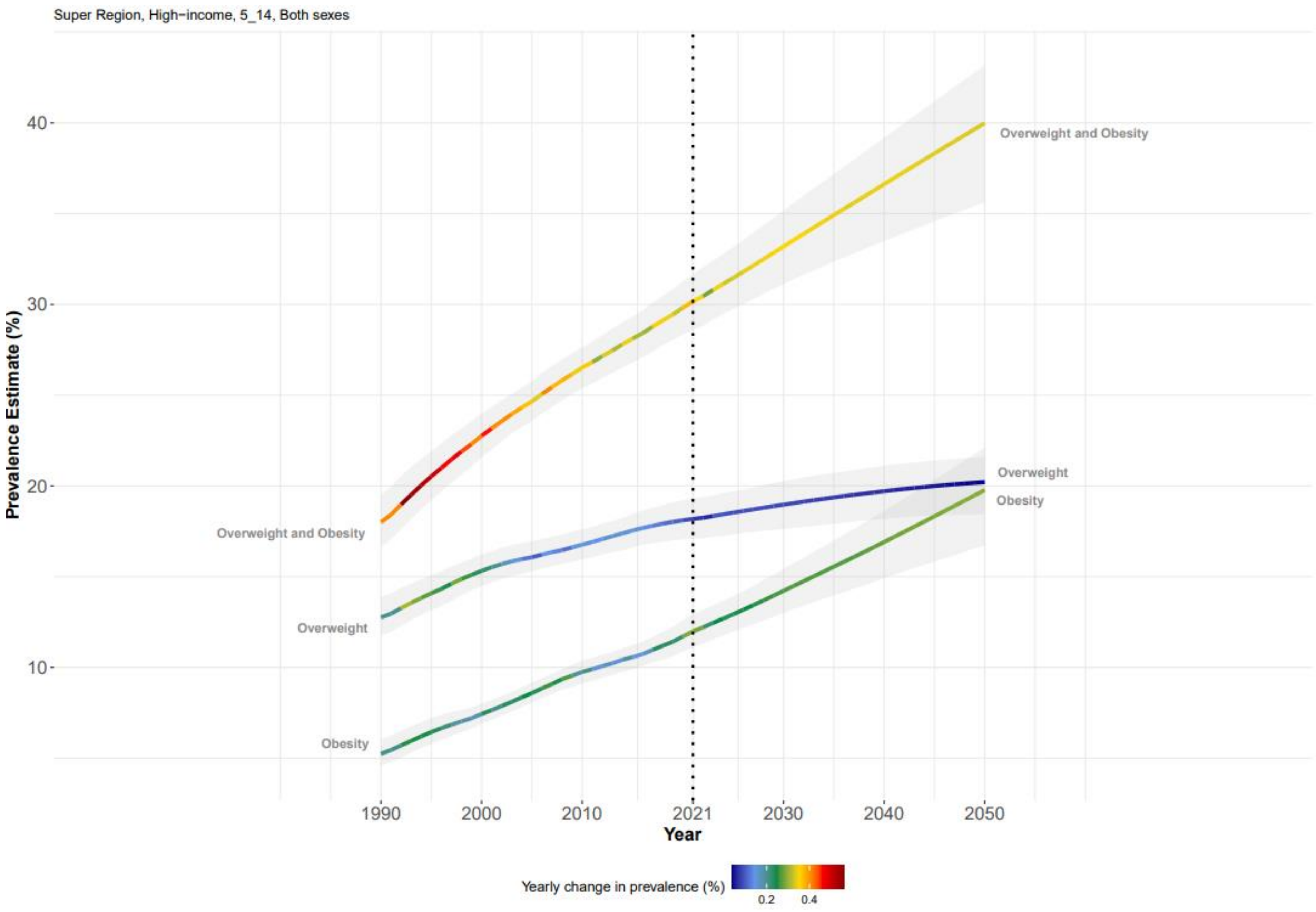
Super Region, High−income, 5_14, Both sexes
Prevalence Estimate (%)
40
30
20
10
1990
2000
2010
2021
2030
2040
2050
Year
Overweight and Obesity
Overweight
Obesity
Overweight and Obesity
Overweight
Obesity
Yearly change in prevalence (%)
0.2
0.4

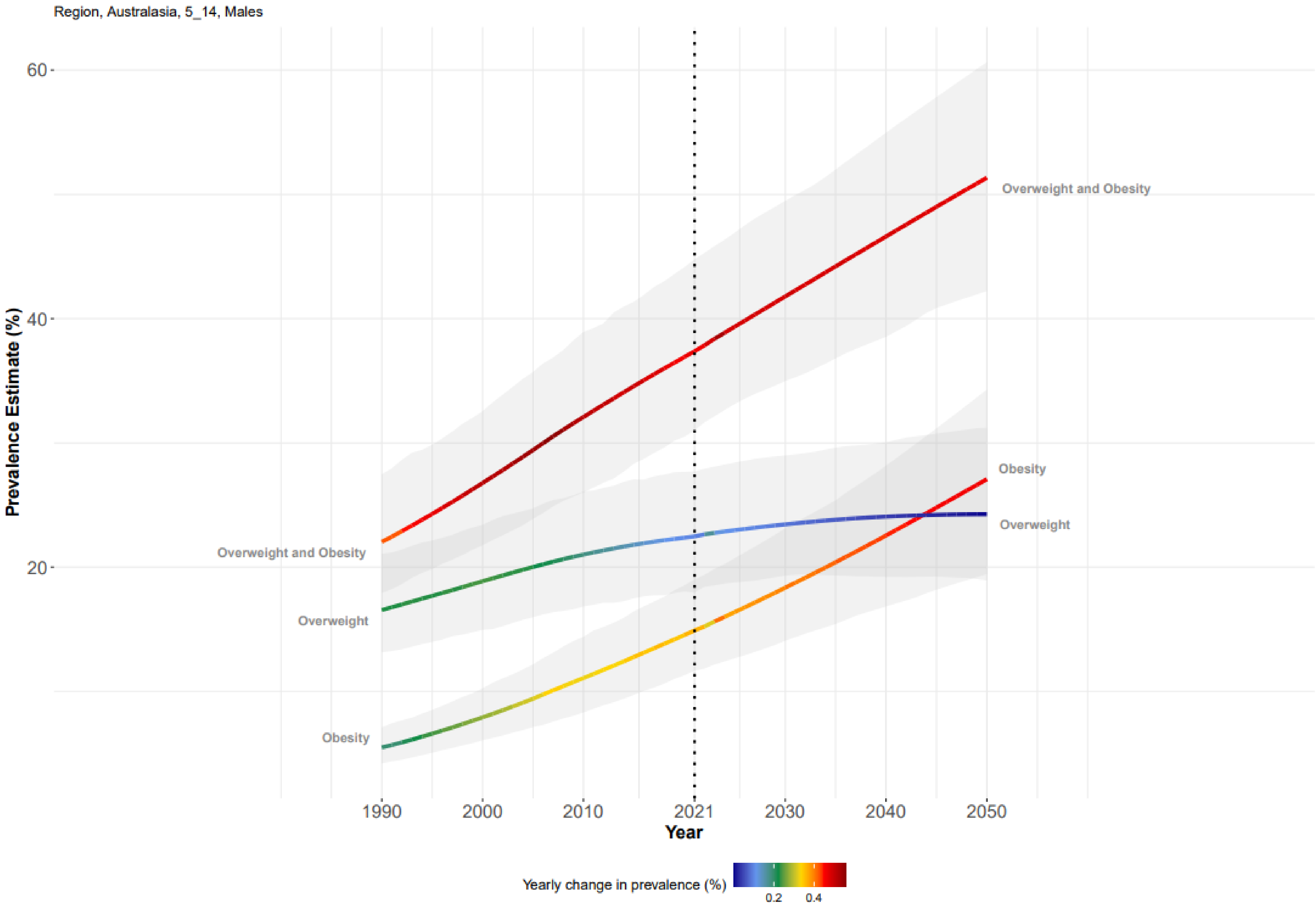

Region, Australasia, 5_14, Males
Prevalence Estimate (%)
60
40
20
Overweight and Obesity
Obesity
Overweight
Overweight and Obesity
Overweight
Obesity
1990
2000
2010
2021
2030
2040
2050
Year
Yearly change in prevalence (%)
0.2
0.4

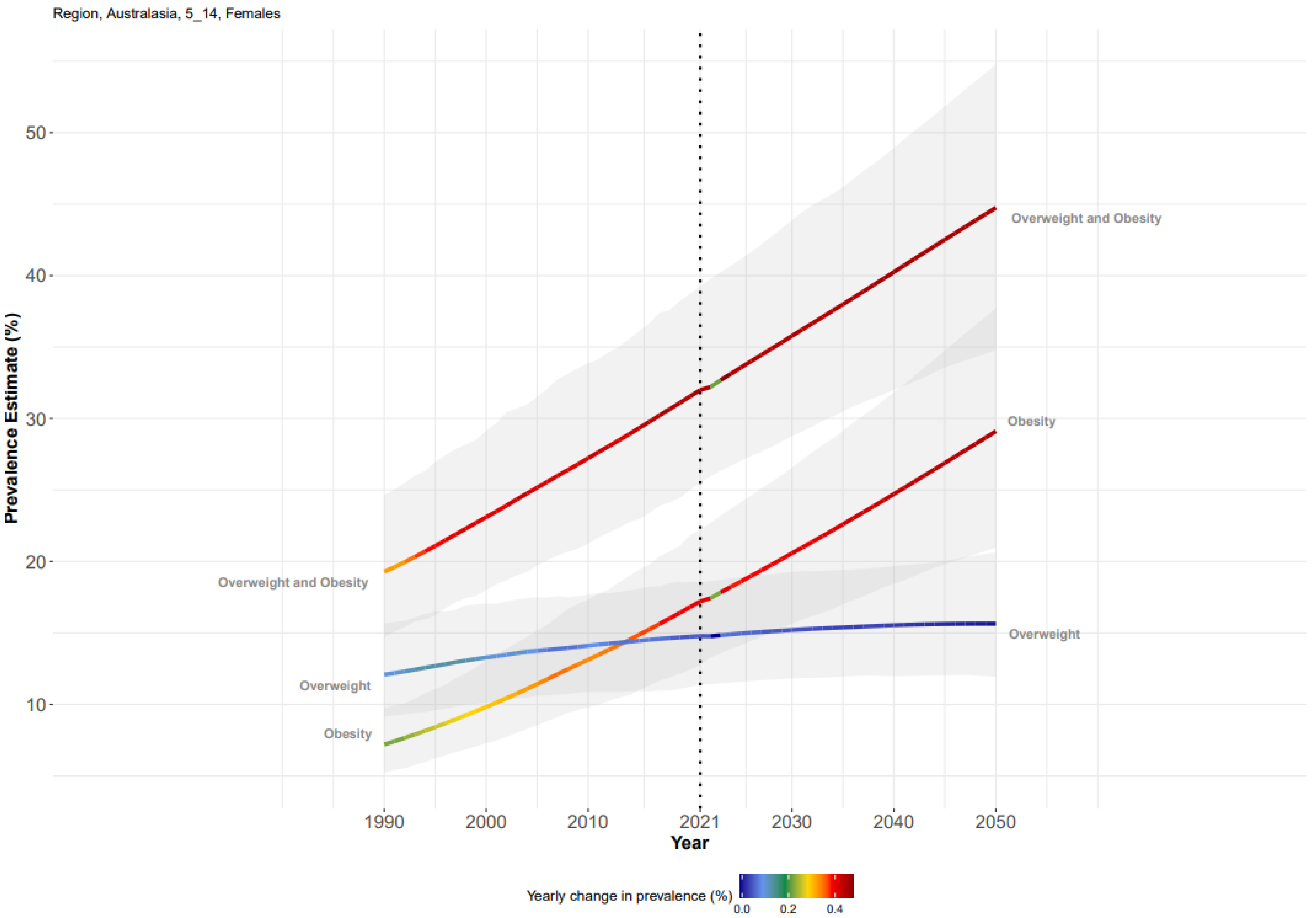

Region, Australasia, 5_14, Females
Prevalence Estimate (%)
50
40
30
20
10
Overweight and Obesity
Overweight
Obesity
Overweight and Obesity
Obesity
Overweight
1990
2000
2010
2021
2030
2040
2050
Year
Yearly change in prevalence (%)
0.0
0.2
0.4

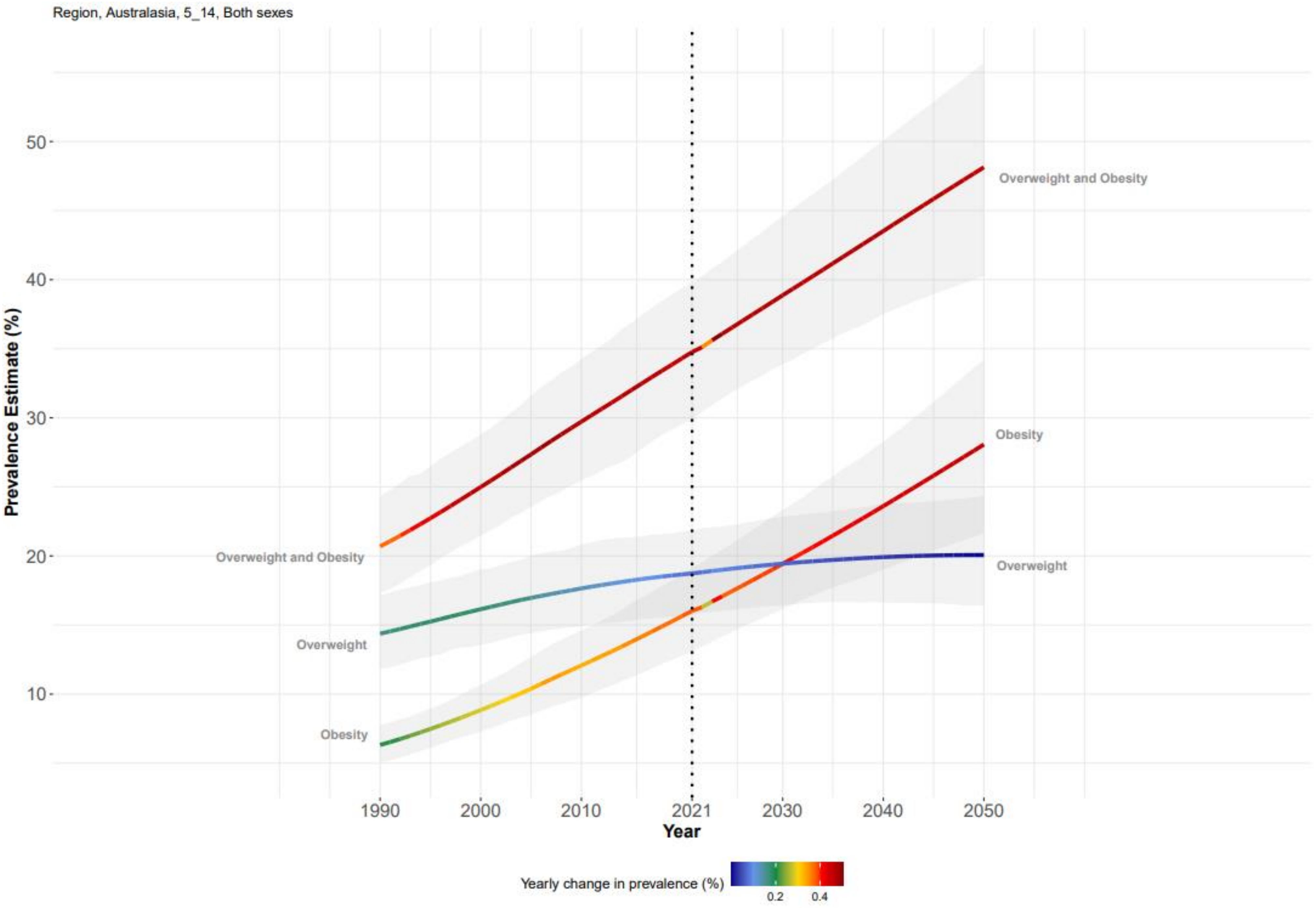

Region, Australasia, 5_14, Both sexes
Prevalence Estimate (%)
50
40
30
20
10
1990
2000
2010
2021
2030
2040
2050
Year
Overweight and Obesity
Overweight
Obesity
Overweight and Obesity
Obesity
Overweight
Yearly change in prevalence (%)
0.2
0.4

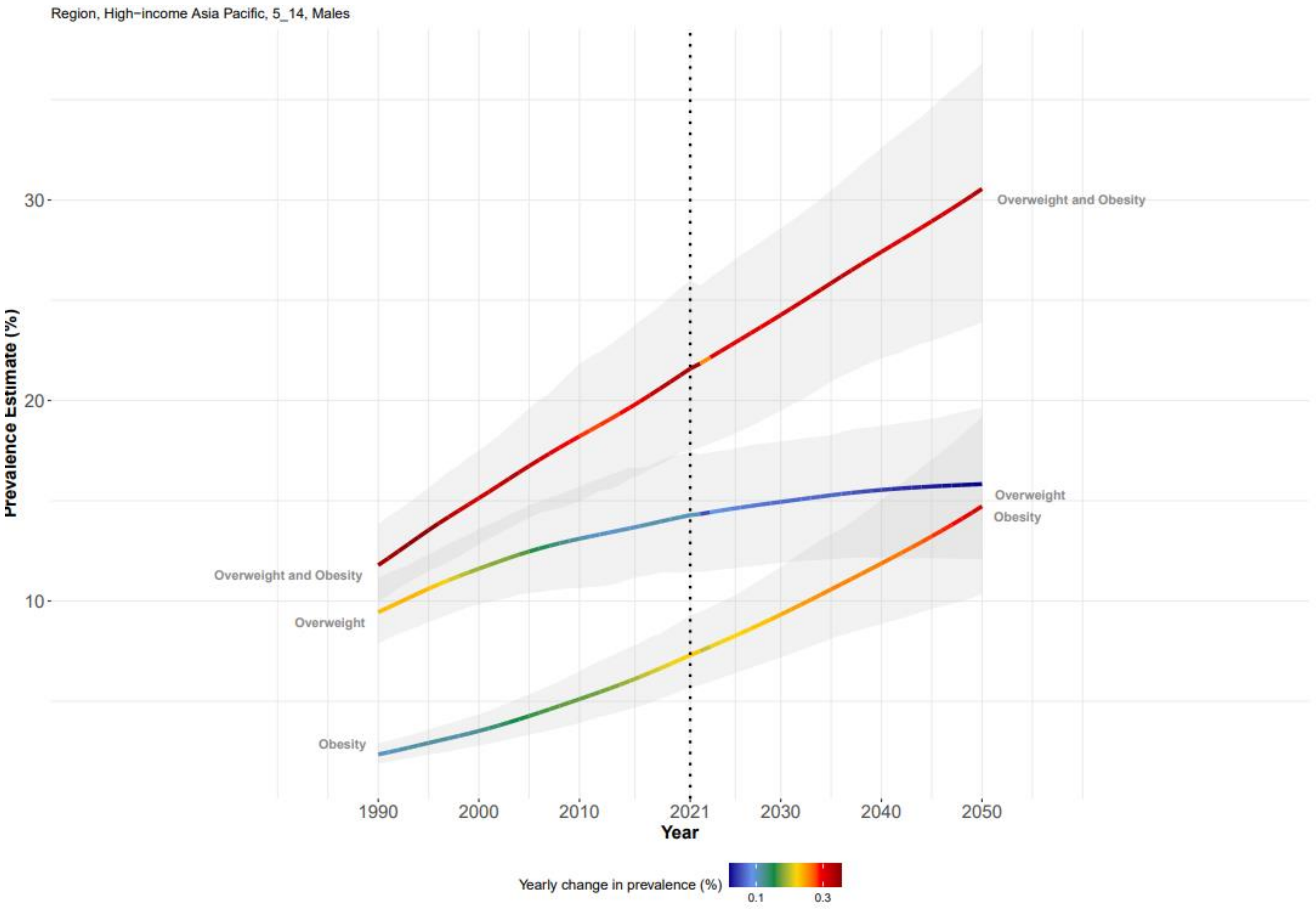
Region, High−income Asia Pacific, 5_14, Males
Prevalence Estimate (%)
30
20
10
Overweight and Obesity
Overweight
Obesity
Overweight and Obesity
Overweight
Obesity
1990
2000
2010
2021
2030
2040
2050
Year
Yearly change in prevalence (%)
0.1
0.3

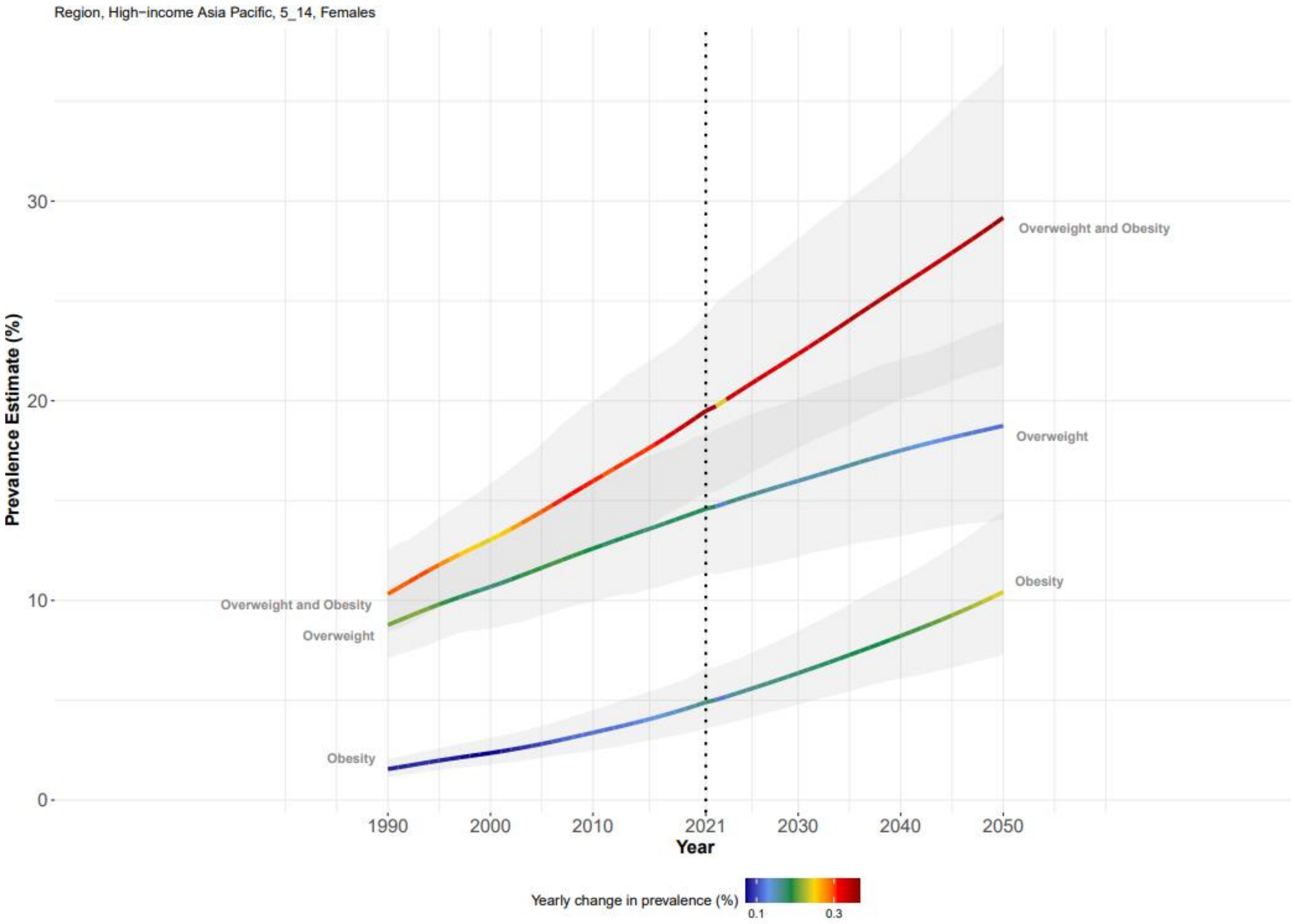

Region, High−income Asia Pacific, 5_14, Females
Prevalence Estimate (%)
30
20
10
0
1990
2000
2010
2021
2030
2040
2050
Year
Overweight and Obesity
Overweight
Obesity
Overweight and Obesity
Overweight
Obesity
Yearly change in prevalence (%)
0.1
0.3

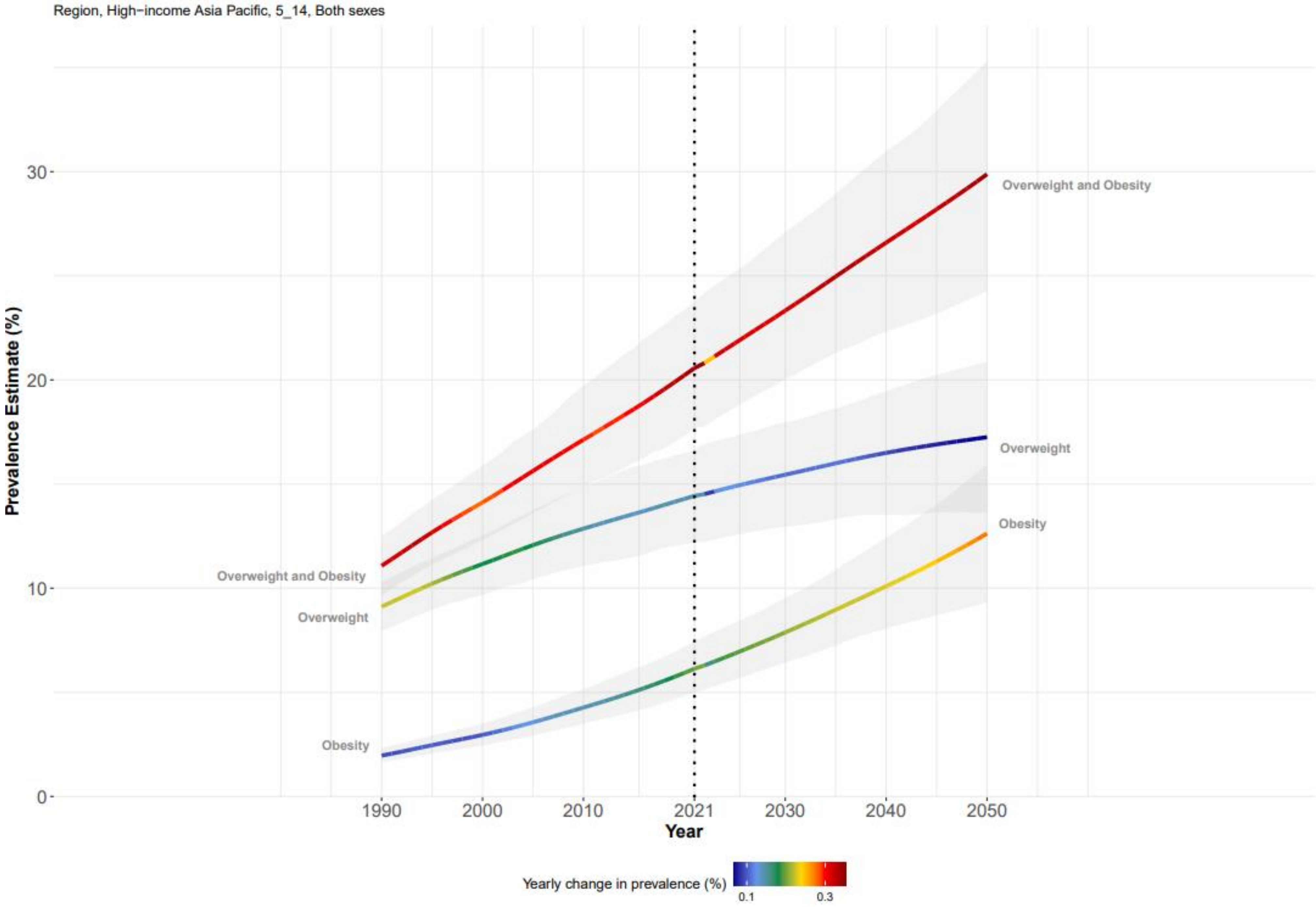

Region, High−income Asia Pacific, 5_14, Both sexes
Prevalence Estimate (%)
30
20
10
0
1990
2000
2010
2021
2030
2040
2050
Year
Overweight and Obesity
Overweight
Obesity
Overweight and Obesity
Overweight
Obesity
Yearly change in prevalence (%)
0.1
0.3

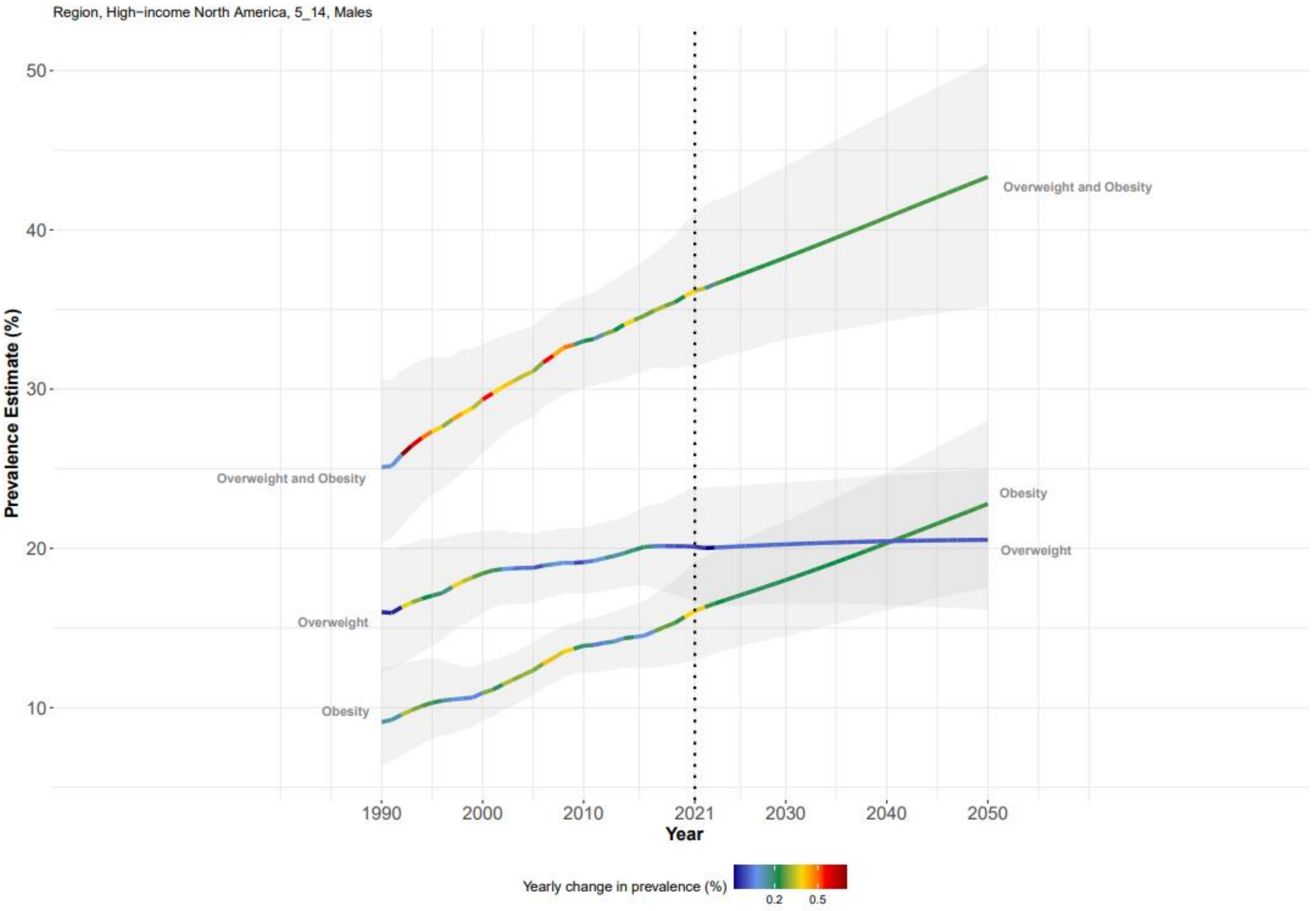

Region, High−income North America, 5_14, Males
Prevalence Estimate (%)
50
40
30
20
10
1990
2000
2010
2021
2030
2040
2050
Year
Overweight and Obesity
Overweight
Obesity
Overweight and Obesity
Obesity
Overweight
Yearly change in prevalence (%)
0.2
0.5

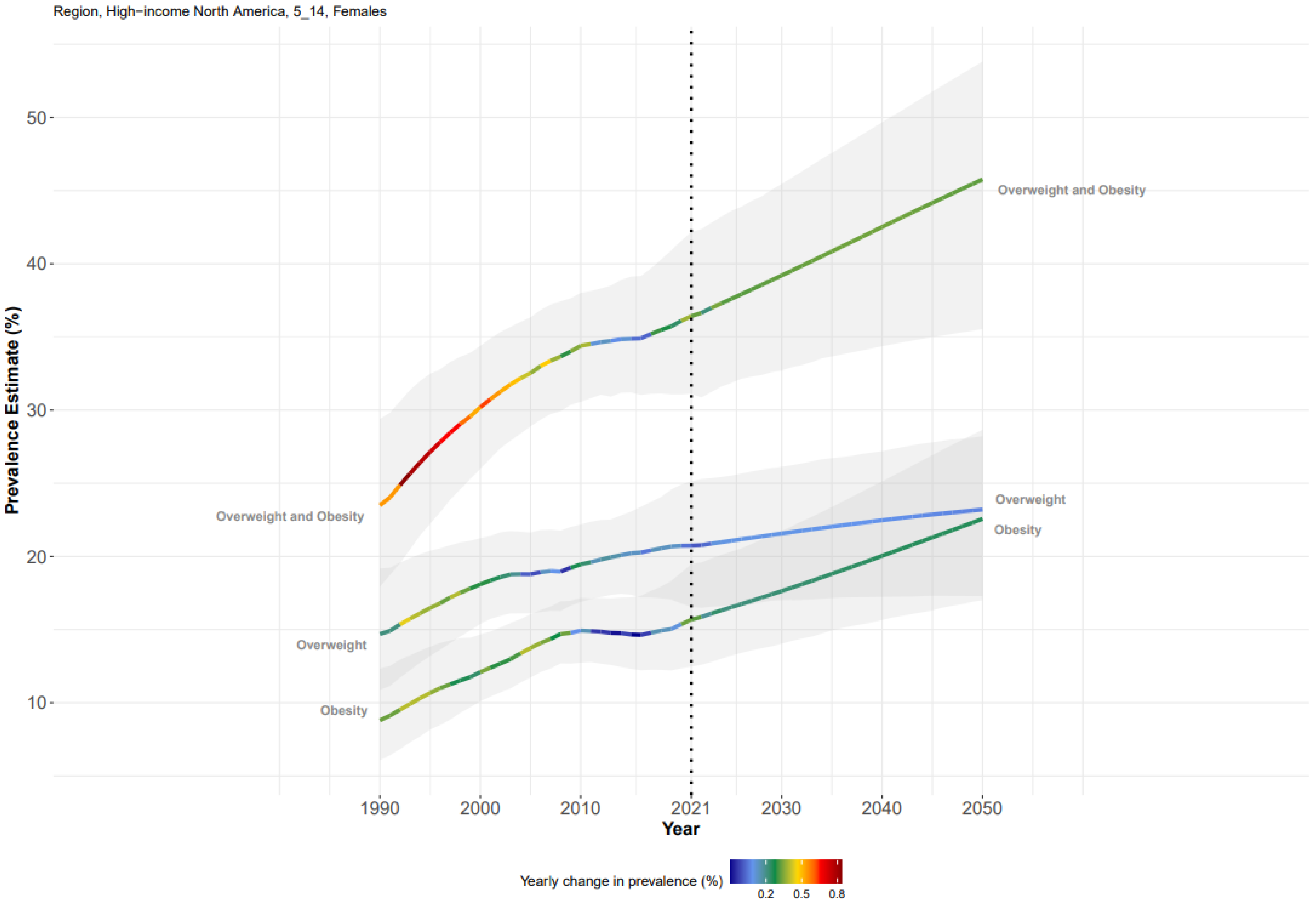
Region, High-income North America, 5_14, Females
Prevalence Estimate (%)
50
40
30
20
10
1990
2000
2010
2021
2030
2040
2050
Year
Overweight and Obesity
Overweight
Obesity
Overweight and Obesity
Overweight
Obesity
Yearly change in prevalence (%)
0.2
0.5
0.8

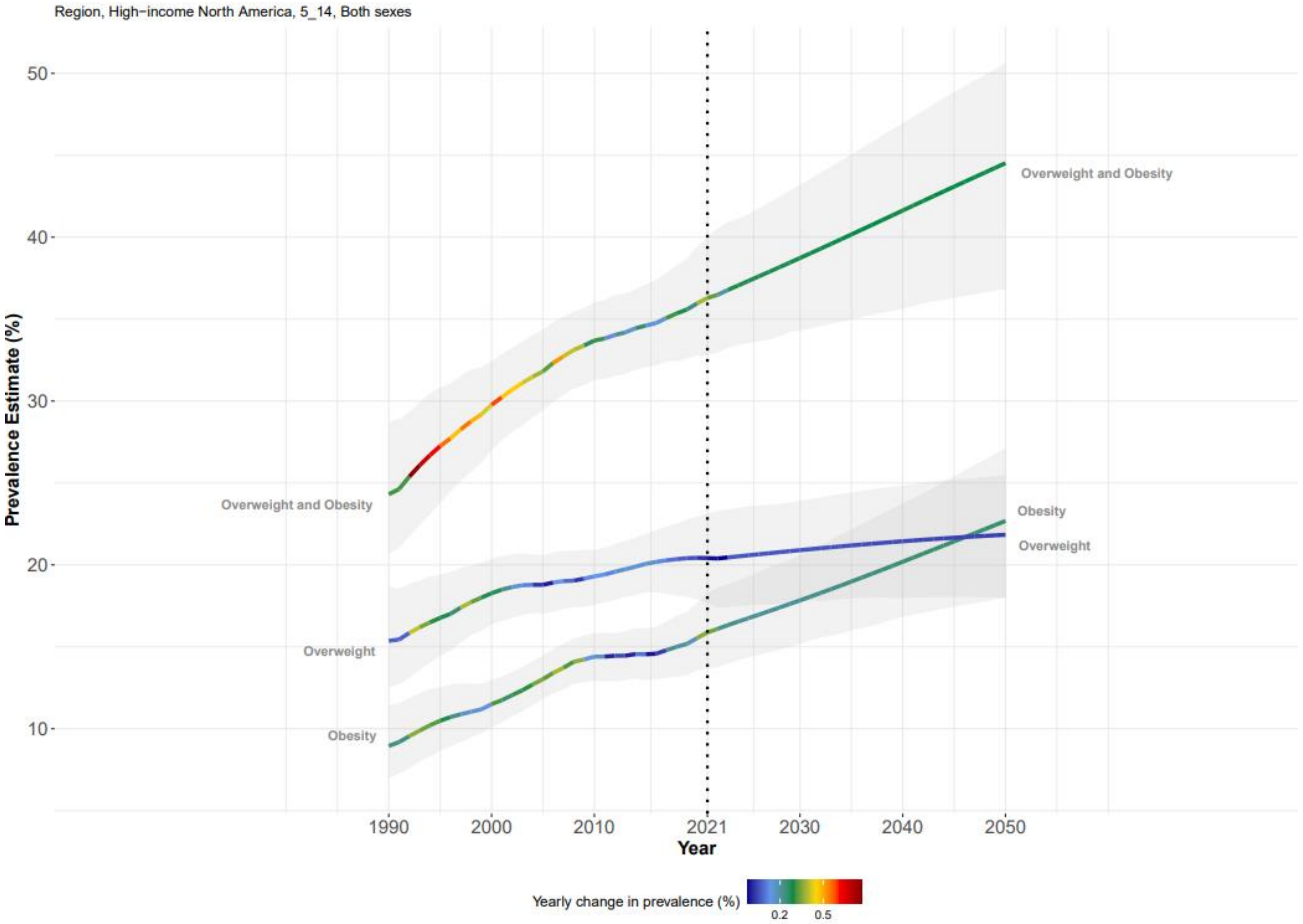

Region, High−income North America, 5_14, Both sexes
Prevalence Estimate (%)
50
40
30
20
10
1990
2000
2010
2021
2030
2040
2050
Year
Overweight and Obesity
Overweight
Obesity
Overweight and Obesity
Obesity
Overweight
Yearly change in prevalence (%)
0.2
0.5

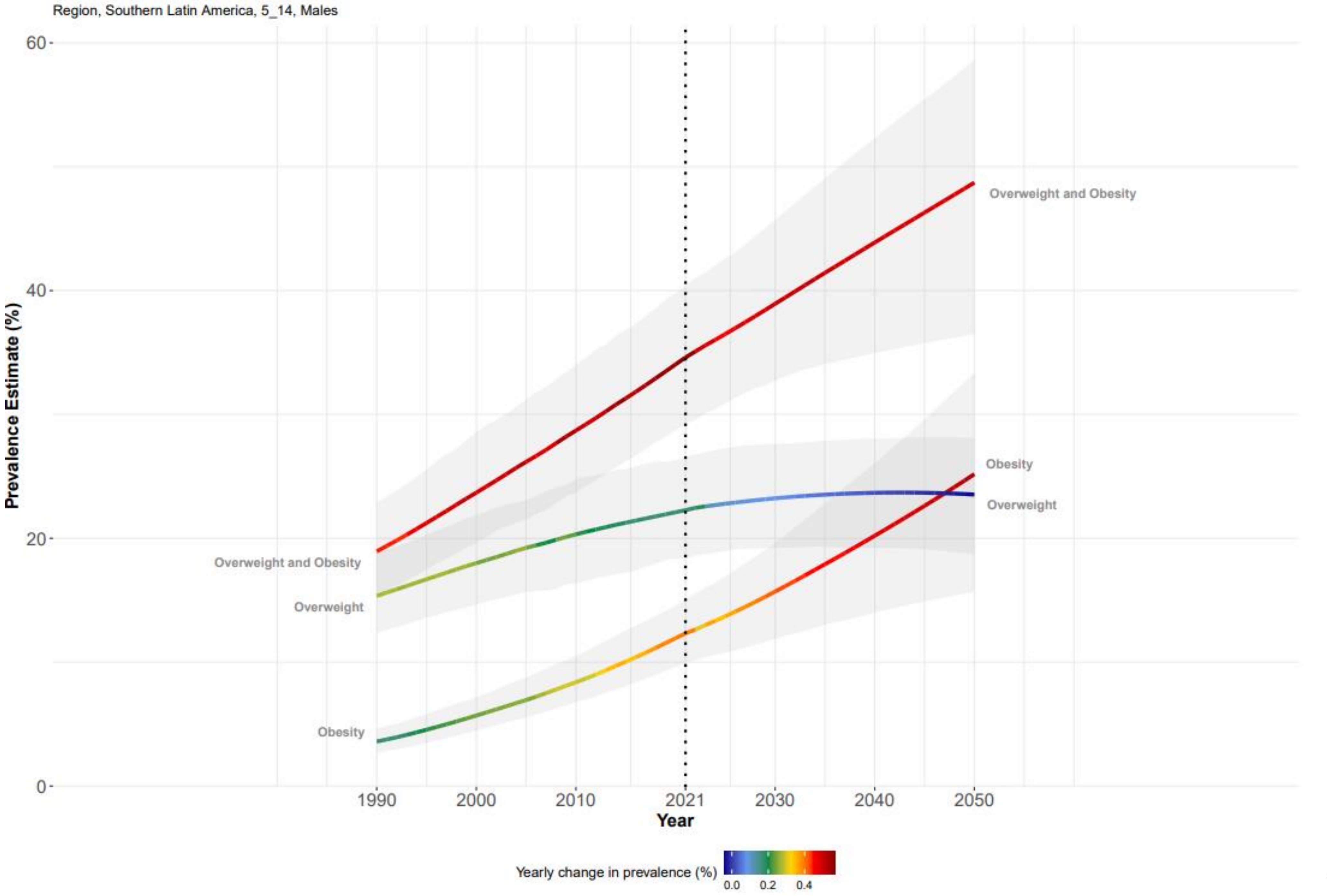

Region, Southern Latin America, 5_14, Males
Prevalence Estimate (%)
60
40
20
0
1990
2000
2010
2021
2030
2040
2050
Year
Overweight and Obesity
Obesity
Overweight
Overweight and Obesity
Overweight
Obesity
Yearly change in prevalence (%)
0.0
0.2
0.4

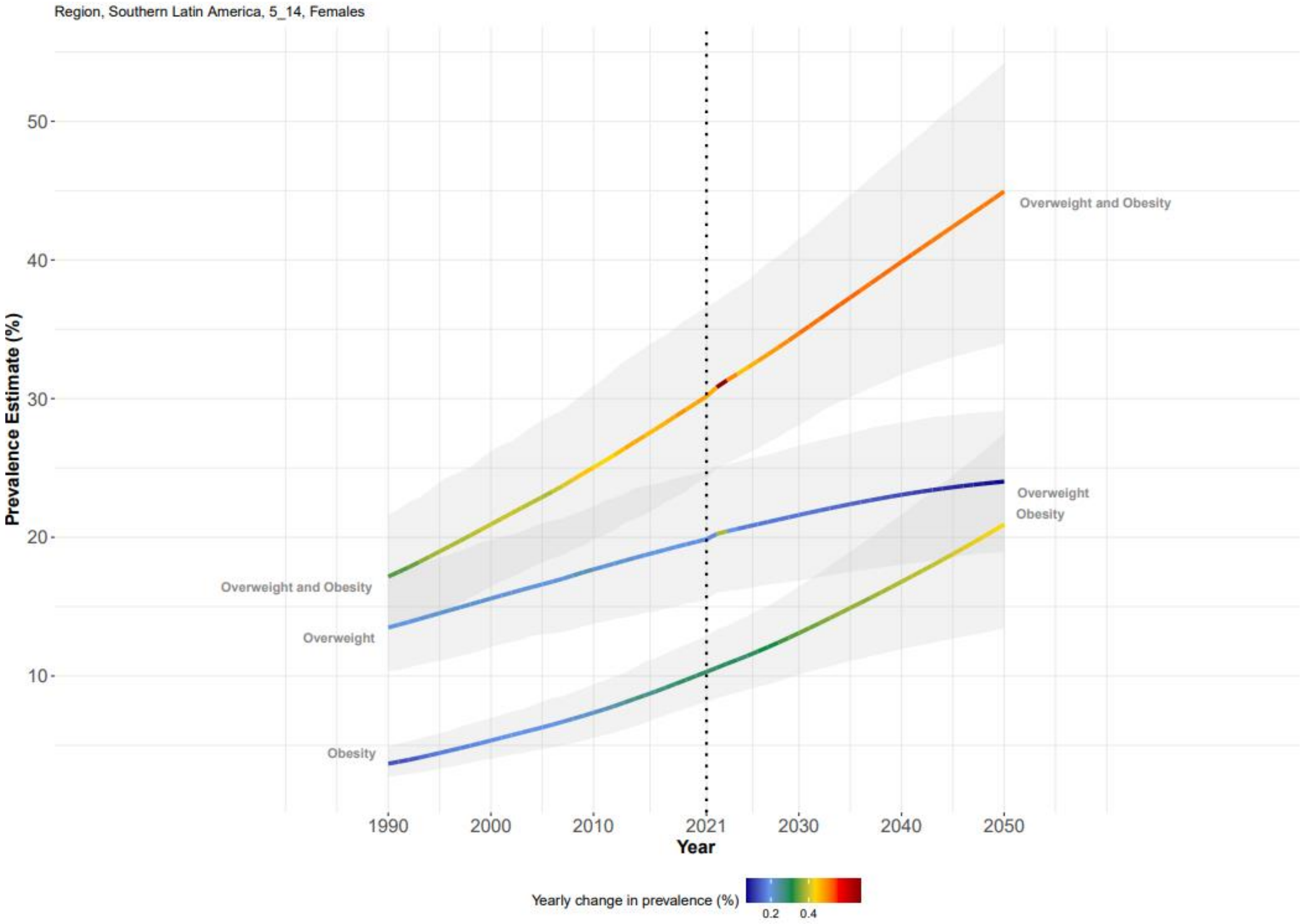
Region, Southern Latin America, 5_14, Females
Prevalence Estimate (%)
50
40
30
20
10
Overweight and Obesity
Overweight
Obesity
Overweight and Obesity
Overweight
Obesity
1990
2000
2010
2021
2030
2040
2050
Year
Yearly change in prevalence (%)
0.2
0.4

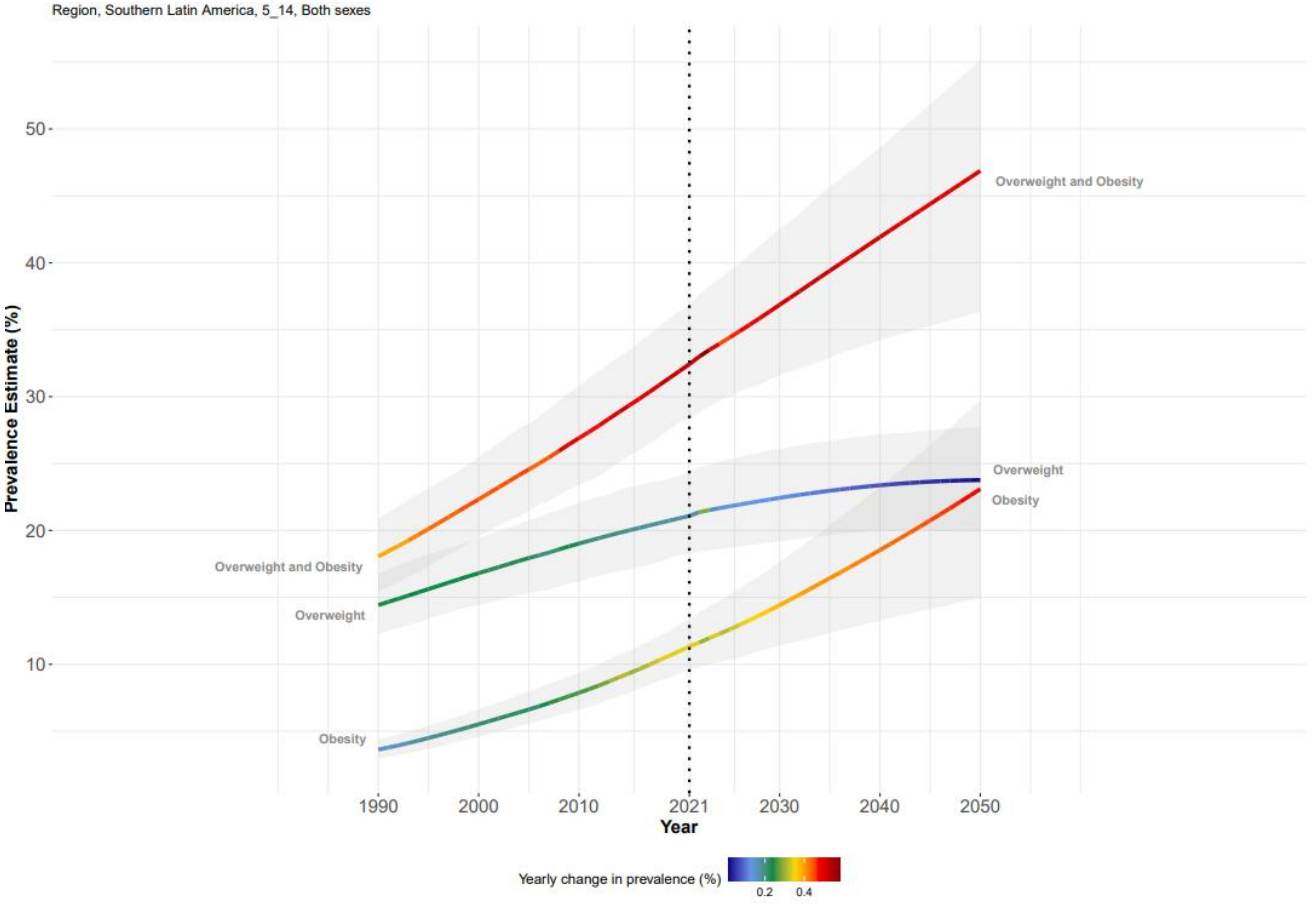

Region, Southern Latin America, 5_14, Both sexes
Prevalence Estimate (%)
50
40
30
20
10
Overweight and Obesity
Overweight
Obesity
Overweight and Obesity
Overweight
Obesity
1990
2000
2010
2021
2030
2040
2050
Year
Yearly change in prevalence (%)
0.2
0.4

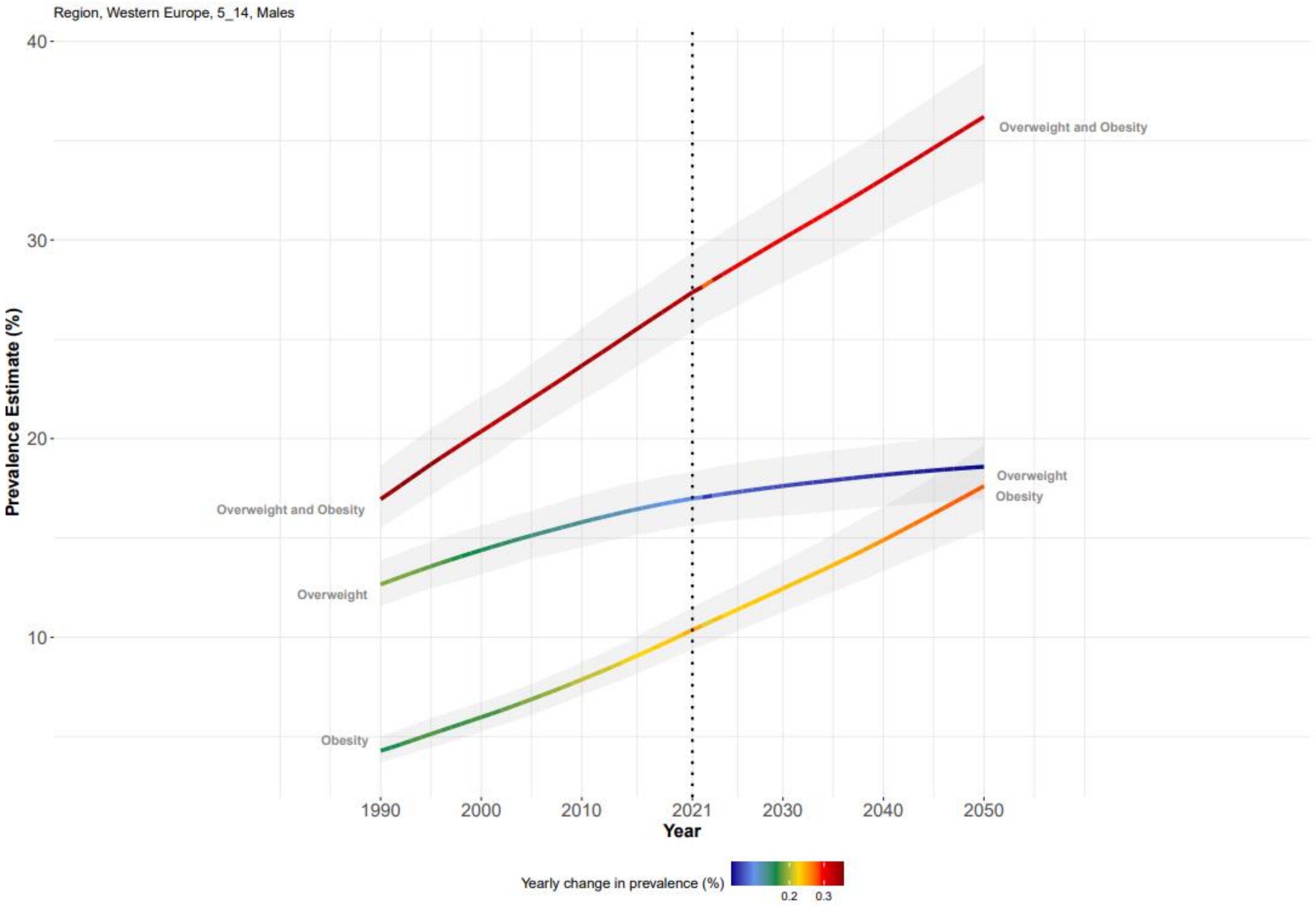

Region, Western Europe, 5_14, Males
Prevalence Estimate (%)
40
30
20
10
Overweight and Obesity
Overweight
Obesity
Overweight and Obesity
Overweight
Obesity
1990
2000
2010
2021
2030
2040
2050
Year
Yearly change in prevalence (%)
0.2
0.3

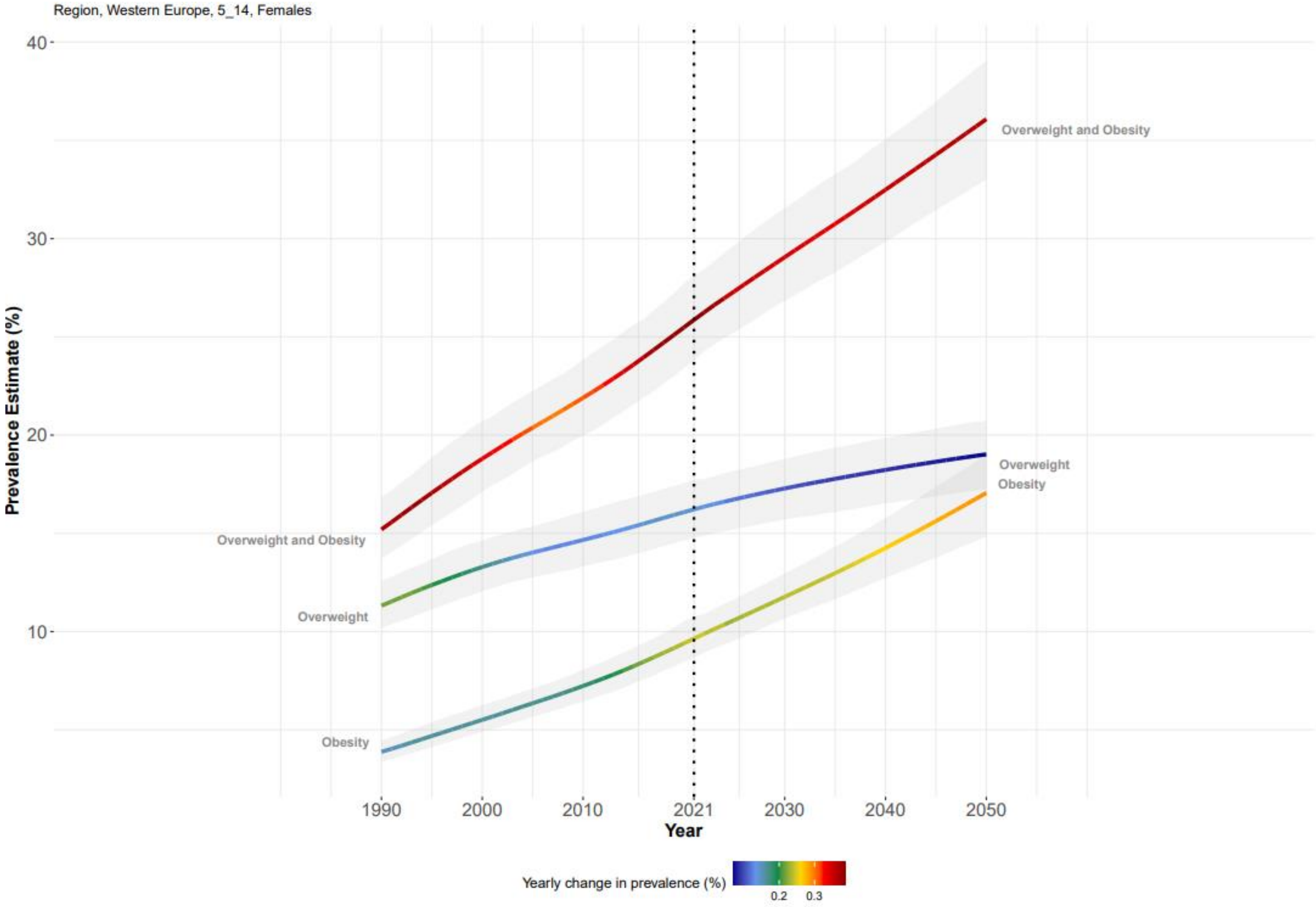
Region, Western Europe, 5_14, Females
Prevalence Estimate (%)
40
30
20
10
Overweight and Obesity
Overweight
Obesity
Overweight and Obesity
Overweight
Obesity
1990
2000
2010
2021
2030
2040
2050
Year
Yearly change in prevalence (%)
0.2
0.3

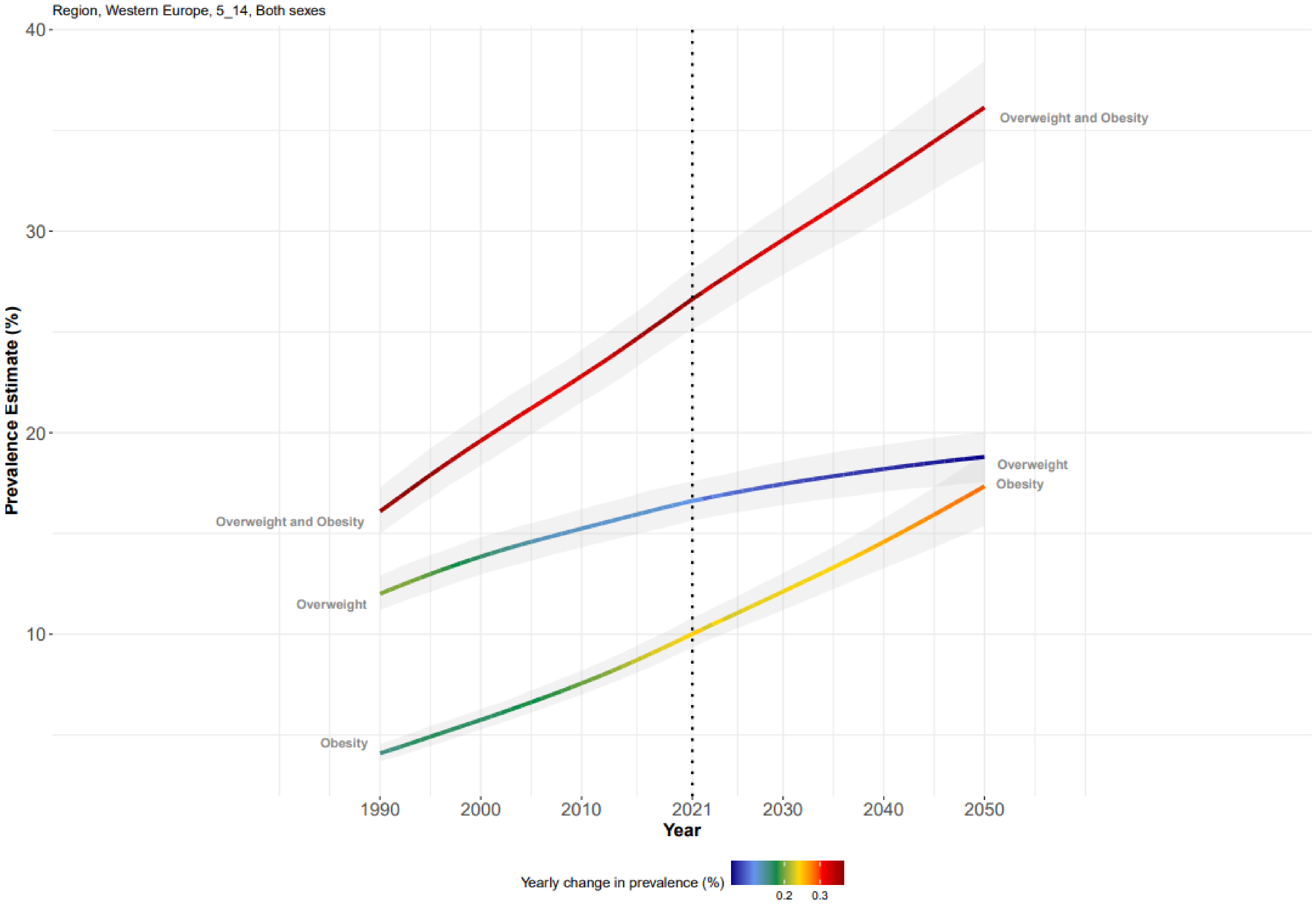
Region, Western Europe, 5_14, Both sexes
Prevalence Estimate (%)
40
30
20
10
1990
2000
2010
2021
2030
2040
2050
Year
Overweight and Obesity
Overweight
Obesity
Overweight and Obesity
Overweight
Obesity
Yearly change in prevalence (%)
0.2
0.3

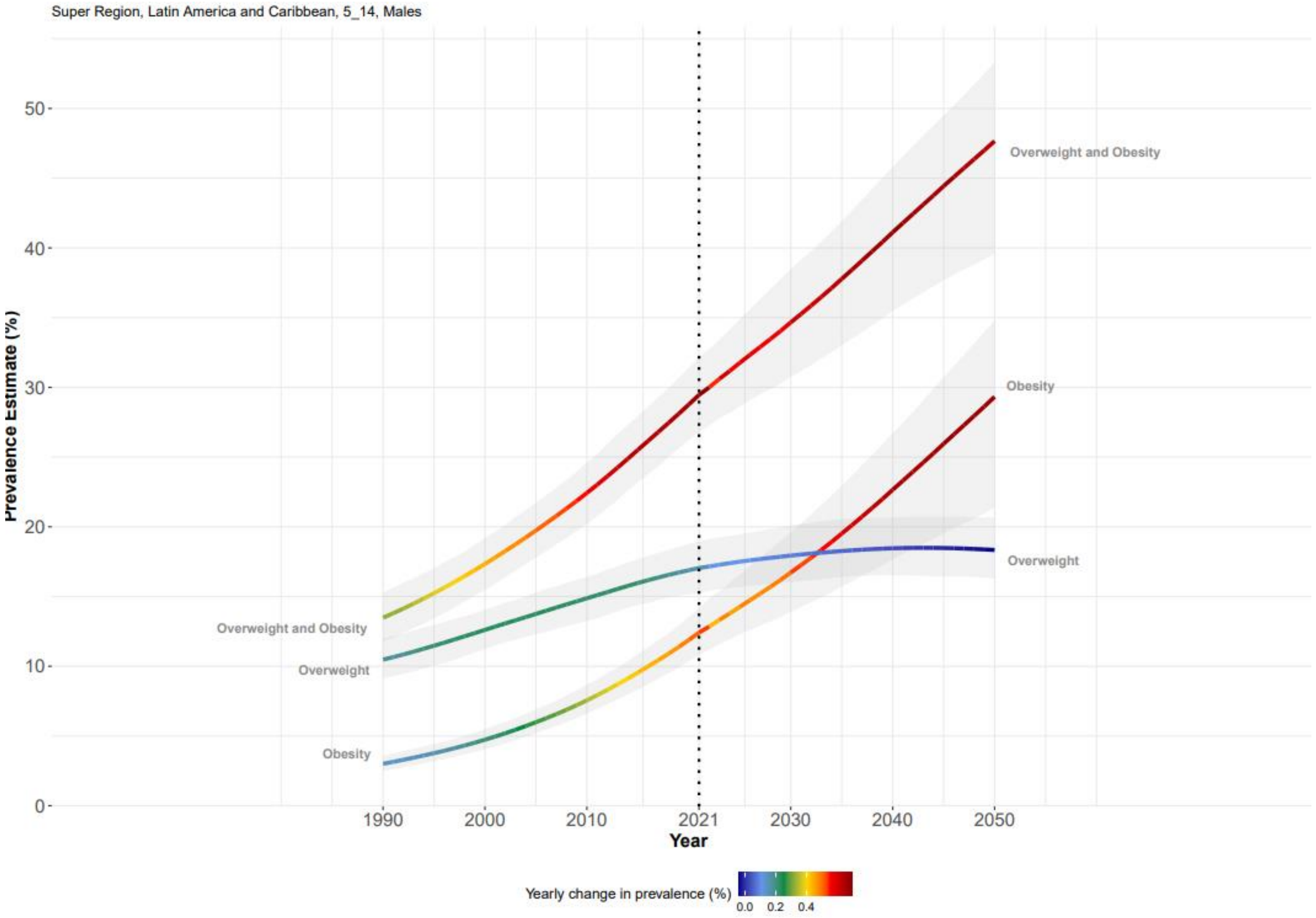
Super Region, Latin America and Caribbean, 5_14, Males
50
40
30
20
10
0
Prevalence Estimate (%)
1990
2000
2010
2021
2030
2040
2050
Year
Overweight and Obesity
Obesity
Overweight
Overweight and Obesity
Overweight
Obesity
Yearly change in prevalence (%)
0.0
0.2
0.4

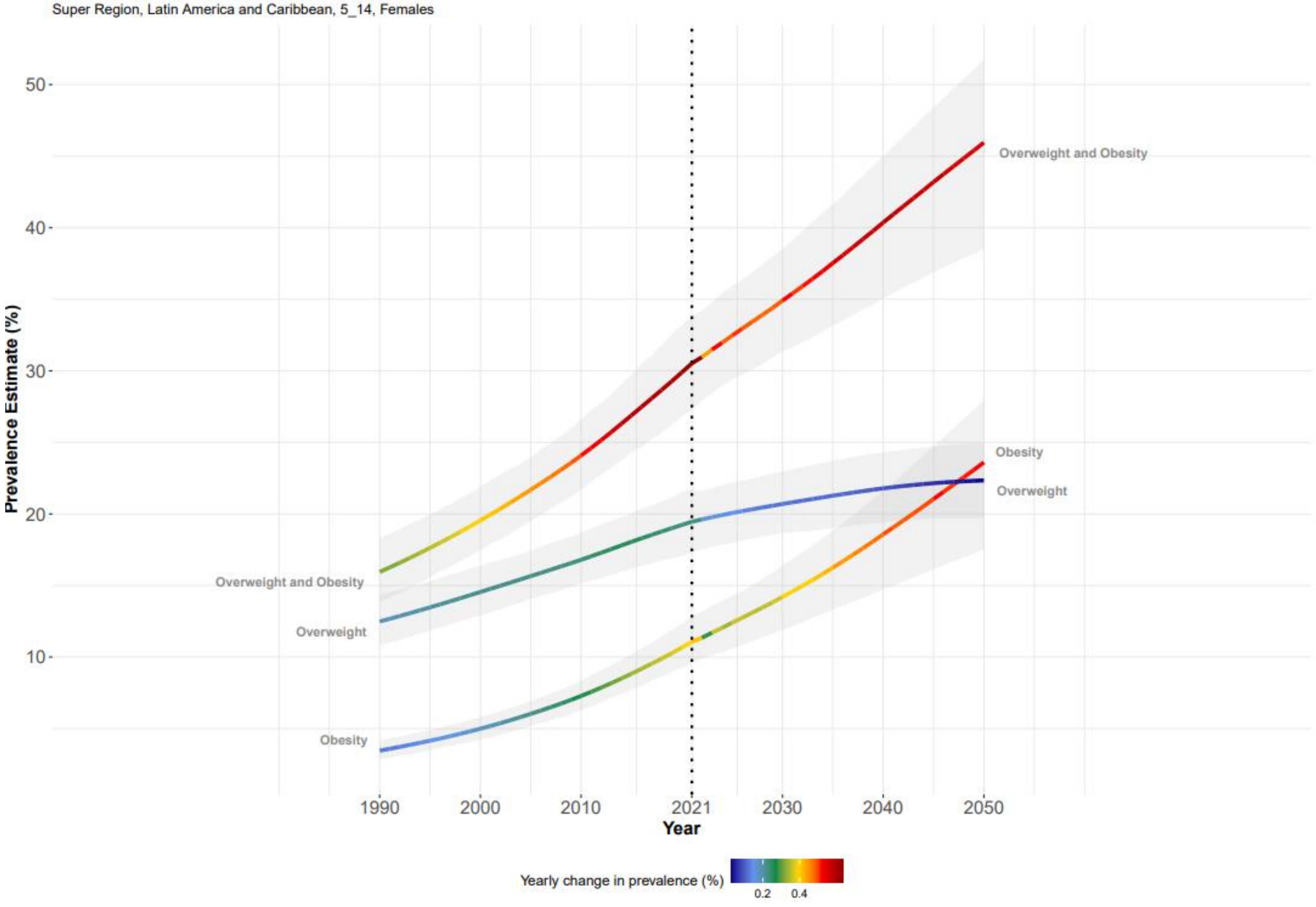

Super Region, Latin America and Caribbean, 5_14, Females
Prevalence Estimate (%)
50
40
30
20
10
1990
2000
2010
2021
2030
2040
2050
Year
Overweight and Obesity
Obesity
Overweight
Overweight and Obesity
Overweight
Obesity
Yearly change in prevalence (%)
0.2
0.4

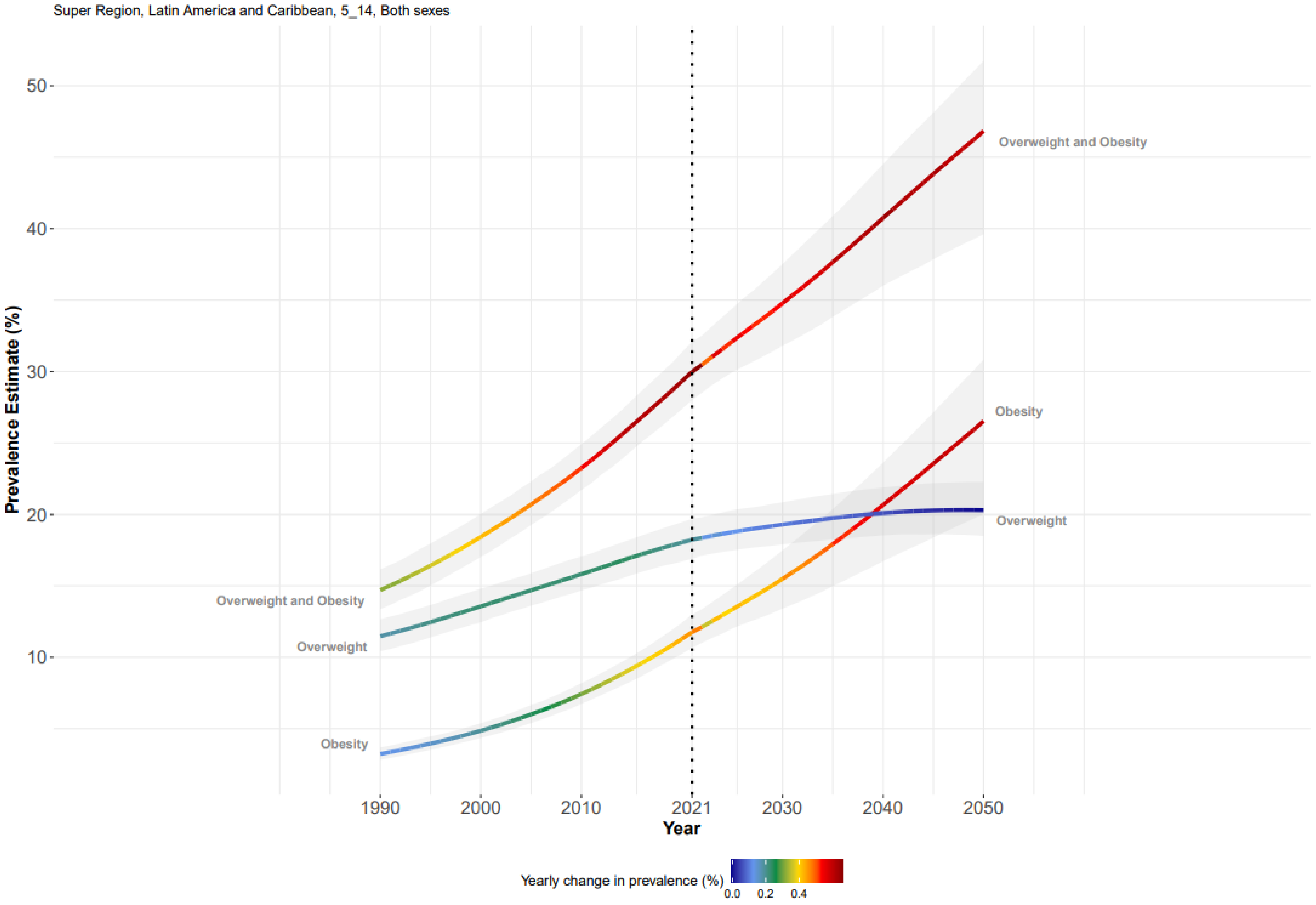

Super Region, Latin America and Caribbean, 5_14, Both sexes
Prevalence Estimate (%)
50
40
30
20
10
Overweight and Obesity
Obesity
Overweight
Overweight and Obesity
Overweight
Obesity
1990
2000
2010
2021
2030
2040
2050
Year
Yearly change in prevalence (%)
0.0
0.2
0.4

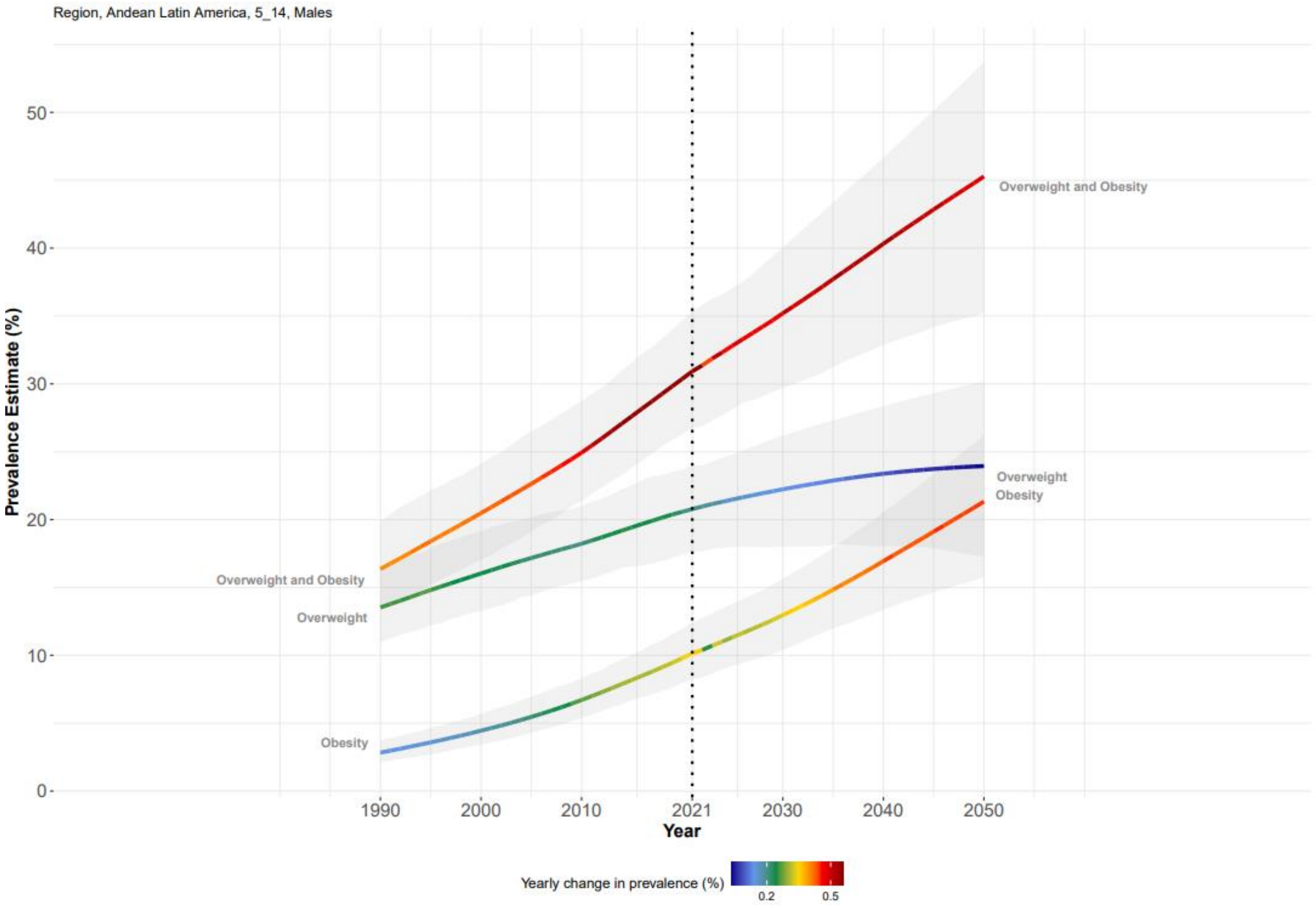
Region, Andean Latin America, 5_14, Males
Prevalence Estimate (%)
50
40
30
20
10
0
1990
2000
2010
2021
2030
2040
2050
Year
Overweight and Obesity
Overweight
Obesity
Overweight and Obesity
Overweight
Obesity
Yearly change in prevalence (%)
0.2
0.5

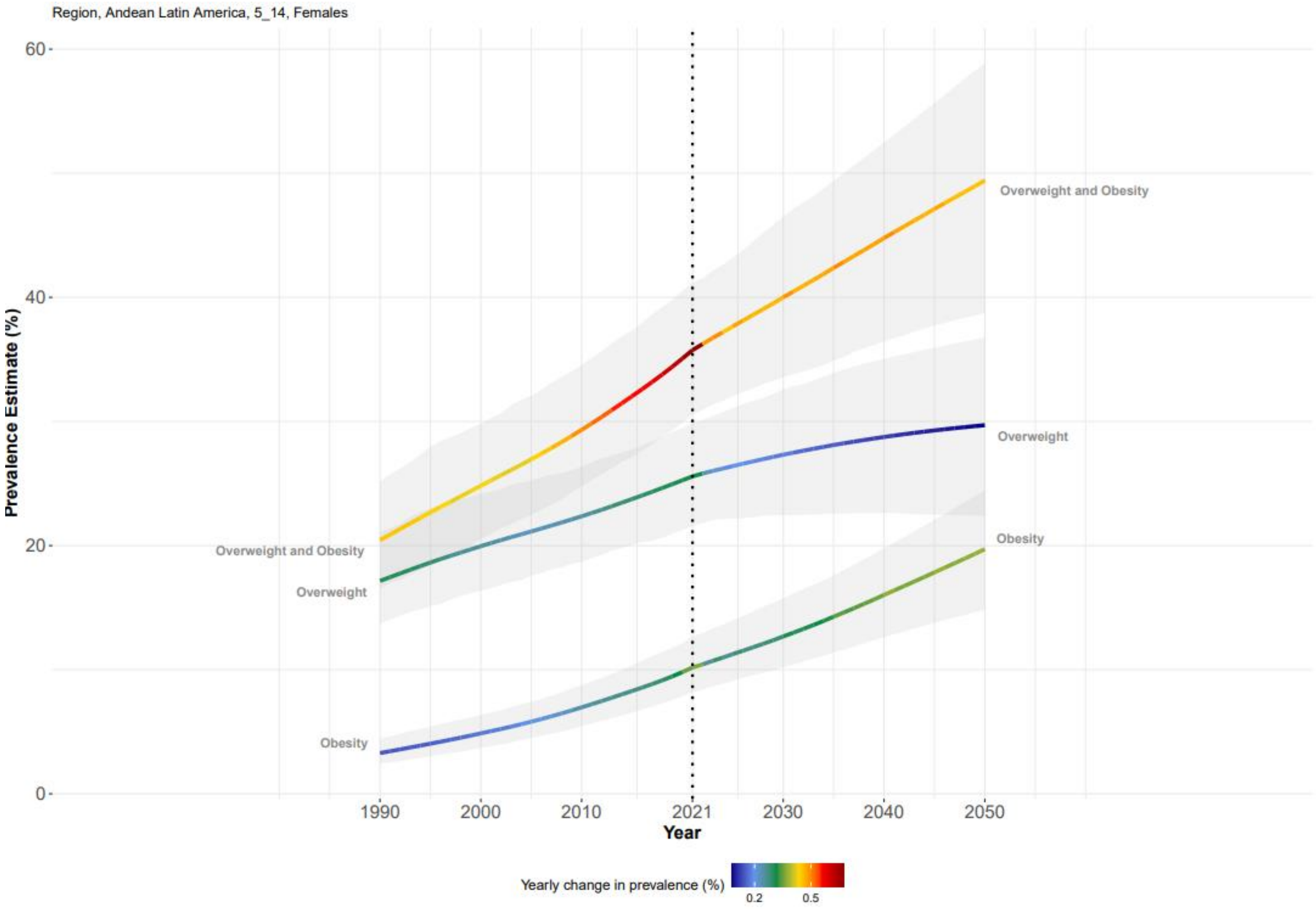
Region, Andean Latin America, 5_14, Females
Prevalence Estimate (%)
60
40
20
0
Overweight and Obesity
Overweight
Obesity
Overweight and Obesity
Overweight
Obesity
1990
2000
2010
2021
2030
2040
2050
Year
Yearly change in prevalence (%)
0.2
0.5

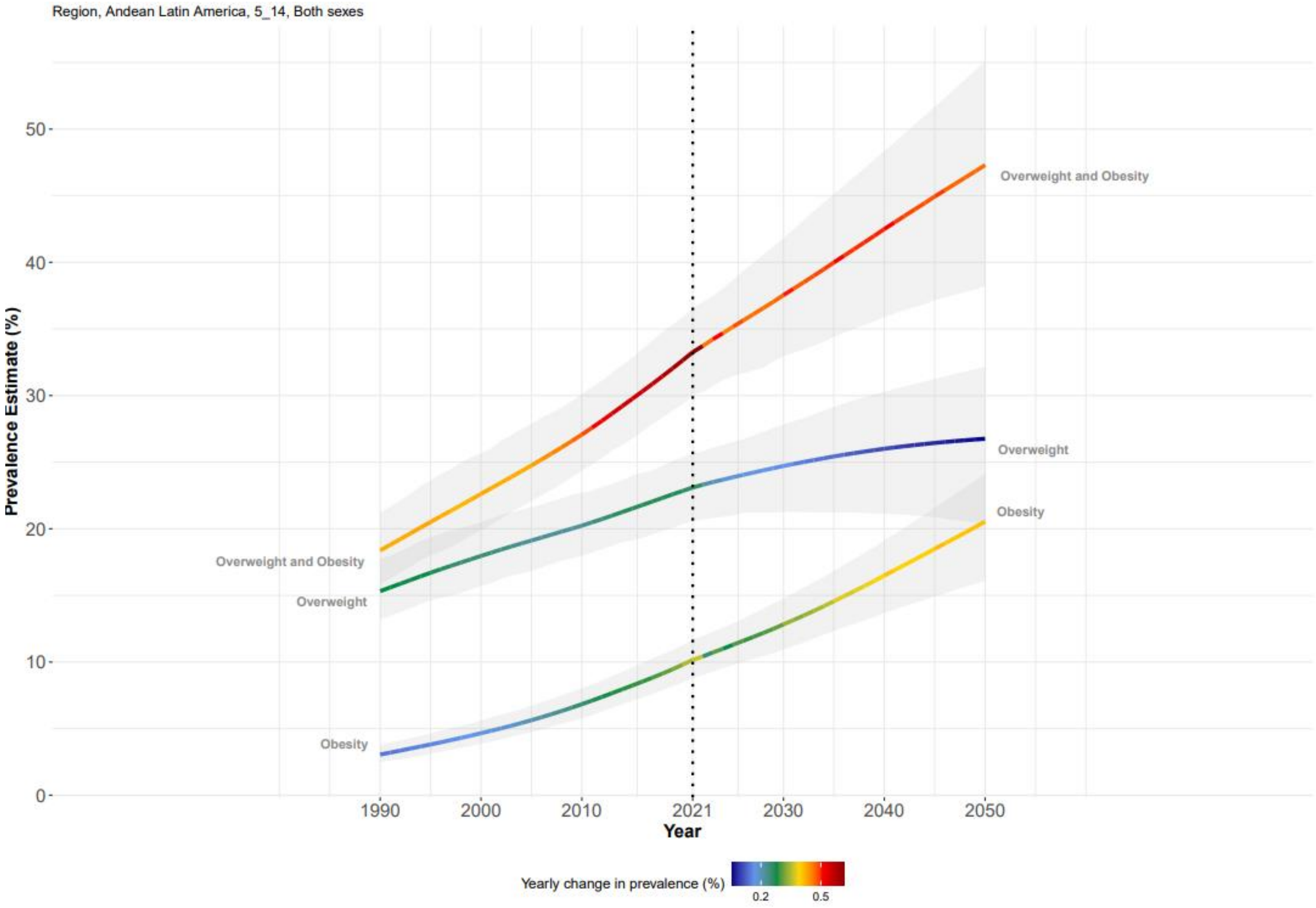
Region, Andean Latin America, 5_14, Both sexes
Prevalence Estimate (%)
50
40
30
20
10
0
1990
2000
2010
2021
2030
2040
2050
Year
Overweight and Obesity
Overweight
Obesity
Overweight and Obesity
Overweight
Obesity
Yearly change in prevalence (%)
0.2
0.5

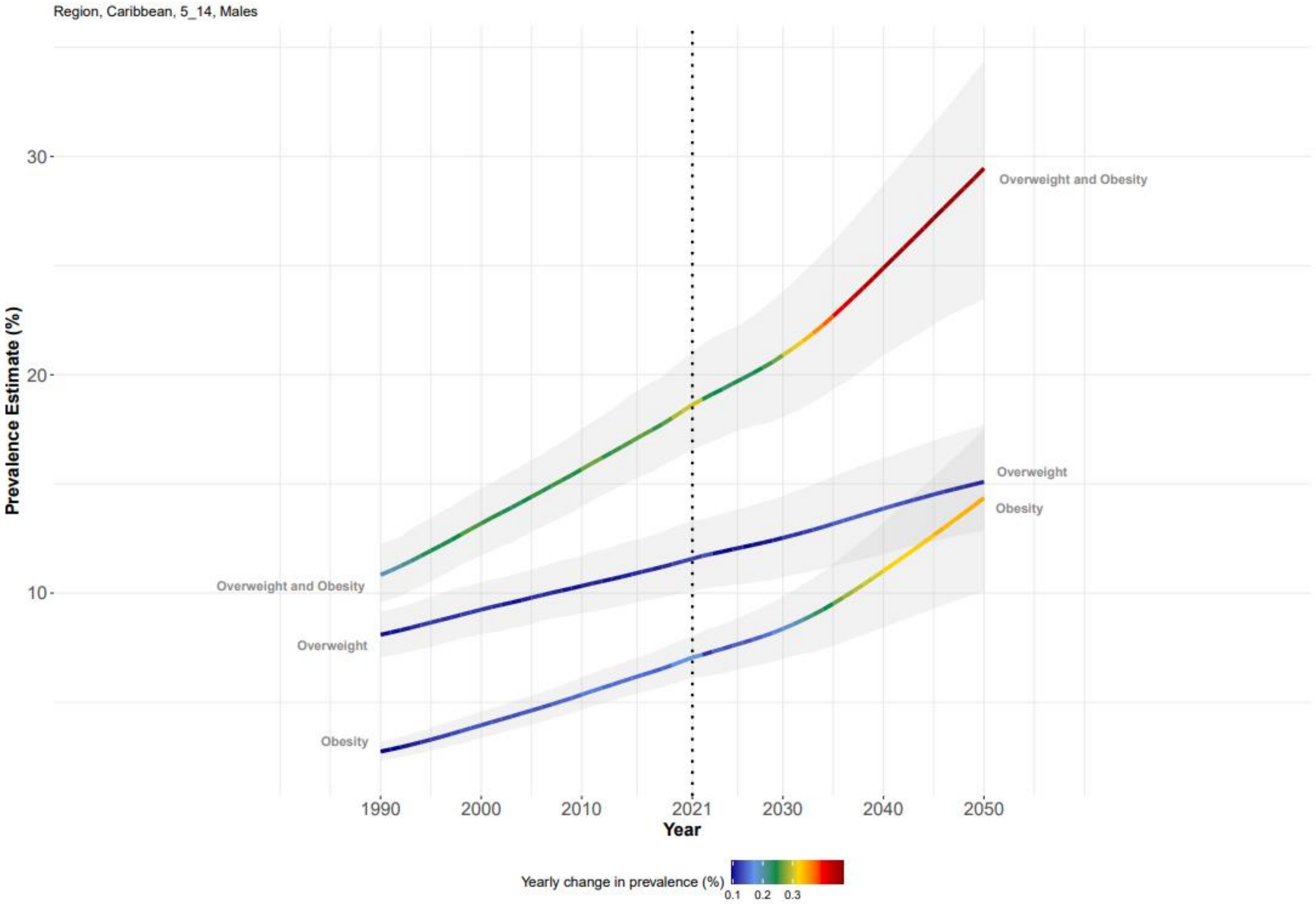
Region, Caribbean, 5_14, Males
Prevalence Estimate (%)
30
20
10
Overweight and Obesity
Overweight
Obesity
Overweight and Obesity
Overweight
Obesity
1990
2000
2010
2021
2030
2040
2050
Year
Yearly change in prevalence (%)
0.1
0.2
0.3

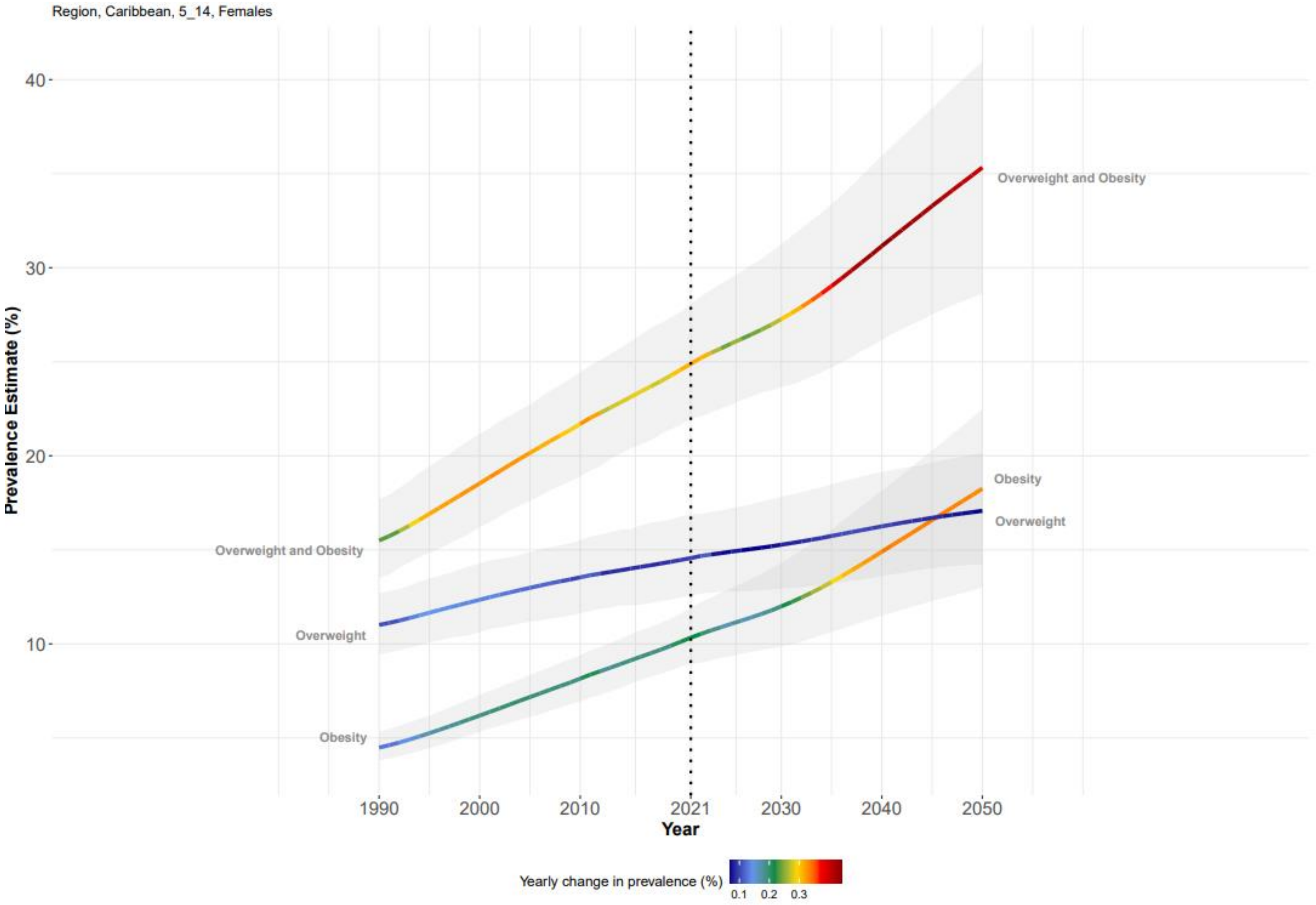

Region, Caribbean, 5_14, Females
Prevalence Estimate (%)
40
30
20
10
Overweight and Obesity
Overweight
Obesity
Overweight and Obesity
Obesity
Overweight
1990
2000
2010
2021
2030
2040
2050
Year
Yearly change in prevalence (%)
0.1
0.2
0.3

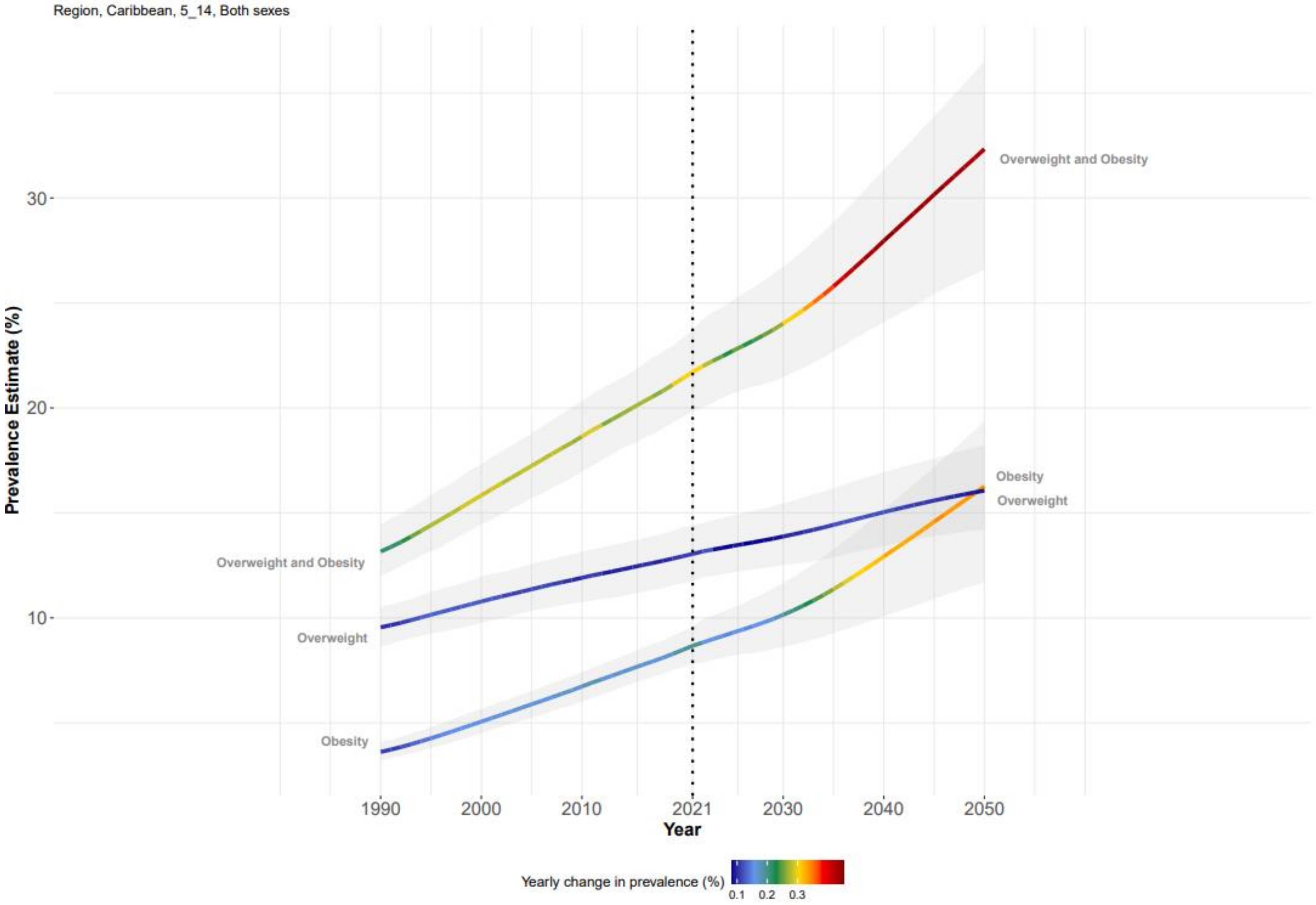

Region, Caribbean, 5_14, Both sexes
Prevalence Estimate (%)
30
20
10
1990
2000
2010
2021
2030
2040
2050
Year
Overweight and Obesity
Overweight
Obesity
Overweight and Obesity
Obesity
Overweight
Yearly change in prevalence (%)
0.1
0.2
0.3

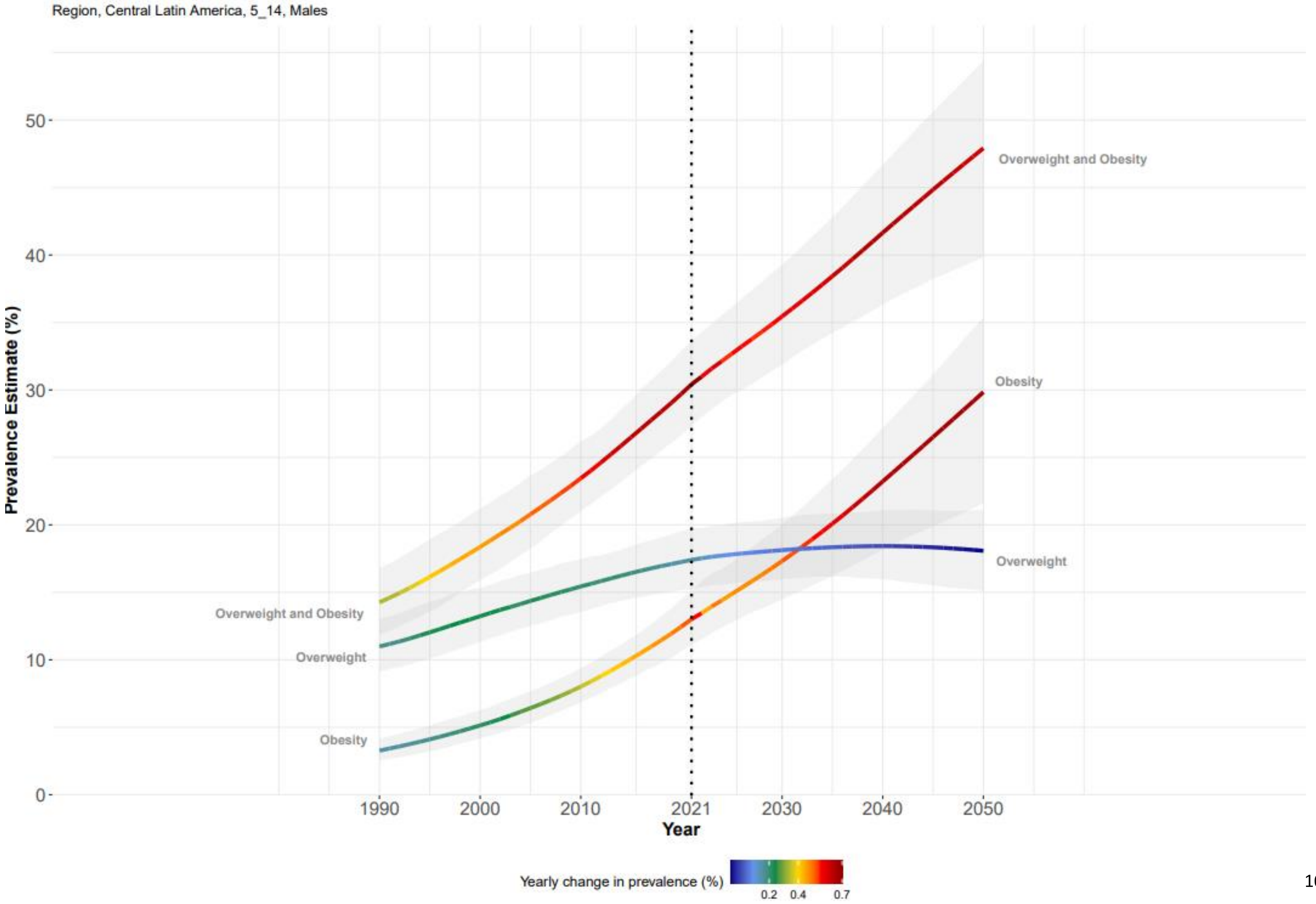
Region, Central Latin America, 5_14, Males
Prevalence Estimate (%)
50
40
30
20
10
0
1990
2000
2010
2021
2030
2040
2050
Year
Overweight and Obesity
Obesity
Overweight
Overweight and Obesity
Overweight
Obesity
Yearly change in prevalence (%)
0.2
0.4
0.7

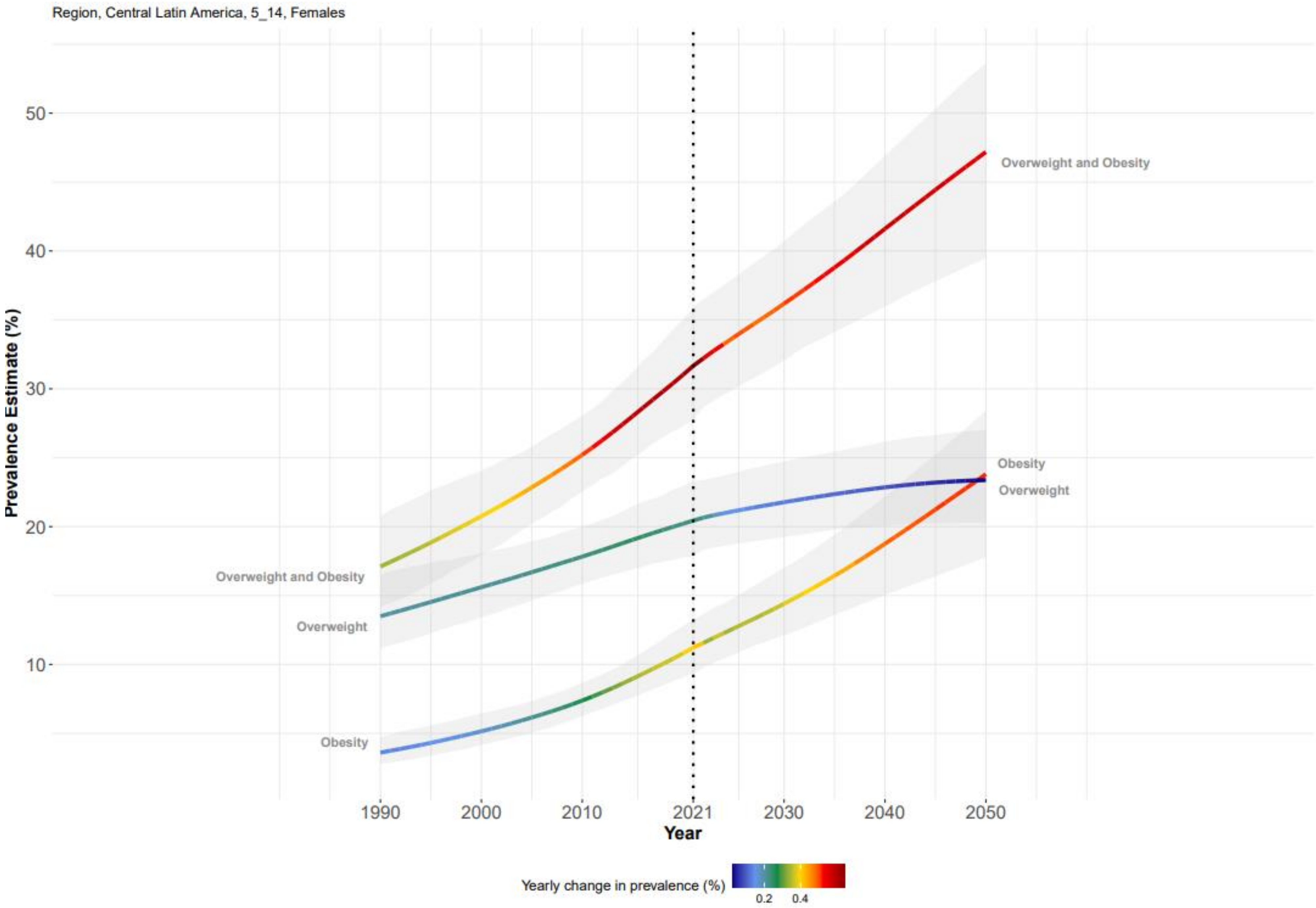

Region, Central Latin America, 5_14, Females
Prevalence Estimate (%)
50
40
30
20
10
Overweight and Obesity
Obesity
Overweight
Overweight and Obesity
Overweight
Obesity
1990
2000
2010
2021
2030
2040
2050
Year
Yearly change in prevalence (%)
0.2
0.4

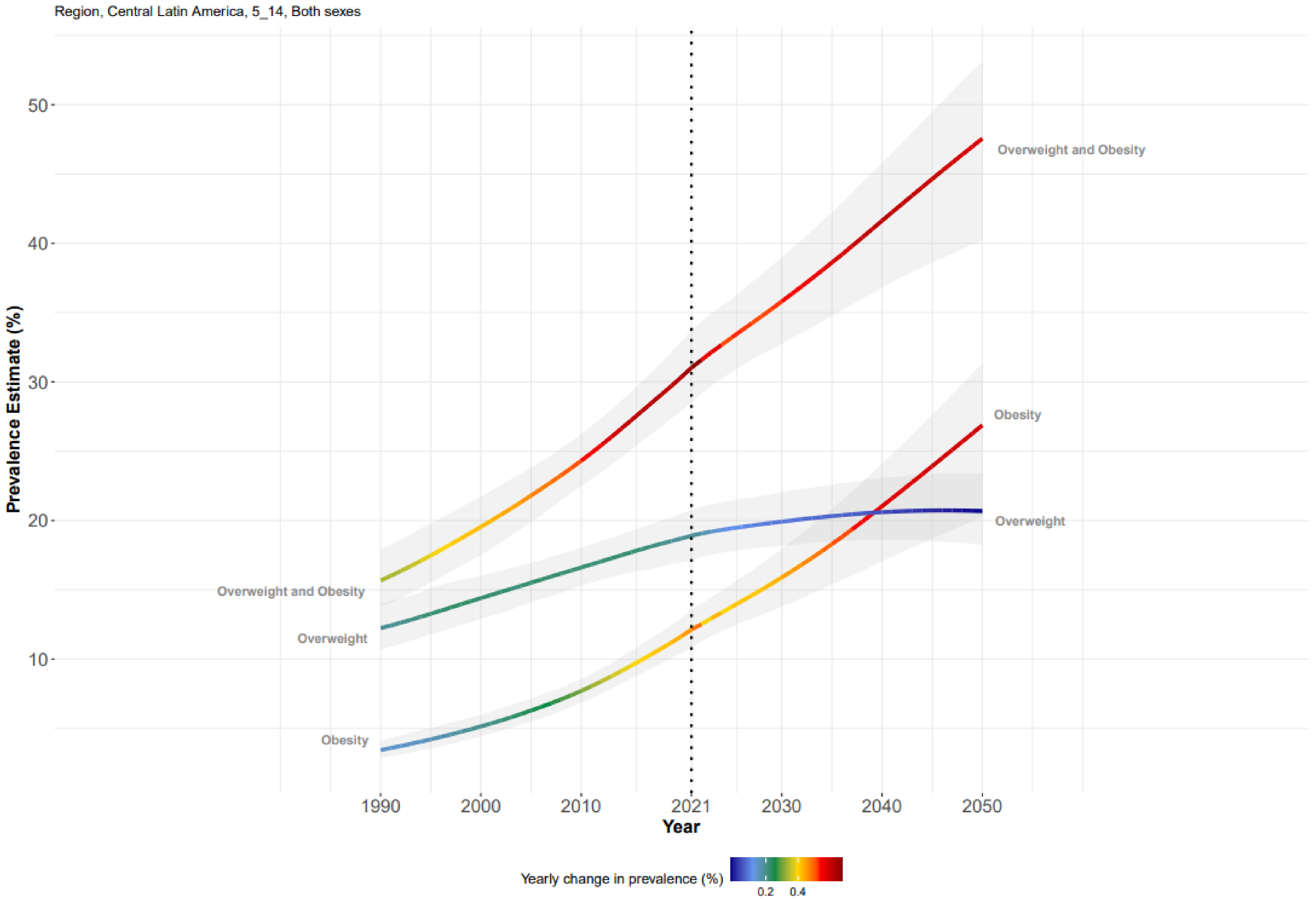

Region, Central Latin America, 5_14, Both sexes
Prevalence Estimate (%)
50
40
30
20
10
Overweight and Obesity
Obesity
Overweight
Overweight and Obesity
Overweight
Obesity
1990
2000
2010
2021
2030
2040
2050
Year
Yearly change in prevalence (%)
0.2
0.4

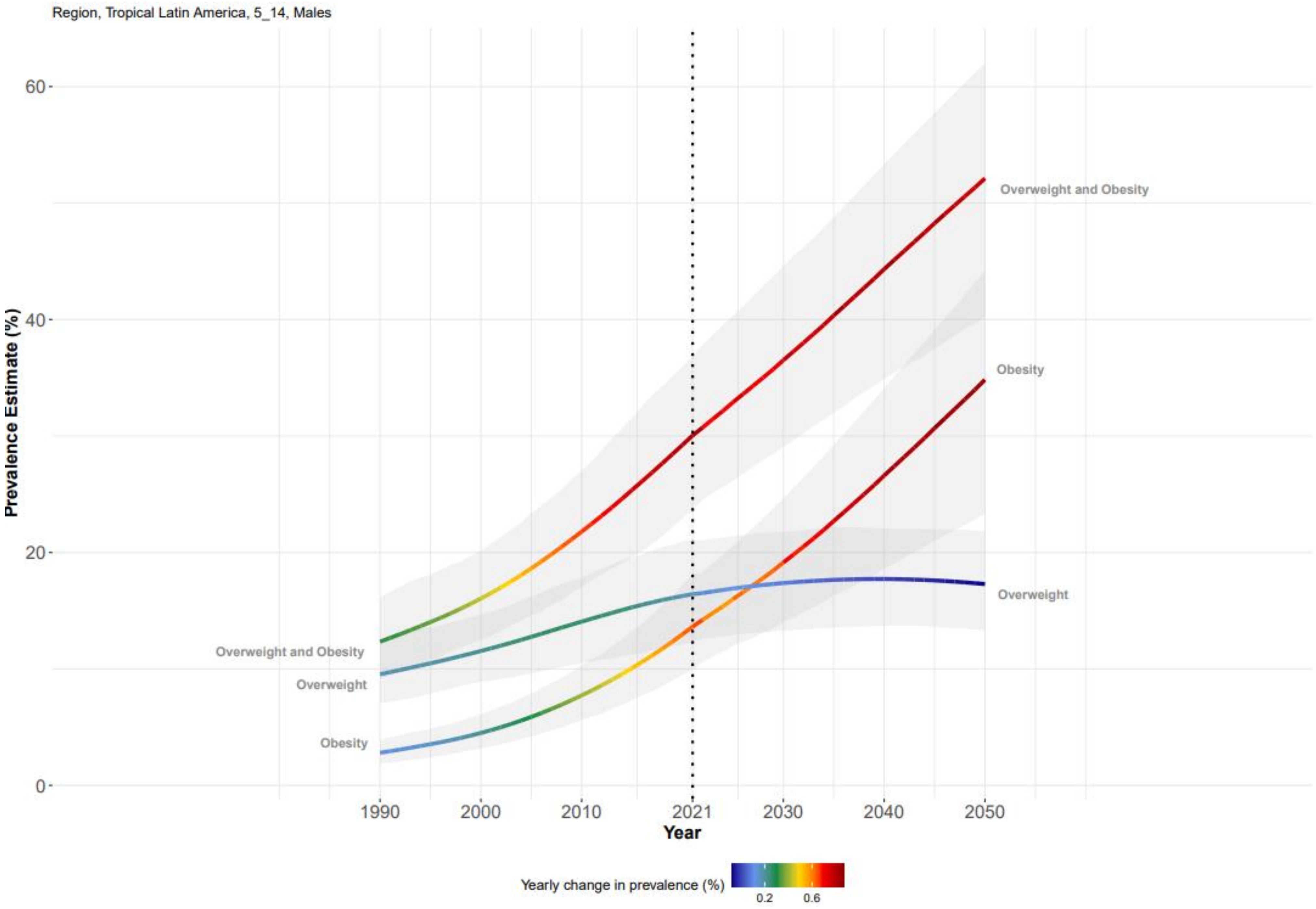

Region, Tropical Latin America, 5_14, Males
Prevalence Estimate (%)
60
40
20
0
1990
2000
2010
2021
2030
2040
2050
Year
Overweight and Obesity
Obesity
Overweight
Overweight and Obesity
Overweight
Obesity
Yearly change in prevalence (%)
0.2
0.6

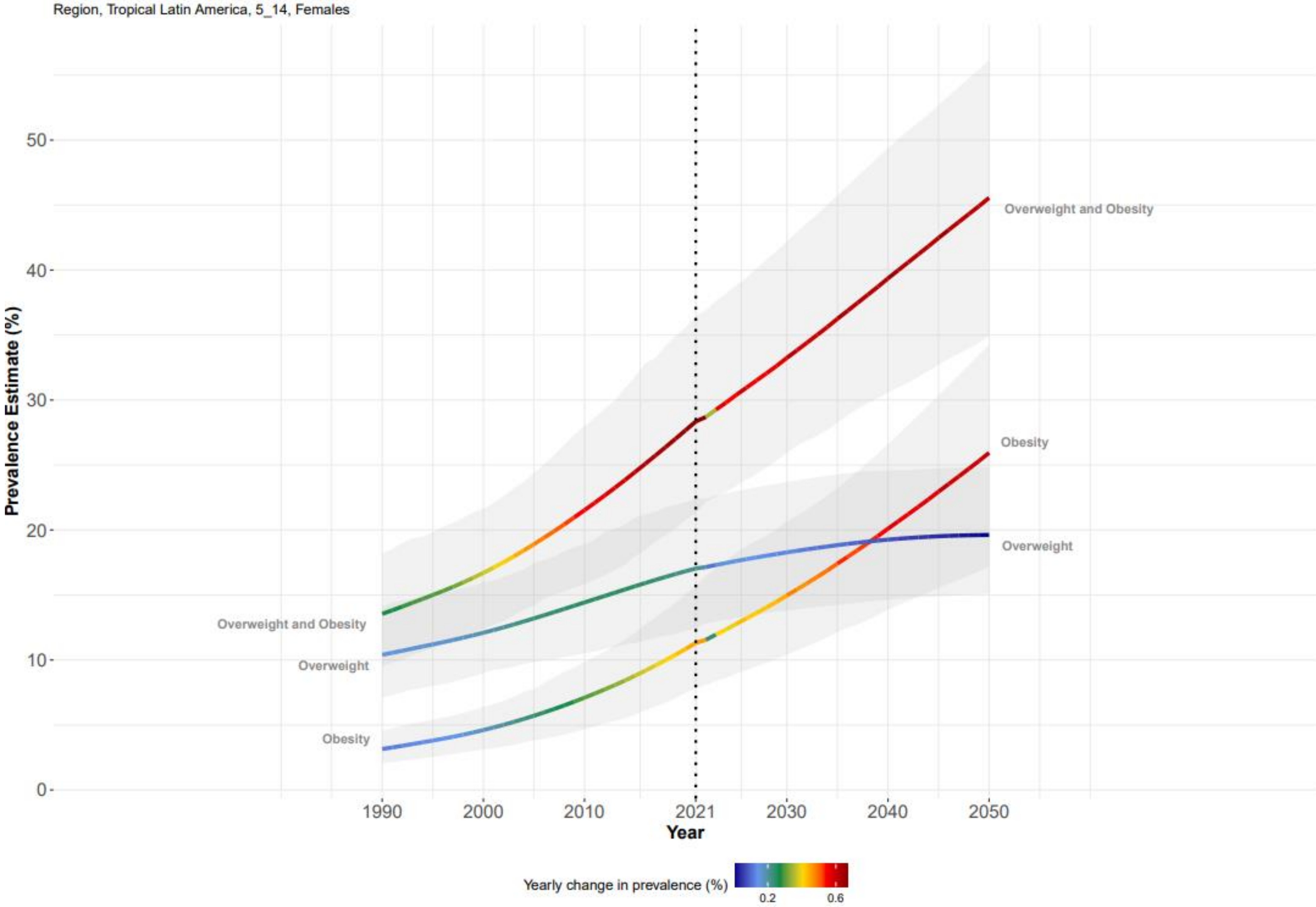

Region, Tropical Latin America, 5_14, Females
Prevalence Estimate (%)
0
10
20
30
40
50
1990
2000
2010
2021
2030
2040
2050
Year
Overweight and Obesity
Obesity
Overweight
Overweight and Obesity
Overweight
Obesity
Yearly change in prevalence (%)
0.2
0.6

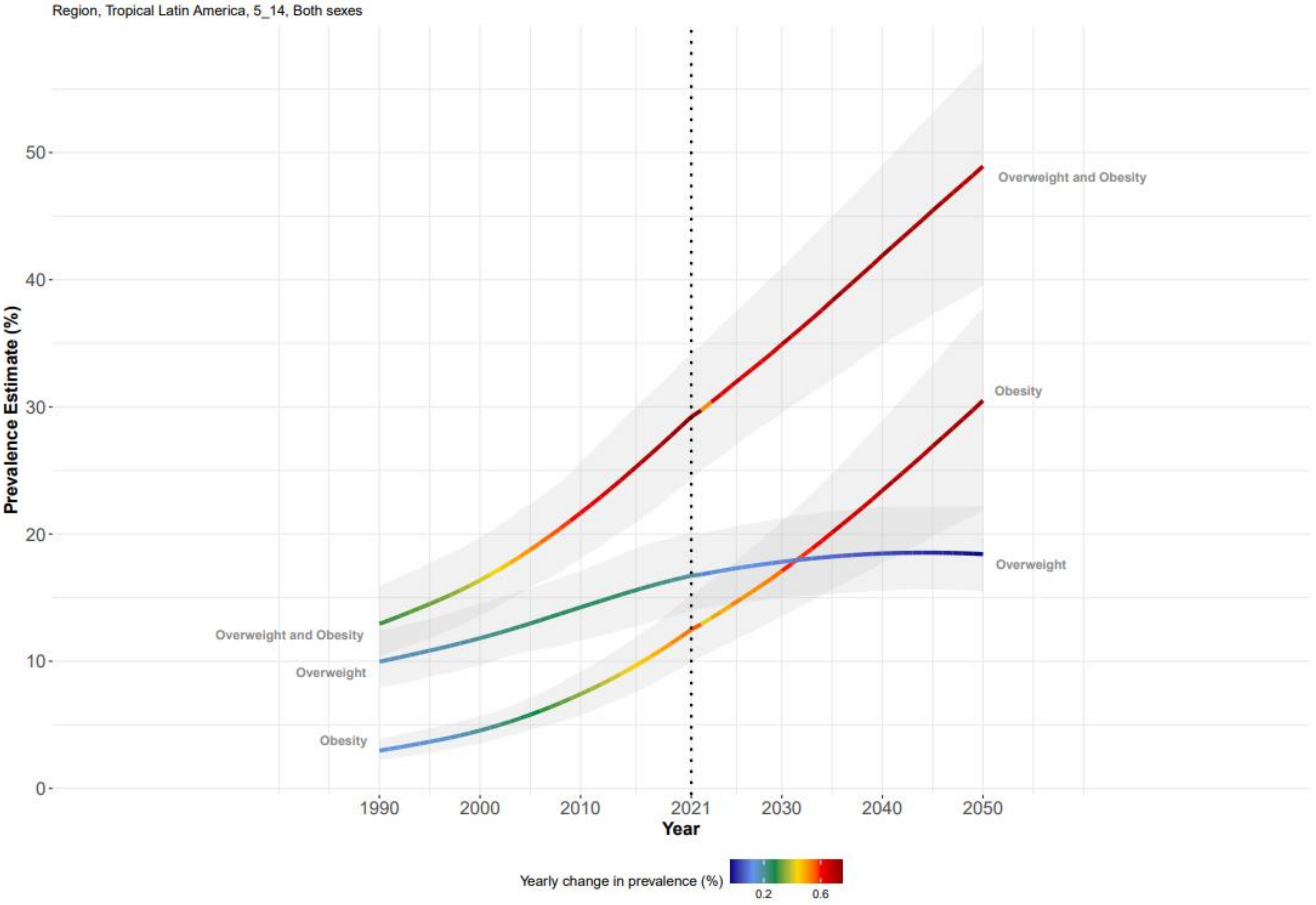

Region, Tropical Latin America, 5_14, Both sexes
Prevalence Estimate (%)
0
10
20
30
40
50
1990
2000
2010
2021
2030
2040
2050
Year
Overweight and Obesity
Overweight
Obesity
Overweight and Obesity
Obesity
Overweight
Yearly change in prevalence (%)
0.2
0.6

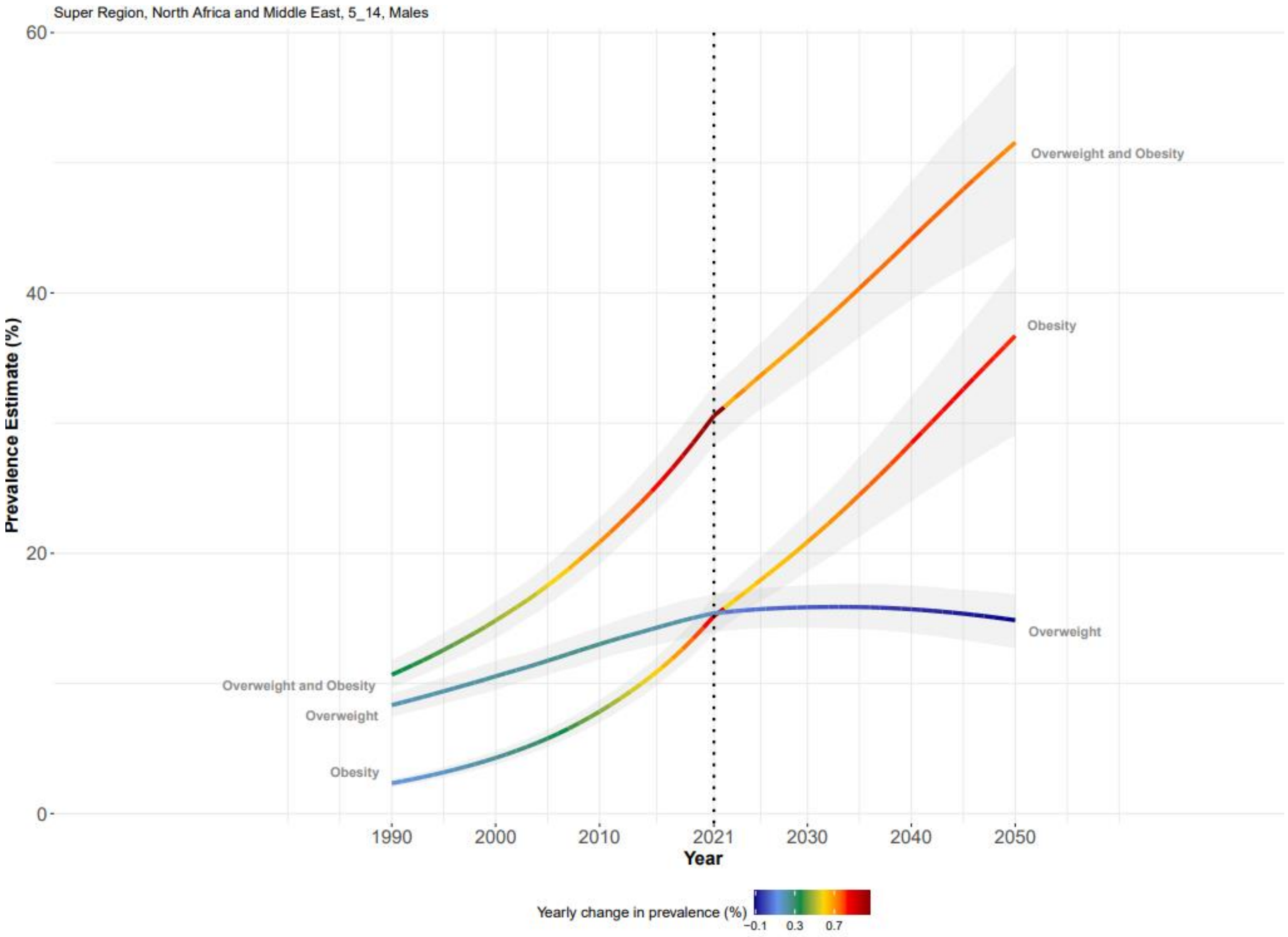
Super Region, North Africa and Middle East, 5_14, Males
Prevalence Estimate (%)
60
40
20
0
1990
2000
2010
2021
2030
2040
2050
Year
Overweight and Obesity
Obesity
Overweight
Overweight and Obesity
Overweight
Obesity
Yearly change in prevalence (%)
−0.1
0.3
0.7

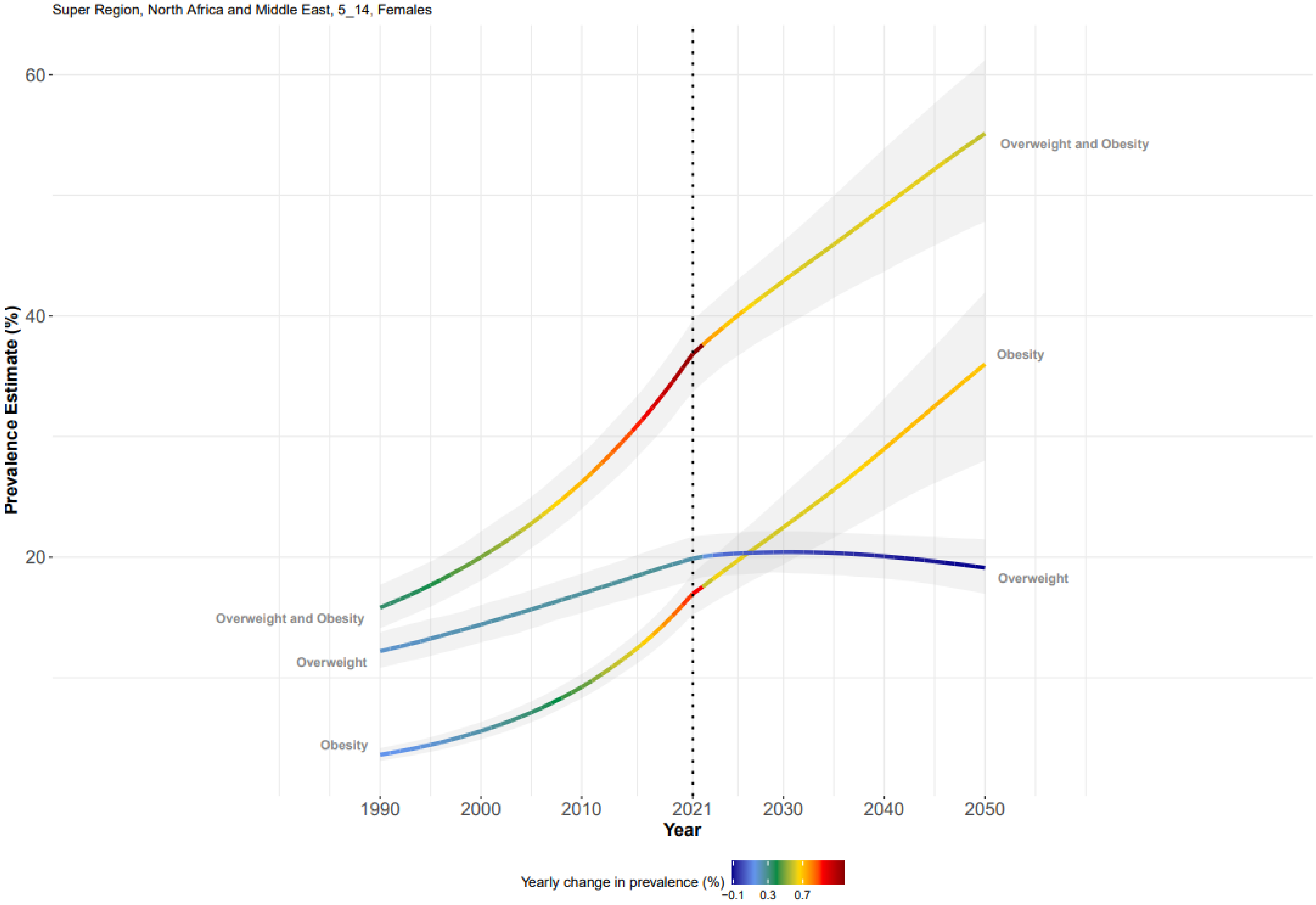

Super Region, North Africa and Middle East, 5_14, Females
Prevalence Estimate (%)
60
40
20
Overweight and Obesity
Overweight
Obesity
Overweight and Obesity
Obesity
Overweight
1990
2000
2010
2021
2030
2040
2050
Year
Yearly change in prevalence (%)
−0.1
0.3
0.7

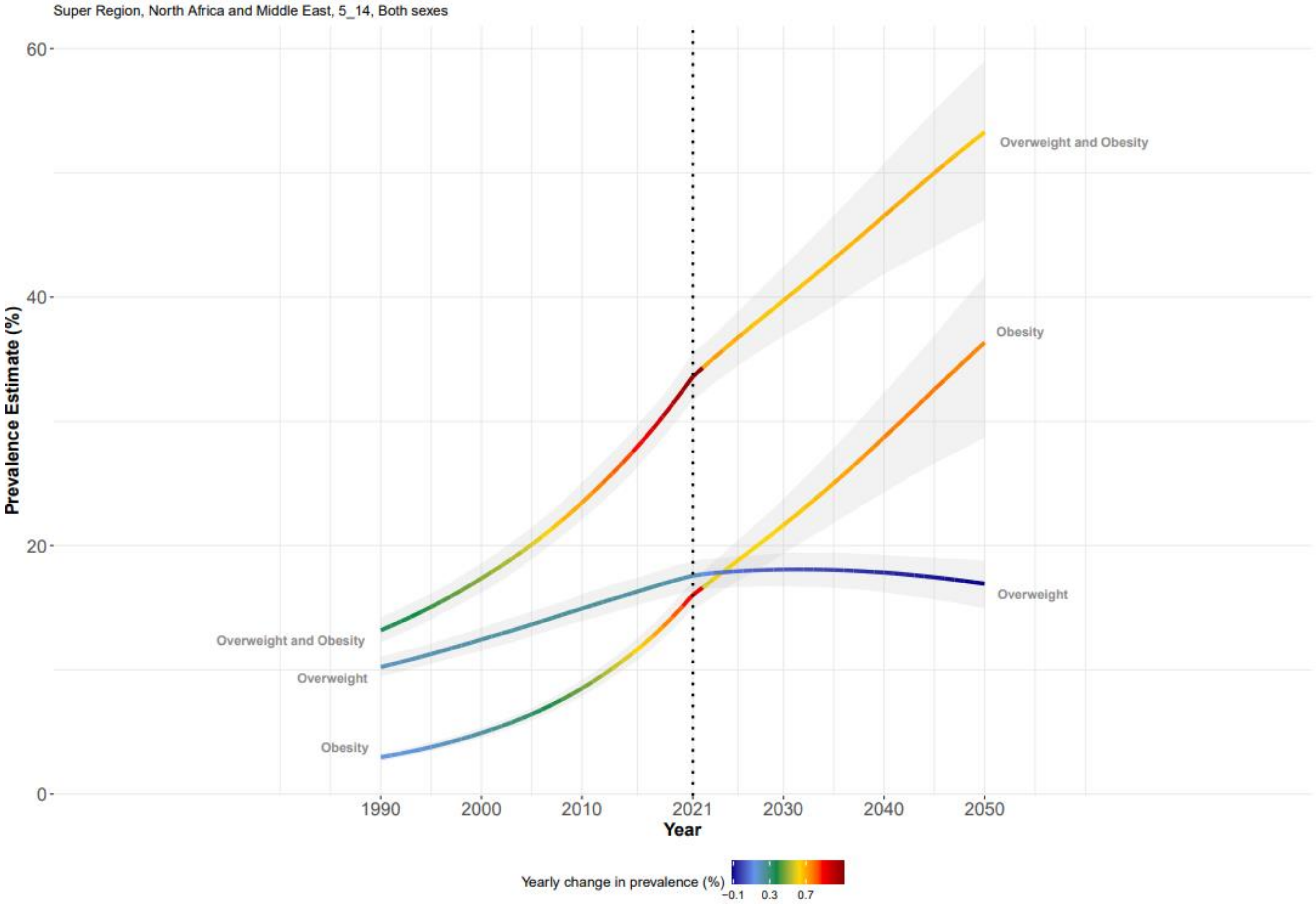

Super Region, North Africa and Middle East, 5_14, Both sexes
Prevalence Estimate (%)
60
40
20
0
Overweight and Obesity
Obesity
Overweight
Overweight and Obesity
Overweight
Obesity
1990
2000
2010
2021
2030
2040
2050
Year
Yearly change in prevalence (%)
−0.1
0.3
0.7

Super Region, Southeast Asia, East Asia, and Oceania, 5_14, Males

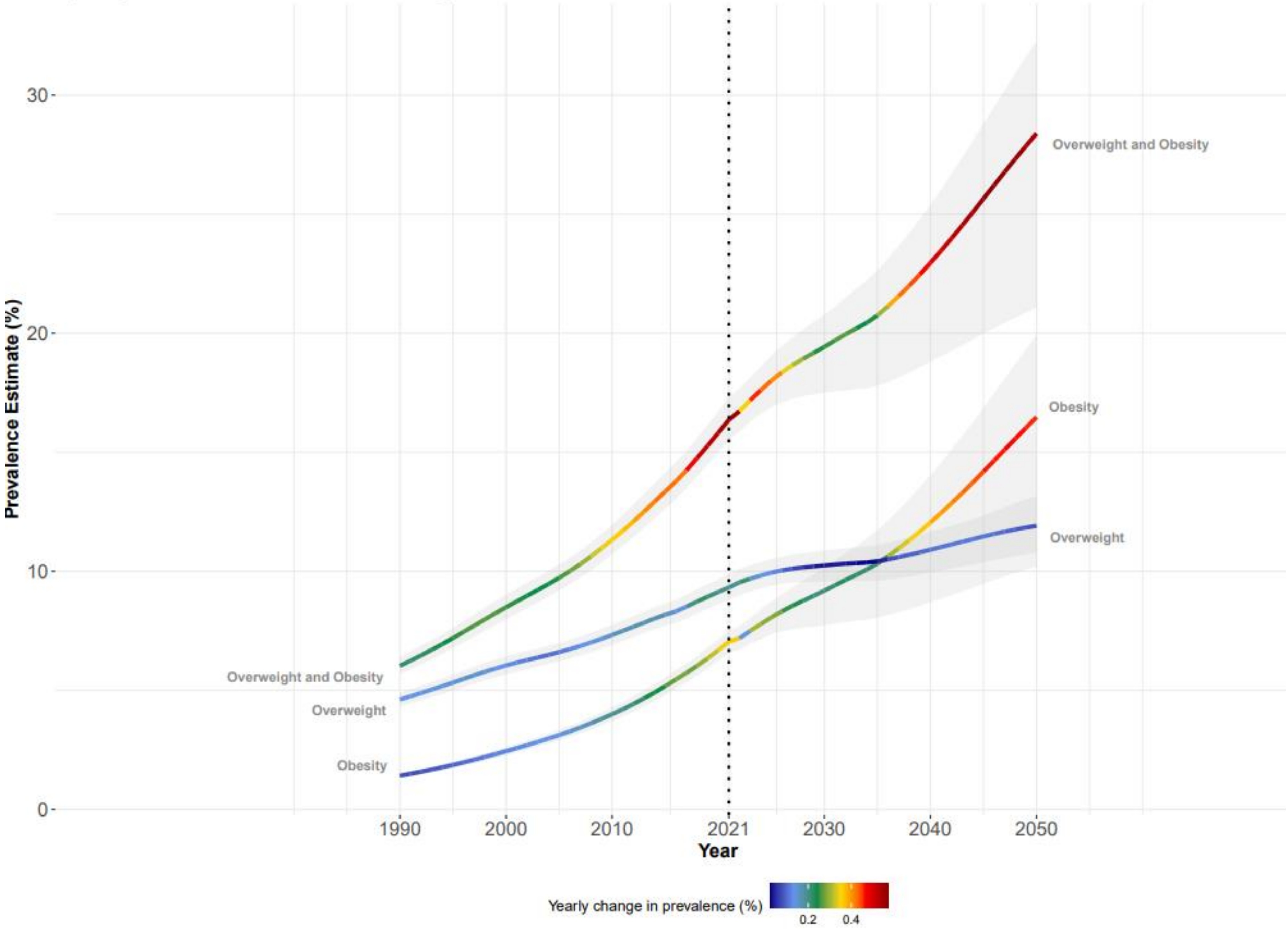

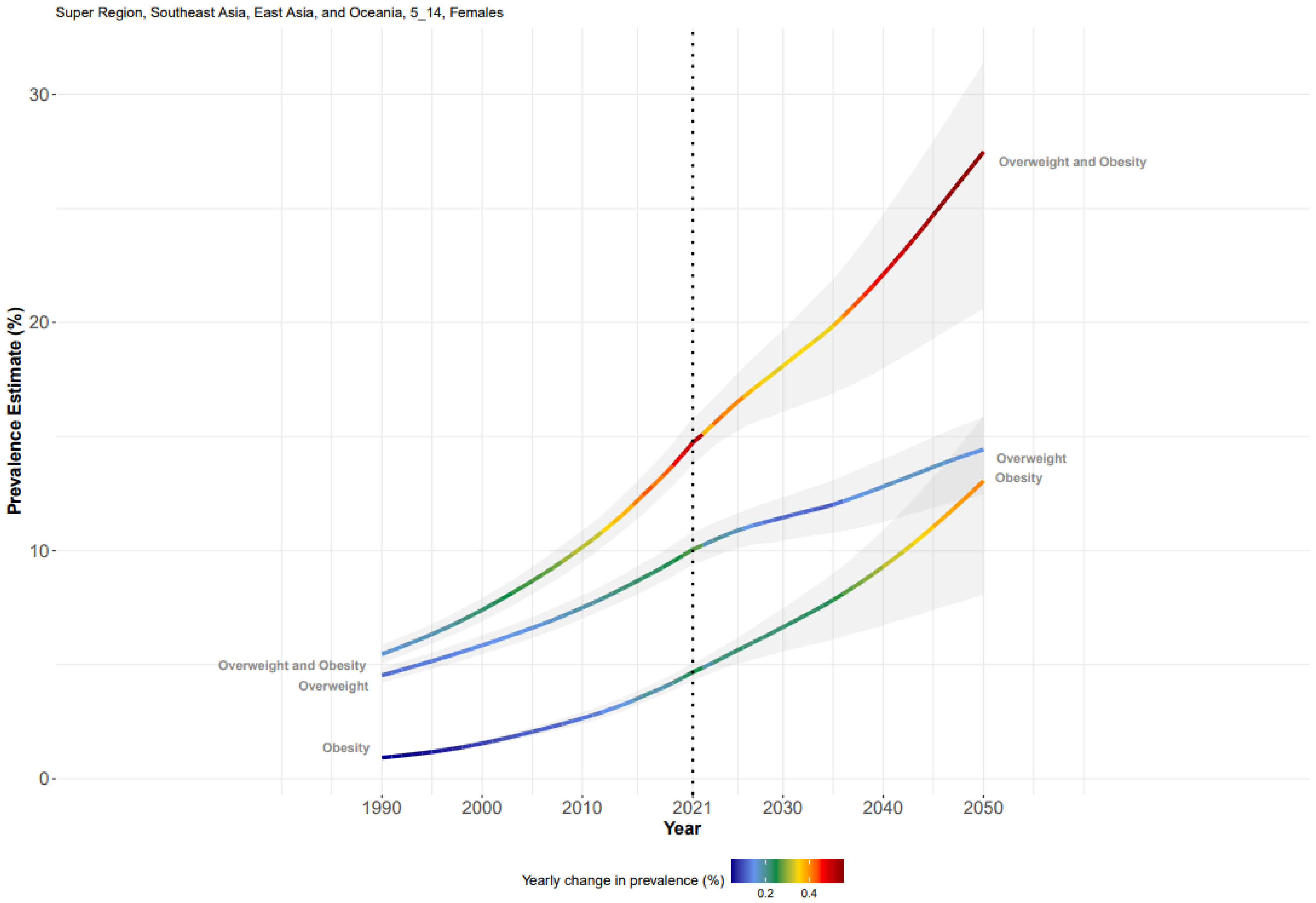

Super Region, Southeast Asia, East Asia, and Oceania, 5_14, Females
Prevalence Estimate (%)
30
20
10
0
Overweight and Obesity
Overweight
Obesity
Overweight and Obesity
Overweight
Obesity
1990
2000
2010
2021
2030
2040
2050
Year
Yearly change in prevalence (%)
0.2
0.4

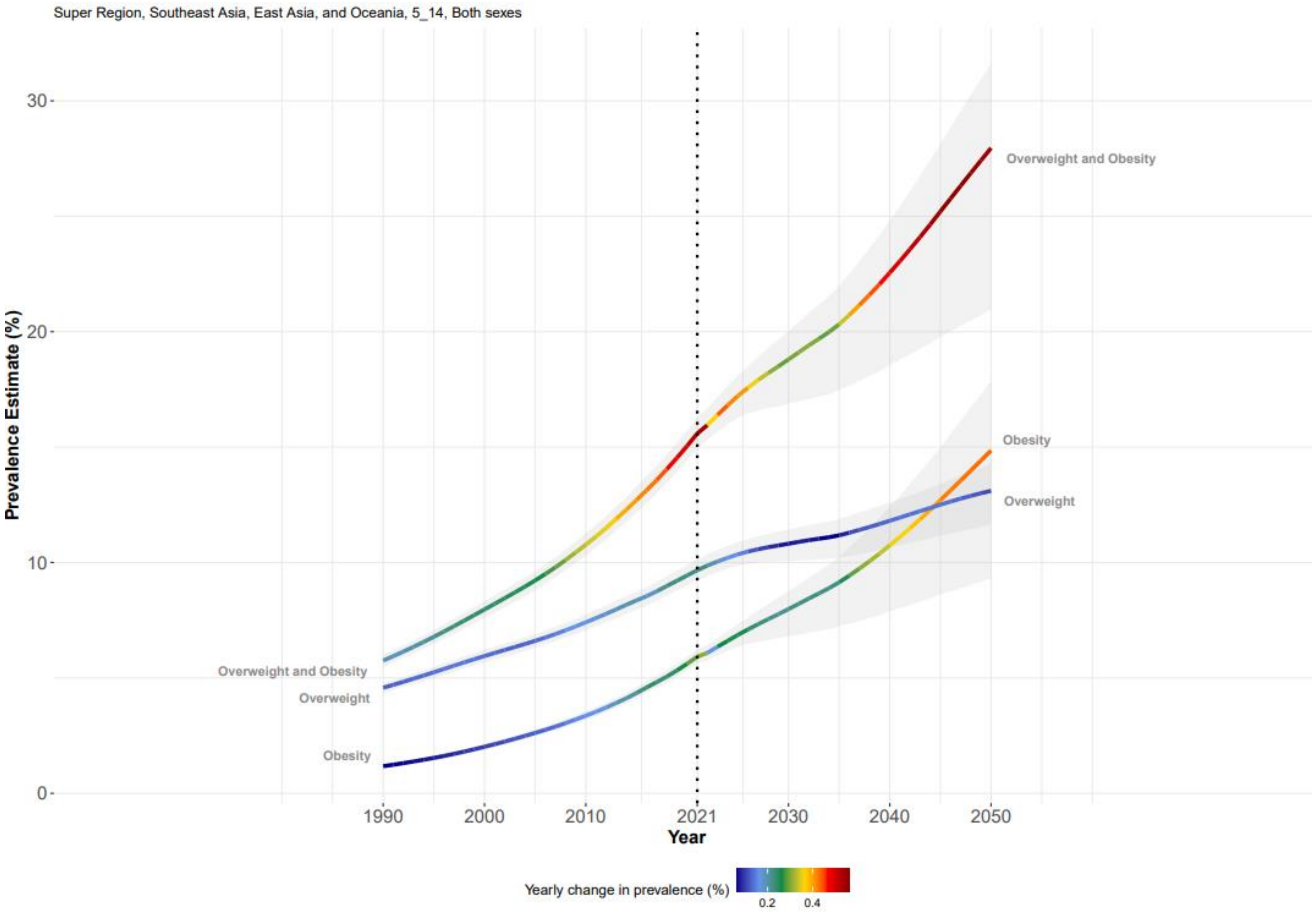

Super Region, Southeast Asia, East Asia, and Oceania, 5_14, Both sexes
30
20
10
0
Prevalence Estimate (%)
Overweight and Obesity
Obesity
Overweight
Overweight and Obesity
Overweight
Obesity
1990
2000
2010
2021
2030
2040
2050
Year
Yearly change in prevalence (%)
0.2
0.4

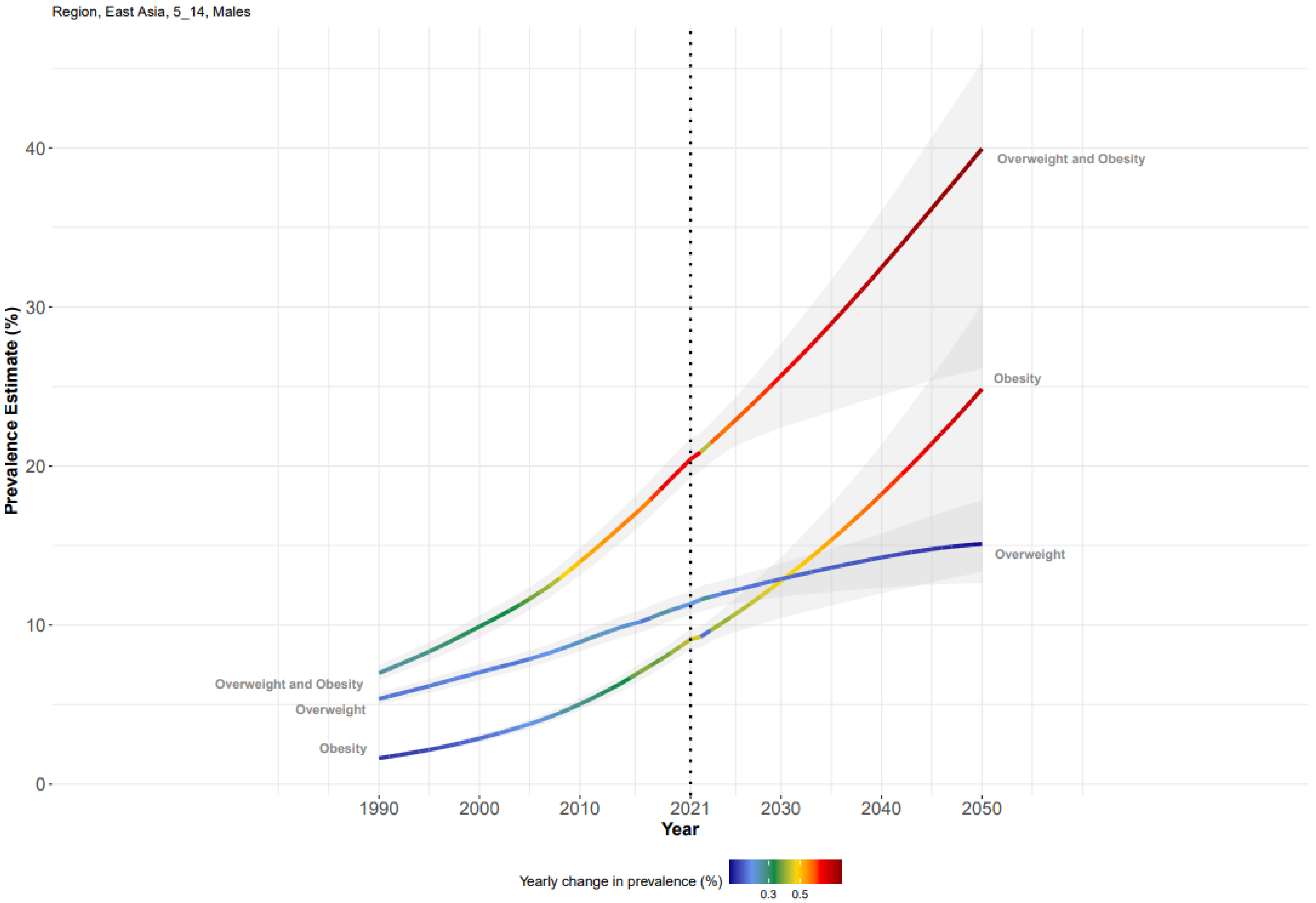

Region, East Asia, 5_14, Males
Prevalence Estimate (%)
40
30
20
10
0
1990
2000
2010
2021
2030
2040
2050
Year
Overweight and Obesity
Obesity
Overweight
Overweight and Obesity
Overweight
Obesity
Yearly change in prevalence (%)
0.3
0.5

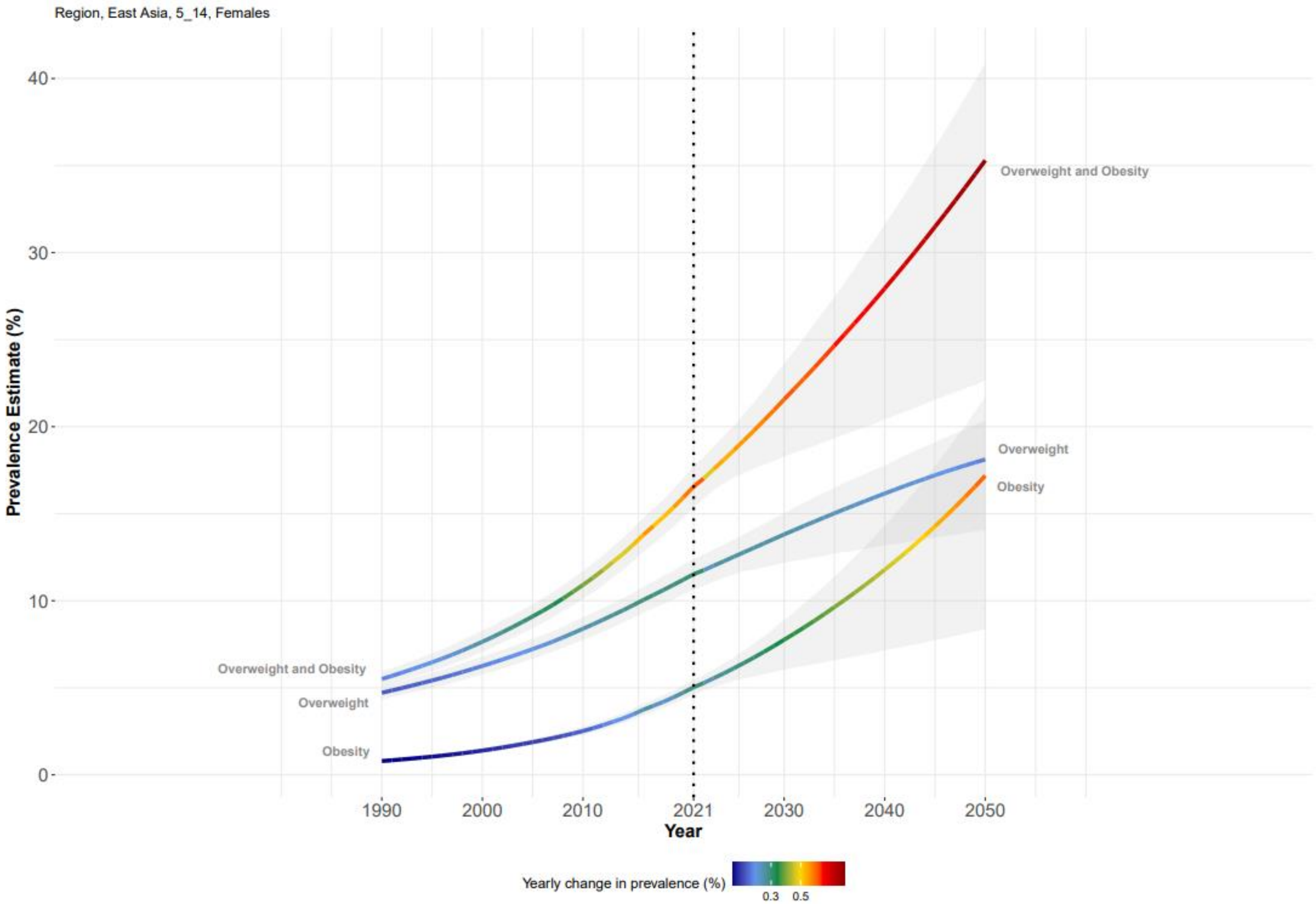
Region, East Asia, 5_14, Females
Prevalence Estimate (%)
40
30
20
10
0
Overweight and Obesity
Overweight
Obesity
Overweight and Obesity
Overweight
Obesity
1990
2000
2010
2021
2030
2040
2050
Year
Yearly change in prevalence (%)
0.3
0.5

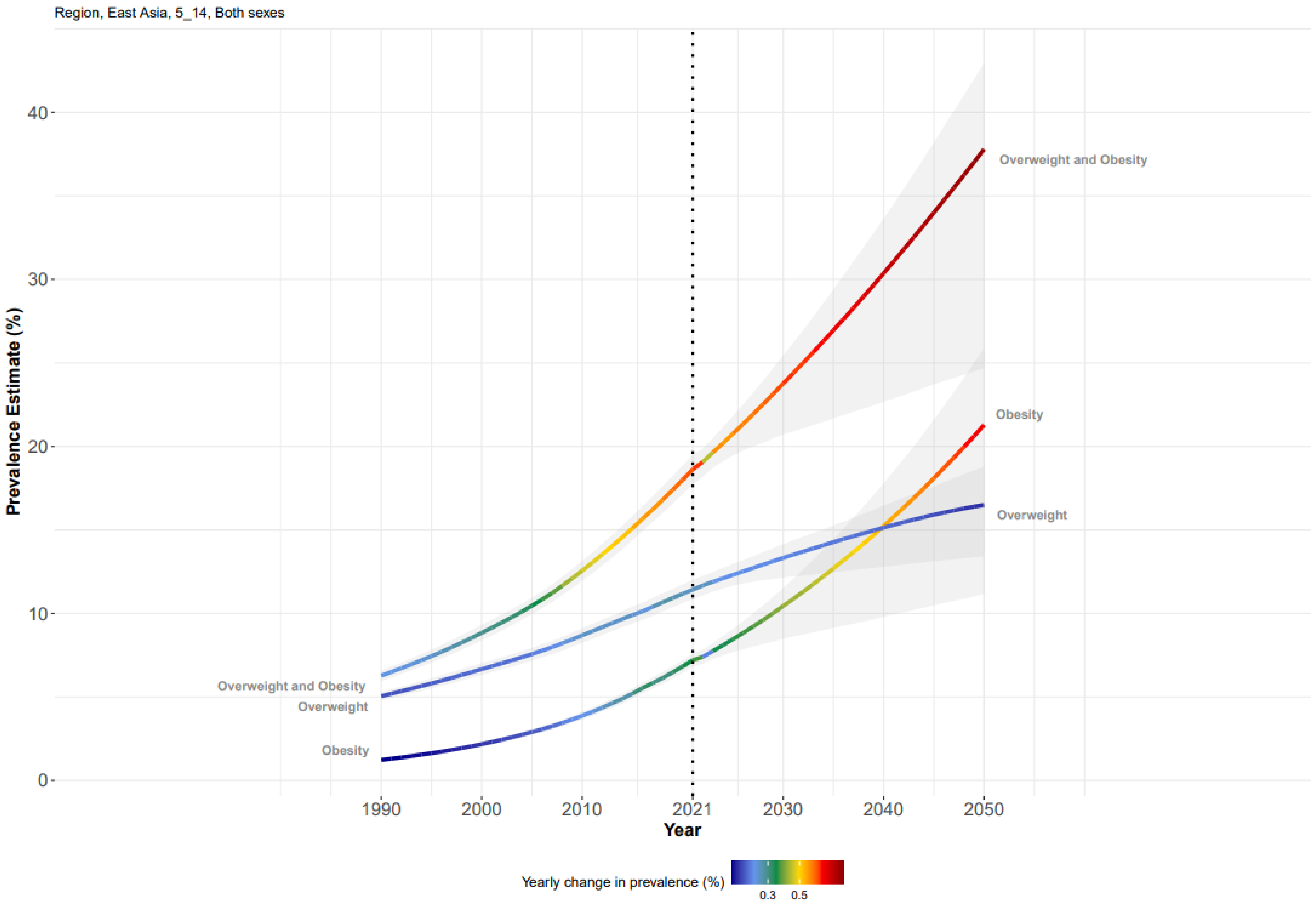

Region, East Asia, 5_14, Both sexes
Prevalence Estimate (%)
40
30
20
10
0
1990
2000
2010
2021
2030
2040
2050
Year
Overweight and Obesity
Obesity
Overweight
Overweight and Obesity
Overweight
Obesity
Yearly change in prevalence (%)
0.3
0.5

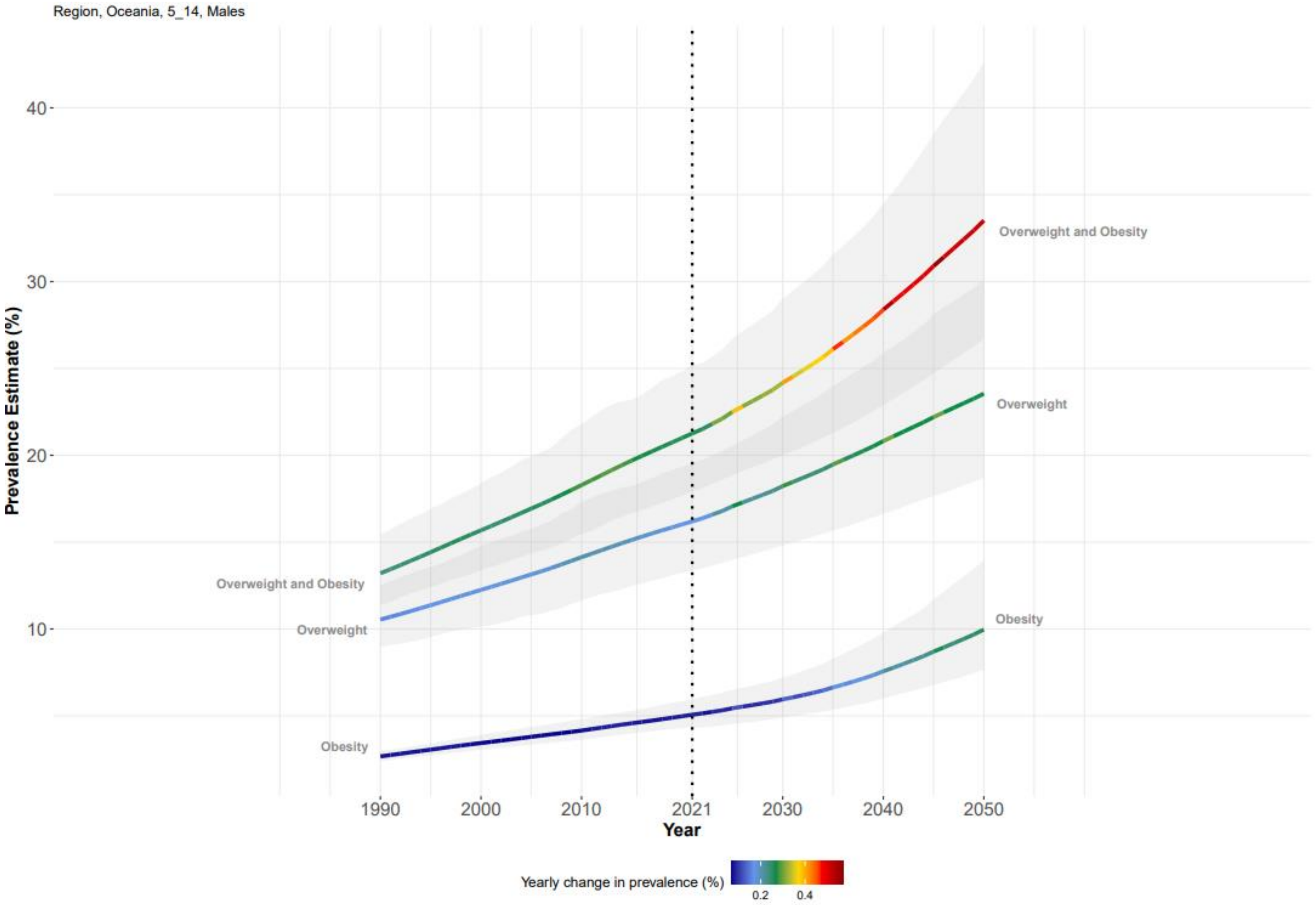

Region, Oceania, 5_14, Males
Prevalence Estimate (%)
40
30
20
10
Overweight and Obesity
Overweight
Obesity
Overweight and Obesity
Overweight
Obesity
1990
2000
2010
2021
2030
2040
2050
Year
Yearly change in prevalence (%)
0.2
0.4

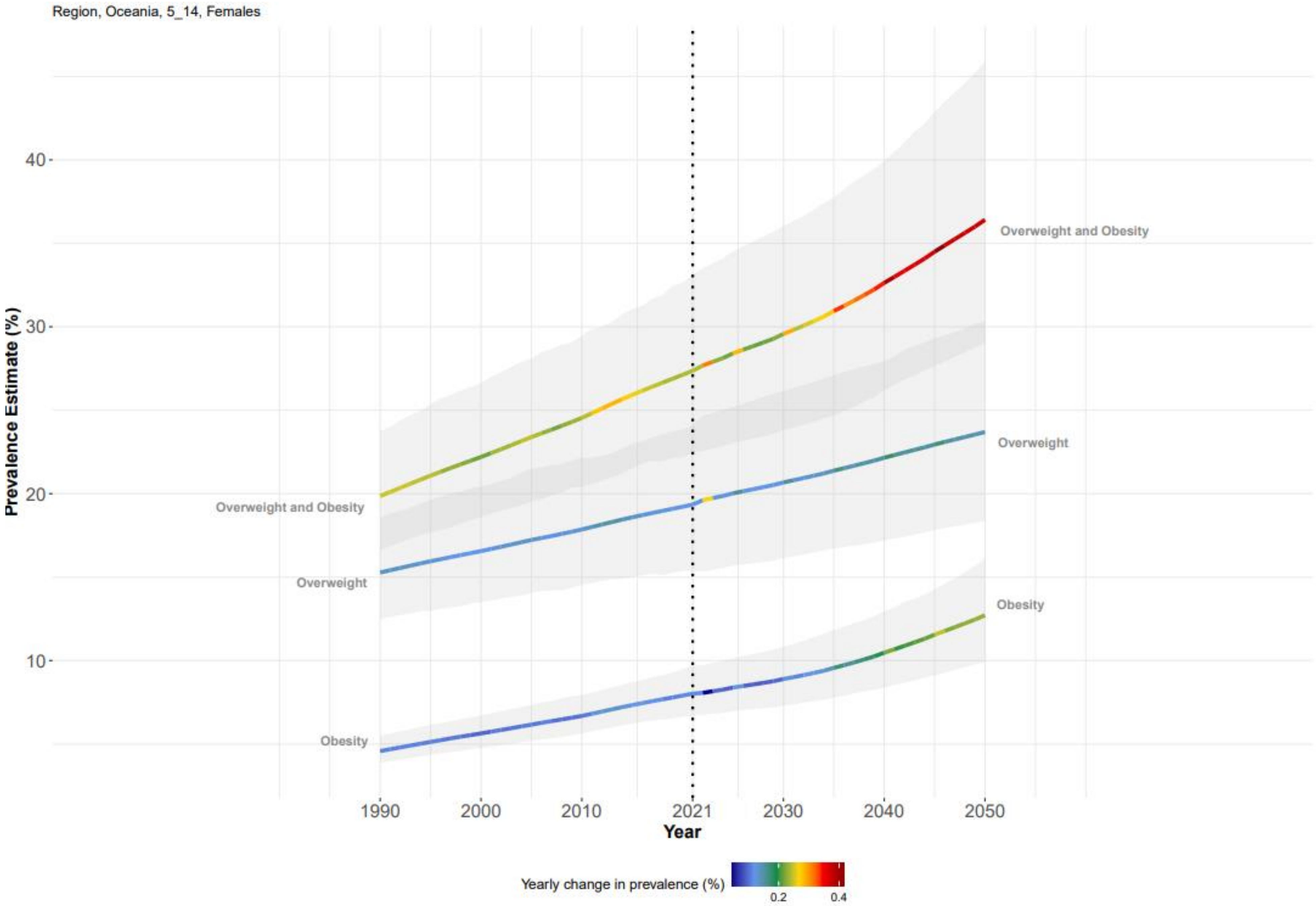

Region, Oceania, 5_14, Females
Prevalence Estimate (%)
40
30
20
10
Overweight and Obesity
Overweight
Obesity
Overweight and Obesity
Overweight
Obesity
1990
2000
2010
2021
2030
2040
2050
Year
Yearly change in prevalence (%)
0.2
0.4

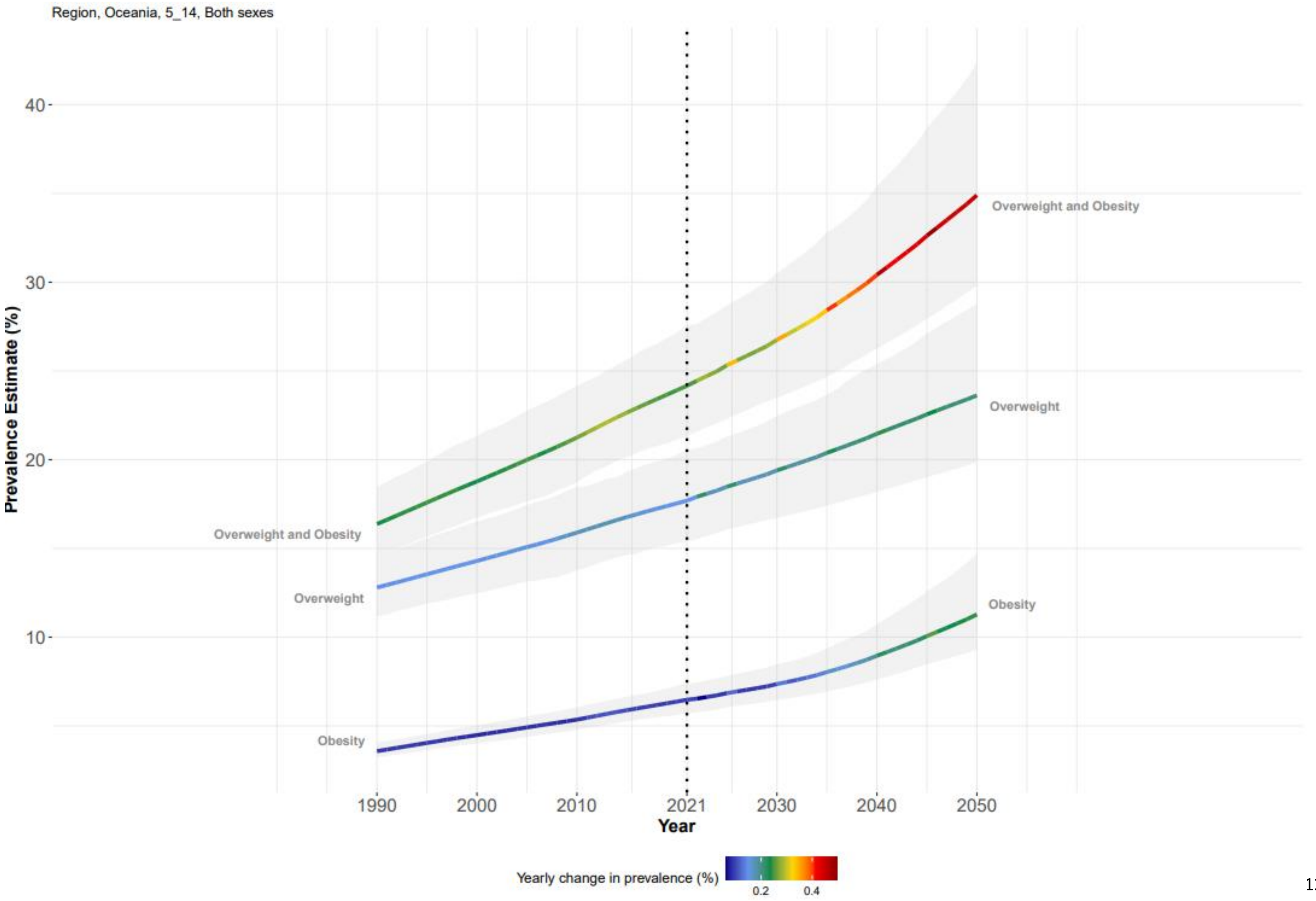

Region, Oceania, 5_14, Both sexes
Prevalence Estimate (%)
40
30
20
10
Overweight and Obesity
Overweight
Obesity
Overweight and Obesity
Overweight
Obesity
1990
2000
2010
2021
2030
2040
2050
Year
Yearly change in prevalence (%)
0.2
0.4

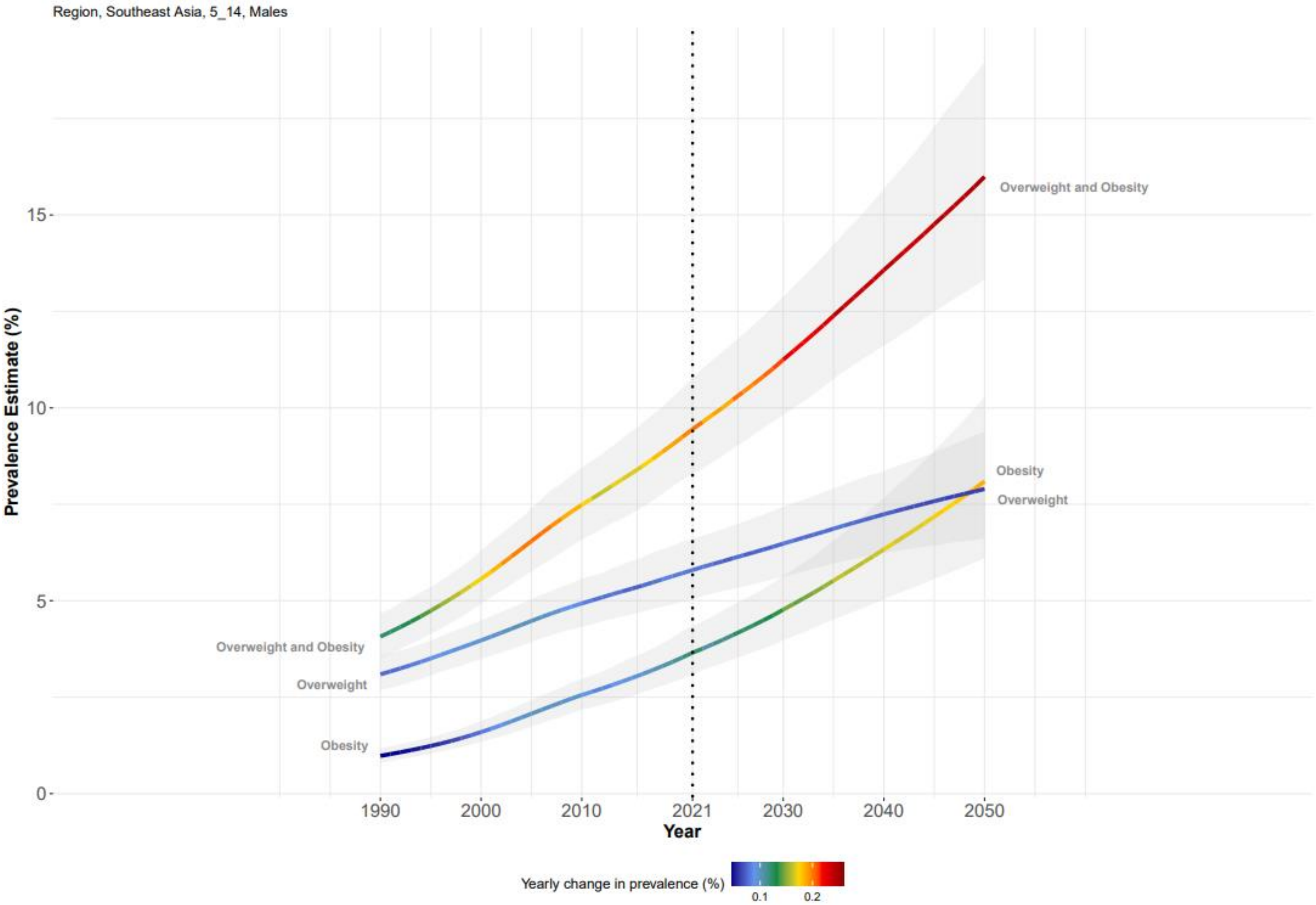

Region, Southeast Asia, 5_14, Males
Prevalence Estimate (%)
15
10
5
0
1990
2000
2010
2021
2030
2040
2050
Year
Overweight and Obesity
Overweight
Obesity
Overweight and Obesity
Obesity
Overweight
Yearly change in prevalence (%)
0.1
0.2

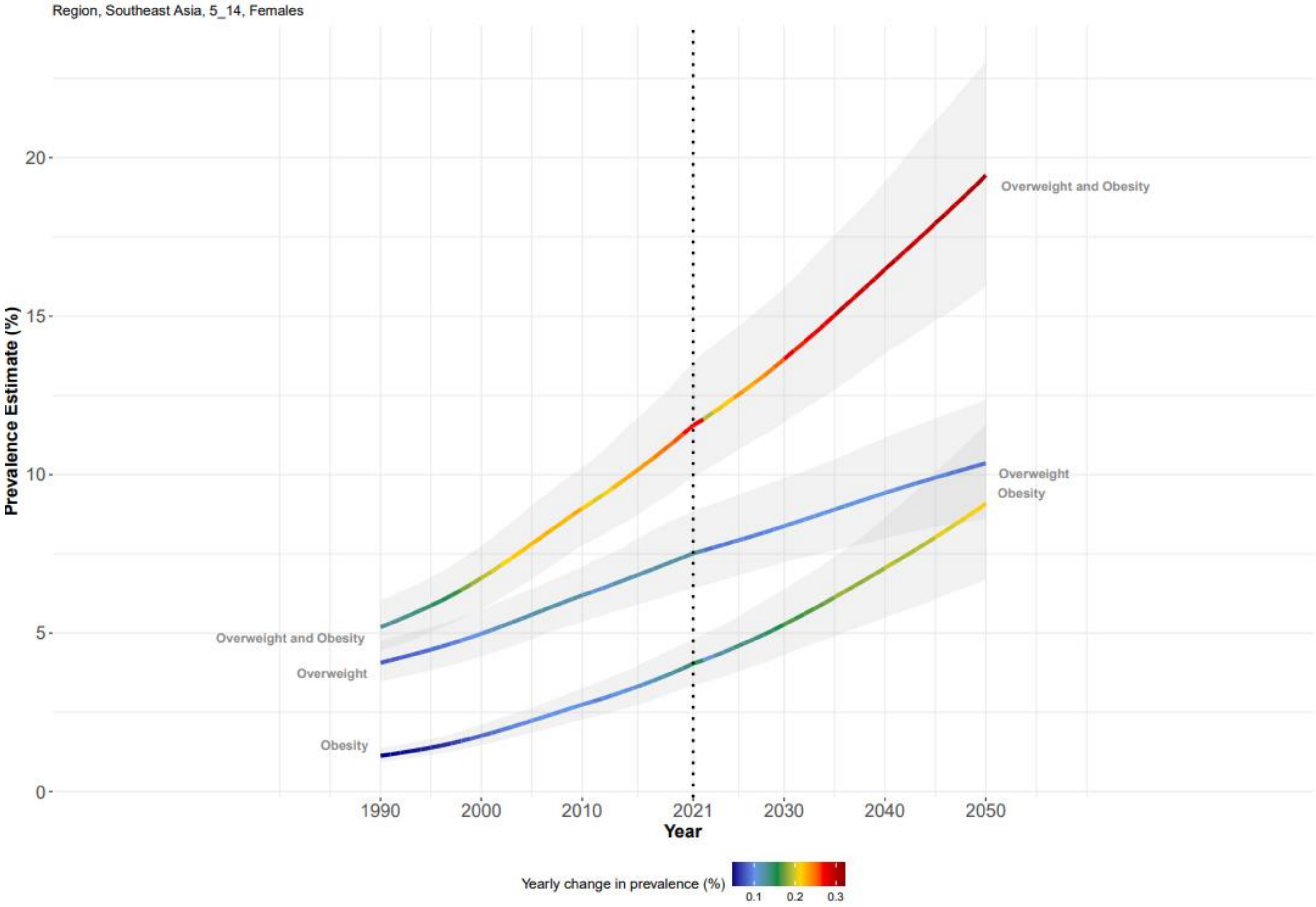
Region, Southeast Asia, 5_14, Females
Prevalence Estimate (%)
0
5
10
15
20
Year
1990
2000
2010
2021
2030
2040
2050
Overweight and Obesity
Overweight
Obesity
Overweight and Obesity
Overweight
Obesity
Yearly change in prevalence (%)
0.1
0.2
0.3

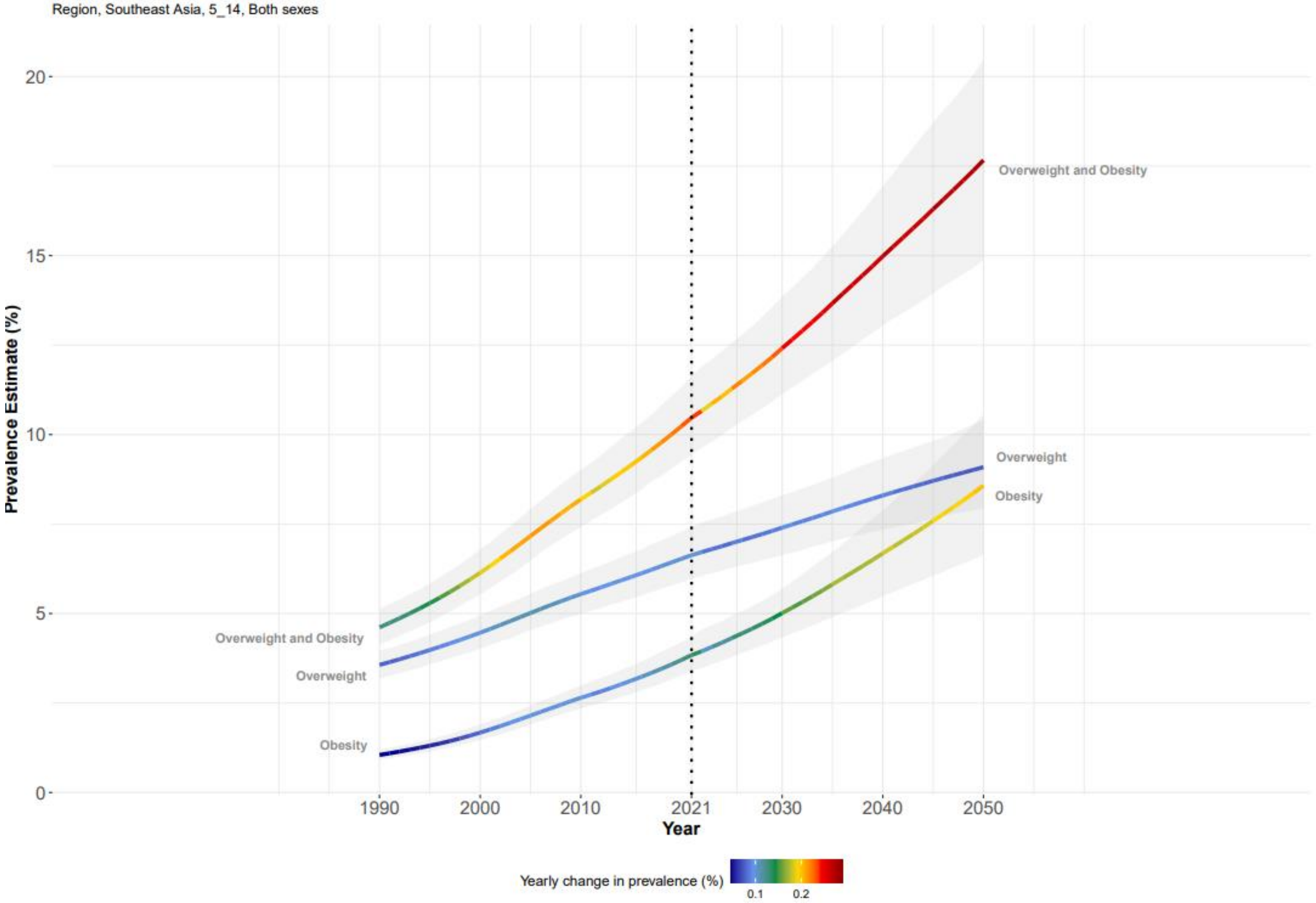
Region, Southeast Asia, 5_14, Both sexes
Prevalence Estimate (%)
20
15
10
5
0
1990
2000
2010
2021
2030
2040
2050
Year
Overweight and Obesity
Overweight
Obesity
Overweight and Obesity
Overweight
Obesity
Yearly change in prevalence (%)
0.1
0.2

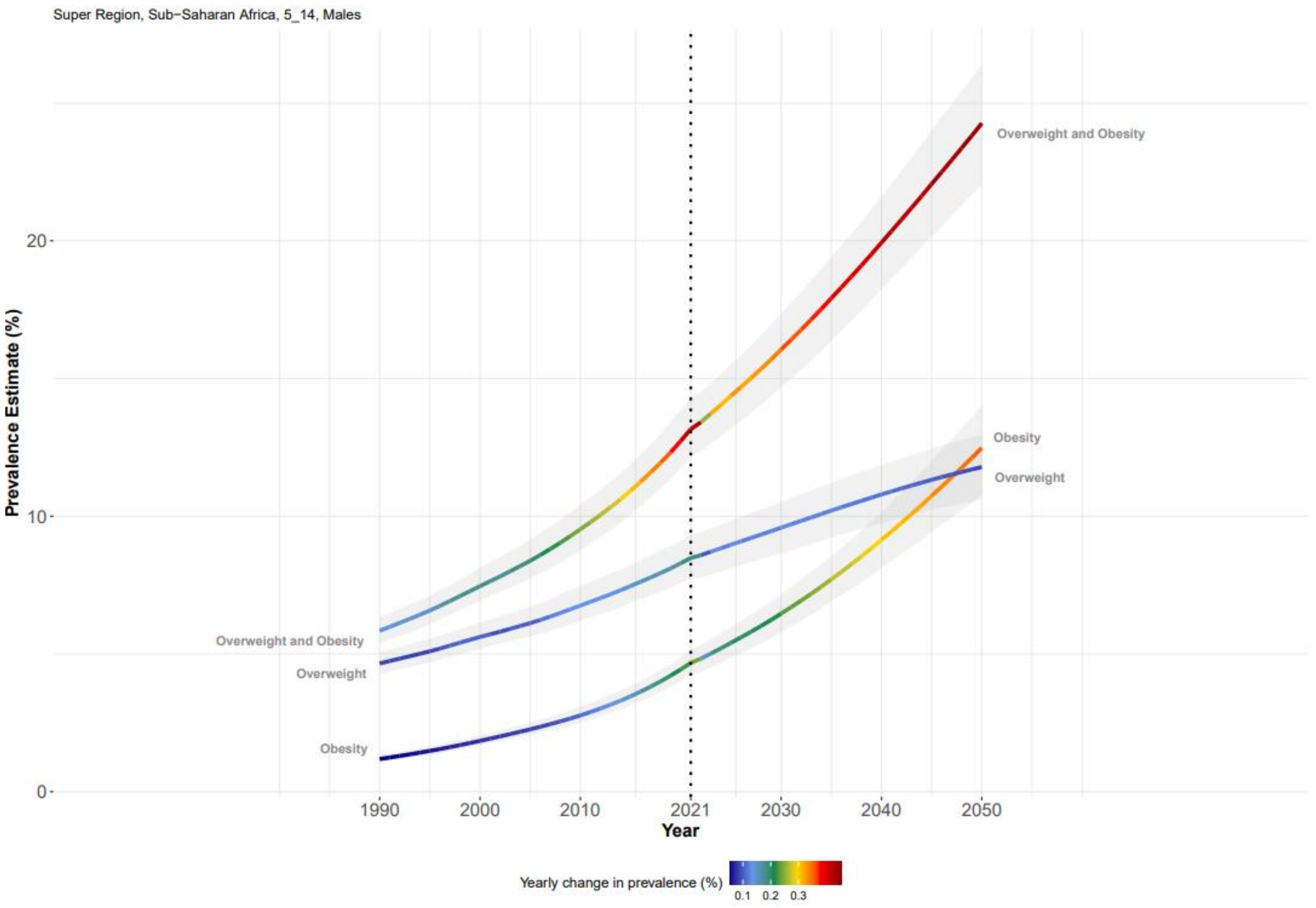

Super Region, Sub−Saharan Africa, 5_14, Males
Prevalence Estimate (%)
20
10
0
1990
2000
2010
2021
2030
2040
2050
Year
Overweight and Obesity
Obesity
Overweight
Overweight and Obesity
Overweight
Obesity
Yearly change in prevalence (%)
0.1
0.2
0.3

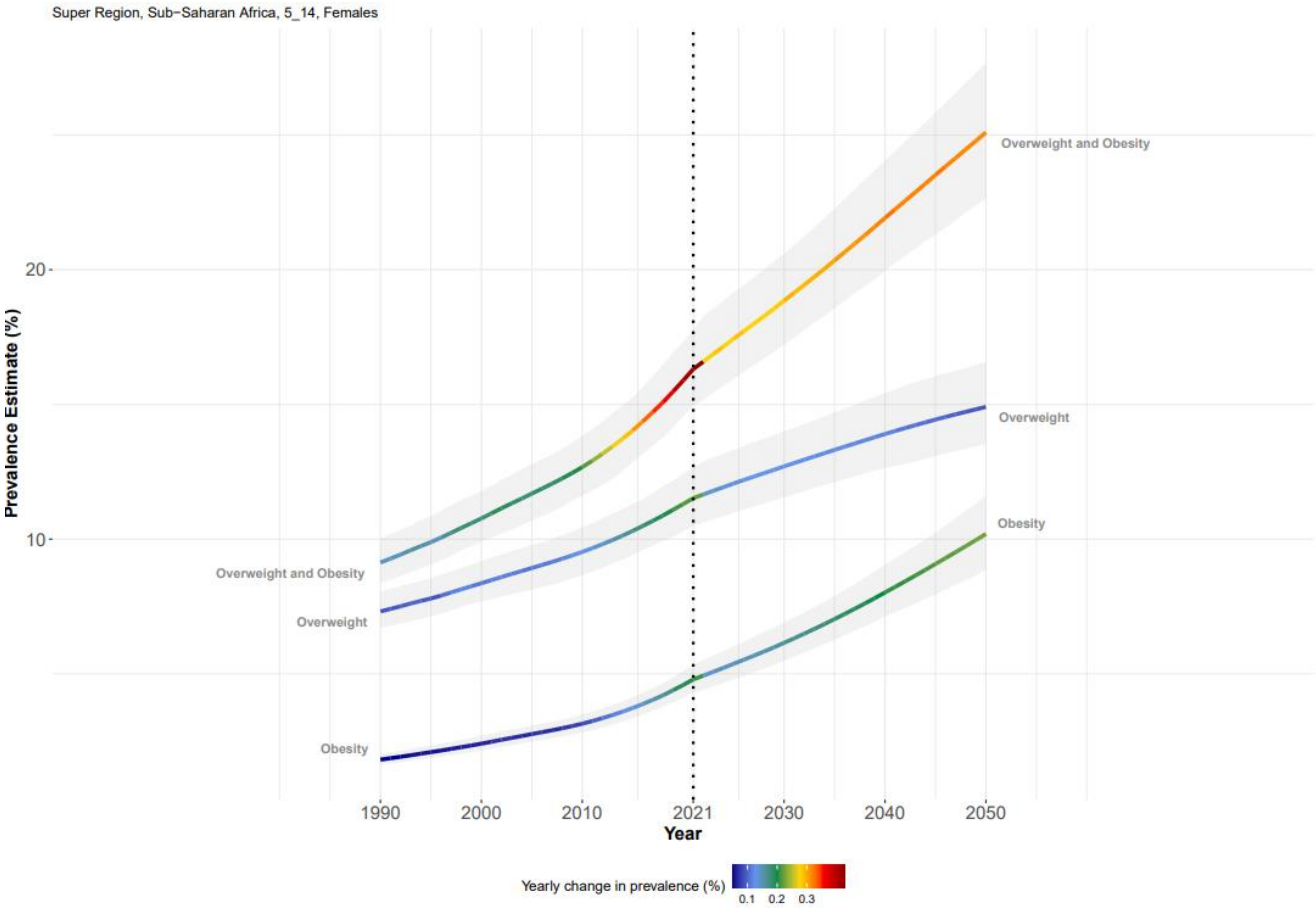

Super Region, Sub-Saharan Africa, 5_14, Females
Prevalence Estimate (%)
20
10
Overweight and Obesity
Overweight
Obesity
Overweight and Obesity
Overweight
Obesity
1990
2000
2010
2021
2030
2040
2050
Year
Yearly change in prevalence (%)
0.1
0.2
0.3

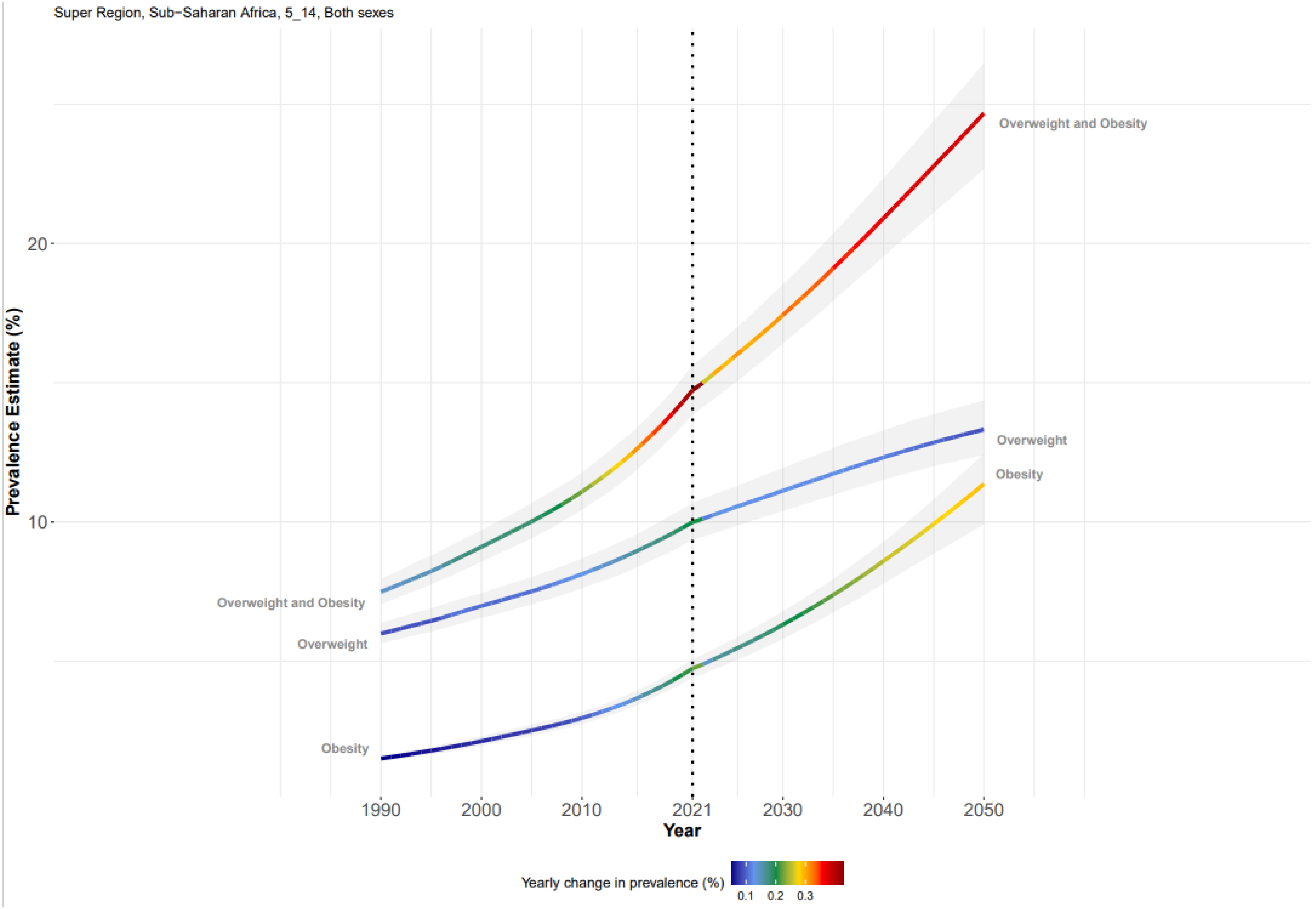
Super Region, Sub−Saharan Africa, 5_14, Both sexes
Prevalence Estimate (%)
20
10
Overweight and Obesity
Overweight
Obesity
Overweight and Obesity
Overweight
Obesity
1990
2000
2010
2021
2030
2040
2050
Year
Yearly change in prevalence (%)
0.1
0.2
0.3

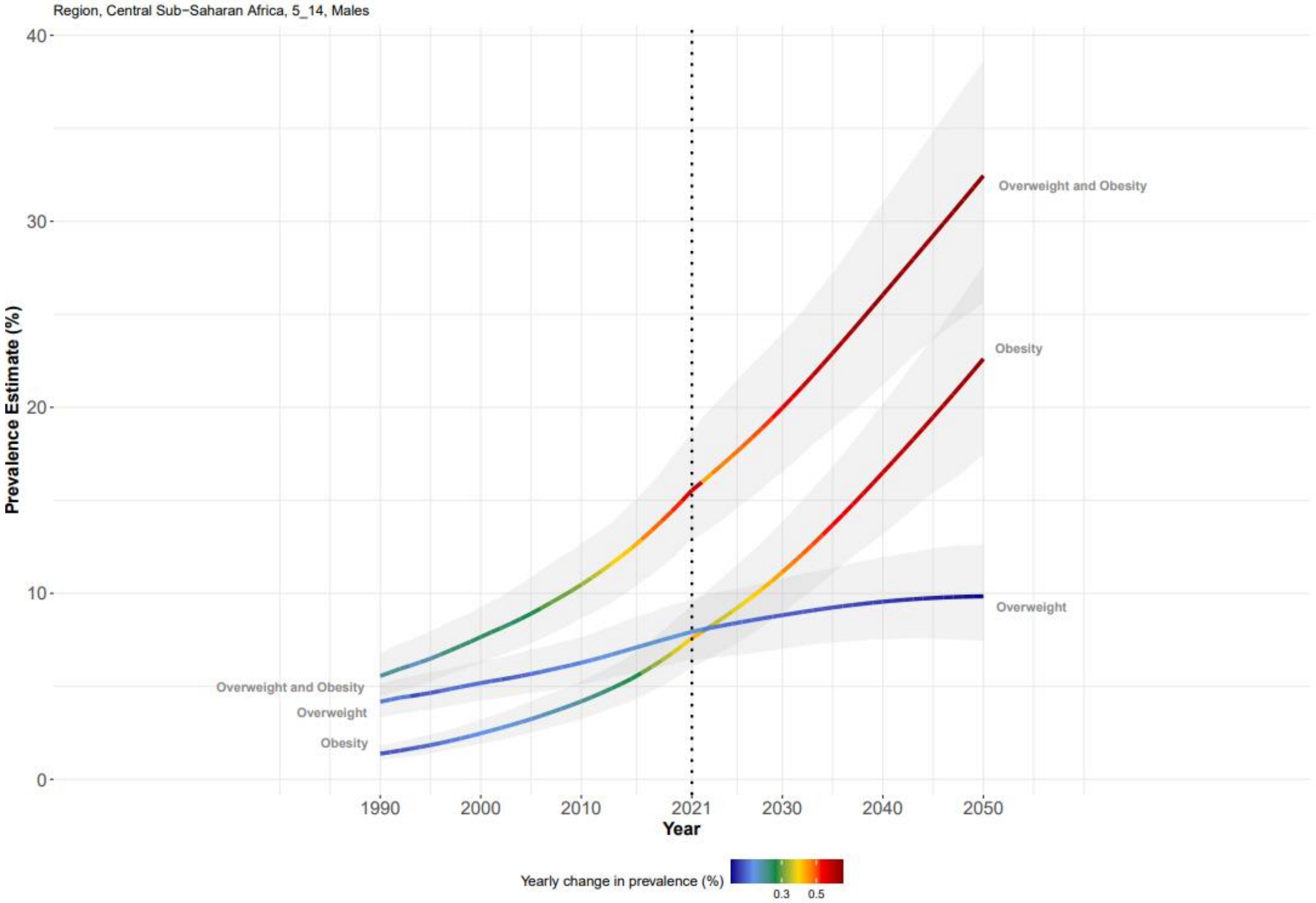

Region, Central Sub−Saharan Africa, 5_14, Males
Prevalence Estimate (%)
40
30
20
10
0
1990
2000
2010
2021
2030
2040
2050
Year
Overweight and Obesity
Obesity
Overweight
Overweight and Obesity
Overweight
Obesity
Yearly change in prevalence (%)
0.3
0.5

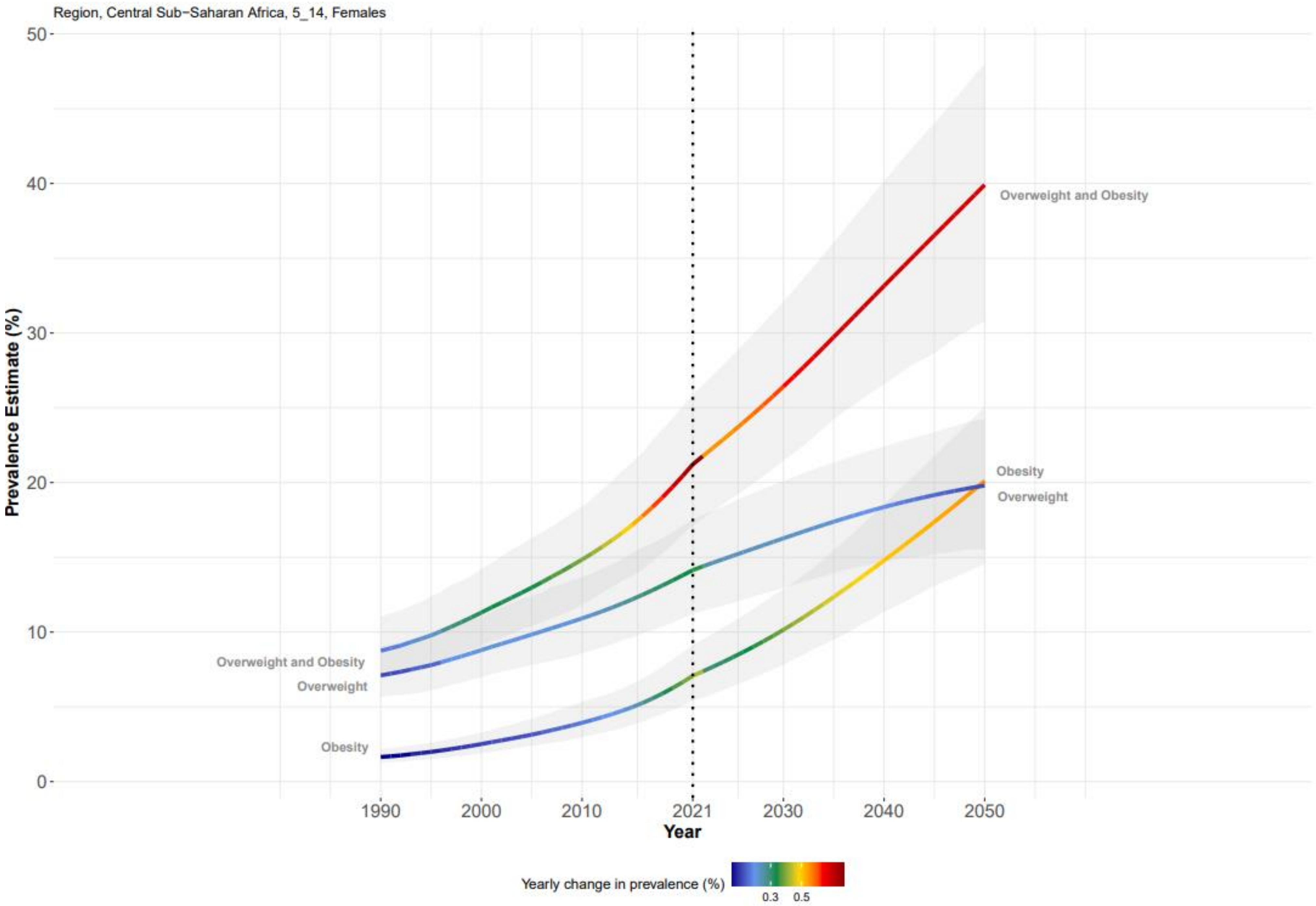
Region, Central Sub−Saharan Africa, 5_14, Females
Prevalence Estimate (%)
0
10
20
30
40
50
1990
2000
2010
2021
2030
2040
2050
Year
Overweight and Obesity
Overweight
Obesity
Overweight and Obesity
Obesity
Overweight
Yearly change in prevalence (%)
0.3
0.5

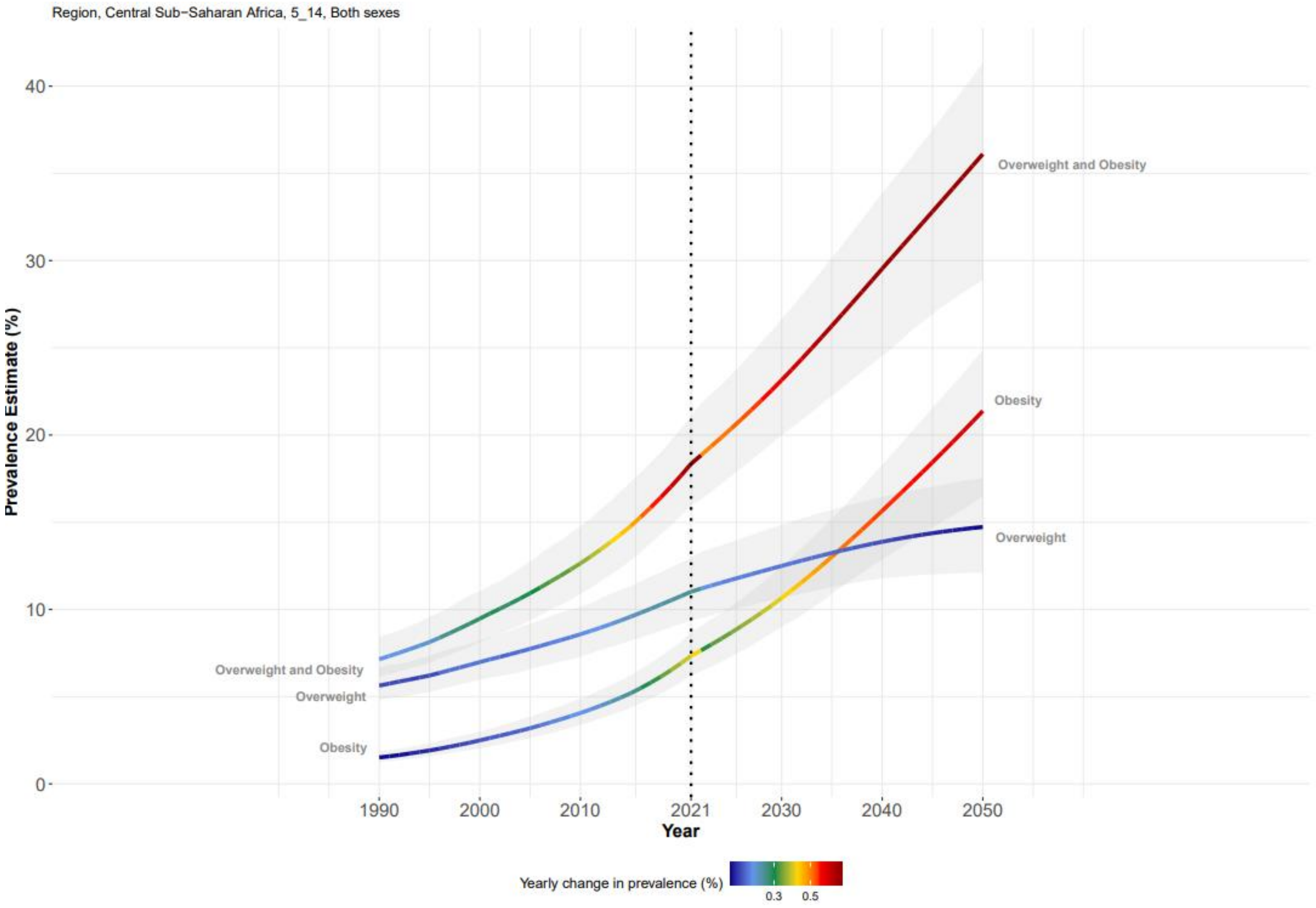

Region, Central Sub−Saharan Africa, 5_14, Both sexes
Prevalence Estimate (%)
40
30
20
10
0
1990
2000
2010
2021
2030
2040
2050
Year
Overweight and Obesity
Obesity
Overweight
Overweight and Obesity
Overweight
Obesity
Yearly change in prevalence (%)
0.3
0.5

Region, Eastern Sub−Saharan Africa, 5_14, Males

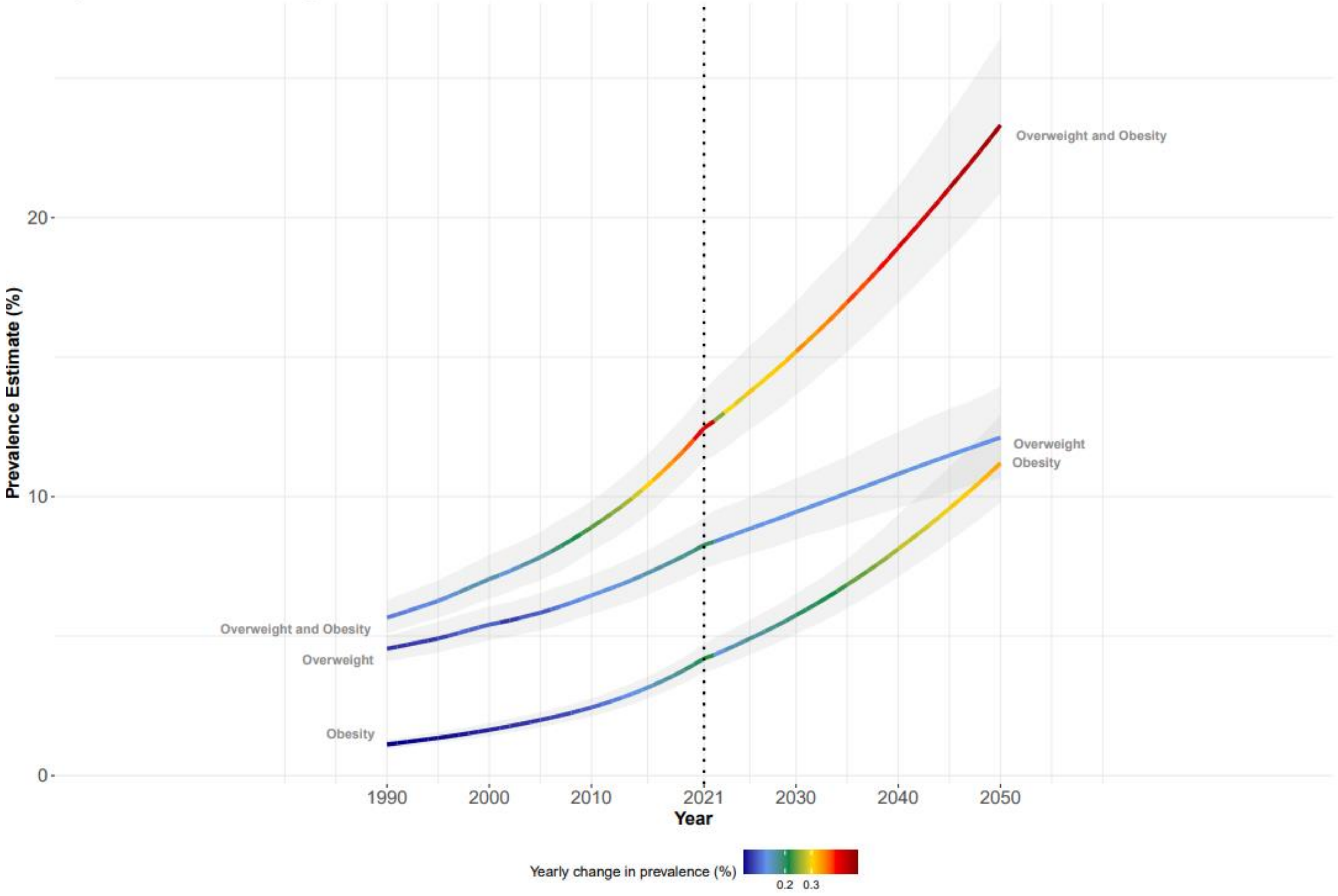

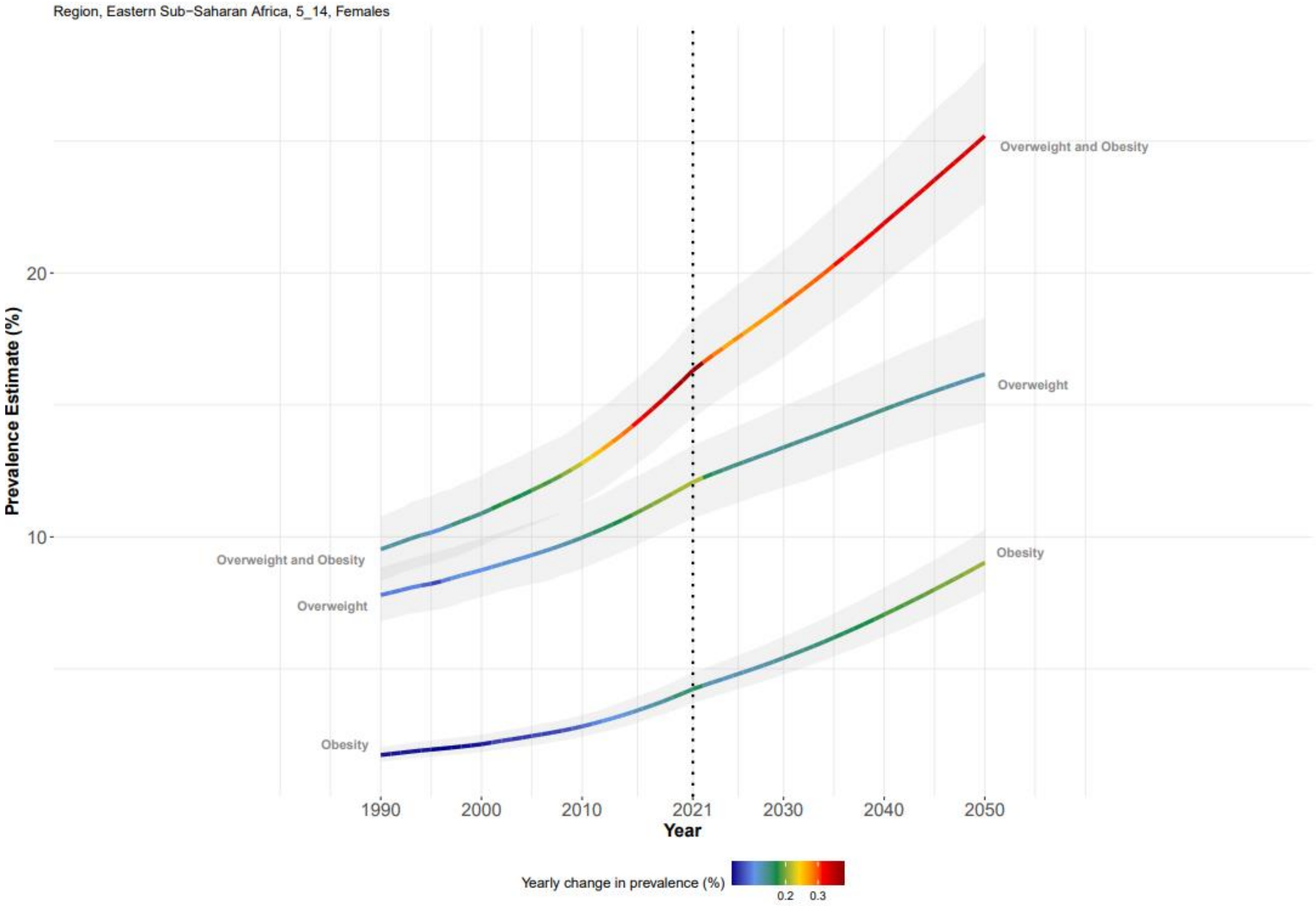

Region, Eastern Sub−Saharan Africa, 5_14, Females
Prevalence Estimate (%)
20
10
Overweight and Obesity
Overweight
Obesity
Overweight and Obesity
Overweight
Obesity
1990
2000
2010
2021
2030
2040
2050
Year
Yearly change in prevalence (%)
0.2
0.3

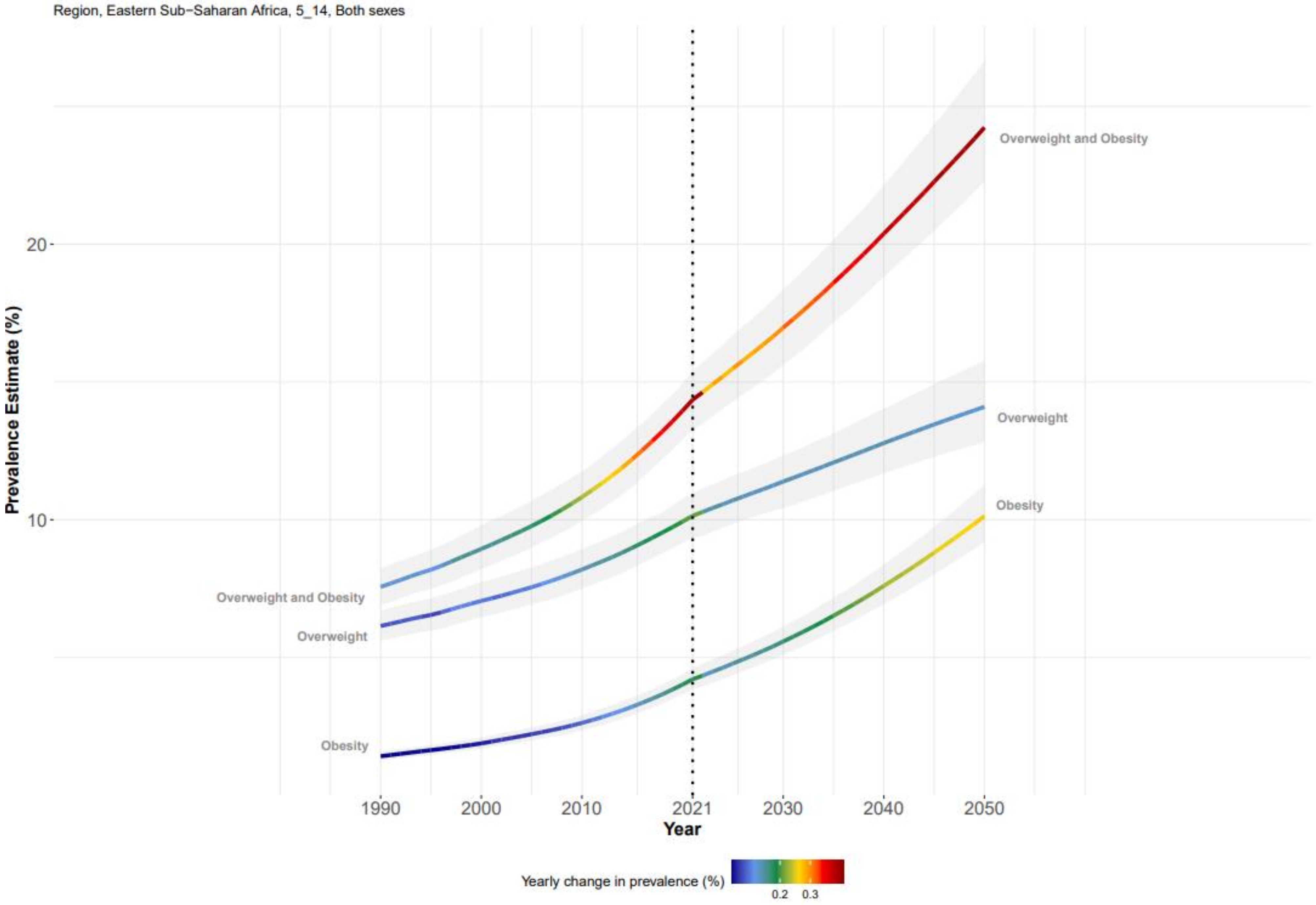
Region, Eastern Sub−Saharan Africa, 5_14, Both sexes
Prevalence Estimate (%)
20
10
Overweight and Obesity
Overweight
Obesity
1990
2000
2010
2021
2030
2040
2050
Year
Yearly change in prevalence (%)
0.2
0.3

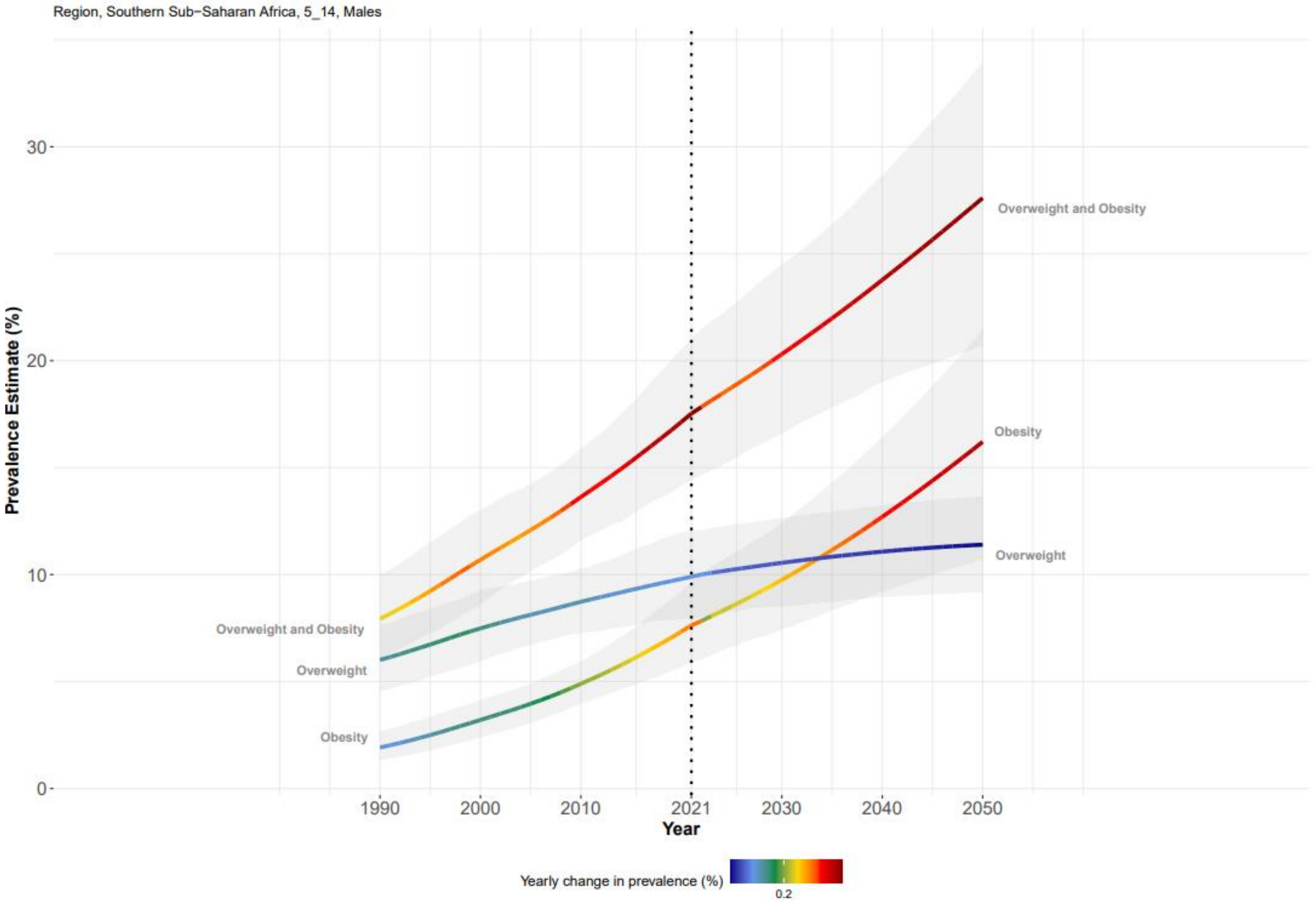
Region, Southern Sub−Saharan Africa, 5_14, Males
Prevalence Estimate (%)
30
20
10
0
1990
2000
2010
2021
2030
2040
2050
Year
Overweight and Obesity
Obesity
Overweight
Overweight and Obesity
Overweight
Obesity
Yearly change in prevalence (%)
0.2

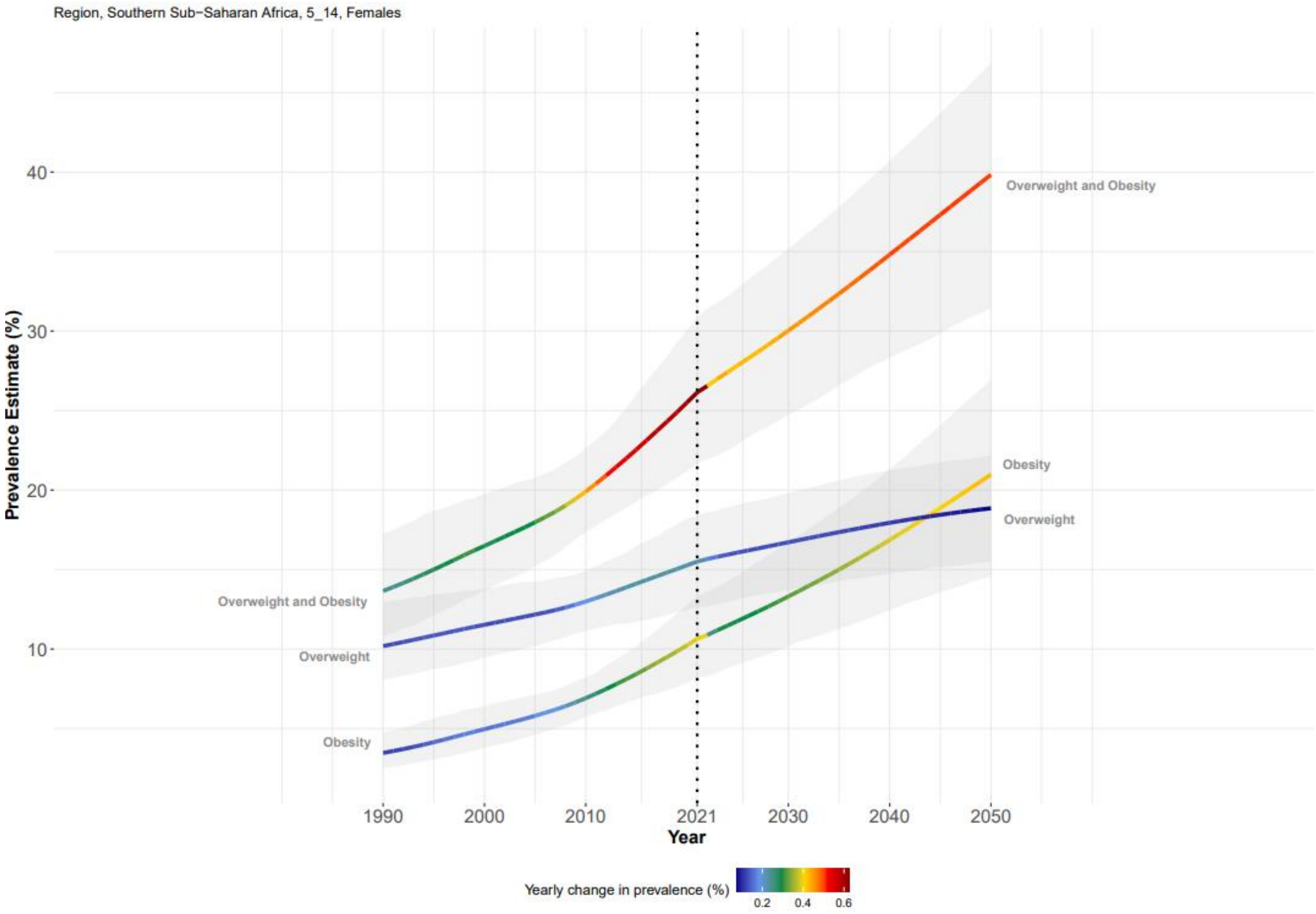
Region, Southern Sub−Saharan Africa, 5_14, Females
Prevalence Estimate (%)
40
30
20
10
Overweight and Obesity
Obesity
Overweight
Overweight and Obesity
Overweight
Obesity
1990
2000
2010
2021
2030
2040
2050
Year
Yearly change in prevalence (%)
0.2
0.4
0.6

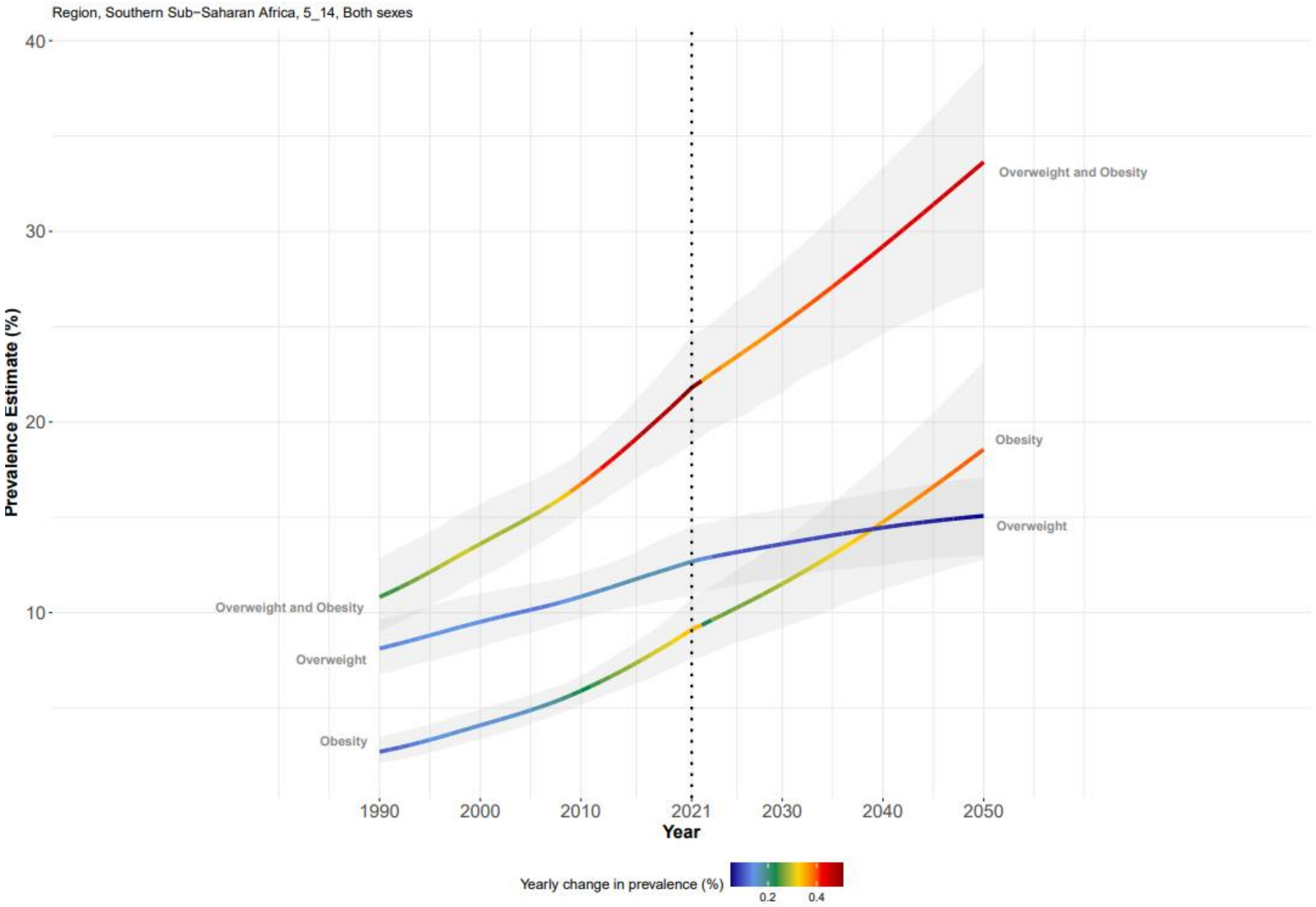
Region, Southern Sub−Saharan Africa, 5_14, Both sexes
Prevalence Estimate (%)
40
30
20
10
1990
2000
2010
2021
2030
2040
2050
Year
Overweight and Obesity
Obesity
Overweight
Overweight and Obesity
Overweight
Obesity
Yearly change in prevalence (%)
0.2
0.4

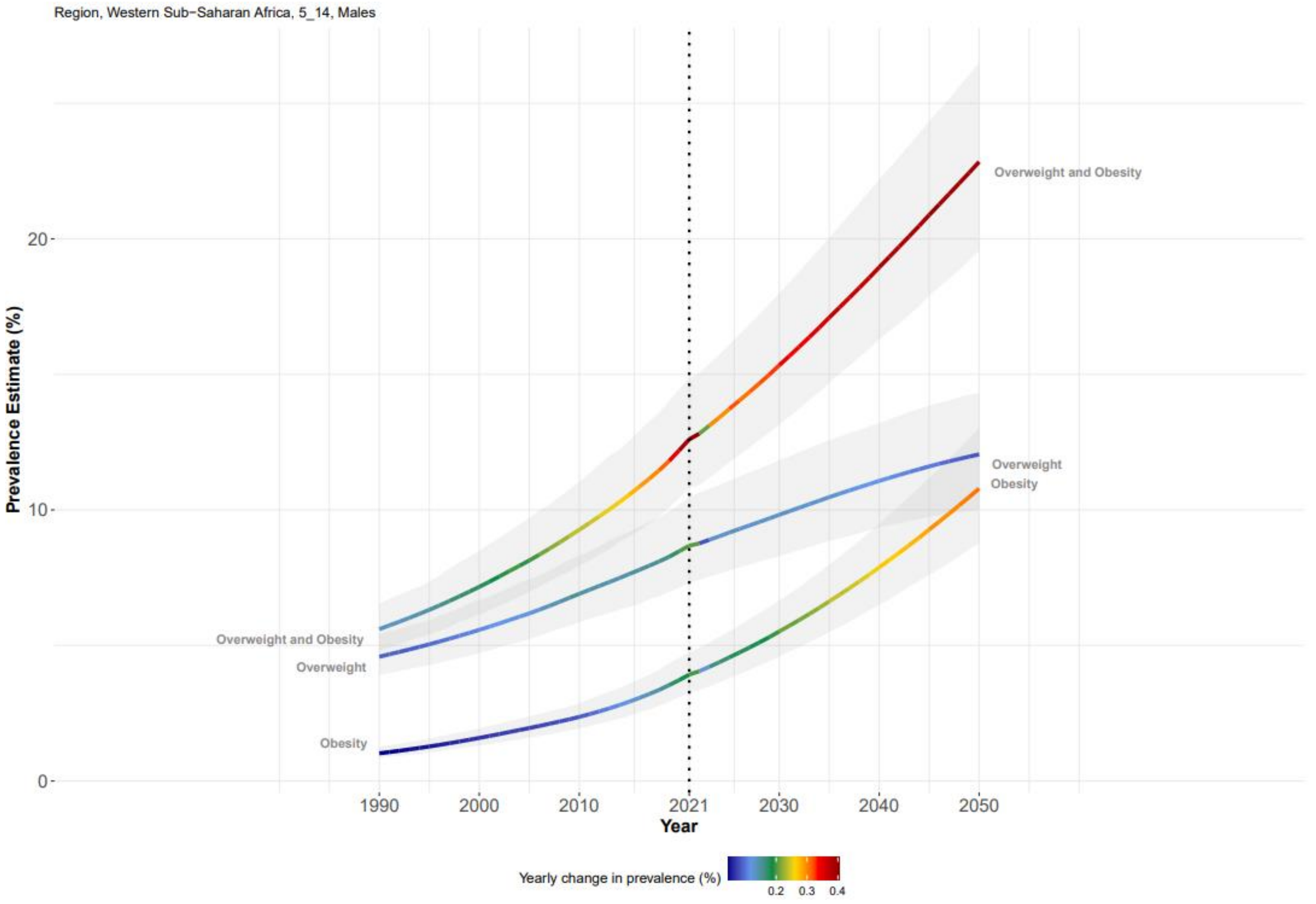

Region, Western Sub−Saharan Africa, 5_14, Males
Prevalence Estimate (%)
20
10
0
1990
2000
2010
2021
2030
2040
2050
Year
Overweight and Obesity
Overweight
Obesity
Overweight and Obesity
Overweight
Obesity
Yearly change in prevalence (%)
0.2
0.3
0.4

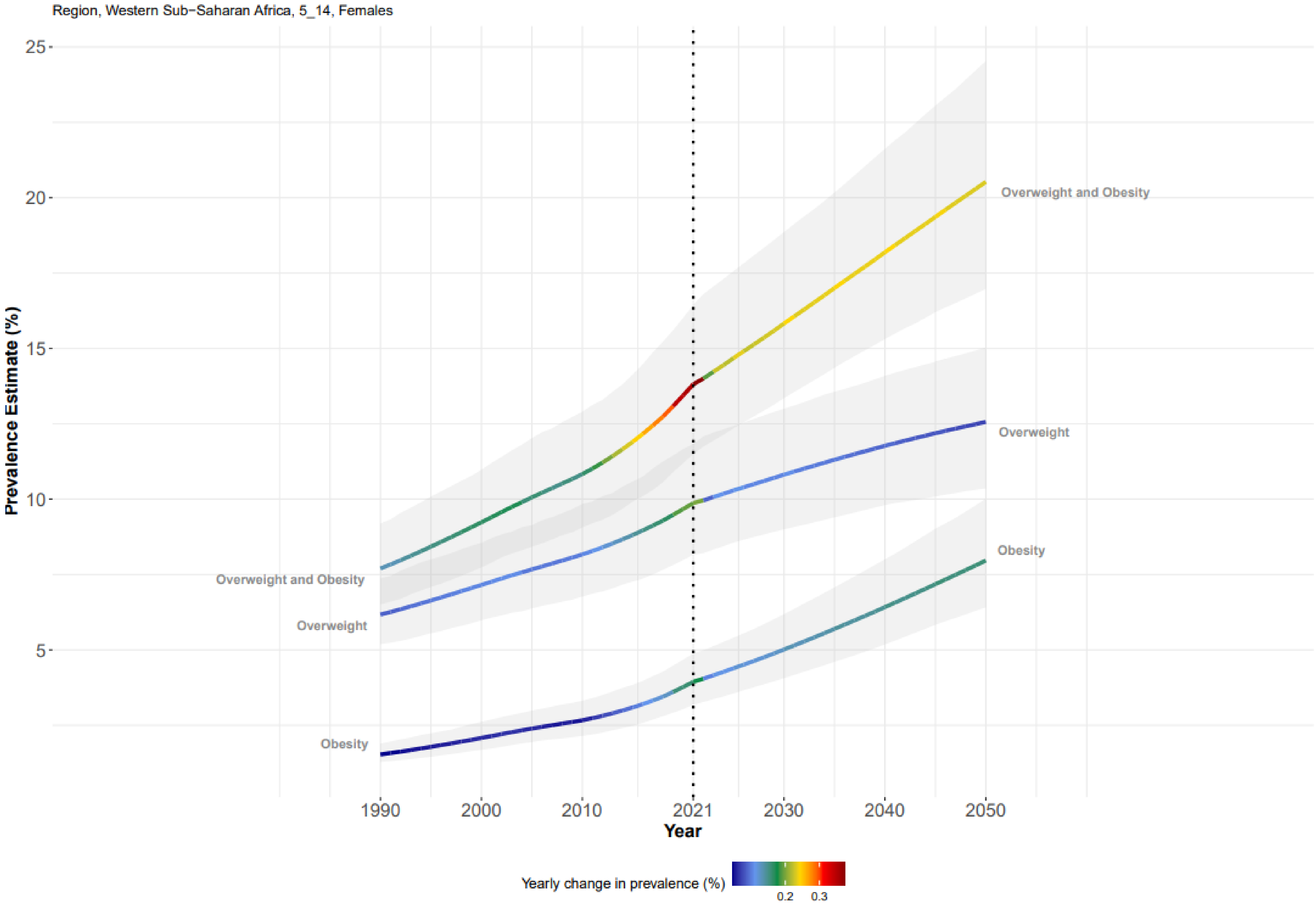

Region, Western Sub−Saharan Africa, 5_14, Females
Prevalence Estimate (%)
25
20
15
10
5
1990
2000
2010
2021
2030
2040
2050
Year
Overweight and Obesity
Overweight
Obesity
Overweight and Obesity
Overweight
Obesity
Yearly change in prevalence (%)
0.2
0.3

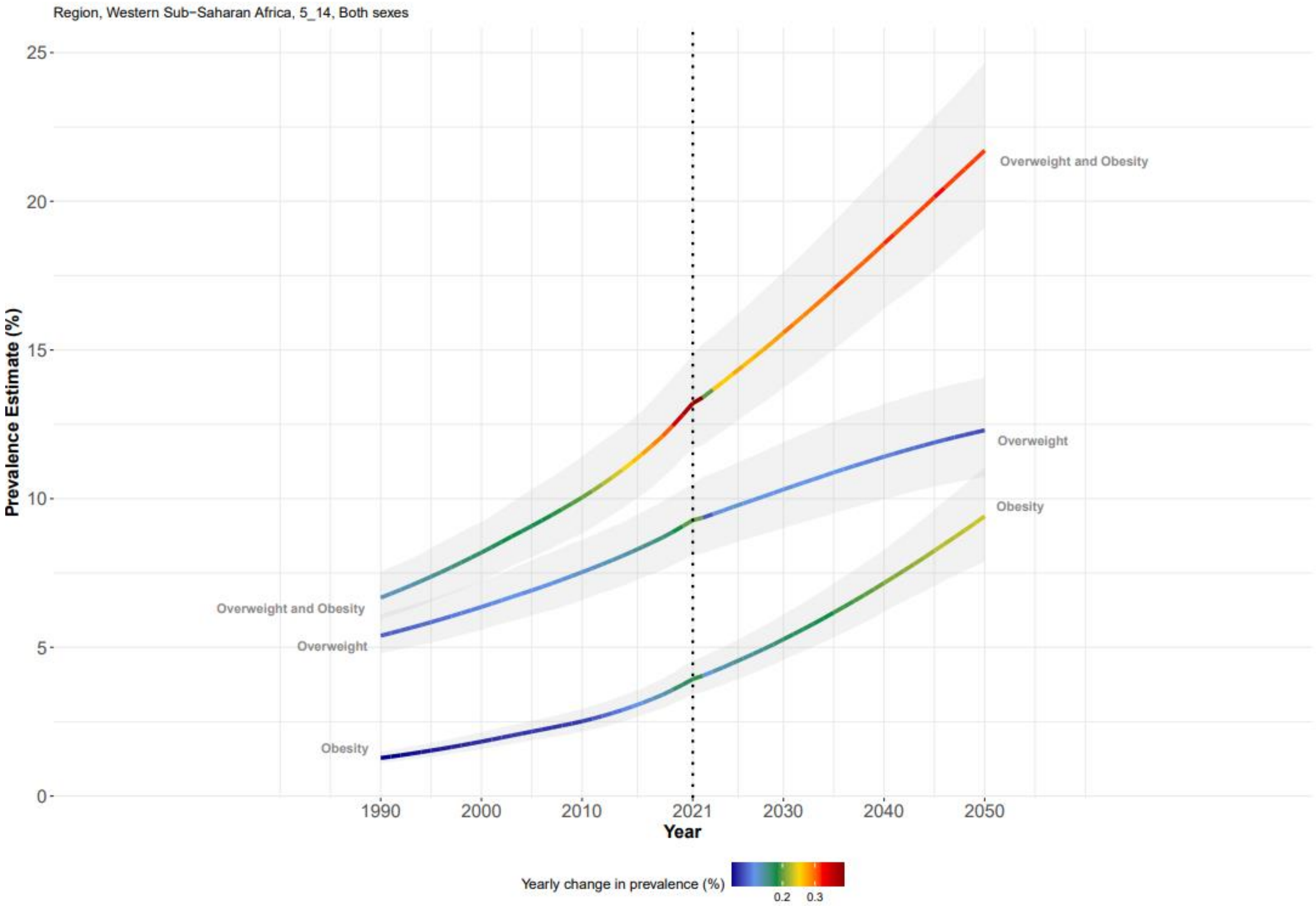

Region, Western Sub−Saharan Africa, 5_14, Both sexes
Prevalence Estimate (%)
25
20
15
10
5
0
1990
2000
2010
2021
2030
2040
2050
Year
Overweight and Obesity
Overweight
Obesity
Overweight and Obesity
Overweight
Obesity
Yearly change in prevalence (%)
0.2
0.3

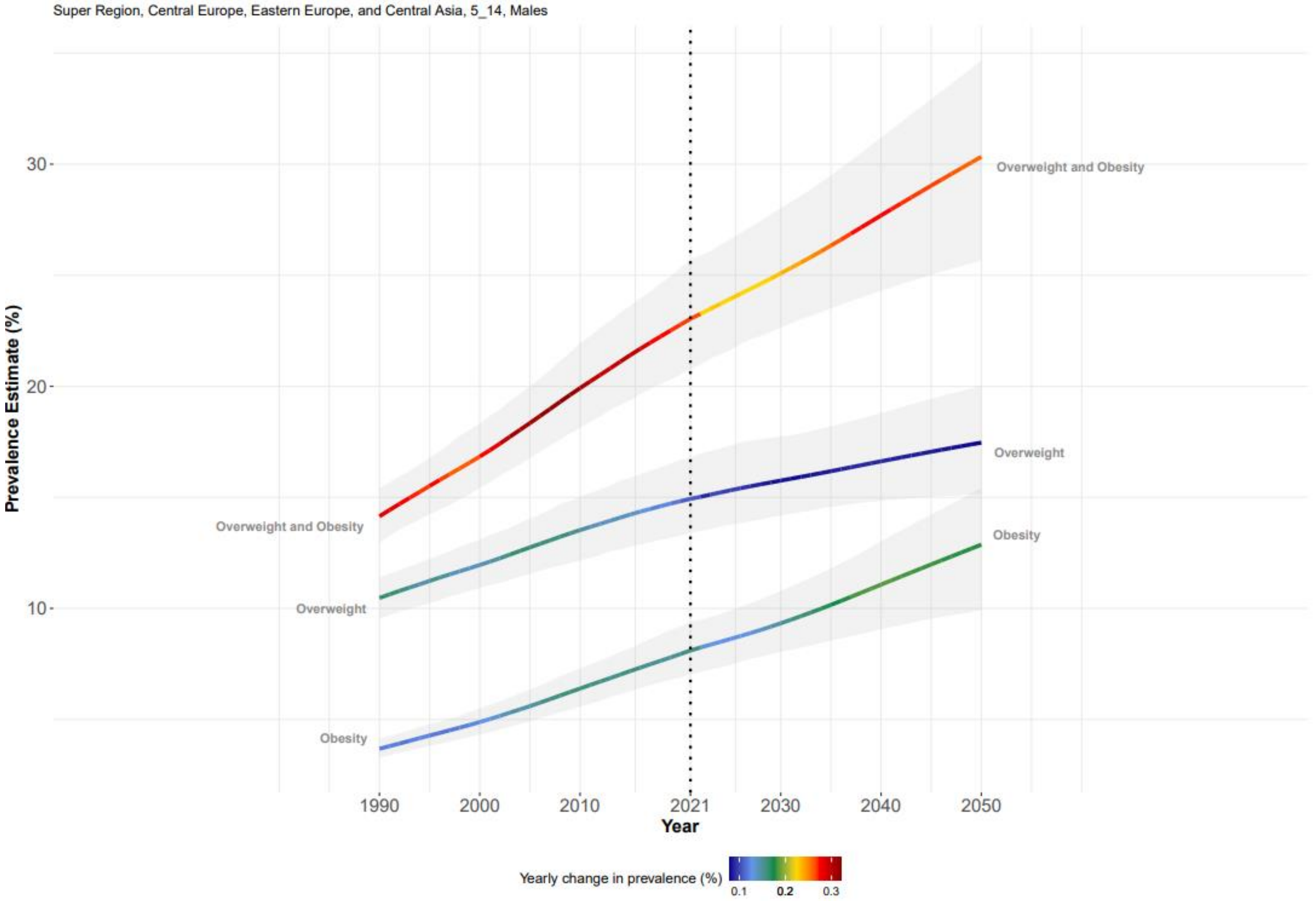

Super Region, Central Europe, Eastern Europe, and Central Asia, 5_14, Males
Prevalence Estimate (%)
30
20
10
Overweight and Obesity
Overweight
Obesity
Overweight and Obesity
Overweight
Obesity
1990
2000
2010
2021
2030
2040
2050
Year
Yearly change in prevalence (%)
0.1
0.2
0.3

Super Region, Central Europe, Eastern Europe, and Central Asia, 5_14, Females

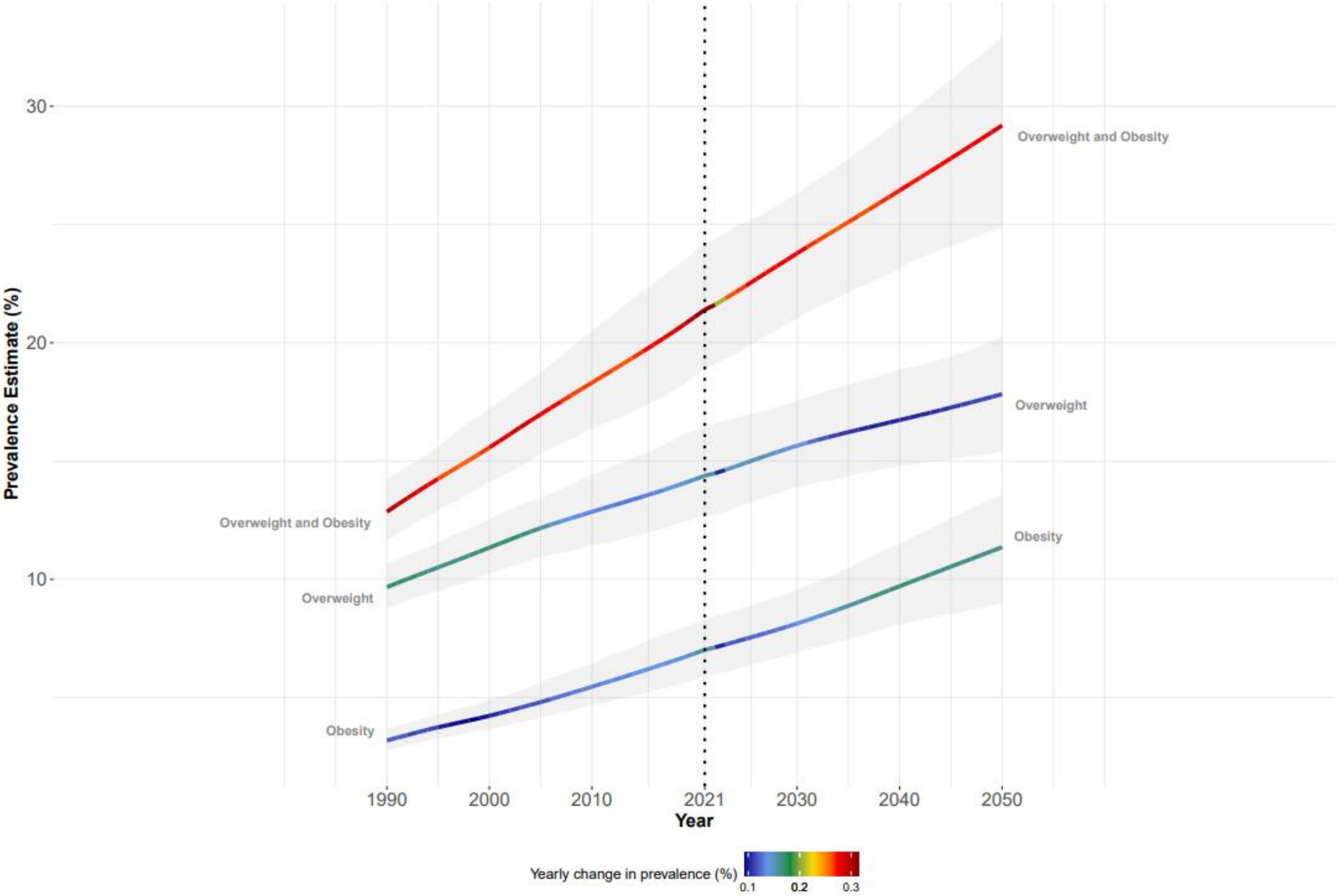

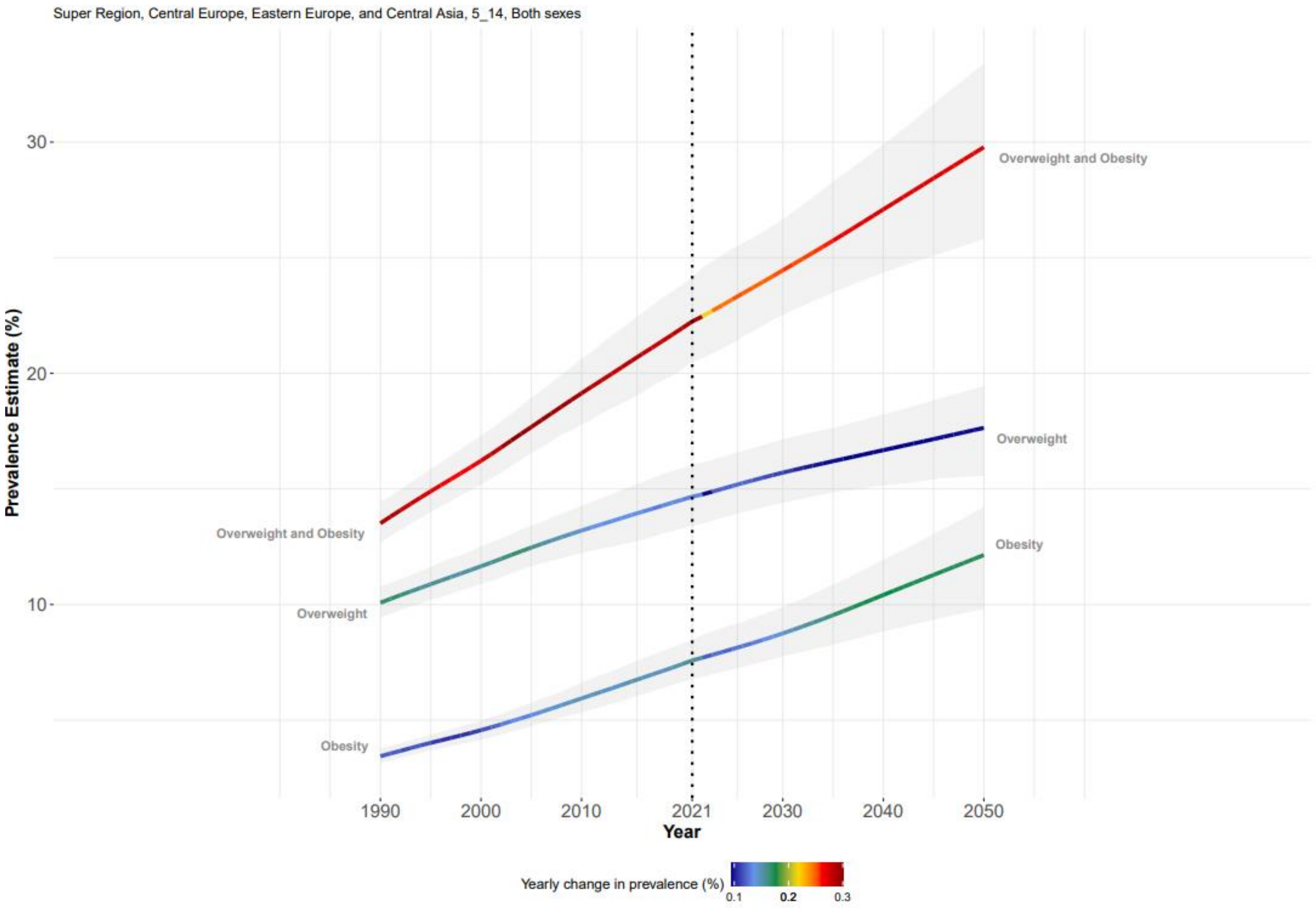

Super Region, Central Europe, Eastern Europe, and Central Asia, 5_14, Both sexes
Prevalence Estimate (%)
30
20
10
Overweight and Obesity
Overweight
Obesity
Overweight and Obesity
Overweight
Obesity
1990
2000
2010
2021
2030
2040
2050
Year
Yearly change in prevalence (%)
0.1
0.2
0.3

Region, Central Asia, 5_14, Males

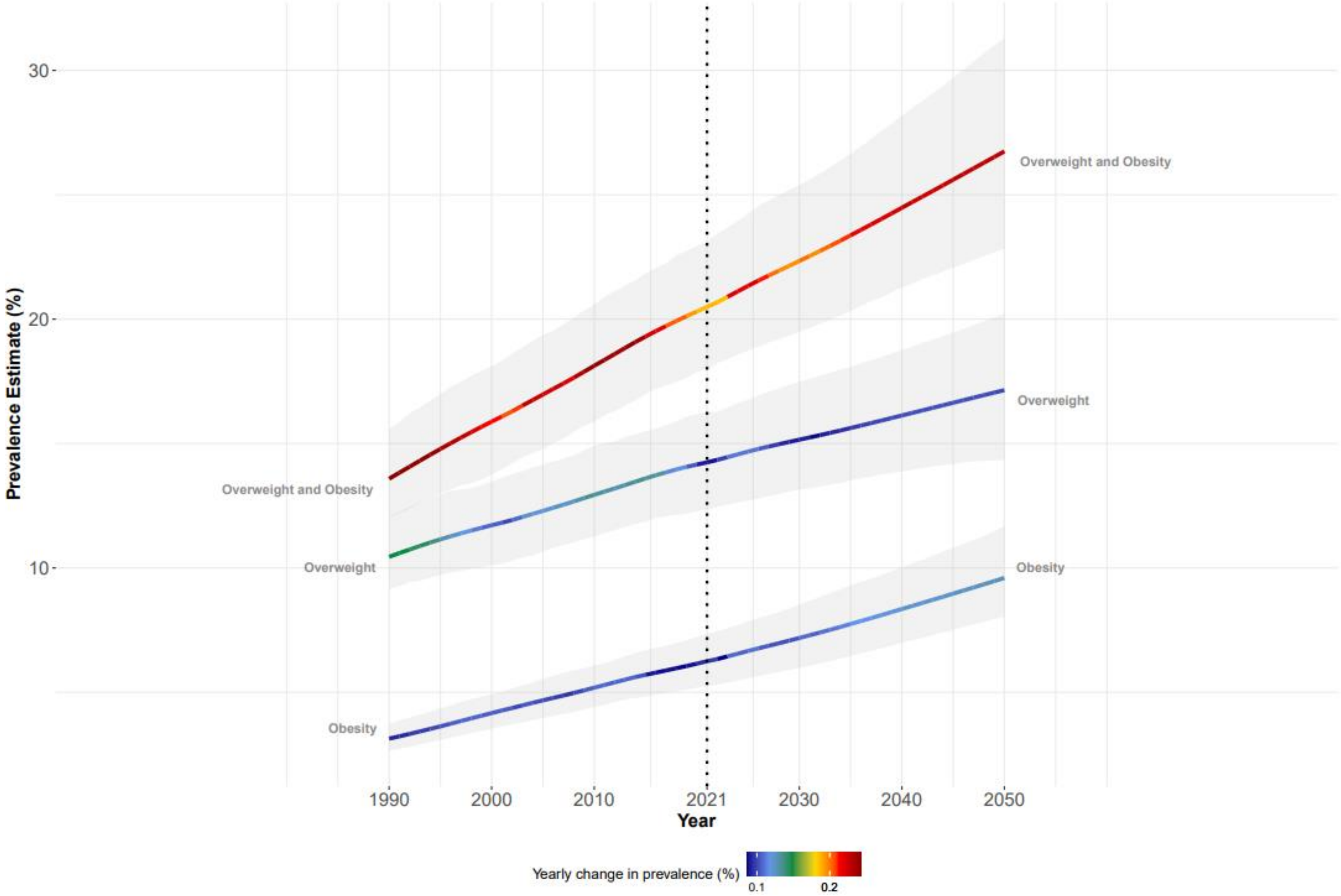

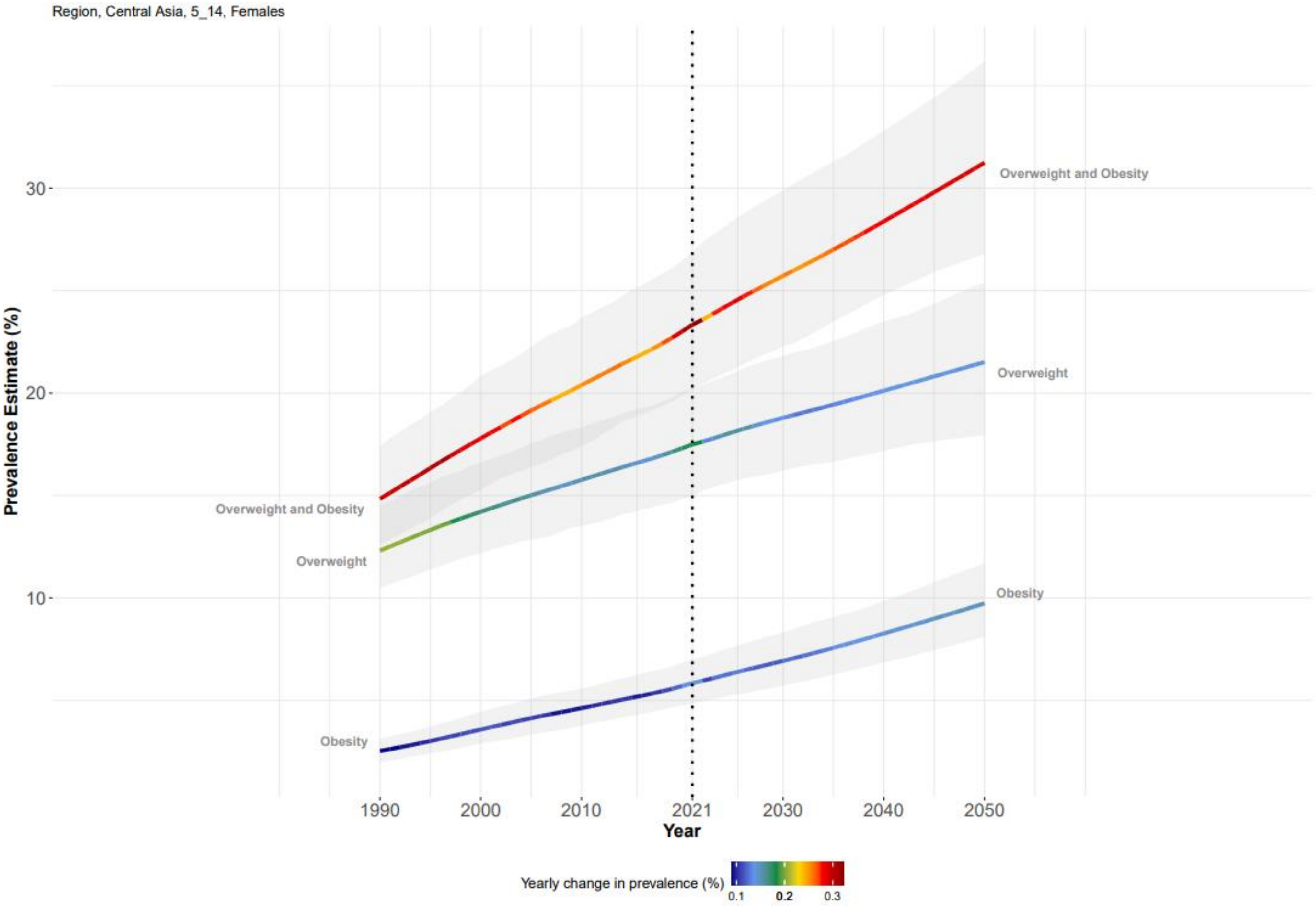

Region, Central Asia, 5_14, Females
Prevalence Estimate (%)
30
20
10
Overweight and Obesity
Overweight
Obesity
Overweight and Obesity
Overweight
Obesity
1990
2000
2010
2021
2030
2040
2050
Year
Yearly change in prevalence (%)
0.1
0.2
0.3

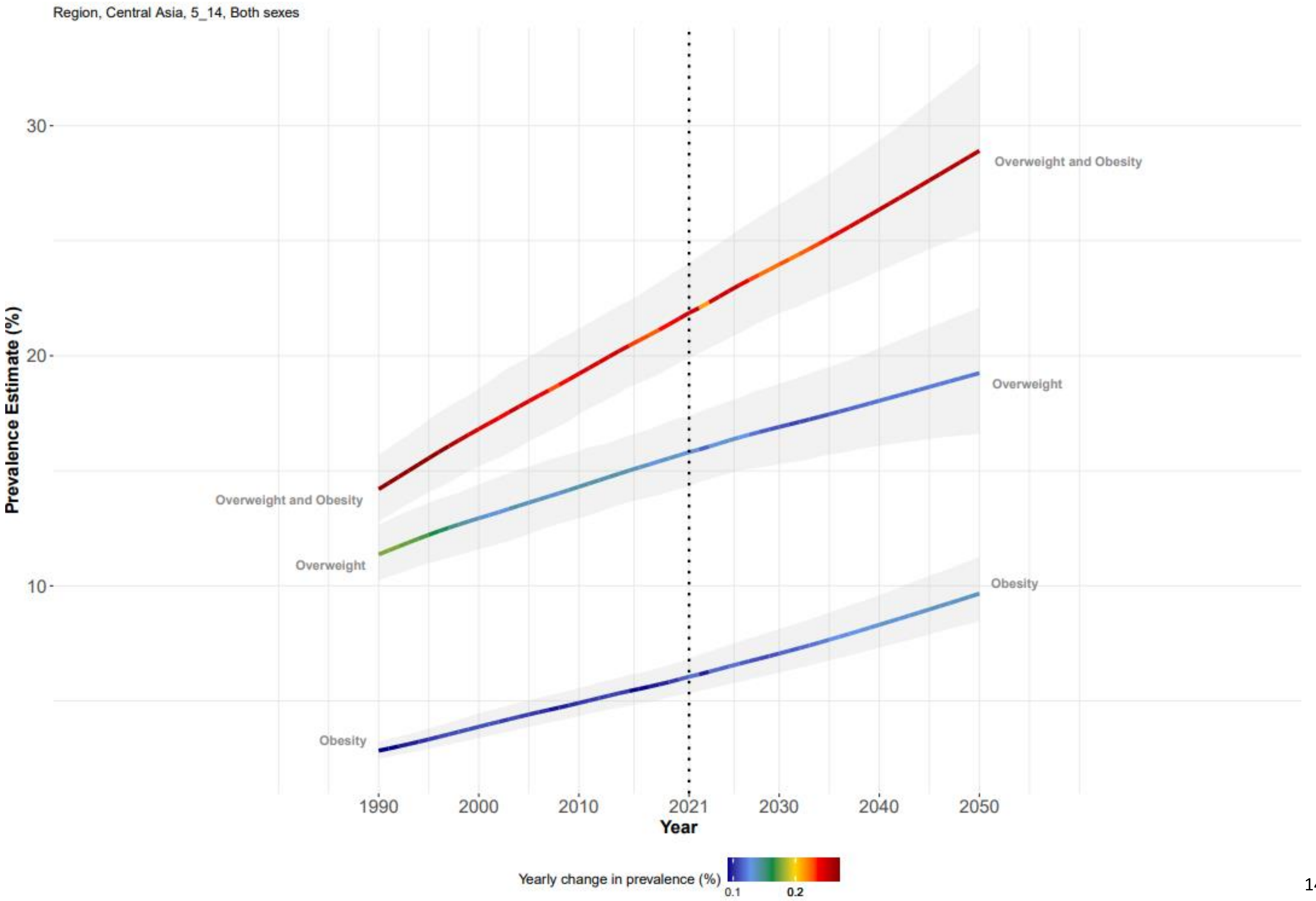

Region, Central Asia, 5_14, Both sexes
Prevalence Estimate (%)
30
20
10
Overweight and Obesity
Overweight
Obesity
Overweight and Obesity
Overweight
Obesity
1990
2000
2010
2021
2030
2040
2050
Year
Yearly change in prevalence (%)
0.1
0.2

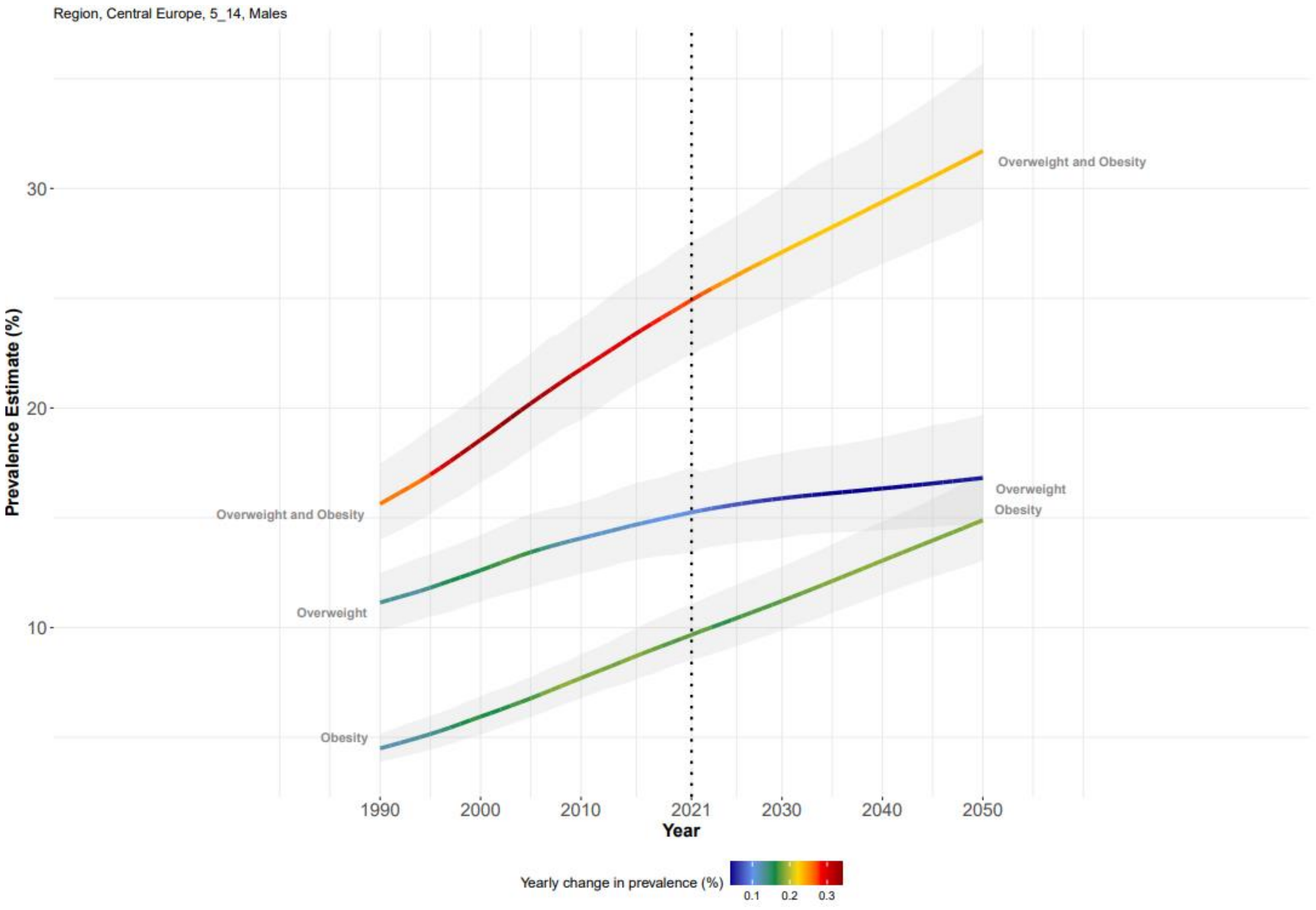
Region, Central Europe, 5_14, Males
Prevalence Estimate (%)
30
20
10
Overweight and Obesity
Overweight
Obesity
Overweight and Obesity
Overweight
Obesity
1990
2000
2010
2021
2030
2040
2050
Year
Yearly change in prevalence (%)
0.1
0.2
0.3

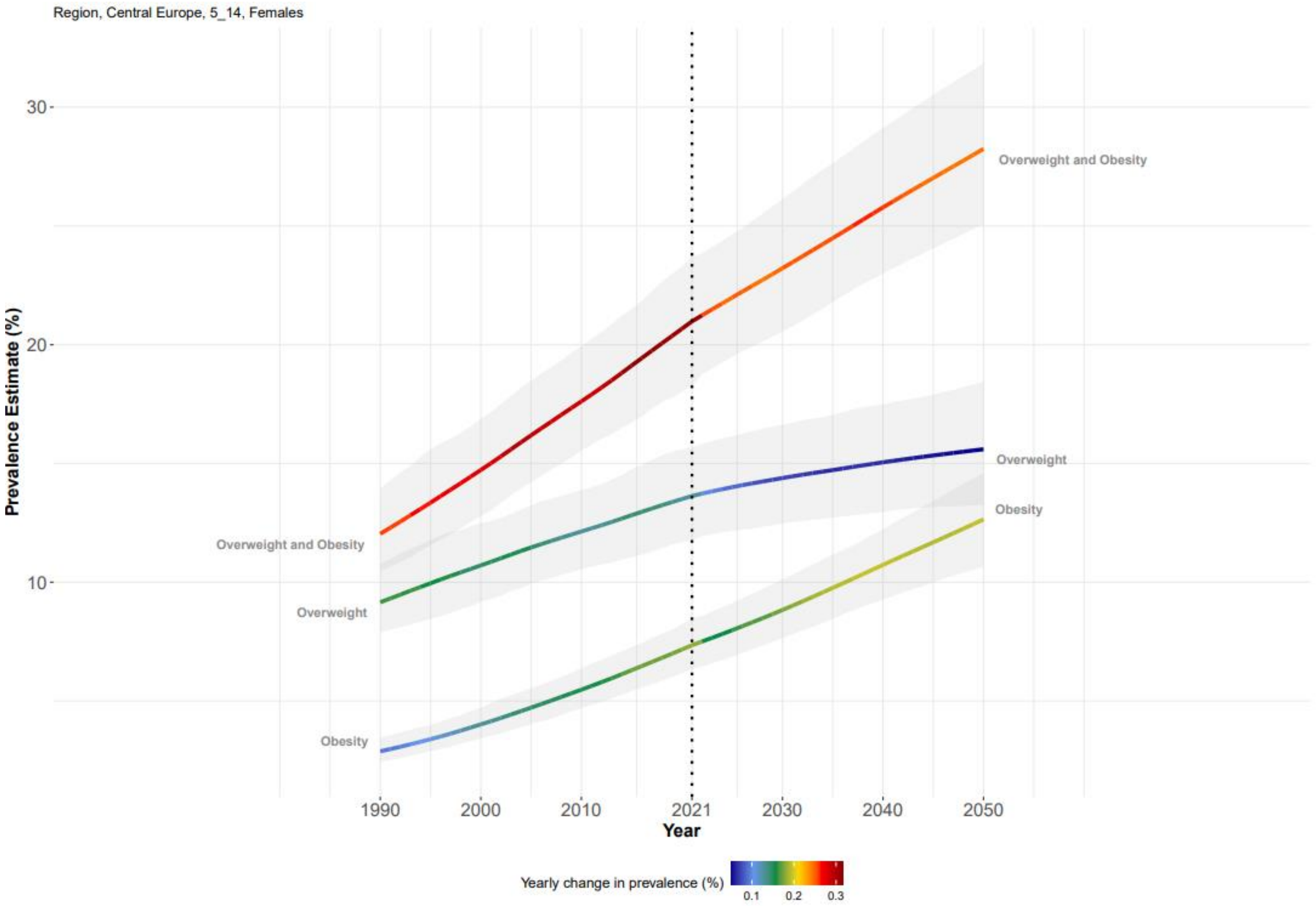

Region, Central Europe, 5_14, Females
Prevalence Estimate (%)
30
20
10
Overweight and Obesity
Overweight
Obesity
Overweight and Obesity
Overweight
Obesity
1990
2000
2010
2021
2030
2040
2050
Year
Yearly change in prevalence (%)
0.1
0.2
0.3

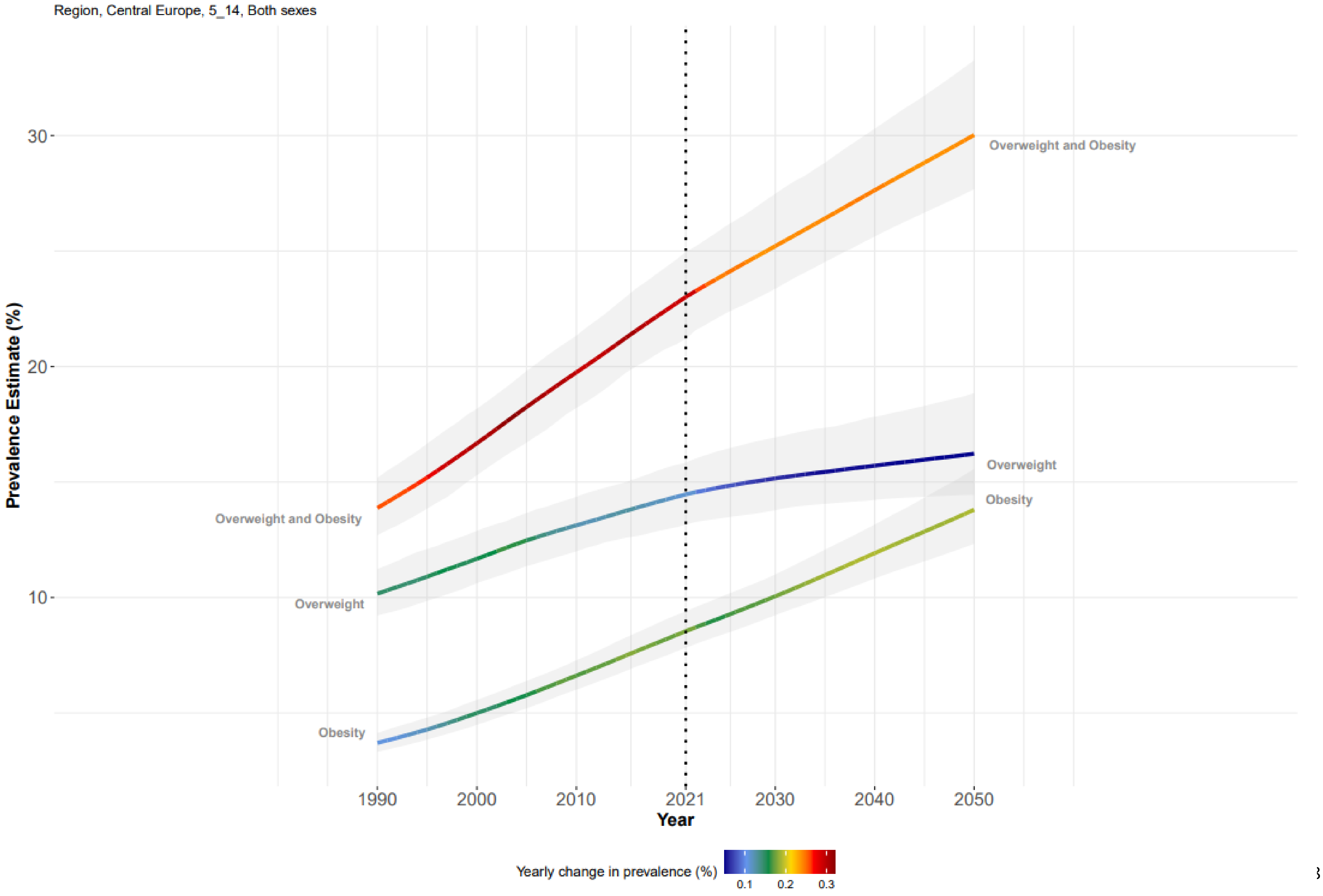
Region, Central Europe, 5_14, Both sexes
Prevalence Estimate (%)
30
20
10
Overweight and Obesity
Overweight
Obesity
Overweight and Obesity
Overweight
Obesity
1990
2000
2010
2021
2030
2040
2050
Year
Yearly change in prevalence (%)
0.1
0.2
0.3

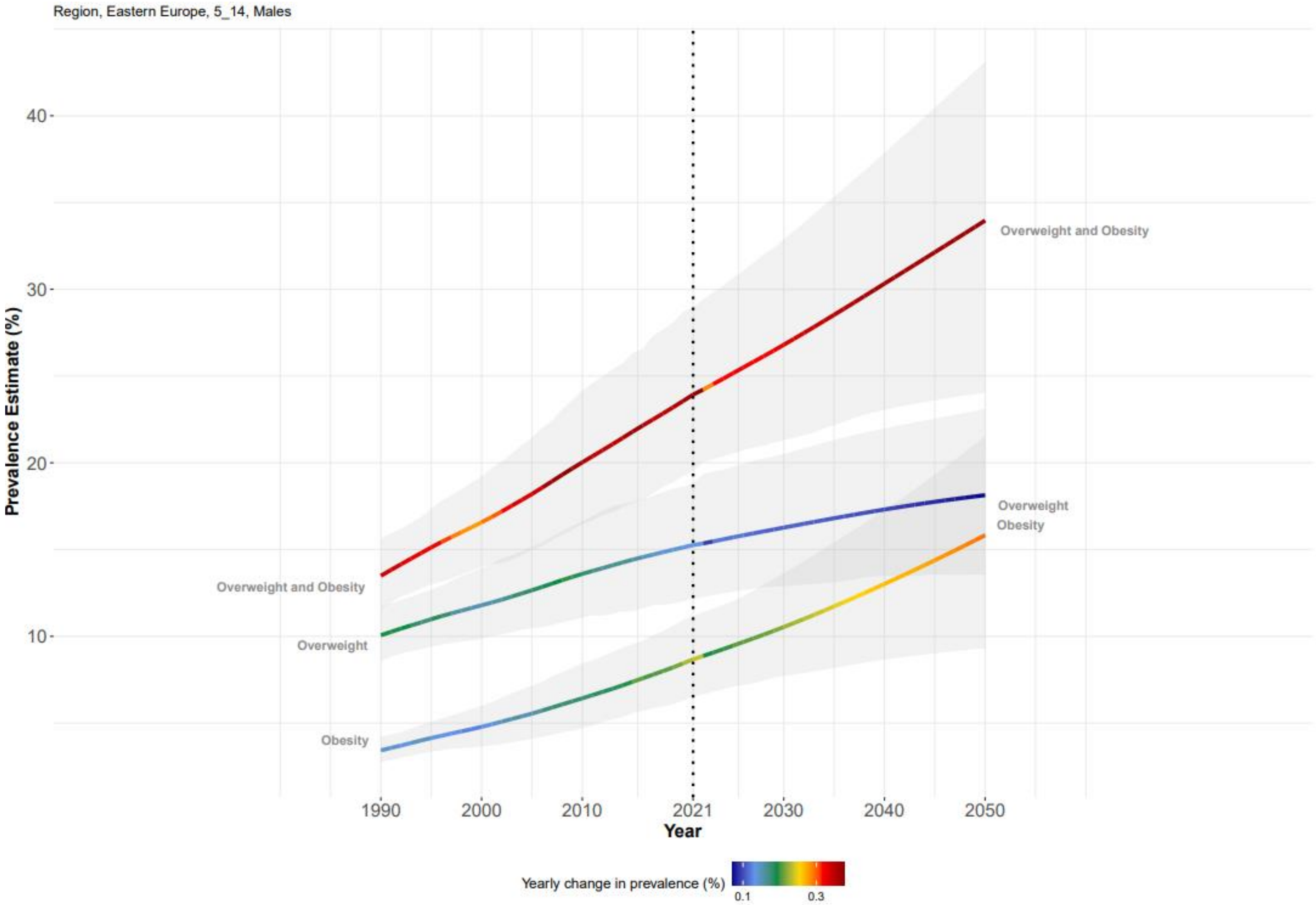

Region, Eastern Europe, 5_14, Males
Prevalence Estimate (%)
40
30
20
10
Overweight and Obesity
Overweight
Obesity
Overweight and Obesity
Overweight
Obesity
1990
2000
2010
2021
2030
2040
2050
Year
Yearly change in prevalence (%)
0.1
0.3

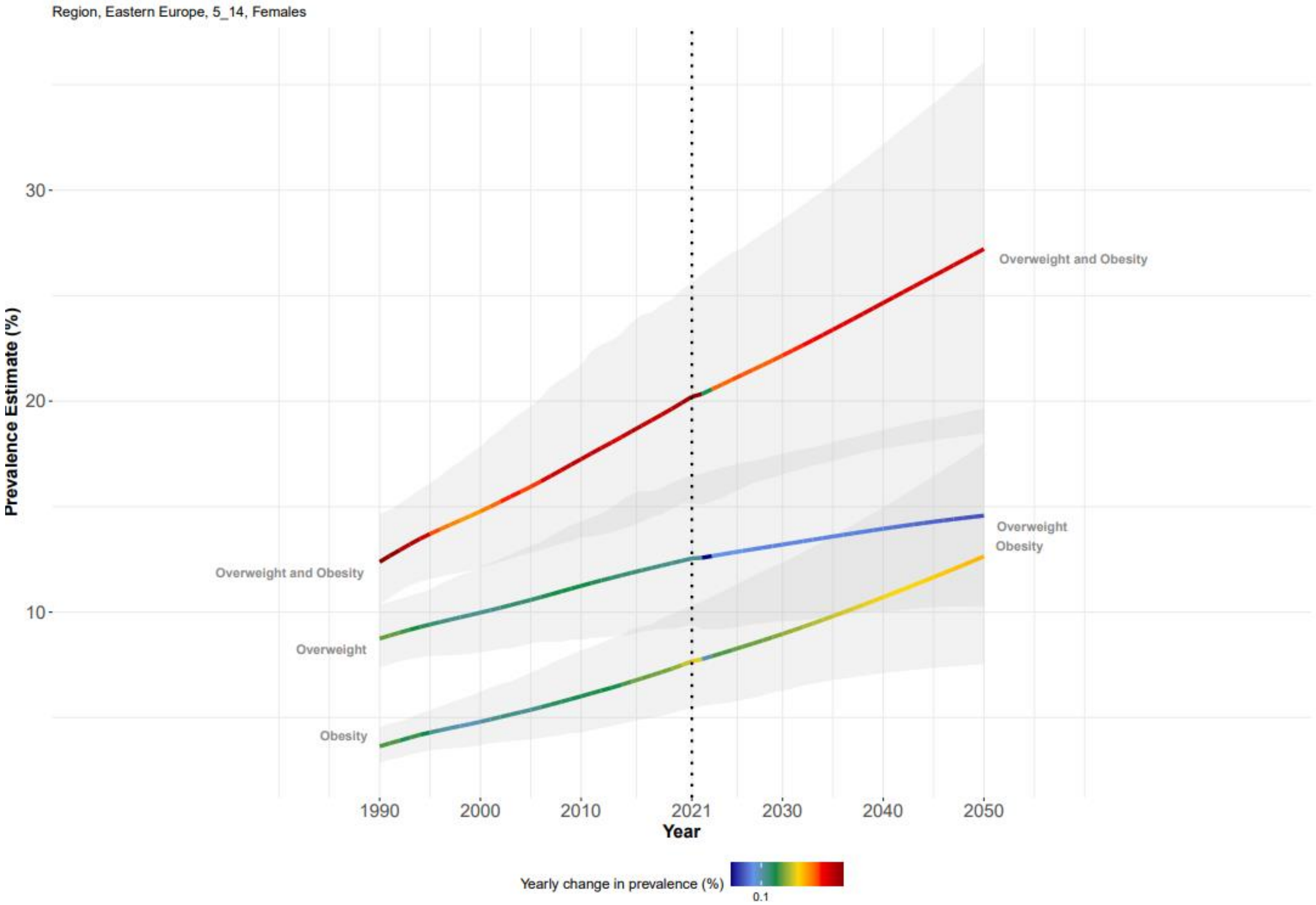

Region, Eastern Europe, 5_14, Females
Prevalence Estimate (%)
30
20
10
Overweight and Obesity
Overweight
Obesity
Overweight and Obesity
Overweight
Obesity
1990
2000
2010
2021
2030
2040
2050
Year
Yearly change in prevalence (%)
0.1

0

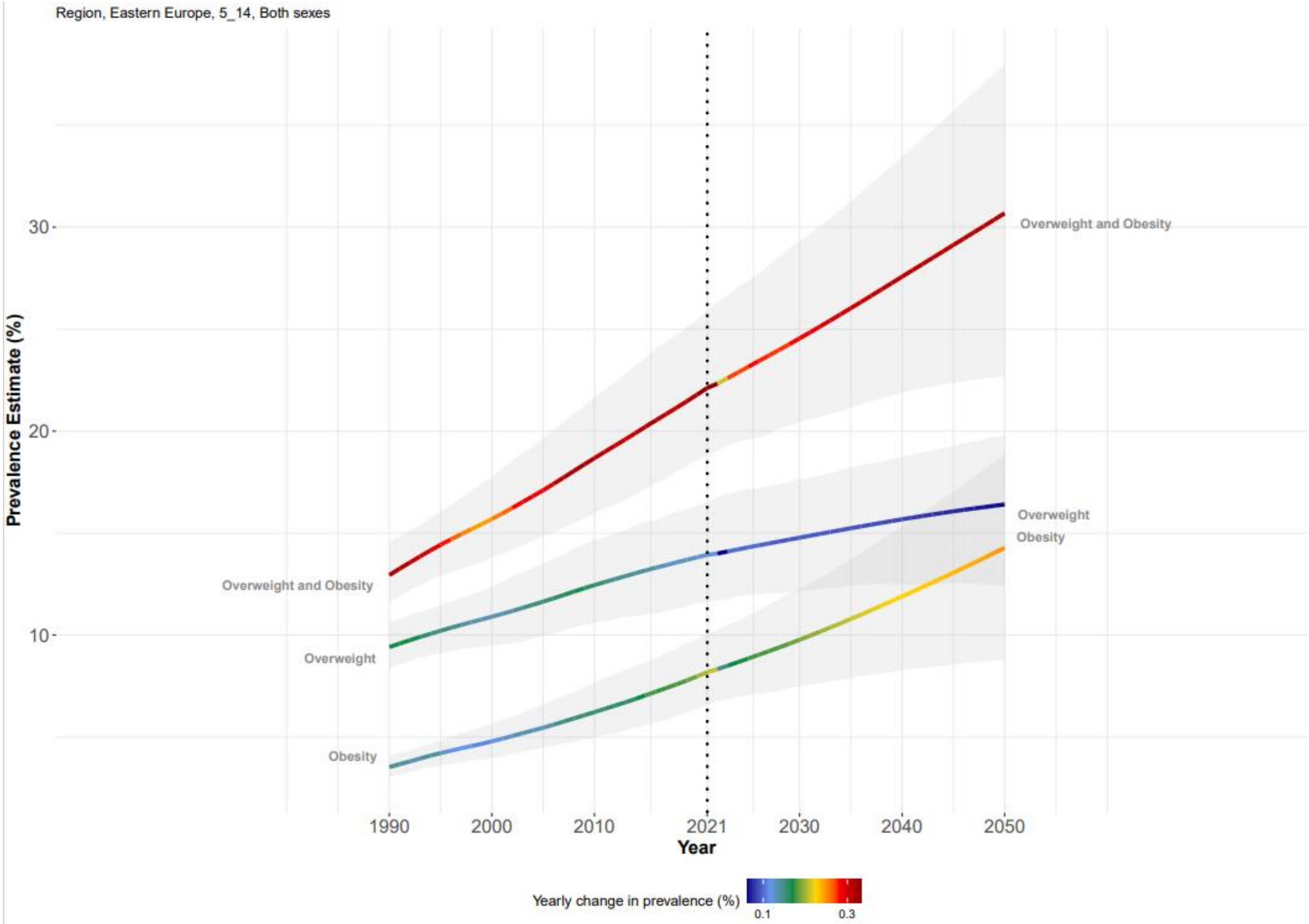

Region, Eastern Europe, 5_14, Both sexes
Prevalence Estimate (%)
30
20
10
Overweight and Obesity
Overweight
Obesity
Overweight and Obesity
Overweight
Obesity
1990
2000
2010
2021
2030
2040
2050
Year
Yearly change in prevalence (%)
0.1
0.3

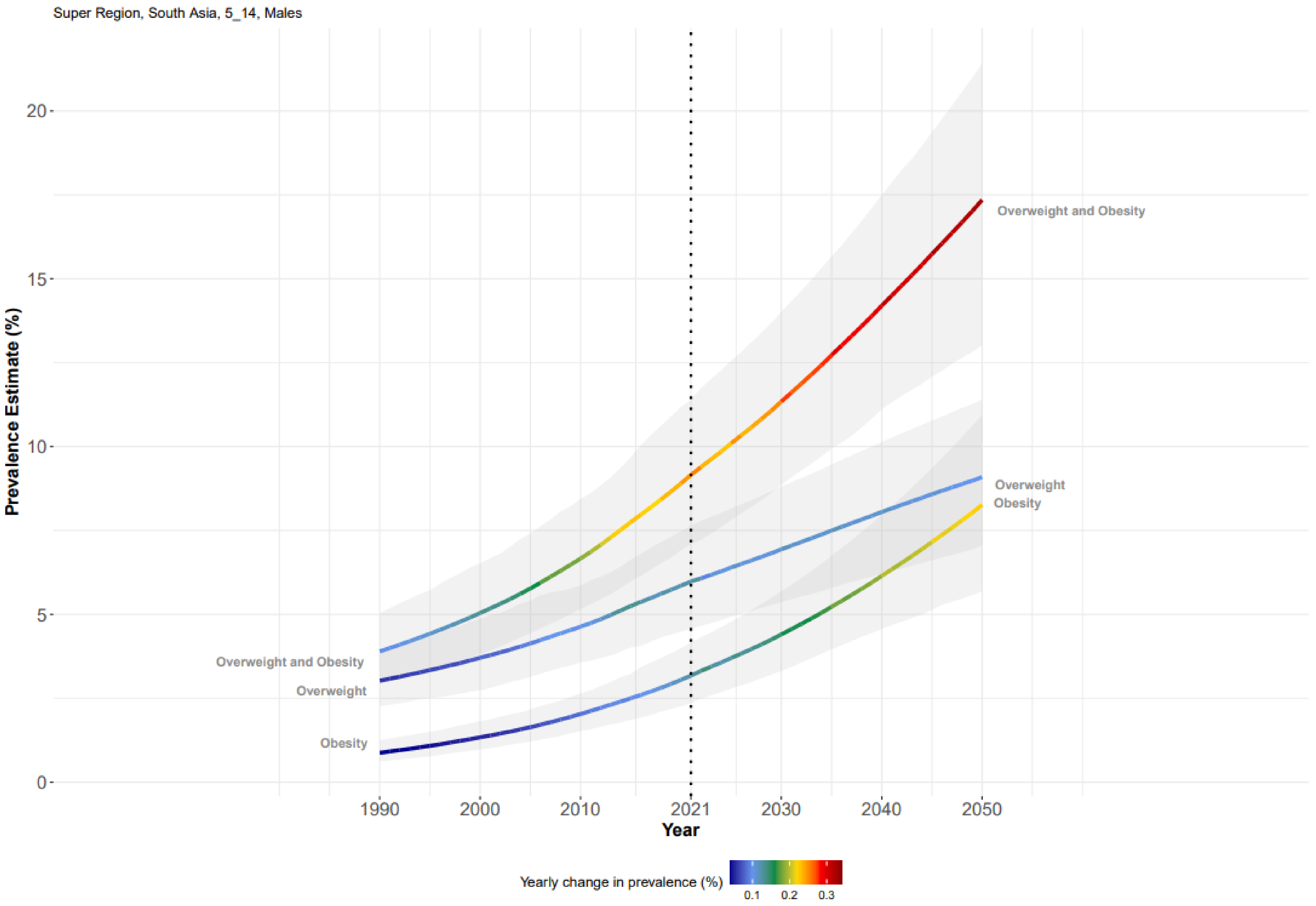

Super Region, South Asia, 5_14, Males
Prevalence Estimate (%)
20
15
10
5
0
1990
2000
2010
2021
2030
2040
2050
Year
Overweight and Obesity
Overweight
Obesity
Overweight and Obesity
Overweight
Obesity
Yearly change in prevalence (%)
0.1
0.2
0.3

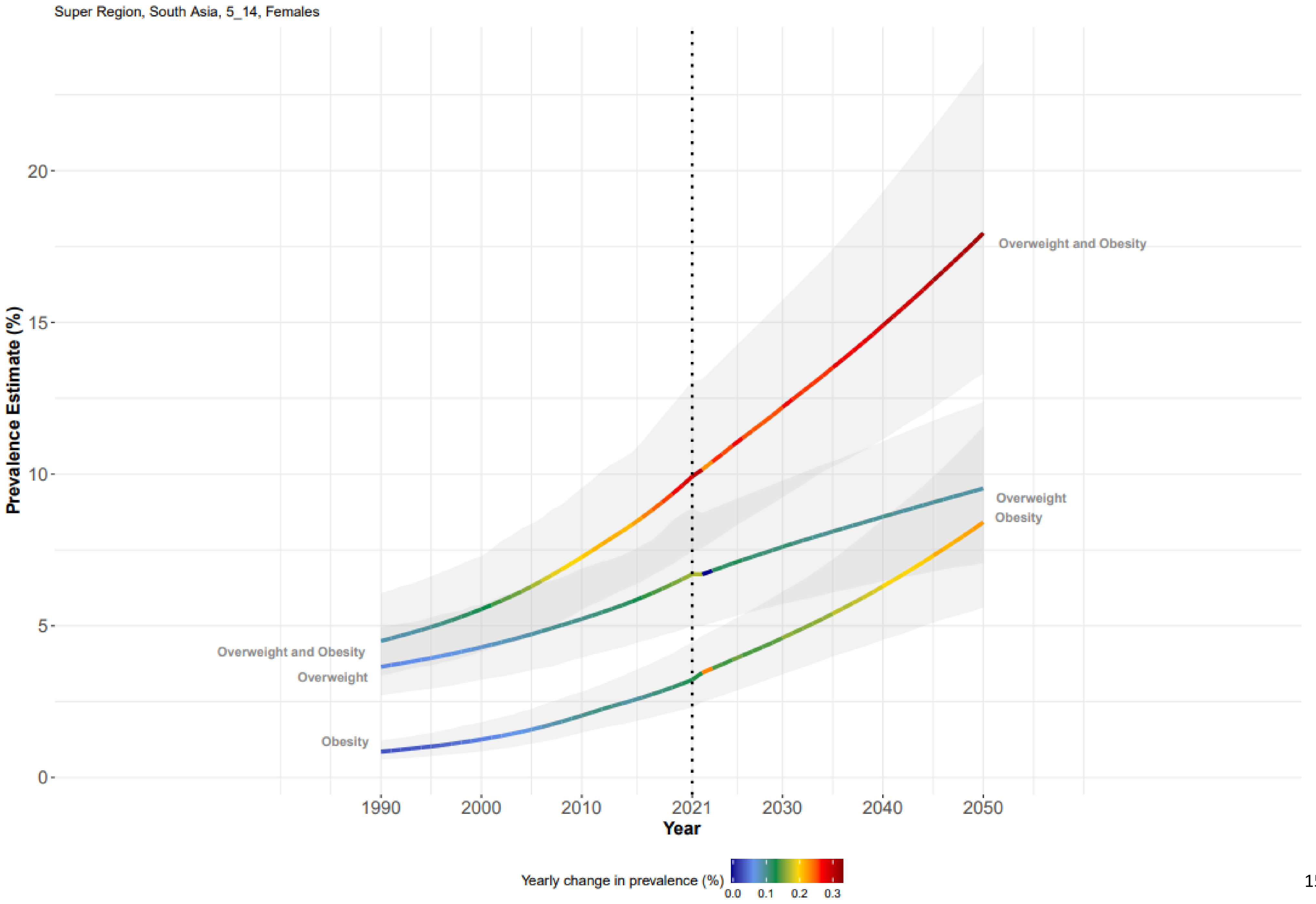
Super Region, South Asia, 5_14, Females
Prevalence Estimate (%)
20
15
10
5
0
1990
2000
2010
2021
2030
2040
2050
Year
Overweight and Obesity
Overweight
Obesity
Overweight and Obesity
Overweight
Obesity
Yearly change in prevalence (%)
0.0
0.1
0.2
0.3

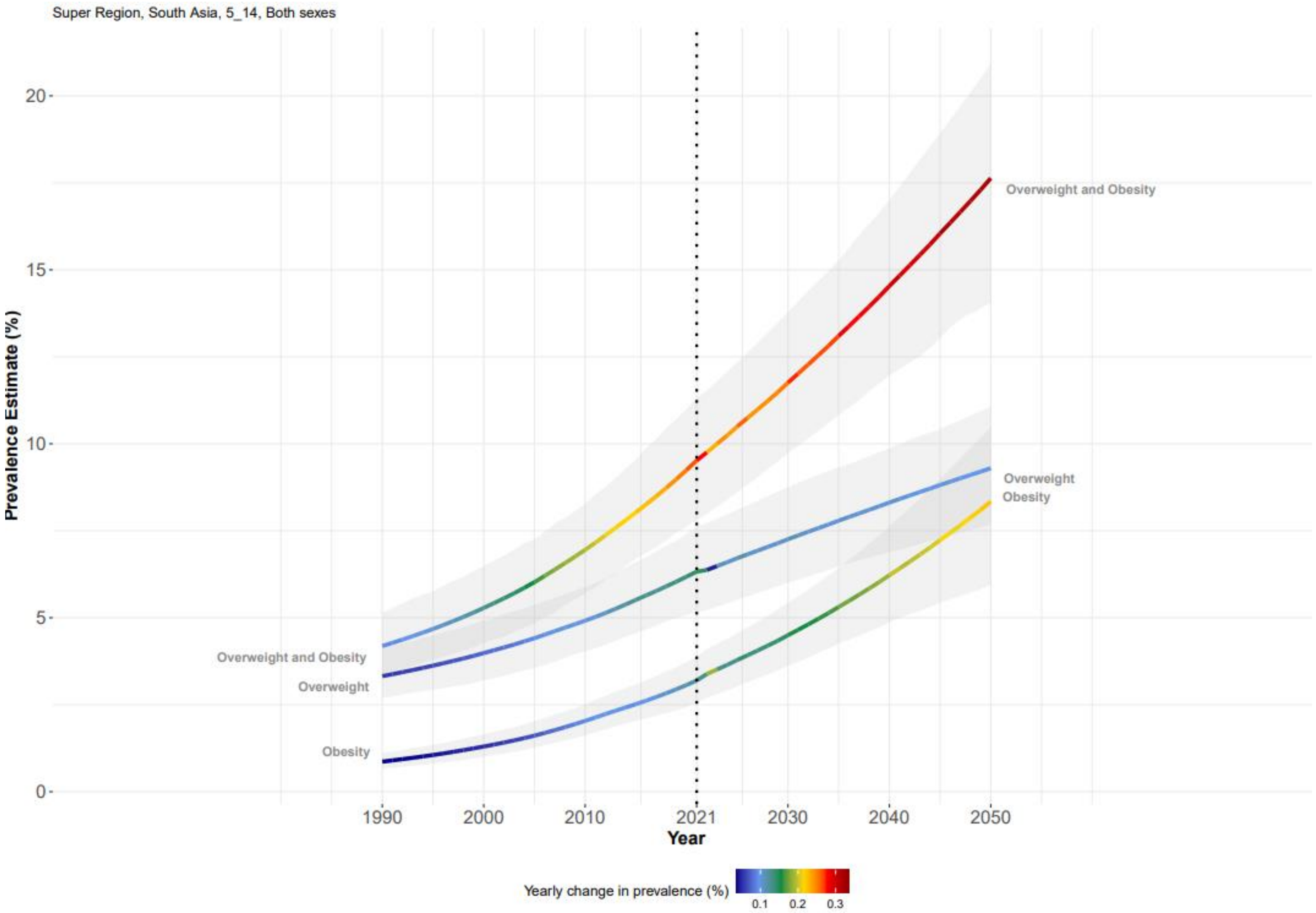

Super Region, South Asia, 5_14, Both sexes
Prevalence Estimate (%)
20
15
10
5
0
1990
2000
2010
2021
2030
2040
2050
Year
Overweight and Obesity
Overweight
Obesity
Overweight and Obesity
Overweight
Obesity
Yearly change in prevalence (%)
0.1
0.2
0.3

Figure S3: Age-standardised prevalence estimates from 1990 to 2021 and forecasts to 2050 for older adolescents aged 15 to 24 years old

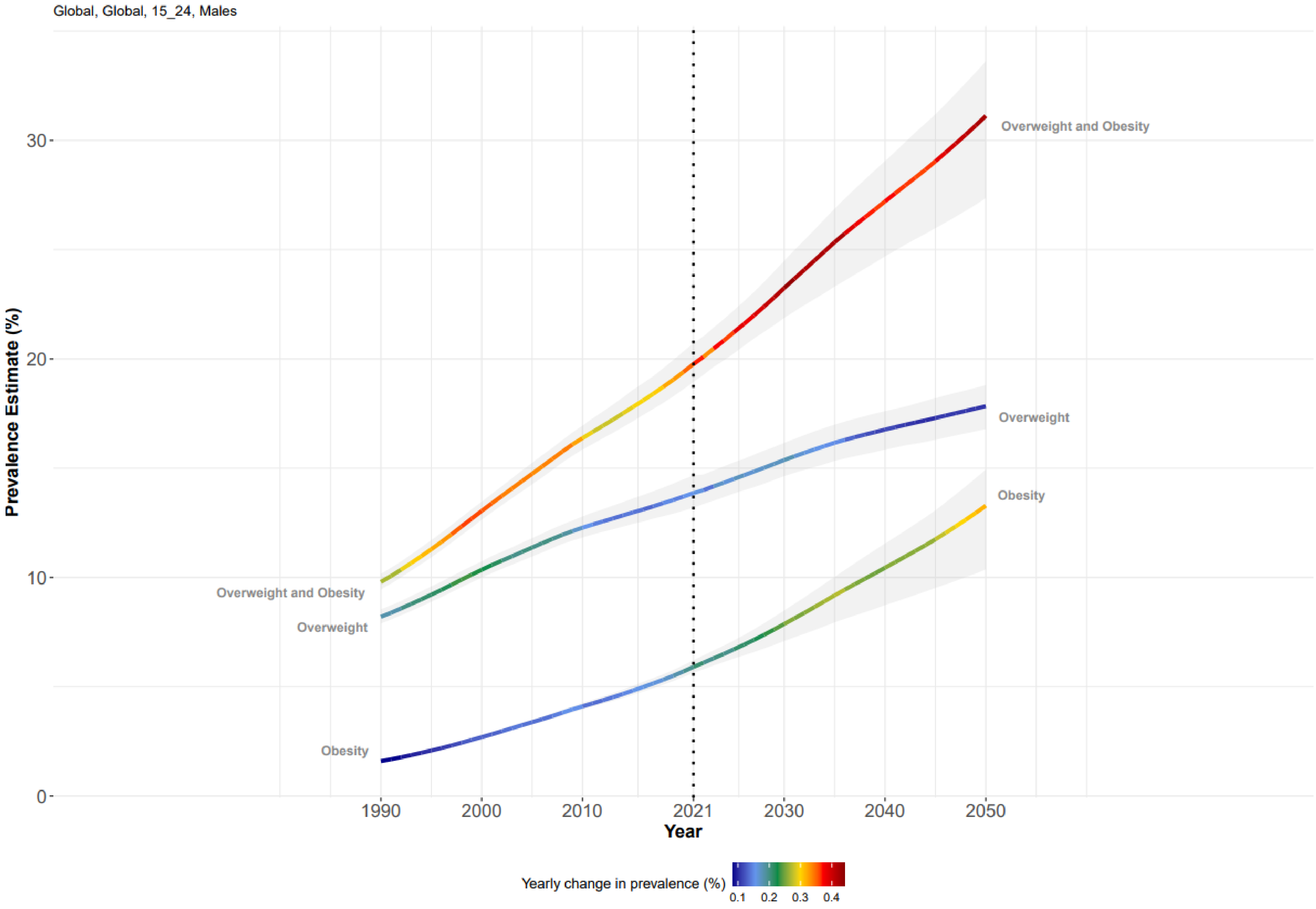

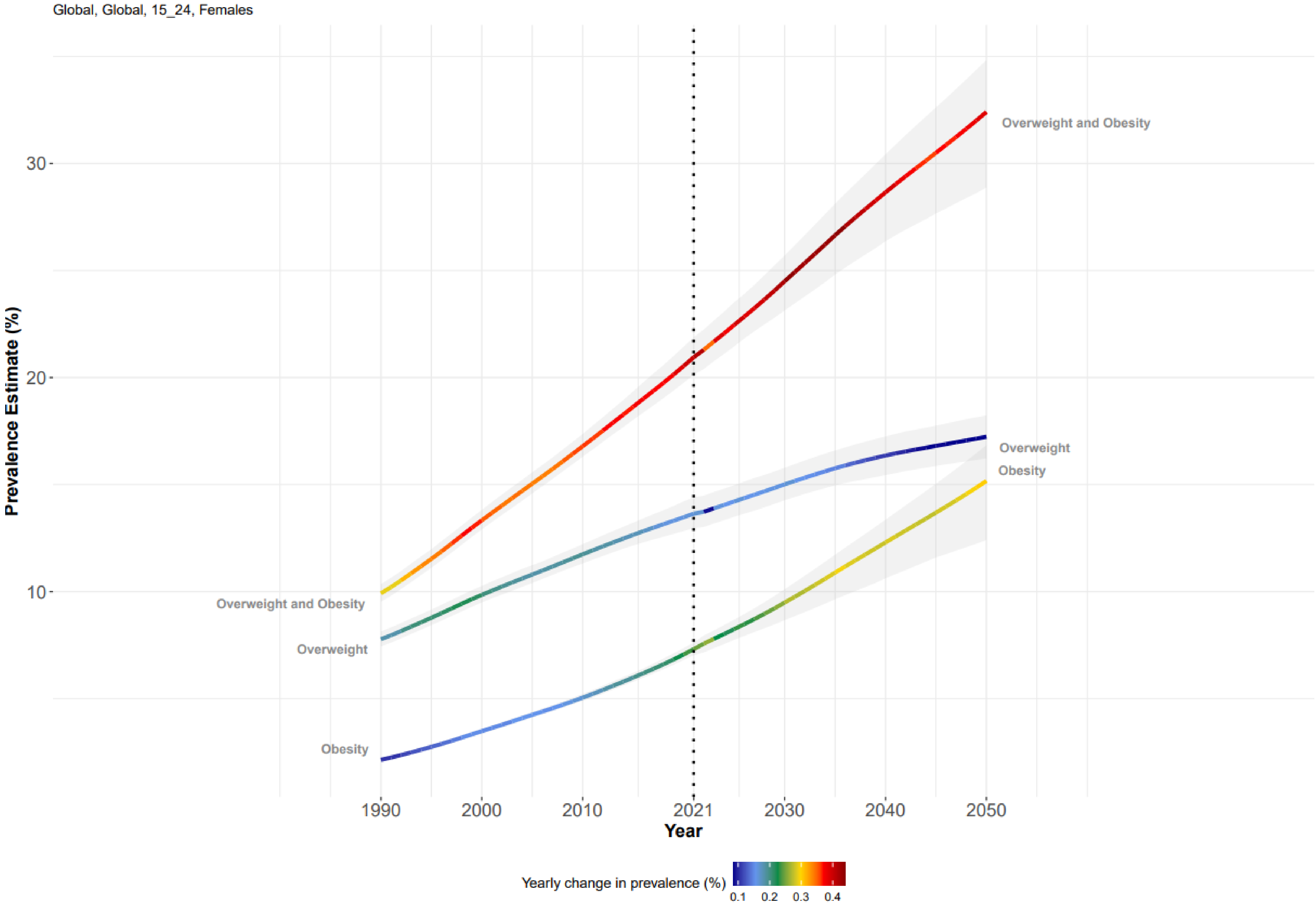
Global, Global, 15_24, Females
Prevalence Estimate (%)
30
20
10
Overweight and Obesity
Overweight
Obesity
Overweight and Obesity
Overweight
Obesity
1990
2000
2010
2021
2030
2040
2050
Year
Yearly change in prevalence (%)
0.1
0.2
0.3
0.4

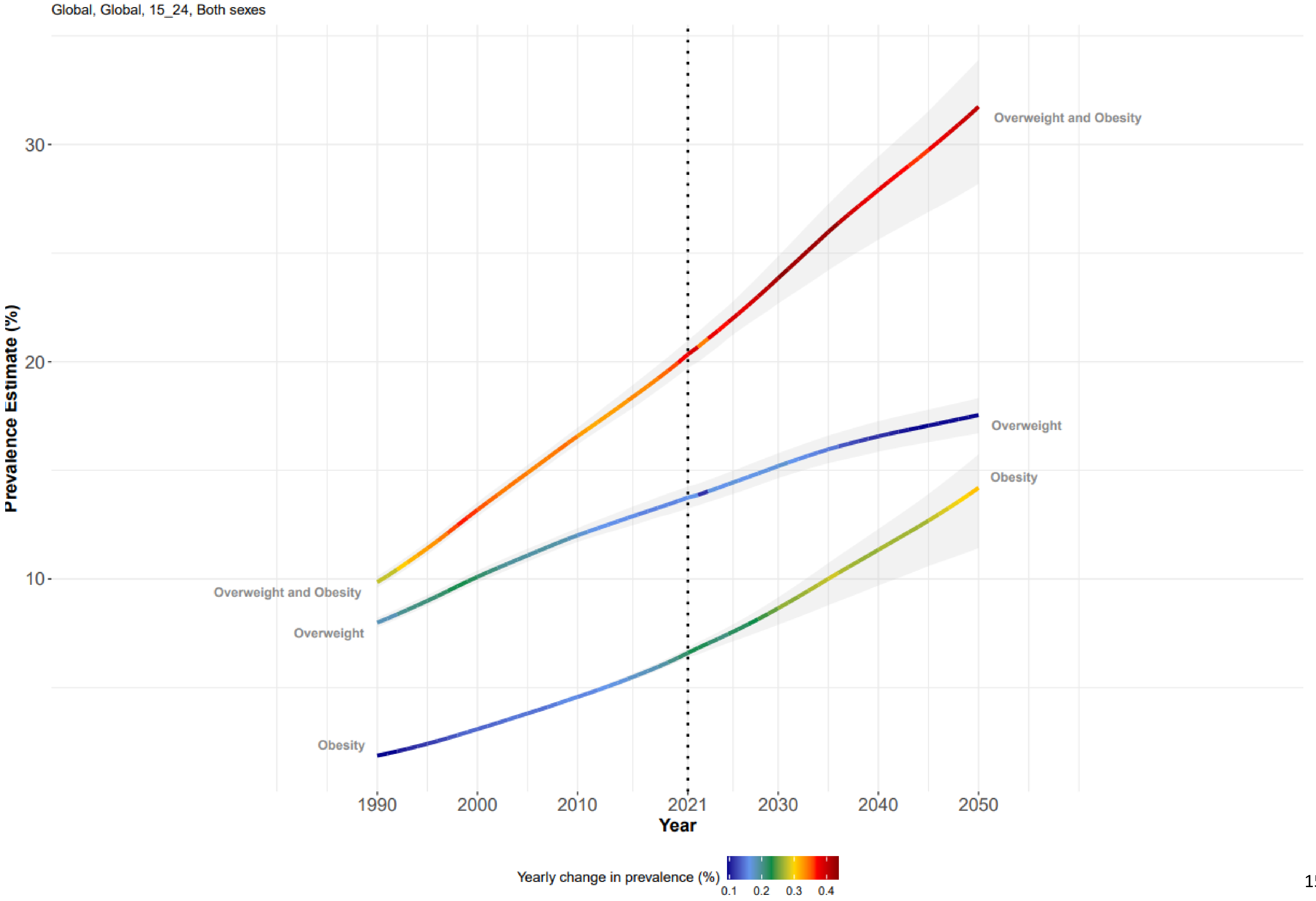

Global, Global, 15_24, Both sexes
Prevalence Estimate (%)
30
20
10
Overweight and Obesity
Overweight
Obesity
Overweight and Obesity
Overweight
Obesity
1990
2000
2010
2021
2030
2040
2050
Year
Yearly change in prevalence (%)
0.1
0.2
0.3
0.4

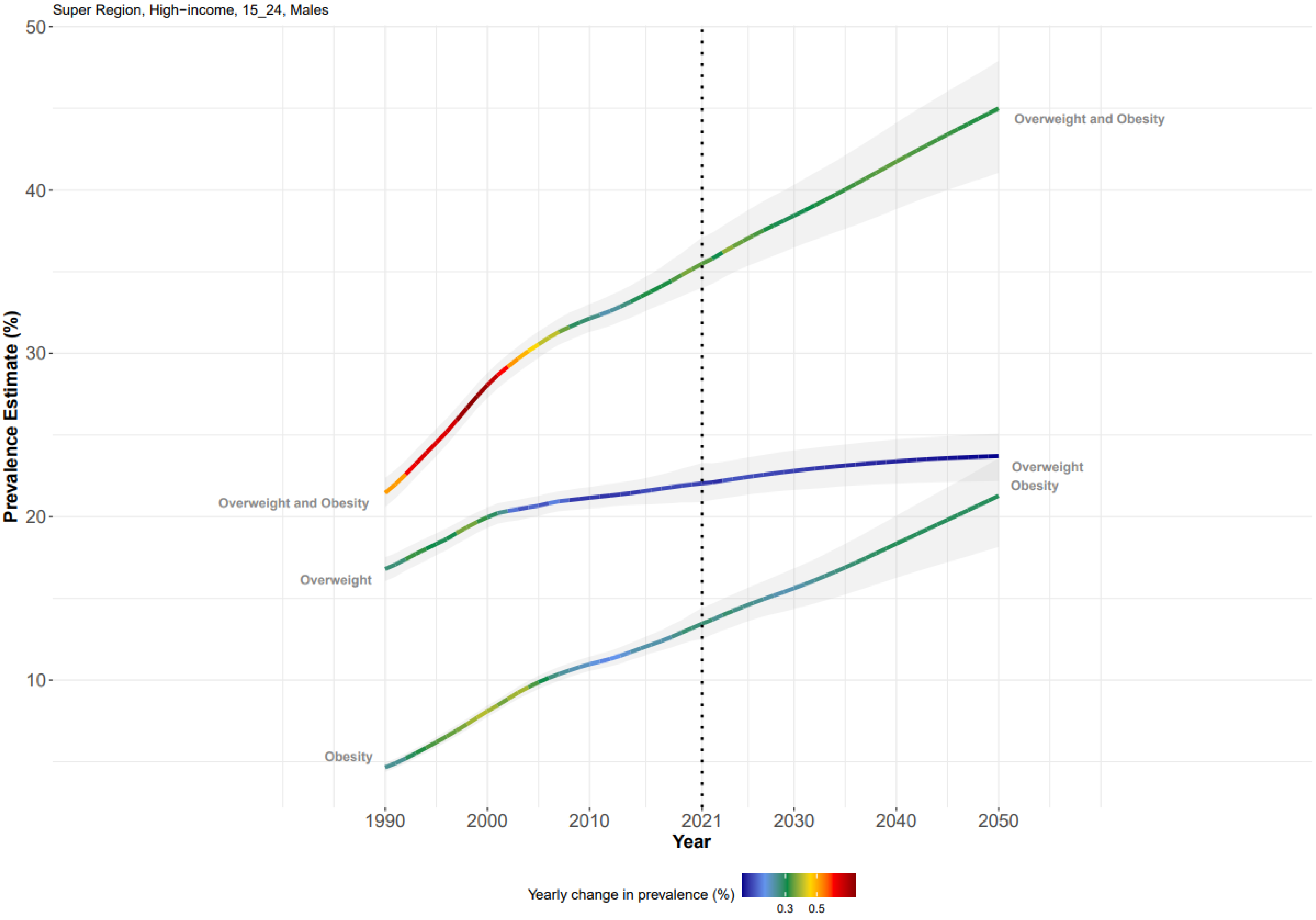

Super Region, High−income, 15_24, Males
Prevalence Estimate (%)
50
40
30
20
10
Overweight and Obesity
Overweight
Obesity
Overweight and Obesity
Overweight
Obesity
1990
2000
2010
2021
2030
2040
2050
Year
Yearly change in prevalence (%)
0.3
0.5

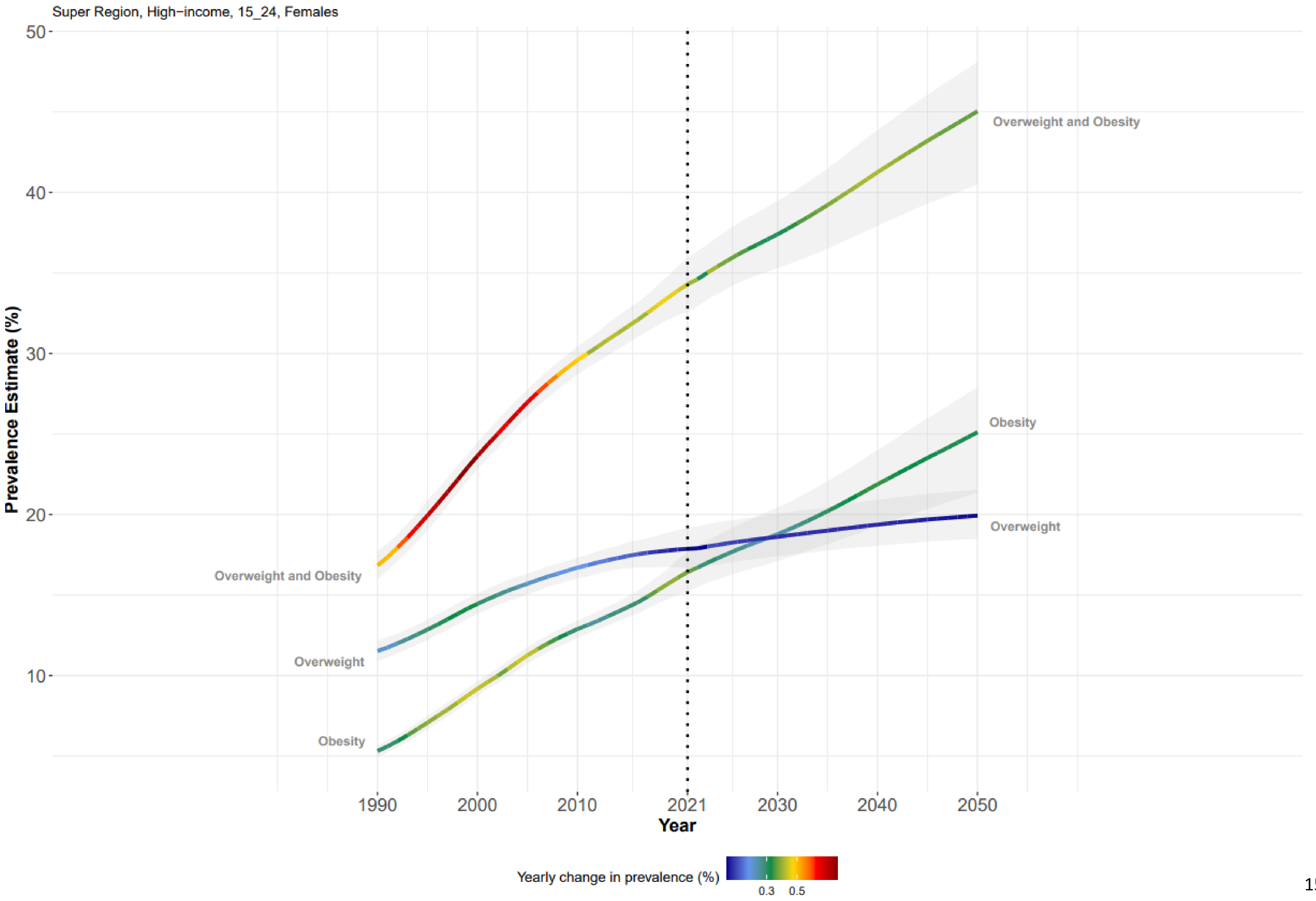
Super Region, High−income, 15_24, Females
Prevalence Estimate (%)
50
40
30
20
10
Overweight and Obesity
Obesity
Overweight
Overweight and Obesity
Overweight
Obesity
1990
2000
2010
2021
2030
2040
2050
Year
Yearly change in prevalence (%)
0.3
0.5

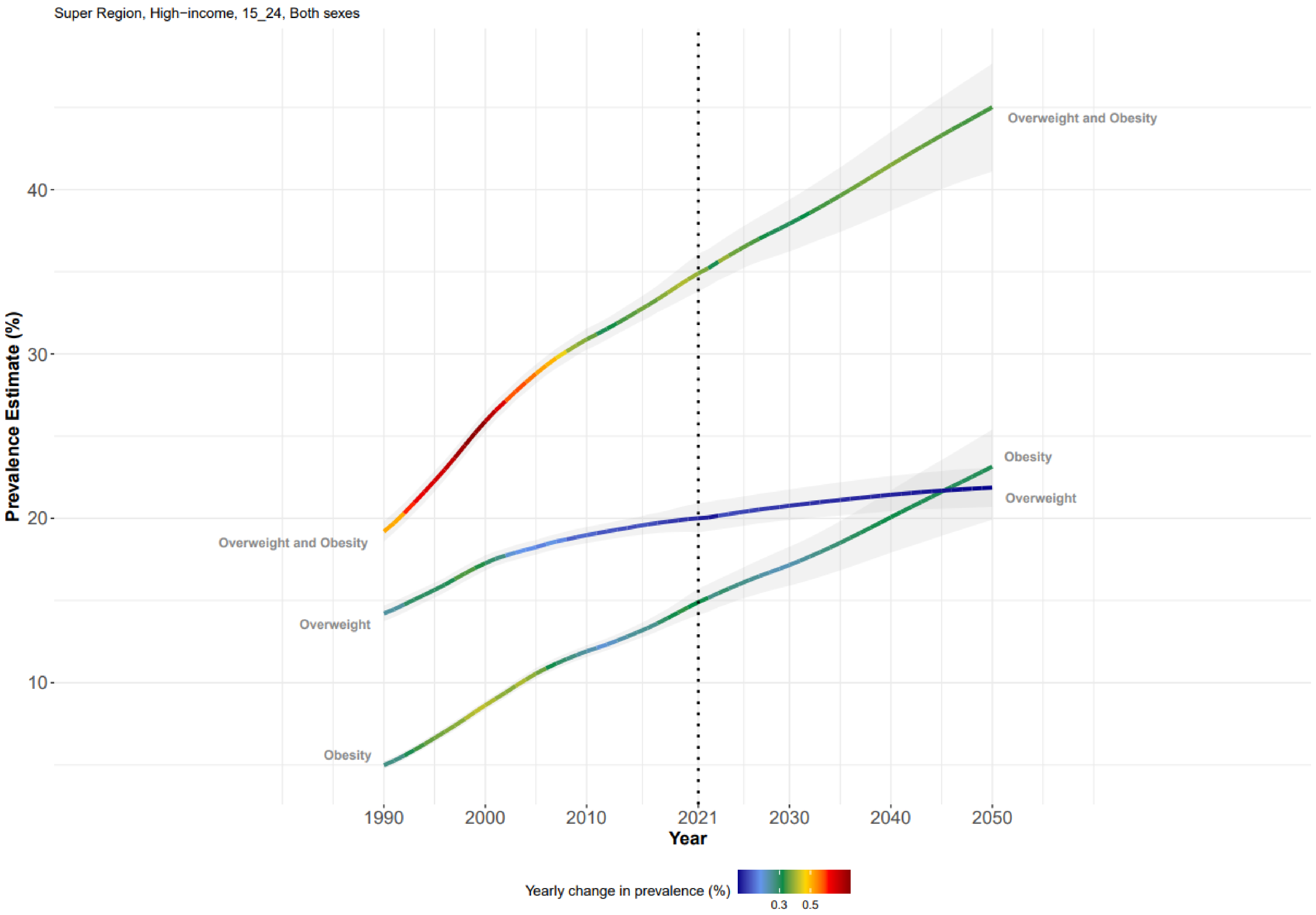

Super Region, High−income, 15_24, Both sexes
Prevalence Estimate (%)
40
30
20
10
Overweight and Obesity
Obesity
Overweight
Overweight and Obesity
Overweight
Obesity
1990
2000
2010
2021
2030
2040
2050
Year
Yearly change in prevalence (%)
0.3
0.5

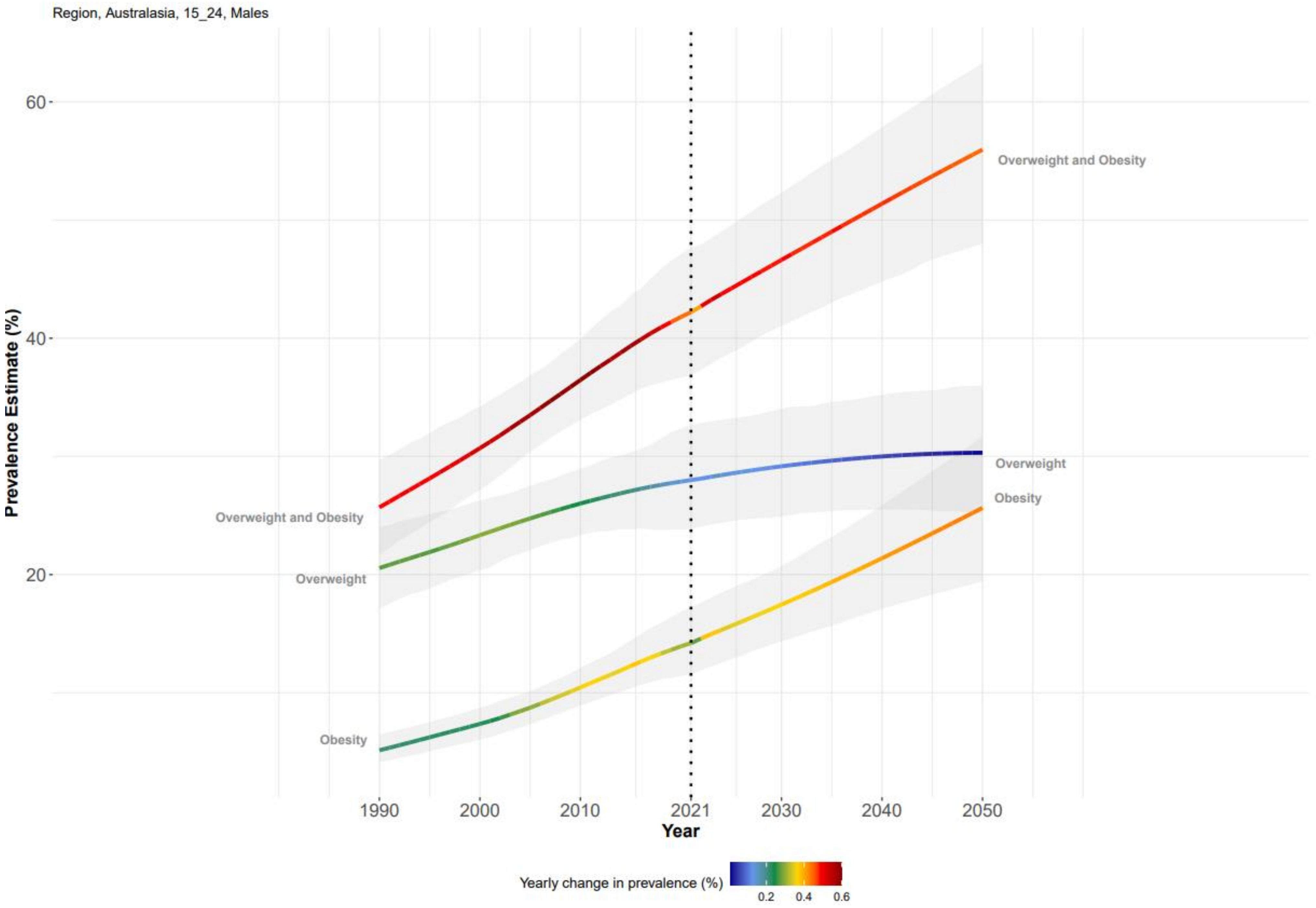

Region, Australasia, 15_24, Males
Prevalence Estimate (%)
60
40
20
Overweight and Obesity
Overweight
Obesity
Overweight and Obesity
Overweight
Obesity
1990
2000
2010
2021
2030
2040
2050
Year
Yearly change in prevalence (%)
0.2
0.4
0.6

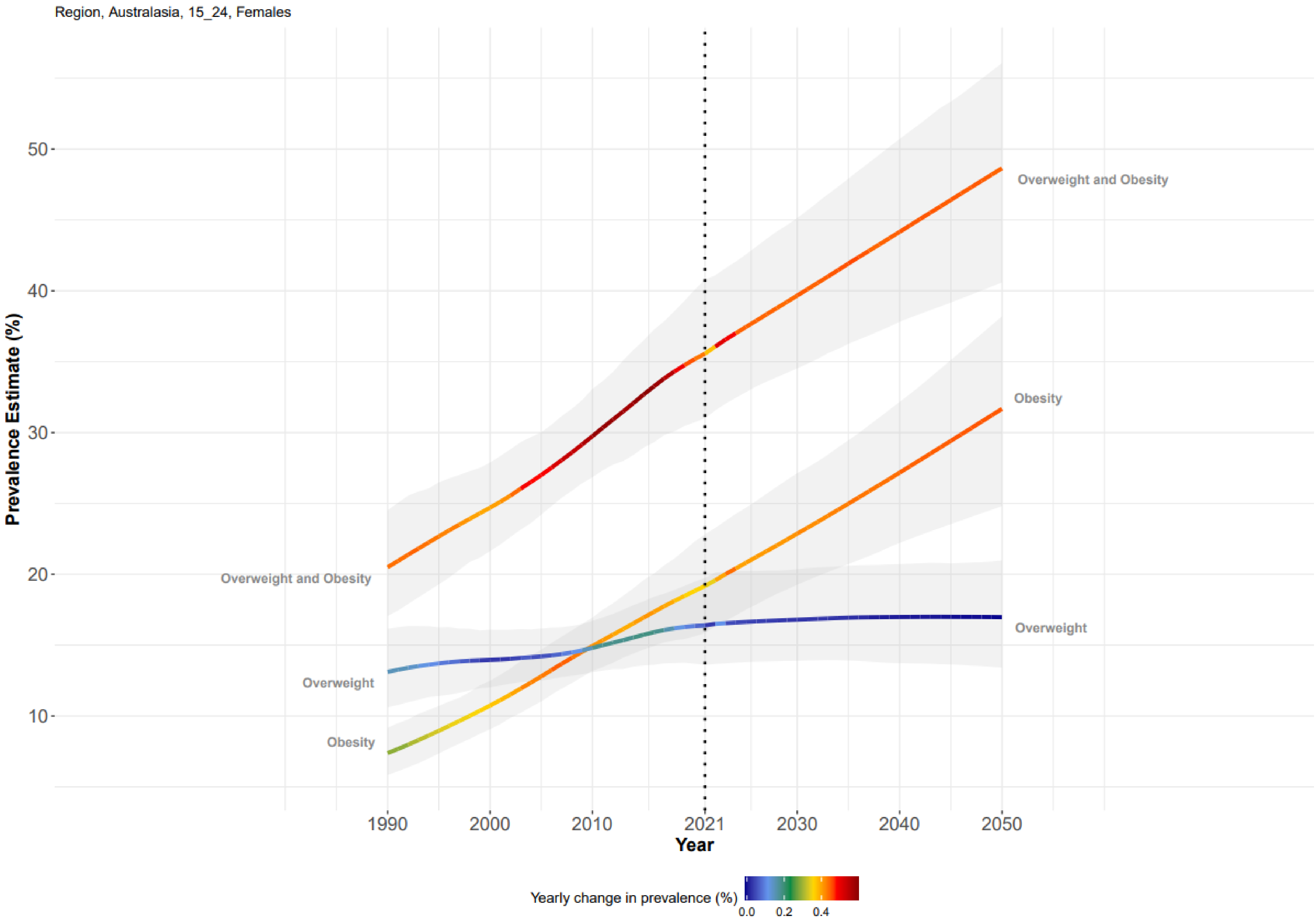

Region, Australasia, 15_24, Females
Prevalence Estimate (%)
50
40
30
20
10
Overweight and Obesity
Overweight
Obesity
Overweight and Obesity
Obesity
Overweight
1990
2000
2010
2021
2030
2040
2050
Year
Yearly change in prevalence (%)
0.0
0.2
0.4

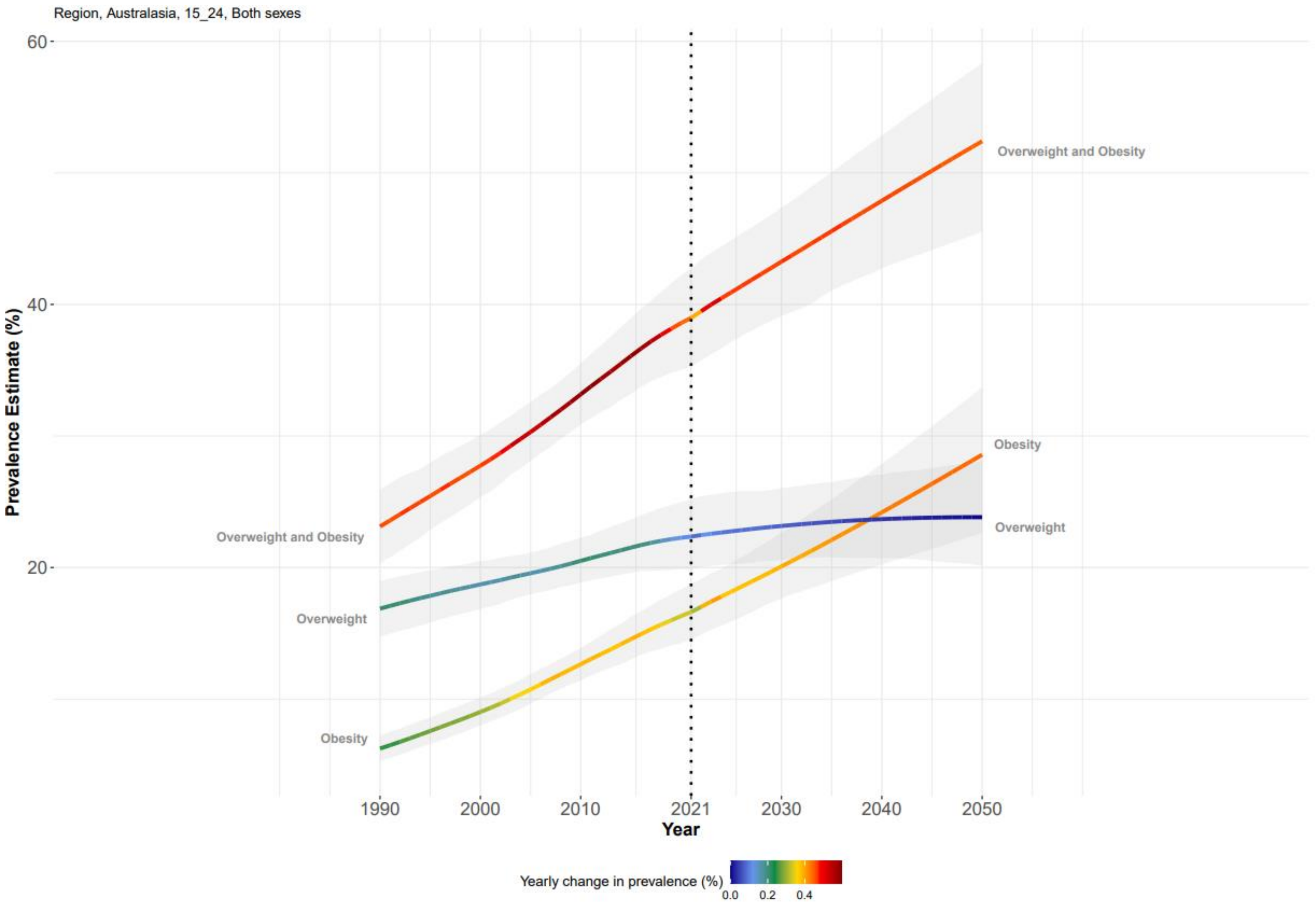

Region, Australasia, 15_24, Both sexes
60
40
20
Prevalence Estimate (%)
Overweight and Obesity
Obesity
Overweight
Overweight and Obesity
Overweight
Obesity
1990
2000
2010
2021
2030
2040
2050
Year
Yearly change in prevalence (%)
0.0
0.2
0.4

Region, High−income Asia Pacific, 15_24, Males

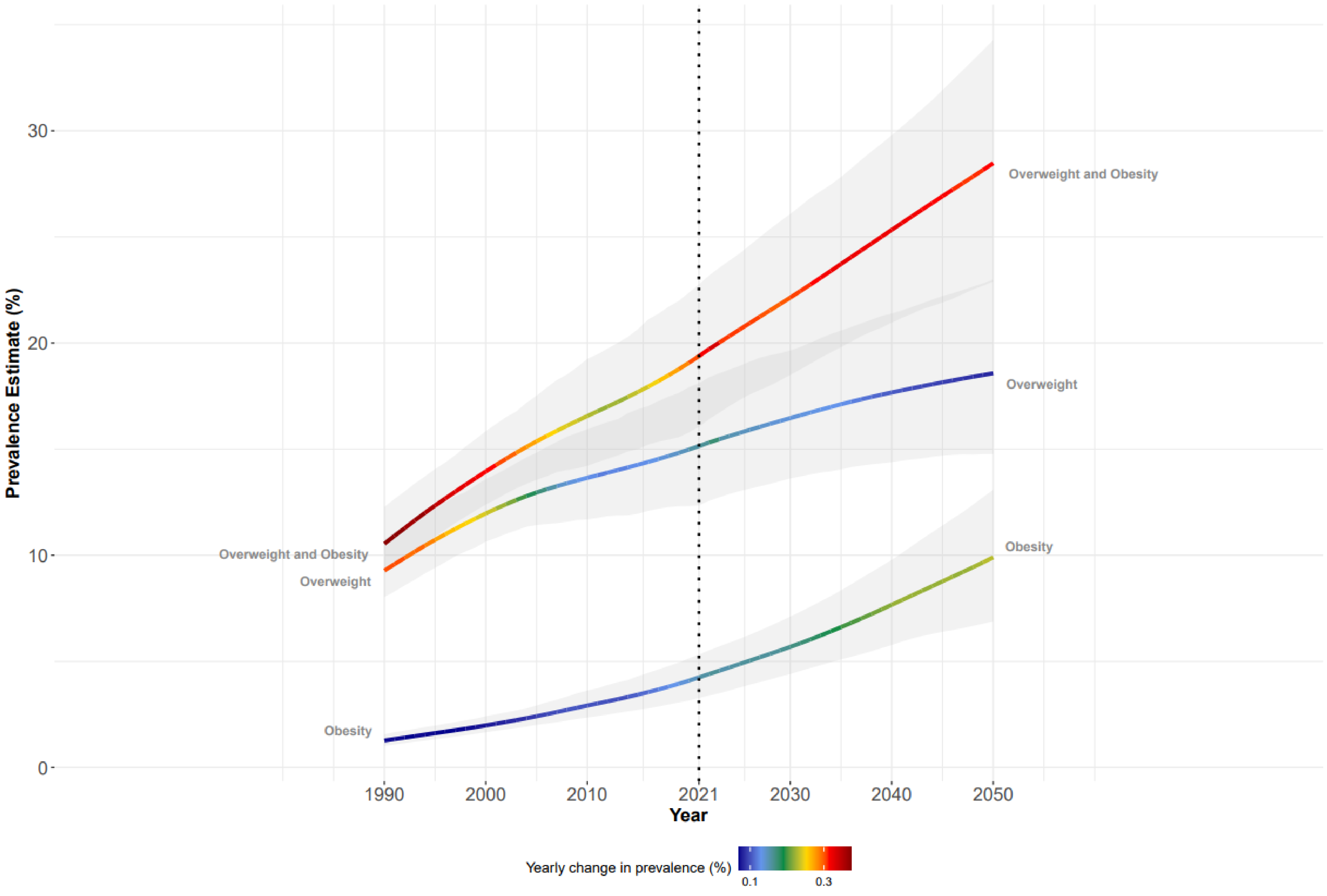

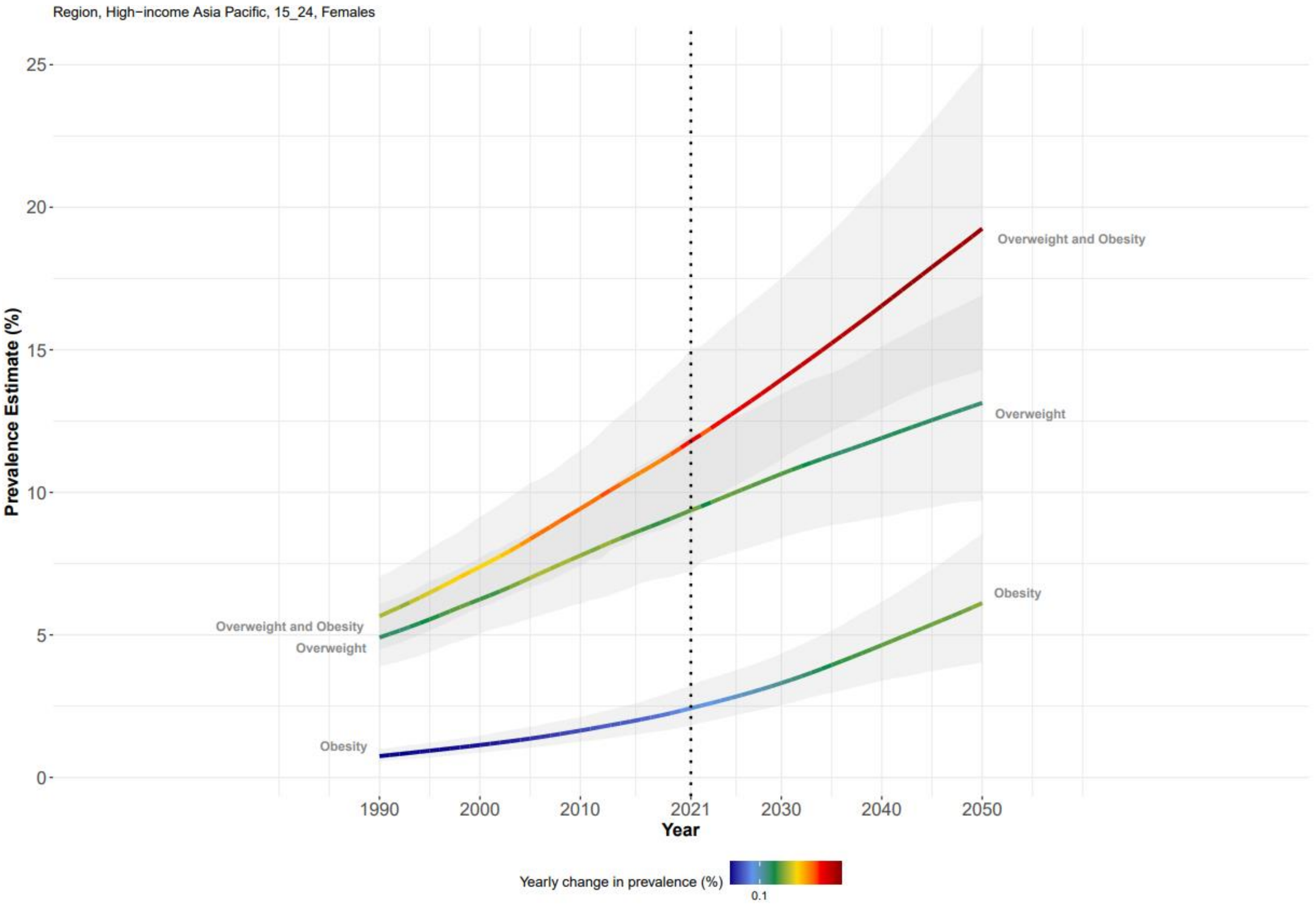
Region, High−income Asia Pacific, 15_24, Females
Prevalence Estimate (%)
25
20
15
10
5
0
1990
2000
2010
2021
2030
2040
2050
Year
Overweight and Obesity
Overweight
Obesity
Overweight and Obesity
Overweight
Obesity
Yearly change in prevalence (%)
0.1

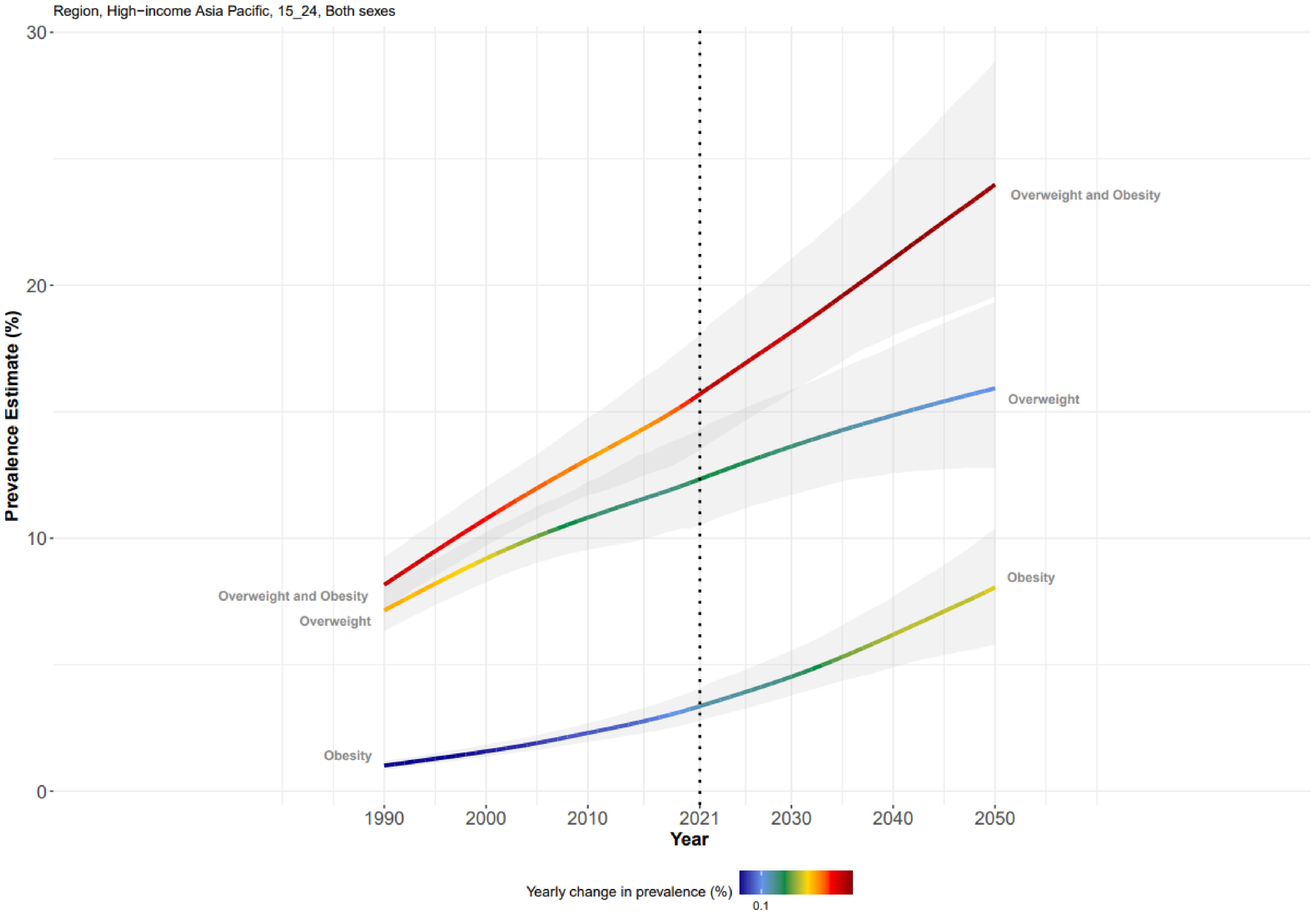

Region, High−income Asia Pacific, 15_24, Both sexes
Prevalence Estimate (%)
30
20
10
0
Overweight and Obesity
Overweight
Obesity
Overweight and Obesity
Overweight
Obesity
1990
2000
2010
2021
2030
2040
2050
Year
Yearly change in prevalence (%)
0.1

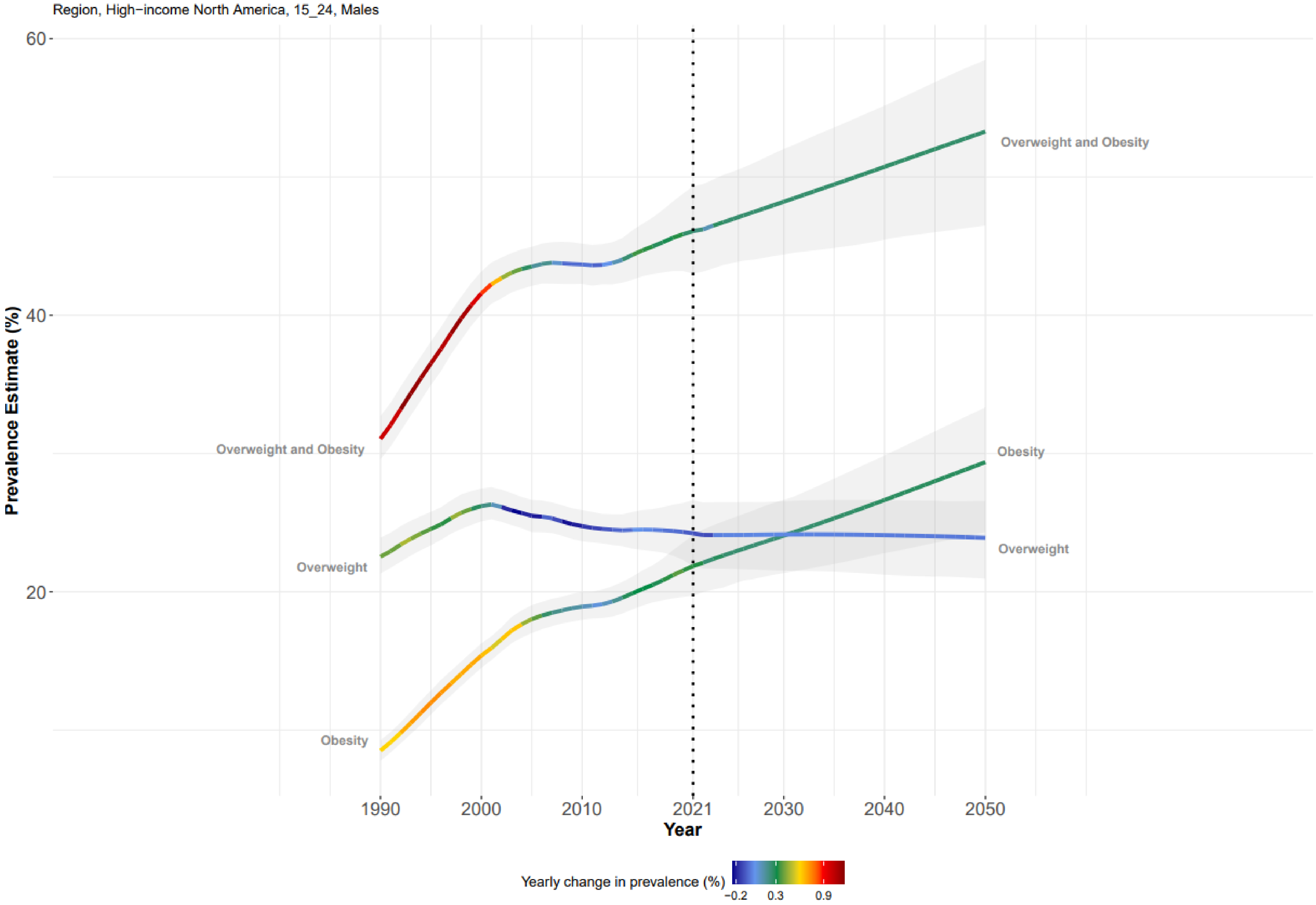
Region, High−income North America, 15_24, Males
60
40
20
Prevalence Estimate (%)
Overweight and Obesity
Overweight
Obesity
Overweight and Obesity
Obesity
Overweight
1990
2000
2010
2021
2030
2040
2050
Year
Yearly change in prevalence (%)
−0.2
0.3
0.9

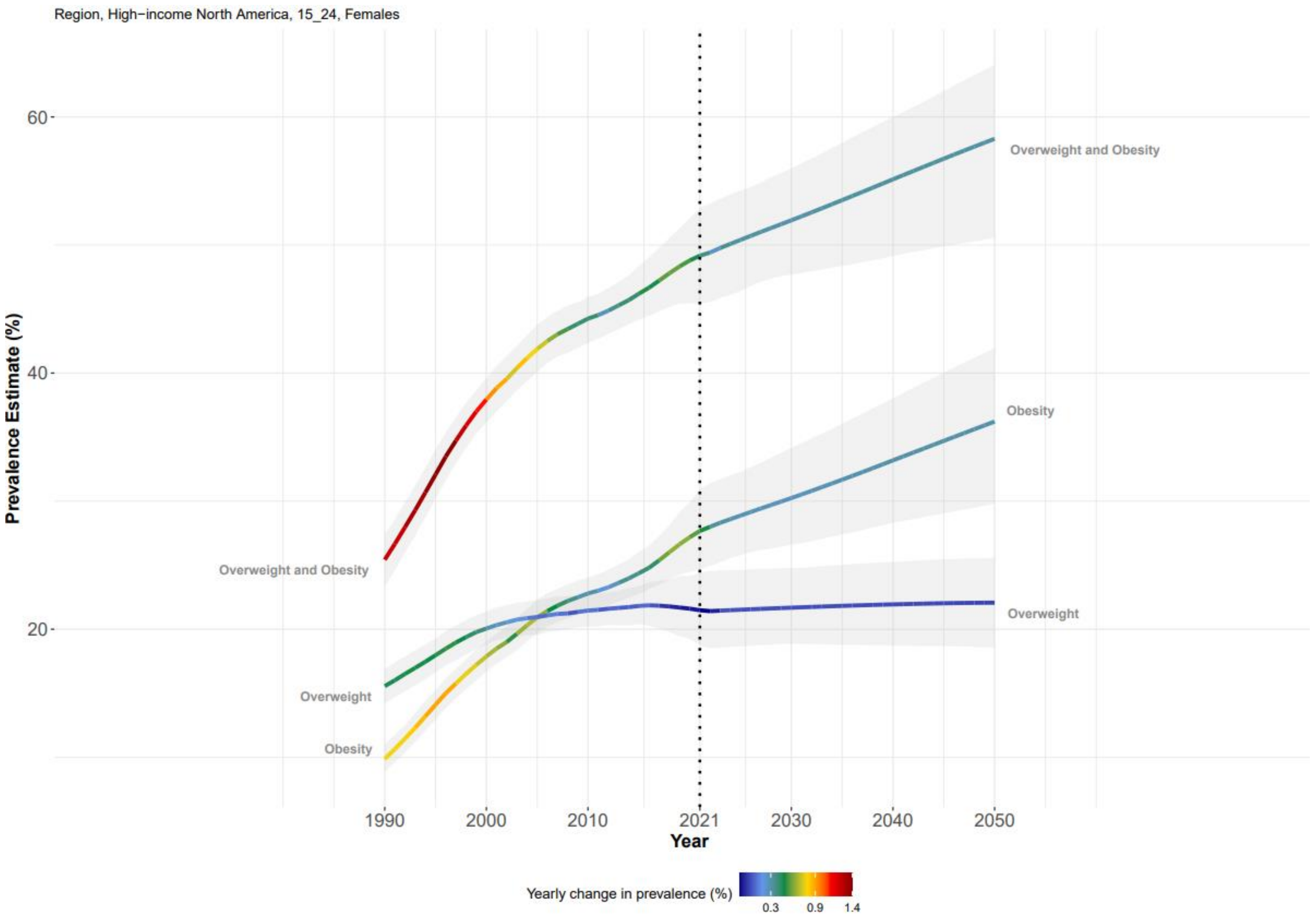
Region, High−income North America, 15_24, Females
Prevalence Estimate (%)
60
40
20
Overweight and Obesity
Obesity
Overweight
Overweight and Obesity
Overweight
Obesity
1990
2000
2010
2021
2030
2040
2050
Year
Yearly change in prevalence (%)
0.3
0.9
1.4

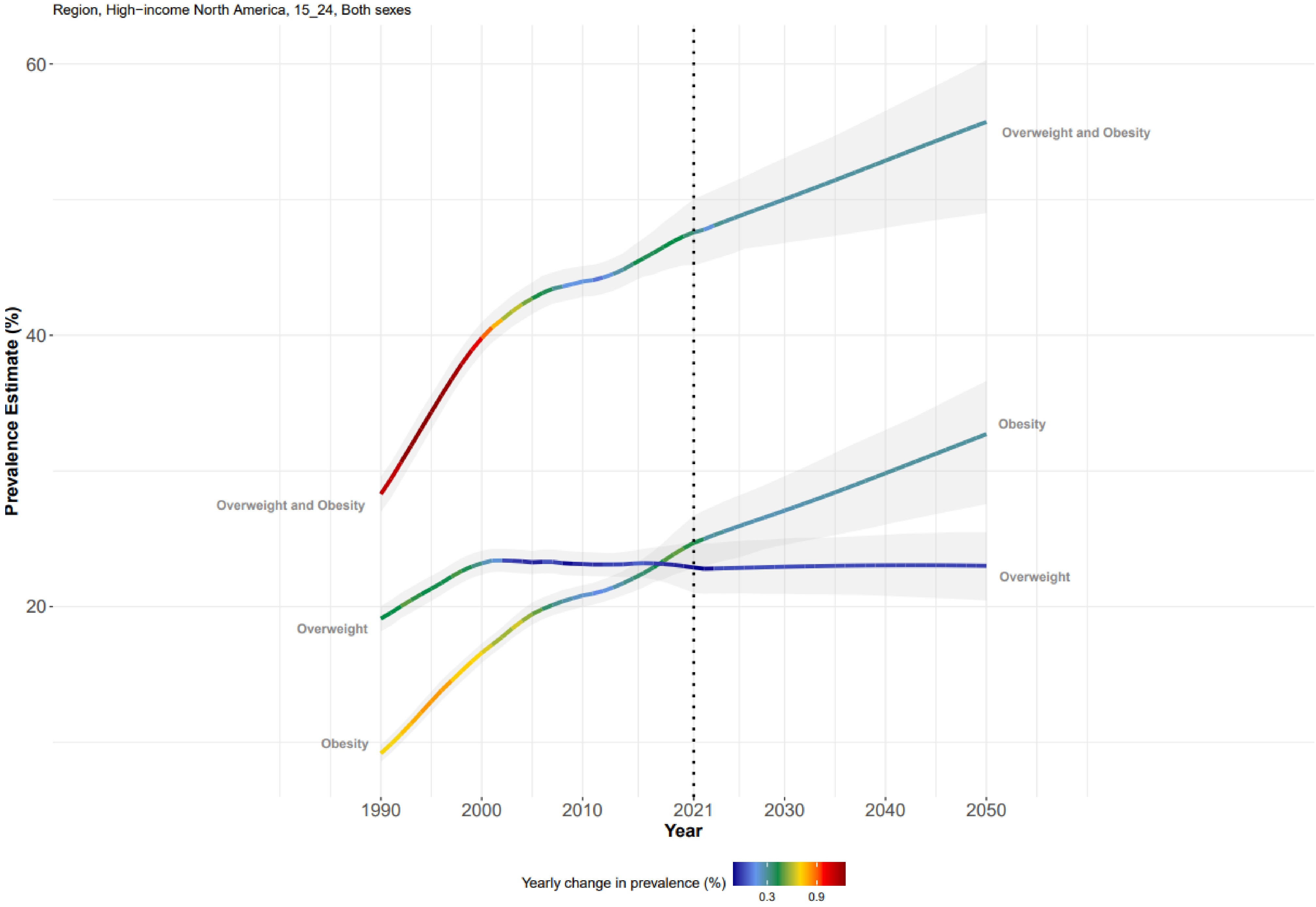

Region, High−income North America, 15_24, Both sexes
60
40
20
Prevalence Estimate (%)
Overweight and Obesity
Overweight
Obesity
Overweight and Obesity
Obesity
Overweight
1990
2000
2010
2021
2030
2040
2050
Year
Yearly change in prevalence (%)
0.3
0.9

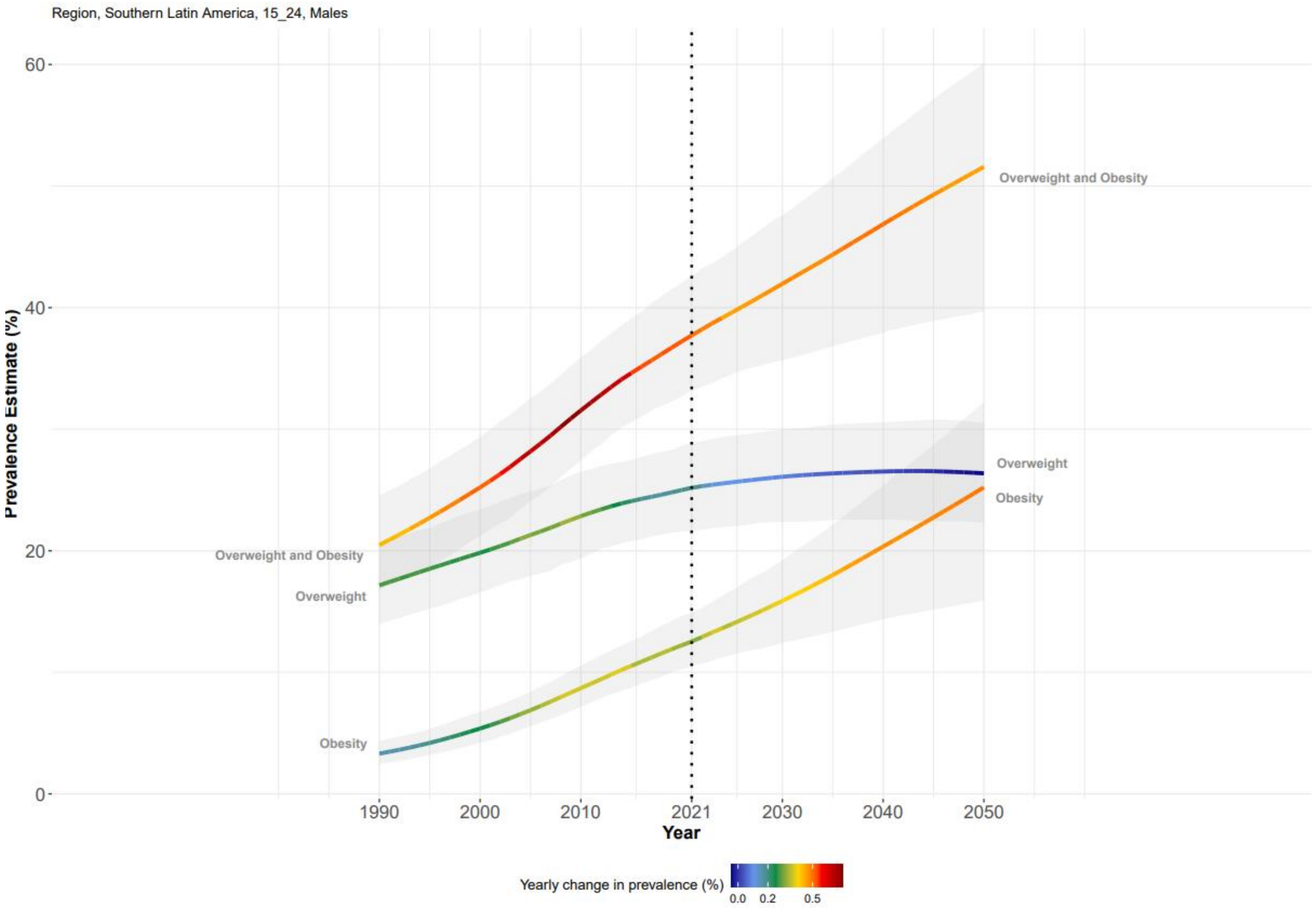
Region, Southern Latin America, 15_24, Males
Prevalence Estimate (%)
60
40
20
0
1990
2000
2010
2021
2030
2040
2050
Year
Overweight and Obesity
Overweight
Obesity
Overweight and Obesity
Overweight
Obesity
Yearly change in prevalence (%)
0.0
0.2
0.5

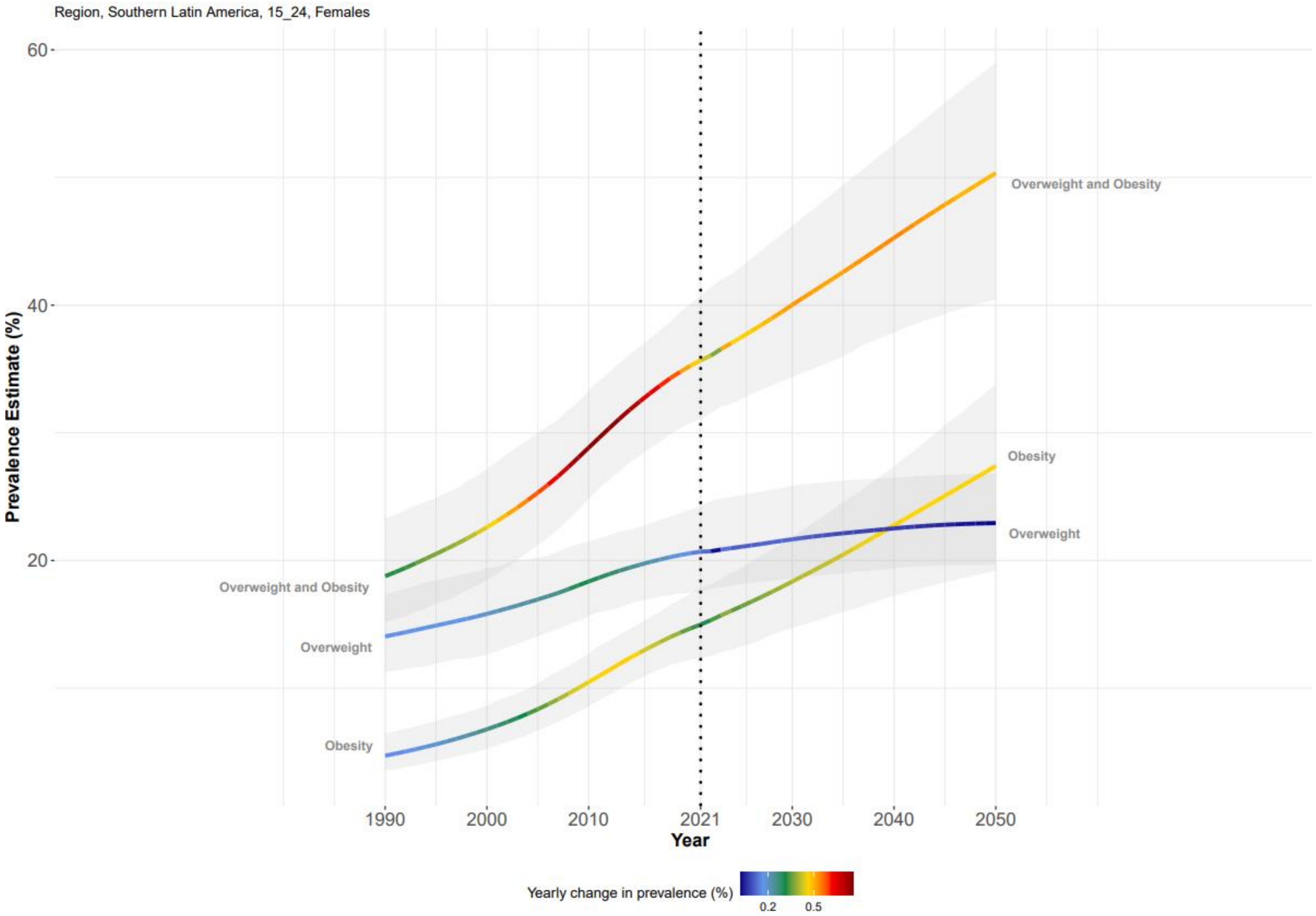
Region, Southern Latin America, 15_24, Females
Prevalence Estimate (%)
60
40
20
Overweight and Obesity
Overweight
Obesity
Overweight and Obesity
Obesity
Overweight
1990
2000
2010
2021
2030
2040
2050
Year
Yearly change in prevalence (%)
0.2
0.5

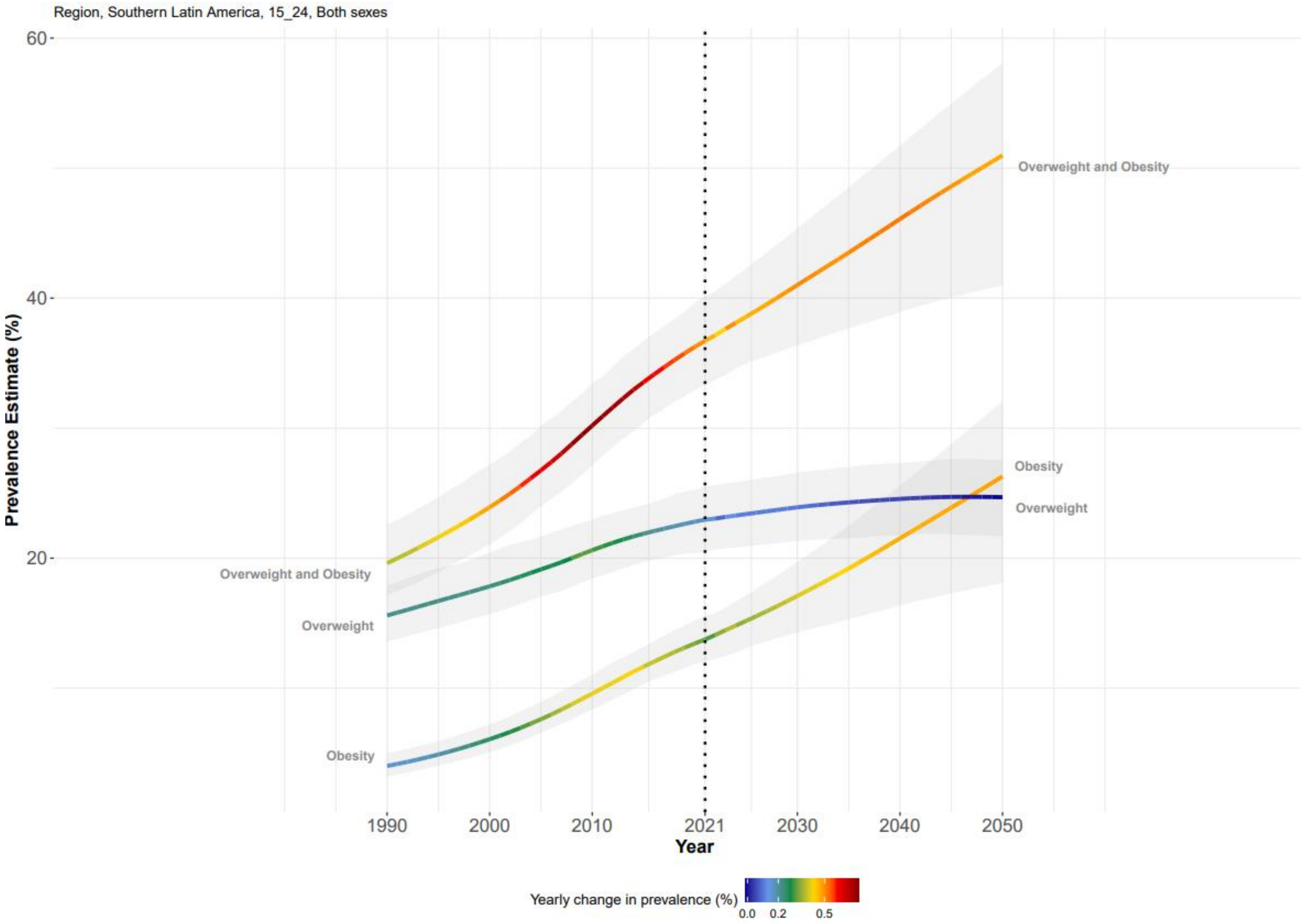

Region, Southern Latin America, 15_24, Both sexes
60
40
20
Prevalence Estimate (%)
Overweight and Obesity
Obesity
Overweight
Overweight and Obesity
Overweight
Obesity
1990
2000
2010
2021
2030
2040
2050
Year
Yearly change in prevalence (%)
0.0
0.2
0.5

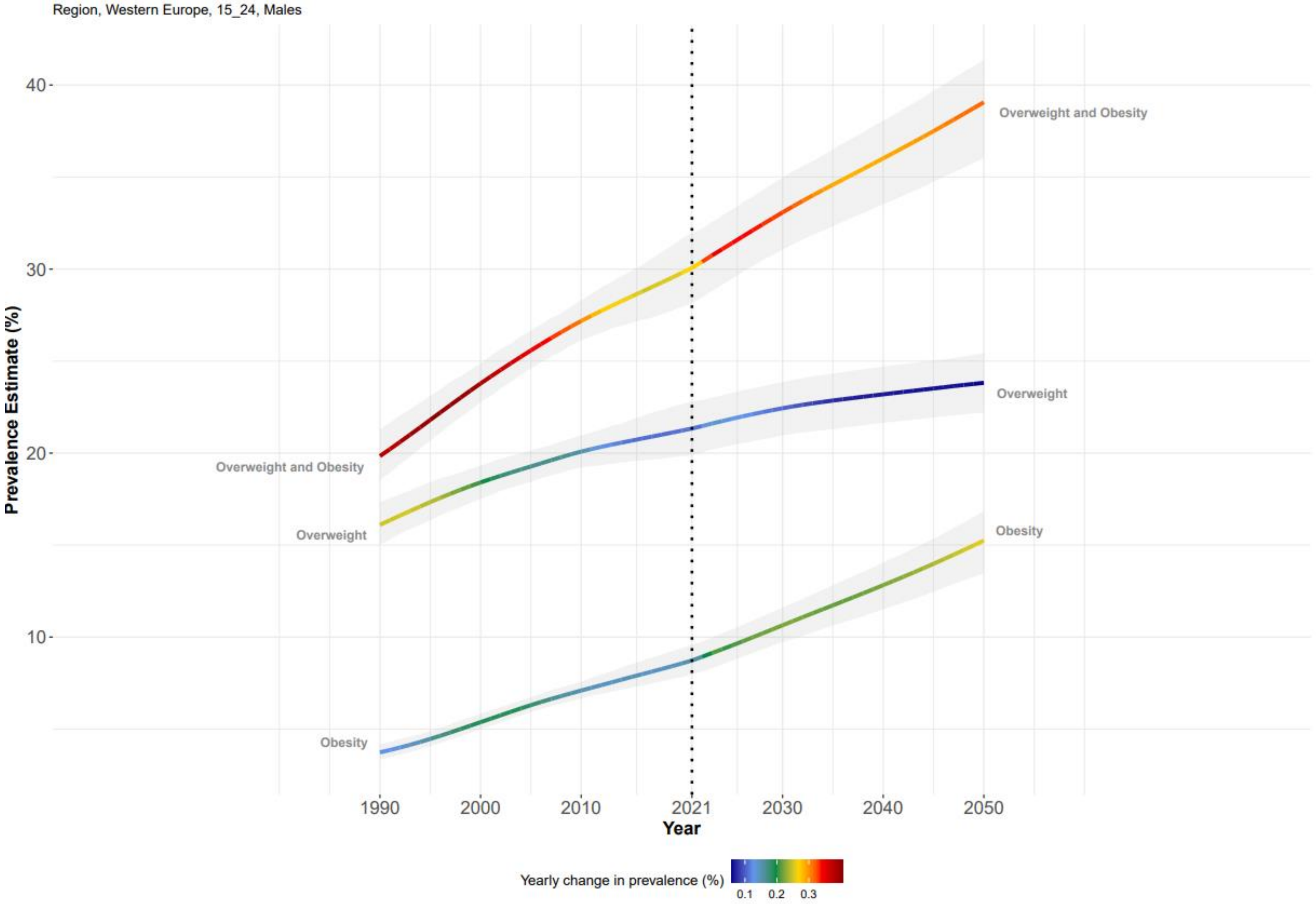

Region, Western Europe, 15_24, Males
Prevalence Estimate (%)
40
30
20
10
Overweight and Obesity
Overweight
Obesity
Overweight and Obesity
Overweight
Obesity
1990
2000
2010
2021
2030
2040
2050
Year
Yearly change in prevalence (%)
0.1
0.2
0.3

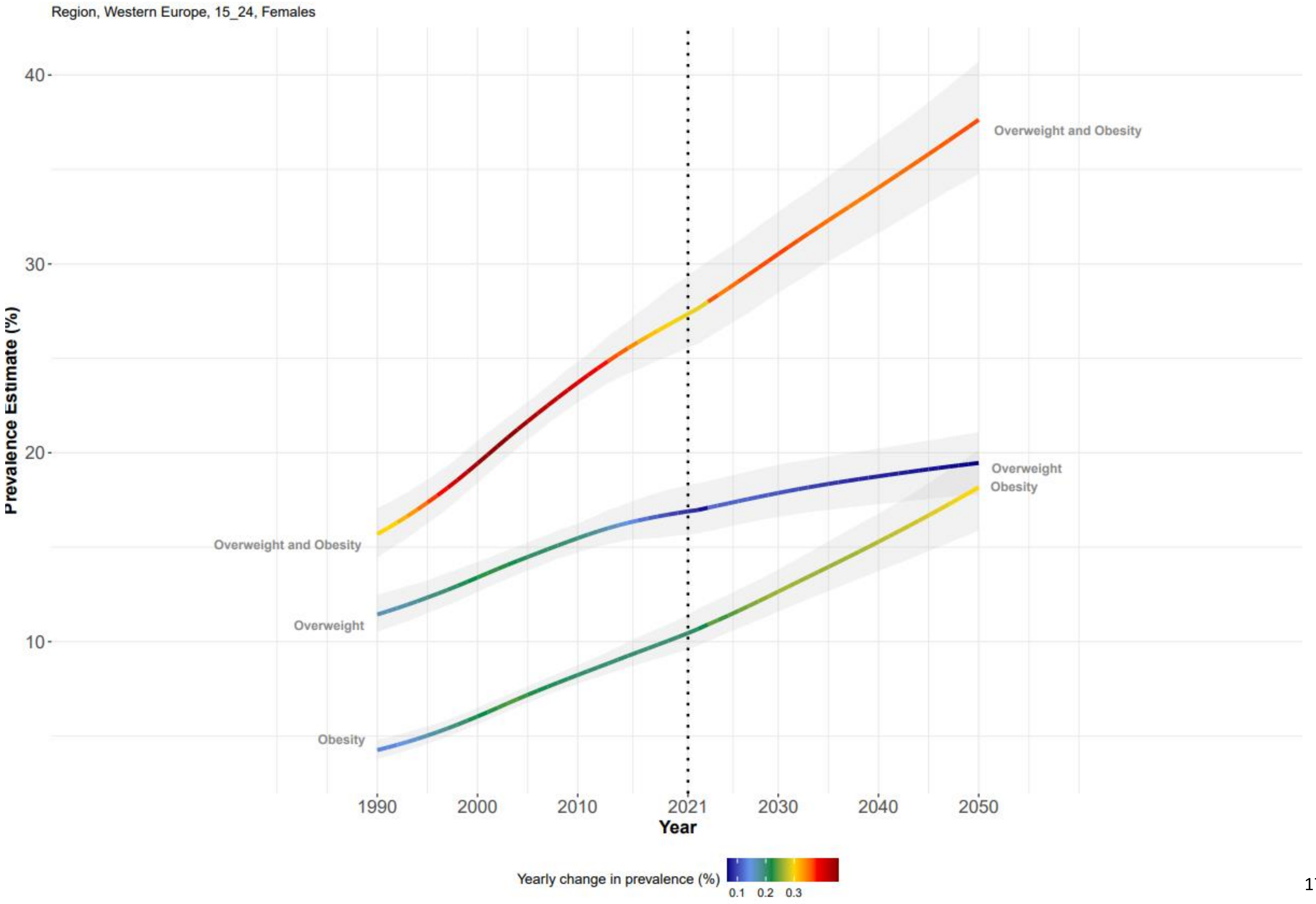

Region, Western Europe, 15_24, Females
Prevalence Estimate (%)
40
30
20
10
Overweight and Obesity
Overweight
Obesity
Overweight and Obesity
Overweight
Obesity
1990
2000
2010
2021
2030
2040
2050
Year
Yearly change in prevalence (%)
0.1
0.2
0.3

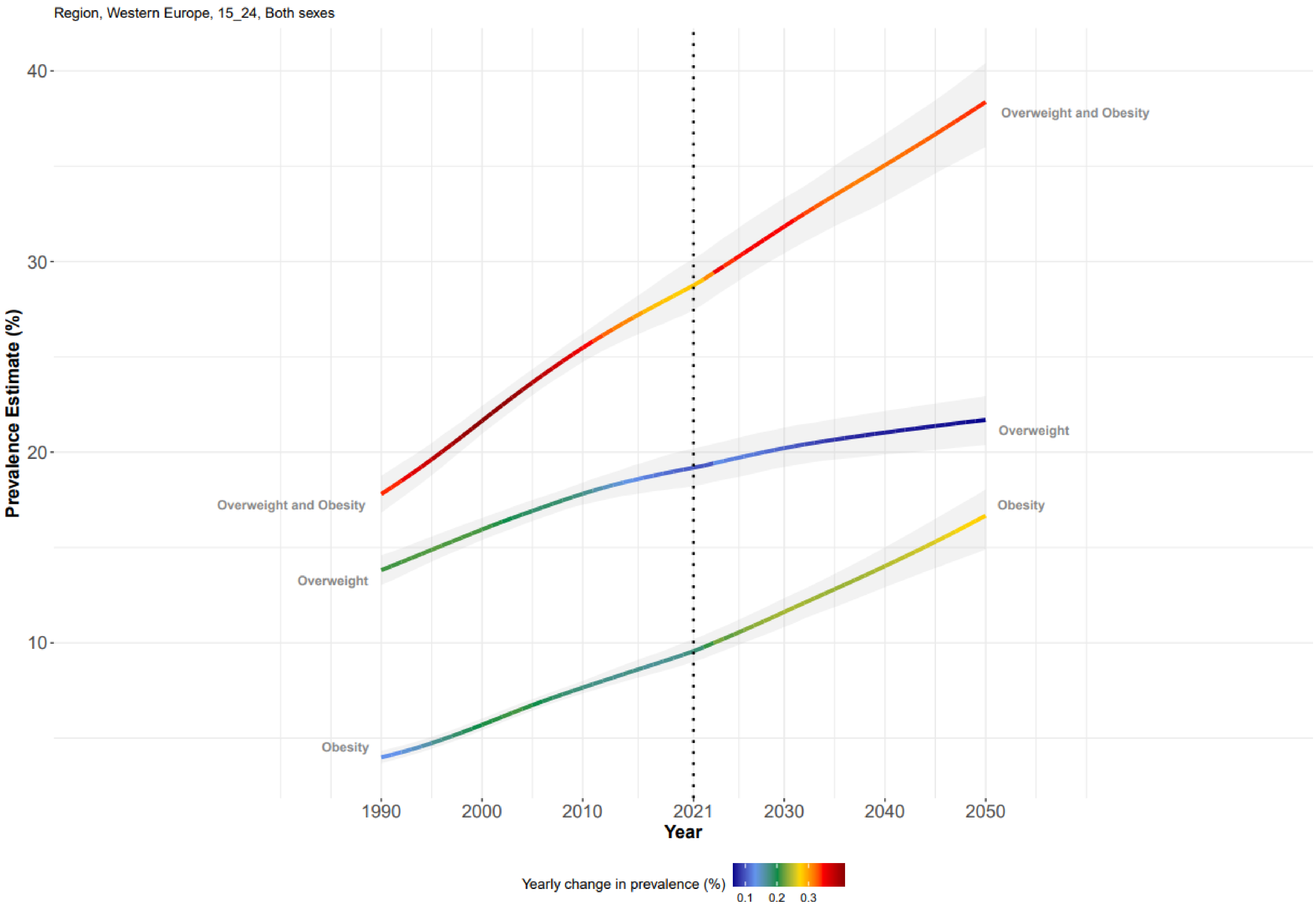
Region, Western Europe, 15_24, Both sexes
Prevalence Estimate (%)
40
30
20
10
Overweight and Obesity
Overweight
Obesity
Overweight and Obesity
Overweight
Obesity
1990
2000
2010
2021
2030
2040
2050
Year
Yearly change in prevalence (%)
0.1
0.2
0.3

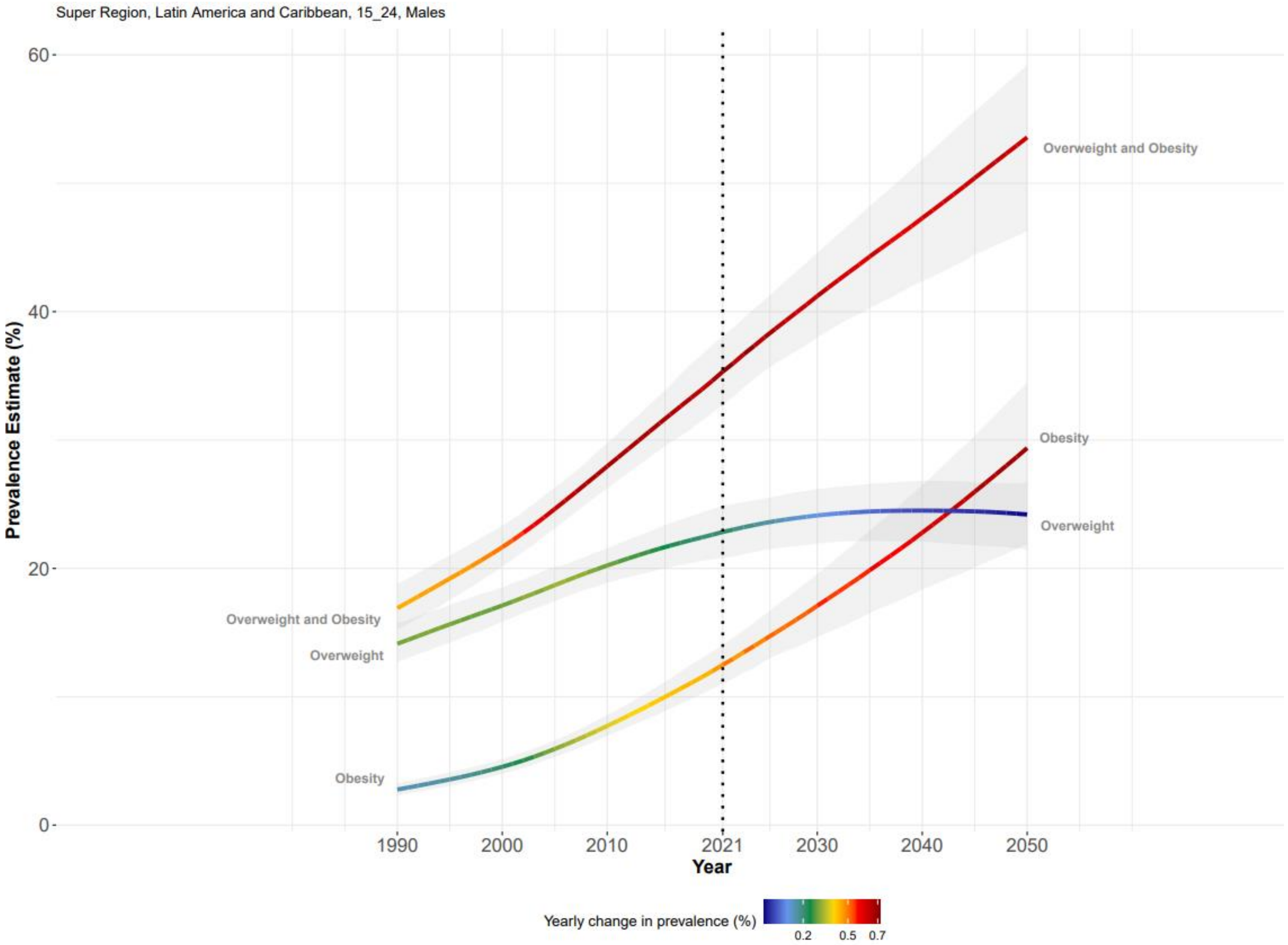
Super Region, Latin America and Caribbean, 15_24, Males
60
40
20
0
Prevalence Estimate (%)
Overweight and Obesity
Obesity
Overweight
Overweight and Obesity
Overweight
Obesity
1990
2000
2010
2021
2030
2040
2050
Year
Yearly change in prevalence (%)
0.2
0.5
0.7

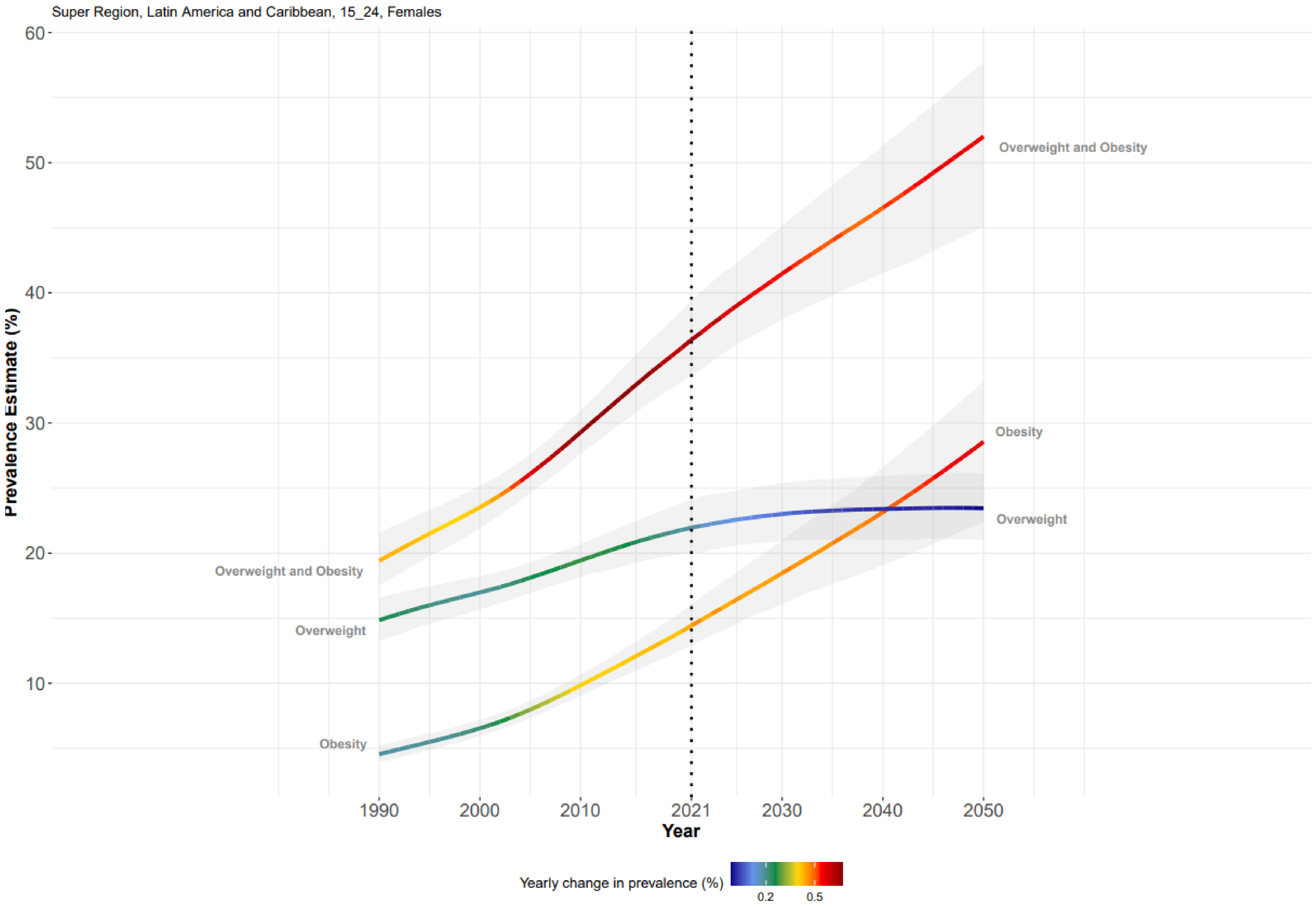
Super Region, Latin America and Caribbean, 15_24, Females
Prevalence Estimate (%)
60
50
40
30
20
10
Overweight and Obesity
Overweight
Obesity
Overweight and Obesity
Obesity
Overweight
1990
2000
2010
2021
2030
2040
2050
Year
Yearly change in prevalence (%)
0.2
0.5

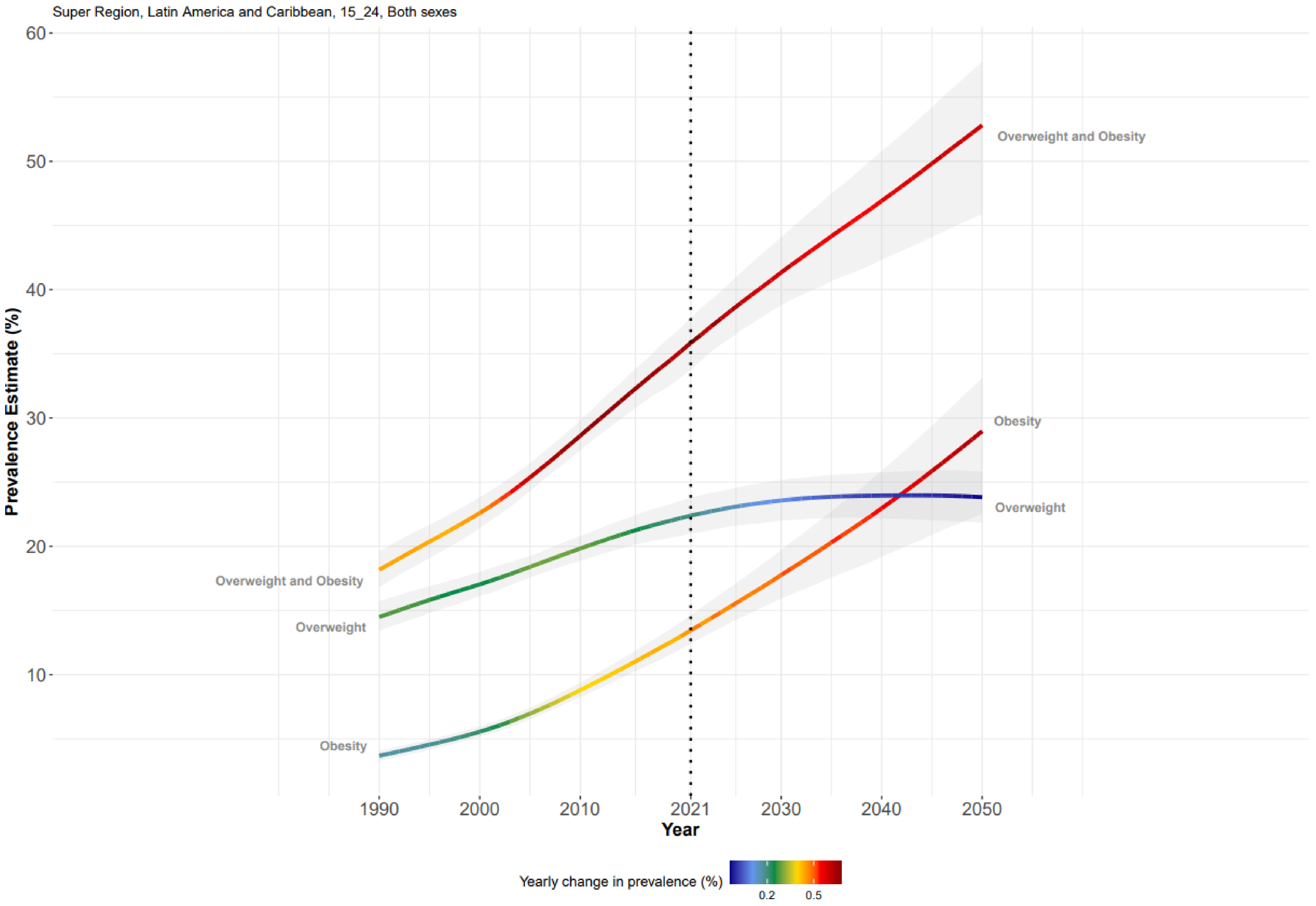

Super Region, Latin America and Caribbean, 15_24, Both sexes
Prevalence Estimate (%)
60
50
40
30
20
10
1990
2000
2010
2021
2030
2040
2050
Year
Overweight and Obesity
Overweight
Obesity
Overweight and Obesity
Obesity
Overweight
Yearly change in prevalence (%)
0.2
0.5

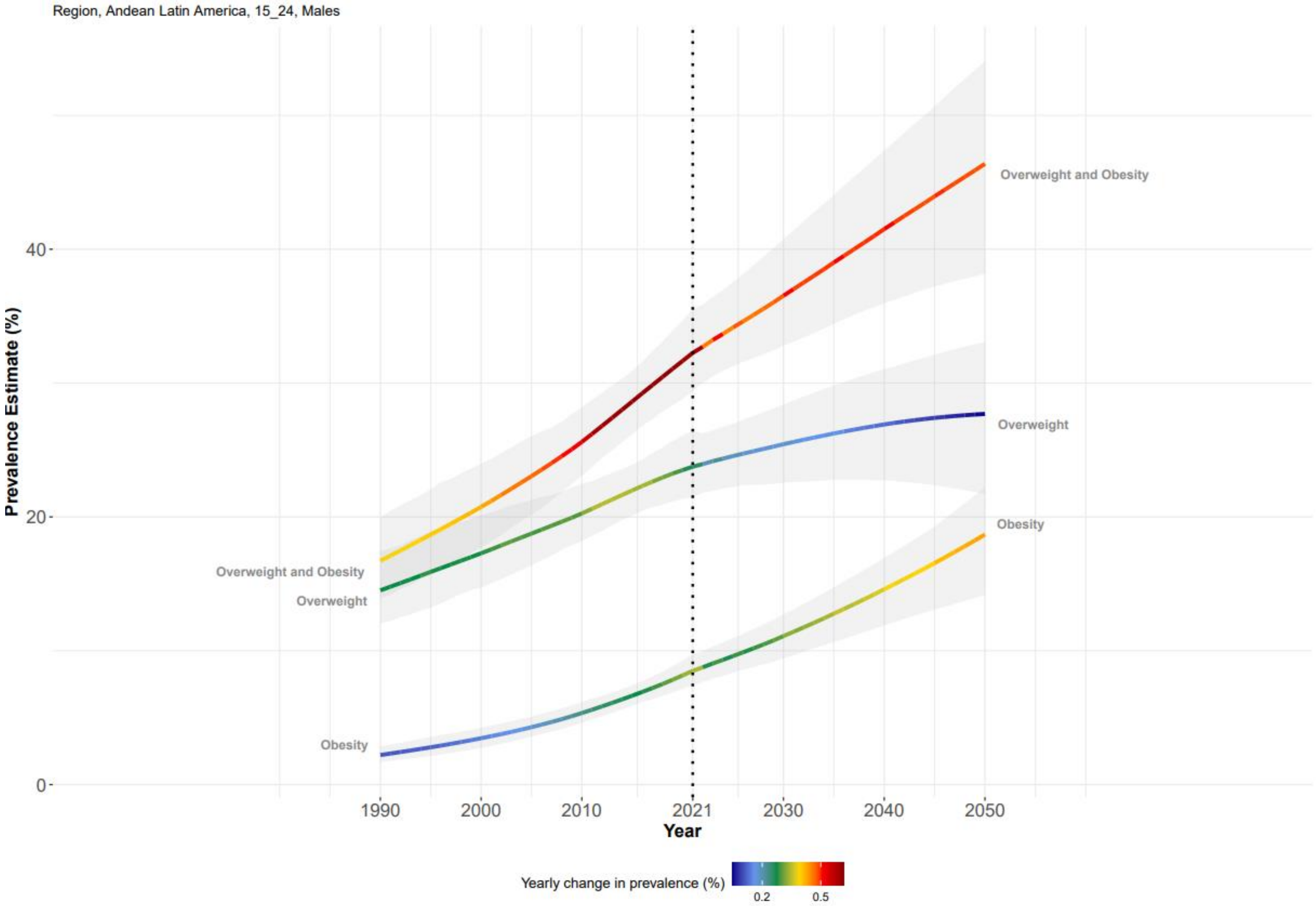

Region, Andean Latin America, 15_24, Males
Prevalence Estimate (%)
40
20
0
1990
2000
2010
2021
2030
2040
2050
Year
Overweight and Obesity
Overweight
Obesity
Overweight and Obesity
Overweight
Obesity
Yearly change in prevalence (%)
0.2
0.5

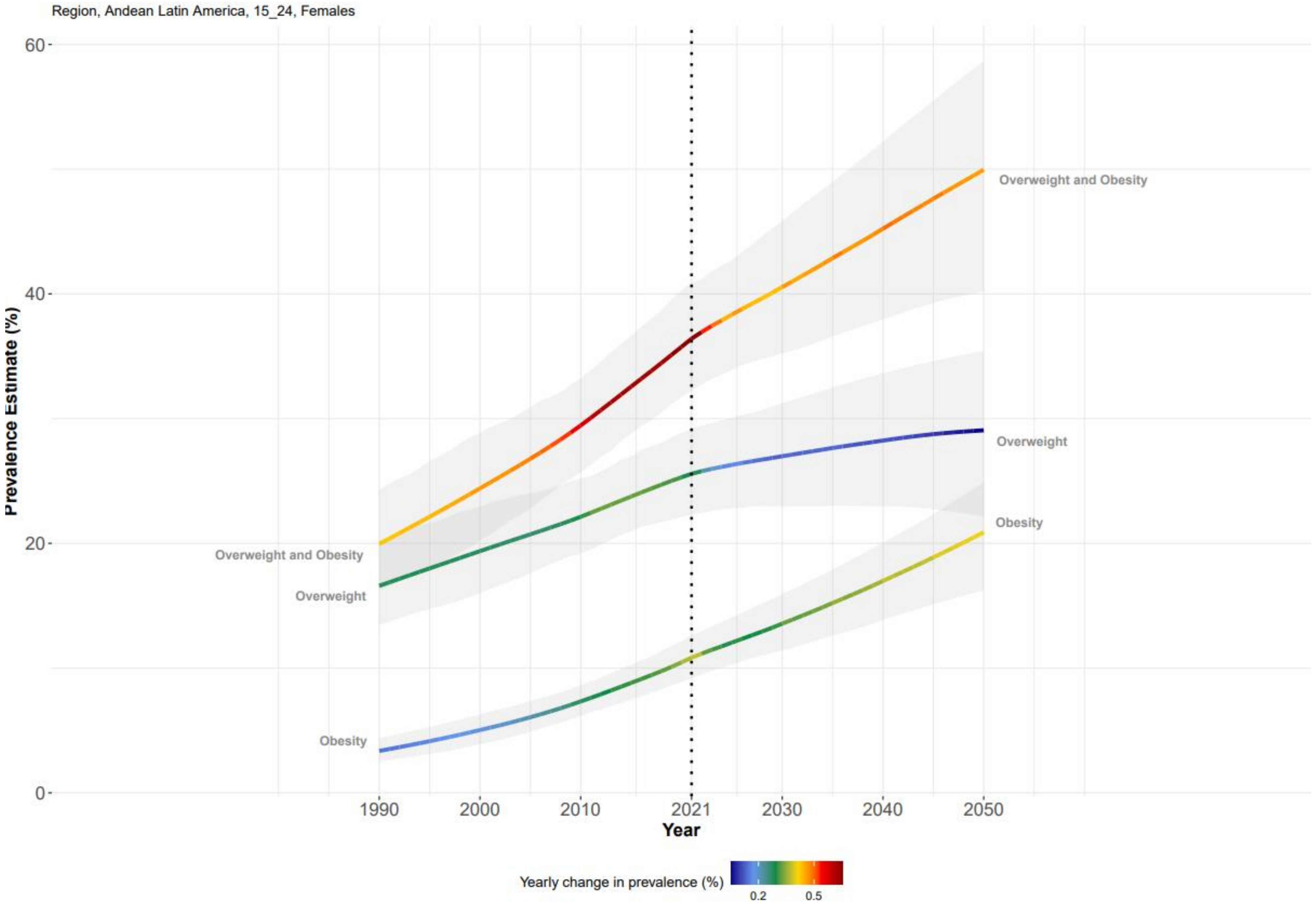
Region, Andean Latin America, 15_24, Females
Prevalence Estimate (%)
60
40
20
0
1990
2000
2010
2021
2030
2040
2050
Year
Overweight and Obesity
Overweight
Obesity
Overweight and Obesity
Overweight
Obesity
Yearly change in prevalence (%)
0.2
0.5

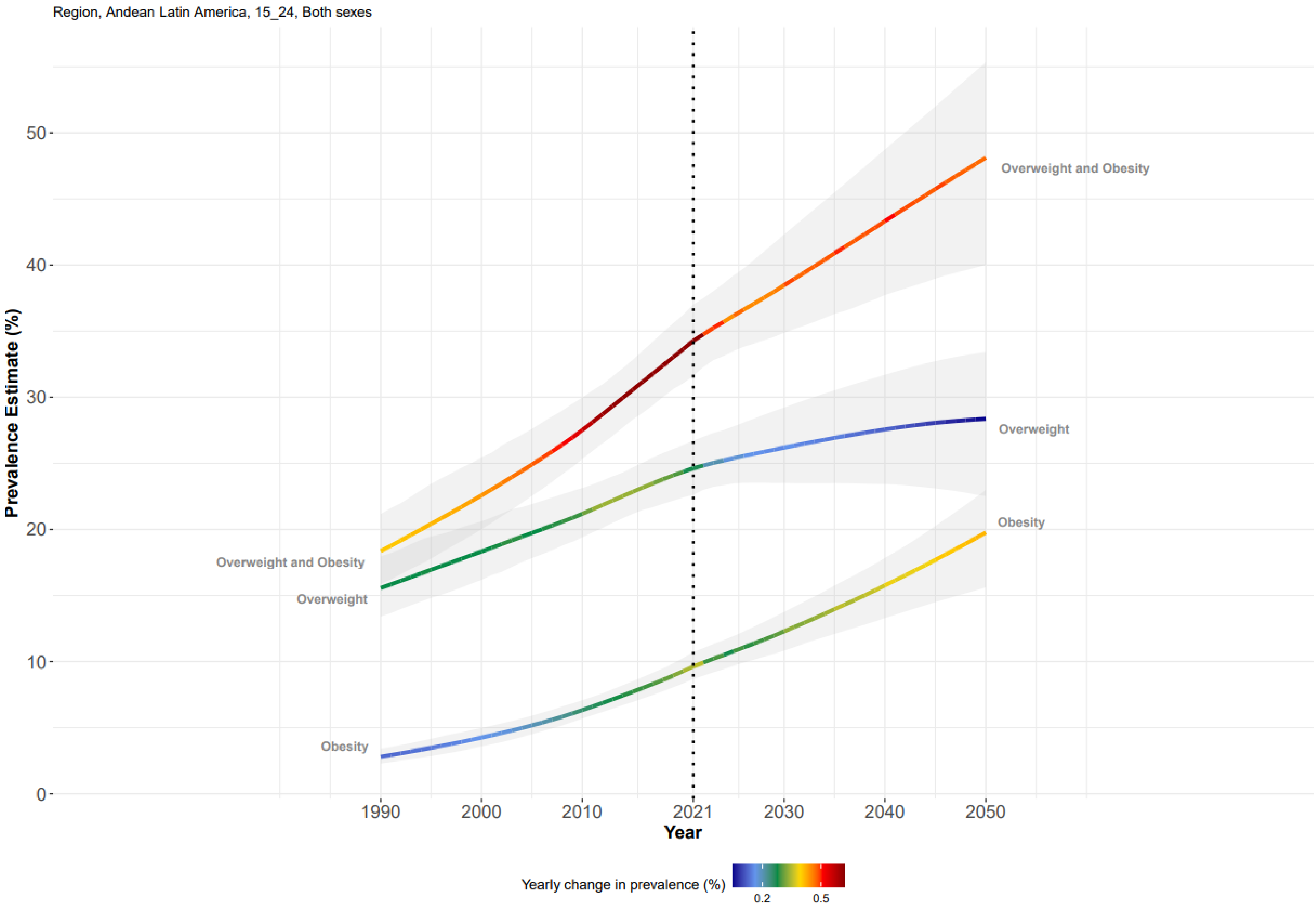
Region, Andean Latin America, 15_24, Both sexes
Prevalence Estimate (%)
0
10
20
30
40
50
1990
2000
2010
2021
2030
2040
2050
Year
Overweight and Obesity
Overweight
Obesity
Overweight and Obesity
Overweight
Obesity
Yearly change in prevalence (%)
0.2
0.5

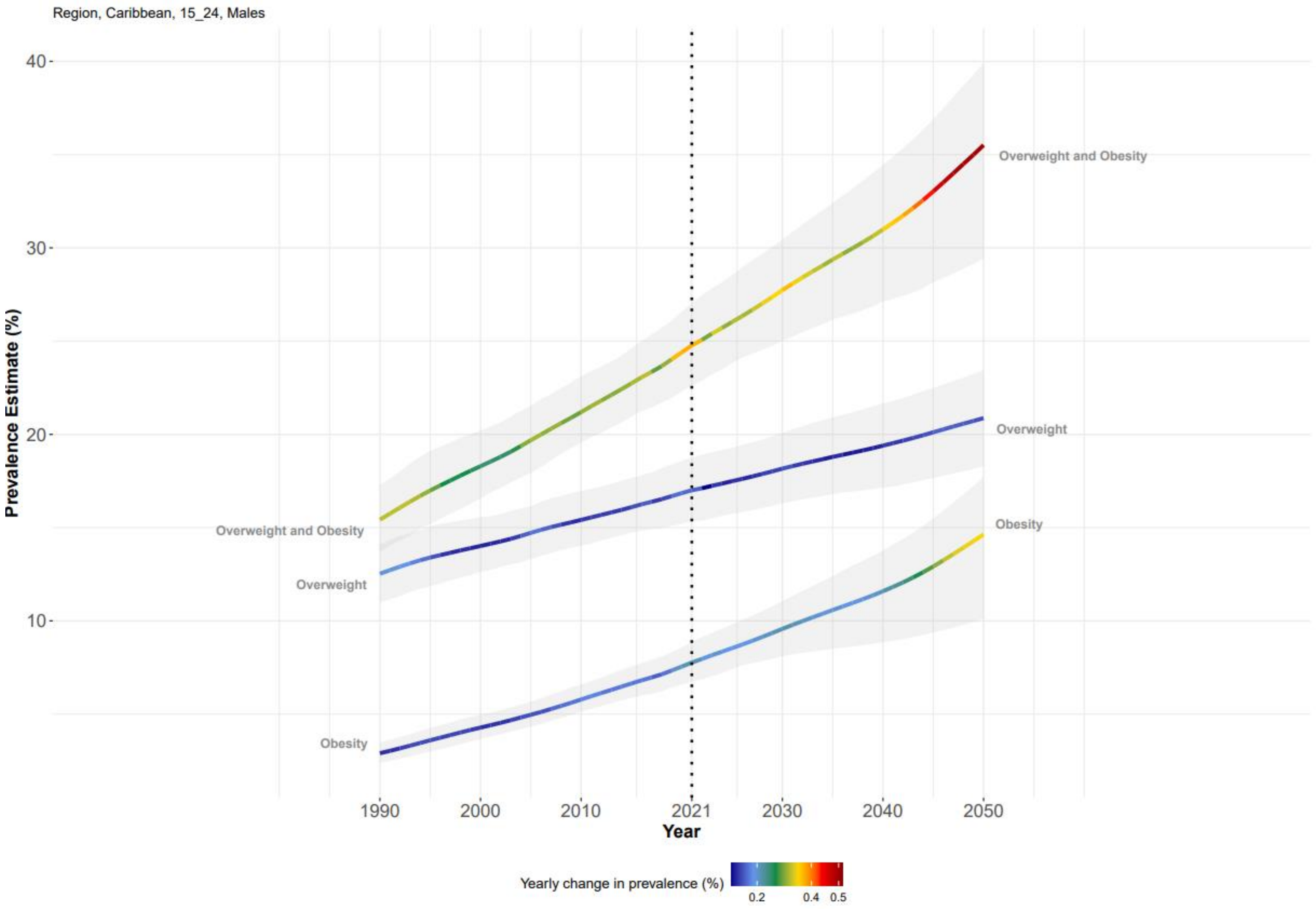
Region, Caribbean, 15_24, Males
Prevalence Estimate (%)
40
30
20
10
Overweight and Obesity
Overweight
Obesity
Overweight and Obesity
Overweight
Obesity
1990
2000
2010
2021
2030
2040
2050
Year
Yearly change in prevalence (%)
0.2
0.4
0.5

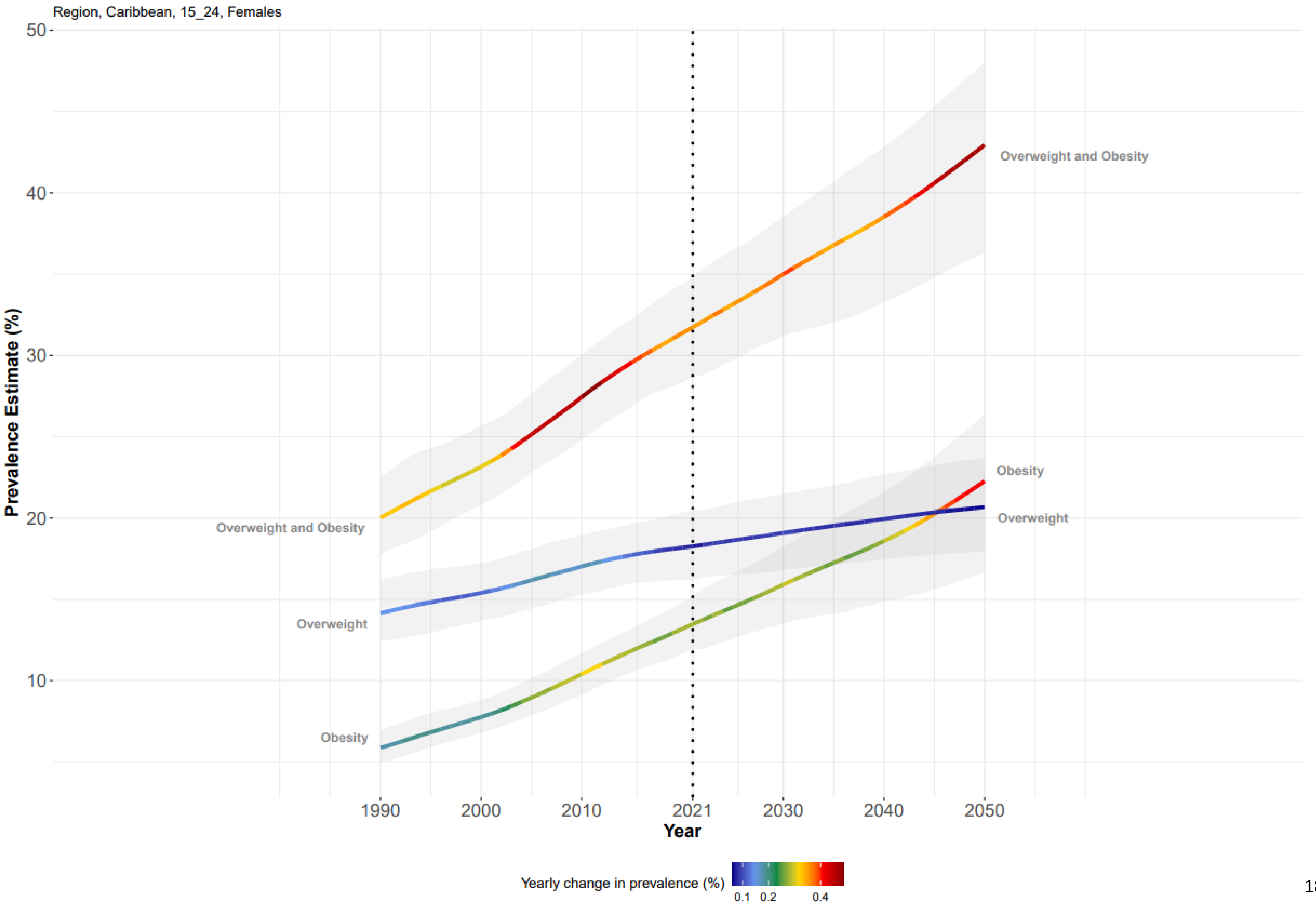
Region, Caribbean, 15_24, Females
Prevalence Estimate (%)
50
40
30
20
10
Overweight and Obesity
Overweight
Obesity
Overweight and Obesity
Obesity
Overweight
1990
2000
2010
2021
2030
2040
2050
Year
Yearly change in prevalence (%)
0.1
0.2
0.4

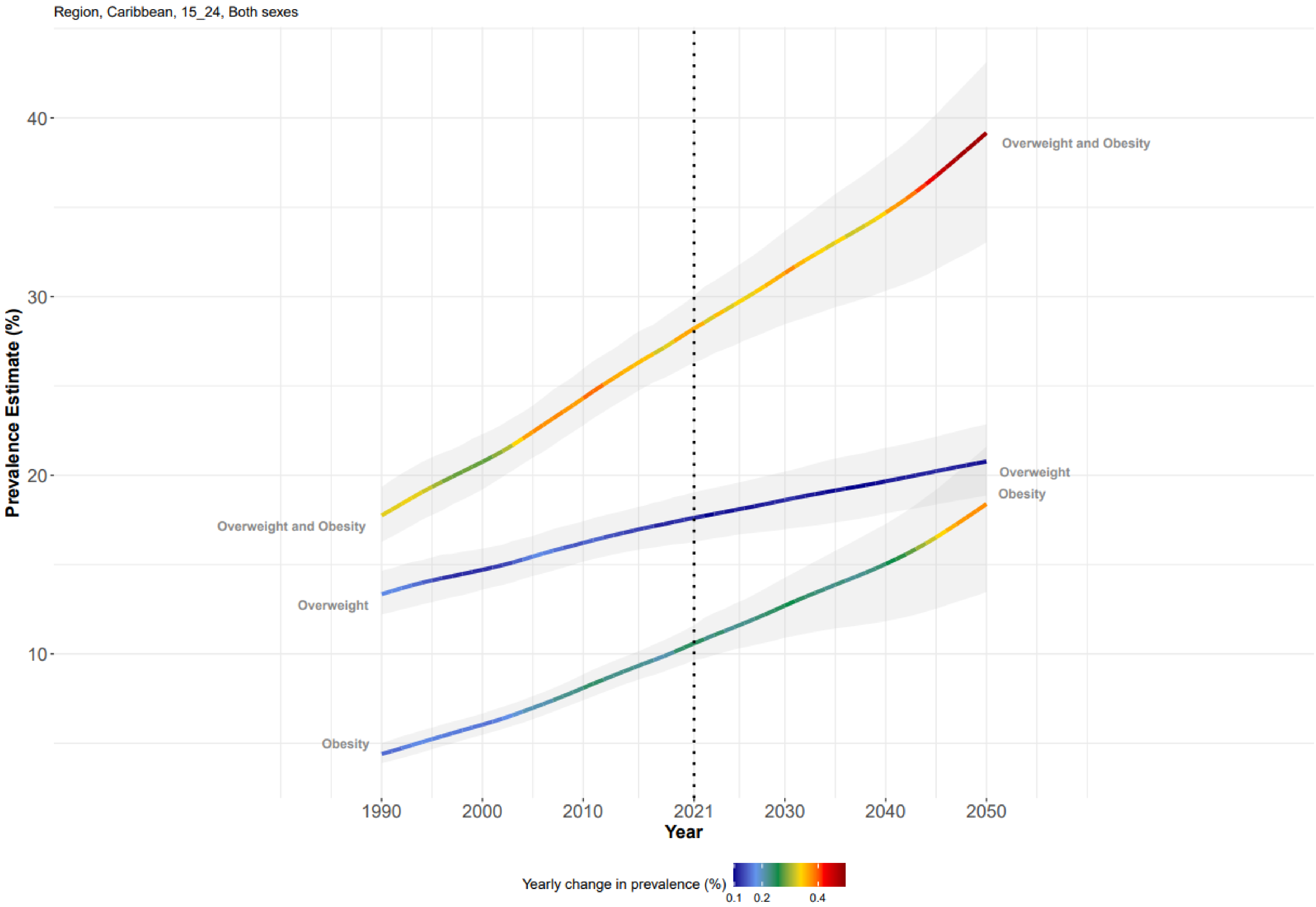
Region, Caribbean, 15_24, Both sexes
Prevalence Estimate (%)
40
30
20
10
Overweight and Obesity
Overweight
Obesity
Overweight and Obesity
Overweight
Obesity
1990
2000
2010
2021
2030
2040
2050
Year
Yearly change in prevalence (%)
0.1
0.2
0.4

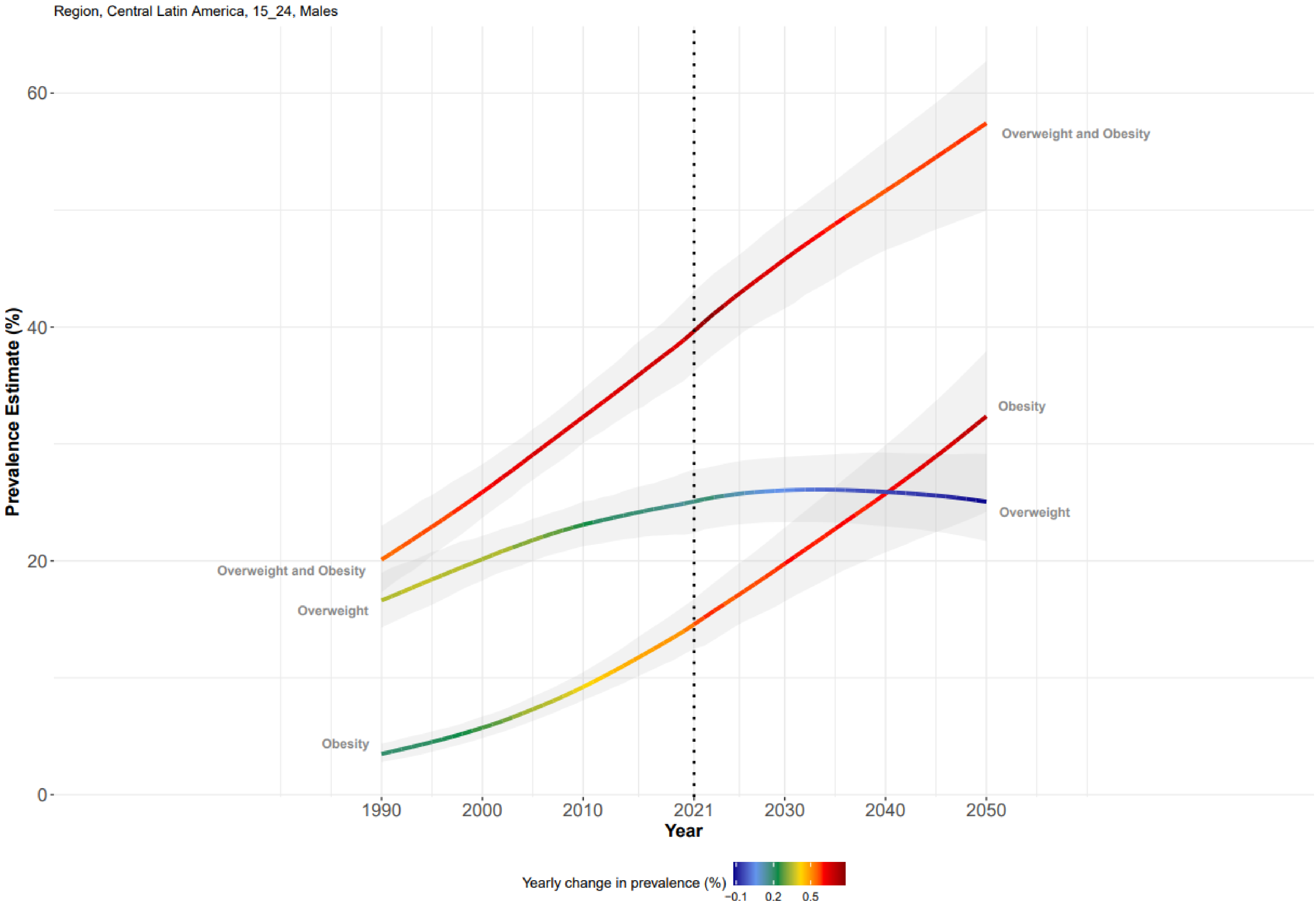
Region, Central Latin America, 15_24, Males
Prevalence Estimate (%)
60
40
20
0
1990
2000
2010
2021
2030
2040
2050
Year
Overweight and Obesity
Overweight
Obesity
Overweight and Obesity
Obesity
Overweight
Yearly change in prevalence (%)
−0.1
0.2
0.5

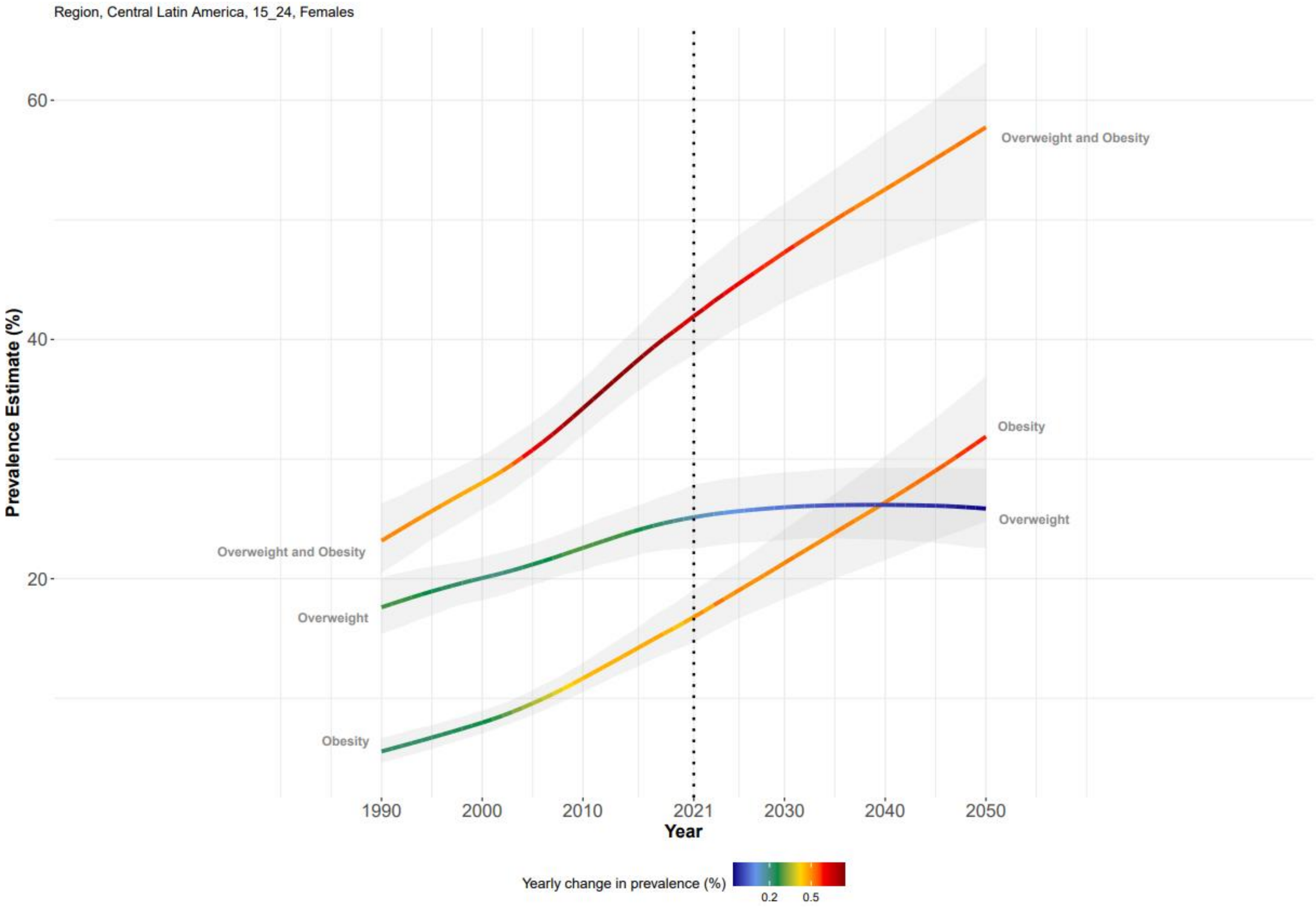
Region, Central Latin America, 15_24, Females
Prevalence Estimate (%)
60
40
20
Overweight and Obesity
Obesity
Overweight
Overweight and Obesity
Overweight
Obesity
1990
2000
2010
2021
2030
2040
2050
Year
Yearly change in prevalence (%)
0.2
0.5

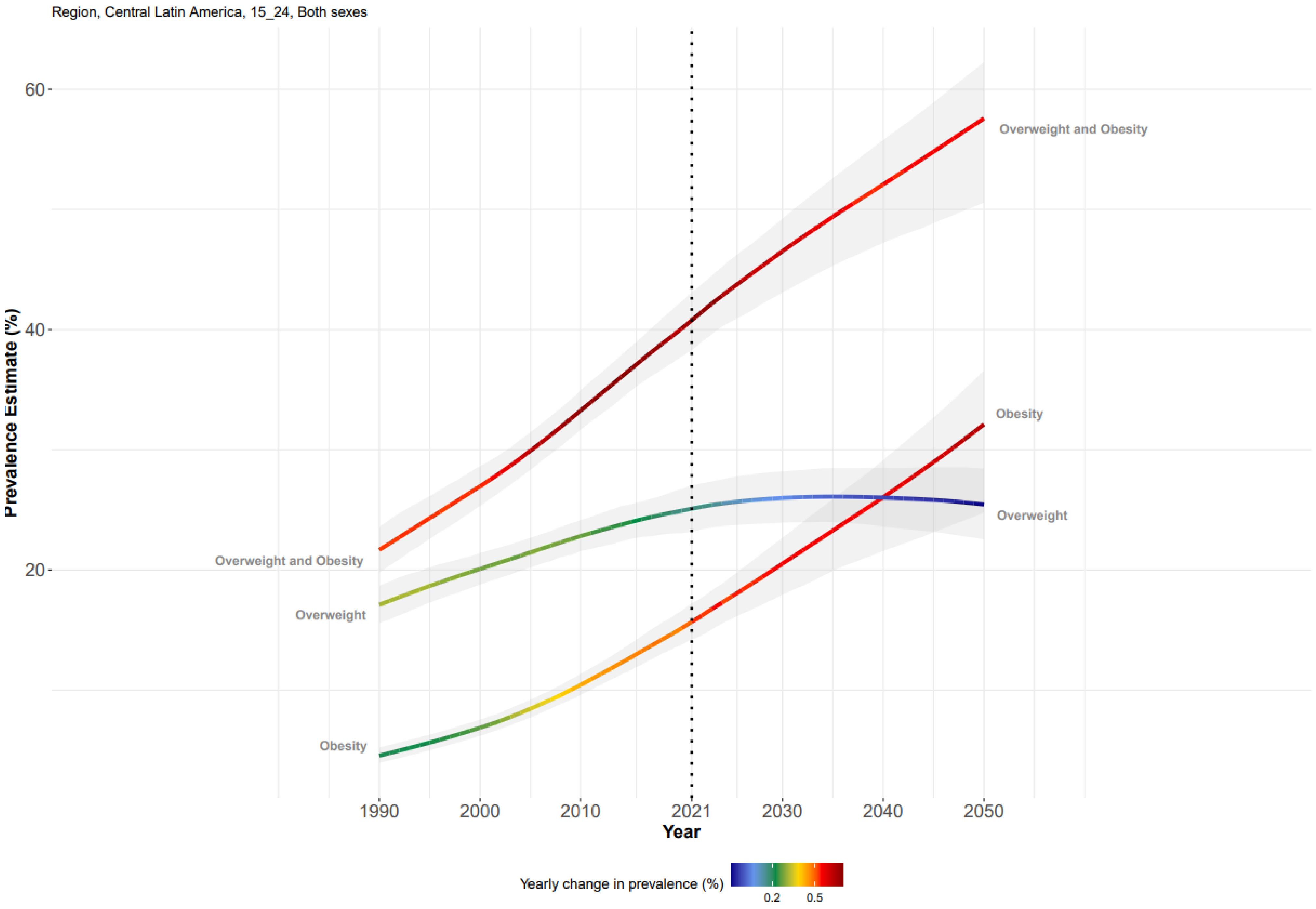

Region, Central Latin America, 15_24, Both sexes
Prevalence Estimate (%)
60
40
20
Overweight and Obesity
Obesity
Overweight
Overweight and Obesity
Overweight
Obesity
1990
2000
2010
2021
2030
2040
2050
Year
Yearly change in prevalence (%)
0.2
0.5

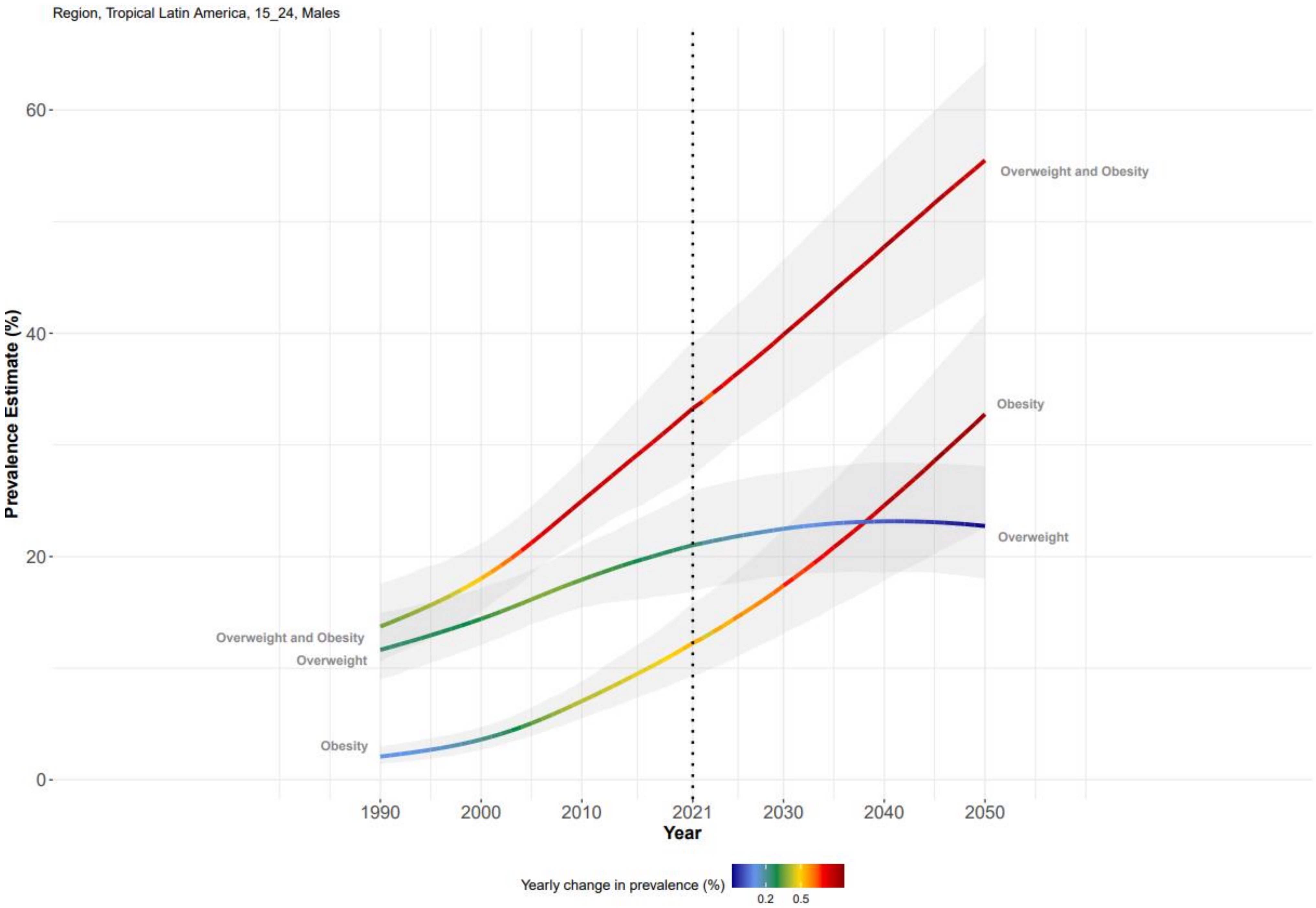

Region, Tropical Latin America, 15_24, Males
Prevalence Estimate (%)
60
40
20
0
1990
2000
2010
2021
2030
2040
2050
Year
Overweight and Obesity
Obesity
Overweight
Overweight and Obesity
Overweight
Obesity
Yearly change in prevalence (%)
0.2
0.5

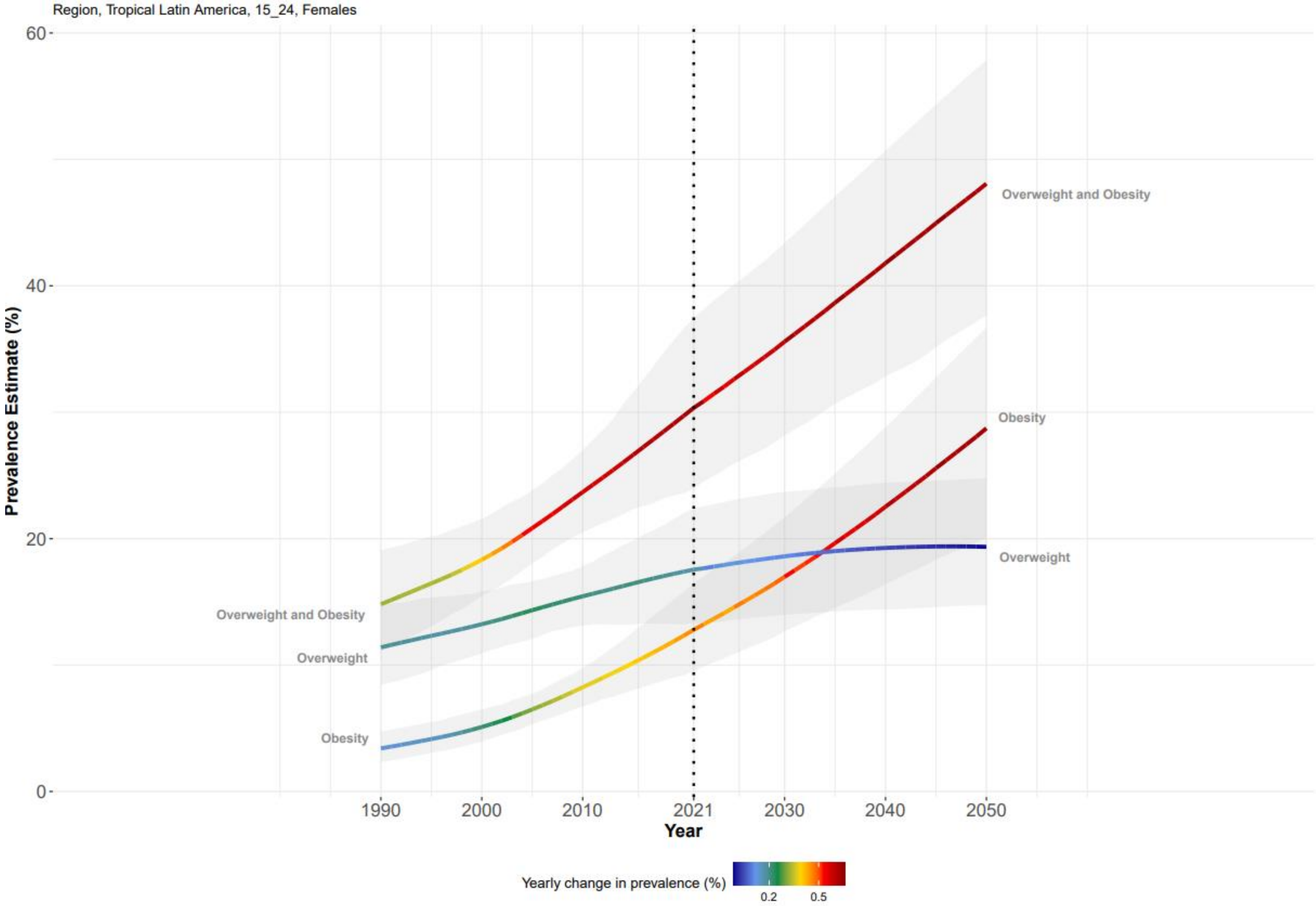

Region, Tropical Latin America, 15_24, Females
60
40
20
0
Prevalence Estimate (%)
Overweight and Obesity
Obesity
Overweight
Overweight and Obesity
Overweight
Obesity
1990
2000
2010
2021
2030
2040
2050
Year
Yearly change in prevalence (%)
0.2
0.5

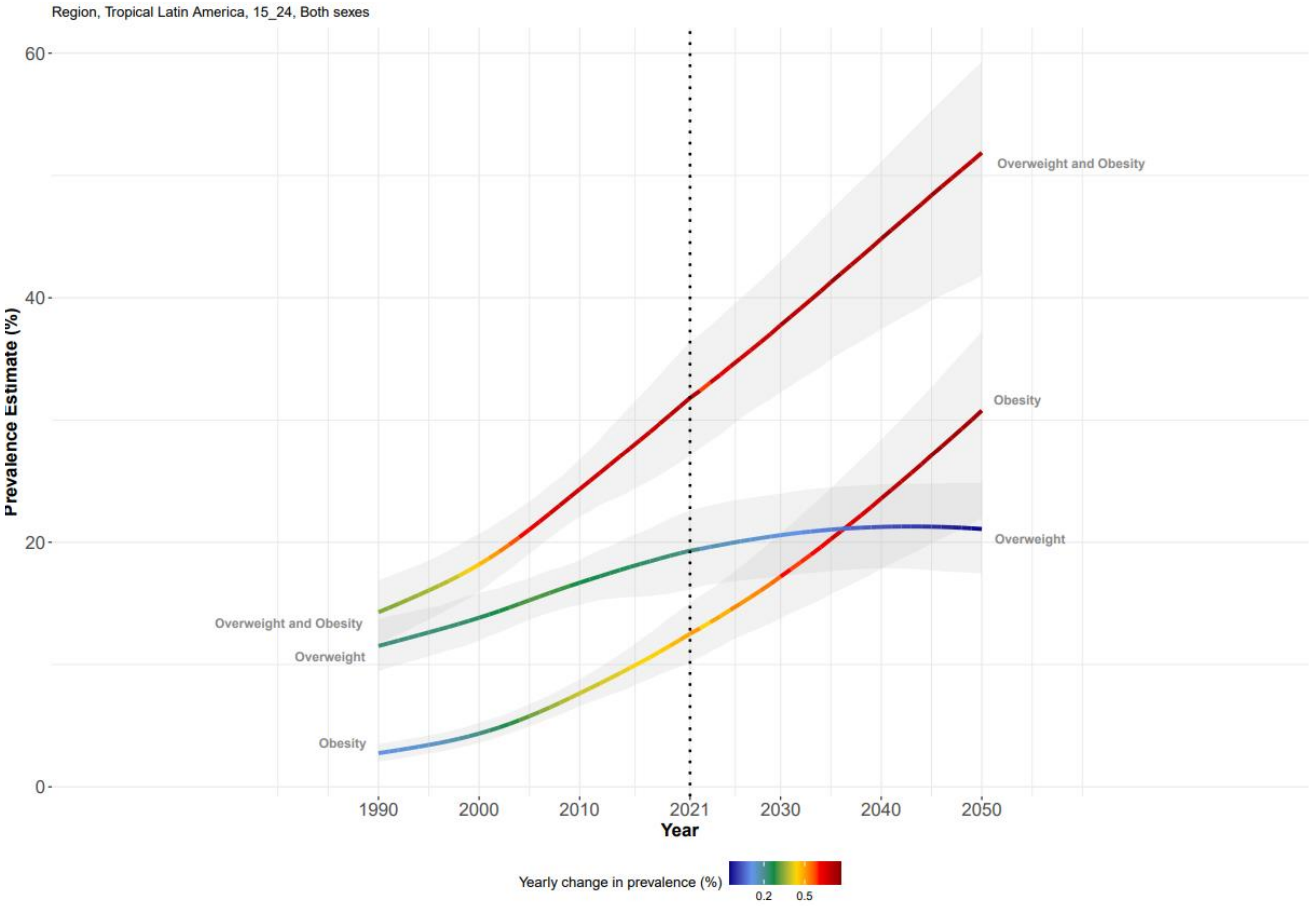

Region, Tropical Latin America, 15_24, Both sexes
Prevalence Estimate (%)
60
40
20
0
Overweight and Obesity
Obesity
Overweight
Overweight and Obesity
Overweight
Obesity
1990
2000
2010
2021
2030
2040
2050
Year
Yearly change in prevalence (%)
0.2
0.5

Super Region, North Africa and Middle East, 15_24, Males

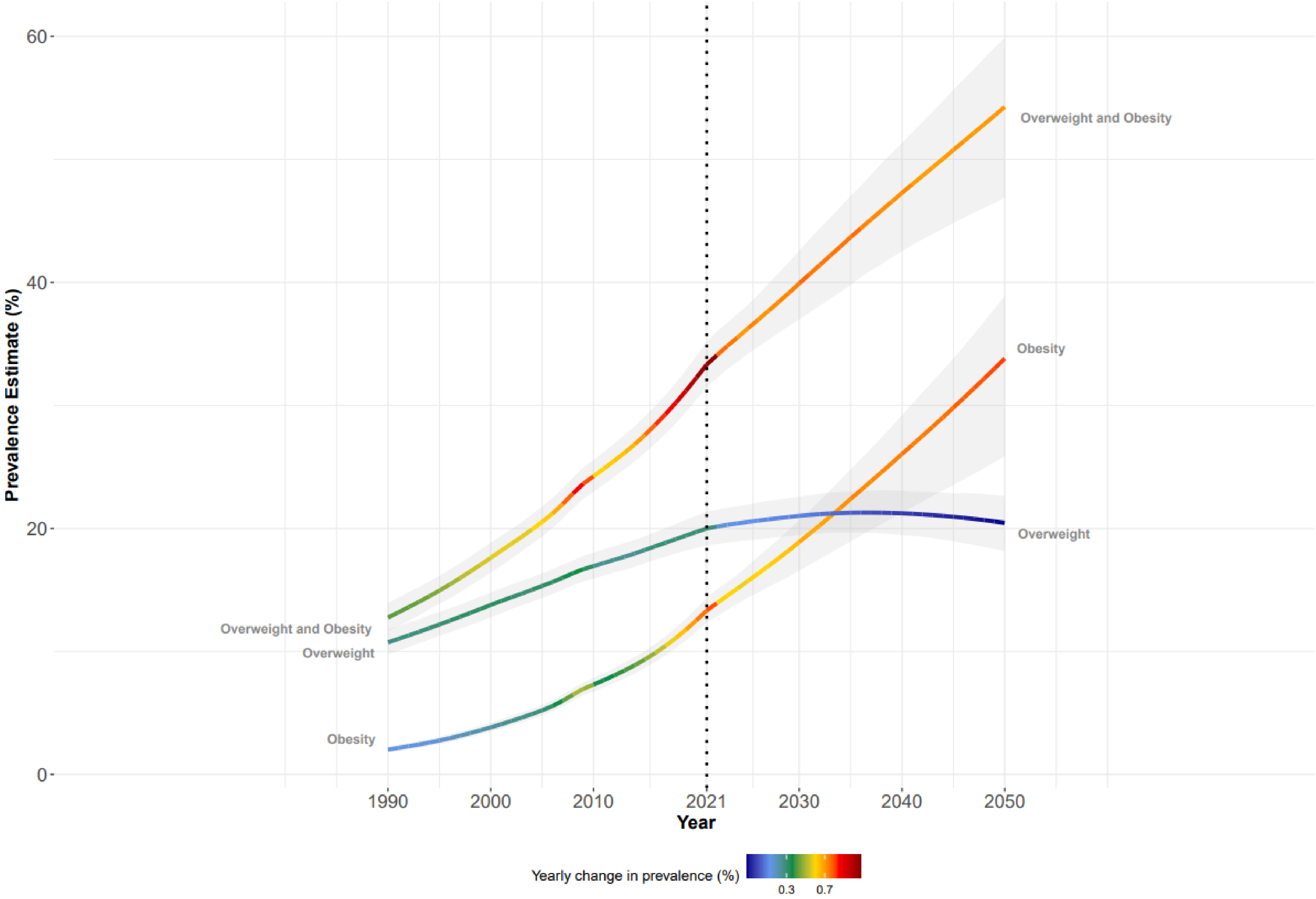

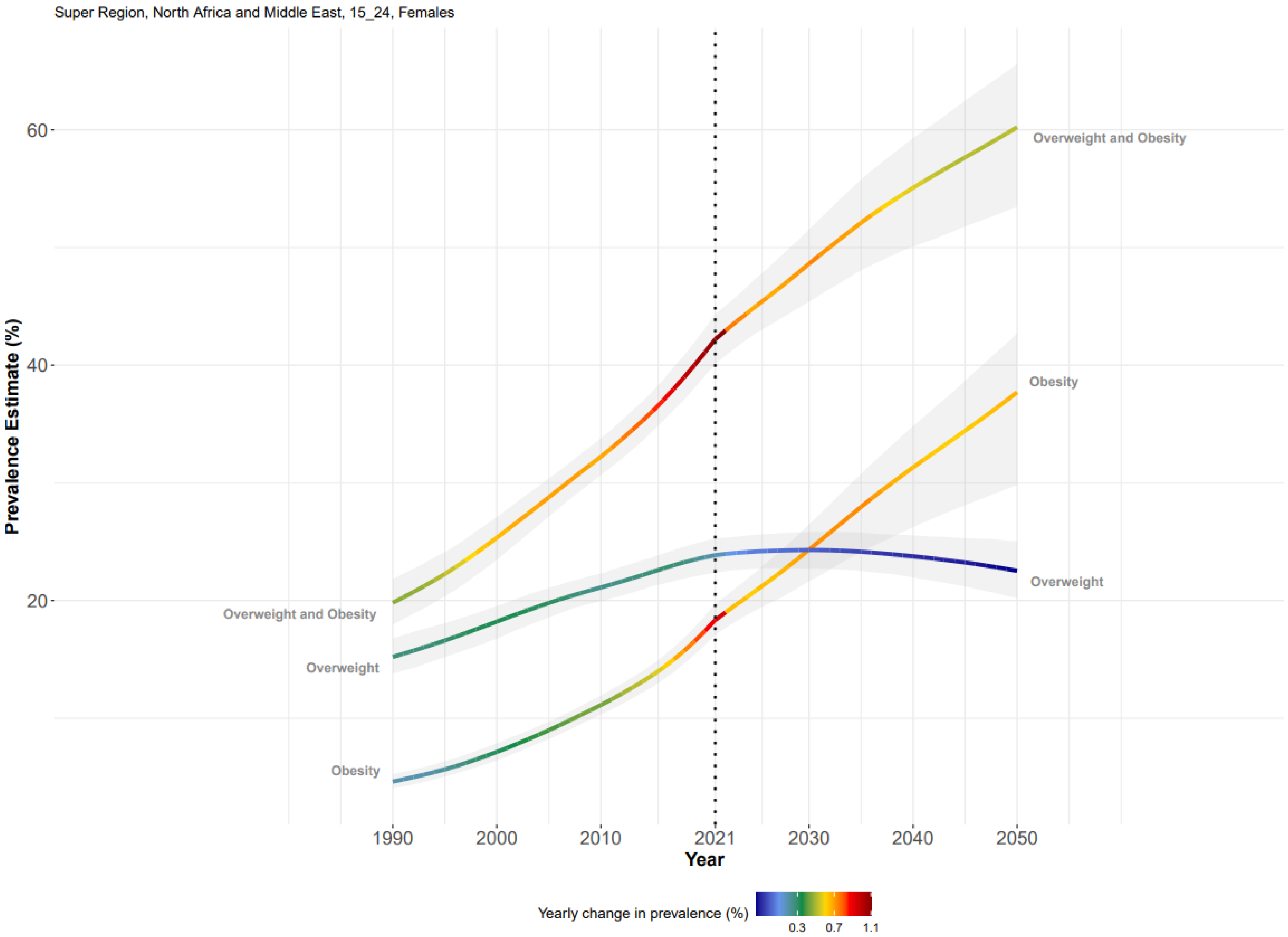

Super Region, North Africa and Middle East, 15_24, Females
Prevalence Estimate (%)
60
40
20
Overweight and Obesity
Overweight
Obesity
Overweight and Obesity
Obesity
Overweight
1990
2000
2010
2021
2030
2040
2050
Year
Yearly change in prevalence (%)
0.3
0.7
1.1

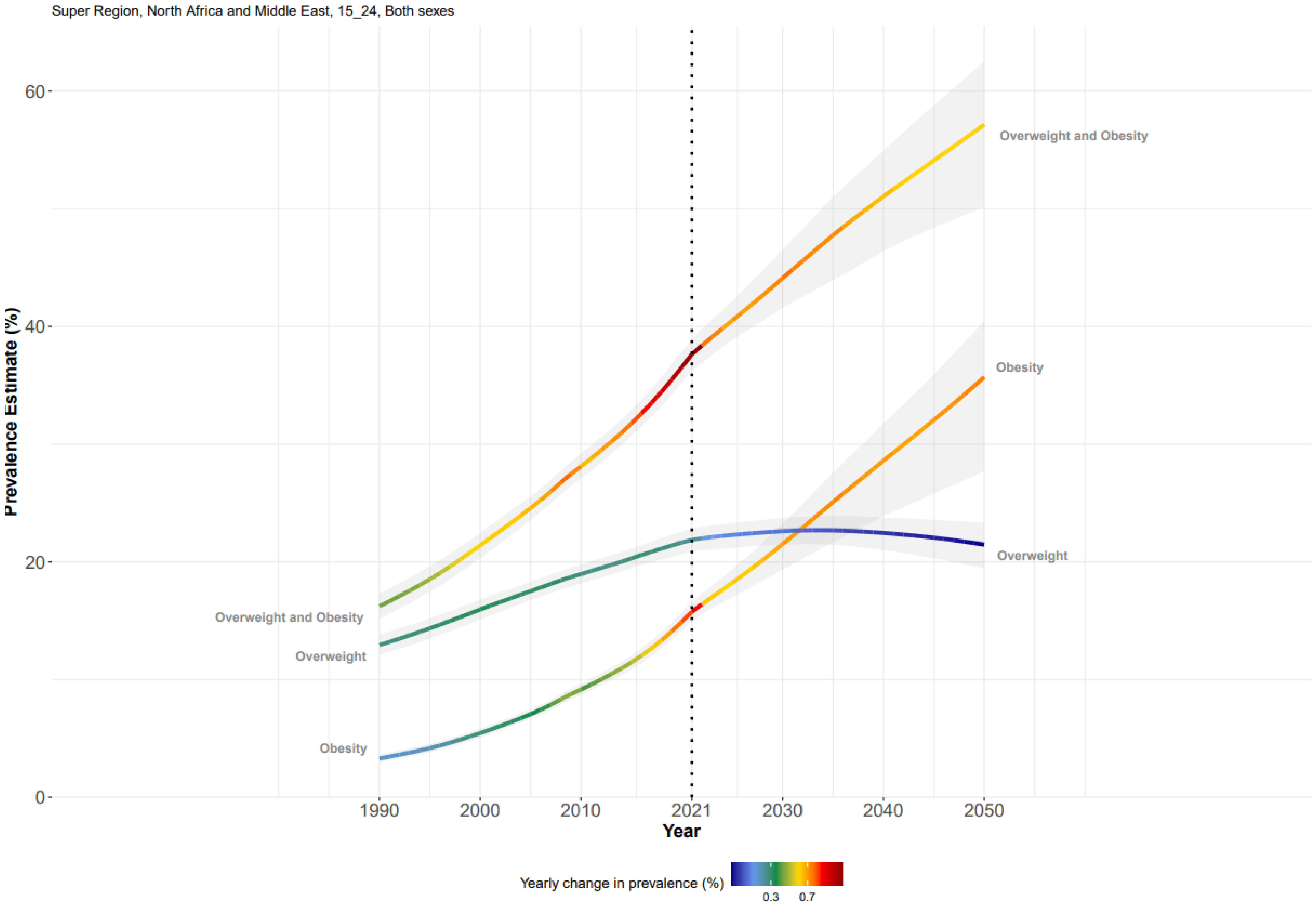

Super Region, North Africa and Middle East, 15_24, Both sexes
Prevalence Estimate (%)
60
40
20
0
1990
2000
2010
2021
2030
2040
2050
Year
Overweight and Obesity
Obesity
Overweight
Overweight and Obesity
Overweight
Obesity
Yearly change in prevalence (%)
0.3
0.7

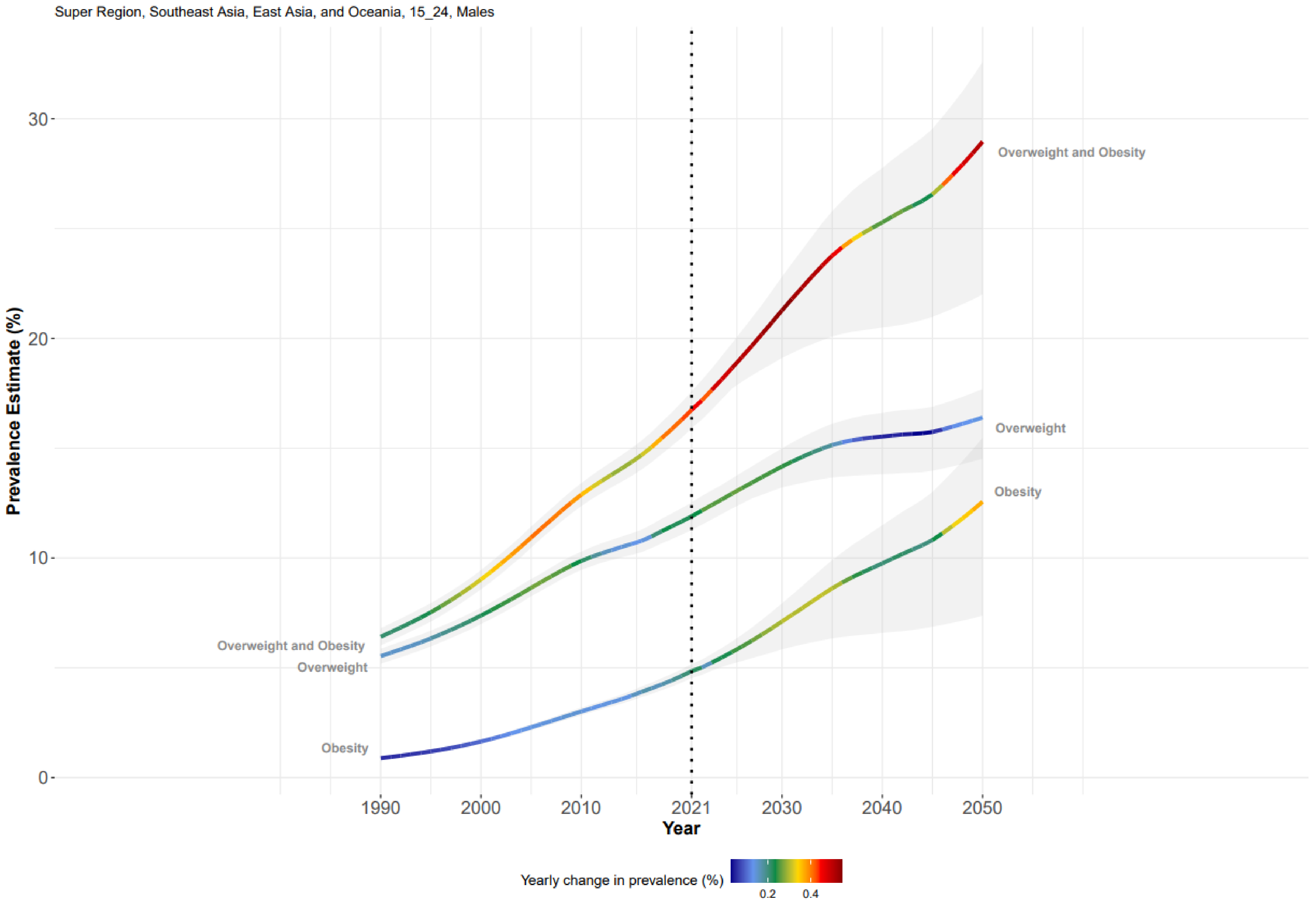

Super Region, Southeast Asia, East Asia, and Oceania, 15_24, Males
Prevalence Estimate (%)
30
20
10
0
1990
2000
2010
2021
2030
2040
2050
Year
Overweight and Obesity
Overweight
Obesity
Overweight and Obesity
Overweight
Obesity
Yearly change in prevalence (%)
0.2
0.4

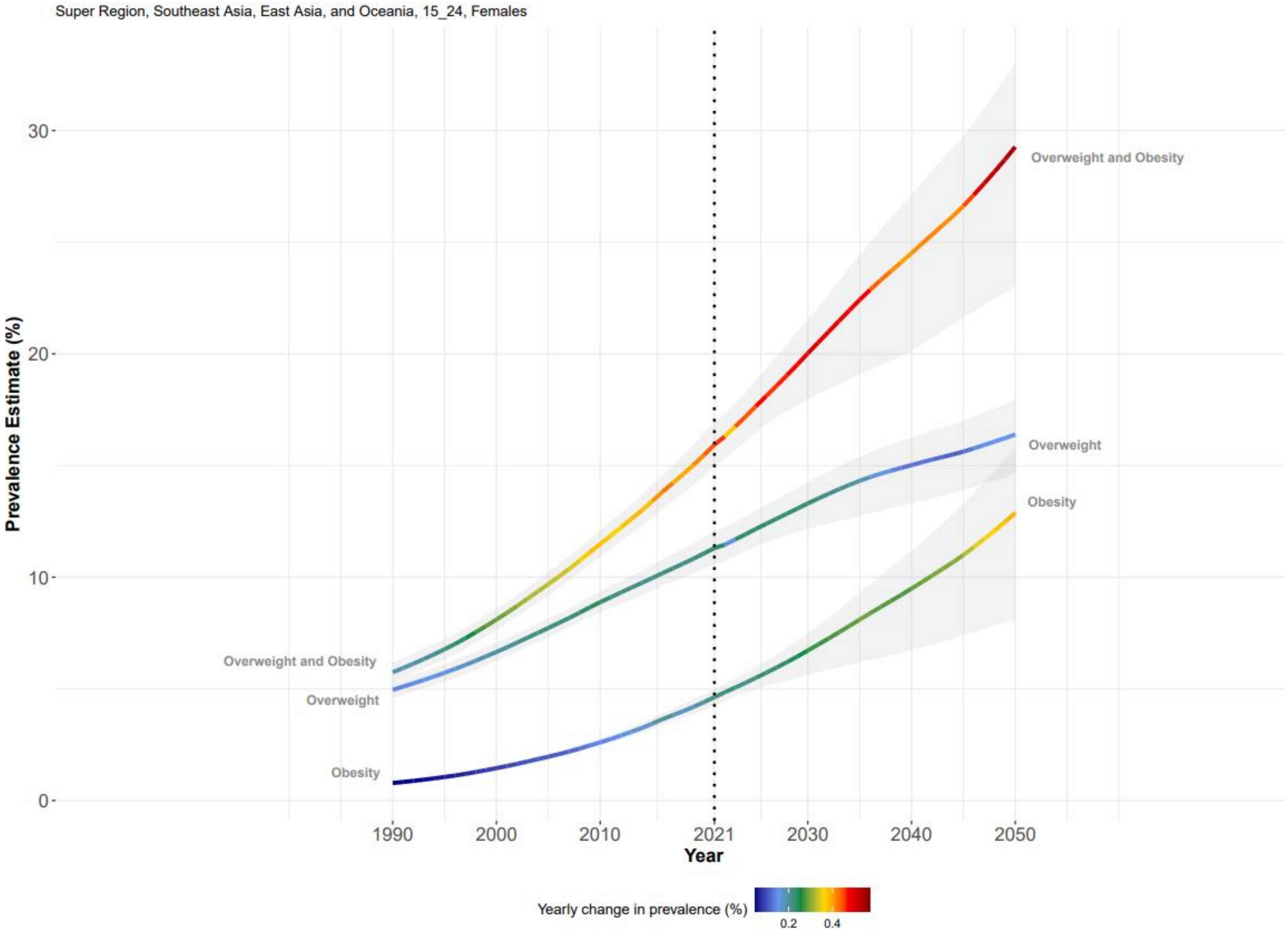
Super Region, Southeast Asia, East Asia, and Oceania, 15_24, Females
Prevalence Estimate (%)
30
20
10
0
1990
2000
2010
2021
2030
2040
2050
Year
Overweight and Obesity
Overweight
Obesity
Overweight and Obesity
Overweight
Obesity
Yearly change in prevalence (%)
0.2
0.4

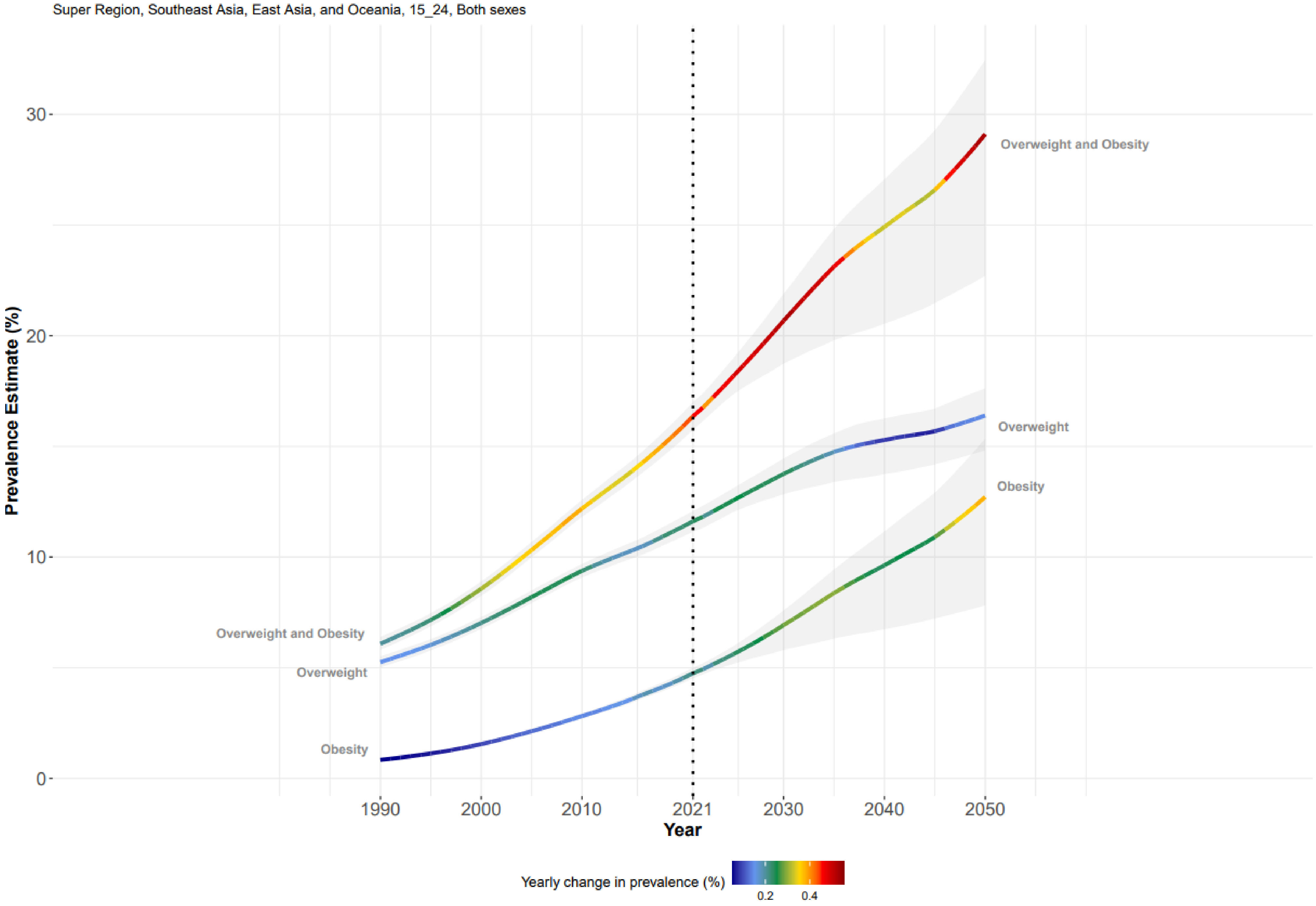

Super Region, Southeast Asia, East Asia, and Oceania, 15_24, Both sexes
Prevalence Estimate (%)
30
20
10
0
Overweight and Obesity
Overweight
Obesity
Overweight and Obesity
Overweight
Obesity
1990
2000
2010
2021
2030
2040
2050
Year
Yearly change in prevalence (%)
0.2
0.4

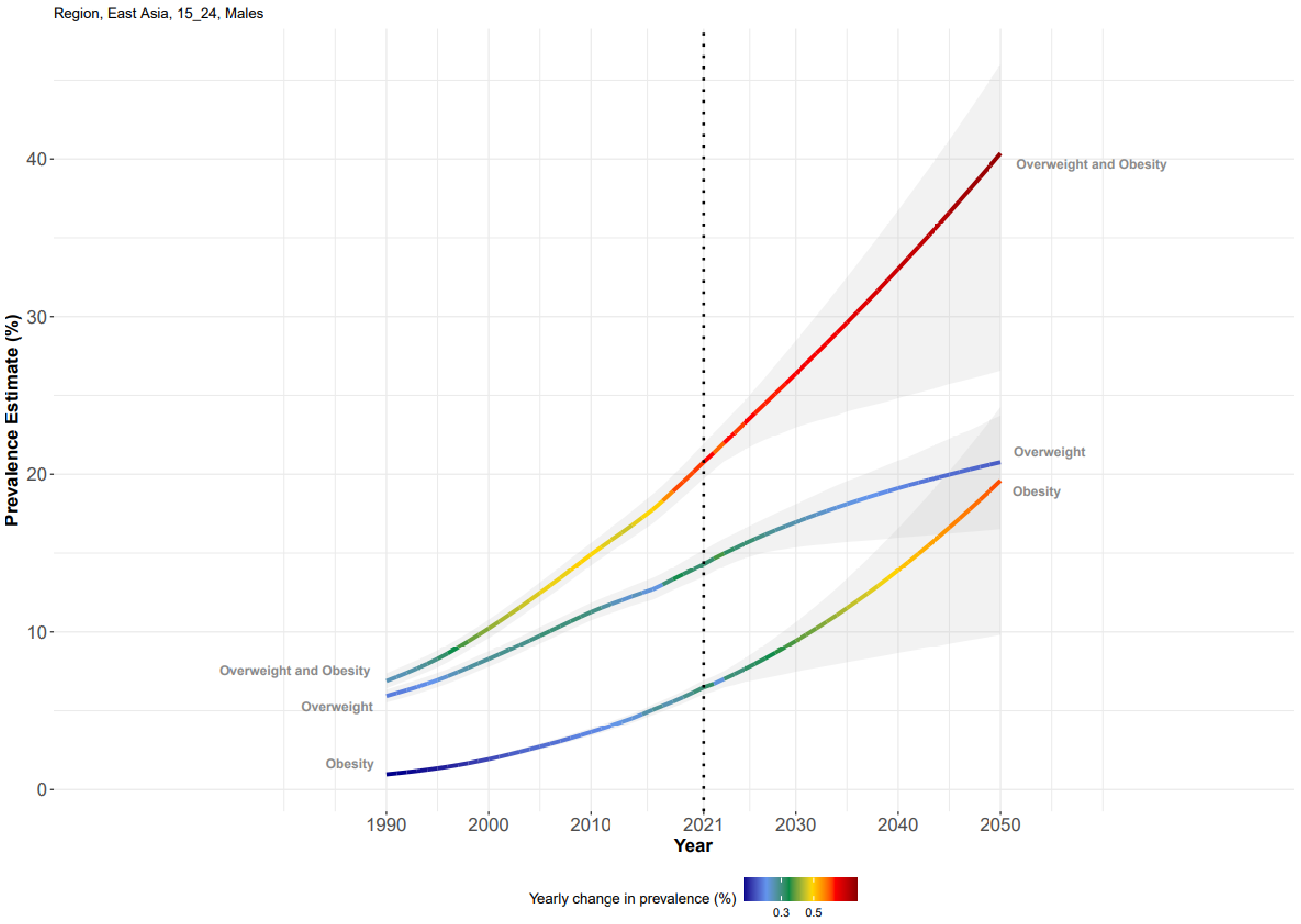
Region, East Asia, 15_24, Males
Prevalence Estimate (%)
40
30
20
10
0
1990
2000
2010
2021
2030
2040
2050
Year
Overweight and Obesity
Overweight
Obesity
Overweight and Obesity
Overweight
Obesity
Yearly change in prevalence (%)
0.3
0.5

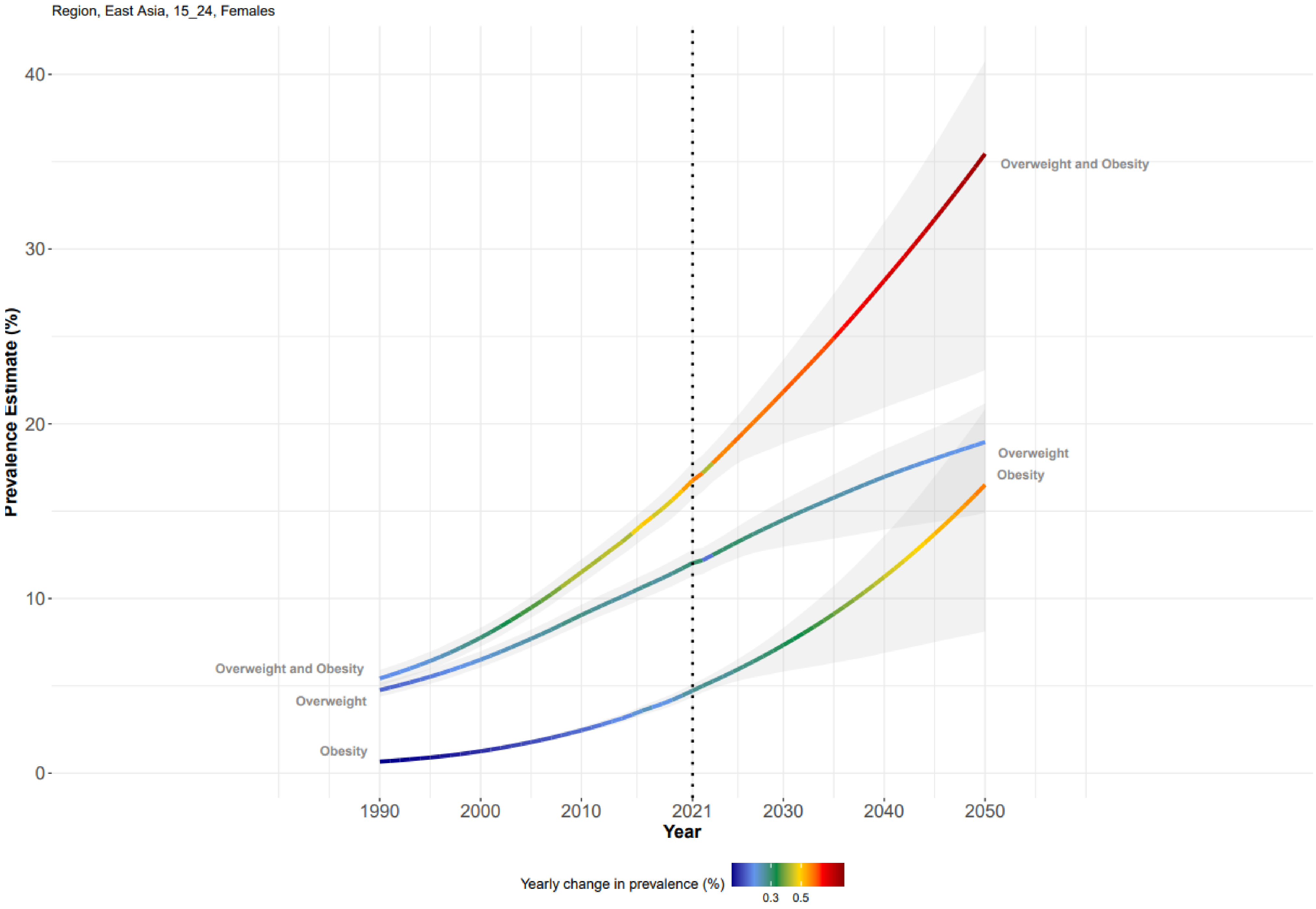
Region, East Asia, 15_24, Females
Prevalence Estimate (%)
40
30
20
10
0
Overweight and Obesity
Overweight
Obesity
Overweight and Obesity
Overweight
Obesity
1990
2000
2010
2021
2030
2040
2050
Year
Yearly change in prevalence (%)
0.3
0.5

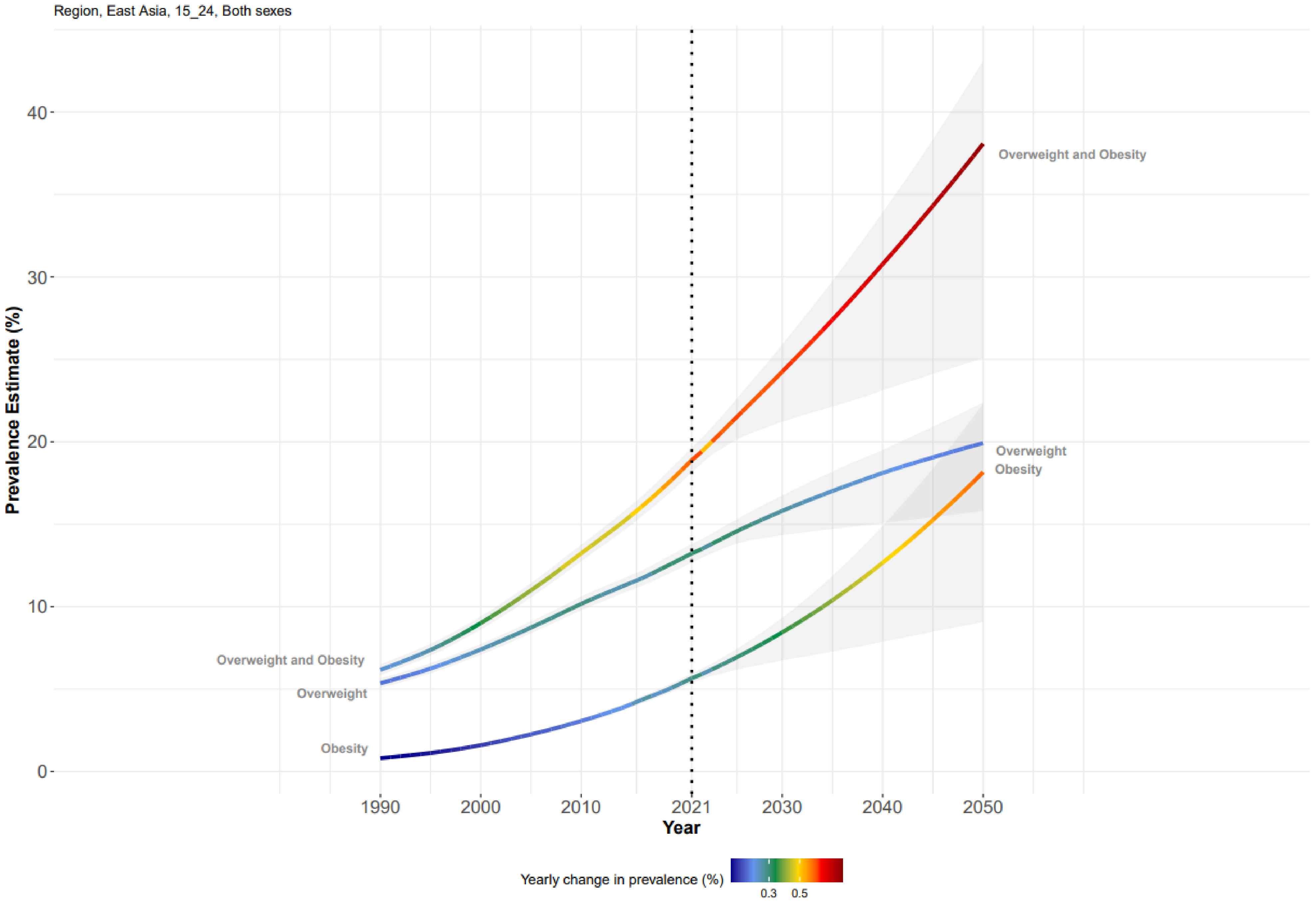
Region, East Asia, 15_24, Both sexes
Prevalence Estimate (%)
40
30
20
10
0
1990
2000
2010
2021
2030
2040
2050
Year
Overweight and Obesity
Overweight
Obesity
Overweight and Obesity
Overweight
Obesity
Yearly change in prevalence (%)
0.3
0.5

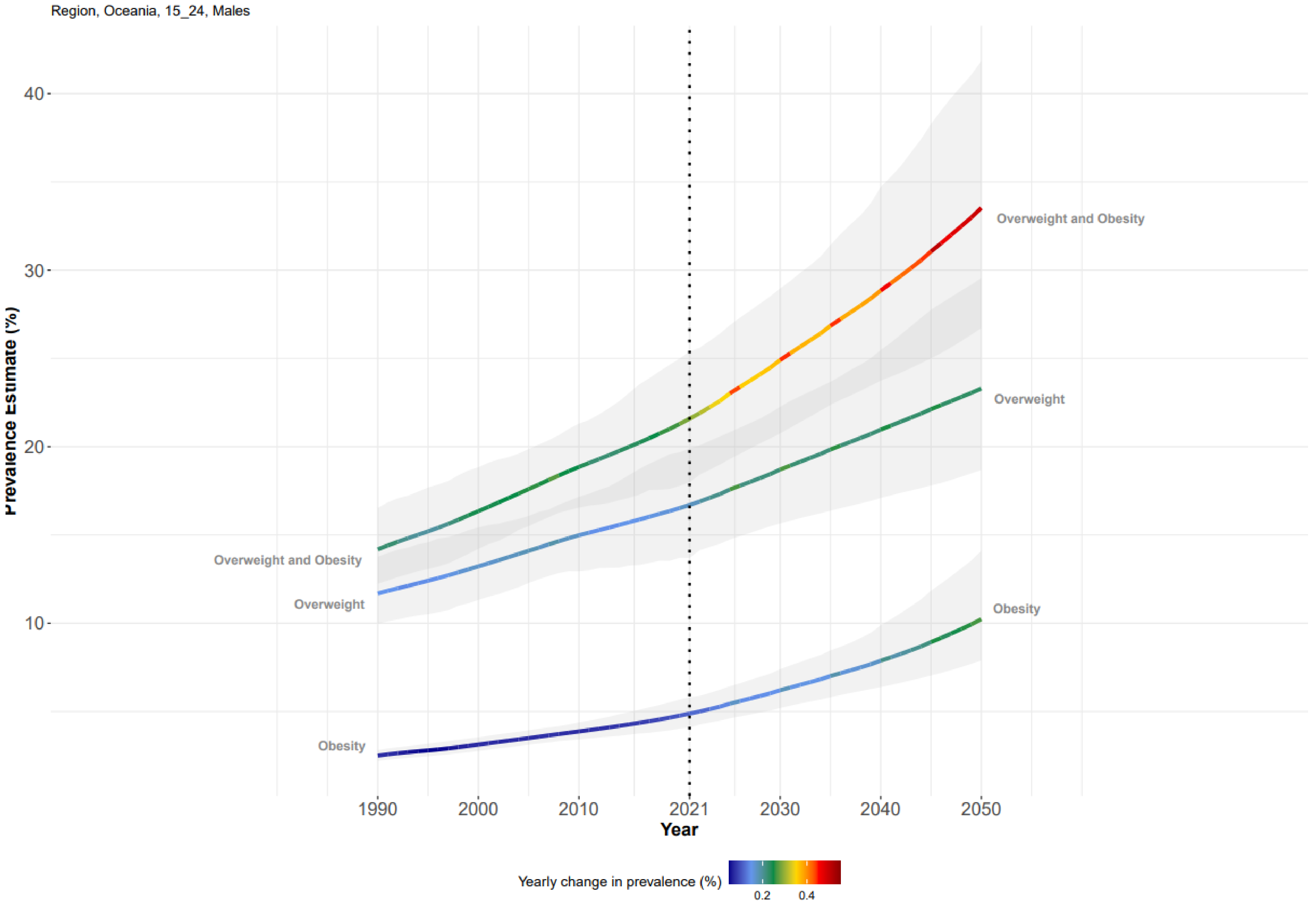
Region, Oceania, 15_24, Males
Prevalence Estimate (%)
40
30
20
10
1990
2000
2010
2021
2030
2040
2050
Year
Overweight and Obesity
Overweight
Obesity
Overweight and Obesity
Overweight
Obesity
Yearly change in prevalence (%)
0.2
0.4

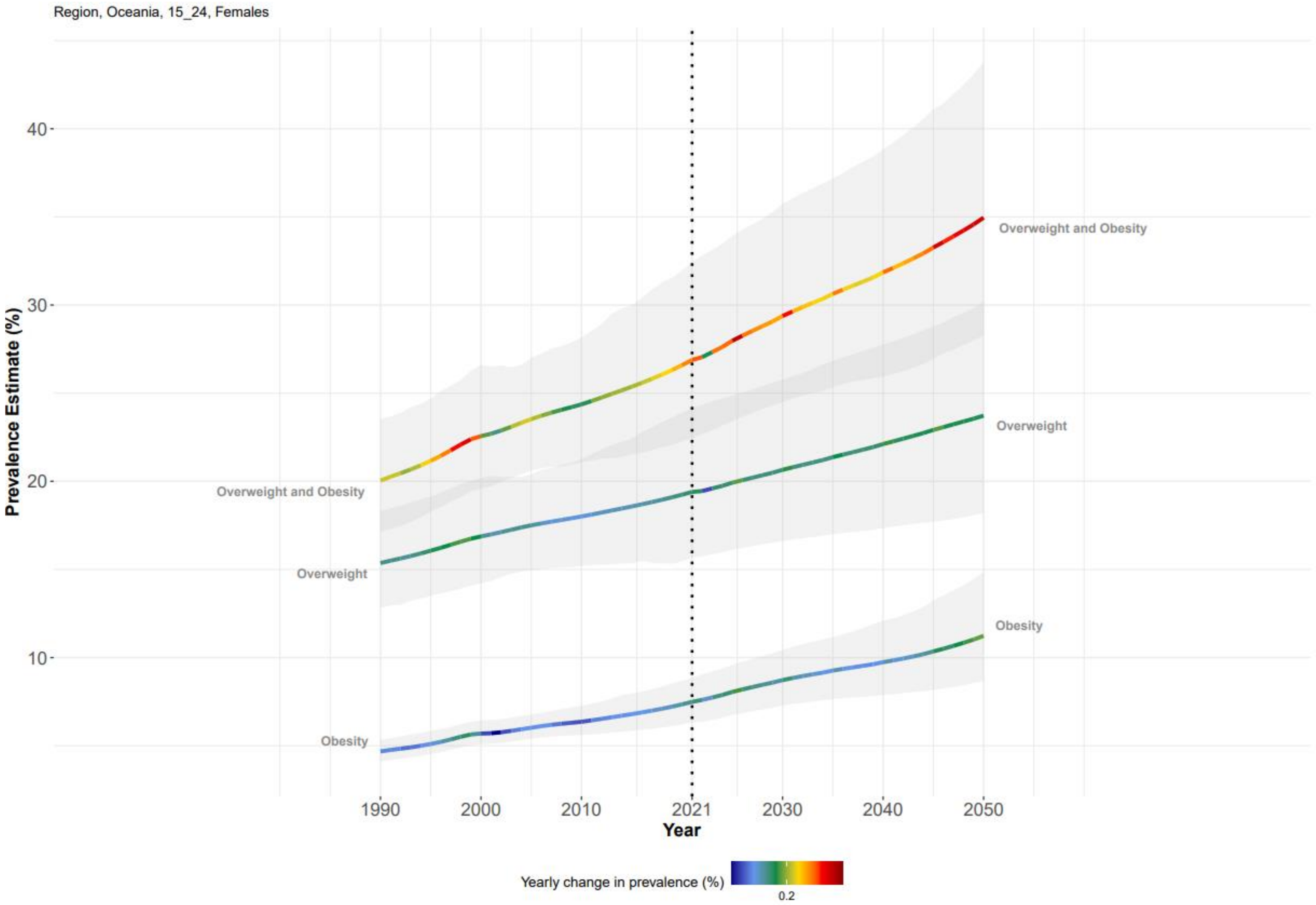
Region, Oceania, 15_24, Females
Prevalence Estimate (%)
40
30
20
10
Overweight and Obesity
Overweight
Obesity
Overweight and Obesity
Overweight
Obesity
1990
2000
2010
2021
2030
2040
2050
Year
Yearly change in prevalence (%)
0.2

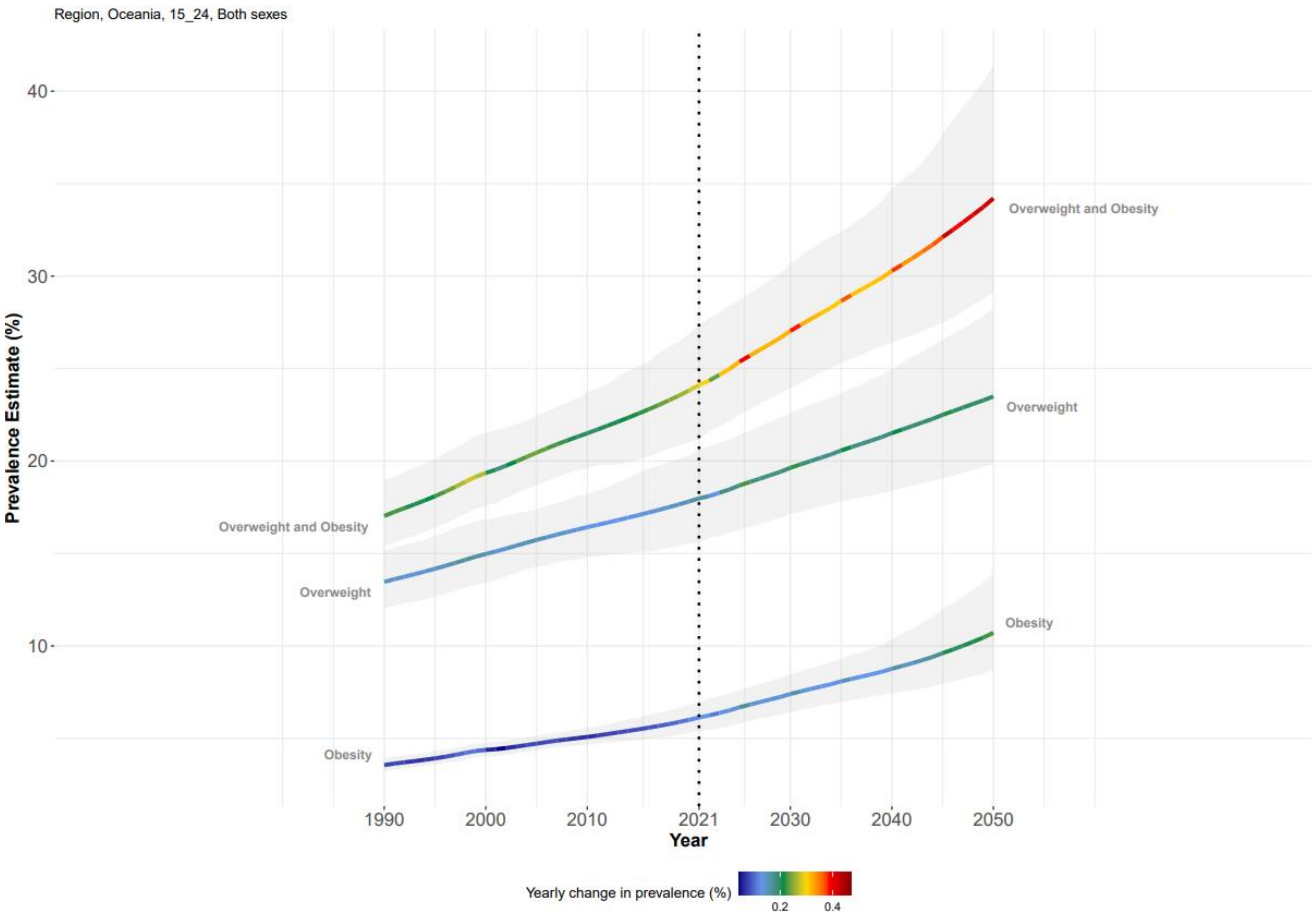
Region, Oceania, 15_24, Both sexes
Prevalence Estimate (%)
40
30
20
10
Overweight and Obesity
Overweight
Obesity
Overweight and Obesity
Overweight
Obesity
1990
2000
2010
2021
2030
2040
2050
Year
Yearly change in prevalence (%)
0.2
0.4

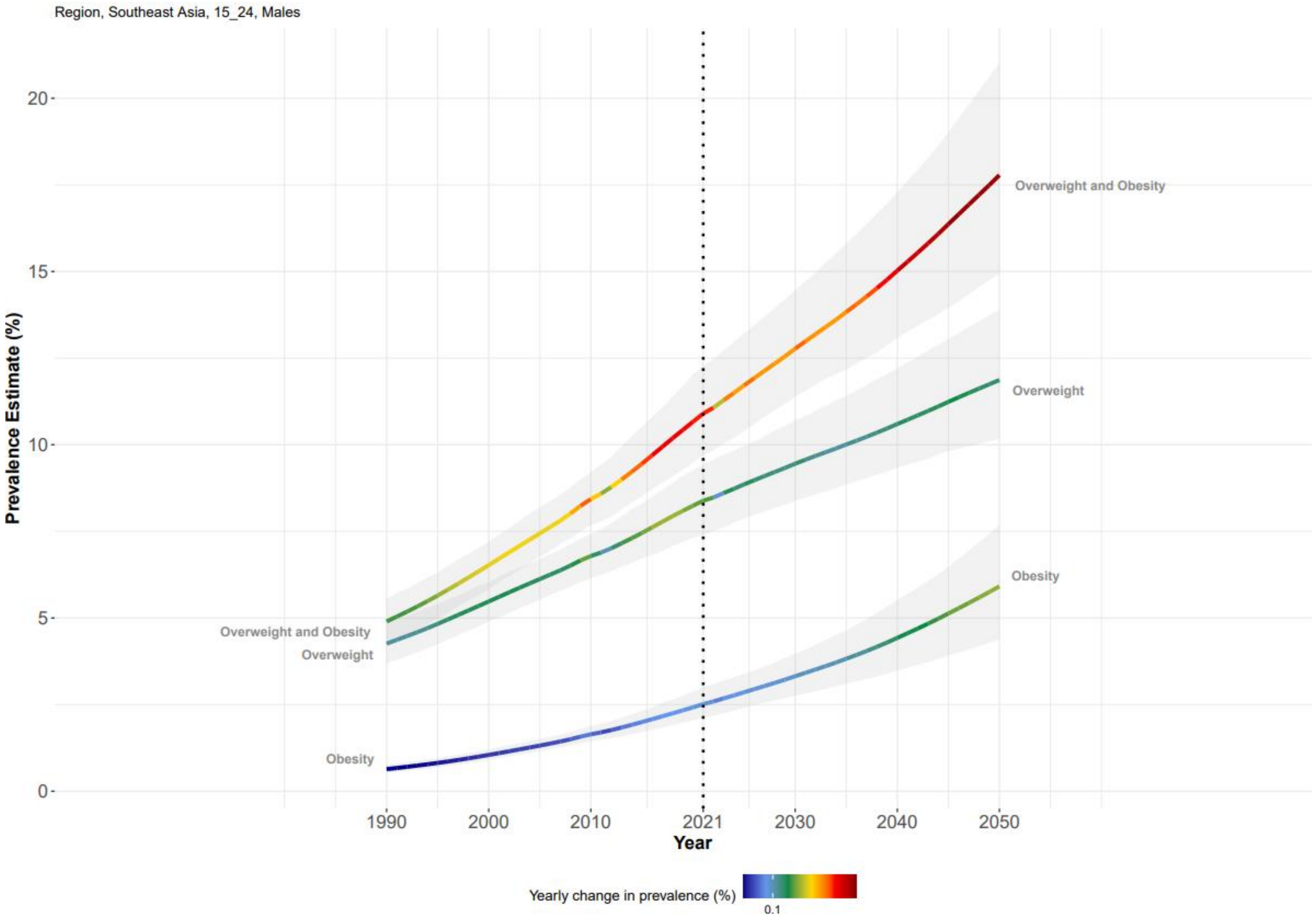

Region, Southeast Asia, 15_24, Males
Prevalence Estimate (%)
20
15
10
5
0
Overweight and Obesity
Overweight
Obesity
Overweight and Obesity
Overweight
Obesity
1990
2000
2010
2021
2030
2040
2050
Year
Yearly change in prevalence (%)
0.1

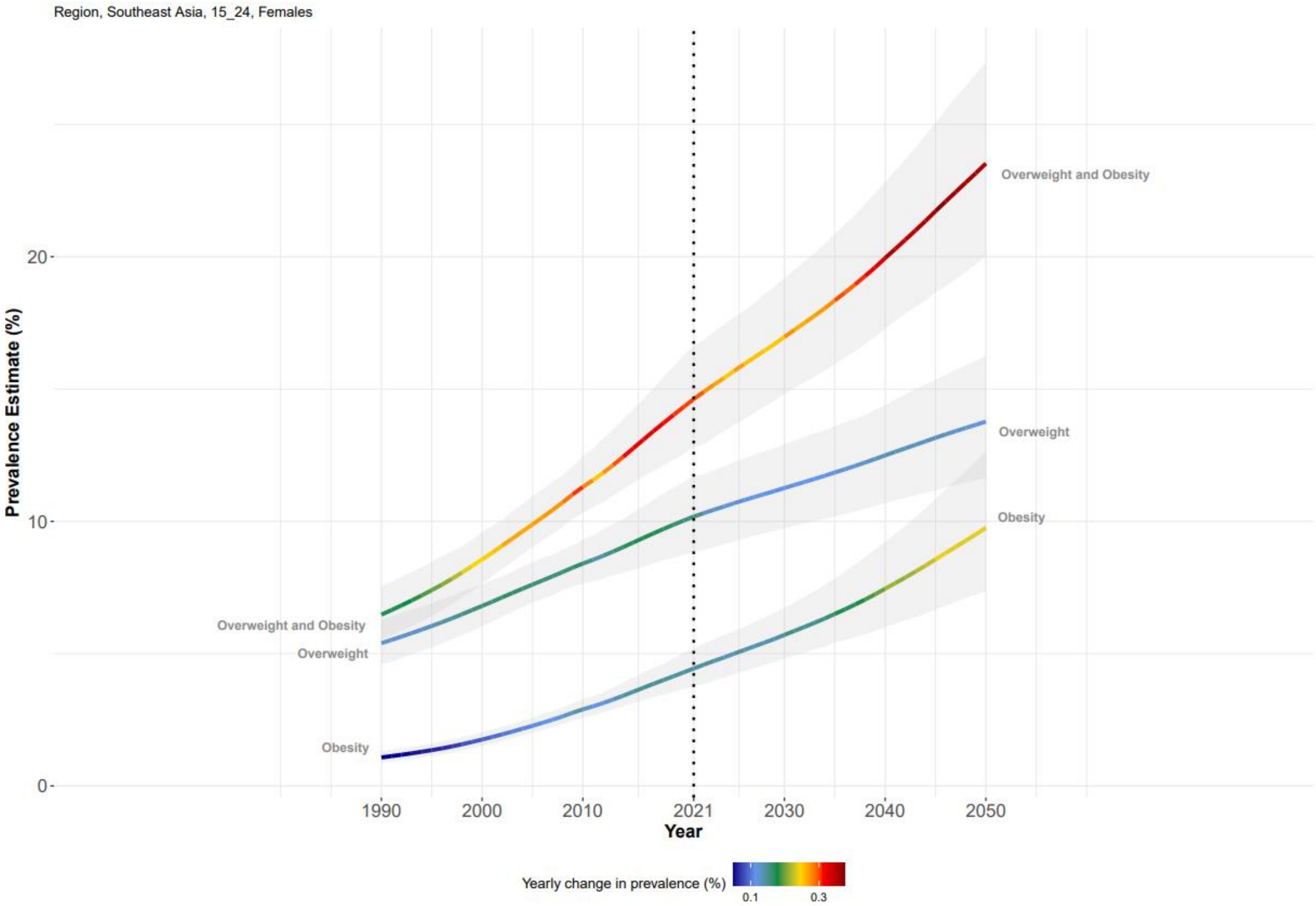

Region, Southeast Asia, 15_24, Females
Prevalence Estimate (%)
20
10
0
1990
2000
2010
2021
2030
2040
2050
Year
Overweight and Obesity
Overweight
Obesity
Overweight and Obesity
Overweight
Obesity
Yearly change in prevalence (%)
0.1
0.3

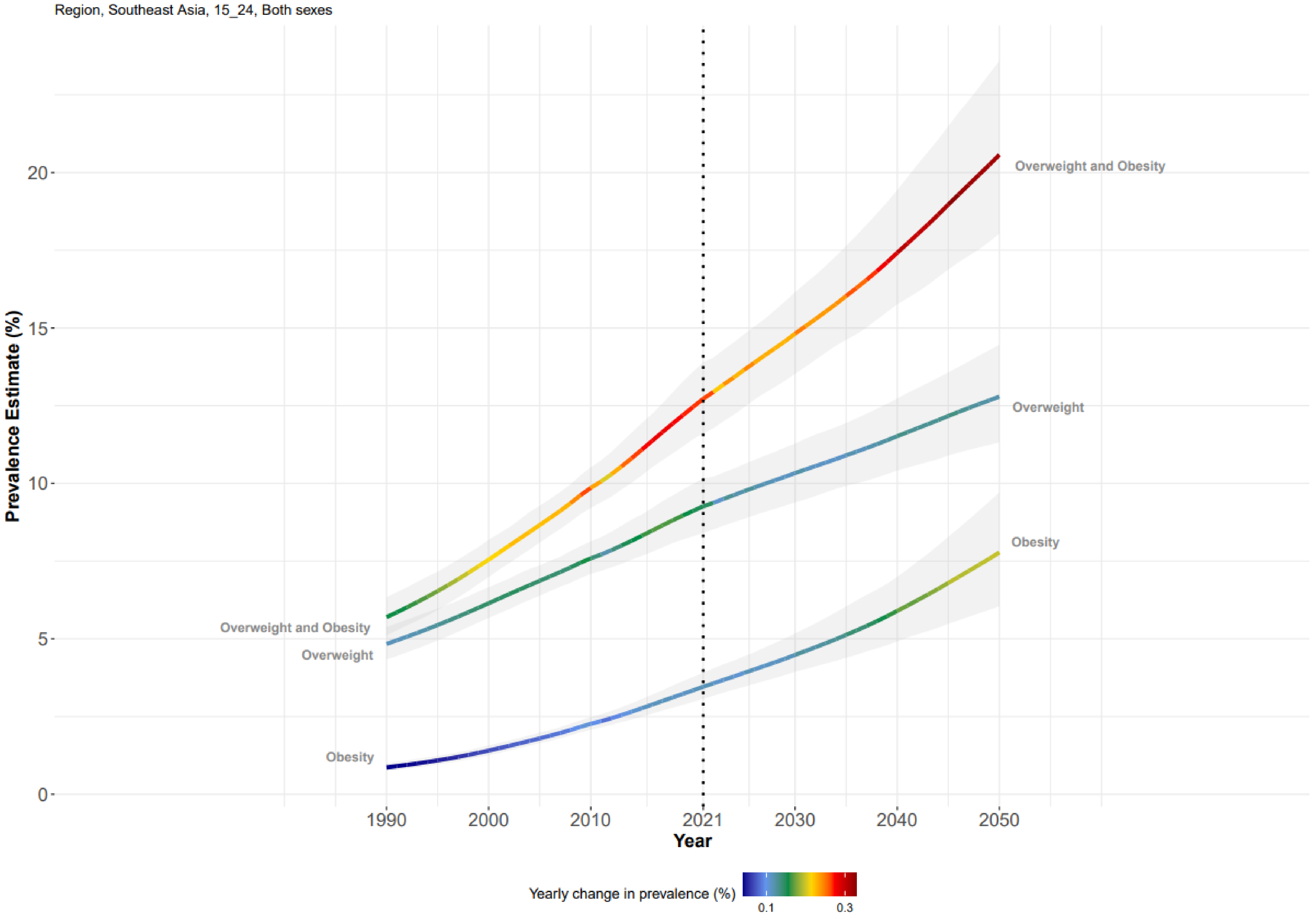

Region, Southeast Asia, 15_24, Both sexes
Prevalence Estimate (%)
20
15
10
5
0
1990
2000
2010
2021
2030
2040
2050
Year
Overweight and Obesity
Overweight
Obesity
Overweight and Obesity
Overweight
Obesity
Yearly change in prevalence (%)
0.1
0.3

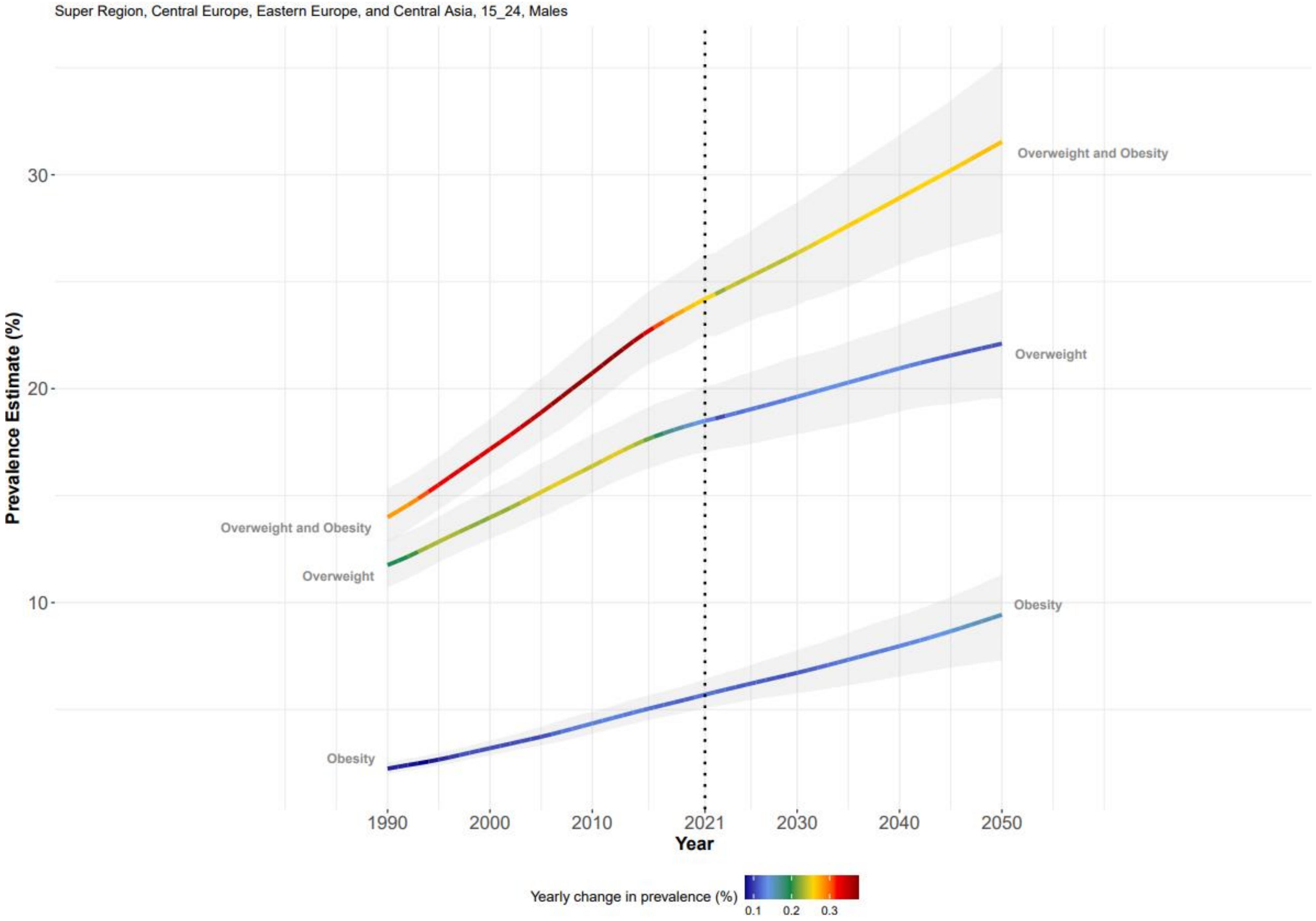
Super Region, Central Europe, Eastern Europe, and Central Asia, 15_24, Males
Prevalence Estimate (%)
30
20
10
Overweight and Obesity
Overweight
Obesity
Overweight and Obesity
Overweight
Obesity
1990
2000
2010
2021
2030
2040
2050
Year
Yearly change in prevalence (%)
0.1
0.2
0.3

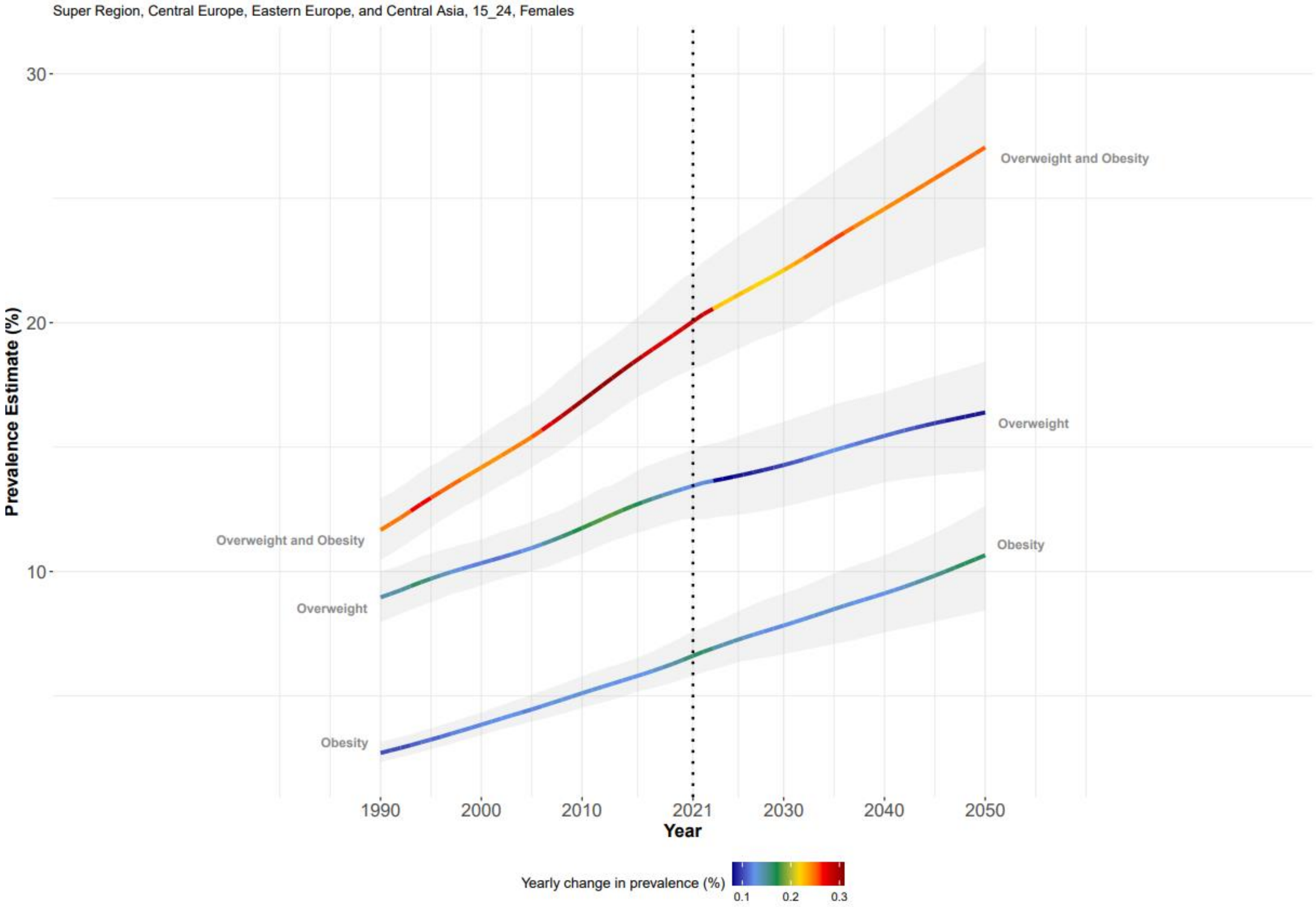

Super Region, Central Europe, Eastern Europe, and Central Asia, 15_24, Females
Prevalence Estimate (%)
30
20
10
Overweight and Obesity
Overweight
Obesity
Overweight and Obesity
Overweight
Obesity
1990
2000
2010
2021
2030
2040
2050
Year
Yearly change in prevalence (%)
0.1
0.2
0.3

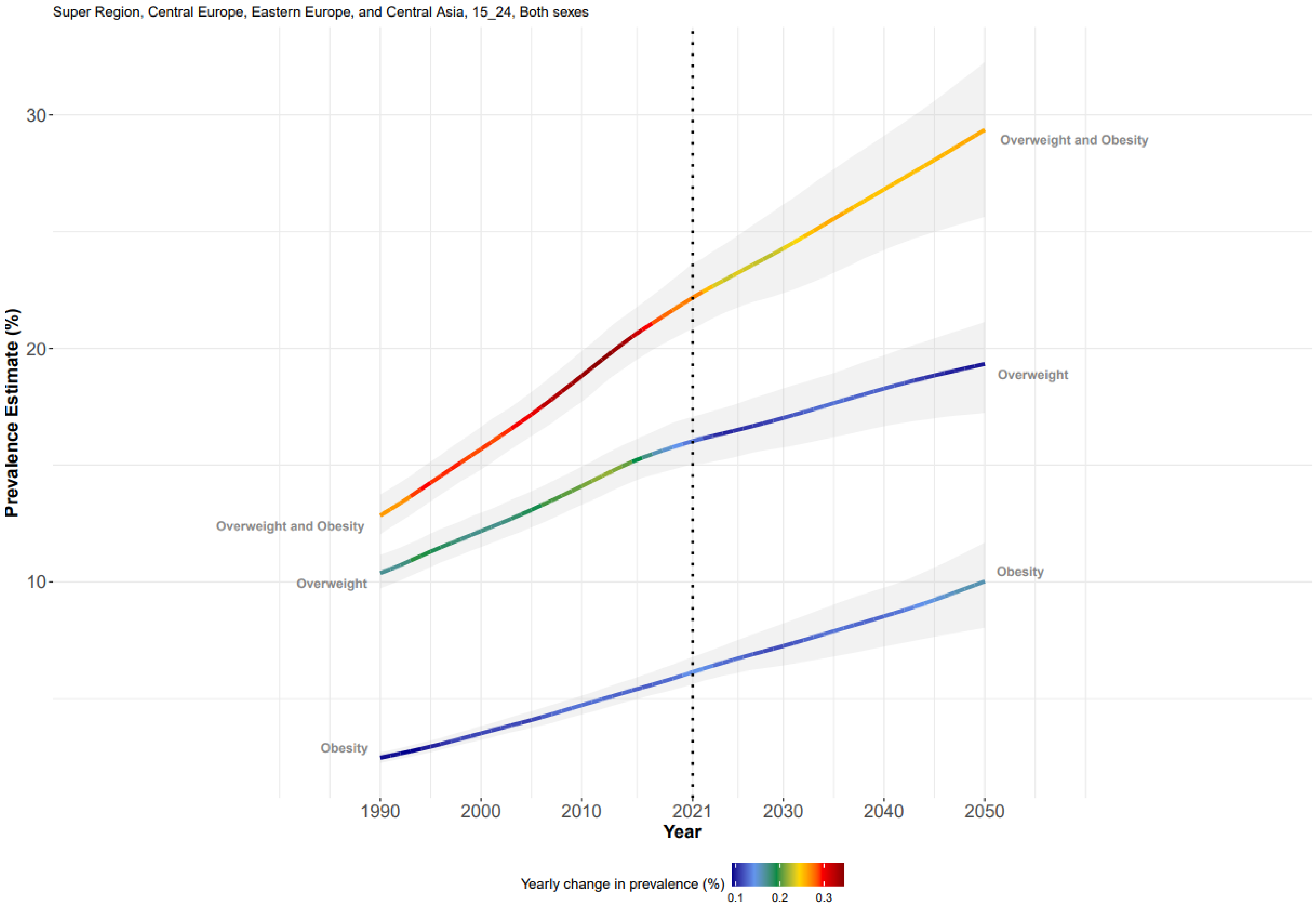

Super Region, Central Europe, Eastern Europe, and Central Asia, 15_24, Both sexes
Prevalence Estimate (%)
30
20
10
Overweight and Obesity
Overweight
Obesity
Overweight and Obesity
Overweight
Obesity
1990
2000
2010
2021
2030
2040
2050
Year
Yearly change in prevalence (%)
0.1
0.2
0.3

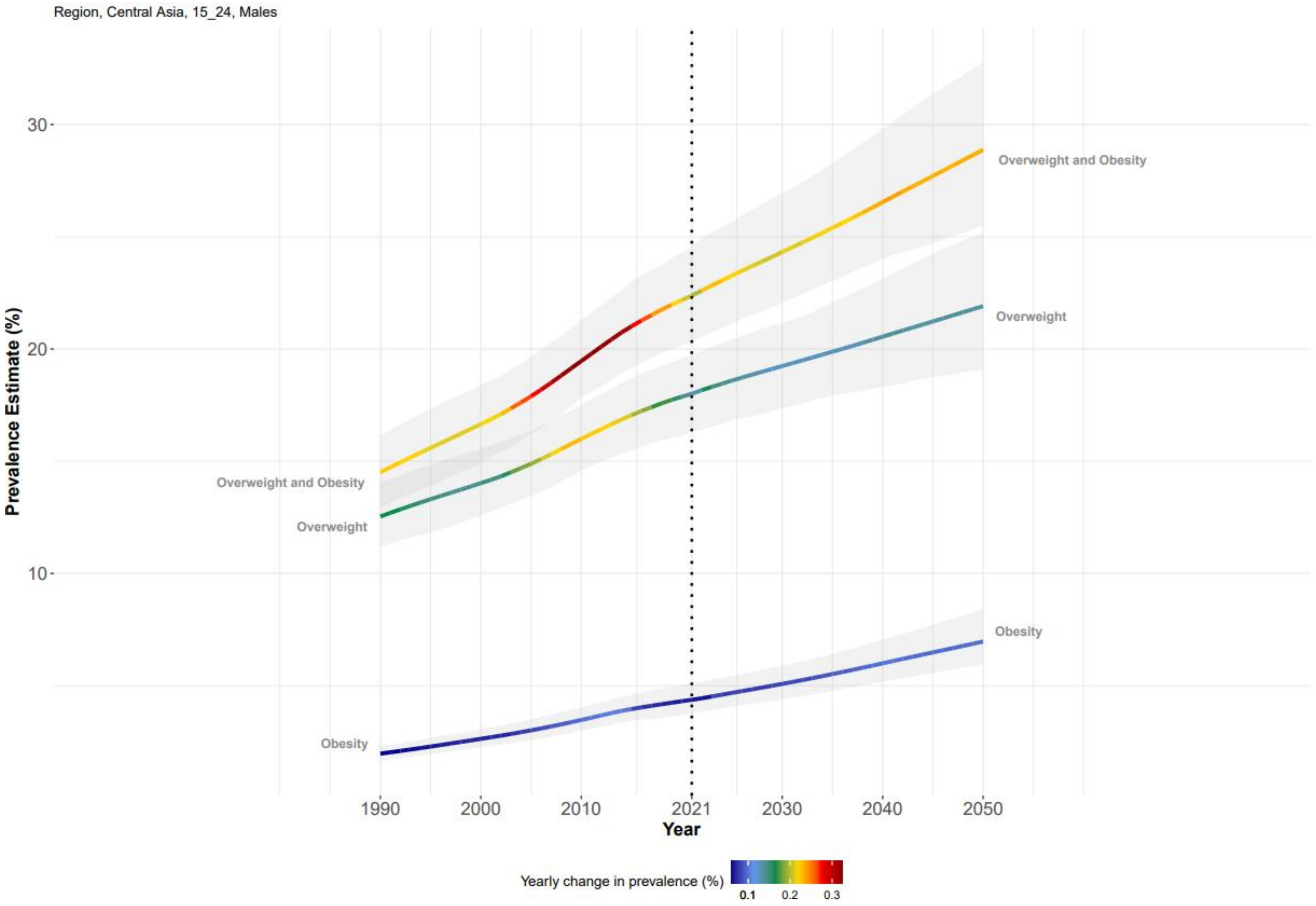

Region, Central Asia, 15_24, Males
Prevalence Estimate (%)
30
20
10
Overweight and Obesity
Overweight
Obesity
Overweight and Obesity
Overweight
Obesity
1990
2000
2010
2021
2030
2040
2050
Year
Yearly change in prevalence (%)
0.1
0.2
0.3

Region, Central Asia, 15_24, Females

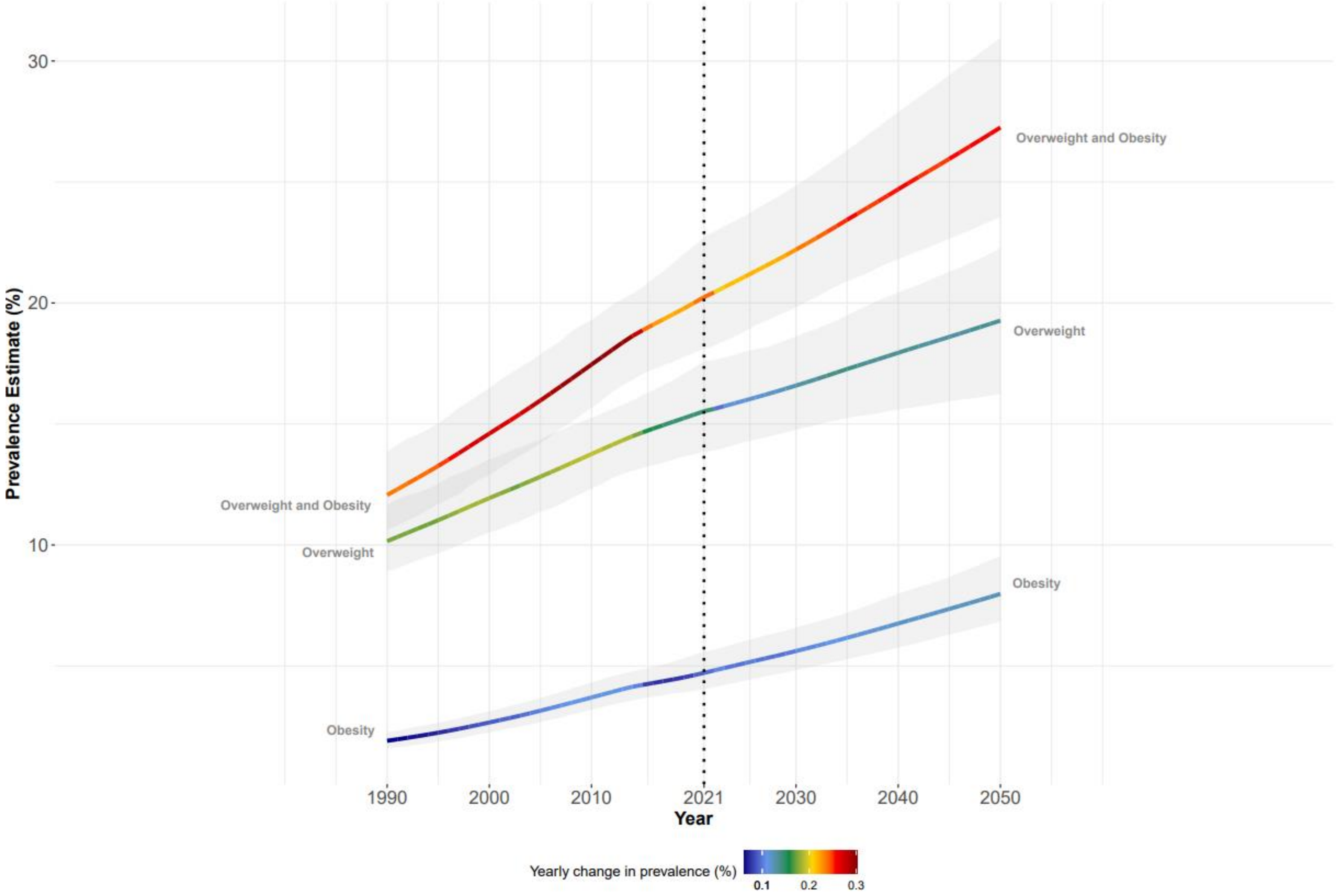

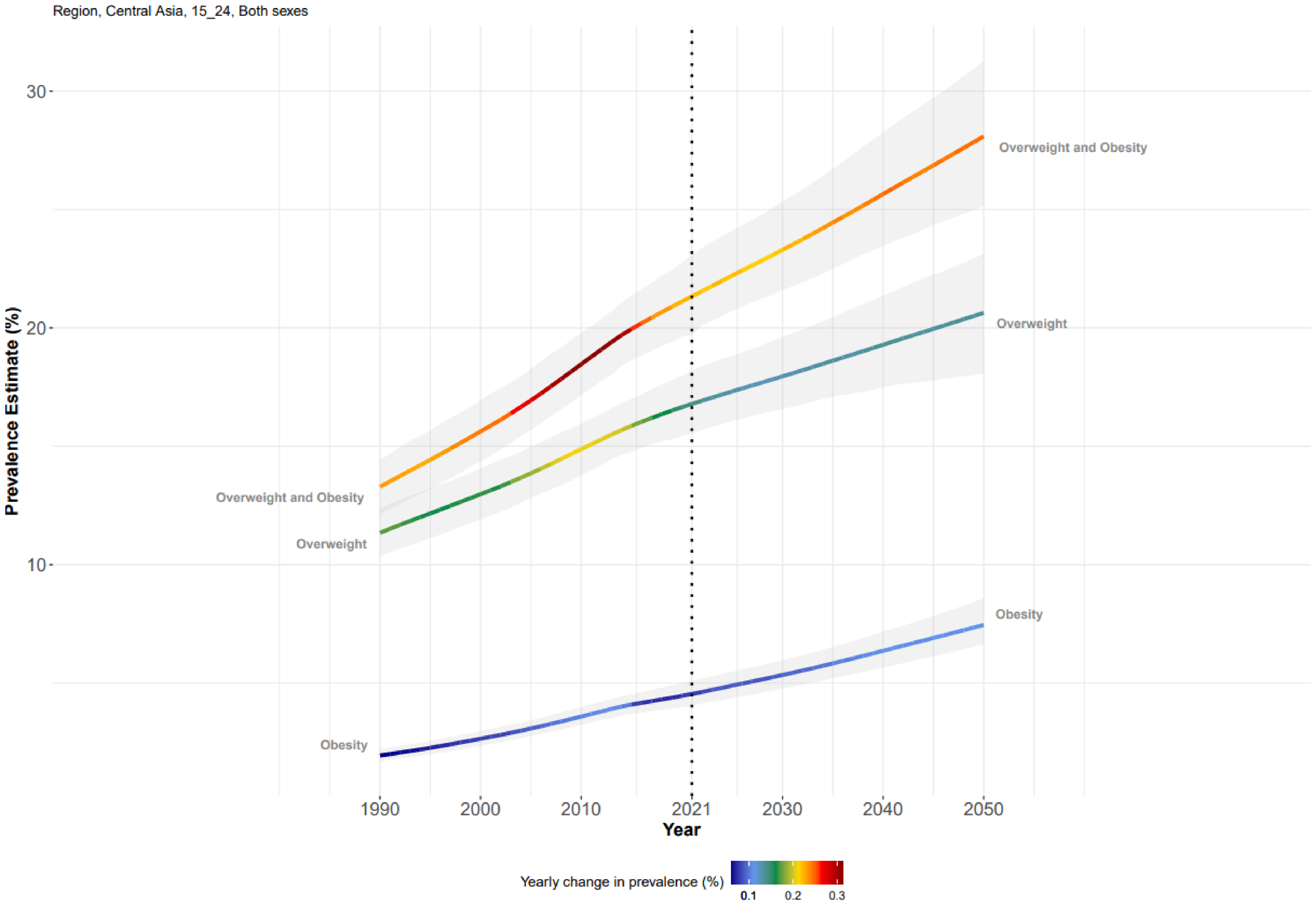
Region, Central Asia, 15_24, Both sexes
Prevalence Estimate (%)
30
20
10
Overweight and Obesity
Overweight
Obesity
Overweight and Obesity
Overweight
Obesity
1990
2000
2010
2021
2030
2040
2050
Year
Yearly change in prevalence (%)
0.1
0.2
0.3

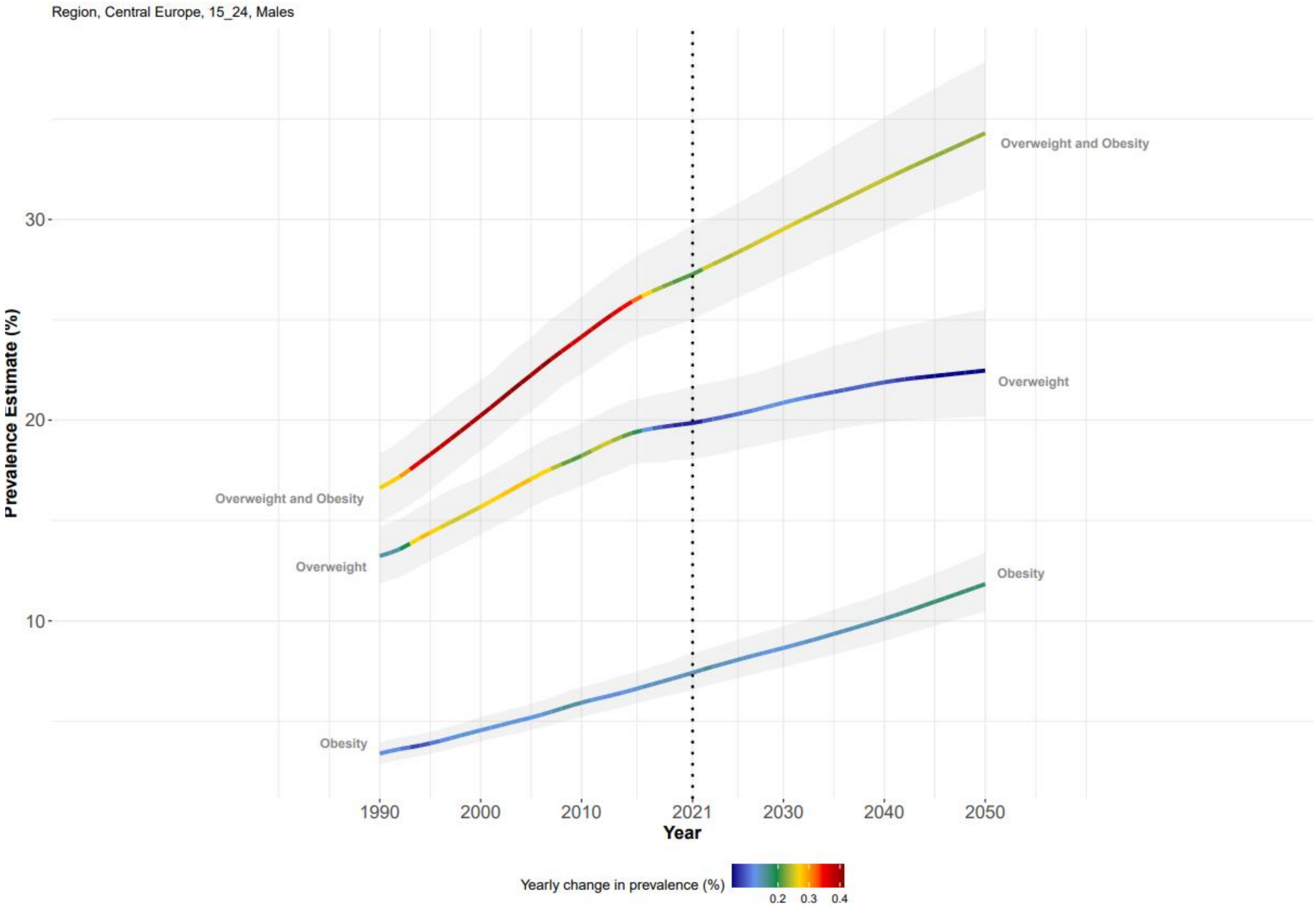

Region, Central Europe, 15_24, Males
Prevalence Estimate (%)
30
20
10
Overweight and Obesity
Overweight
Obesity
Overweight and Obesity
Overweight
Obesity
1990
2000
2010
2021
2030
2040
2050
Year
Yearly change in prevalence (%)
0.2
0.3
0.4

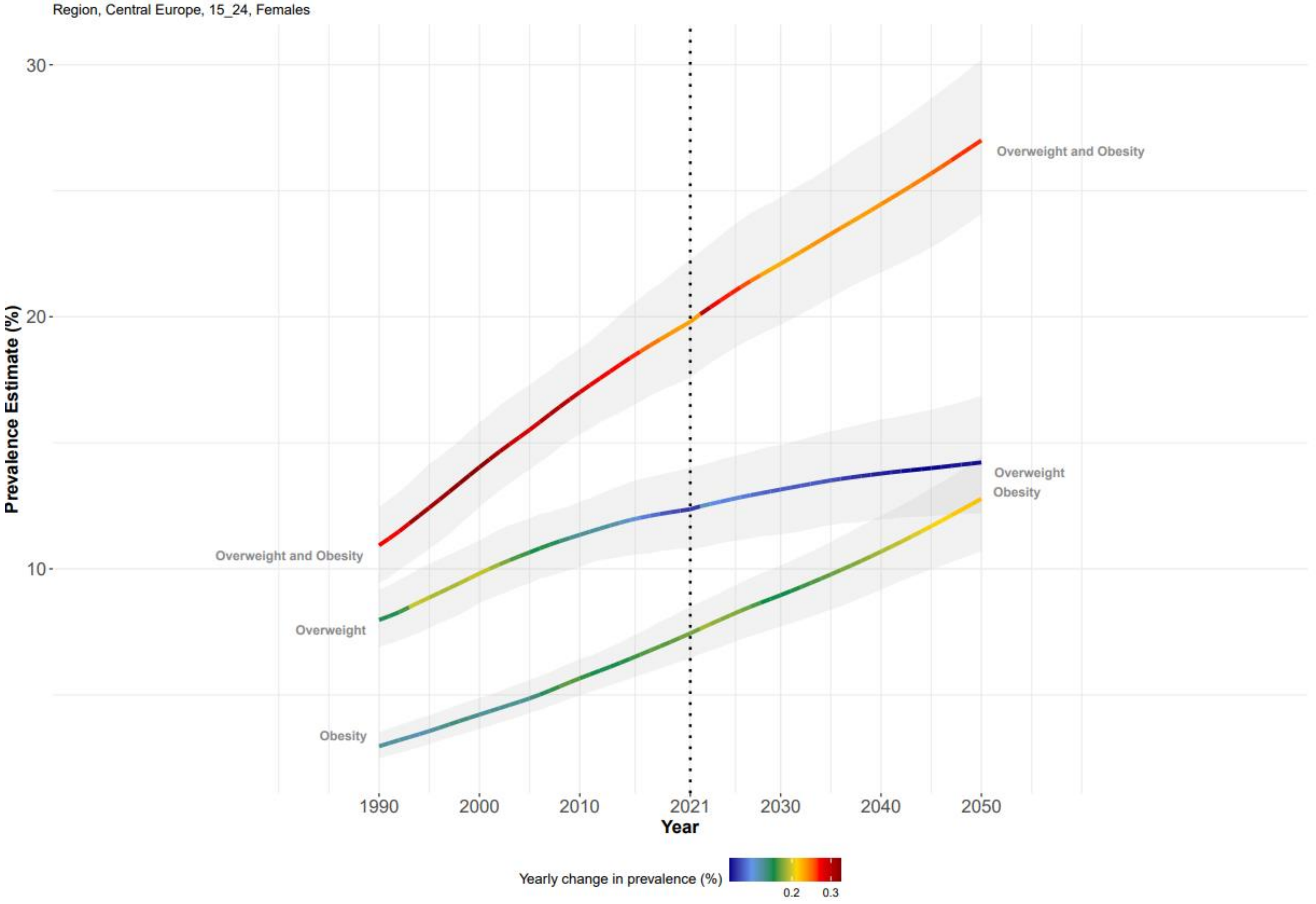

Region, Central Europe, 15_24, Females
30
20
10
Prevalence Estimate (%)
1990
2000
2010
2021
2030
2040
2050
Year
Overweight and Obesity
Overweight
Obesity
Overweight and Obesity
Overweight
Obesity
Yearly change in prevalence (%)
0.2
0.3

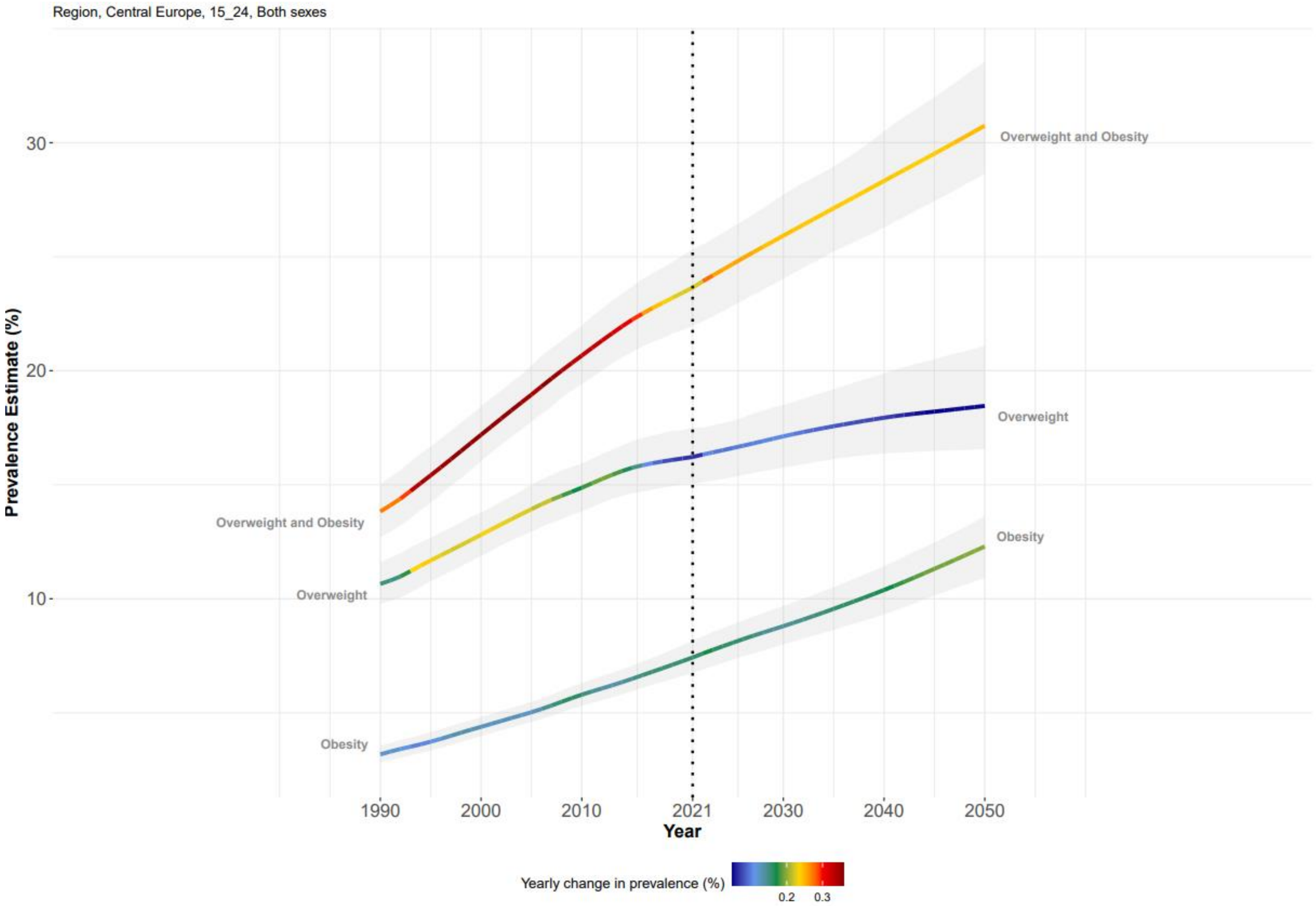
Region, Central Europe, 15_24, Both sexes
Prevalence Estimate (%)
30
20
10
1990
2000
2010
2021
2030
2040
2050
Year
Overweight and Obesity
Overweight
Obesity
Overweight and Obesity
Overweight
Obesity
Yearly change in prevalence (%)
0.2
0.3

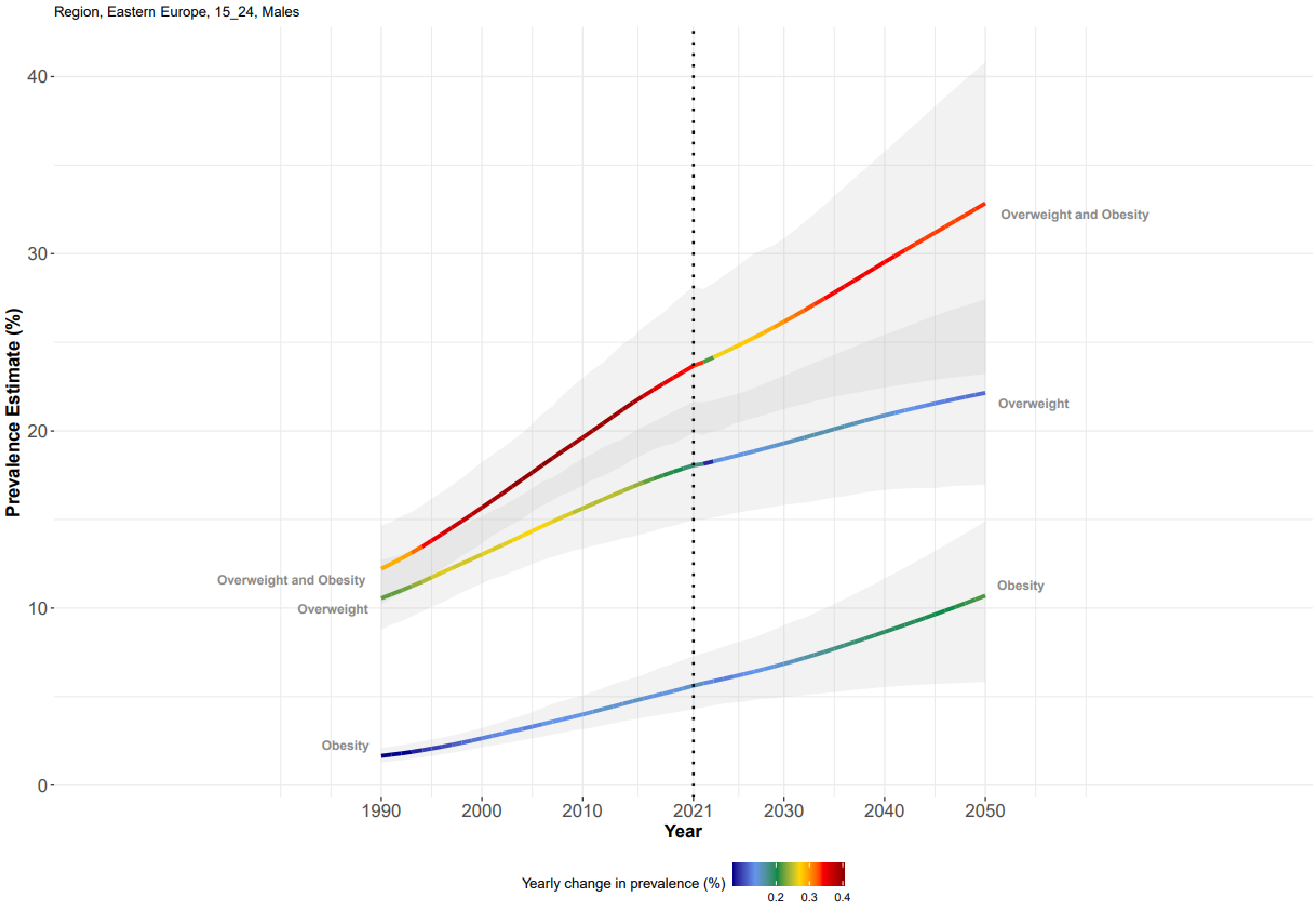

Region, Eastern Europe, 15_24, Males
Prevalence Estimate (%)
0
10
20
30
40
1990
2000
2010
2021
2030
2040
2050
Year
Overweight and Obesity
Overweight
Obesity
Overweight and Obesity
Overweight
Obesity
Yearly change in prevalence (%)
0.2
0.3
0.4

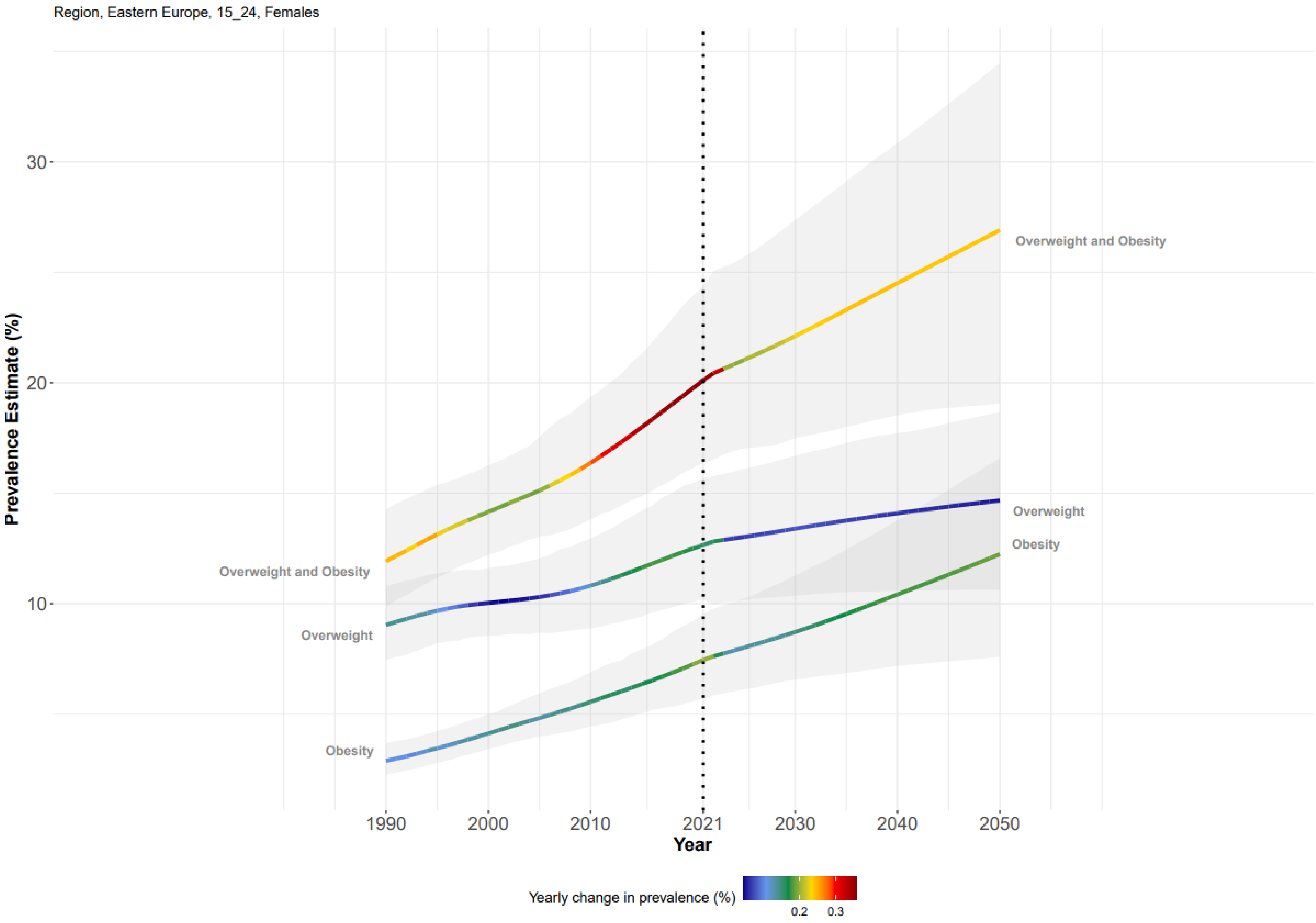
Region, Eastern Europe, 15_24, Females
Prevalence Estimate (%)
30
20
10
Overweight and Obesity
Overweight
Obesity
Overweight and Obesity
Overweight
Obesity
1990
2000
2010
2021
2030
2040
2050
Year
Yearly change in prevalence (%)
0.2
0.3

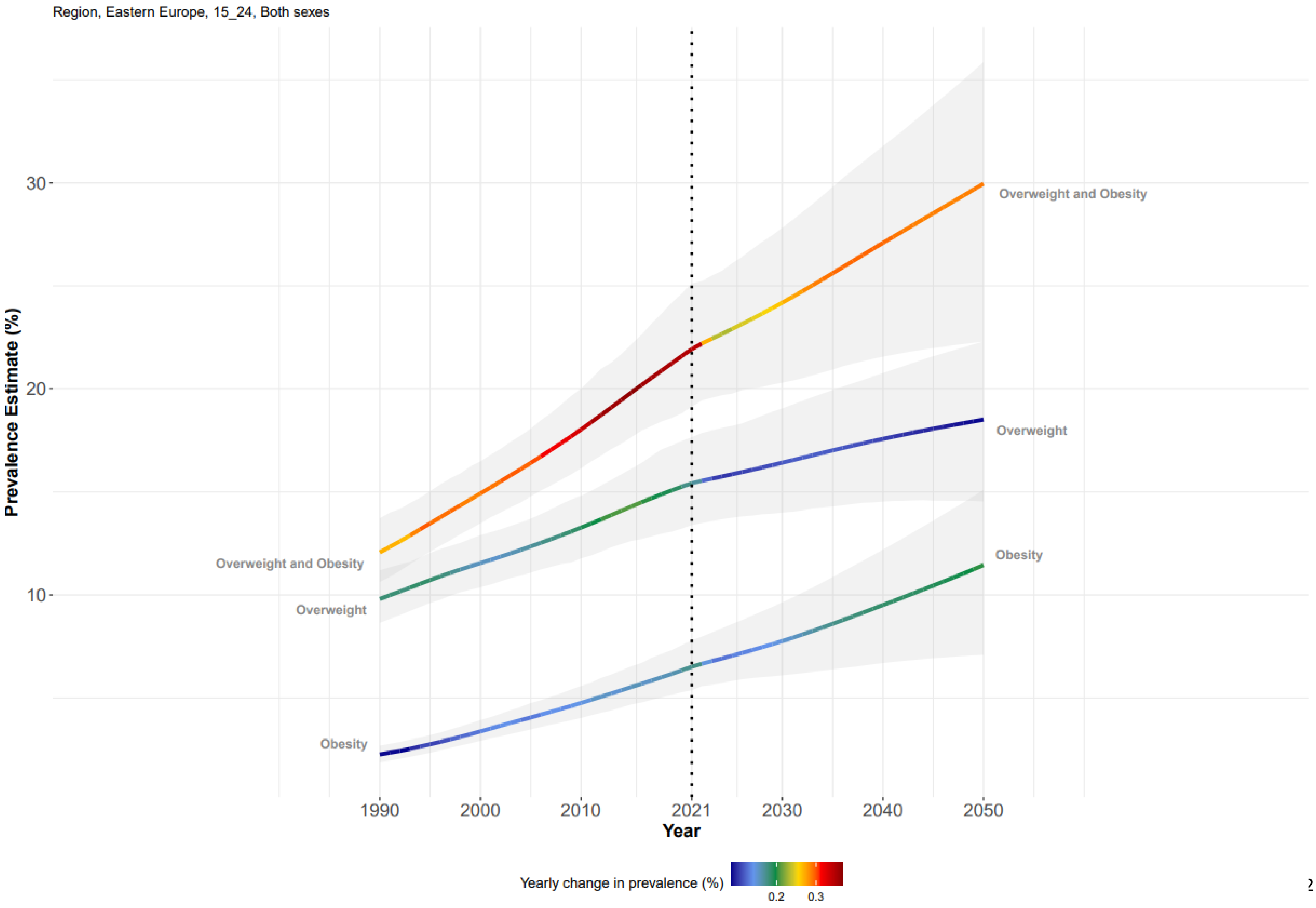

Region, Eastern Europe, 15_24, Both sexes
Prevalence Estimate (%)
30
20
10
Overweight and Obesity
Overweight
Obesity
Overweight and Obesity
Overweight
Obesity
1990
2000
2010
2021
2030
2040
2050
Year
Yearly change in prevalence (%)
0.2
0.3

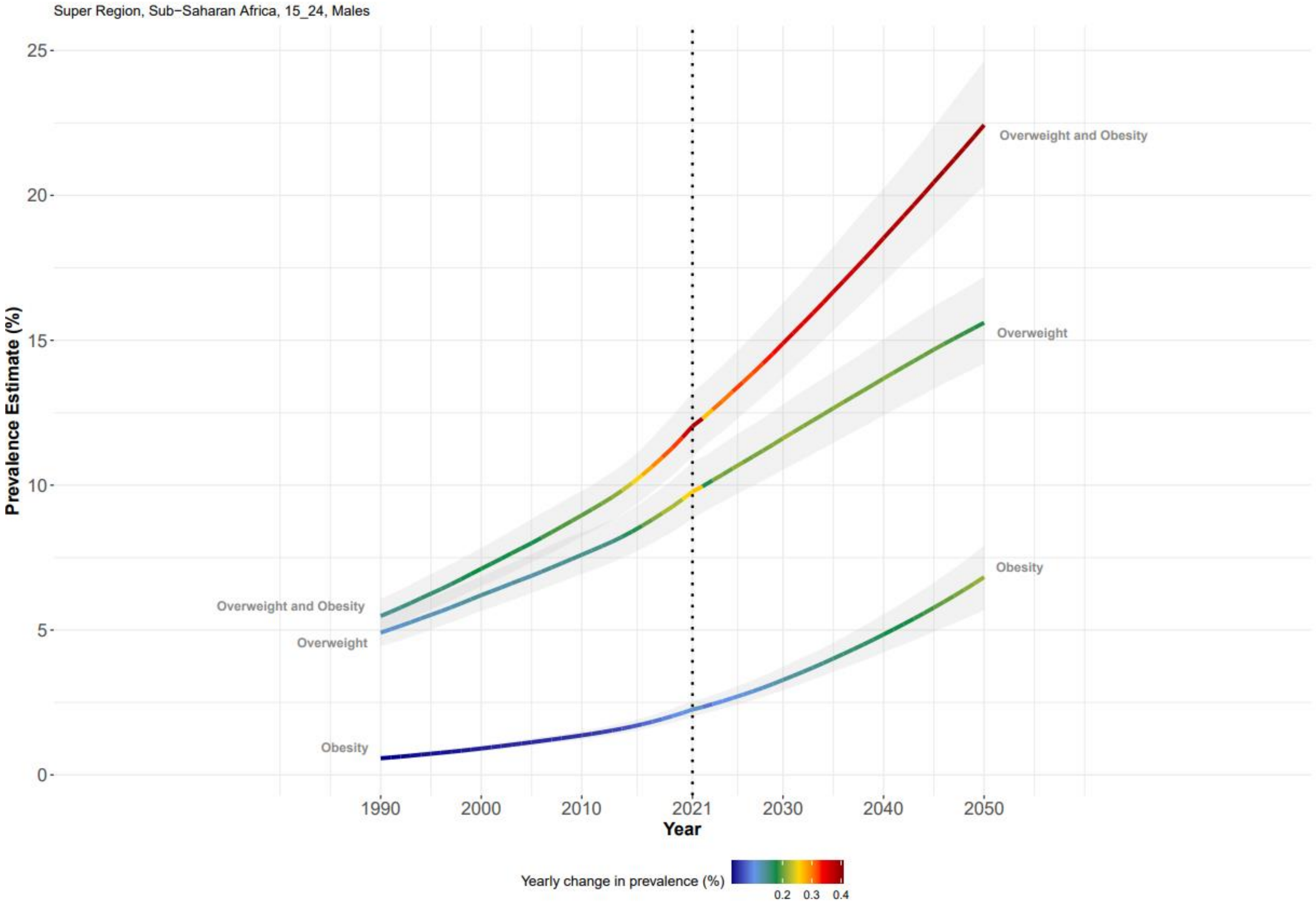

Super Region, Sub−Saharan Africa, 15_24, Males
Prevalence Estimate (%)
25
20
15
10
5
0
Overweight and Obesity
Overweight
Obesity
Overweight and Obesity
Overweight
Obesity
1990
2000
2010
2021
2030
2040
2050
Year
Yearly change in prevalence (%)
0.2
0.3
0.4

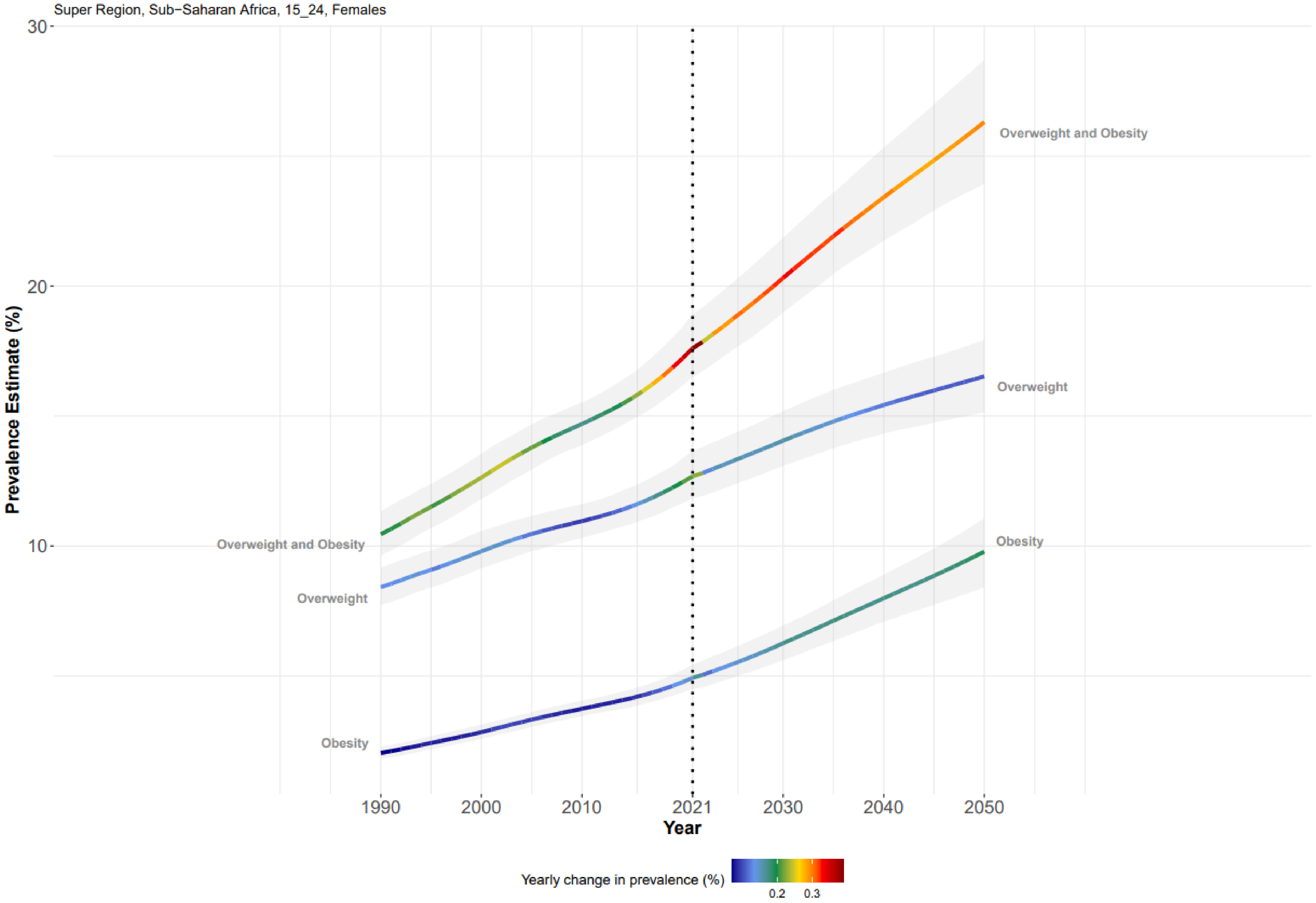

Super Region, Sub−Saharan Africa, 15_24, Females
Prevalence Estimate (%)
30
20
10
Overweight and Obesity
Overweight
Obesity
Overweight and Obesity
Overweight
Obesity
1990
2000
2010
2021
2030
2040
2050
Year
Yearly change in prevalence (%)
0.2
0.3

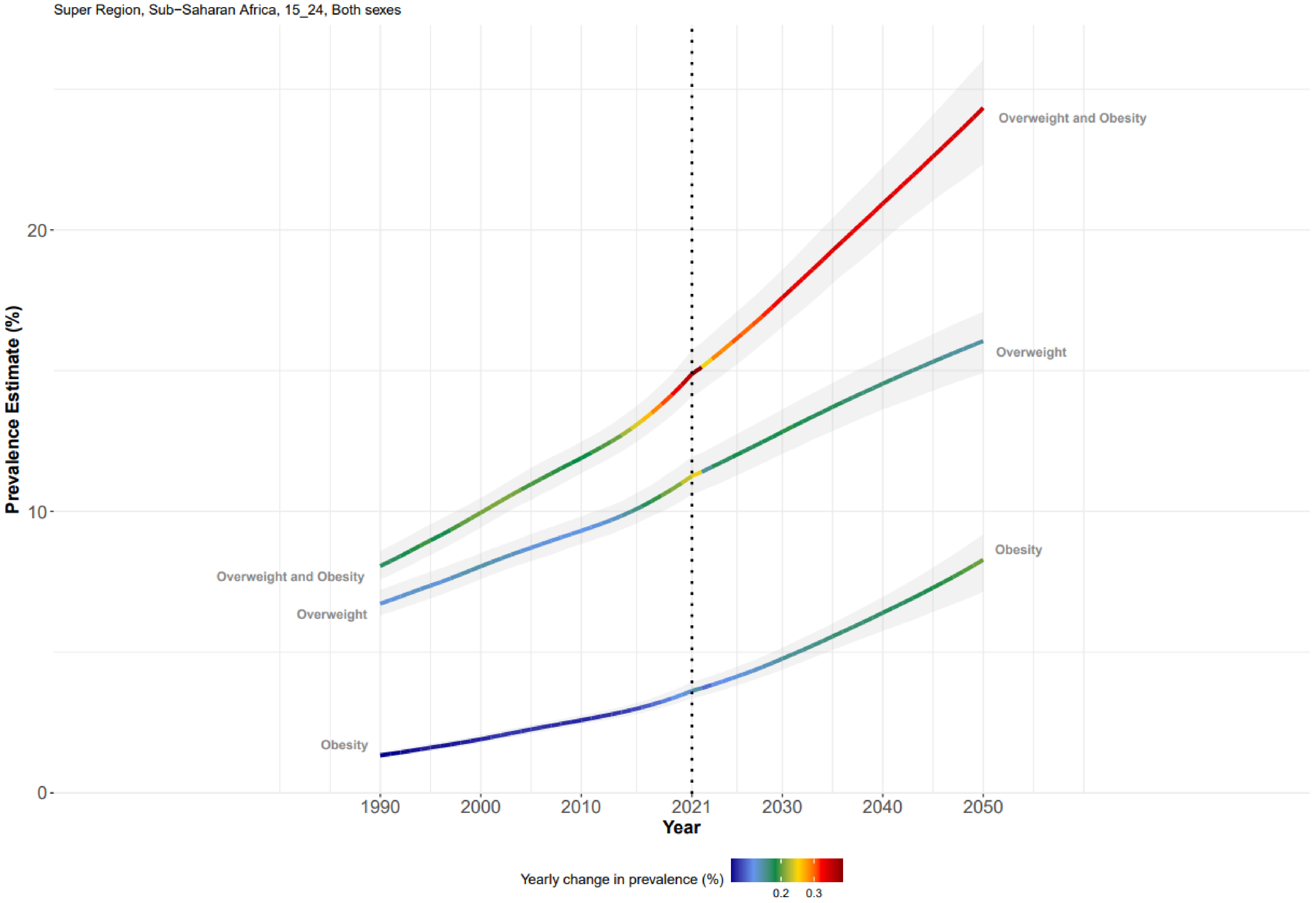

Super Region, Sub−Saharan Africa, 15_24, Both sexes
Prevalence Estimate (%)
20
10
0
Overweight and Obesity
Overweight
Obesity
Overweight and Obesity
Overweight
Obesity
1990
2000
2010
2021
2030
2040
2050
Year
Yearly change in prevalence (%)
0.2
0.3

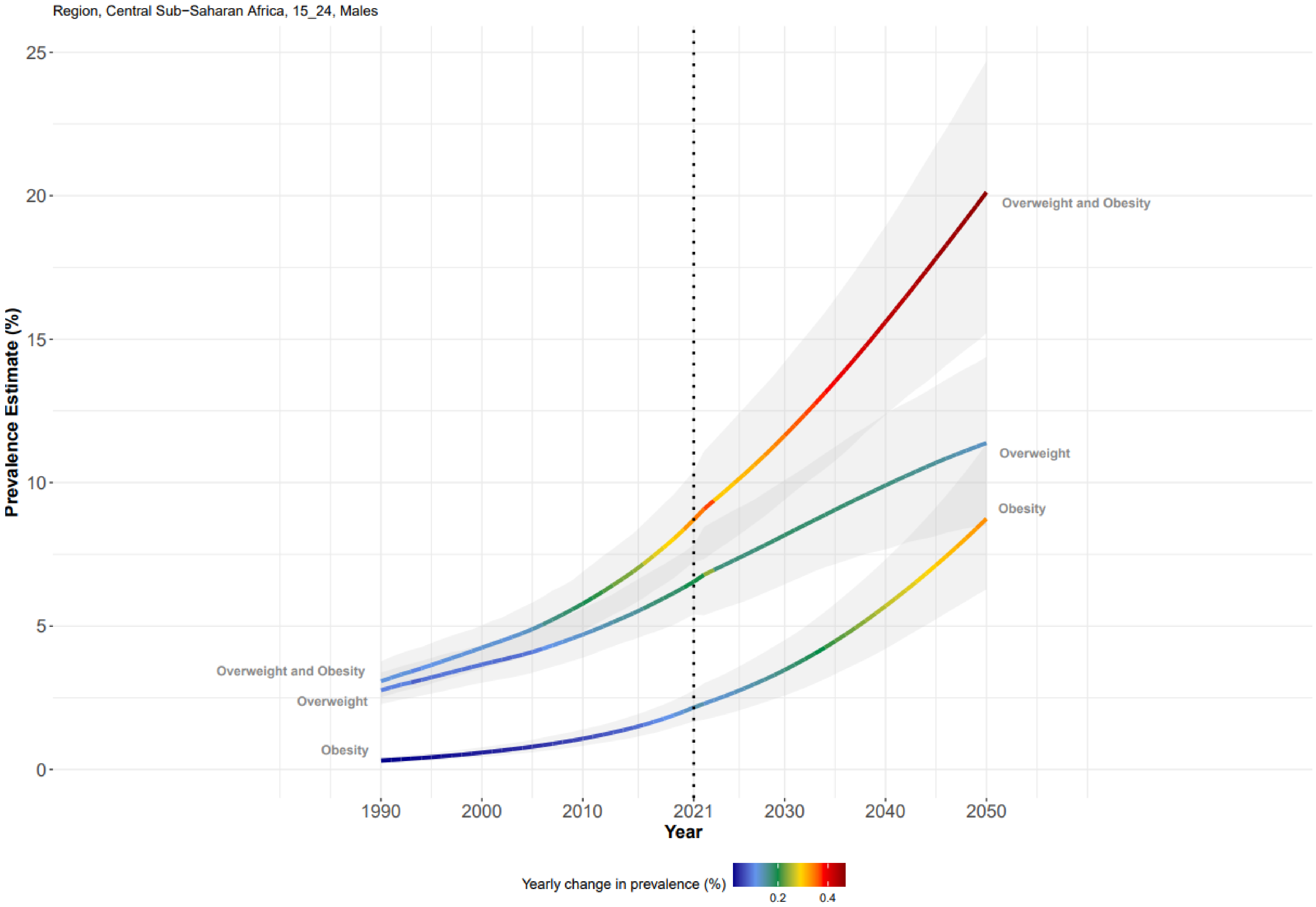
Region, Central Sub−Saharan Africa, 15_24, Males
Prevalence Estimate (%)
25
20
15
10
5
0
Overweight and Obesity
Overweight
Obesity
Overweight and Obesity
Overweight
Obesity
1990
2000
2010
2021
2030
2040
2050
Year
Yearly change in prevalence (%)
0.2
0.4

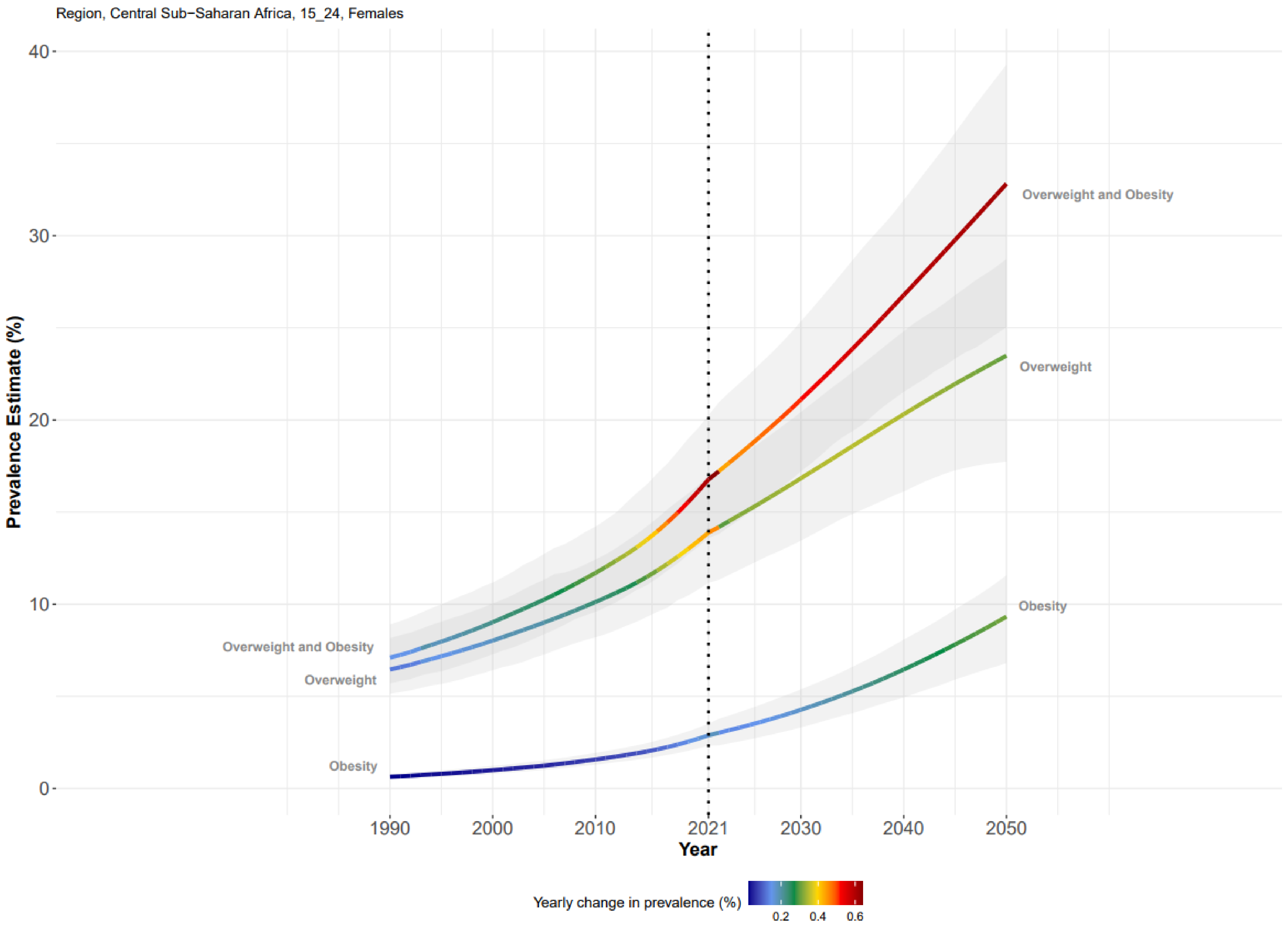
Region, Central Sub−Saharan Africa, 15_24, Females
40
30
20
10
0
Prevalence Estimate (%)
Overweight and Obesity
Overweight
Obesity
Overweight and Obesity
Overweight
Obesity
1990
2000
2010
2021
2030
2040
2050
Year
Yearly change in prevalence (%)
0.2
0.4
0.6

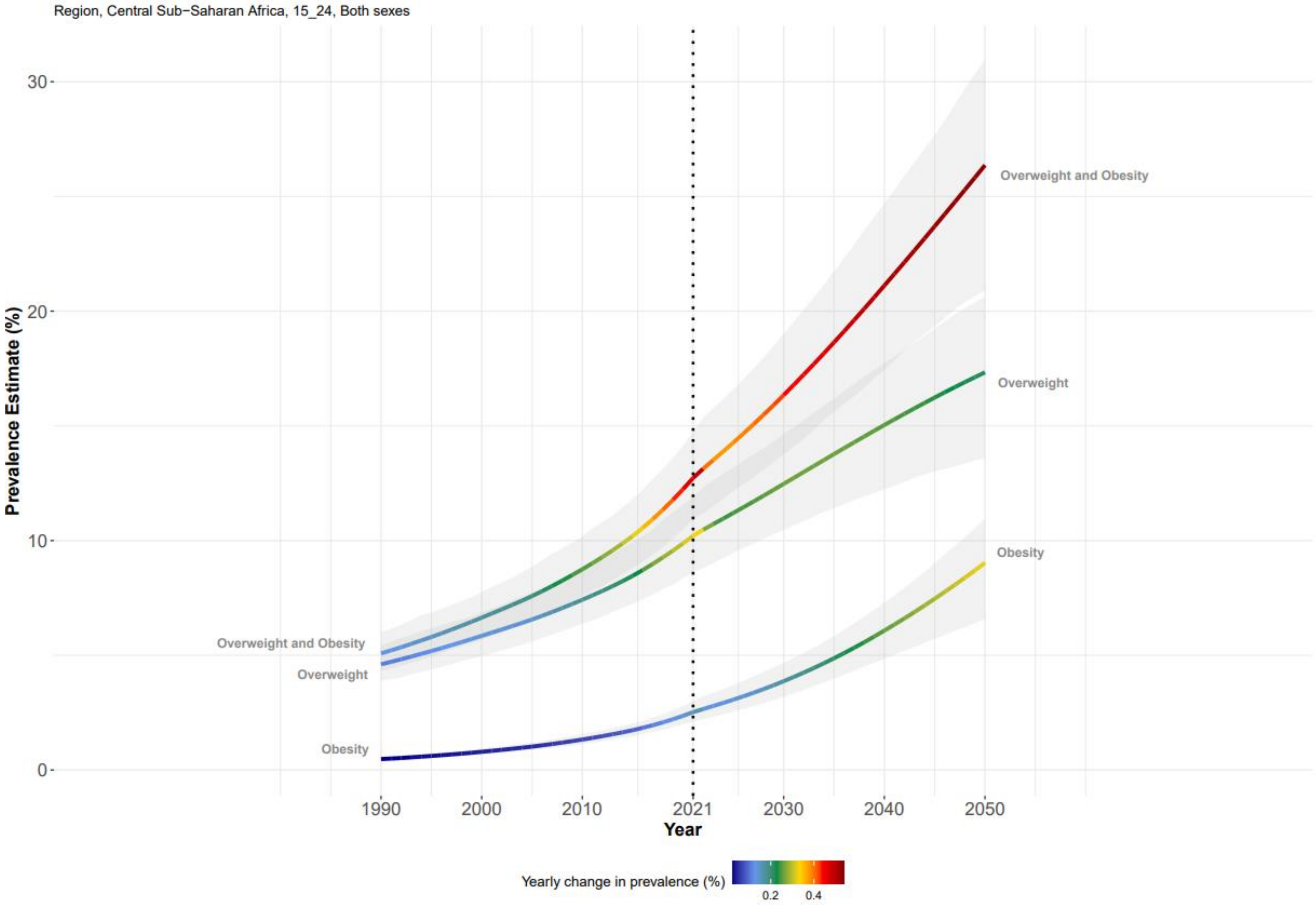

Region, Central Sub−Saharan Africa, 15_24, Both sexes
Prevalence Estimate (%)
30
20
10
0
Overweight and Obesity
Overweight
Obesity
Overweight and Obesity
Overweight
Obesity
1990
2000
2010
2021
2030
2040
2050
Year
Yearly change in prevalence (%)
0.2
0.4

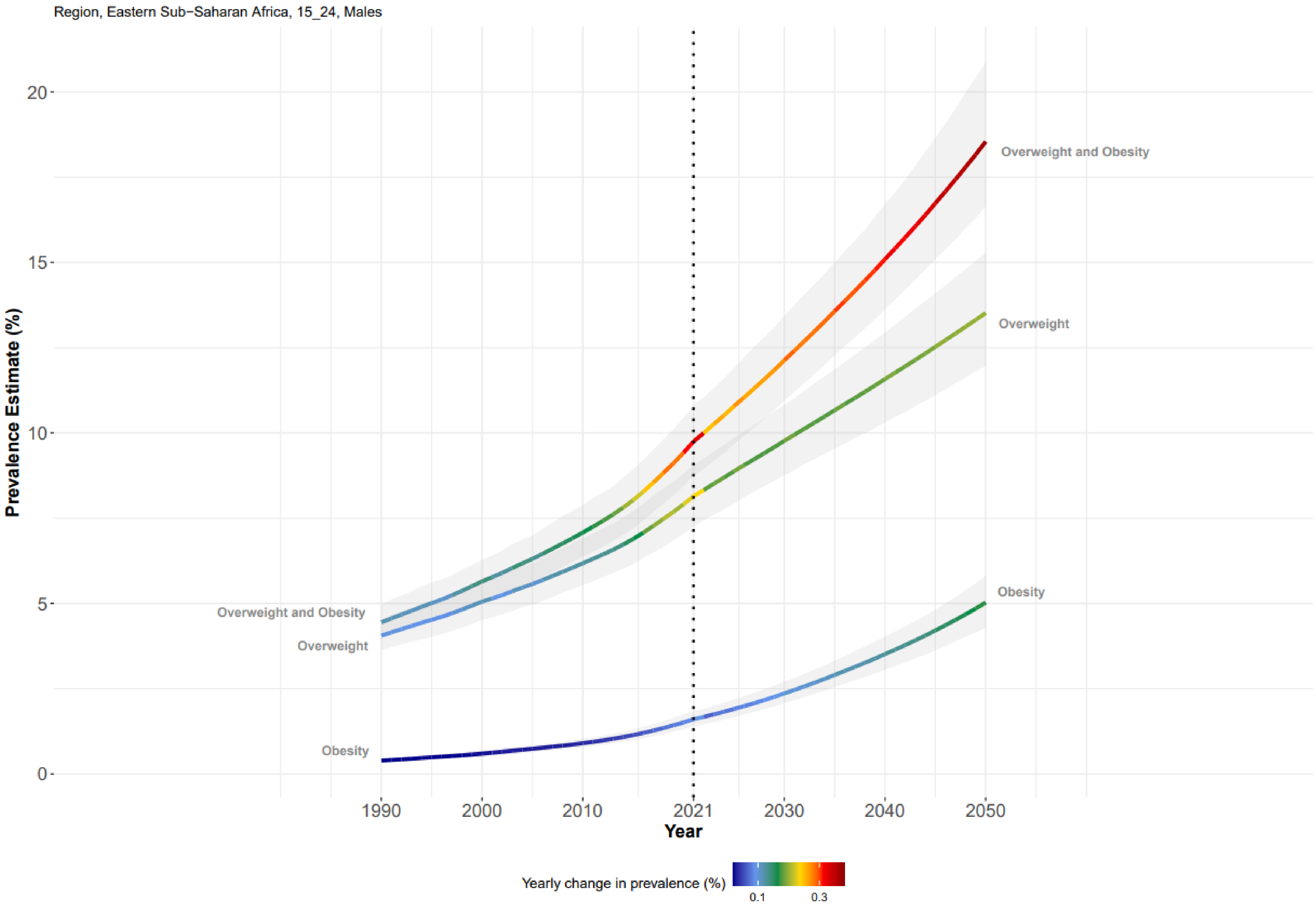

Region, Eastern Sub−Saharan Africa, 15_24, Males
Prevalence Estimate (%)
20
15
10
5
0
Overweight and Obesity
Overweight
Obesity
Overweight and Obesity
Overweight
Obesity
1990
2000
2010
2021
2030
2040
2050
Year
Yearly change in prevalence (%)
0.1
0.3

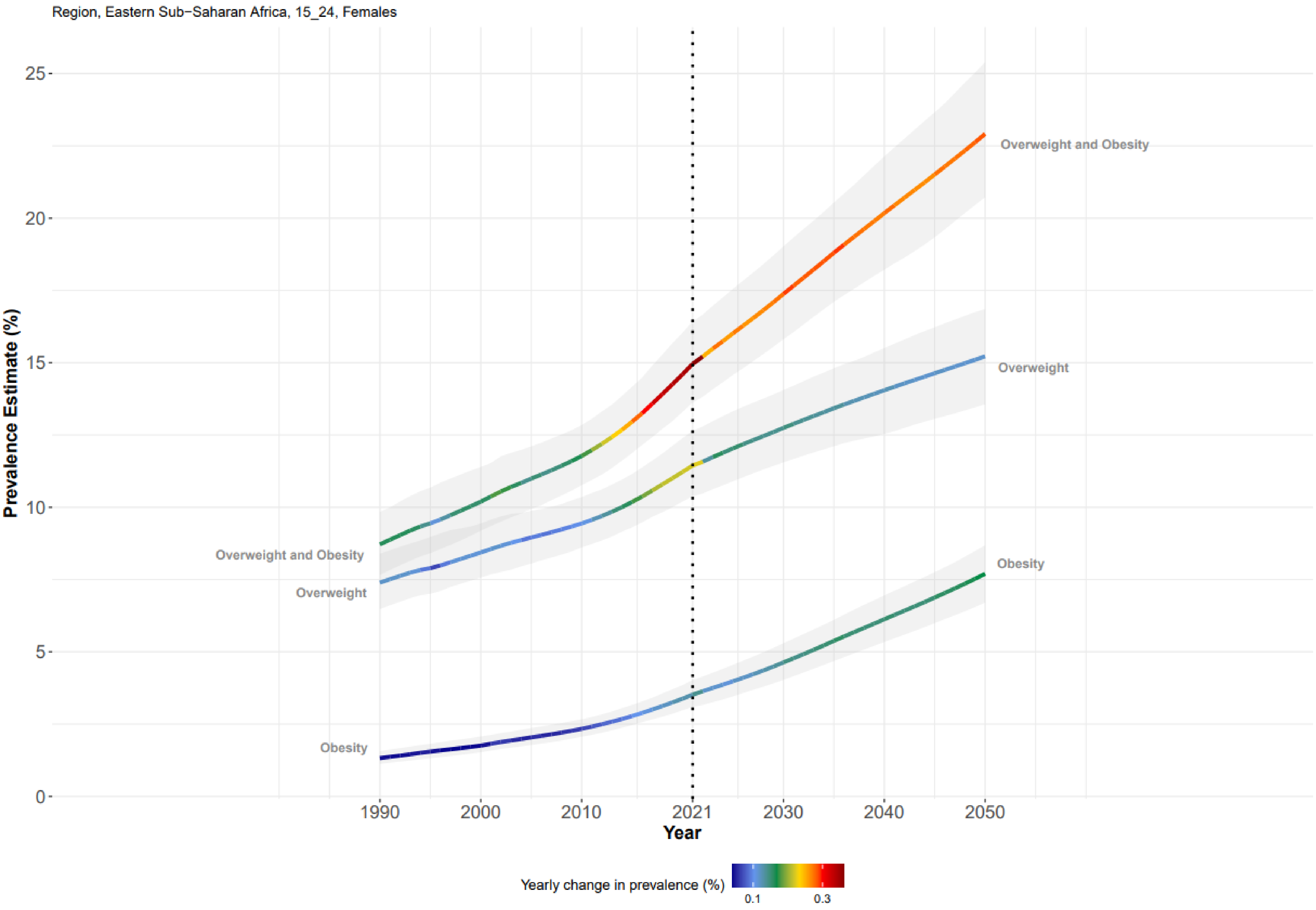
Region, Eastern Sub−Saharan Africa, 15_24, Females
Prevalence Estimate (%)
25
20
15
10
5
0
1990
2000
2010
2021
2030
2040
2050
Year
Overweight and Obesity
Overweight
Obesity
Overweight and Obesity
Overweight
Obesity
Yearly change in prevalence (%)
0.1
0.3

Region, Eastern Sub−Saharan Africa, 15_24, Both sexes

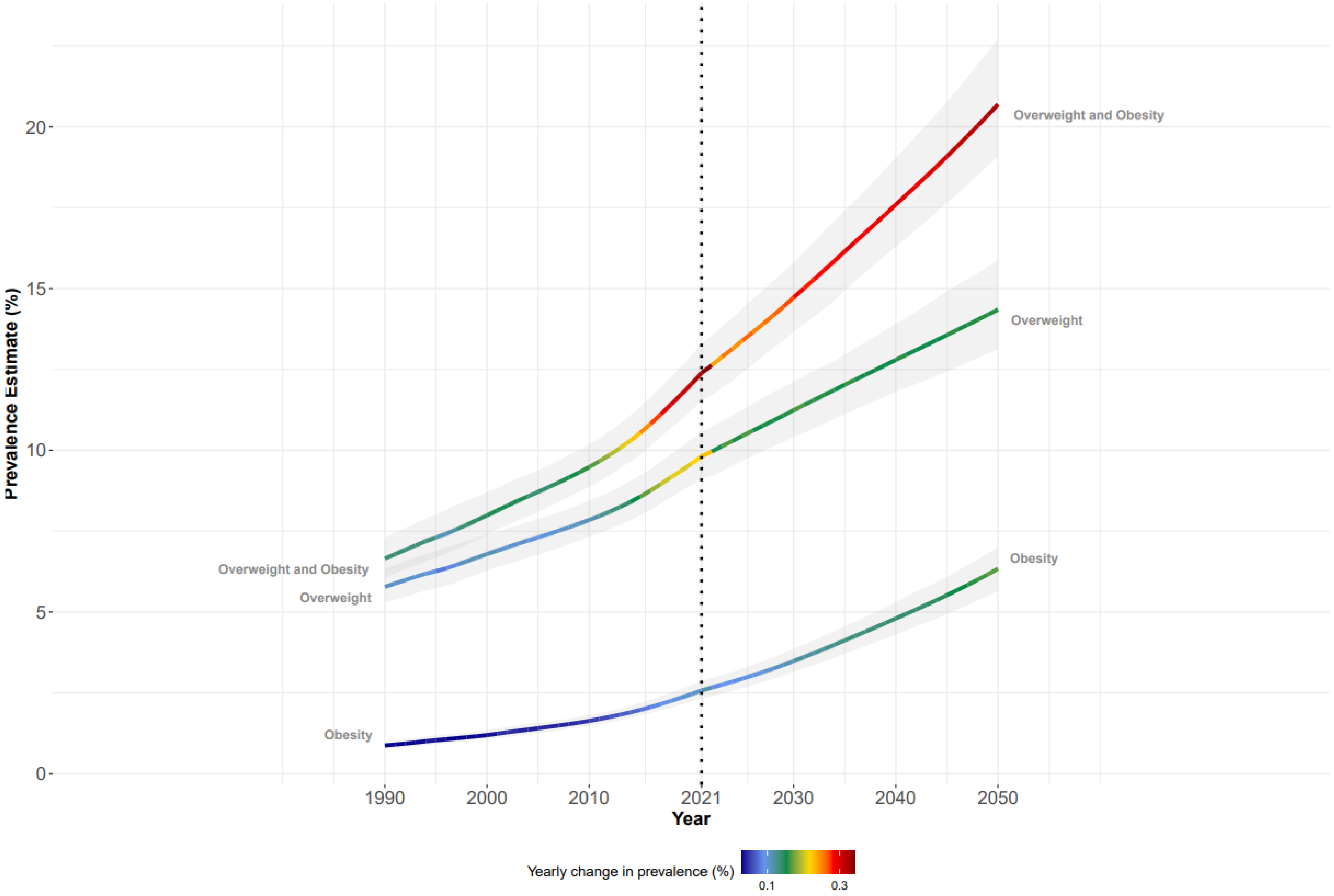

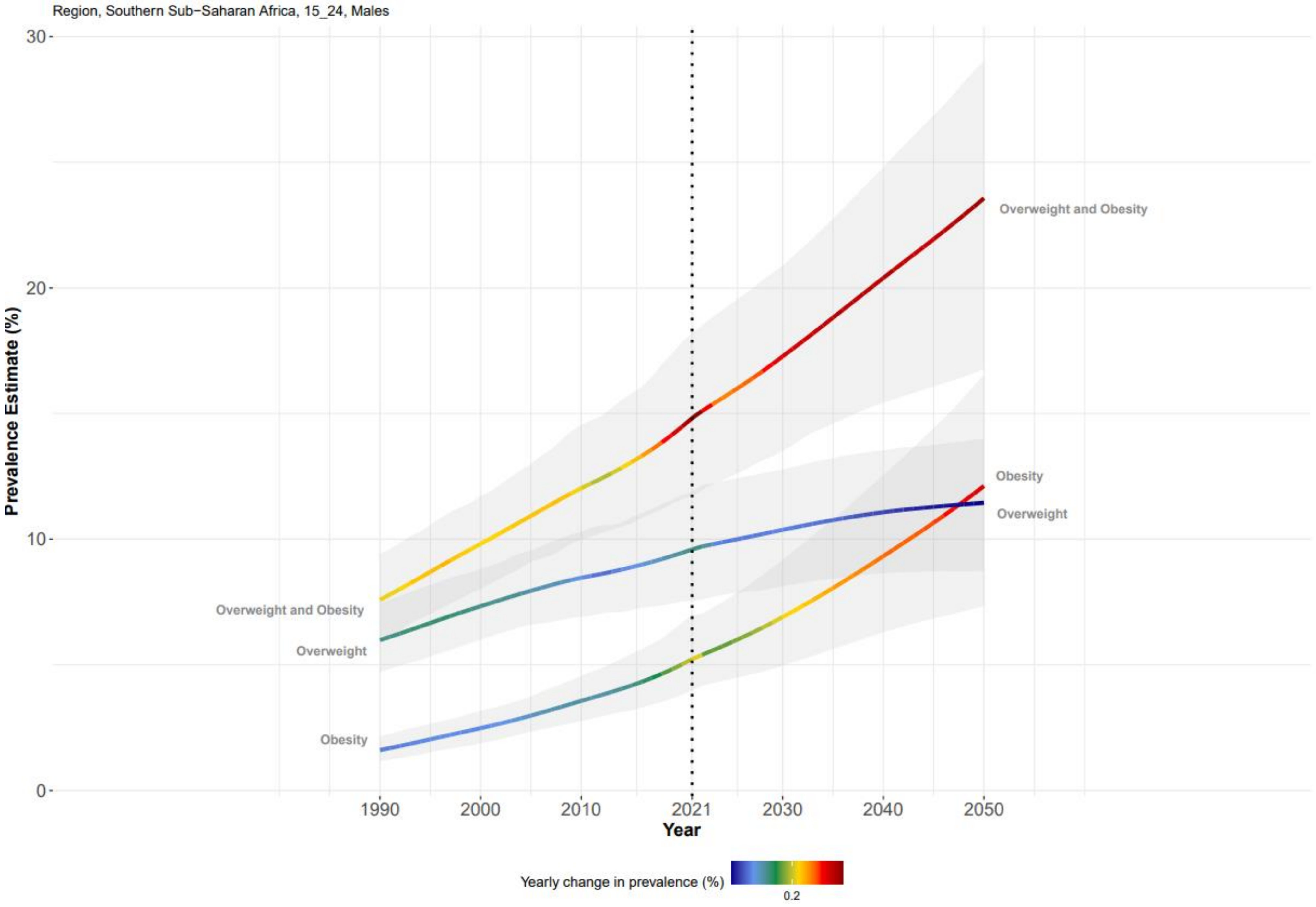

Region, Southern Sub−Saharan Africa, 15_24, Males
30
20
10
0
Prevalence Estimate (%)
Overweight and Obesity
Overweight
Obesity
Overweight and Obesity
Obesity
Overweight
1990
2000
2010
2021
2030
2040
2050
Year
Yearly change in prevalence (%)
0.2

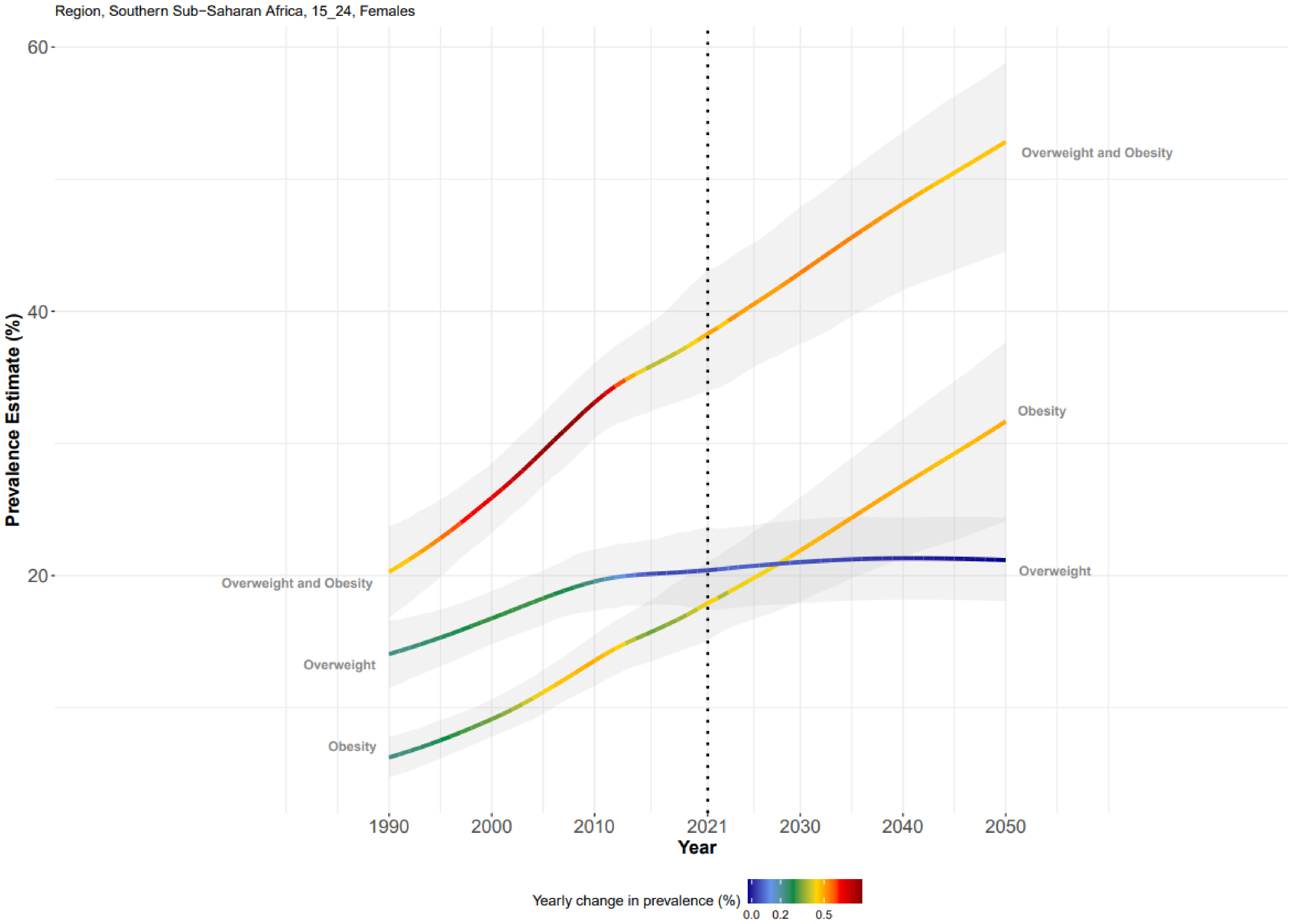
Region, Southern Sub−Saharan Africa, 15_24, Females
60
40
20
Prevalence Estimate (%)
Overweight and Obesity
Overweight
Obesity
Overweight and Obesity
Obesity
Overweight
1990
2000
2010
2021
2030
2040
2050
Year
Yearly change in prevalence (%)
0.0
0.2
0.5

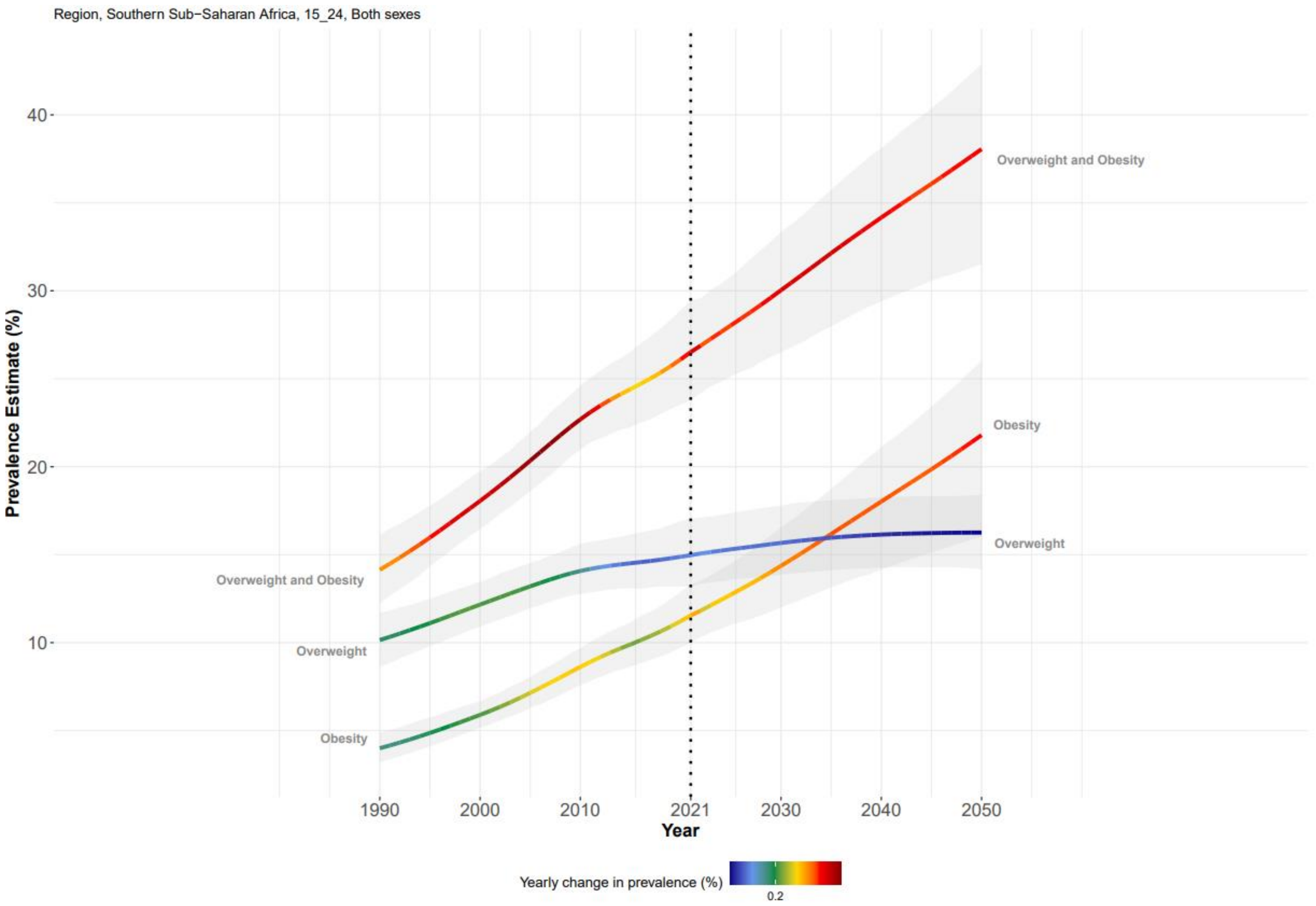

Region, Southern Sub−Saharan Africa, 15_24, Both sexes
Prevalence Estimate (%)
40
30
20
10
Overweight and Obesity
Obesity
Overweight
Overweight and Obesity
Overweight
Obesity
1990
2000
2010
2021
2030
2040
2050
Year
Yearly change in prevalence (%)
0.2

Region, Western Sub−Saharan Africa, 15_24, Males

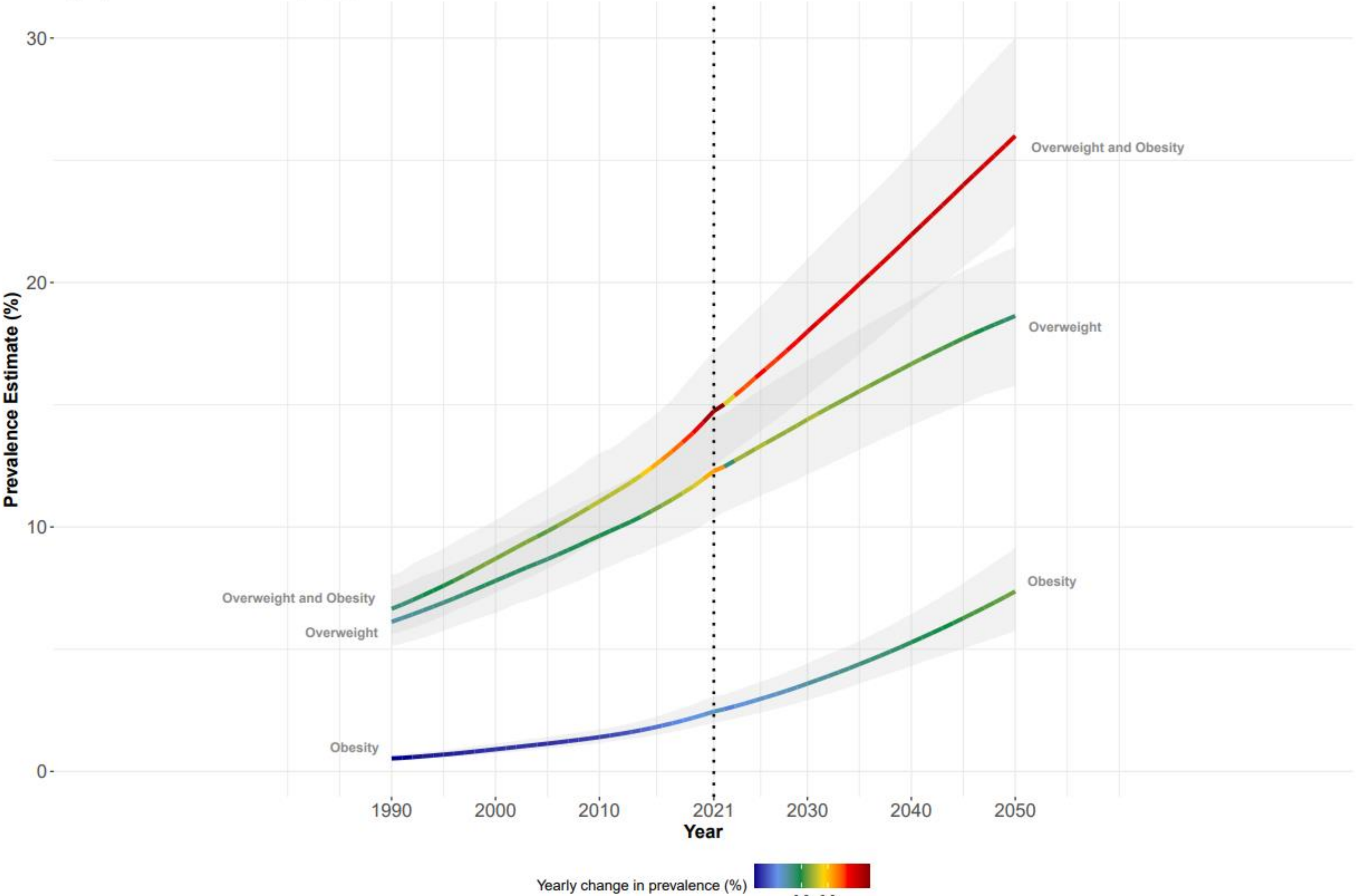

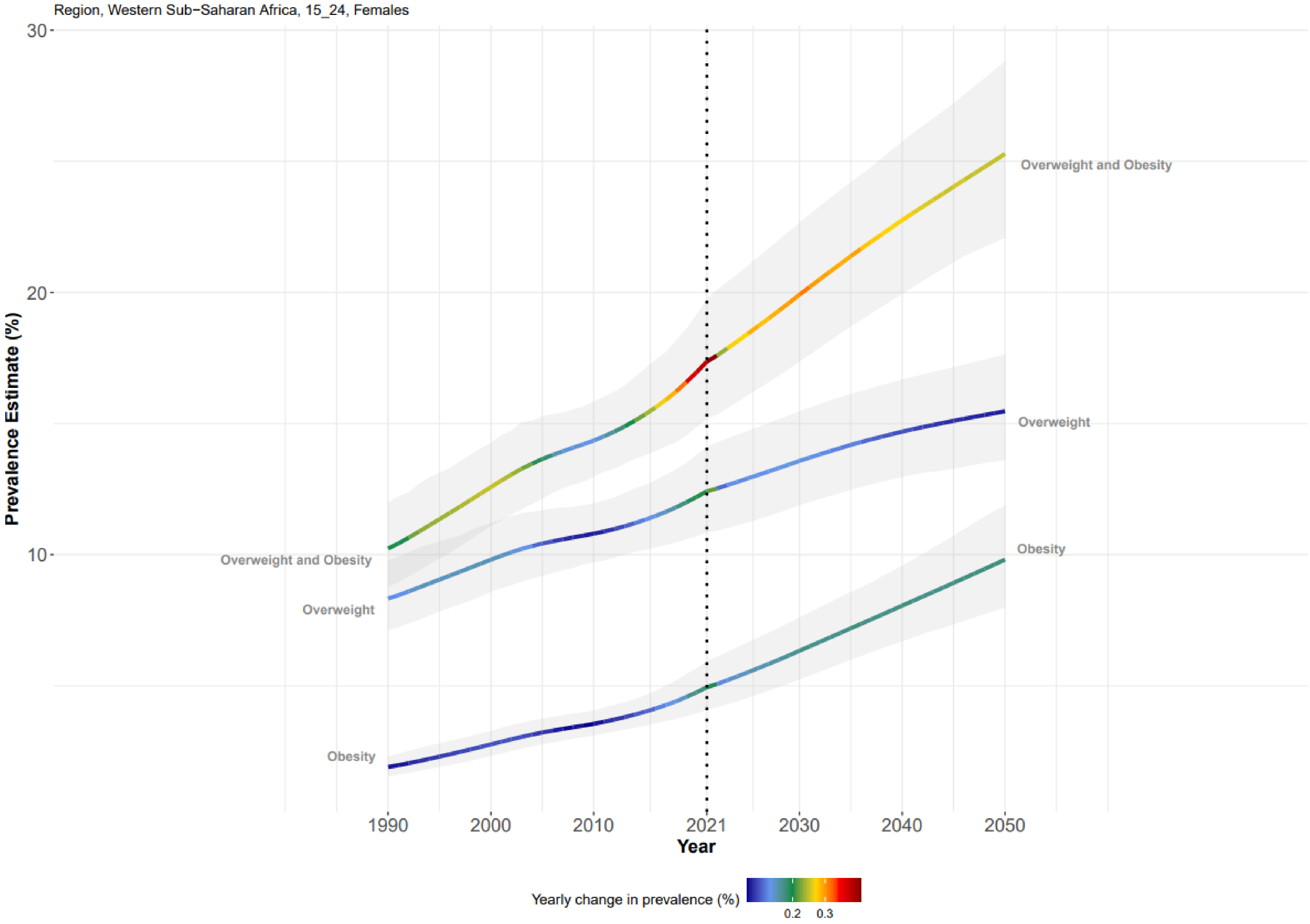

Region, Western Sub−Saharan Africa, 15_24, Females
Prevalence Estimate (%)
30
20
10
Overweight and Obesity
Overweight
Obesity
Overweight and Obesity
Overweight
Obesity
1990
2000
2010
2021
2030
2040
2050
Year
Yearly change in prevalence (%)
0.2
0.3

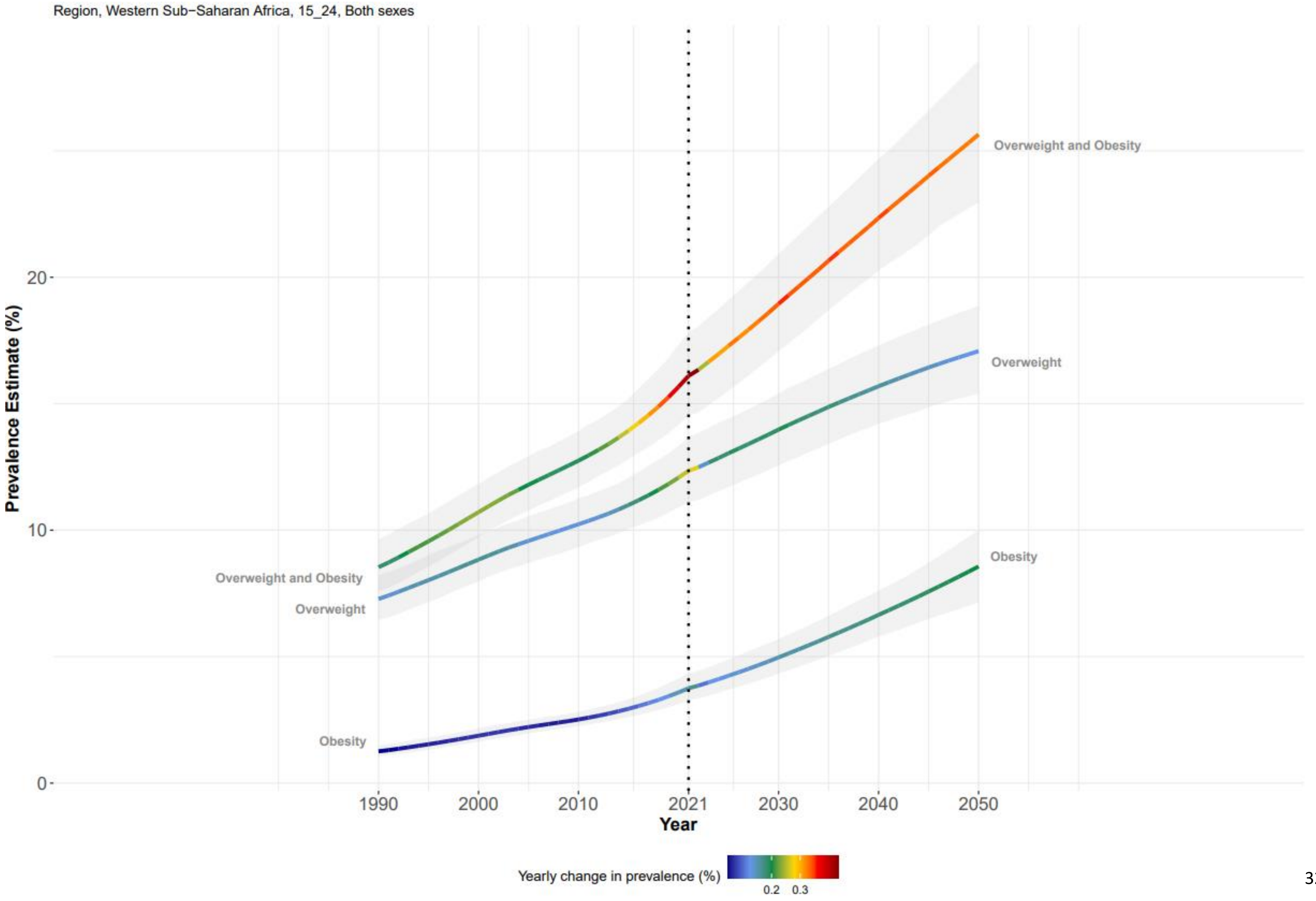

Region, Western Sub−Saharan Africa, 15_24, Both sexes
Prevalence Estimate (%)
20
10
0
1990
2000
2010
2021
2030
2040
2050
Year
Overweight and Obesity
Overweight
Obesity
Overweight and Obesity
Overweight
Obesity
Yearly change in prevalence (%)
0.2
0.3

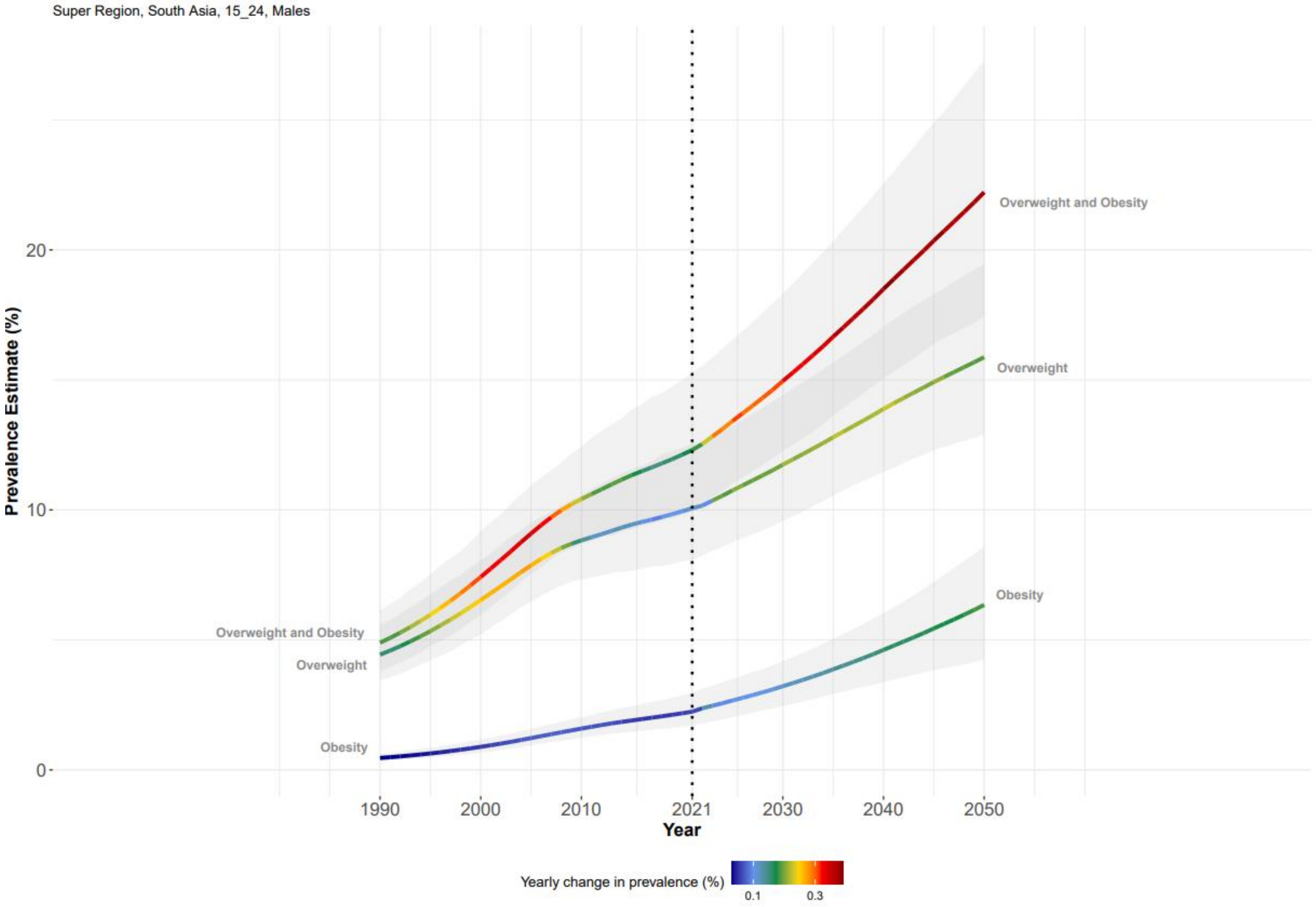
Super Region, South Asia, 15_24, Males
Prevalence Estimate (%)
0
10
20
1990
2000
2010
2021
2030
2040
2050
Year
Overweight and Obesity
Overweight
Obesity
Overweight and Obesity
Overweight
Obesity
Yearly change in prevalence (%)
0.1
0.3

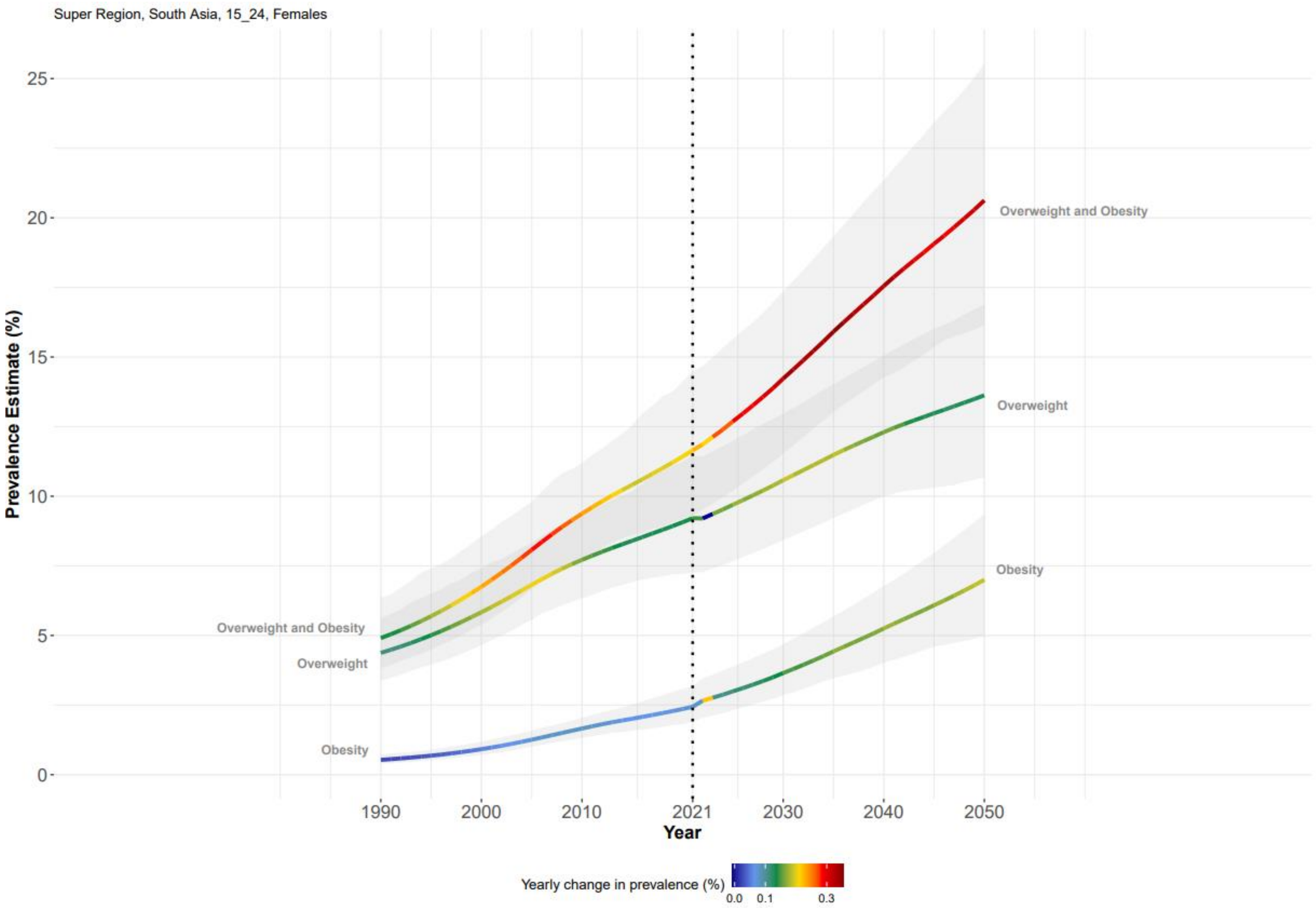

Super Region, South Asia, 15_24, Females
Prevalence Estimate (%)
25
20
15
10
5
0
1990
2000
2010
2021
2030
2040
2050
Year
Overweight and Obesity
Overweight
Obesity
Overweight and Obesity
Overweight
Obesity
Yearly change in prevalence (%)
0.0
0.1
0.3

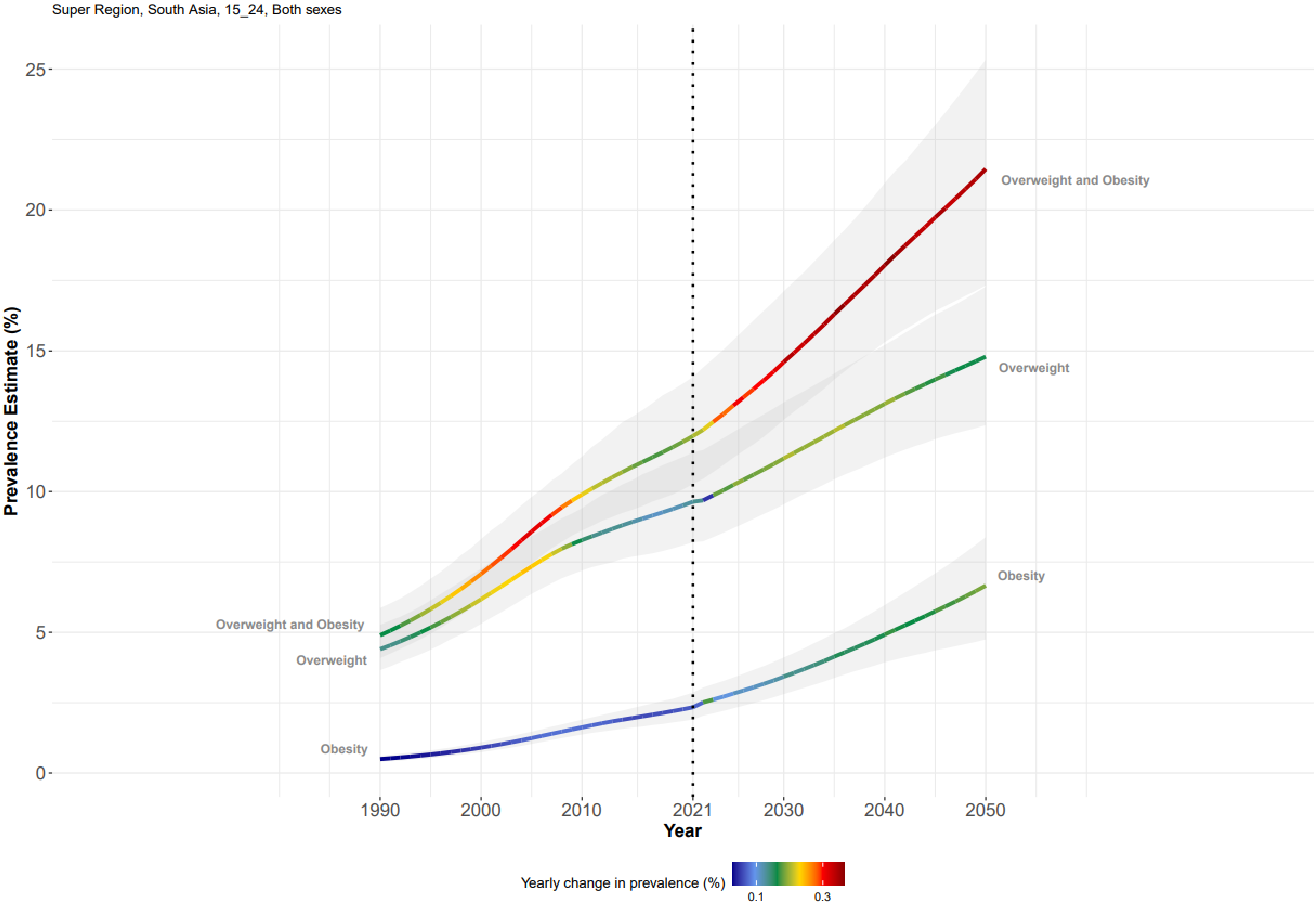
Super Region, South Asia, 15_24, Both sexes
Prevalence Estimate (%)
25
20
15
10
5
0
1990
2000
2010
2021
2030
2040
2050
Year
Overweight and Obesity
Overweight
Obesity
Overweight and Obesity
Overweight
Obesity
Yearly change in prevalence (%)
0.1
0.3

Figure S4: Ratio of overweight to obesity 1990-2050 for children and young adolescents 5-14 years old by sex and country

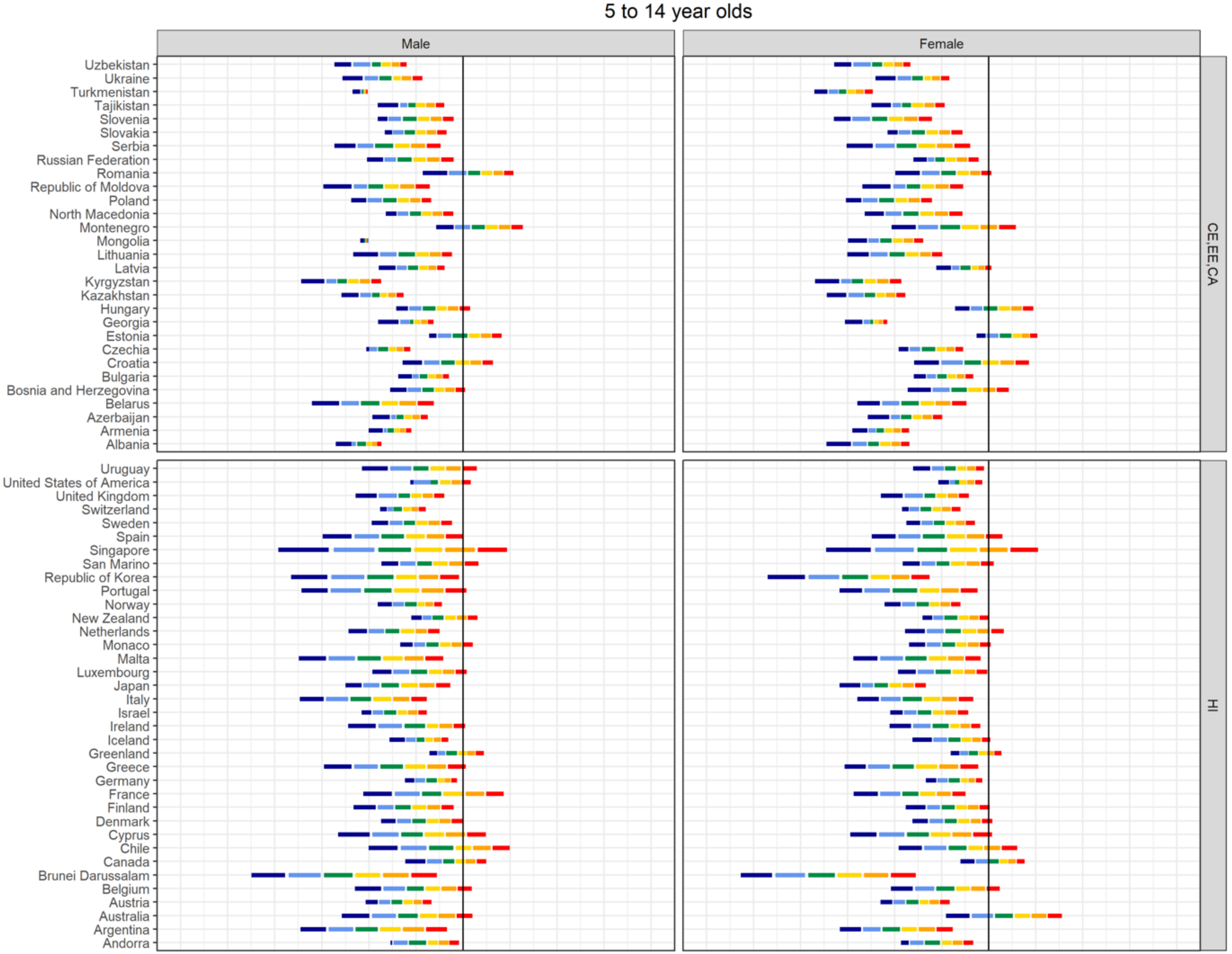

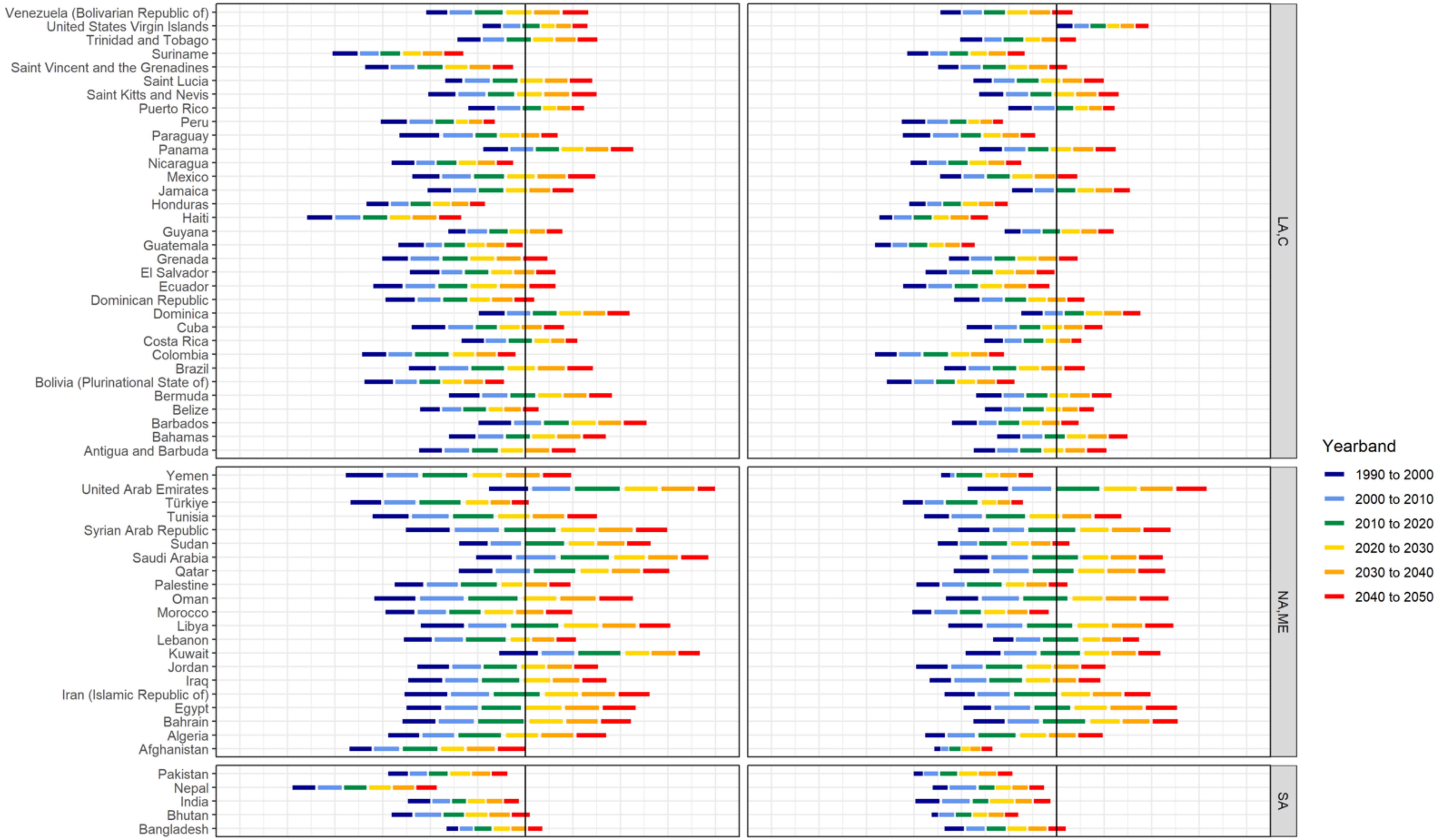
Venezuela (Bolivarian Republic of)
United States Virgin Islands
Trinidad and Tobago
Suriname
Saint Vincent and the Grenadines
Saint Lucia
Saint Kitts and Nevis
Puerto Rico
Peru
Paraguay
Panama
Nicaragua
Mexico
Jamaica
Honduras
Haiti
Guyana
Guatemala
Grenada
El Salvador
Ecuador
Dominican Republic
Dominica
Cuba
Costa Rica
Colombia
Brazil
Bolivia (Plurinational State of)
Bermuda
Belize
Barbados
Bahamas
Antigua and Barbuda
Yemen
United Arab Emirates
Türkiye
Tunisia
Syrian Arab Republic
Sudan
Saudi Arabia
Qatar
Palestine
Oman
Morocco
Libya
Lebanon
Kuwait
Jordan
Iraq
Iran (Islamic Republic of)
Egypt
Bahrain
Algeria
Afghanistan
Pakistan
Nepal
India
Bhutan
Bangladesh
LA,C
NA,ME
SA
Yearband
1990 to 2000
2000 to 2010
2010 to 2020
2020 to 2030
2030 to 2040
2040 to 2050

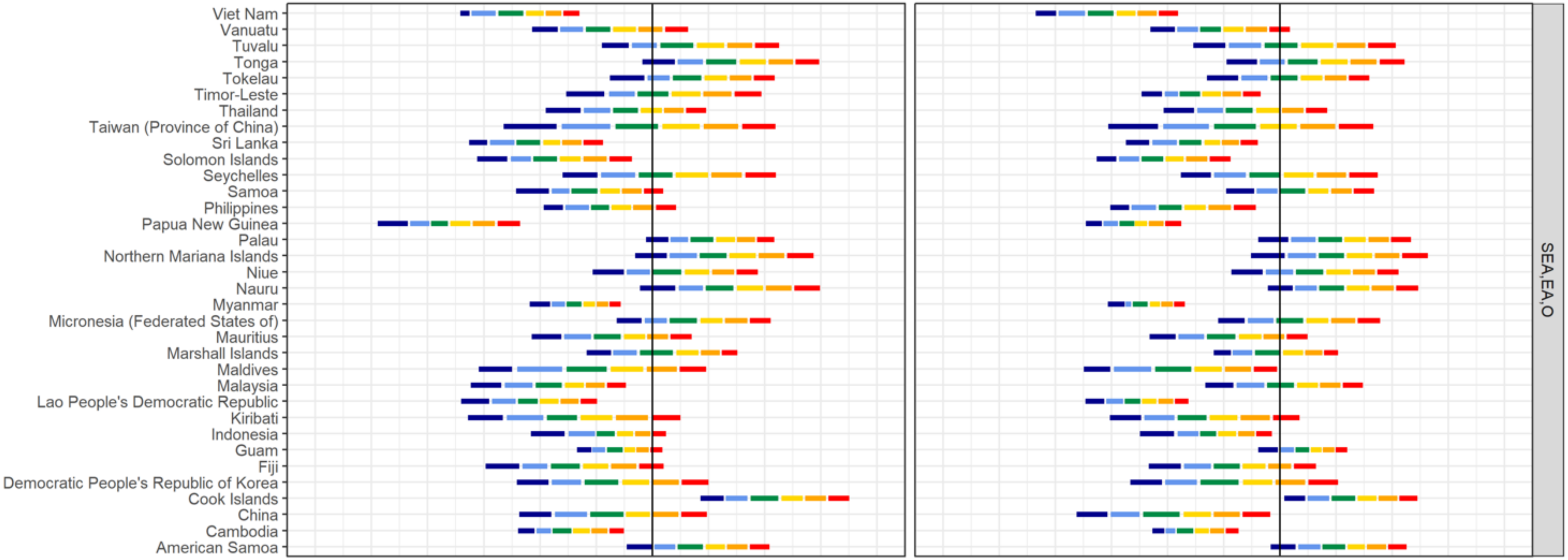
Viet Nam
Vanuatu
Tuvalu
Tonga
Tokelau
Timor-Leste
Thailand
Taiwan (Province of China)
Sri Lanka
Solomon Islands
Seychelles
Samoa
Philippines
Papua New Guinea
Palau
Northern Mariana Islands
Niue
Nauru
Myanmar
Micronesia (Federated States of)
Mauritius
Marshall Islands
Maldives
Malaysia
Lao People's Democratic Republic
Kiribati
Indonesia
Guam
Fiji
Democratic People's Republic of Korea
Cook Islands
China
Cambodia
American Samoa
SEA,EA,O

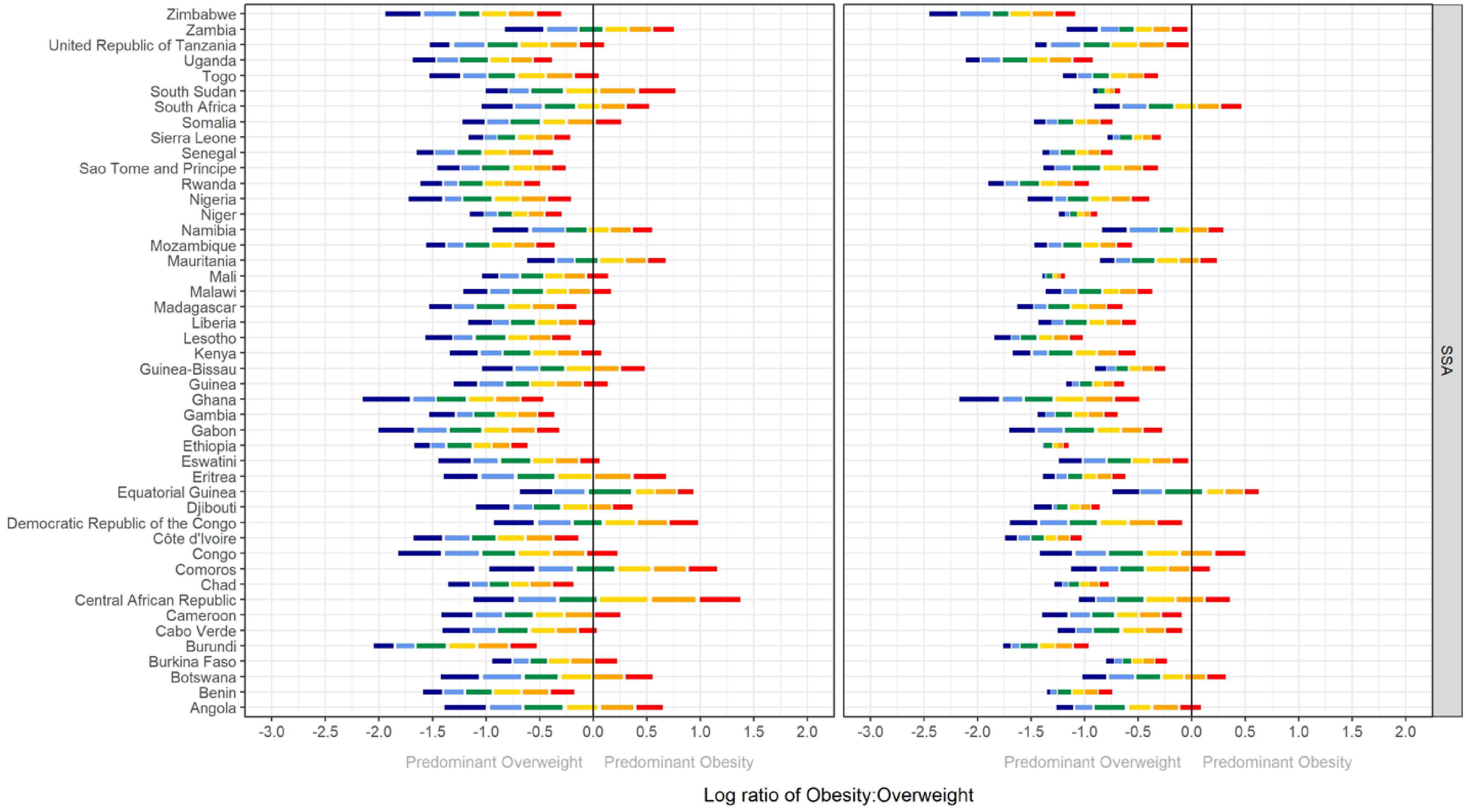
Zimbabwe
Zambia
United Republic of Tanzania
Uganda
Togo
South Sudan
South Africa
Somalia
Sierra Leone
Senegal
Sao Tome and Principe
Rwanda
Nigeria
Niger
Namibia
Mozambique
Mauritania
Mali
Malawi
Madagascar
Liberia
Lesotho
Kenya
Guinea-Bissau
Guinea
Ghana
Gambia
Gabon
Ethiopia
Eswatini
Eritrea
Equatorial Guinea
Djibouti
Democratic Republic of the Congo
Côte d'Ivoire
Congo
Comoros
Chad
Central African Republic
Cameroon
Cabo Verde
Burundi
Burkina Faso
Botswana
Benin
Angola
-3.0 -2.5 -2.0 -1.5 -1.0 -0.5 0.0 0.5 1.0 1.5 2.0
Predominant Overweight
Predominant Obesity
SSA
Log ratio of Obesity:Overweight

Figure S5: Ratio of overweight to obesity 1990-2050 for older adolescents 15-24 years old by sex and country

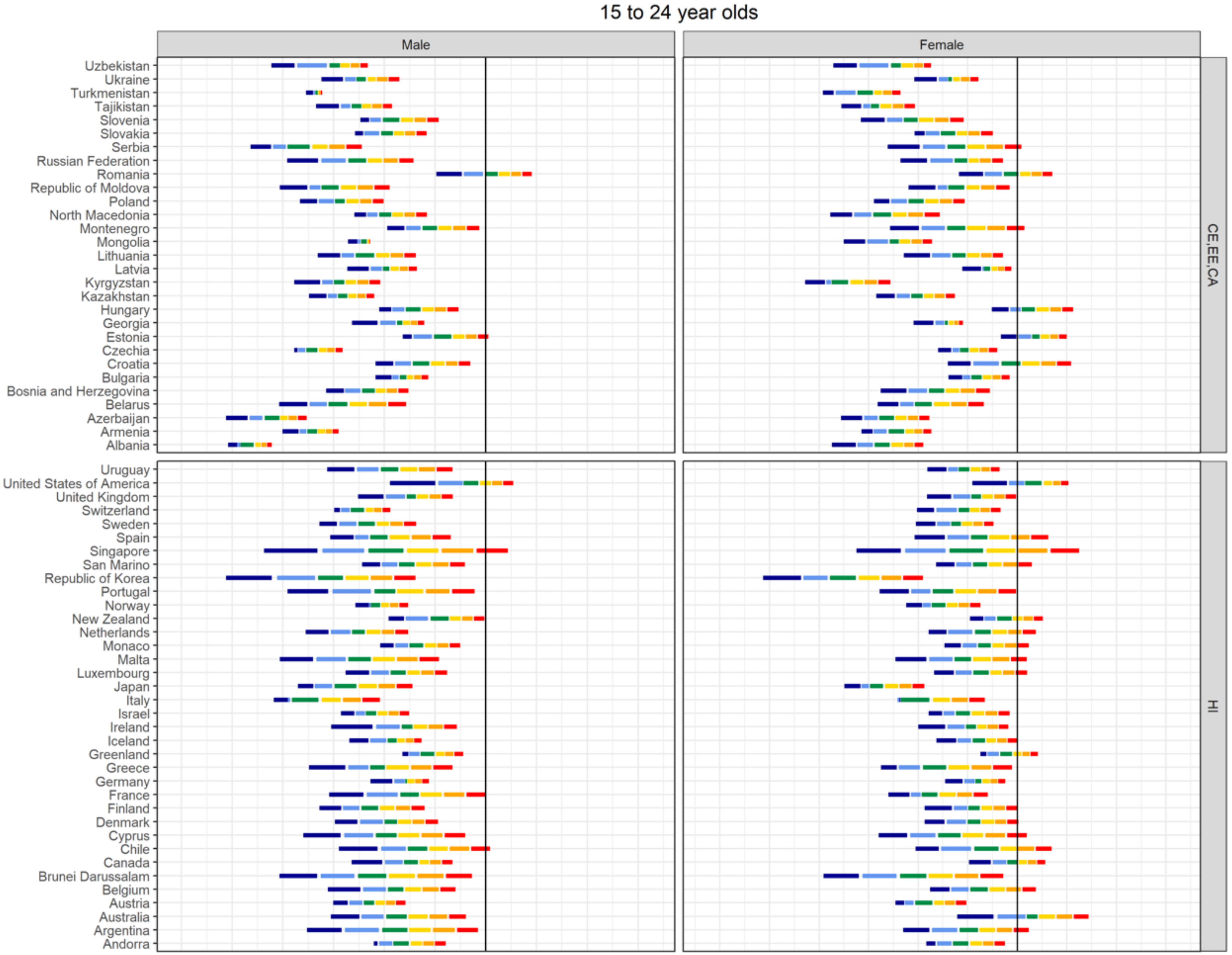

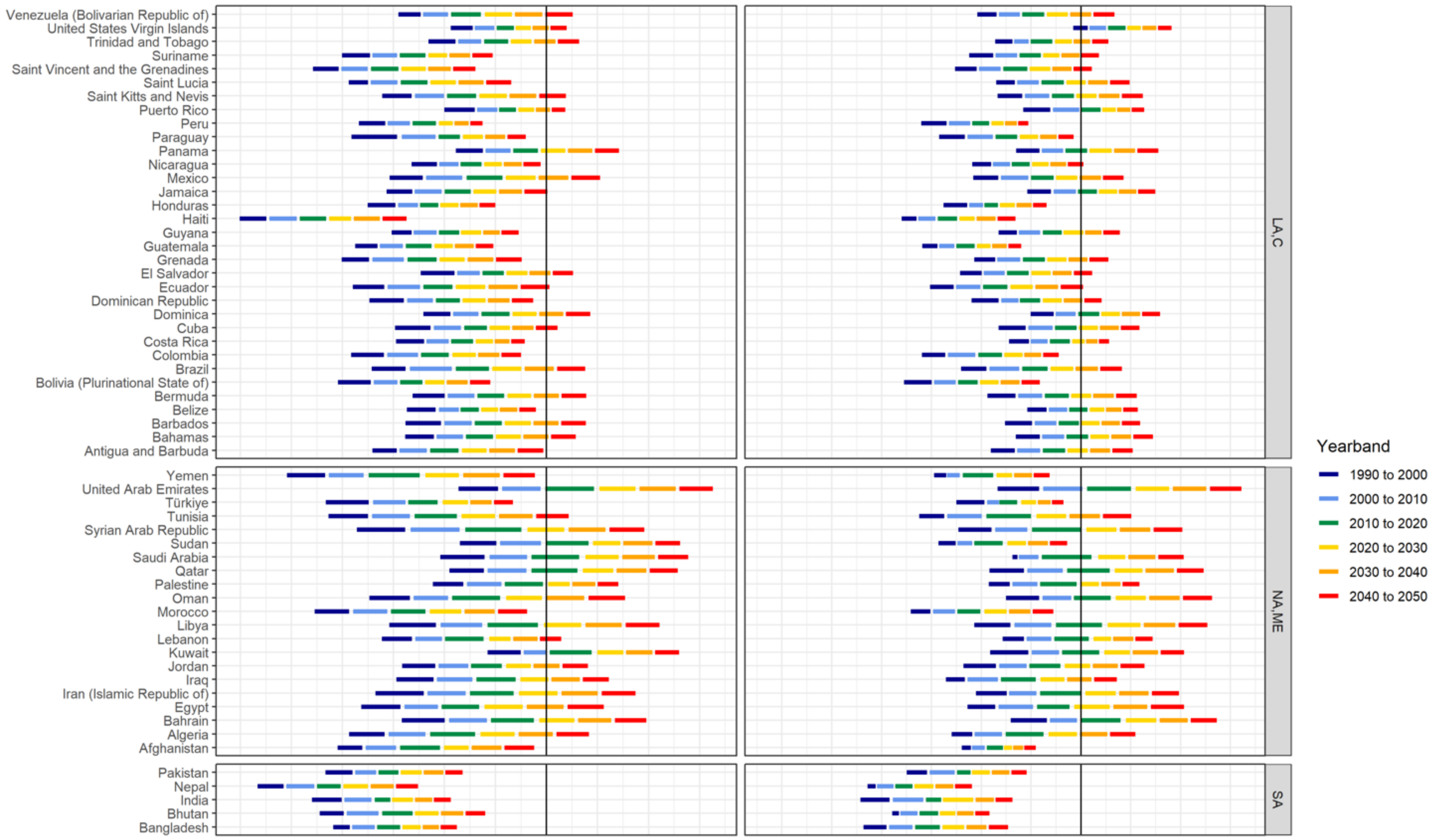

Venezuela (Bolivarian Republic of)
United States Virgin Islands
Trinidad and Tobago
Suriname
Saint Vincent and the Grenadines
Saint Lucia
Saint Kitts and Nevis
Puerto Rico
Peru
Paraguay
Panama
Nicaragua
Mexico
Jamaica
Honduras
Haiti
Guyana
Guatemala
Grenada
El Salvador
Ecuador
Dominican Republic
Dominica
Cuba
Costa Rica
Colombia
Brazil
Bolivia (Plurinational State of)
Bermuda
Belize
Barbados
Bahamas
Antigua and Barbuda
LA,C
Yemen
United Arab Emirates
Türkiye
Tunisia
Syrian Arab Republic
Sudan
Saudi Arabia
Qatar
Palestine
Oman
Morocco
Libya
Lebanon
Kuwait
Jordan
Iraq
Iran (Islamic Republic of)
Egypt
Bahrain
Algeria
Afghanistan
NA,ME
Pakistan
Nepal
India
Bhutan
Bangladesh
SA
Yearband
1990 to 2000
2000 to 2010
2010 to 2020
2020 to 2030
2030 to 2040
2040 to 2050

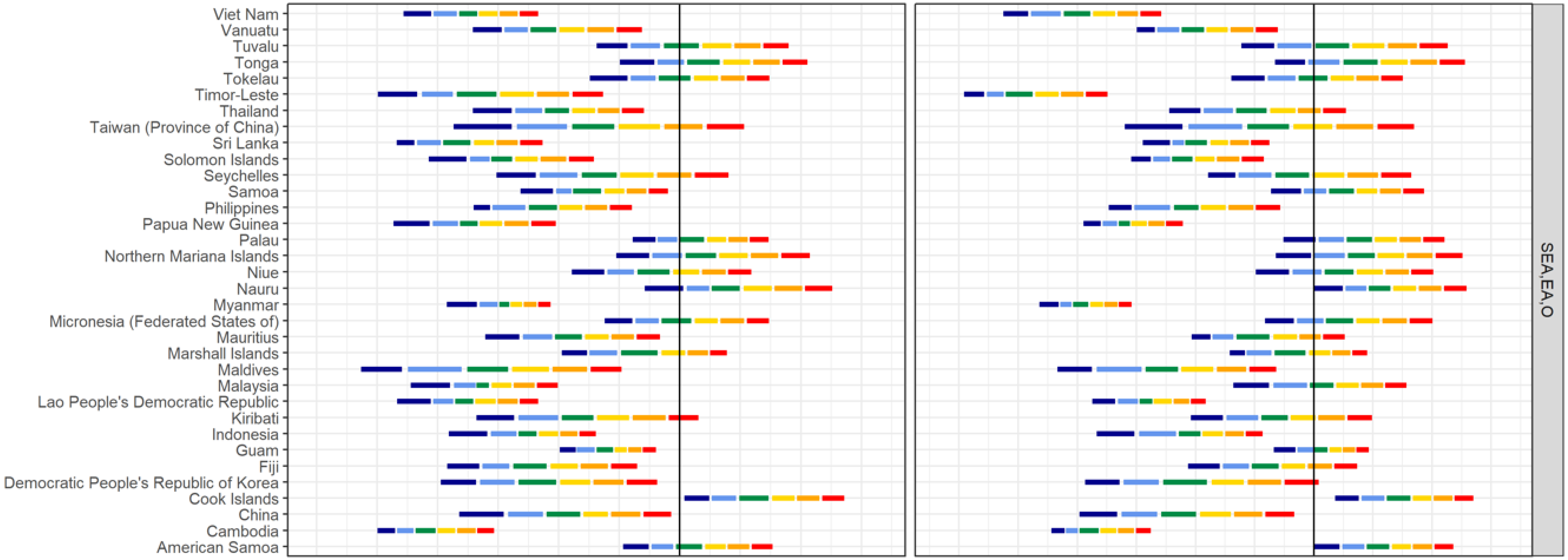

Viet Nam
Vanuatu
Tuvalu
Tonga
Tokelau
Timor-Leste
Thailand
Taiwan (Province of China)
Sri Lanka
Solomon Islands
Seychelles
Samoa
Philippines
Papua New Guinea
Palau
Northern Mariana Islands
Niue
Nauru
Myanmar
Micronesia (Federated States of)
Mauritius
Marshall Islands
Maldives
Malaysia
Lao People's Democratic Republic
Kiribati
Indonesia
Guam
Fiji
Democratic People's Republic of Korea
Cook Islands
China
Cambodia
American Samoa
SEA,EA,O

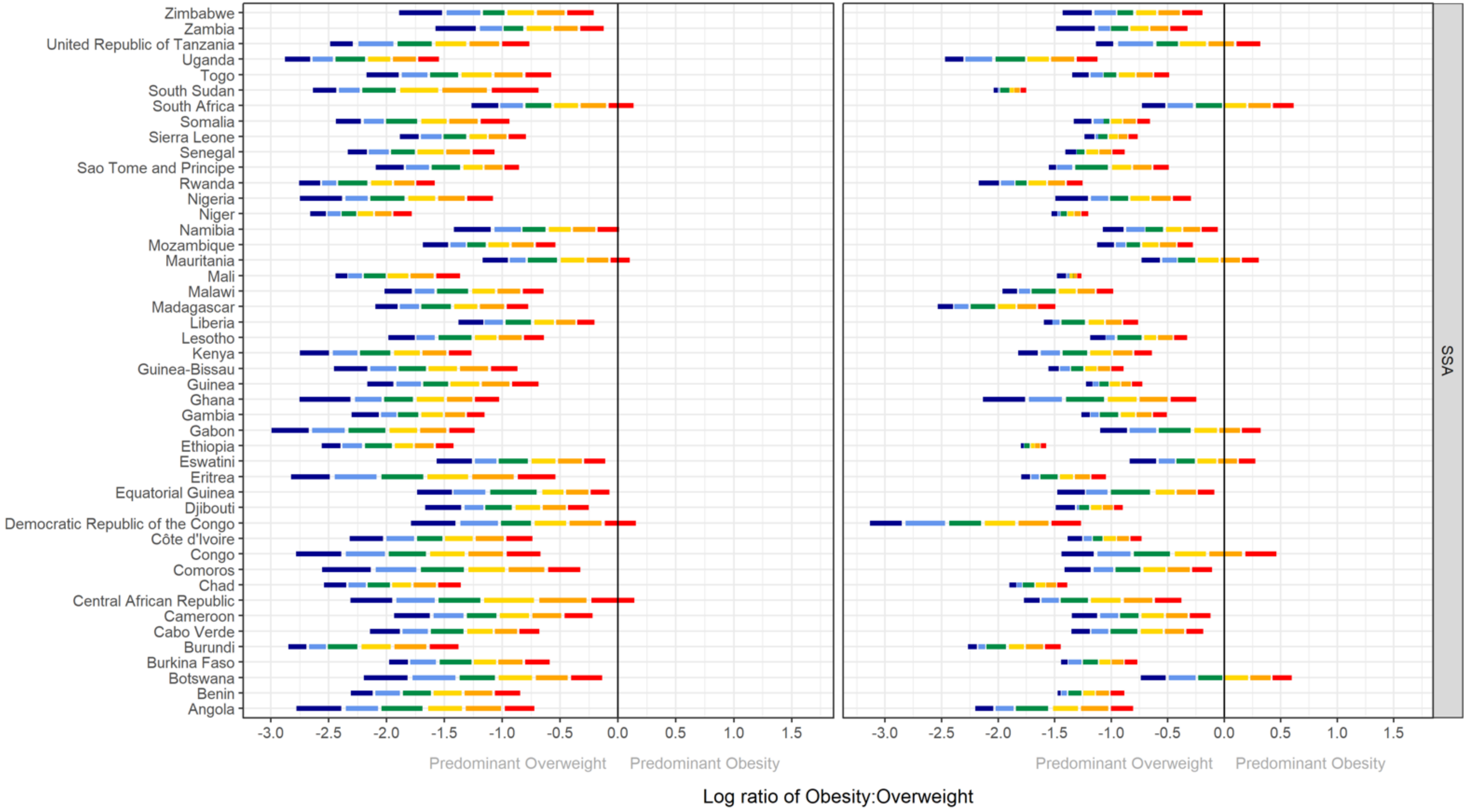
Zimbabwe
Zambia
United Republic of Tanzania
Uganda
Togo
South Sudan
South Africa
Somalia
Sierra Leone
Senegal
Sao Tome and Principe
Rwanda
Nigeria
Niger
Namibia
Mozambique
Mauritania
Mali
Malawi
Madagascar
Liberia
Lesotho
Kenya
Guinea-Bissau
Guinea
Ghana
Gambia
Gabon
Ethiopia
Eswatini
Eritrea
Equatorial Guinea
Djibouti
Democratic Republic of the Congo
Côte d'Ivoire
Congo
Comoros
Chad
Central African Republic
Cameroon
Cabo Verde
Burundi
Burkina Faso
Botswana
Benin
Angola
SSA
-3.0 -2.5 -2.0 -1.5 -1.0 -0.5 0.0 0.5 1.0 1.5
Predominant Overweight
Predominant Obesity
Log ratio of Obesity:Overweight

Figure S6: Ternary diagram of global change (1990-2050) in obesity, overweight, non-overweight prevalence, by age and sex

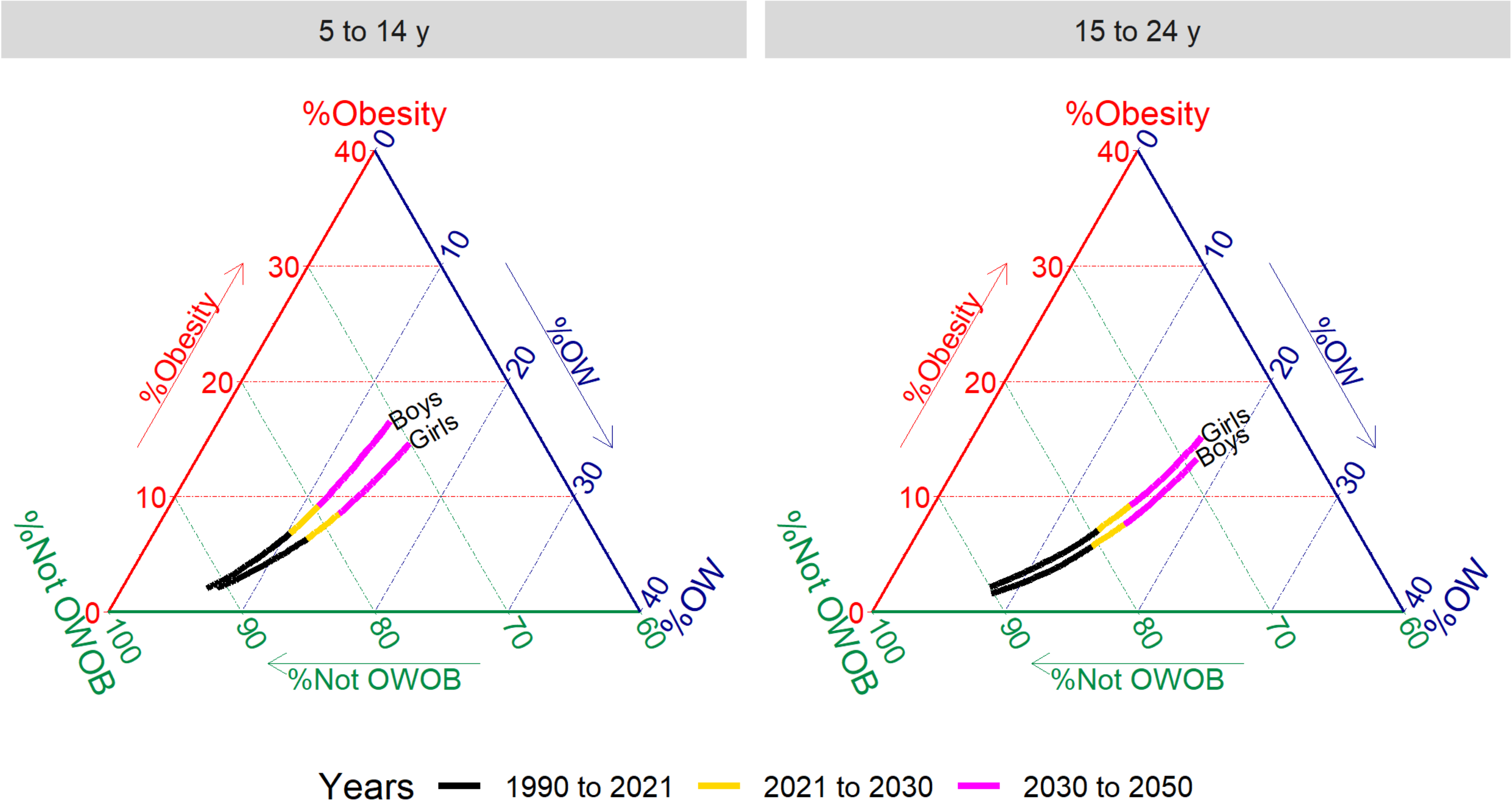

Figure S7: Ternary diagram of change (1990, 2021, 2030, and 2050) in obesity, overweight, non-overweight/obesity prevalence, by age, sex, and super-region

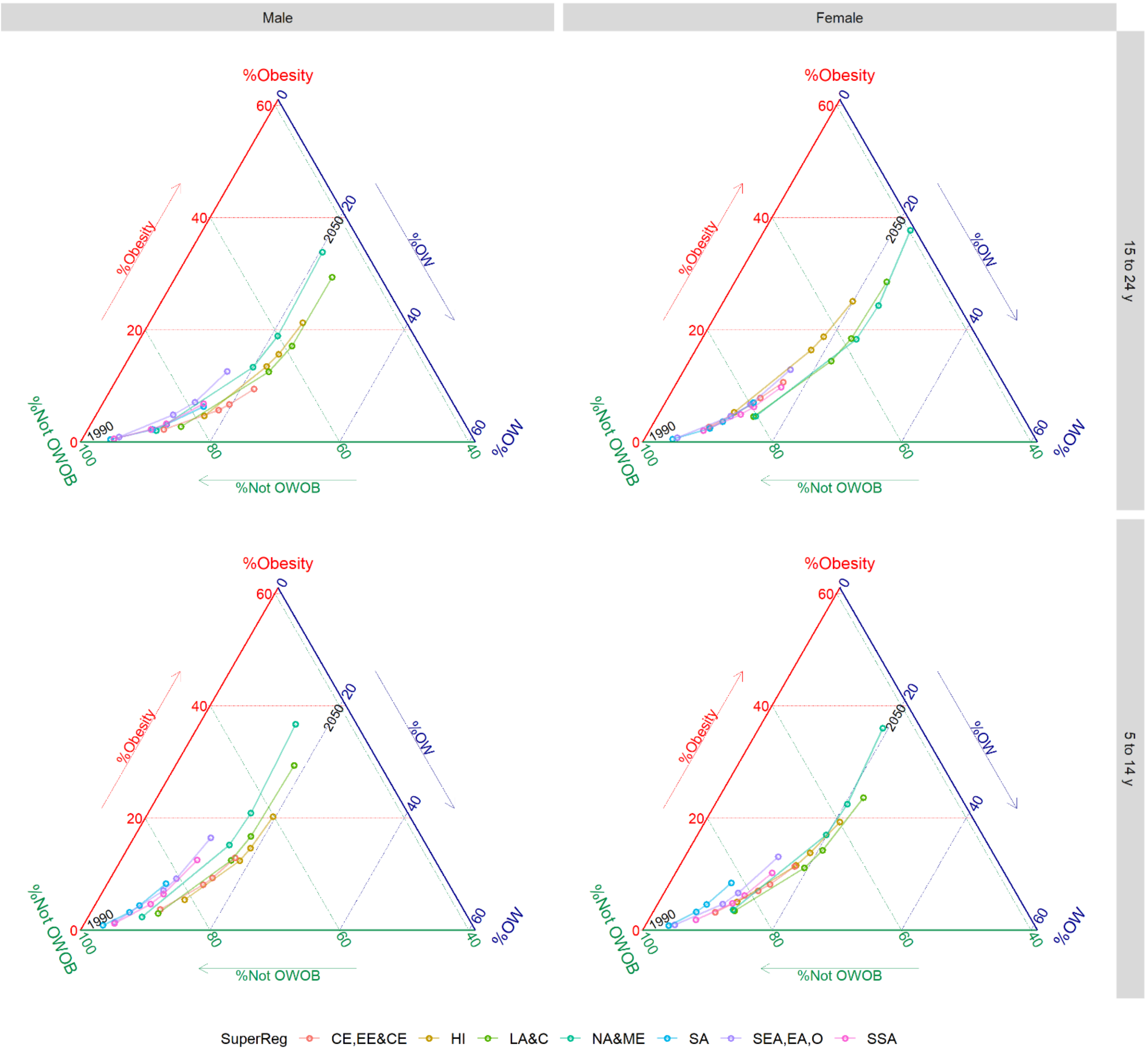

Figure S8: Country-level heatmaps of estimated age-standardised prevalence of obesity, 1990, 2021, 2030, 2050 by sex, super-region, and age group 5-14 vs. 15-24 years

		5 to 14 years							
		Female				Male			
		1990	2021	2030	2050	1990	2021	2030	2050
High-income	Andorra	5.1	10.6	12.5	17.1	6.3	11.0	12.8	17.1
	Argentina	2.6	7.8	10.1	17.0	2.8	9.4	12.3	20.6
	Australia	6.7	17.3	20.8	29.8	4.5	14.5	18.2	27.5
	Austria	3.5	7.2	8.4	11.6	4.6	8.3	9.4	12.2
	Belgium	3.3	10.1	12.2	18.2	2.7	8.2	10.1	15.0
	Brunei Darussalam	0.2	1.8	2.8	7.1	0.6	3.9	5.9	13.2
	Canada	8.8	15.2	17.1	21.5	9.1	17.2	19.2	24.5
	Chile	5.8	16.8	20.4	30.0	5.7	20.9	25.3	37.0
	Cyprus	2.2	9.4	12.3	20.7	3.2	12.7	16.1	25.1
	Denmark	5.8	12.1	14.3	19.4	5.9	11.6	13.5	18.0
	Finland	5.0	11.5	13.5	18.6	4.5	11.4	13.6	19.2
	France	2.3	7.5	9.7	15.4	3.1	10.6	13.5	21.1
	Germany	6.6	11.7	13.2	16.7	8.5	13.4	14.5	17.3
	Greece	2.4	8.8	12.0	20.1	3.6	12.9	16.7	25.9
	Greenland	11.9	17.5	18.8	22.4	11.9	18.0	20.1	24.6
	Iceland	6.1	13.4	15.2	19.8	7.5	12.7	13.9	16.8
	Ireland	4.3	11.5	13.3	18.3	4.1	13.9	16.2	22.7
	Israel	4.1	8.5	10.4	15.1	4.3	7.9	9.2	12.5
	Italy	2.0	6.6	8.7	14.3	2.1	7.3	9.6	15.7
	Japan	2.0	5.0	6.2	9.4	2.9	7.1	8.9	13.4
	Luxembourg	3.4	8.9	10.6	15.2	4.8	11.4	13.5	18.5
	Malta	2.1	8.1	10.5	16.9	1.7	6.8	9.1	15.2
	Monaco	6.8	14.0	16.3	22.1	8.9	15.5	17.5	22.5
	Netherlands	4.2	10.8	13.3	19.2	2.8	6.8	8.1	11.4
	New Zealand	9.8	16.6	19.3	25.6	10.4	16.8	19.3	25.1
	Norway	4.4	9.5	11.1	15.3	5.6	10.7	12.0	15.5
	Portugal	2.2	9.2	12.3	20.4	2.0	9.3	12.6	20.9
	Republic of Korea	0.6	4.5	6.2	11.5	1.3	7.8	10.4	17.2
	San Marino	5.6	12.4	15.1	22.1	6.9	14.1	16.8	23.9
	Singapore	0.7	6.3	9.2	19.6	0.8	7.5	10.9	22.7
	Spain	2.5	9.4	12.5	20.8	3.0	11.8	15.0	23.4
	Sweden	4.9	9.5	11.2	15.5	5.2	10.3	12.1	16.9
	Switzerland	4.6	7.6	8.7	11.5	5.8	8.2	9.2	11.5
	United Kingdom	5.3	12.0	13.7	18.4	4.3	9.9	11.4	15.5
	United States of America	8.8	15.7	17.7	22.7	9.1	15.9	17.9	22.6
	Uruguay	6.8	13.7	15.9	20.9	3.5	9.8	11.9	17.6

Prevalence %

Best observed — Worst observed

		15 to 24 years							
		Female				Male			
		1990	2021	2030	2050	1990	2021	2030	2050
High-income	Andorra	4.8	9.9	11.8	16.3	4.9	8.7	10.2	13.9
	Argentina	4.0	13.6	16.8	25.6	3.0	11.4	14.5	23.3
	Australia	6.6	18.4	22.3	31.4	4.4	13.2	16.6	25.3
	Austria	3.3	6.6	7.7	10.6	3.5	6.4	7.3	9.7
	Belgium	4.5	12.0	14.6	21.2	2.6	8.2	10.0	15.1
	Brunei Darussalam	0.9	7.0	9.9	19.3	1.4	8.3	11.6	22.2
	Canada	7.6	16.1	18.0	22.6	5.8	13.6	15.4	20.0
	Chile	6.0	19.3	23.0	32.8	4.2	16.5	20.4	31.0
	Cyprus	2.4	9.9	13.0	21.5	2.4	10.1	13.0	21.1
	Denmark	4.5	12.2	14.5	19.7	3.5	8.9	10.5	14.4
	Finland	4.5	11.4	13.4	18.5	3.0	8.2	9.9	14.4
	France	2.5	7.7	9.8	15.7	2.6	8.9	11.6	18.7
	Germany	6.0	11.4	12.8	16.2	5.9	9.5	10.5	12.8
	Greece	3.2	9.6	12.8	21.1	3.5	11.3	14.9	23.5
	Greenland	13.6	19.6	21.3	25.2	9.4	15.0	16.6	20.6
	Iceland	5.9	14.2	16.3	21.0	4.6	9.6	10.5	13.0
	Ireland	5.0	12.8	14.7	19.9	4.0	12.1	14.4	20.3
	Israel	4.3	8.7	10.5	15.2	3.4	6.3	7.4	10.2
	Italy	2.6	5.9	7.7	12.9	1.9	4.9	6.5	11.0
	Japan	1.0	2.4	3.1	4.9	1.6	4.3	5.6	8.9
	Luxembourg	3.4	8.9	10.6	15.1	3.5	8.7	10.3	14.5
	Malta	2.9	10.0	12.8	20.0	1.6	6.6	8.7	14.7
	Monaco	6.9	14.1	16.4	22.1	7.1	12.9	14.8	19.3
	Netherlands	4.5	13.3	16.1	22.5	2.1	5.3	6.5	9.2
	New Zealand	11.0	22.7	25.8	32.9	8.9	18.9	21.5	27.5
	Norway	4.6	9.0	10.6	14.6	4.5	7.3	8.3	11.0
	Portugal	2.8	9.7	13.1	21.5	1.8	8.4	11.4	19.3
	Republic of Korea	0.3	2.0	2.9	5.9	0.6	3.7	5.2	9.5
	San Marino	5.6	12.5	15.2	22.1	5.5	11.7	14.1	20.5
	Singapore	0.9	7.2	10.5	21.5	0.8	7.4	11.0	23.0
	Spain	3.4	10.8	14.2	23.1	3.9	9.6	12.3	19.7
	Sweden	4.3	9.7	11.5	15.9	3.0	7.1	8.5	12.2
	Switzerland	3.7	7.8	8.9	11.7	3.1	5.5	6.2	8.0
	United Kingdom	6.8	15.4	17.5	22.9	5.3	12.1	13.8	18.3
	United States of America	10.1	28.8	31.7	38.0	8.8	22.7	25.1	30.6
	Uruguay	5.7	12.8	14.8	19.6	2.7	8.5	10.6	15.9

Prevalence %

Best observed — Worst observed

		5 to 14 years							
		Female				Male			
		1990	2021	2030	2050	1990	2021	2030	2050
North Africa and Middle East	Afghanistan	2.8	4.7	5.4	8.1	0.8	4.0	5.7	14.2
	Algeria	2.8	14.4	20.6	37.5	1.4	10.2	15.6	31.8
	Bahrain	8.2	34.8	44.0	61.8	4.0	22.5	30.7	49.1
	Egypt	5.7	27.2	36.7	58.0	3.2	20.5	29.9	52.6
	Iran (Islamic Republic of)	3.2	20.6	28.8	48.5	1.8	16.4	24.2	44.5
	Iraq	3.1	14.8	19.8	33.0	2.5	15.0	20.4	34.0
	Jordan	2.8	16.7	22.3	37.1	2.9	14.1	18.5	30.1
	Kuwait	7.4	33.7	41.4	57.1	12.2	42.8	49.9	63.5
	Lebanon	6.1	22.7	27.8	41.3	4.4	21.3	26.6	40.6
	Libya	4.1	26.6	36.0	57.1	3.4	24.1	33.4	54.6
	Morocco	3.2	10.7	14.4	23.9	2.0	9.4	13.7	26.2
	Oman	3.7	24.8	33.7	54.4	2.0	19.4	27.6	49.2
	Palestine	2.4	11.9	15.3	24.9	2.0	11.4	14.7	24.5
	Qatar	5.2	28.8	37.0	55.8	8.8	34.9	43.2	60.2
	Saudi Arabia	6.7	33.9	41.9	58.4	6.8	35.9	44.4	60.9
	Sudan	2.5	9.7	12.8	21.7	1.7	9.8	13.5	24.8
	Syrian Arab Republic	4.7	28.3	37.1	57.1	2.9	26.4	36.0	57.9
	Tunisia	2.7	16.0	23.3	42.8	1.7	13.8	20.5	39.3
	Türkiye	2.1	9.5	12.7	20.5	1.6	11.6	15.8	26.4
	United Arab Emirates	7.0	38.1	48.2	67.5	11.7	46.7	56.0	72.9
	Yemen	2.9	6.9	8.6	14.5	0.8	8.4	13.1	30.5

Prevalence %

Best observed — Worst observed

		15 to 24 years							
		Female				Male			
		1990	2021	2030	2050	1990	2021	2030	2050
North Africa and Middle East	Afghanistan	3.8	7.7	8.7	12.5	1.0	5.2	7.4	17.3
	Algeria	3.4	16.1	22.7	39.9	1.1	8.4	13.3	28.7
	Bahrain	11.0	38.0	47.4	64.6	4.3	23.6	31.9	50.3
	Egypt	6.8	26.3	35.8	57.2	2.0	13.3	20.7	41.7
	Iran (Islamic Republic of)	4.4	22.0	30.4	50.0	1.5	14.2	21.6	41.5
	Iraq	4.0	16.9	22.0	35.6	2.9	16.4	22.0	36.2
	Jordan	5.8	22.3	28.6	44.1	3.4	13.9	18.1	30.1
	Kuwait	8.3	33.5	41.2	56.9	12.8	37.5	44.6	59.2
	Lebanon	6.2	22.8	27.9	41.3	4.1	19.9	24.9	38.3
	Libya	5.1	29.6	39.6	60.2	2.8	21.7	30.7	52.1
	Morocco	3.0	9.3	12.6	21.5	0.8	5.1	7.8	17.2
	Oman	6.5	28.6	37.3	57.3	2.1	17.1	24.7	46.0
	Palestine	6.1	23.4	27.9	39.2	4.3	19.6	24.3	35.9
	Qatar	6.8	31.9	40.2	58.4	8.6	35.7	44.0	60.9
	Saudi Arabia	9.8	30.5	38.5	55.4	6.3	32.4	41.0	58.5
	Sudan	4.3	14.9	18.8	29.2	2.3	13.4	18.1	31.4
	Syrian Arab Republic	4.4	26.5	35.1	55.0	2.1	21.6	30.6	52.7
	Tunisia	2.4	16.0	23.0	42.1	1.1	9.8	15.2	31.9
	Türkiye	3.7	13.2	17.1	26.0	1.5	9.2	12.8	22.2
	United Arab Emirates	7.8	36.8	46.7	66.2	9.6	38.8	48.6	67.4
	Yemen	1.9	5.0	6.4	11.4	0.4	5.4	9.0	23.3

Prevalence %

Best observed — Worst observed

Latin America and Caribbean

	5 to 14 years							
	Female				Male			
	1990	2021	2030	2050	1990	2021	2030	2050
Antigua and Barbuda	5.6	16.1	20.5	32.4	3.5	12.1	15.9	27.5
Bahamas	8.2	21.4	26.4	38.8	5.7	17.7	22.8	36.2
Barbados	6.4	18.2	22.7	34.1	4.8	16.2	20.8	32.4
Belize	6.7	16.4	19.5	27.5	3.2	10.7	13.5	21.9
Bermuda	7.0	20.3	24.9	37.1	6.9	22.1	27.8	42.1
Bolivia (Plurinational State of)	3.2	10.6	13.6	22.2	2.6	9.7	12.8	22.3
Brazil	3.2	11.4	15.1	26.2	2.8	13.7	19.3	35.2
Colombia	1.4	5.5	7.5	13.0	1.2	6.3	9.0	16.8
Costa Rica	6.5	16.5	19.8	27.5	6.2	18.7	22.8	32.2
Cuba	5.2	16.3	21.0	32.8	2.9	11.4	15.1	26.0
Dominica	12.0	27.3	32.5	44.8	6.2	19.8	25.3	39.8
Dominican Republic	3.4	11.6	15.0	24.4	1.5	6.4	8.8	16.1
Ecuador	2.6	9.2	12.0	20.5	1.9	9.9	14.0	26.0
El Salvador	3.4	10.6	13.7	22.4	2.0	8.4	11.5	21.0
Grenada	3.5	11.1	14.5	24.6	1.6	7.3	10.4	20.7
Guatemala	1.6	4.7	6.2	10.5	2.1	7.5	10.3	18.8
Guyana	5.3	13.1	16.8	25.3	2.4	7.1	9.6	15.7
Haiti	1.0	2.9	3.9	7.5	0.4	2.2	3.2	7.7
Honduras	2.1	5.7	7.2	11.6	1.2	4.2	5.6	10.1
Jamaica	8.7	21.4	25.8	36.7	2.5	8.8	11.6	19.8
Mexico	4.5	14.3	18.6	30.2	4.0	16.9	22.8	38.3
Nicaragua	2.3	6.8	9.0	14.9	2.2	7.8	10.3	18.0
Panama	3.6	12.0	15.9	27.2	3.2	11.5	15.6	27.8
Paraguay	2.5	9.4	12.0	18.9	2.7	11.8	15.5	24.5
Peru	3.6	10.6	12.7	18.2	3.3	10.4	12.5	18.2
Puerto Rico	10.5	24.6	28.0	35.8	10.3	25.8	29.5	37.8
Saint Kitts and Nevis	5.9	17.9	22.7	35.6	3.8	14.4	19.4	33.2
Saint Lucia	3.3	10.1	13.5	22.9	1.8	6.7	9.3	17.4
Saint Vincent and the Grenadines	2.8	9.1	12.2	20.9	1.1	4.8	7.0	13.7
Suriname	1.3	4.3	5.7	10.2	0.6	2.2	3.0	6.1
Trinidad and Tobago	3.8	11.0	13.9	22.3	3.8	12.1	15.4	25.1
United States Virgin Islands	18.6	32.3	36.0	45.1	11.4	23.8	27.0	35.8
Venezuela (Bolivarian Republic of)	4.5	13.1	17.1	28.9	4.5	15.0	20.5	35.8

Prevalence %

Best observed — Worst observed

Latin America and Caribbean

	15 to 24 years							
	Female				Male			
	1990	2021	2030	2050	1990	2021	2030	2050
Antigua and Barbuda	6.2	17.8	22.4	34.4	2.8	10.2	13.9	24.9
Bahamas	9.0	23.0	27.9	40.5	4.5	15.3	20.0	33.1
Barbados	9.1	23.5	28.5	40.6	4.5	16.2	21.3	34.3
Belize	11.4	24.8	28.6	37.8	4.0	12.8	16.1	25.1
Bermuda	7.0	19.8	24.4	36.6	5.4	18.5	23.6	37.4
Bolivia (Plurinational State of)	2.8	9.5	12.4	20.6	1.8	7.4	9.8	17.7
Brazil	3.4	12.9	17.1	29.0	2.1	12.4	17.6	33.2
Colombia	2.6	10.3	13.3	21.1	1.5	8.1	11.3	20.2
Costa Rica	6.7	16.8	20.1	27.7	4.0	13.0	16.4	24.1
Cuba	7.2	20.5	25.5	37.9	3.3	12.9	16.9	28.3
Dominica	11.2	26.3	31.4	43.6	3.5	13.4	18.0	30.9
Dominican Republic	4.3	14.0	17.9	28.1	2.0	7.9	10.8	19.2
Ecuador	4.4	13.1	16.8	27.0	2.1	10.0	14.1	26.3
El Salvador	6.4	17.4	21.8	32.5	3.7	13.6	18.0	29.6
Grenada	4.5	13.7	17.5	28.3	1.3	6.5	9.4	19.0
Guatemala	3.5	9.2	11.5	17.8	1.8	7.5	10.1	18.0
Guyana	5.2	13.4	17.3	26.1	2.3	7.1	9.7	16.1
Haiti	1.5	4.2	5.5	10.2	0.2	1.2	1.8	4.6
Honduras	2.8	7.4	9.4	14.7	1.5	4.9	6.5	11.4
Jamaica	9.1	22.9	27.5	38.5	2.1	7.9	10.6	18.7
Mexico	6.8	21.6	26.9	40.1	4.4	19.8	26.4	42.8
Nicaragua	6.0	14.5	18.0	26.5	3.4	11.0	14.3	23.2
Panama	3.1	10.5	14.0	24.2	1.6	6.8	9.6	18.9
Paraguay	3.0	10.6	13.5	20.8	1.8	8.8	11.7	19.4
Peru	3.0	10.1	12.1	17.5	2.4	8.1	9.9	14.8
Puerto Rico	10.0	24.4	27.8	35.5	8.2	20.3	24.0	31.8
Saint Kitts and Nevis	6.5	19.5	24.5	37.6	3.0	12.4	16.9	30.1
Saint Lucia	5.2	14.9	18.9	30.2	1.3	5.6	8.1	16.3
Saint Vincent and the Grenadines	3.1	10.4	13.8	23.0	0.8	4.0	5.8	11.7
Suriname	3.4	10.5	13.2	21.2	1.4	5.5	7.3	13.4
Trinidad and Tobago	7.1	17.0	20.9	31.2	5.2	15.9	20.3	32.0
United States Virgin Islands	18.4	32.9	36.6	45.6	8.4	18.6	21.6	29.9
Venezuela (Bolivarian Republic of)	6.2	17.0	21.6	34.4	3.8	13.2	18.2	32.7

Prevalence %

Best observed — Worst observed

		5 to 14 years							
		Female				Male			
		1990	2021	2030	2050	1990	2021	2030	2050
South Asia	Bangladesh	0.8	2.9	4.3	8.6	0.7	2.1	2.8	5.1
	Bhutan	2.1	5.1	6.7	11.4	0.9	4.1	5.9	11.9
	India	0.8	3.4	4.9	9.0	0.9	3.5	4.9	9.0
	Nepal	1.3	5.0	7.0	12.9	0.3	1.7	2.8	6.7
	Pakistan	1.0	2.5	3.4	6.0	0.7	2.4	3.4	6.8
Southeast Asia, East Asia, and Oceania	American Samoa	22.6	42.7	47.7	59.1	21.1	40.3	45.4	56.6
	Cambodia	0.9	2.0	2.5	4.2	0.6	1.7	2.3	4.2
	China	0.8	4.9	7.4	16.7	1.6	9.0	12.5	24.6
	Cook Islands	32.0	52.2	57.6	68.1	33.0	55.0	60.7	71.6
	DPR of Korea	1.7	9.3	13.5	26.7	2.2	9.7	13.5	24.7
	Fiji	5.4	18.3	23.4	36.5	2.7	11.4	15.6	27.6
	Guam	16.7	28.7	31.6	38.8	17.4	27.3	29.5	35.5
	Indonesia	0.9	3.6	4.7	8.1	0.8	3.4	4.5	7.3
	Kiribati	5.0	17.9	23.9	39.8	5.5	20.2	26.9	44.1
	Lao People's Democratic Republic	0.7	1.9	2.5	4.1	0.3	1.4	2.1	4.2
	Malaysia	4.0	14.0	18.1	29.1	1.7	7.1	9.2	15.7
	Maldives	0.8	4.4	6.4	12.4	0.8	6.5	9.7	19.3
	Marshall Islands	13.4	27.6	32.5	42.1	9.5	25.0	30.4	41.5
	Mauritius	2.5	9.8	12.9	22.0	2.3	8.9	11.6	19.1
	Micronesia (Federated States of)	12.4	31.0	37.2	51.5	12.0	30.1	36.5	50.9
	Myanmar	1.1	2.6	3.2	5.1	0.9	2.6	3.2	5.5
	Nauru	25.4	46.3	52.1	64.2	24.0	49.6	56.6	70.4
	Niue	14.9	37.2	44.2	58.2	15.2	36.2	42.8	56.2
	Northern Mariana Islands	18.8	42.5	49.6	64.2	23.9	47.2	53.8	67.3
	Palau	19.3	40.9	46.8	59.3	22.5	40.3	45.4	56.1
	Papua New Guinea	2.3	5.0	6.0	9.7	0.7	2.5	3.5	7.3
	Philippines	1.1	3.8	5.2	9.9	1.6	4.6	6.1	10.7
	Samoa	21.4	42.0	47.7	59.8	8.0	22.5	27.5	39.9
	Seychelles	4.1	16.3	22.1	37.5	2.8	13.6	19.0	33.6
	Solomon Islands	3.0	8.4	10.7	18.9	1.6	6.1	8.4	17.1
	Sri Lanka	1.8	6.5	8.1	13.4	0.9	3.3	4.3	8.0
	Taiwan (Province of China)	1.5	13.3	19.8	38.0	3.1	22.0	30.5	49.8
	Thailand	2.2	9.5	13.1	22.8	1.8	8.0	10.9	18.6
	Timor-Leste	0.9	3.0	4.3	8.6	0.9	5.3	8.0	17.2
	Tokelau	10.9	30.3	36.5	49.8	16.2	40.5	47.2	60.1
	Tonga	20.6	44.2	51.4	65.8	22.6	48.6	56.2	70.1
	Tuvalu	9.1	29.8	38.3	56.0	11.8	33.0	41.1	57.4
	Vanuatu	3.5	10.1	13.1	22.5	2.2	7.4	10.0	18.8
	Viet Nam	0.3	1.2	1.7	3.3	0.4	1.3	1.8	3.2

Prevalence %

Best observed — Worst observed

		15 to 24 years							
		Female				Male			
		1990	2021	2030	2050	1990	2021	2030	2050
South Asia	Bangladesh	0.5	3.0	4.5	9.2	0.4	1.6	2.3	4.4
	Bhutan	1.7	4.5	6.0	10.3	0.6	3.0	4.5	9.6
	India	0.4	2.0	3.1	6.0	0.4	2.2	3.2	6.1
	Nepal	0.6	1.7	2.6	5.5	0.3	1.8	2.8	6.8
	Pakistan	1.4	4.4	5.9	10.1	0.7	3.0	4.2	8.2
Southeast Asia, East Asia, and Oceania	American Samoa	25.9	45.7	50.8	61.6	19.4	36.9	41.7	52.8
	Cambodia	0.4	0.9	1.2	2.1	0.2	0.7	0.9	1.9
	China	0.7	4.6	7.2	16.2	0.9	6.4	9.3	19.4
	Cook Islands	35.1	55.9	60.9	70.7	29.3	50.9	56.5	67.9
	DPR of Korea	0.7	4.8	7.4	17.2	0.8	4.6	6.7	14.2
	Fiji	6.2	19.5	24.7	37.9	1.9	8.8	12.2	22.4
	Guam	16.4	28.9	31.9	39.0	14.0	22.3	24.4	30.0
	Indonesia	0.7	4.0	5.4	9.4	0.4	2.1	2.8	4.8
	Kiribati	9.6	27.1	33.4	48.6	5.5	20.2	26.5	42.7
	Lao People's Democratic Republic	0.8	2.3	3.0	5.1	0.2	0.9	1.2	2.7
	Malaysia	4.6	17.2	21.9	34.1	1.2	4.6	6.1	10.8
	Maldives	1.1	6.7	9.7	18.1	0.4	3.7	6.0	13.5
	Marshall Islands	13.1	26.6	31.3	40.8	7.4	19.9	24.7	35.1
	Mauritius	3.2	11.3	14.8	24.5	1.8	7.3	9.8	16.6
	Micronesia (Federated States of)	15.1	34.7	40.9	54.8	10.5	27.2	33.0	46.9
	Myanmar	0.6	1.6	2.0	3.3	0.5	1.6	2.1	3.6
	Nauru	30.1	49.8	55.6	67.0	25.3	49.0	55.9	69.3
	Niue	15.4	37.6	44.8	58.5	12.2	30.7	36.8	49.9
	Northern Mariana Islands	19.3	42.8	49.8	64.2	19.5	41.1	47.8	61.9
	Palau	19.0	40.0	46.1	58.5	18.1	34.1	38.9	49.5
	Papua New Guinea	1.8	3.8	4.7	7.7	0.8	2.8	3.9	8.1
	Philippines	1.1	3.9	5.4	10.4	1.0	3.5	4.6	8.5
	Samoa	23.9	45.9	51.3	62.8	8.0	21.4	25.9	37.1
	Seychelles	5.1	18.7	25.2	41.1	1.9	10.3	14.8	28.2
	Solomon Islands	3.6	9.1	11.6	20.0	1.1	4.3	6.0	12.8
	Sri Lanka	1.7	5.2	6.6	11.2	0.5	2.0	2.8	5.3
	Taiwan (Province of China)	1.3	12.2	18.3	36.0	1.8	14.9	22.0	40.3
	Thailand	2.4	10.0	13.7	23.8	1.3	5.9	8.1	14.7
	Timor-Leste	0.2	0.9	1.4	3.2	0.1	1.0	1.8	5.1
	Tokelau	11.5	31.2	37.0	50.0	13.1	34.8	41.1	54.1
	Tonga	23.5	49.3	56.1	69.4	17.2	40.8	48.2	63.0
	Tuvalu	12.2	35.4	43.8	60.5	11.0	31.1	38.7	54.5
	Vanuatu	3.4	9.7	12.6	21.7	1.5	5.4	7.6	15.1
	Viet Nam	0.2	0.9	1.2	2.5	0.3	1.1	1.5	2.8

Prevalence %

Best observed — Worst observed

Region	Country	5 to 14 years							
		Female				Male			
		1990	2021	2030	2050	1990	2021	2030	2050
Central Europe, Eastern Europe, and Central Asia	Albania	2.2	6.3	7.5	10.0	3.9	7.6	8.3	9.7
	Armenia	3.0	5.8	6.6	8.6	4.5	8.1	9.1	11.5
	Azerbaijan	3.2	7.4	8.7	12.3	4.5	8.2	9.4	12.3
	Belarus	1.9	6.3	7.8	12.4	1.6	6.0	7.6	12.4
	Bosnia and Herzegovina	3.9	11.6	14.1	20.1	5.0	11.7	13.4	17.4
	Bulgaria	4.1	7.7	8.8	11.4	6.3	10.7	11.9	14.4
	Croatia	3.0	9.5	12.1	18.0	6.7	14.8	17.5	22.9
	Czechia	3.4	6.4	7.4	10.0	5.4	8.2	9.2	11.5
	Estonia	6.4	11.6	13.3	17.5	6.2	12.1	14.1	19.3
	Georgia	2.9	5.2	5.8	7.1	5.0	8.7	9.4	11.2
	Hungary	5.7	11.9	14.2	19.6	6.1	12.0	13.8	18.5
	Kazakhstan	2.1	5.3	6.3	9.0	2.7	5.9	6.7	8.9
	Kyrgyzstan	1.7	4.4	5.4	8.2	1.9	4.4	5.3	7.8
	Latvia	3.4	6.7	7.8	10.3	3.3	7.3	8.7	12.0
	Lithuania	1.1	3.5	4.4	6.2	2.4	7.5	9.0	12.2
	Mongolia	2.5	5.4	6.2	8.4	3.0	4.3	4.6	5.2
	Montenegro	4.2	13.4	17.1	26.4	11.5	22.8	25.8	33.0
	North Macedonia	2.5	7.2	9.0	13.9	5.7	10.7	12.1	16.0
	Poland	2.3	5.9	7.1	10.1	3.5	8.4	9.9	13.4
	Republic of Moldova	1.8	5.6	7.0	11.2	1.7	5.6	7.1	11.6
	Romania	2.5	7.3	8.9	12.5	4.6	11.1	12.8	16.6
	Russian Federation	4.4	8.5	9.7	13.3	4.0	9.6	11.6	17.0
	Serbia	2.5	9.4	12.1	19.3	2.4	7.4	9.3	14.4
	Slovakia	2.2	4.5	5.4	7.8	4.5	7.9	9.0	11.9
	Slovenia	2.1	5.8	7.3	11.4	6.3	12.1	14.2	19.1
	Tajikistan	2.8	6.0	7.3	10.3	3.1	6.3	7.5	10.5
	Turkmenistan	2.0	3.7	4.2	5.5	3.6	5.2	5.5	6.3
	Ukraine	1.9	5.0	5.9	8.8	2.1	5.8	6.7	9.8
	Uzbekistan	2.7	6.5	7.7	10.8	2.7	6.4	7.6	10.4

Prevalence %

Best observed — Worst observed

Region	Country	15 to 24 years							
		Female				Male			
		1990	2021	2030	2050	1990	2021	2030	2050
Central Europe, Eastern Europe, and Central Asia	Albania	1.3	4.0	4.8	6.6	1.4	2.7	3.0	3.5
	Armenia	1.9	3.8	4.4	5.8	1.5	3.3	3.9	5.1
	Azerbaijan	2.2	5.5	6.7	9.7	1.2	2.7	3.1	4.3
	Belarus	1.8	5.6	7.1	11.4	1.3	5.1	6.5	10.8
	Bosnia and Herzegovina	1.4	4.9	6.2	9.8	2.3	6.0	7.0	9.5
	Bulgaria	3.8	7.2	8.3	10.8	4.3	7.7	8.6	10.6
	Croatia	3.3	9.9	12.6	18.5	5.1	11.6	13.8	18.5
	Czechia	3.7	6.3	7.3	9.8	3.0	4.6	5.2	6.7
	Estonia	6.5	12.2	14.0	18.2	4.8	10.8	12.7	17.6
	Georgia	4.3	9.0	10.0	12.0	5.5	11.2	12.2	14.4
	Hungary	6.1	12.7	15.0	20.5	5.4	10.9	12.8	17.2
	Kazakhstan	1.8	4.5	5.3	7.7	1.9	4.2	4.9	6.6
	Kyrgyzstan	1.0	2.6	3.3	5.1	1.8	4.4	5.3	7.8
	Latvia	3.7	8.0	9.1	11.9	2.4	6.5	7.6	10.5
	Lithuania	1.7	5.8	7.0	9.5	2.0	6.0	7.2	10.0
	Mongolia	1.9	5.0	5.8	7.8	2.8	4.5	4.8	5.4
	Montenegro	2.3	8.3	10.8	17.9	7.1	14.6	17.0	23.0
	North Macedonia	1.2	4.2	5.3	8.8	4.2	8.3	9.6	12.9
	Poland	2.3	6.0	7.3	10.4	2.3	5.8	6.9	9.6
	Republic of Moldova	2.7	8.1	10.0	15.3	1.8	5.8	7.4	11.8
	Romania	3.1	8.7	10.2	14.1	4.6	11.5	13.2	17.1
	Russian Federation	3.1	7.7	9.0	12.4	1.5	5.7	7.1	11.0
	Serbia	2.6	10.2	13.1	20.6	1.3	4.2	5.3	8.6
	Slovakia	2.2	4.5	5.3	7.6	3.8	7.1	8.1	10.8
	Slovenia	2.3	6.1	7.7	12.0	5.1	9.8	11.6	15.8
	Tajikistan	2.0	4.2	5.1	7.4	2.4	5.5	6.5	9.1
	Turkmenistan	1.9	3.7	4.2	5.6	2.6	3.8	4.0	4.6
	Ukraine	2.4	6.7	7.7	11.2	1.9	5.1	5.8	8.6
	Uzbekistan	1.6	5.1	6.2	8.8	1.3	4.1	4.9	6.9

Prevalence %

Best observed — Worst observed

Region	Country	5 to 14 years							
		Female				Male			
		1990	2021	2030	2050	1990	2021	2030	2050
Sub-Saharan Africa	Angola	3.1	9.9	13.4	23.9	1.7	9.7	13.8	27.2
	Benin	2.1	3.7	4.5	7.3	1.0	3.8	6.0	13.8
	Botswana	2.9	10.1	13.5	23.3	1.1	7.3	11.1	23.6
	Burkina Faso	3.1	4.7	5.4	7.2	2.4	5.7	7.3	12.5
	Burundi	1.0	2.1	2.7	5.0	0.5	1.7	2.7	6.9
	Cabo Verde	1.7	5.7	7.8	13.2	1.0	4.7	6.8	13.1
	Cameroon	2.8	9.4	12.7	21.8	1.9	9.0	13.0	25.9
	Central African Republic	3.4	9.0	12.8	24.6	1.9	10.3	17.6	39.8
	Chad	1.7	2.8	3.2	4.6	1.1	3.0	3.9	7.8
	Comoros	4.6	14.1	18.2	28.8	3.2	19.0	26.5	45.7
	Congo	2.2	11.2	16.1	31.2	0.8	5.3	8.1	18.1
	Côte d'Ivoire	0.5	1.2	1.5	2.6	0.4	2.1	3.3	8.1
	Democratic Republic of the Congo	1.0	5.2	7.9	17.1	1.3	6.4	9.5	19.4
	Djibouti	3.0	6.0	7.3	10.5	1.9	7.7	11.2	21.2
	Equatorial Guinea	8.2	28.4	34.0	46.6	5.8	29.2	35.7	50.3
	Eritrea	1.0	2.4	3.1	5.4	0.5	3.6	6.2	16.9
	Eswatini	4.4	13.2	16.7	26.0	1.5	7.5	10.4	20.0
	Ethiopia	1.6	2.2	2.4	3.1	0.5	1.9	2.8	6.1
	Gabon	2.2	9.0	12.4	21.1	0.8	4.9	7.1	13.9
	Gambia	0.8	1.6	2.0	3.4	0.4	1.2	1.7	3.1
	Ghana	0.7	3.3	4.7	9.3	0.5	2.3	3.4	6.9
	Guinea	1.9	3.2	3.8	5.8	0.8	2.8	4.1	8.8
	Guinea-Bissau	2.1	3.9	4.8	7.5	1.2	4.5	6.7	14.2
	Kenya	1.4	4.6	6.4	11.7	1.1	4.6	6.4	12.3
	Lesotho	1.6	4.0	5.0	8.2	0.8	3.7	5.1	10.4
	Liberia	1.9	4.8	6.2	10.3	1.8	5.9	7.9	13.6
	Madagascar	0.9	2.5	3.3	5.9	0.9	2.9	4.1	8.0
	Malawi	3.2	8.3	10.3	16.1	2.6	9.1	11.8	19.6
	Mali	1.3	1.8	2.1	2.9	1.0	2.6	3.4	6.4
	Mauritania	3.7	9.1	12.0	19.2	2.4	7.7	10.6	18.4
	Mozambique	1.9	4.5	5.9	10.4	1.1	3.7	5.3	10.6
	Namibia	2.8	8.9	10.9	17.0	1.8	8.2	10.9	18.9
	Niger	1.3	1.9	2.1	2.8	0.9	1.8	2.3	4.1
	Nigeria	1.3	4.0	5.3	9.4	0.9	4.0	5.8	12.0
	Rwanda	1.7	4.2	5.5	9.4	1.1	3.4	4.7	8.7
	Sao Tome and Principe	1.9	5.8	7.7	13.4	1.1	4.4	6.1	11.1
	Senegal	1.8	3.5	4.2	6.4	0.7	2.4	3.5	7.3
	Sierra Leone	3.6	5.8	7.0	10.3	1.9	5.0	6.7	12.0
	Somalia	1.7	3.4	4.1	6.9	1.1	3.9	5.4	11.5
	South Africa	4.6	14.3	18.2	29.3	2.5	9.9	12.8	21.1
	South Sudan	3.5	4.5	4.9	6.2	1.7	5.6	8.7	21.3
	Togo	1.5	3.9	5.2	9.3	0.8	3.4	5.3	11.8
	Uganda	1.3	4.1	5.5	10.4	1.0	3.8	5.1	9.8
	United Republic of Tanzania	1.7	5.8	8.3	15.9	1.2	5.0	7.1	14.0
	Zambia	3.6	10.2	12.7	19.8	4.0	13.0	16.7	26.6
	Zimbabwe	0.6	2.4	3.4	6.7	0.5	2.2	3.1	6.5

Prevalence %

Best observed — Worst observed

Region	Country	15 to 24 years							
		Female				Male			
		1990	2021	2030	2050	1990	2021	2030	2050
Sub-Saharan Africa	Angola	0.9	3.5	5.1	10.7	0.3	2.2	3.6	9.3
	Benin	2.2	3.7	4.6	7.4	0.5	2.2	3.5	8.7
	Botswana	3.6	12.0	15.8	26.1	0.3	2.7	4.4	11.4
	Burkina Faso	1.5	2.3	2.7	3.8	0.7	2.1	2.8	5.4
	Burundi	0.5	1.0	1.4	2.7	0.2	0.5	0.9	2.5
	Cabo Verde	2.3	7.3	9.8	16.1	0.6	3.3	4.9	9.8
	Cameroon	3.5	10.1	13.5	23.0	1.0	5.6	8.5	18.4
	Central African Republic	1.5	4.3	6.6	14.5	0.4	3.1	6.2	20.3
	Chad	0.9	1.4	1.6	2.5	0.3	0.9	1.2	2.7
	Comoros	2.9	9.8	12.9	21.9	0.7	6.3	10.1	23.0
	Congo	2.6	13.1	18.6	34.6	0.4	2.9	4.6	11.7
	Côte d'Ivoire	2.4	5.0	6.1	9.2	0.7	3.3	5.2	11.9
	Democratic Republic of the Congo	0.2	1.5	2.5	6.6	0.3	1.9	3.0	7.4
	Djibouti	2.5	4.9	5.9	8.6	0.9	4.2	6.3	13.2
	Equatorial Guinea	3.0	13.8	17.7	28.3	1.4	10.7	14.6	26.0
	Eritrea	0.6	1.4	1.8	3.2	0.1	1.0	1.8	6.8
	Eswatini	7.2	18.6	22.9	33.5	0.9	4.7	6.7	13.8
	Ethiopia	1.2	1.4	1.6	2.0	0.2	0.9	1.3	3.0
	Gabon	4.8	17.0	21.8	33.2	0.4	2.7	4.0	8.4
	Gambia	2.2	4.6	5.7	9.0	0.5	1.8	2.5	4.6
	Ghana	1.2	6.0	8.5	15.5	0.3	1.4	2.1	4.5
	Guinea	2.5	4.5	5.4	8.0	0.4	1.5	2.3	5.4
	Guinea-Bissau	1.6	3.0	3.7	5.9	0.3	1.6	2.6	6.5
	Kenya	1.6	5.8	8.0	13.9	0.4	1.9	2.8	6.1
	Lesotho	4.7	11.1	13.2	19.5	0.3	1.4	2.0	4.5
	Liberia	2.0	4.7	6.0	10.1	1.9	6.0	8.0	13.6
	Madagascar	0.3	0.8	1.1	2.1	0.3	1.3	1.8	3.8
	Malawi	1.5	3.9	5.1	8.6	0.9	3.3	4.5	8.6
	Mali	1.5	2.4	2.8	3.8	0.3	0.7	1.0	2.3
	Mauritania	8.1	17.4	21.9	31.8	2.8	8.7	12.1	20.9
	Mozambique	1.5	3.4	4.5	8.1	0.6	2.1	3.0	6.3
	Namibia	3.4	8.4	10.5	16.5	1.2	4.7	6.4	12.2
	Niger	1.5	2.1	2.3	3.1	0.2	0.5	0.6	1.1
	Nigeria	1.7	5.1	6.7	11.5	0.5	2.6	3.8	8.4
	Rwanda	1.0	2.7	3.7	6.5	0.2	0.7	1.0	2.1
	Sao Tome and Principe	2.5	7.3	9.6	15.9	0.7	3.3	4.6	8.7
	Senegal	3.1	4.2	5.1	7.6	0.5	1.6	2.4	5.2
	Sierra Leone	1.9	3.8	4.6	7.0	0.6	2.2	3.0	5.8
	Somalia	2.1	4.6	5.6	9.0	0.3	1.1	1.7	4.3
	South Africa	7.6	22.7	27.8	40.6	2.1	6.7	8.8	15.6
	South Sudan	0.8	1.1	1.2	1.6	0.2	0.8	1.4	5.8
	Togo	2.1	5.1	6.8	11.7	0.4	2.1	3.5	8.3
	Uganda	0.8	2.8	3.9	7.7	0.2	1.0	1.5	3.2
	United Republic of Tanzania	1.9	6.7	9.5	17.4	0.4	2.1	3.1	7.1
	Zambia	2.4	7.4	9.4	15.3	1.5	5.4	7.3	13.5
	Zimbabwe	2.2	7.2	9.5	16.5	0.3	1.8	2.5	5.3

Prevalence %

Best observed — Worst observed

Figure S9: Country-level heatmaps of estimated age-standardised prevalence of overweight and obesity, 1990, 2021, 2030, 2050 by sex, super-region, and age group (5-year age bands)

		5 to 9								10 to 14							
		Female				Male				Female				Male			
		1990	2021	2030	2050	1990	2021	2030	2050	1990	2021	2030	2050	1990	2021	2030	2050
South Asia	Bangladesh	3.0	6.9	8.9	14.5	2.1	4.3	5.3	8.2	3.8	8.8	11.5	18.3	2.7	5.7	6.9	10.6
	Bhutan	9.4	15.8	18.7	26.2	4.1	10.5	13.3	21.4	10.9	18.8	22.4	30.8	5.1	13.0	16.2	25.4
	India	4.7	10.6	13.0	19.3	3.9	9.2	11.5	17.6	4.2	9.9	12.2	17.9	4.5	10.7	13.2	19.8
	Nepal	6.6	18.1	22.7	33.7	3.0	9.7	13.0	22.0	5.6	10.7	13.5	21.3	4.1	11.5	15.4	25.6
	Pakistan	4.3	7.0	8.4	11.9	2.7	5.8	7.2	11.5	6.8	12.0	14.0	19.3	4.4	9.9	12.1	18.8
Southeast Asia, East Asia, and Oceania	American Samoa	45.7	64.9	69.2	77.5	46.0	64.0	67.7	75.4	48.9	67.2	71.1	79.1	49.4	66.6	70.7	77.8
	Cambodia	3.6	6.1	7.2	10.2	2.4	4.9	6.0	9.1	3.6	6.2	7.3	10.3	2.6	5.5	6.7	10.2
	China	5.3	16.0	20.8	34.4	6.5	19.4	24.3	38.5	5.5	16.6	21.6	35.4	7.1	21.0	26.3	40.8
	Cook Islands	61.4	77.3	81.2	87.5	52.7	71.1	75.2	83.1	64.3	79.4	82.9	88.7	56.3	73.8	78.0	85.1
	DPR of Korea	9.2	24.9	31.1	46.4	10.2	23.5	28.5	41.1	6.6	19.1	24.4	38.4	9.0	21.4	26.0	38.1
	Fiji	22.3	43.5	49.6	62.8	13.9	31.6	37.5	51.4	23.5	44.1	50.2	63.3	14.9	33.4	39.9	53.9
	Guam	36.3	50.2	52.9	59.7	50.0	60.9	62.5	66.6	37.5	50.9	53.8	60.5	53.0	63.8	65.5	69.4
	Indonesia	3.8	9.6	11.5	16.6	3.1	8.3	10.0	14.0	4.0	10.0	12.0	17.2	3.0	7.9	9.6	13.5
	Kiribati	26.6	50.1	57.8	72.6	32.1	56.3	63.3	76.8	28.5	52.7	59.7	74.1	36.2	60.7	67.4	79.9
	Lao PDR	4.0	7.3	8.5	11.7	2.2	5.2	6.7	10.9	5.3	9.6	11.3	15.4	2.3	5.5	7.0	11.4
	Malaysia	11.0	24.3	29.4	41.1	9.2	20.8	24.3	33.4	12.6	27.8	32.9	45.1	11.2	24.3	28.1	37.8
	Maldives	3.8	10.2	12.7	19.3	4.5	15.2	19.6	30.3	6.8	17.8	21.8	31.2	5.1	16.6	21.1	32.3
	Marshall Islands	36.8	53.5	58.1	66.4	25.7	44.1	49.4	60.0	38.4	55.5	59.8	67.9	27.5	46.3	51.5	62.1
	Mauritius	9.5	21.5	25.7	36.3	8.2	18.4	21.9	30.1	12.0	26.6	30.8	42.2	10.4	22.3	26.2	35.1
	Micronesia (Fed States)	32.4	53.8	59.4	70.9	27.1	48.4	54.5	67.4	35.7	57.4	63.5	74.4	29.8	51.1	57.5	70.0
	Myanmar	5.3	10.0	11.4	15.7	3.2	6.7	7.8	11.6	6.5	11.8	13.6	18.5	3.9	8.0	9.4	13.8
	Nauru	51.4	69.9	73.6	81.5	47.6	70.3	75.3	84.5	55.8	73.7	77.6	84.6	54.1	75.8	80.3	88.0
	Niue	37.1	61.0	67.0	77.9	39.6	62.7	68.0	77.6	39.0	62.7	68.6	79.1	42.4	64.1	69.2	78.6
	Northern Mariana Islands	42.1	65.1	70.8	80.9	50.7	70.1	74.4	82.7	44.1	66.8	72.2	81.9	53.0	71.8	76.0	83.9
	Palau	42.2	63.3	68.3	77.5	45.5	62.6	66.7	74.6	43.4	64.5	69.0	78.1	47.1	63.7	67.7	75.4
	Papua New Guinea	15.0	23.1	25.8	33.2	8.6	17.6	21.0	30.9	15.3	22.7	25.7	33.0	9.1	17.7	21.2	31.1
	Philippines	5.8	11.7	14.1	20.7	5.1	9.9	11.9	17.2	6.8	13.7	16.2	23.6	6.6	12.7	15.2	21.5
	Samoa	53.9	73.3	77.2	84.6	33.2	56.4	62.5	74.7	58.1	76.2	80.2	86.8	36.4	60.1	66.2	77.7
	Seychelles	12.6	29.5	35.5	50.1	8.5	23.6	29.6	43.6	15.7	34.9	41.7	56.6	9.7	25.7	31.7	46.1
	Solomon Islands	17.8	30.6	34.6	46.6	8.4	19.0	23.2	36.2	19.5	32.8	37.5	49.7	9.5	20.8	25.4	38.9
	Sri Lanka	9.4	20.9	23.7	31.8	5.4	11.9	14.0	20.0	8.1	17.6	20.0	27.3	5.6	12.3	14.3	20.5
	Taiwan (Province of China)	8.2	29.0	36.7	54.3	14.3	40.7	49.2	65.2	8.3	29.3	37.1	54.7	15.7	43.4	51.7	67.5
	Thailand	7.4	19.3	24.2	35.4	5.2	14.5	17.9	26.4	9.8	23.3	28.2	40.2	7.7	19.5	24.0	34.1
	Timor-Leste	3.7	8.7	11.2	18.1	3.0	10.5	14.1	25.1	4.0	9.5	12.0	19.4	2.7	9.1	12.2	22.1
	Tokelau	31.0	54.6	60.7	71.6	38.7	65.0	70.5	79.5	32.9	56.5	62.3	72.9	41.0	67.0	72.5	81.0
	Tonga	52.9	73.9	78.6	86.8	45.9	69.5	75.3	85.2	54.8	75.8	80.5	88.1	48.9	72.4	77.9	86.9
	Tuvalu	27.4	52.5	60.6	74.8	28.8	53.5	61.2	74.8	30.4	56.5	63.7	77.2	31.9	57.0	64.4	77.3
	Vanuatu	12.0	22.7	26.9	38.2	7.6	16.0	19.4	29.7	17.1	31.0	35.9	48.5	9.7	19.7	23.9	35.5
	Viet Nam	2.4	5.9	7.2	10.7	2.2	4.9	6.1	8.8	2.9	6.9	8.5	12.5	2.4	5.5	6.8	9.7

Prevalence %

Best observed — Worst observed

		15 to 19								20 to 24							
		Female				Male				Female				Male			
		1990	2021	2030	2050	1990	2021	2030	2050	1990	2021	2030	2050	1990	2021	2030	2050
South Asia	Bangladesh	3.8	8.9	11.5	18.4	2.5	5.3	6.6	10.1	7.1	21.6	26.8	38.7	5.1	11.1	13.6	19.9
	Bhutan	9.1	15.8	18.7	26.2	3.8	10.0	12.7	20.5	17.5	31.0	35.9	46.4	7.8	18.4	22.5	33.8
	India	4.0	10.1	12.3	17.8	3.6	9.1	11.2	17.0	4.5	10.4	12.5	18.3	6.0	15.4	18.6	26.8
	Nepal	4.1	8.0	10.3	16.6	3.9	10.4	13.8	23.2	7.4	14.0	17.8	27.3	8.0	19.2	24.7	38.3
	Pakistan	6.7	12.8	15.1	20.8	3.9	9.2	11.3	17.6	12.2	22.9	26.7	34.9	9.9	21.8	25.6	36.6
Southeast Asia, East Asia, and Oceania	American Samoa	42.9	61.5	66.1	74.9	41.8	59.0	63.2	71.5	61.4	77.7	80.7	86.5	59.5	75.0	77.8	83.6
	Cambodia	2.7	4.8	5.5	7.9	2.0	4.2	5.2	7.9	4.7	8.0	9.3	13.0	3.5	7.2	8.8	13.2
	China	4.5	14.2	18.6	31.4	5.3	17.1	22.0	35.0	6.4	19.1	25.0	39.3	8.4	24.2	30.8	45.7
	Cook Islands	58.6	75.8	79.8	86.4	47.8	66.8	71.5	80.2	70.7	84.7	87.2	91.7	67.7	83.1	85.9	90.8
	DPR of Korea	5.0	15.2	19.7	32.3	6.5	16.2	19.9	30.4	6.0	17.3	21.9	35.2	7.0	17.6	21.3	32.2
	Fiji	19.1	38.3	44.2	57.8	11.0	27.0	32.7	46.3	29.4	52.5	58.7	71.0	18.7	41.9	48.6	62.6
	Guam	30.4	47.4	50.5	57.4	44.4	56.6	58.3	62.6	48.9	61.5	64.1	70.1	59.6	64.6	66.4	70.3
	Indonesia	4.0	10.5	12.6	18.1	2.3	6.0	7.4	10.5	6.9	18.5	21.9	29.9	4.4	11.5	13.6	18.7
	Kiribati	23.3	44.1	51.7	67.4	28.9	53.6	60.7	74.8	51.1	77.2	81.9	89.8	41.4	67.3	73.5	84.2
	Lao PDR	5.2	9.4	10.9	14.9	1.6	3.9	5.1	8.4	7.3	13.3	15.5	20.7	3.0	7.3	9.0	14.5
	Malaysia	11.2	29.7	35.1	47.7	9.0	22.9	26.8	36.3	16.4	33.2	38.8	51.6	15.0	30.2	34.5	44.9
	Maldives	7.6	20.4	24.5	34.5	4.0	13.7	17.8	27.9	13.9	34.5	40.3	52.0	7.4	23.9	30.1	43.3
	Marshall Islands	32.8	48.6	53.0	61.7	21.1	37.8	42.6	53.4	47.2	60.2	64.4	72.2	33.4	48.8	54.1	64.5
	Mauritius	10.5	23.1	27.4	38.3	8.1	18.1	21.5	29.6	14.2	31.3	36.6	48.6	13.6	28.9	33.4	43.2
	Micronesia (Fed States)	29.9	51.4	57.4	69.1	23.4	42.9	49.0	62.4	46.3	66.9	71.7	80.9	37.3	60.1	65.7	76.8
	Myanmar	5.3	10.0	11.4	15.7	3.0	6.2	7.3	11.0	8.3	13.9	15.8	21.4	4.8	10.2	12.0	17.4
	Nauru	50.2	68.5	73.0	81.1	46.7	69.5	75.0	84.3	70.7	82.2	85.1	90.1	72.1	86.7	89.5	93.9
	Niue	33.2	56.2	63.1	74.8	34.9	56.3	61.8	72.5	48.5	71.7	76.8	85.1	49.1	70.2	74.7	82.8
	Northern Mariana Islands	38.1	61.0	67.2	78.2	45.3	64.7	69.6	78.9	54.1	75.0	79.3	87.0	60.2	77.2	80.8	87.3
	Palau	37.3	58.3	63.7	73.8	38.9	55.6	59.9	68.6	50.3	70.0	74.4	82.3	51.2	67.4	71.2	78.4
	Papua New Guinea	12.9	19.6	21.8	28.5	6.5	13.0	15.8	24.1	15.4	23.3	26.0	33.4	12.1	22.4	26.5	37.7
	Philippines	5.2	10.4	12.8	19.0	4.8	9.4	11.1	16.1	9.9	18.0	21.1	29.8	8.8	16.4	19.2	26.6
	Samoa	52.2	73.3	77.3	84.6	29.3	52.3	58.6	71.5	64.7	82.6	85.4	90.5	46.7	70.9	75.7	84.7
	Seychelles	13.3	31.0	37.7	52.5	7.0	20.3	25.3	38.5	21.8	44.7	52.0	66.3	13.8	34.5	41.3	56.3
	Solomon Islands	15.6	27.4	31.5	43.2	6.9	15.7	19.3	31.0	25.8	40.9	45.9	58.3	12.7	26.8	32.0	46.9
	Sri Lanka	6.5	14.2	16.5	22.9	4.1	9.6	11.5	16.7	11.6	21.2	24.3	32.5	7.7	17.1	19.8	27.4
	Taiwan (Province of China)	7.1	25.6	32.6	50.0	12.8	36.9	45.2	61.7	8.7	27.6	35.3	52.9	14.8	42.1	50.7	66.5
	Thailand	9.9	20.1	25.0	36.4	6.4	15.4	18.9	27.8	11.3	29.2	34.9	47.9	10.4	25.3	30.0	41.3
	Timor-Leste	3.4	8.1	10.3	16.8	1.4	4.9	6.7	12.8	5.6	13.4	16.6	25.9	1.9	6.5	8.9	16.6
	Tokelau	27.7	50.5	55.7	67.3	33.4	59.0	65.1	75.2	42.1	66.4	71.5	80.3	48.1	72.8	77.5	84.7
	Tonga	49.2	71.2	76.3	85.2	40.8	64.9	71.2	82.3	63.3	83.7	87.1	92.4	50.2	73.9	79.2	87.7
	Tuvalu	25.8	51.0	58.8	73.4	25.2	48.7	56.1	70.6	44.5	71.4	77.5	87.0	41.1	65.7	72.4	83.2
	Vanuatu	14.1	26.2	30.6	42.6	7.1	15.0	18.5	28.5	23.7	41.9	47.4	60.3	12.4	25.6	30.5	43.6
	Viet Nam	2.5	5.6	6.9	10.3	3.2	6.9	8.5	12.1	2.9	7.3	9.0	13.3	3.2	6.5	7.9	11.3

Prevalence %

Best observed — Worst observed

		5 to 9								10 to 14							
		Female				Male				Female				Male			
		1990	2021	2030	2050	1990	2021	2030	2050	1990	2021	2030	2050	1990	2021	2030	2050
Central Europe, Eastern Europe, and Central Asia	Albania	17.9	30.7	33.5	38.3	18.2	28.2	29.6	32.0	11.3	21.5	23.6	27.7	19.4	29.6	31.2	33.7
	Armenia	19.1	27.3	29.3	33.5	18.2	26.4	28.4	33.1	12.5	19.0	20.4	23.7	15.4	23.1	25.4	29.8
	Azerbaijan	14.6	24.1	26.5	32.5	14.7	21.4	23.6	27.9	14.7	23.8	26.3	32.3	17.8	25.6	27.8	32.5
	Belarus	10.5	20.7	23.2	30.0	9.0	18.8	21.1	27.6	8.6	17.7	20.0	26.2	10.2	21.5	24.3	31.3
	Bosnia and Herzegovina	17.6	33.7	37.5	45.5	16.5	28.3	30.6	35.3	8.4	18.0	20.4	26.5	15.3	26.6	28.9	33.6
	Bulgaria	13.9	20.3	22.1	25.6	18.1	25.5	27.2	30.3	12.8	19.0	20.6	24.0	19.4	26.9	28.7	31.9
	Croatia	9.7	20.0	23.3	30.1	18.4	29.8	32.8	38.3	9.5	19.3	22.6	29.3	20.6	31.9	35.1	40.8
	Czechia	12.4	18.0	19.6	23.6	20.2	25.5	27.0	30.8	11.9	17.2	18.6	22.5	21.2	27.0	28.7	32.6
	Estonia	13.7	20.6	22.9	27.9	14.5	22.5	25.1	31.2	13.8	20.5	22.8	27.9	15.8	24.3	26.8	33.1
	Georgia	15.7	20.8	22.5	25.7	12.3	16.4	17.2	19.2	17.2	24.9	26.6	30.2	23.0	30.7	31.5	34.3
	Hungary	13.8	22.6	25.5	31.7	17.6	26.8	29.1	34.5	14.0	22.7	25.5	31.8	19.3	28.9	31.4	36.9
	Kazakhstan	15.7	25.8	28.0	34.3	12.0	19.7	21.3	25.2	11.8	19.9	22.0	27.4	12.7	20.7	22.3	26.4
	Kyrgyzstan	13.6	22.4	25.3	31.4	11.5	18.2	20.2	25.3	10.9	18.6	20.8	26.3	13.0	20.3	22.5	27.9
	Latvia	9.3	14.6	16.4	20.2	11.1	18.2	20.4	25.6	9.4	15.0	16.7	20.5	12.1	19.8	22.3	27.8
	Lithuania	6.0	12.0	13.4	16.4	9.6	19.1	21.2	25.2	5.7	12.0	13.6	16.6	10.4	20.3	22.5	26.6
	Mongolia	13.8	19.7	21.2	24.5	12.5	16.6	17.6	19.7	13.3	21.1	22.5	25.9	11.8	16.4	17.2	19.4
	Montenegro	17.5	33.0	38.1	48.5	26.3	41.1	44.3	51.2	14.4	29.2	33.8	43.8	27.2	39.9	43.0	49.8
	North Macedonia	14.0	25.8	29.5	37.3	17.3	25.5	27.2	31.6	9.2	18.0	20.5	27.0	19.8	29.0	31.1	35.9
	Poland	12.6	21.7	23.9	28.6	14.5	24.4	26.4	31.0	13.0	21.9	24.1	28.8	15.8	26.3	28.5	33.3
	Republic of Moldova	7.7	15.0	17.2	23.0	6.7	13.2	15.4	21.1	9.6	19.1	22.0	28.8	12.3	24.0	27.5	35.7
	Romania	9.4	18.0	20.5	25.5	11.3	19.8	21.6	25.7	8.9	16.8	19.1	23.9	11.9	20.7	22.8	27.0
	Russian Federation	18.1	24.4	26.3	31.2	17.6	27.5	30.3	37.6	10.1	19.0	20.5	24.8	12.6	24.2	27.0	33.9
	Serbia	14.7	30.0	34.4	44.0	10.6	20.0	22.8	29.2	13.0	28.2	32.4	41.8	13.3	25.5	29.0	36.2
	Slovakia	8.9	13.6	14.9	18.4	14.4	20.3	21.7	25.4	8.6	13.0	14.4	17.7	15.1	21.4	23.0	26.9
	Slovenia	12.9	22.3	25.1	31.8	21.3	30.3	33.0	38.7	13.4	23.1	26.1	32.9	22.9	33.0	35.9	41.7
	Tajikistan	12.4	19.2	21.5	26.5	9.7	15.2	17.0	21.5	12.7	20.0	22.2	27.3	11.6	18.3	20.3	25.4
	Turkmenistan	14.1	19.7	20.7	23.5	13.9	17.9	19.1	21.6	14.8	21.0	22.4	25.3	16.9	22.0	23.3	26.1
	Ukraine	8.2	15.2	16.9	22.5	9.6	17.4	19.1	24.3	8.1	14.9	16.4	22.0	9.9	18.5	20.3	25.8
	Uzbekistan	19.7	30.3	33.4	40.3	14.3	22.7	25.2	30.9	12.9	21.8	24.4	30.4	12.3	20.2	22.4	27.7

Prevalence %

Best observed — Worst observed

		15 to 19								20 to 24							
		Female				Male				Female				Male			
		1990	2021	2030	2050	1990	2021	2030	2050	1990	2021	2030	2050	1990	2021	2030	2050
Central Europe, Eastern Europe, and Central Asia	Albania	6.9	14.3	15.8	19.0	14.7	23.6	24.8	27.0	12.4	22.4	24.4	28.5	23.0	34.5	35.9	38.5
	Armenia	8.5	12.8	13.8	16.4	10.1	15.6	17.2	20.6	14.0	18.6	19.9	23.2	15.4	26.4	28.5	33.2
	Azerbaijan	11.7	19.6	22.0	27.4	13.8	21.0	22.7	26.9	19.3	29.4	32.5	39.2	19.4	26.0	28.1	32.8
	Belarus	6.3	13.3	15.3	20.5	7.4	16.3	18.5	24.4	12.0	24.4	27.2	34.6	16.2	33.4	36.9	45.1
	Bosnia and Herzegovina	4.4	10.0	11.8	15.9	10.0	18.3	19.9	23.7	10.0	20.4	23.4	30.0	16.8	29.5	31.7	36.5
	Bulgaria	10.3	15.9	17.6	20.6	14.5	21.4	22.7	25.6	12.9	19.0	20.8	24.2	19.9	27.9	29.8	33.1
	Croatia	7.6	15.9	18.6	24.4	15.9	25.1	27.6	32.8	12.1	23.2	27.0	34.3	24.5	38.2	41.7	47.7
	Czechia	10.1	14.3	15.8	19.3	15.9	20.5	22.2	25.6	13.8	18.6	20.4	24.6	29.6	36.8	38.5	42.9
	Estonia	11.5	17.6	19.6	24.2	11.6	18.8	21.0	26.4	17.1	26.3	28.9	34.7	20.4	33.1	36.2	43.4
	Georgia	14.1	23.0	24.8	28.3	20.9	29.5	31.0	33.8	19.4	31.3	33.2	37.2	31.9	44.0	45.4	48.5
	Hungary	11.5	20.6	23.1	29.0	15.2	24.9	27.5	32.8	16.5	25.9	28.8	35.5	27.1	38.3	41.2	47.3
	Kazakhstan	6.7	12.0	13.4	17.2	9.0	14.8	16.3	19.5	11.5	20.2	22.1	27.6	17.2	26.9	28.8	33.4
	Kyrgyzstan	6.5	11.4	12.9	16.8	10.0	16.8	19.0	23.9	13.6	22.3	24.7	30.9	17.6	27.6	30.2	36.6
	Latvia	7.7	14.0	15.6	19.3	8.6	16.2	18.2	23.0	12.7	22.9	25.1	30.2	15.3	30.3	33.3	40.0
	Lithuania	4.2	10.1	11.3	13.9	8.3	16.1	17.8	21.3	10.0	20.9	23.2	27.5	17.1	31.5	34.3	39.3
	Mongolia	10.1	16.9	18.0	21.0	7.7	11.3	12.0	13.6	16.3	25.9	27.6	31.5	19.7	26.9	28.5	31.5
	Montenegro	8.7	18.6	21.8	30.1	19.7	30.5	33.2	39.6	12.6	25.7	29.6	39.2	32.2	45.7	49.0	55.8
	North Macedonia	6.5	13.7	15.9	21.4	15.1	23.0	24.9	29.2	12.1	24.0	27.2	34.8	24.7	35.5	37.9	43.0
	Poland	11.5	20.4	22.7	27.2	11.4	21.2	23.0	27.2	12.8	22.0	24.2	29.0	22.0	37.0	39.5	45.0
	Republic of Moldova	8.1	16.9	19.5	25.9	10.2	21.6	25.0	32.8	13.8	26.5	30.0	38.0	20.8	39.0	43.2	52.4
	Romania	7.1	14.4	16.3	20.6	8.9	16.7	18.5	22.3	10.6	20.0	22.4	27.7	15.6	26.6	29.2	34.1
	Russian Federation	8.9	15.7	17.5	21.3	8.6	17.6	19.7	25.4	17.9	25.4	27.5	32.6	16.3	31.1	34.2	41.8
	Serbia	9.3	21.9	25.7	34.2	10.7	20.5	23.3	29.8	14.9	32.8	37.4	47.1	18.1	34.3	38.2	46.1
	Slovakia	7.1	10.5	11.6	14.4	11.2	16.4	17.6	20.7	9.9	15.1	16.5	20.3	24.3	33.4	35.5	40.3
	Slovenia	10.7	18.9	21.8	28.0	17.8	26.7	29.2	34.6	16.8	26.5	30.0	37.4	28.1	38.8	41.9	47.9
	Tajikistan	10.0	16.0	17.5	21.9	8.7	13.8	15.7	19.9	17.3	26.3	29.0	34.9	22.5	34.7	38.2	45.3
	Turkmenistan	12.4	17.2	18.4	21.0	12.7	17.0	18.0	20.3	18.2	22.1	23.4	26.4	23.4	30.1	31.7	35.1
	Ukraine	5.8	13.9	15.4	20.7	7.0	14.2	15.5	20.1	12.8	25.6	27.7	35.3	16.5	28.7	31.0	37.8
	Uzbekistan	8.8	16.6	18.7	23.8	8.3	15.1	16.9	21.2	14.4	26.8	29.6	36.3	16.1	28.4	31.2	37.4

Prevalence %

Best observed — Worst observed

		5 to 9								10 to 14							
		Female				Male				Female				Male			
		1990	2021	2030	2050	1990	2021	2030	2050	1990	2021	2030	2050	1990	2021	2030	2050
High-income	Andorra	18.5	28.5	31.2	37.6	19.0	25.8	28.3	33.6	17.8	27.7	30.6	37.0	21.0	28.4	30.9	36.4
	Argentina	15.1	27.0	31.4	41.5	17.6	31.0	34.8	43.7	15.8	27.7	32.2	42.4	19.4	33.4	37.5	46.7
	Australia	17.0	30.3	34.0	43.2	18.8	34.9	39.6	49.6	17.4	30.6	34.5	43.7	22.8	40.2	44.9	55.1
	Austria	14.9	22.5	24.8	29.5	16.8	23.0	24.4	28.0	14.5	21.9	23.9	28.5	18.3	24.9	26.5	30.3
	Belgium	11.4	22.2	25.2	32.0	8.7	16.1	18.1	23.1	14.1	26.5	29.3	36.7	13.9	25.0	28.0	34.5
	Brunei Darussalam	3.4	9.4	12.0	20.3	4.5	12.3	15.6	24.7	4.1	12.0	15.2	24.9	7.7	20.2	24.9	36.8
	Canada	20.4	28.3	30.8	35.9	24.3	34.3	36.4	42.0	21.0	29.0	31.5	36.6	27.4	37.5	39.6	45.3
	Chile	20.4	37.1	41.1	51.3	20.4	42.1	47.1	58.2	21.3	38.2	43.1	53.3	22.5	44.7	49.9	60.9
	Cyprus	11.9	26.5	31.0	40.8	14.2	29.6	33.4	42.2	11.9	26.9	30.7	40.5	16.8	34.2	38.5	47.6
	Denmark	18.5	28.9	31.9	38.2	19.1	27.7	29.9	34.9	19.0	28.8	31.5	37.8	20.9	29.7	32.1	37.3
	Finland	16.8	27.3	30.0	36.3	18.0	29.5	32.3	38.8	17.0	28.0	31.2	37.6	20.2	32.3	35.2	41.9
	France	12.0	23.0	26.7	35.0	11.4	22.6	26.0	33.5	12.1	23.3	26.7	35.1	12.5	24.6	28.1	35.9
	Germany	19.8	27.4	29.8	34.8	23.5	30.0	31.2	34.8	19.4	27.3	29.5	34.4	25.0	31.6	33.1	36.8
	Greece	13.2	27.4	32.1	42.0	17.7	34.4	39.2	48.8	13.7	28.1	33.2	43.2	20.6	39.0	43.9	53.6
	Greenland	27.6	34.5	35.8	39.5	26.0	33.5	36.0	40.7	32.1	39.3	40.6	44.5	32.0	40.6	43.1	48.1
	Iceland	19.5	31.2	33.5	39.1	23.0	30.4	31.8	35.1	20.0	31.3	33.5	39.2	25.1	32.8	34.5	38.0
	Ireland	16.4	28.7	31.4	38.1	17.1	32.5	35.4	42.8	16.6	29.0	31.7	38.4	19.3	36.1	39.4	47.0
	Israel	15.9	24.4	27.5	34.3	16.1	22.1	24.2	29.0	15.3	23.7	26.7	33.3	18.2	25.3	27.7	32.9
	Italy	9.8	19.6	23.1	31.0	13.1	24.8	28.6	36.9	10.1	19.8	23.2	31.2	15.4	27.9	32.1	40.8
	Japan	12.6	20.0	22.6	28.6	11.7	18.7	21.0	26.2	11.4	18.7	21.3	27.1	14.5	23.1	25.5	31.3
	Luxembourg	12.7	22.5	24.9	31.0	16.6	26.6	29.2	34.9	12.2	21.6	24.1	30.2	18.4	29.2	31.7	37.7
	Malta	10.8	23.0	26.5	34.8	10.1	21.0	24.3	31.6	11.3	23.7	27.5	36.0	12.3	24.8	28.6	36.4
	Monaco	23.0	34.3	37.1	43.8	24.7	33.3	35.7	40.9	22.4	33.4	36.8	43.5	27.7	37.2	39.5	44.9
	Netherlands	14.3	25.1	28.5	35.5	12.0	19.2	21.1	25.1	14.6	25.2	28.6	35.5	13.0	20.7	22.5	26.7
	New Zealand	28.2	37.9	41.8	49.7	26.3	34.4	37.3	43.8	30.8	41.1	44.8	52.8	30.7	40.0	43.0	49.7
	Norway	17.4	27.4	30.1	36.1	18.9	27.2	29.2	33.9	17.7	27.3	29.7	35.7	20.2	29.0	31.1	35.9
	Portugal	12.1	26.6	31.3	41.5	11.4	25.1	29.0	37.1	13.8	30.0	34.9	45.5	15.6	32.2	36.4	45.3
	Republic of Korea	6.7	19.7	23.4	32.2	8.3	22.0	25.1	32.6	8.0	21.0	24.9	34.0	11.2	25.7	29.7	37.7
	San Marino	20.0	31.2	34.9	43.4	22.0	32.0	35.1	42.4	19.4	30.5	34.3	42.7	24.5	35.1	38.3	45.9
	Singapore	4.4	15.0	18.9	30.2	5.3	17.9	22.2	34.0	4.9	16.6	20.5	32.2	7.2	22.3	27.1	40.0
	Spain	11.0	23.3	27.7	37.8	15.5	31.3	35.7	45.0	11.7	24.7	29.4	39.9	17.8	35.4	39.5	48.9
	Sweden	16.7	24.4	26.9	33.2	18.2	26.1	28.7	34.5	16.7	24.8	27.5	33.8	19.7	28.4	31.1	37.1
	Switzerland	17.0	22.4	24.0	27.9	20.1	24.3	25.8	29.2	15.2	20.6	22.1	25.9	19.2	23.1	24.7	28.1
	United Kingdom	20.7	31.0	33.4	39.2	15.8	24.4	26.4	31.3	23.0	34.7	37.2	43.2	19.6	29.9	32.2	37.6
	United States of America	25.2	33.1	35.9	42.8	26.9	30.9	32.8	37.7	22.3	41.7	44.6	51.6	23.0	41.9	44.1	49.3
	Uruguay	21.3	32.1	35.7	42.4	12.9	22.6	25.4	31.7	22.5	33.7	36.7	43.5	14.4	24.8	27.5	34.0

Prevalence %

Best observed — Worst observed

		15 to 19								20 to 24							
		Female				Male				Female				Male			
		1990	2021	2030	2050	1990	2021	2030	2050	1990	2021	2030	2050	1990	2021	2030	2050
High-income	Andorra	13.9	22.1	24.7	30.4	15.8	21.7	23.6	28.4	19.5	30.0	33.0	39.6	24.1	32.4	35.2	41.0
	Argentina	12.4	26.0	29.9	39.9	14.9	26.9	30.4	39.0	21.7	41.5	46.4	57.3	25.8	44.7	49.3	58.5
	Australia	14.5	25.9	29.6	38.4	17.6	33.3	37.8	47.8	23.3	41.0	45.8	55.6	31.5	50.0	55.0	64.8
	Austria	11.3	17.7	19.2	23.2	13.8	19.4	20.8	24.1	17.7	26.3	28.6	33.7	25.2	32.1	34.0	38.2
	Belgium	12.5	23.4	26.5	33.6	11.4	20.5	23.0	28.8	18.6	32.8	36.2	44.2	19.2	32.4	35.6	42.7
	Brunei Darussalam	4.1	12.9	16.2	26.3	7.6	19.9	24.1	35.9	11.3	36.0	42.7	57.5	16.2	40.1	46.4	60.0
	Canada	17.0	26.2	28.2	33.1	21.8	30.5	32.7	38.1	23.2	38.3	41.0	46.6	33.4	49.9	52.4	58.2
	Chile	16.8	31.1	34.8	44.7	16.0	36.7	41.4	52.6	28.7	53.8	58.3	67.7	28.5	54.3	59.3	69.3
	Cyprus	10.0	22.9	26.6	35.9	14.3	29.7	33.7	42.5	14.4	31.5	36.2	46.5	19.5	38.4	42.6	51.8
	Denmark	14.0	25.7	28.4	34.4	15.7	23.9	26.0	30.7	18.3	34.0	37.4	44.1	22.8	36.3	39.0	44.5
	Finland	14.0	23.5	26.0	31.9	15.0	25.3	27.8	33.9	18.1	32.4	35.7	42.5	22.6	37.7	41.1	48.1
	France	9.5	20.6	24.0	31.9	10.8	20.4	23.5	30.7	14.4	28.7	32.7	41.9	18.6	31.7	35.8	44.5
	Germany	15.2	23.2	25.2	29.8	19.4	23.9	25.3	28.5	21.5	32.1	34.2	39.4	29.7	36.7	38.5	42.4
	Greece	13.7	24.1	28.4	37.9	19.0	35.7	40.7	50.5	18.2	33.6	38.9	49.4	28.5	47.8	52.9	62.4
	Greenland	28.1	34.5	36.1	39.8	24.8	32.9	34.9	39.6	38.9	45.8	47.8	51.7	37.2	46.8	48.7	53.7
	Iceland	14.9	27.5	30.2	35.6	17.7	26.1	27.5	30.6	23.8	40.3	43.2	49.2	27.5	39.2	40.9	44.6
	Ireland	15.5	26.8	29.4	35.9	17.3	29.6	32.7	39.9	21.6	37.4	40.4	47.6	28.2	44.4	47.6	55.3
	Israel	11.8	19.2	21.5	27.4	14.0	19.9	21.8	26.3	17.8	26.0	29.0	35.9	21.6	29.7	32.4	38.0
	Italy	9.5	15.3	18.1	24.9	13.4	23.5	27.3	35.4	12.8	24.2	28.2	37.1	20.7	35.6	40.3	49.7
	Japan	6.5	11.3	12.9	17.0	11.2	17.8	20.0	25.0	6.3	11.8	13.6	17.9	12.3	21.4	24.1	29.7
	Luxembourg	9.2	16.9	19.0	24.2	13.6	22.5	24.6	29.9	13.6	24.6	27.2	33.7	21.4	32.8	35.6	41.8
	Malta	9.8	20.7	24.0	32.0	10.0	20.5	23.6	30.7	15.7	31.0	35.2	44.6	17.0	32.9	37.0	45.6
	Monaco	17.4	27.1	29.7	35.8	21.7	29.6	32.0	37.1	25.7	37.9	41.2	48.1	33.3	43.7	46.2	51.7
	Netherlands	12.0	22.6	25.6	32.2	9.7	17.1	19.1	22.9	19.4	38.7	42.6	50.6	19.4	28.2	30.7	35.8
	New Zealand	24.6	40.4	44.1	52.2	24.5	36.7	39.7	46.3	33.1	53.0	56.9	64.6	39.9	55.6	58.7	65.1
	Norway	14.5	22.9	25.2	30.7	15.5	22.1	24.1	28.4	23.3	32.1	34.9	41.2	26.0	33.7	35.9	41.1
	Portugal	12.1	25.2	30.0	40.1	10.8	23.3	26.8	34.7	16.6	30.6	35.9	46.6	18.3	34.4	38.6	47.5
	Republic of Korea	4.4	12.3	15.1	21.9	7.5	17.2	20.0	26.5	3.6	11.4	13.8	20.3	8.9	20.0	23.2	30.4
	San Marino	14.9	24.3	27.7	35.4	18.9	28.4	31.5	38.5	22.1	34.6	38.5	47.2	29.8	41.8	45.4	53.1
	Singapore	3.9	13.1	16.4	26.7	5.6	17.6	22.0	33.7	7.0	21.3	26.3	39.6	11.1	29.7	35.8	49.7
	Spain	10.4	20.8	24.7	34.4	17.5	28.2	31.9	40.8	15.6	29.8	34.8	45.9	26.9	40.9	45.0	54.6
	Sweden	14.2	22.0	24.6	30.6	16.4	21.9	24.1	29.4	18.3	31.7	34.8	41.8	21.3	34.3	37.5	44.0
	Switzerland	11.3	16.3	17.7	20.9	13.2	18.2	19.5	22.4	16.3	24.9	26.5	30.7	20.9	29.4	31.0	34.8
	United Kingdom	20.7	32.6	35.1	41.0	18.2	28.2	30.4	35.6	26.9	42.4	45.1	51.3	30.6	43.8	46.4	52.3
	United States of America	22.9	45.6	48.7	55.8	26.1	40.2	42.4	47.6	29.3	56.4	59.5	66.0	37.1	53.6	55.7	60.7
	Uruguay	19.4	31.0	34.0	40.7	10.9	20.9	23.6	29.6	20.3	35.7	38.9	45.8	20.0	35.9	39.7	47.1

Prevalence %

Best observed — Worst observed

Latin America and Caribbean

	5 to 9								10 to 14							
	Female				Male				Female				Male			
	1990	2021	2030	2050	1990	2021	2030	2050	1990	2021	2030	2050	1990	2021	2030	2050
Antigua and Barbuda	17.8	32.5	37.2	49.0	12.2	24.1	28.3	39.2	20.6	37.0	42.3	54.3	16.5	31.6	36.5	48.5
Bahamas	21.6	38.4	43.4	54.9	15.7	30.0	35.1	47.2	25.4	42.9	48.2	59.7	21.0	38.5	44.1	56.6
Barbados	24.7	43.5	48.8	60.1	8.9	19.6	23.7	33.4	26.6	45.5	50.9	62.0	16.9	33.2	38.3	50.0
Belize	17.1	29.3	32.5	40.1	9.9	21.2	24.7	34.2	25.2	40.5	44.2	52.3	16.0	32.2	36.8	47.9
Bermuda	22.9	41.3	46.0	57.0	20.0	38.4	44.0	56.2	24.0	43.1	48.0	59.0	24.7	44.6	50.1	62.0
Bolivia	22.3	40.2	45.4	56.6	15.8	32.1	36.8	49.1	22.6	41.0	46.0	57.1	17.1	33.7	39.0	51.4
Brazil	13.9	28.7	33.7	46.3	12.3	29.6	36.2	52.2	13.1	27.8	32.7	45.0	12.4	30.7	37.2	52.8
Colombia	9.8	19.8	23.6	32.6	6.8	17.6	22.0	32.5	12.2	24.9	28.9	38.8	8.9	22.1	27.0	38.5
Costa Rica	20.2	34.4	38.9	48.3	17.4	34.5	39.0	49.3	20.8	35.7	39.7	49.2	19.1	36.9	41.9	52.3
Cuba	16.9	32.6	38.2	50.3	10.0	22.4	26.3	37.8	20.7	38.1	43.7	55.9	15.2	30.7	36.2	49.0
Dominica	28.8	46.6	52.0	62.8	16.1	33.5	39.3	52.9	30.1	48.1	53.2	63.9	16.4	33.9	39.6	53.2
Dominican Republic	11.8	24.6	28.9	39.4	6.5	14.7	17.8	26.0	14.9	29.9	34.7	45.9	9.7	21.4	25.6	35.7
Ecuador	14.2	28.2	31.9	41.0	10.2	25.8	31.0	43.0	17.3	31.0	35.0	44.4	12.8	29.4	34.7	47.0
El Salvador	12.3	24.2	28.4	37.8	7.3	17.5	21.7	32.3	21.3	37.7	42.6	53.2	10.0	23.5	28.2	40.3
Grenada	12.9	25.4	29.8	41.2	7.2	17.3	21.3	32.6	16.1	30.7	35.4	47.5	10.1	23.4	28.7	41.8
Guatemala	11.2	20.4	24.0	32.7	9.9	20.4	24.9	36.7	14.1	25.0	29.0	38.5	10.4	22.4	27.3	39.7
Guyana	13.2	23.9	28.1	37.1	6.3	12.8	15.6	22.2	15.5	27.3	32.0	41.5	9.4	18.6	22.5	30.8
Haiti	6.6	12.2	14.5	21.1	4.3	10.6	13.4	22.0	8.1	15.0	17.4	25.0	4.8	11.7	14.5	23.7
Honduras	11.0	19.2	21.6	28.5	7.2	14.2	16.8	23.9	13.5	23.1	25.8	33.4	8.5	16.7	19.3	27.2
Jamaica	21.7	37.7	42.3	52.6	9.1	18.8	22.1	30.6	23.3	39.5	44.3	54.6	9.9	20.4	23.9	32.8
Mexico	18.6	33.4	38.5	50.5	14.6	32.9	38.7	52.0	21.1	41.0	46.7	58.7	20.1	42.1	48.3	61.5
Nicaragua	12.0	22.3	26.0	34.6	10.5	22.5	26.7	37.4	14.1	25.3	29.3	38.4	11.6	24.4	28.7	39.7
Panama	12.9	26.8	31.8	44.3	9.6	22.5	27.8	41.4	10.5	22.5	27.2	39.0	6.5	16.0	20.2	31.8
Paraguay	15.1	29.7	33.4	42.3	12.2	26.3	31.2	41.1	14.9	29.7	34.0	43.0	13.4	28.1	32.8	42.9
Peru	24.1	40.1	43.9	52.8	19.4	32.8	36.2	44.2	19.9	34.5	38.4	47.2	17.8	31.2	34.5	42.4
Puerto Rico	27.5	44.4	47.9	54.9	27.8	45.2	49.1	56.5	28.4	45.4	48.5	55.4	30.6	49.2	52.8	60.0
Saint Kitts and Nevis	17.6	33.9	39.1	51.6	12.1	26.1	31.3	44.1	21.0	38.7	44.1	56.7	16.4	34.4	40.4	53.9
Saint Lucia	9.1	18.2	22.0	31.7	4.5	10.5	13.2	20.7	13.4	26.0	31.1	42.5	7.8	17.5	21.3	31.7
St Vincent & Grenadines	11.3	22.5	26.9	37.1	5.8	13.4	16.6	25.3	13.4	26.3	31.2	42.1	8.3	18.6	23.1	33.7
Suriname	6.3	12.4	14.4	20.7	3.5	7.4	8.9	13.5	9.2	17.8	20.7	28.6	6.7	13.0	15.5	22.5
Trinidad and Tobago	10.2	20.2	23.7	32.8	7.5	16.0	18.9	27.4	18.3	32.9	37.6	48.6	16.0	30.8	35.4	46.9
US Virgin Islands	36.8	50.5	53.6	61.2	27.9	42.3	45.3	53.0	37.7	52.1	55.9	63.3	30.7	45.6	48.5	56.1
Venezuela	18.2	32.6	37.8	50.6	16.0	30.9	37.1	51.8	21.2	37.7	43.2	56.1	18.8	35.9	42.2	57.0

Prevalence %

Best observed — Worst observed

	15 to 19								20 to 24							
	Female				Male				Female				Male			
Latin America and Caribbean	1990	2021	2030	2050	1990	2021	2030	2050	1990	2021	2030	2050	1990	2021	2030	2050
Antigua and Barbuda	17.3	31.8	36.8	48.5	13.5	26.4	31.1	42.5	25.8	44.8	50.1	61.8	23.0	41.2	47.0	59.1
Bahamas	21.6	37.6	42.8	54.3	17.2	33.0	38.2	50.6	31.3	50.8	55.5	66.5	28.5	48.3	53.9	65.8
Barbados	23.2	39.9	44.9	56.3	16.6	32.7	38.2	49.9	34.6	54.5	59.8	70.1	28.2	49.0	54.6	65.8
Belize	25.3	40.7	44.6	52.8	14.6	29.9	34.4	45.4	37.1	54.6	58.3	65.9	24.9	44.8	50.0	61.1
Bermuda	20.0	35.5	40.1	51.2	19.1	37.3	42.5	54.8	29.8	48.2	53.3	64.0	32.0	54.5	60.0	71.0
Bolivia	16.2	31.6	36.5	47.5	11.8	25.1	29.4	40.9	21.9	40.1	45.3	56.5	19.7	38.3	43.6	56.1
Brazil	11.2	25.2	29.8	41.9	10.0	28.0	34.2	49.7	18.6	35.8	41.8	55.0	17.7	39.3	46.4	62.5
Colombia	11.9	24.9	29.0	38.9	7.9	21.1	26.0	37.4	18.5	40.4	45.8	56.6	15.7	36.1	42.5	55.3
Costa Rica	16.2	29.0	33.0	42.1	14.2	29.0	33.8	43.8	25.2	41.9	46.1	55.6	28.7	49.9	54.9	64.7
Cuba	18.1	34.8	40.0	52.1	13.2	28.3	33.4	45.9	29.5	48.5	54.4	66.0	22.9	43.2	49.1	62.0
Dominica	24.7	41.5	46.5	57.6	11.7	26.1	31.3	44.3	35.0	54.4	59.2	69.3	19.1	38.5	44.6	58.2
Dominican Republic	13.7	28.2	32.9	44.0	9.3	20.0	23.8	33.6	20.5	41.4	46.7	58.3	17.3	32.2	37.3	48.9
Ecuador	20.9	35.1	39.4	49.1	10.9	25.9	30.7	42.6	28.5	44.3	48.7	58.3	22.0	43.6	49.4	61.8
El Salvador	23.2	40.0	45.2	55.8	10.3	24.0	29.3	41.6	32.6	52.4	58.0	67.8	22.8	44.2	50.9	63.9
Grenada	13.1	26.0	30.3	41.8	7.8	19.2	23.7	35.7	22.6	40.2	45.7	58.1	14.2	31.0	37.1	51.1
Guatemala	14.9	27.9	32.1	42.1	8.3	20.7	25.1	37.0	26.4	43.3	48.4	59.1	19.6	40.1	46.4	60.1
Guyana	13.1	22.7	26.8	35.7	8.6	16.9	20.4	28.3	21.6	36.4	42.1	52.3	16.8	30.8	36.4	46.6
Haiti	7.2	13.3	15.4	22.3	3.7	9.0	11.2	18.7	13.4	24.0	27.7	37.6	5.8	13.9	17.0	27.2
Honduras	11.6	20.3	23.2	30.4	6.3	12.9	15.2	21.9	16.7	28.2	32.1	40.6	13.7	25.4	29.3	39.2
Jamaica	19.4	34.9	39.5	49.8	8.3	17.6	21.1	29.4	30.4	49.6	54.2	64.1	15.9	30.7	35.2	45.7
Mexico	21.1	42.9	48.8	60.7	19.1	42.9	49.4	62.5	32.8	56.1	61.1	71.8	31.1	56.4	62.5	73.9
Nicaragua	15.7	27.7	31.9	41.3	10.3	21.7	26.3	36.9	32.2	49.5	54.4	64.1	22.6	41.5	47.1	59.3
Panama	7.3	16.5	20.3	30.4	4.0	10.3	13.1	21.9	11.1	24.1	28.4	40.4	7.2	18.1	22.5	34.8
Paraguay	11.7	24.6	28.2	36.6	9.9	21.6	25.6	34.7	19.0	36.1	40.7	50.1	18.5	36.3	41.6	52.2
Peru	13.7	25.9	28.9	37.0	11.9	22.3	25.2	32.1	22.7	44.5	48.7	57.7	22.9	41.0	45.0	53.2
Puerto Rico	24.4	37.6	40.8	47.8	23.5	40.6	44.5	52.0	31.3	51.1	54.3	61.2	38.0	54.1	57.8	64.8
Saint Kitts and Nevis	17.0	33.4	38.7	51.2	13.3	29.0	34.0	47.1	26.4	46.8	52.6	64.8	23.0	43.9	50.1	63.3
Saint Lucia	12.7	25.0	29.2	40.3	7.6	17.0	20.7	31.0	21.9	39.8	45.3	57.5	14.1	29.9	35.5	48.4
St Vincent & Grenadines	10.9	22.2	26.7	36.9	6.6	15.3	18.8	28.1	17.8	33.5	38.9	50.5	11.8	25.8	30.7	42.7
Suriname	11.1	21.5	24.7	33.6	8.7	17.7	20.8	29.3	16.8	30.5	34.5	44.6	15.2	28.7	32.6	43.2
Trinidad and Tobago	19.1	32.9	37.6	48.6	16.6	31.3	36.3	47.9	29.2	45.5	50.6	61.7	27.5	46.1	51.6	63.1
US Virgin Islands	31.4	45.7	48.7	56.5	24.3	38.2	41.1	48.7	45.3	62.1	65.1	71.8	36.0	49.7	53.0	60.5
Venezuela	18.8	33.9	39.3	52.1	14.5	29.5	35.2	49.7	29.3	48.3	53.7	66.0	26.0	45.9	52.6	66.8

Prevalence %

Best observed — Worst observed

		5 to 9								10 to 14							
		Female				Male				Female				Male			
		1990	2021	2030	2050	1990	2021	2030	2050	1990	2021	2030	2050	1990	2021	2030	2050
North Africa and Middle East	Afghanistan	11.7	15.7	17.0	21.9	4.5	11.4	13.9	24.3	14.5	19.2	20.8	26.4	6.7	16.2	19.6	32.5
	Algeria	13.2	33.6	41.2	58.4	6.5	20.6	26.7	42.5	15.3	37.6	46.0	62.9	8.0	24.7	31.9	48.5
	Bahrain	25.1	55.2	63.3	76.5	15.8	39.6	46.7	61.1	31.1	63.2	70.4	81.7	21.2	48.7	56.5	69.7
	Egypt	19.5	47.0	55.9	72.4	14.8	41.4	51.1	69.7	22.7	52.4	61.0	76.4	14.2	40.3	49.7	68.5
	Iran (Islamic Republic of)	13.3	39.6	48.3	65.8	7.6	28.5	36.3	54.8	14.3	41.0	49.8	67.2	8.6	31.2	39.8	58.3
	Iraq	13.8	33.4	39.0	51.9	9.0	26.2	31.5	43.6	15.8	37.1	43.1	56.0	12.9	34.8	41.1	53.5
	Jordan	11.7	33.0	39.2	53.1	9.0	23.2	27.5	37.7	19.3	46.5	53.2	66.1	14.8	34.9	39.8	50.9
	Kuwait	25.4	57.1	63.5	75.0	26.3	55.1	61.0	71.6	28.0	60.8	67.1	77.7	30.3	60.4	66.1	75.8
	Lebanon	16.7	37.8	43.2	56.6	16.0	39.4	45.3	58.5	19.3	42.2	47.9	61.0	24.3	53.5	59.3	70.7
	Libya	15.0	44.6	53.8	71.5	12.1	36.7	45.3	63.5	19.0	51.3	60.1	76.2	15.2	43.8	52.6	69.7
	Morocco	17.9	33.4	38.9	49.6	11.3	25.0	30.8	44.2	17.6	34.0	39.5	50.2	9.7	22.3	27.6	40.3
	Oman	14.6	43.6	52.2	69.3	10.6	36.6	44.6	62.6	16.9	48.2	56.8	73.0	12.9	42.2	50.5	67.6
	Palestine	10.8	27.7	32.0	42.2	8.5	22.3	25.8	35.1	14.6	37.1	41.7	52.2	11.9	30.0	34.3	44.5
	Qatar	19.5	50.4	58.1	72.7	23.0	50.0	57.1	70.2	21.5	53.0	60.7	74.7	29.9	59.2	65.5	76.8
	Saudi Arabia	23.7	57.5	64.4	76.1	17.9	49.4	56.2	69.2	26.9	61.6	68.2	78.9	18.5	50.6	57.8	70.5
	Sudan	7.9	18.8	22.5	32.2	4.0	13.0	16.3	26.4	14.6	32.5	37.7	49.6	6.2	19.3	24.0	36.7
	Syrian Arab Republic	17.8	49.9	58.0	73.9	12.1	42.4	51.2	69.2	18.5	51.0	59.3	74.7	14.4	46.8	55.6	72.7
	Tunisia	12.5	35.6	44.2	62.9	9.6	31.7	39.8	57.5	14.3	38.9	47.9	66.0	10.7	32.6	40.5	58.1
	Türkiye	12.7	30.0	36.2	49.3	10.6	31.1	37.0	49.7	13.3	30.8	37.0	50.1	12.8	35.5	41.6	54.2
	United Arab Emirates	24.1	60.0	67.9	80.7	26.9	60.8	68.0	80.1	25.7	62.6	70.1	82.2	31.1	65.5	72.0	83.0
	Yemen	14.9	24.2	27.4	36.8	5.6	21.8	28.5	47.7	10.4	18.3	21.1	29.2	6.3	24.4	31.4	51.0

Prevalence %

Best observed — Worst observed

		15 to 19								20 to 24							
		Female				Male				Female				Male			
		1990	2021	2030	2050	1990	2021	2030	2050	1990	2021	2030	2050	1990	2021	2030	2050
North Africa and Middle East	Afghanistan	11.9	16.0	17.3	22.3	5.1	12.7	15.6	26.7	20.7	33.0	35.1	42.5	11.8	26.8	31.6	47.5
	Algeria	12.5	33.1	40.9	58.1	5.8	18.8	24.7	39.9	19.6	44.0	52.1	68.4	11.0	30.7	38.6	55.8
	Bahrain	30.7	59.9	67.1	79.4	18.3	42.7	49.8	63.9	35.8	64.7	72.2	83.0	25.9	55.7	62.9	74.9
	Egypt	20.5	46.2	55.1	71.9	9.6	28.5	37.3	56.8	36.0	63.0	70.8	83.5	19.4	47.2	57.0	74.6
	Iran (Islamic Republic of)	12.2	36.2	45.0	63.0	6.2	24.4	31.7	50.0	22.1	50.2	58.9	74.7	13.3	41.5	50.8	68.3
	Iraq	13.7	33.7	38.9	51.9	11.3	31.3	37.0	49.4	25.7	51.6	57.7	69.3	19.8	44.4	50.6	62.5
	Jordan	18.0	40.7	47.2	60.9	12.5	27.6	32.1	42.9	31.3	56.7	62.8	74.3	22.4	41.7	46.8	57.8
	Kuwait	23.2	53.7	60.6	72.8	28.8	52.3	58.3	69.5	34.7	66.8	72.5	81.8	42.8	68.6	73.4	81.6
	Lebanon	15.4	35.1	40.6	54.0	18.8	47.4	53.3	65.7	24.6	51.3	57.1	69.0	31.4	62.5	67.9	77.6
	Libya	15.7	46.2	55.7	73.1	11.8	36.2	44.6	62.8	24.4	58.7	67.5	81.5	20.5	52.1	61.1	76.2
	Morocco	15.0	27.4	32.6	42.9	6.8	15.3	19.1	29.6	24.4	40.5	46.3	57.2	10.6	26.9	33.1	46.8
	Oman	13.4	40.1	48.3	66.0	9.5	33.0	41.3	59.5	27.9	58.0	65.8	79.6	19.4	52.3	60.4	75.4
	Palestine	15.7	38.6	43.0	53.5	11.8	29.6	34.1	44.2	27.3	55.1	59.7	69.0	23.3	48.6	53.8	63.6
	Qatar	16.8	46.5	54.2	69.6	24.3	55.1	61.8	74.0	30.9	63.2	70.0	81.5	37.8	66.6	72.3	81.8
	Saudi Arabia	23.2	51.2	58.7	71.6	18.1	48.2	55.3	68.4	35.9	61.4	67.9	78.8	30.2	61.3	67.5	78.0
	Sudan	15.6	36.8	42.6	54.6	5.2	16.4	20.4	32.1	29.0	55.7	60.9	71.5	10.1	27.9	33.7	48.1
	Syrian Arab Republic	15.0	43.6	52.0	69.0	10.9	38.9	47.8	66.4	24.5	60.2	67.7	81.0	20.3	57.0	65.3	80.0
	Tunisia	11.4	35.2	43.9	62.4	7.8	26.1	33.4	50.8	18.3	46.8	55.8	72.7	13.6	39.1	47.9	65.0
	Türkiye	10.5	30.7	37.1	50.4	10.4	27.2	32.7	45.2	22.8	44.1	51.0	63.7	18.6	42.4	48.9	61.2
	United Arab Emirates	25.2	52.5	60.8	75.7	29.4	54.6	62.4	76.1	26.2	64.1	71.6	83.2	35.6	69.7	75.7	85.4
	Yemen	7.0	11.9	13.9	20.0	4.6	18.6	25.0	43.2	13.1	21.7	25.0	34.1	7.6	28.1	35.8	56.0

Prevalence %

Best observed — Worst observed

Region	Country	5 to 9 Female 1990	5 to 9 Female 2021	5 to 9 Female 2030	5 to 9 Female 2050	5 to 9 Male 1990	5 to 9 Male 2021	5 to 9 Male 2030	5 to 9 Male 2050	10 to 14 Female 1990	10 to 14 Female 2021	10 to 14 Female 2030	10 to 14 Female 2050	10 to 14 Male 1990	10 to 14 Male 2021	10 to 14 Male 2030	10 to 14 Male 2050
Sub-Saharan Africa	Angola	14.3	28.2	33.5	46.4	8.3	21.8	26.6	41.0	13.8	27.2	32.4	45.2	8.6	22.1	27.3	41.8
	Benin	9.6	14.1	16.1	21.9	6.2	14.1	18.6	31.4	10.6	15.4	17.5	23.6	5.6	12.9	16.8	28.9
	Botswana	10.8	23.0	28.0	40.0	6.3	19.4	25.2	40.8	10.8	23.8	28.3	40.4	4.7	14.9	19.5	33.3
	Burkina Faso	10.7	13.8	14.7	17.3	9.5	15.9	18.3	24.7	9.1	12.0	13.0	15.2	7.8	12.7	14.6	20.1
	Burundi	6.8	10.8	12.8	18.6	4.3	8.4	10.9	18.7	6.6	10.2	12.1	17.8	4.4	8.4	10.9	18.7
	Cabo Verde	7.2	15.3	18.7	25.9	4.9	12.8	16.3	25.4	8.2	17.8	21.6	29.5	4.8	13.1	16.7	26.0
	Cameroon	13.6	27.5	32.7	44.7	10.3	25.5	31.9	47.9	15.1	29.2	34.7	46.9	9.1	22.9	28.5	44.0
	Central African Republic	13.0	22.5	27.5	41.3	7.4	19.3	27.3	48.9	13.1	23.0	28.4	42.3	8.1	20.5	29.0	51.0
	Chad	8.2	11.0	11.8	15.0	5.4	9.6	11.4	17.7	7.4	10.1	11.1	14.2	5.0	9.2	10.8	16.8
	Comoros	19.5	37.3	42.9	55.1	11.3	33.2	41.3	59.4	17.5	34.0	39.2	51.2	12.2	34.7	42.8	60.8
	Congo	8.8	23.5	29.1	44.1	5.6	15.1	19.3	31.2	13.8	33.2	40.3	56.3	6.2	16.9	21.2	33.8
	Côte d'Ivoire	3.1	5.6	6.5	9.1	2.7	7.6	10.0	17.8	3.4	6.3	7.4	10.3	2.5	7.1	9.4	16.9
	Djibouti	15.0	23.6	27.3	34.2	7.0	17.4	22.4	35.0	16.8	25.7	29.2	36.4	7.7	18.5	23.7	36.7
	DR Congo	6.6	18.0	23.1	36.7	4.7	12.8	16.8	28.1	6.5	17.0	21.7	34.9	4.3	11.5	14.9	25.3
	Equatorial Guinea	25.7	53.4	59.6	71.8	17.1	49.0	56.3	70.2	25.0	52.6	59.0	71.2	17.4	48.4	55.7	69.7
	Eritrea	5.5	9.5	11.1	16.0	2.8	9.3	13.3	26.9	4.8	8.7	10.1	14.6	2.4	8.0	11.6	24.0
	Eswatini	18.2	34.2	39.1	50.8	9.4	23.9	29.4	43.8	20.7	38.1	43.3	55.1	6.3	16.9	21.3	33.6
	Ethiopia	7.3	9.8	10.5	12.4	3.1	7.8	10.1	17.2	8.0	10.5	11.3	13.3	3.1	8.0	10.3	17.5
	Gabon	12.2	27.5	33.0	45.0	6.0	16.5	20.7	30.4	15.9	34.4	40.5	52.9	7.3	20.3	24.9	35.6
	Gambia	2.8	4.1	4.8	6.6	1.8	3.5	4.3	6.4	5.6	8.9	10.3	14.1	2.4	4.8	5.9	8.6
	Ghana	5.4	12.3	14.9	21.0	4.8	10.0	12.5	18.5	7.8	17.5	20.6	28.1	4.7	9.5	11.7	17.4
	Guinea	7.4	9.5	10.7	14.3	3.9	7.6	9.6	15.9	9.0	12.9	14.7	19.3	3.8	8.1	10.4	17.1
	Guinea-Bissau	7.4	10.9	12.6	17.2	4.6	10.4	13.5	22.9	7.3	10.9	12.4	17.0	4.4	10.3	13.6	23.0
	Kenya	8.2	16.0	19.7	28.5	4.9	11.5	14.3	22.2	9.9	20.2	24.6	34.6	5.6	13.5	16.7	25.5
	Lesotho	10.0	18.1	20.8	27.9	5.3	13.7	16.8	26.4	13.1	23.0	25.9	33.9	3.7	10.0	12.2	19.8
	Liberia	9.2	16.2	19.0	26.2	7.7	15.5	18.8	26.7	10.1	18.2	21.4	29.2	7.9	15.9	19.2	27.2
	Madagascar	6.9	12.4	14.4	20.4	4.9	9.6	11.6	17.4	4.4	7.8	9.5	13.8	4.7	9.3	11.4	17.2
	Malawi	16.8	28.7	32.3	41.2	12.0	25.1	29.0	38.6	14.4	25.5	28.9	37.5	10.4	21.1	24.8	33.6
	Mali	5.8	7.5	8.4	11.3	3.9	6.5	7.7	11.7	7.0	9.4	10.5	13.9	4.0	6.9	8.2	12.4
	Mauritania	8.7	15.6	18.9	26.3	5.0	10.9	13.9	21.5	16.5	28.0	32.9	42.7	9.1	19.0	23.5	34.4
	Mozambique	11.5	19.0	22.6	31.7	7.5	15.4	19.3	29.4	8.7	14.4	17.3	25.0	5.3	11.0	14.0	22.1
	Namibia	8.5	18.1	20.9	28.3	6.6	17.9	21.5	31.4	10.3	20.5	23.3	31.2	6.2	15.5	18.9	27.9
	Niger	5.6	7.1	7.7	9.3	3.7	5.7	6.5	9.4	6.1	7.6	8.1	9.7	3.7	5.8	6.8	9.7
	Nigeria	6.8	12.8	15.2	21.2	5.3	12.2	15.1	23.9	7.8	15.8	18.6	25.5	6.4	16.0	19.6	30.1
	Rwanda	14.3	23.4	27.1	36.4	7.7	15.4	18.7	26.9	11.6	19.1	22.6	31.1	5.2	10.2	12.6	18.7
	Sao Tome and Principe	8.1	16.3	19.7	28.1	5.3	12.3	15.0	23.0	11.0	21.8	25.7	35.5	6.7	15.4	18.6	27.9
	Senegal	7.3	11.2	12.5	16.3	4.2	8.6	10.9	17.2	11.1	16.7	18.7	23.7	4.5	9.4	11.9	18.7
	Sierra Leone	12.5	16.8	19.2	25.6	7.8	14.3	17.2	25.6	10.1	14.6	16.8	22.6	7.9	16.0	19.2	28.1
	Somalia	8.6	12.8	14.4	20.1	5.1	10.2	12.3	20.3	9.3	14.1	16.2	22.4	5.0	10.0	12.3	20.2
	South Africa	16.1	30.8	35.7	47.7	10.7	23.2	26.9	35.9	16.0	30.9	35.8	47.8	8.6	19.5	22.7	31.0
	South Sudan	13.8	16.1	17.1	20.2	7.0	13.9	18.5	33.3	10.7	12.7	13.5	16.1	5.9	11.5	15.4	28.9
	Togo	5.9	10.7	13.3	19.8	4.2	10.2	13.6	22.9	7.5	13.7	16.6	24.2	4.3	10.4	13.8	23.2
	Uganda	12.6	23.7	27.6	38.0	7.7	16.5	19.4	28.4	11.1	21.7	25.4	35.3	4.9	10.9	13.1	19.9
	Unt Republic Tanzania	9.5	18.8	23.2	33.6	7.2	15.3	18.4	27.3	8.6	17.1	21.2	31.0	6.7	14.2	17.4	25.8
	Zambia	15.2	27.2	30.9	39.9	13.7	25.6	30.0	40.5	14.8	27.6	31.6	40.7	12.8	23.5	27.5	37.6
	Zimbabwe	6.9	13.2	15.8	23.0	4.2	8.8	10.6	16.0	8.4	18.3	21.5	30.3	3.5	7.8	9.5	14.5

Prevalence %

Best observed — Worst observed

Sub-Saharan Africa	15 to 19								20 to 24							
	Female				Male				Female				Male			
	1990	2021	2030	2050	1990	2021	2030	2050	1990	2021	2030	2050	1990	2021	2030	2050
Angola	8.8	18.3	22.2	33.0	4.3	12.2	15.7	26.3	10.0	20.5	24.9	36.3	5.4	15.1	19.0	31.1
Benin	9.0	13.1	15.3	20.9	3.5	8.6	11.6	20.9	14.5	20.0	22.7	30.1	7.8	17.9	23.1	37.6
Botswana	9.4	20.7	24.9	36.3	2.3	7.8	10.5	19.5	12.8	27.3	32.4	45.0	3.9	12.9	17.1	29.8
Burkina Faso	6.0	7.3	7.9	9.5	5.0	7.6	8.9	12.6	10.0	11.4	12.2	14.4	7.3	11.3	13.0	18.0
Burundi	4.4	6.8	8.2	12.2	2.3	4.4	5.7	10.3	6.3	9.4	11.2	16.5	3.5	6.5	8.2	14.4
Cabo Verde	8.0	17.2	20.7	28.4	3.9	10.5	13.5	21.4	14.0	28.0	33.1	42.8	8.0	20.3	25.7	37.5
Cameroon	13.2	26.4	31.4	43.3	5.7	15.6	20.4	33.5	21.0	36.3	42.2	55.0	10.6	26.8	33.5	49.7
Central African Republic	9.5	16.9	21.5	33.8	4.6	12.4	18.2	36.5	11.1	19.8	24.7	37.8	5.0	13.9	20.1	39.3
Chad	5.3	7.1	7.9	10.2	2.9	5.2	6.3	10.1	7.9	10.3	11.3	14.5	5.2	9.1	10.8	16.7
Comoros	12.7	26.2	30.7	42.0	8.9	27.4	34.7	52.5	17.3	33.6	38.7	50.7	10.6	31.5	39.2	57.3
Congo	13.8	33.4	40.0	56.0	4.4	12.8	16.5	27.4	13.4	33.6	40.8	56.9	8.6	22.4	27.8	42.1
Côte d’Ivoire	6.7	12.3	14.3	19.3	4.8	12.0	16.0	27.0	17.3	26.8	30.2	38.2	11.4	24.8	31.3	46.9
Djibouti	14.2	21.8	24.4	30.9	5.5	13.9	17.9	29.1	12.7	19.7	22.4	28.6	6.3	15.2	19.6	31.3
DR Congo	4.3	11.7	15.2	25.9	1.7	4.8	6.5	11.9	6.5	16.3	21.1	34.2	2.4	6.6	8.8	15.8
Equatorial Guinea	15.5	37.8	44.0	58.2	8.6	29.6	36.5	52.4	16.7	40.4	46.3	60.3	9.8	33.1	39.8	55.6
Eritrea	3.8	6.8	8.0	11.7	1.5	5.1	7.3	16.1	4.7	7.8	9.2	13.4	2.1	6.9	9.8	20.9
Eswatini	18.2	34.4	39.5	51.2	3.5	10.2	13.2	22.2	29.6	50.5	55.8	67.0	7.0	19.0	23.6	36.6
Ethiopia	6.9	8.5	9.1	10.8	2.2	5.6	7.3	12.8	9.9	10.1	10.8	12.8	3.4	8.5	11.0	18.6
Gabon	14.7	32.3	38.0	50.5	5.6	15.9	19.9	29.5	24.1	46.2	52.6	64.5	10.7	28.1	34.0	46.0
Gambia	6.9	10.9	12.4	16.8	3.1	6.5	7.8	11.3	13.1	21.3	24.3	31.3	8.4	17.3	20.1	27.5
Ghana	8.4	15.5	18.5	25.5	3.1	6.3	7.8	11.9	14.7	30.8	35.7	45.5	7.0	12.8	15.6	22.7
Guinea	8.4	12.9	14.5	19.1	2.5	5.4	7.0	11.8	14.0	21.0	23.4	29.9	4.8	10.0	12.9	20.7
Guinea-Bissau	6.5	9.8	11.2	15.4	3.0	7.0	9.3	16.4	11.8	16.8	19.1	25.4	5.9	13.2	16.9	27.9
Kenya	8.5	19.7	23.8	33.7	4.7	11.2	13.9	21.6	14.4	30.4	35.7	47.4	8.4	18.8	23.2	33.9
Lesotho	15.0	26.2	29.5	38.1	1.6	4.3	5.4	9.2	25.7	41.7	45.7	55.3	3.1	8.3	10.3	16.9
Liberia	8.9	15.8	18.7	25.8	6.0	12.6	15.2	22.0	15.4	24.9	28.7	37.9	13.0	24.7	29.2	39.2
Madagascar	2.7	4.6	5.5	8.1	2.5	5.2	6.3	9.8	5.1	8.9	10.6	15.3	3.8	7.7	9.4	14.4
Malawi	9.3	18.0	20.8	28.0	6.4	12.8	15.3	21.9	15.1	23.9	27.2	35.6	8.9	17.2	20.1	28.0
Mali	6.5	9.4	10.5	13.9	2.6	4.5	5.4	8.3	9.3	14.7	16.3	21.2	4.7	7.9	9.4	14.2
Mauritania	18.9	31.9	37.3	47.6	9.0	18.2	22.7	33.3	31.4	47.4	53.1	63.3	14.4	28.3	34.1	46.8
Mozambique	4.9	8.2	10.0	15.1	2.8	6.1	7.7	12.7	7.6	12.8	15.5	22.6	5.2	10.8	13.8	21.8
Namibia	10.5	17.8	20.6	27.9	4.6	10.6	13.1	20.1	16.0	27.5	31.2	40.3	7.3	16.0	19.5	28.8
Niger	5.8	7.3	7.9	9.5	2.3	3.5	4.0	5.8	11.3	13.5	14.5	17.2	4.1	6.2	7.0	10.0
Nigeria	6.7	12.9	15.3	21.4	5.2	12.9	16.0	25.1	11.5	21.1	24.4	32.7	10.4	23.9	28.6	41.4
Rwanda	7.1	12.1	14.5	20.7	2.2	4.7	5.8	9.0	12.1	24.7	28.8	38.6	3.9	8.3	10.3	15.6
Sao Tome and Principe	10.5	21.1	25.1	34.8	5.5	13.4	16.5	25.0	18.0	33.1	38.3	49.5	8.1	18.9	22.7	33.2
Senegal	12.1	15.0	16.8	21.5	3.2	6.7	8.5	13.7	19.2	22.0	24.5	30.5	7.7	14.6	18.2	27.5
Sierra Leone	6.3	9.3	10.7	14.8	3.9	8.2	10.2	15.9	10.9	19.5	22.3	29.4	5.8	11.8	14.5	21.8
Somalia	7.3	11.5	13.3	18.7	2.8	5.7	7.2	12.3	13.5	23.2	25.9	34.3	4.5	9.1	11.1	18.5
South Africa	19.1	39.3	44.5	56.8	6.4	14.6	17.4	24.5	27.9	51.8	57.4	68.8	12.6	22.0	25.6	34.5
South Sudan	5.5	6.8	7.3	8.9	2.3	4.9	6.7	14.5	7.7	9.5	10.3	12.4	3.3	7.1	9.8	20.1
Togo	7.4	13.2	16.4	24.0	2.9	7.4	9.9	17.2	13.4	23.2	27.6	38.1	5.7	13.6	18.0	29.2
Uganda	8.5	16.2	19.0	27.5	2.7	6.4	7.8	12.3	11.6	21.9	25.7	35.8	5.9	13.7	16.3	24.3
Unt Republic Tanzania	5.5	11.6	14.5	22.1	3.8	8.7	10.8	16.8	10.0	22.2	27.1	38.5	7.5	15.6	19.0	28.1
Zambia	11.5	22.4	25.5	33.7	7.2	14.1	16.5	23.9	15.0	26.6	30.3	39.2	10.9	21.0	24.6	34.1
Zimbabwe	8.2	17.5	20.6	29.2	1.6	4.1	5.0	7.7	14.5	28.9	33.2	44.0	3.5	8.7	10.6	16.0

Prevalence %

Best observed — Worst observed

Figure S10: Country-level heatmaps of estimated age-standardised prevalence of obesity, 1990, 2021, 2030, 2050 by sex, super-region, and age group (5-year age bands)

5 to 9

Super-region	Country	Female 1990	Female 2021	Female 2030	Female 2050	Male 1990	Male 2021	Male 2030	Male 2050
South Asia	Bangladesh	1.0	3.5	5.1	9.8	0.8	2.2	2.9	5.2
	Bhutan	2.9	6.6	8.5	14.2	1.1	4.7	6.7	13.2
	India	1.2	4.6	6.5	11.7	1.2	4.1	5.7	10.3
	Nepal	1.8	7.7	10.6	19.0	0.3	1.8	2.8	6.8
	Pakistan	1.2	2.5	3.5	6.0	0.6	2.1	2.9	5.8
Southeast Asia, East Asia, and Oceania	American Samoa	25.4	46.4	51.5	62.7	22.6	42.3	47.3	58.4
	Cambodia	1.2	2.7	3.4	5.6	0.7	2.1	2.8	5.0
	China	0.9	5.6	8.5	18.6	1.8	9.6	13.4	25.8
	Cook Islands	35.4	56.2	61.7	71.8	34.3	56.3	61.8	72.6
	Democratic People's Republic of Korea	2.4	12.4	17.6	32.8	2.9	11.9	16.2	28.5
	Fiji	6.4	21.0	26.6	40.6	3.0	12.6	17.1	29.8
	Guam	18.8	31.8	34.8	42.2	19.4	30.0	32.3	38.5
	Indonesia	1.1	4.6	6.0	10.0	1.0	4.3	5.6	8.8
	Kiribati	6.0	20.7	27.6	44.7	6.2	22.2	29.4	47.3
	Lao People's Democratic Republic	0.8	2.2	2.8	4.7	0.4	1.7	2.4	4.8
	Malaysia	4.3	14.6	18.9	30.0	1.9	7.8	10.2	17.0
	Maldives	0.9	5.0	7.0	13.0	1.1	7.6	11.2	21.4
	Marshall Islands	15.6	31.0	36.2	46.1	10.3	26.5	31.9	43.2
	Mauritius	2.8	10.5	13.7	23.1	2.5	9.4	12.2	19.7
	Micronesia (Federated States of)	13.7	33.4	39.5	53.9	12.5	31.3	37.7	52.2
	Myanmar	1.5	3.6	4.5	7.0	1.0	2.9	3.5	5.9
	Nauru	27.6	49.2	54.9	66.8	24.3	49.9	57.0	70.8
	Niue	17.5	41.6	48.8	62.8	16.8	39.1	45.8	59.1
	Northern Mariana Islands	21.8	46.9	54.1	68.2	26.3	50.1	56.6	69.6
	Palau	22.4	45.1	51.1	63.3	24.5	42.9	48.0	58.5
	Papua New Guinea	3.1	6.6	8.0	12.7	0.7	2.4	3.3	7.0
	Philippines	1.4	4.5	6.0	11.2	1.7	4.8	6.4	10.9
	Samoa	24.0	46.1	51.7	63.7	8.9	24.7	30.0	43.1
	Seychelles	4.6	17.7	23.6	39.0	3.2	15.0	20.7	35.4
	Solomon Islands	3.6	9.7	12.3	21.2	1.7	6.6	9.1	18.3
	Sri Lanka	2.3	8.4	10.4	16.7	1.1	4.0	5.2	9.4
	Taiwan (Province of China)	1.8	15.1	22.0	40.7	3.5	23.6	32.5	51.8
	Thailand	2.4	10.2	14.0	24.1	1.8	8.0	10.7	18.2
	Timor-Leste	1.3	4.4	6.2	11.8	1.2	6.7	9.9	20.2
	Tokelau	12.9	34.3	40.9	54.4	17.8	43.2	49.8	62.6
	Tonga	23.4	48.3	55.3	69.4	24.2	50.6	58.2	72.0
	Tuvalu	10.3	32.1	41.1	58.9	12.5	33.9	42.2	58.6
	Vanuatu	4.3	12.0	15.4	25.7	2.4	7.8	10.4	19.3
	Viet Nam	0.3	1.4	2.0	3.9	0.4	1.5	2.1	3.7

10.0 50.0

10 to 14

Super-region	Country	Female 1990	Female 2021	Female 2030	Female 2050	Male 1990	Male 2021	Male 2030	Male 2050
South Asia	Bangladesh	0.6	2.2	3.4	7.3	0.6	2.0	2.7	5.0
	Bhutan	1.3	3.4	4.7	8.4	0.7	3.4	5.1	10.6
	India	0.4	2.1	3.2	6.2	0.7	2.9	4.1	7.7
	Nepal	0.7	2.1	3.1	6.4	0.3	1.7	2.7	6.6
	Pakistan	0.8	2.4	3.3	6.0	0.7	2.8	4.0	7.9
Southeast Asia, East Asia, and Oceania	American Samoa	19.7	38.8	43.7	55.3	19.5	38.0	43.4	54.8
	Cambodia	0.5	1.2	1.5	2.7	0.4	1.3	1.8	3.4
	China	0.6	4.1	6.3	14.7	1.4	8.2	11.6	23.3
	Cook Islands	28.5	48.0	53.3	64.1	31.6	53.7	59.5	70.6
	Democratic People's Republic of Korea	0.9	5.9	9.0	20.1	1.5	7.5	10.5	20.6
	Fiji	4.4	15.4	19.9	32.3	2.3	10.0	13.9	25.3
	Guam	14.4	25.4	28.2	35.2	15.3	24.4	26.6	32.4
	Indonesia	0.6	2.5	3.4	6.1	0.6	2.5	3.4	5.7
	Kiribati	3.9	14.9	19.9	34.7	4.8	18.0	24.2	40.6
	Lao People's Democratic Republic	0.6	1.6	2.1	3.6	0.3	1.2	1.7	3.6
	Malaysia	3.6	13.3	17.3	28.2	1.5	6.3	8.2	14.3
	Maldives	0.6	3.8	5.7	11.7	0.6	5.3	8.1	17.1
	Marshall Islands	11.1	24.0	28.6	37.8	8.7	23.4	28.7	39.7
	Mauritius	2.3	9.1	12.0	20.8	2.1	8.3	11.0	18.4
	Micronesia (Federated States of)	11.1	28.5	34.8	49.0	11.4	28.8	35.1	49.5
	Myanmar	0.6	1.5	1.9	3.1	0.8	2.3	2.9	5.0
	Nauru	22.9	43.2	49.0	61.5	23.7	49.3	56.2	69.9
	Niue	12.2	32.4	39.2	53.3	13.4	33.2	39.7	53.0
	Northern Mariana Islands	15.6	37.8	44.9	60.0	21.4	44.1	50.8	64.8
	Palau	16.1	36.3	42.3	55.0	20.3	37.6	42.7	53.5
	Papua New Guinea	1.5	3.2	3.9	6.6	0.8	2.6	3.7	7.6
	Philippines	0.8	3.1	4.2	8.5	1.5	4.4	5.8	10.4
	Samoa	18.6	37.7	43.4	55.5	6.9	20.1	24.8	36.5
	Seychelles	3.6	14.9	20.6	36.0	2.4	12.1	17.3	31.7
	Solomon Islands	2.5	7.0	9.1	16.3	1.4	5.4	7.6	15.7
	Sri Lanka	1.2	4.5	5.7	9.9	0.7	2.6	3.4	6.4
	Taiwan (Province of China)	1.2	11.4	17.5	35.1	2.7	20.2	28.3	47.6
	Thailand	2.0	8.7	12.0	21.4	1.8	8.1	11.1	19.1
	Timor-Leste	0.4	1.6	2.3	5.2	0.6	3.8	6.0	13.9
	Tokelau	8.8	26.1	31.8	44.9	14.5	37.7	44.5	57.4
	Tonga	17.6	39.7	47.2	62.0	20.9	46.4	54.0	68.1
	Tuvalu	7.9	27.2	35.3	52.9	11.1	31.9	40.0	56.2
	Vanuatu	2.6	8.0	10.7	19.1	2.0	6.9	9.6	18.3
	Viet Nam	0.2	1.0	1.4	2.8	0.3	1.0	1.4	2.6

10.0 50.0

		15 to 19							
		Female				Male			
		1990	2021	2030	2050	1990	2021	2030	2050
South Asia	Bangladesh	0.3	1.5	2.4	5.3	0.3	1.0	1.5	2.9
	Bhutan	1.0	2.6	3.5	6.5	0.4	1.9	3.0	6.7
	India	0.3	1.9	3.0	5.6	0.3	1.7	2.5	5.0
	Nepal	0.4	1.1	1.7	3.7	0.2	1.3	2.1	5.2
	Pakistan	0.7	2.5	3.4	6.1	0.5	2.2	3.1	6.4
Southeast Asia, East Asia, and Oceania	American Samoa	18.0	36.4	41.6	53.4	13.8	29.3	34.0	45.5
	Cambodia	0.2	0.6	0.8	1.5	0.2	0.6	0.8	1.6
	China	0.5	3.8	6.0	14.0	0.8	5.8	8.4	18.0
	Cook Islands	26.5	46.5	51.8	63.0	23.6	44.8	50.7	63.1
	Democratic People's Republic of Korea	0.6	4.5	7.0	16.4	0.9	4.8	7.1	14.8
	Fiji	3.8	13.9	18.2	30.4	1.3	6.2	8.9	17.6
	Guam	12.2	24.6	27.4	34.4	10.1	18.1	20.0	25.1
	Indonesia	0.5	2.7	3.6	6.6	0.4	1.7	2.3	3.9
	Kiribati	3.3	12.7	17.6	32.1	2.9	12.7	17.7	32.4
	Lao People's Democratic Republic	0.5	1.5	2.0	3.4	0.1	0.6	0.9	2.0
	Malaysia	3.6	15.2	19.6	31.3	0.9	4.5	6.0	10.8
	Maldives	0.6	4.5	6.6	13.4	0.3	2.6	4.4	10.4
	Marshall Islands	9.8	21.8	26.2	35.3	5.4	16.4	20.5	30.3
	Mauritius	2.1	8.2	10.9	19.3	1.3	5.6	7.6	13.5
	Micronesia (Federated States of)	9.7	26.4	32.5	46.7	7.3	21.2	26.6	40.4
	Myanmar	0.4	1.1	1.4	2.4	0.5	1.6	2.1	3.7
	Nauru	21.4	41.3	47.4	60.1	17.3	40.8	48.4	63.3
	Niue	10.8	29.9	37.0	51.4	9.0	25.0	30.7	43.9
	Northern Mariana Islands	13.9	35.4	42.7	58.1	15.0	35.0	41.7	56.5
	Palau	14.3	33.5	39.6	52.6	14.3	28.7	33.4	44.2
	Papua New Guinea	1.2	2.6	3.3	5.5	0.5	2.0	2.9	6.2
	Philippines	0.7	2.8	4.0	7.9	0.8	2.4	3.3	6.3
	Samoa	17.5	37.2	42.8	55.1	4.3	13.5	17.1	27.2
	Seychelles	3.1	13.4	18.8	33.7	1.4	8.2	12.0	24.0
	Solomon Islands	2.1	6.1	7.9	14.7	0.8	3.4	4.8	10.7
	Sri Lanka	1.1	3.7	4.8	8.5	0.4	1.4	2.0	4.0
	Taiwan (Province of China)	1.1	10.9	16.6	33.8	2.1	15.1	22.2	40.7
	Thailand	2.1	7.9	11.1	20.2	1.3	5.5	7.6	13.9
	Timor-Leste	0.2	0.6	1.0	2.3	0.1	1.0	1.7	4.9
	Tokelau	7.7	24.0	29.3	42.5	9.5	28.7	35.0	48.3
	Tonga	15.8	37.7	45.1	60.3	15.0	36.9	44.3	59.7
	Tuvalu	7.0	25.4	33.5	51.2	7.3	23.7	30.7	46.9
	Vanuatu	2.0	6.4	8.6	16.0	1.1	4.5	6.4	13.1
	Viet Nam	0.2	0.7	1.0	2.1	0.3	1.1	1.6	2.9

10.0 – 50.0

		20 to 24							
		Female				Male			
		1990	2021	2030	2050	1990	2021	2030	2050
South Asia	Bangladesh	0.8	4.6	6.7	13.3	0.5	2.2	3.1	5.9
	Bhutan	2.5	6.5	8.6	14.3	0.8	4.2	6.1	12.6
	India	0.5	2.2	3.3	6.4	0.5	2.7	3.8	7.3
	Nepal	0.8	2.4	3.5	7.3	0.5	2.3	3.7	8.4
	Pakistan	2.1	6.5	8.6	14.4	0.9	3.8	5.2	10.1
Southeast Asia, East Asia, and Oceania	American Samoa	34.4	55.7	60.5	70.3	25.3	44.9	49.8	60.7
	Cambodia	0.5	1.2	1.5	2.7	0.2	0.8	1.1	2.2
	China	0.8	5.4	8.5	18.4	1.1	7.0	10.3	21.0
	Cook Islands	44.3	66.0	70.5	78.8	35.3	57.5	62.7	73.0
	Democratic People's Republic of Korea	0.8	5.2	7.8	18.0	0.8	4.3	6.2	13.5
	Fiji	8.7	25.5	31.5	45.9	2.5	11.5	15.7	27.5
	Guam	20.8	33.5	36.6	43.9	18.2	26.7	29.1	35.1
	Indonesia	1.0	5.5	7.3	12.4	0.5	2.5	3.3	5.8
	Kiribati	16.3	42.5	50.1	66.0	8.2	28.2	35.9	53.7
	Lao People's Democratic Republic	1.1	3.2	4.1	6.8	0.3	1.1	1.6	3.4
	Malaysia	5.7	19.3	24.4	37.0	1.4	4.7	6.1	10.8
	Maldives	1.6	9.0	12.9	23.1	0.5	4.9	7.8	16.8
	Marshall Islands	16.6	31.6	36.7	46.6	9.6	23.7	29.0	40.1
	Mauritius	4.5	14.5	18.9	30.0	2.3	9.1	12.0	20.0
	Micronesia (Federated States of)	20.8	43.4	49.8	63.4	13.9	33.5	39.7	53.8
	Myanmar	0.8	2.0	2.5	4.2	0.5	1.6	2.0	3.6
	Nauru	39.4	58.7	64.1	74.4	33.7	57.7	63.9	75.7
	Niue	20.3	45.7	53.0	66.0	15.6	36.7	43.1	56.2
	Northern Mariana Islands	25.0	50.8	57.3	70.7	24.3	47.6	54.2	67.6
	Palau	24.0	46.9	53.0	64.9	22.2	39.9	44.6	55.2
	Papua New Guinea	2.3	5.0	6.2	9.9	1.0	3.7	5.0	10.1
	Philippines	1.6	5.1	6.9	13.1	1.3	4.5	6.1	10.9
	Samoa	30.7	55.1	60.3	70.8	11.9	29.9	35.2	47.6
	Seychelles	7.1	24.3	31.9	49.1	2.4	12.5	17.7	32.7
	Solomon Islands	5.2	12.4	15.5	25.6	1.4	5.3	7.3	14.9
	Sri Lanka	2.4	6.7	8.5	14.1	0.7	2.7	3.6	6.8
	Taiwan (Province of China)	1.6	13.5	20.0	38.3	1.6	14.6	21.7	40.0
	Thailand	2.7	12.3	16.5	27.7	1.3	6.2	8.6	15.4
	Timor-Leste	0.3	1.2	1.8	4.1	0.1	1.1	1.9	5.3
	Tokelau	15.6	38.7	45.1	58.1	16.9	41.2	47.7	60.2
	Tonga	31.6	61.6	67.8	79.0	19.5	45.0	52.3	66.5
	Tuvalu	17.8	46.0	54.8	70.3	15.0	38.9	47.2	62.7
	Vanuatu	4.9	13.3	16.9	27.8	1.8	6.4	8.9	17.3
	Viet Nam	0.2	1.0	1.5	3.0	0.2	1.0	1.4	2.6

10.0 – 50.0

		5 to 9							
		Female				Male			
		1990	2021	2030	2050	1990	2021	2030	2050
Central Europe, Eastern Europe, and Central Asia	Albania	3.1	8.6	10.1	13.3	5.1	10.0	10.9	12.6
	Armenia	4.2	7.9	9.0	11.7	6.0	10.5	11.7	14.7
	Azerbaijan	4.4	10.0	11.6	16.1	6.0	10.5	12.1	15.5
	Belarus	2.4	7.6	9.3	14.6	1.7	6.2	7.8	12.7
	Bosnia and Herzegovina	6.2	17.6	21.0	29.0	6.2	14.0	15.8	20.2
	Bulgaria	5.0	9.1	10.4	13.3	6.9	11.8	13.0	15.6
	Croatia	3.5	10.8	13.6	19.9	7.2	15.7	18.5	24.0
	Czechia	4.1	7.6	8.7	11.7	6.3	9.4	10.4	13.0
	Estonia	7.3	12.9	14.7	19.1	6.7	12.8	15.0	20.3
	Georgia	2.6	4.2	4.7	5.8	3.9	6.3	6.9	8.4
	Hungary	6.4	13.1	15.5	21.1	6.6	12.9	14.8	19.7
	Kazakhstan	2.7	6.8	7.9	11.3	3.0	6.6	7.5	9.9
	Kyrgyzstan	2.3	5.9	7.2	10.8	2.0	4.8	5.8	8.5
	Latvia	3.9	7.6	8.7	11.5	3.7	8.0	9.4	13.0
	Lithuania	1.3	4.2	5.2	7.3	2.7	8.3	9.9	13.3
	Mongolia	3.3	7.1	8.2	10.9	3.4	4.7	5.0	5.6
	Montenegro	5.6	16.6	20.8	31.2	13.0	25.5	28.8	36.3
	North Macedonia	3.7	10.4	13.0	19.5	6.1	11.3	12.8	16.7
	Poland	2.7	6.9	8.3	11.7	3.9	9.3	10.8	14.5
	Republic of Moldova	1.8	5.5	6.8	10.8	1.6	5.0	6.4	10.5
	Romania	3.0	8.5	10.3	14.4	4.8	11.5	13.1	17.0
	Russian Federation	6.8	11.4	13.1	17.5	5.8	12.4	14.8	21.2
	Serbia	3.0	10.6	13.6	21.4	2.8	7.9	9.8	15.0
	Slovakia	2.7	5.4	6.4	9.1	5.1	8.8	10.0	13.1
	Slovenia	2.6	6.8	8.5	13.1	7.2	13.3	15.6	20.7
	Tajikistan	3.7	7.7	9.2	12.9	3.4	6.8	8.0	11.2
	Turkmenistan	2.4	4.4	5.0	6.5	4.0	5.6	6.0	6.9
	Ukraine	2.3	6.0	7.0	10.4	2.5	6.5	7.5	11.0
	Uzbekistan	4.0	9.1	10.9	14.9	3.5	7.9	9.3	12.7

10.0 50.0

		10 to 14							
		Female				Male			
		1990	2021	2030	2050	1990	2021	2030	2050
Central Europe, Eastern Europe, and Central Asia	Albania	1.2	3.9	4.7	6.5	2.5	5.1	5.6	6.6
	Armenia	1.7	3.5	4.0	5.4	2.9	5.5	6.3	8.2
	Azerbaijan	1.9	4.6	5.5	8.2	2.8	5.7	6.6	8.8
	Belarus	1.4	4.9	6.1	10.1	1.5	5.7	7.3	12.1
	Bosnia and Herzegovina	1.4	5.3	6.6	10.6	3.8	9.3	10.8	14.4
	Bulgaria	3.2	6.2	7.1	9.4	5.6	9.6	10.8	13.1
	Croatia	2.5	8.2	10.5	16.0	6.2	13.8	16.5	21.8
	Czechia	2.6	5.2	6.0	8.2	4.5	6.9	7.8	9.9
	Estonia	5.5	10.3	11.9	15.8	5.7	11.2	13.2	18.2
	Georgia	3.3	6.2	6.9	8.4	6.2	11.2	12.0	14.2
	Hungary	5.0	10.7	12.8	18.0	5.5	11.0	12.8	17.3
	Kazakhstan	1.4	3.8	4.5	6.7	2.3	5.2	5.9	7.9
	Kyrgyzstan	1.0	2.7	3.4	5.4	1.7	4.0	4.8	7.2
	Latvia	2.8	5.9	6.8	9.1	2.9	6.6	7.9	11.1
	Lithuania	0.8	2.8	3.5	5.1	2.0	6.6	7.9	11.0
	Mongolia	1.6	3.6	4.1	5.7	2.7	4.0	4.2	4.8
	Montenegro	2.7	10.0	13.1	21.4	9.9	19.8	22.6	29.6
	North Macedonia	1.1	3.7	4.8	8.0	5.2	10.0	11.4	15.2
	Poland	1.8	4.8	5.8	8.4	3.1	7.5	8.9	12.2
	Republic of Moldova	1.8	5.7	7.3	11.6	1.9	6.2	7.9	12.8
	Romania	1.9	6.0	7.3	10.6	4.3	10.6	12.4	16.3
	Russian Federation	1.8	5.3	6.1	8.8	2.1	6.6	8.1	12.5
	Serbia	1.9	8.1	10.5	17.2	2.0	7.0	8.8	13.7
	Slovakia	1.7	3.6	4.4	6.4	3.8	6.9	8.0	10.7
	Slovenia	1.7	4.8	6.1	9.6	5.4	10.8	12.7	17.3
	Tajikistan	1.9	4.3	5.2	7.6	2.7	5.8	6.9	9.8
	Turkmenistan	1.5	2.9	3.3	4.4	3.3	4.7	5.0	5.7
	Ukraine	1.4	4.0	4.7	7.2	1.7	5.0	5.8	8.6
	Uzbekistan	1.3	3.6	4.4	6.4	1.9	4.8	5.7	8.0

10.0 50.0

Central Europe, Eastern Europe, and Central Asia	15 to 19							
	Female				Male			
	1990	2021	2030	2050	1990	2021	2030	2050
Albania	0.8	2.8	3.4	4.8	1.1	2.2	2.5	3.0
Armenia	1.2	2.4	2.7	3.7	1.1	2.3	2.7	3.6
Azerbaijan	1.4	3.3	4.1	6.2	1.3	3.3	3.8	5.2
Belarus	1.1	3.9	5.0	8.4	0.9	3.6	4.7	8.2
Bosnia and Herzegovina	0.6	2.4	3.1	5.3	1.7	4.6	5.4	7.5
Bulgaria	2.7	5.4	6.4	8.4	3.3	6.2	6.9	8.6
Croatia	2.1	7.0	9.0	13.8	3.7	8.9	10.6	14.6
Czechia	2.7	4.6	5.5	7.6	2.7	4.2	4.8	6.3
Estonia	5.0	9.1	10.6	14.2	3.5	7.3	8.8	12.6
Georgia	3.3	6.9	7.7	9.4	4.9	10.1	11.0	13.1
Hungary	4.5	10.2	12.0	16.9	3.4	7.5	9.0	12.6
Kazakhstan	1.0	2.7	3.3	4.9	1.3	3.0	3.6	4.9
Kyrgyzstan	0.6	1.5	1.9	3.2	1.3	3.2	3.9	5.9
Latvia	2.6	5.6	6.5	8.7	1.7	4.5	5.4	7.7
Lithuania	0.6	2.4	3.0	4.4	1.4	3.9	4.8	6.9
Mongolia	1.1	2.6	3.1	4.3	1.6	2.5	2.7	3.1
Montenegro	1.3	5.4	7.3	13.0	5.3	11.6	13.5	18.8
North Macedonia	0.8	3.0	3.9	6.7	3.2	6.5	7.7	10.5
Poland	1.7	4.9	5.9	8.6	1.7	5.2	6.1	8.5
Republic of Moldova	1.7	5.4	6.9	11.0	1.1	4.0	5.1	8.7
Romania	1.6	5.3	6.4	9.3	2.7	7.4	8.7	11.7
Russian Federation	1.5	4.6	5.5	7.9	1.0	4.0	4.9	8.0
Serbia	1.6	7.6	10.0	16.5	1.0	3.2	4.1	7.0
Slovakia	1.5	3.0	3.6	5.3	2.1	4.2	4.8	6.7
Slovenia	1.3	4.0	5.1	8.3	3.1	6.4	7.7	11.0
Tajikistan	1.2	2.9	3.5	5.2	1.4	3.1	3.8	5.6
Turkmenistan	1.2	2.4	2.7	3.7	1.9	2.8	3.0	3.5
Ukraine	1.1	3.8	4.4	6.8	0.9	2.9	3.4	5.3
Uzbekistan	0.9	3.2	3.9	5.8	1.0	3.0	3.6	5.2

10.0 50.0

Central Europe, Eastern Europe, and Central Asia	20 to 24							
	Female				Male			
	1990	2021	2030	2050	1990	2021	2030	2050
Albania	1.8	5.2	6.3	8.5	1.6	3.2	3.5	4.1
Armenia	2.7	5.4	6.1	8.0	1.9	4.5	5.1	6.6
Azerbaijan	3.1	7.9	9.4	13.4	1.0	2.1	2.5	3.4
Belarus	2.5	7.4	9.2	14.6	1.8	6.6	8.3	13.5
Bosnia and Herzegovina	2.3	7.6	9.5	14.6	2.9	7.5	8.6	11.6
Bulgaria	5.1	9.1	10.4	13.3	5.4	9.4	10.4	12.7
Croatia	4.5	13.1	16.4	23.4	6.5	14.4	17.1	22.5
Czechia	4.7	8.0	9.2	12.2	3.3	5.0	5.6	7.1
Estonia	8.1	15.4	17.6	22.6	6.3	14.5	16.9	22.8
Georgia	5.4	11.3	12.3	14.7	6.3	12.4	13.5	15.9
Hungary	7.7	15.4	18.1	24.3	7.6	14.5	16.8	22.2
Kazakhstan	2.5	6.4	7.5	10.7	2.6	5.5	6.3	8.3
Kyrgyzstan	1.6	3.8	4.7	7.2	2.4	5.7	6.8	9.9
Latvia	4.9	10.5	11.9	15.3	3.2	8.6	10.0	13.5
Lithuania	2.8	9.3	11.2	15.0	2.7	8.1	9.7	13.4
Mongolia	2.8	7.4	8.6	11.4	4.0	6.5	7.0	7.8
Montenegro	3.3	11.3	14.4	23.1	9.0	17.8	20.7	27.4
North Macedonia	1.7	5.3	6.7	10.9	5.3	10.1	11.6	15.4
Poland	2.9	7.2	8.7	12.3	2.9	6.5	7.8	10.7
Republic of Moldova	3.9	10.9	13.3	19.8	2.5	7.8	9.7	15.1
Romania	4.8	12.3	14.3	19.2	6.7	15.8	18.1	22.8
Russian Federation	4.9	11.1	12.8	17.2	2.1	7.6	9.3	14.2
Serbia	3.6	13.0	16.4	25.0	1.6	5.2	6.5	10.3
Slovakia	3.0	6.0	7.0	10.0	5.5	10.2	11.6	15.2
Slovenia	3.4	8.4	10.5	15.9	7.3	13.5	15.7	21.0
Tajikistan	2.7	5.6	6.7	9.7	3.5	7.9	9.4	12.9
Turkmenistan	2.6	5.2	5.8	7.6	3.3	4.8	5.1	5.7
Ukraine	3.8	9.9	11.2	15.9	3.0	7.3	8.4	12.1
Uzbekistan	2.2	7.2	8.6	12.1	1.7	5.3	6.3	8.7

10.0 50.0

		5 to 9							
		Female				Male			
		1990	2021	2030	2050	1990	2021	2030	2050
High-income	Andorra	5.9	11.9	13.9	18.9	6.5	11.3	13.2	17.5
	Argentina	3.1	9.0	11.6	19.3	3.1	10.5	13.6	22.4
	Australia	7.6	19.1	22.7	32.0	4.8	15.4	19.2	28.8
	Austria	4.2	8.4	9.8	13.3	5.0	8.8	9.9	12.8
	Belgium	3.5	10.3	12.7	18.6	2.4	7.2	8.9	13.2
	Brunei Darussalam	0.3	1.8	2.8	7.2	0.5	3.3	5.0	11.4
	Canada	9.7	16.5	18.5	23.1	9.6	18.1	20.1	25.5
	Chile	6.7	18.9	22.4	32.5	6.3	22.5	27.1	39.1
	Cyprus	2.6	10.5	13.8	22.7	3.3	12.8	16.1	25.0
	Denmark	6.7	13.7	16.2	21.7	6.5	12.6	14.5	19.2
	Finland	5.8	12.8	15.1	20.4	5.0	12.3	14.6	20.4
	France	2.8	8.7	11.1	17.5	3.3	11.2	14.3	21.9
	Germany	7.7	13.2	14.9	18.8	9.4	14.6	15.7	18.7
	Greece	2.8	10.0	13.4	22.1	3.7	13.4	17.4	26.9
	Greenland	12.3	17.8	19.2	22.8	11.7	17.5	19.5	23.9
	Iceland	7.0	15.1	17.1	22.0	8.3	13.7	15.1	18.1
	Ireland	4.9	12.9	14.9	20.2	4.4	14.5	16.8	23.4
	Israel	4.7	9.7	11.7	16.8	4.5	8.0	9.3	12.6
	Italy	2.3	7.5	9.8	15.9	2.3	7.9	10.3	16.7
	Japan	2.7	6.3	7.8	11.6	3.2	7.4	9.3	13.9
	Luxembourg	3.9	9.9	11.7	16.5	5.0	11.7	13.9	19.0
	Malta	2.3	8.7	11.2	18.0	1.7	6.8	9.0	15.1
	Monaco	7.7	15.7	18.0	24.1	9.1	15.8	18.0	23.1
	Netherlands	4.8	12.2	14.9	21.1	3.2	7.5	8.9	12.3
	New Zealand	10.4	17.4	20.3	26.8	10.6	16.9	19.4	25.3
	Norway	5.1	11.2	13.0	17.6	6.3	11.8	13.2	16.9
	Portugal	2.4	9.8	13.1	21.6	1.9	9.0	12.1	20.1
	Republic of Korea	0.7	5.4	7.4	13.3	1.5	8.5	11.1	18.0
	San Marino	6.4	13.8	16.8	24.2	7.1	14.5	17.3	24.4
	Singapore	0.8	6.6	9.6	20.1	0.7	7.4	10.8	22.2
	Spain	2.9	10.4	13.7	22.5	3.3	12.4	15.9	24.6
	Sweden	5.8	10.8	12.6	17.3	5.8	11.2	13.1	18.0
	Switzerland	5.7	9.4	10.5	13.7	7.0	9.9	11.0	13.7
	United Kingdom	5.7	12.6	14.5	19.3	4.3	9.8	11.3	15.3
	United States of America	10.5	15.2	17.3	22.3	10.3	13.9	15.7	20.0
	Uruguay	7.7	15.4	17.9	23.3	3.8	10.5	12.7	18.5

10.0 50.0

		10 to 14							
		Female				Male			
		1990	2021	2030	2050	1990	2021	2030	2050
High-income	Andorra	4.3	9.2	11.0	15.2	6.1	10.7	12.4	16.7
	Argentina	2.1	6.4	8.5	14.6	2.4	8.3	10.9	18.8
	Australia	5.7	15.4	18.8	27.5	4.2	13.5	17.0	26.0
	Austria	2.8	6.0	7.0	9.8	4.2	7.7	8.8	11.5
	Belgium	3.2	9.8	11.8	17.7	3.0	9.3	11.3	16.8
	Brunei Darussalam	0.2	1.7	2.7	7.0	0.7	4.6	6.9	15.1
	Canada	7.8	13.8	15.6	19.9	8.5	16.3	18.2	23.4
	Chile	4.8	14.6	18.2	27.4	5.1	19.1	23.3	34.7
	Cyprus	1.8	8.2	10.7	18.5	3.2	12.7	16.1	25.3
	Denmark	4.8	10.4	12.2	16.9	5.2	10.6	12.4	16.7
	Finland	4.1	10.0	11.9	16.6	4.1	10.4	12.6	17.9
	France	1.8	6.3	8.1	13.2	2.8	9.9	12.7	20.2
	Germany	5.5	10.0	11.3	14.5	7.5	12.1	13.1	15.8
	Greece	2.0	7.4	10.4	18.0	3.4	12.3	15.8	24.9
	Greenland	11.5	17.1	18.4	22.0	12.2	18.6	20.7	25.3
	Iceland	5.1	11.5	13.2	17.4	6.7	11.5	12.7	15.5
	Ireland	3.5	10.0	11.7	16.2	3.8	13.2	15.6	21.9
	Israel	3.4	7.3	9.0	13.2	4.1	7.7	9.1	12.4
	Italy	1.6	5.6	7.4	12.5	1.9	6.7	8.9	14.7
	Japan	1.4	3.6	4.6	7.1	2.6	6.7	8.4	12.8
	Luxembourg	3.0	7.9	9.5	13.8	4.6	11.1	13.1	18.0
	Malta	1.9	7.4	9.7	15.8	1.6	6.9	9.2	15.4
	Monaco	5.8	12.3	14.5	19.9	8.7	15.1	17.0	22.0
	Netherlands	3.5	9.4	11.7	17.1	2.5	6.1	7.3	10.3
	New Zealand	9.0	15.8	18.3	24.4	10.2	16.7	19.1	24.9
	Norway	3.5	7.8	9.1	12.8	4.9	9.6	10.8	14.0
	Portugal	2.0	8.5	11.4	19.2	2.1	9.6	13.1	21.8
	Republic of Korea	0.5	3.6	5.0	9.5	1.2	7.1	9.6	16.3
	San Marino	4.9	10.8	13.4	19.9	6.6	13.7	16.3	23.3
	Singapore	0.6	6.0	8.8	19.1	0.8	7.6	11.1	23.3
	Spain	2.2	8.3	11.2	19.1	2.8	11.1	14.1	22.2
	Sweden	4.0	8.1	9.7	13.7	4.6	9.3	11.1	15.6
	Switzerland	3.4	5.8	6.7	9.0	4.5	6.4	7.3	9.3
	United Kingdom	4.8	11.3	13.0	17.5	4.3	10.0	11.6	15.7
	United States of America	6.9	16.3	18.2	23.1	7.7	18.1	20.2	25.3
	Uruguay	5.7	12.0	13.9	18.4	3.1	9.1	11.1	16.7

10.0 50.0

		15 to 19							
		Female				Male			
		1990	2021	2030	2050	1990	2021	2030	2050
High-income	Andorra	3.7	8.0	9.7	13.6	4.1	7.5	8.7	12.1
	Argentina	1.8	6.9	8.9	15.4	1.5	6.2	8.2	14.7
	Australia	4.8	13.4	16.5	24.6	2.9	9.2	11.9	19.3
	Austria	2.3	4.9	5.7	8.1	2.6	4.9	5.7	7.6
	Belgium	3.2	9.2	11.4	17.1	2.0	6.9	8.5	13.0
	Brunei Darussalam	0.3	2.3	3.5	8.6	0.6	4.0	5.9	13.4
	Canada	6.6	13.6	15.2	19.3	4.9	11.2	12.8	17.0
	Chile	3.8	12.6	15.5	24.1	3.2	13.6	17.0	27.1
	Cyprus	1.8	7.6	10.0	17.5	2.4	10.0	12.9	21.0
	Denmark	3.8	9.9	11.8	16.4	3.0	7.3	8.7	12.1
	Finland	3.6	8.8	10.5	14.8	2.5	6.6	8.1	12.0
	France	1.5	5.8	7.6	12.5	1.9	7.2	9.4	15.6
	Germany	4.8	9.3	10.6	13.6	5.2	8.5	9.4	11.6
	Greece	2.0	6.5	8.9	15.7	2.9	9.8	13.1	21.2
	Greenland	10.5	15.8	17.3	20.7	7.8	12.9	14.3	18.1
	Iceland	4.4	10.5	12.1	16.2	3.6	7.6	8.4	10.5
	Ireland	3.9	9.7	11.3	15.8	3.1	9.7	11.8	17.1
	Israel	2.9	6.5	7.9	11.8	2.8	5.3	6.3	8.8
	Italy	1.9	3.8	5.2	9.1	1.6	3.7	5.0	8.8
	Japan	0.9	2.3	3.0	4.8	1.8	4.0	5.2	8.3
	Luxembourg	2.6	6.8	8.2	12.1	3.0	7.6	9.0	12.8
	Malta	2.1	7.5	9.7	15.8	1.1	5.0	6.7	11.7
	Monaco	5.0	10.8	12.6	17.5	5.7	10.7	12.4	16.5
	Netherlands	3.4	8.8	11.0	16.2	1.5	4.2	5.2	7.6
	New Zealand	7.8	17.0	19.7	26.1	6.6	14.0	16.3	21.7
	Norway	3.2	6.9	8.2	11.6	3.2	5.6	6.6	8.9
	Portugal	2.1	7.7	10.6	18.1	1.3	6.7	9.2	16.1
	Republic of Korea	0.3	1.8	2.7	5.6	0.6	4.0	5.5	10.0
	San Marino	4.0	9.4	11.6	17.6	4.3	9.7	11.8	17.5
	Singapore	0.6	5.3	7.9	17.0	0.5	5.0	7.7	17.6
	Spain	2.4	7.9	10.6	18.1	3.1	7.7	10.0	16.6
	Sweden	3.6	7.2	8.6	12.3	3.0	5.4	6.5	9.6
	Switzerland	2.7	5.7	6.6	8.8	2.6	4.7	5.4	6.9
	United Kingdom	5.2	11.9	13.8	18.4	4.1	9.5	11.0	15.0
	United States of America	8.3	24.6	27.3	33.5	7.7	18.6	20.8	26.0
	Uruguay	5.5	11.1	13.0	17.4	1.9	6.8	8.6	13.2

10.0 50.0

		20 to 24							
		Female				Male			
		1990	2021	2030	2050	1990	2021	2030	2050
High-income	Andorra	5.9	12.0	14.1	19.2	5.8	9.9	11.7	15.8
	Argentina	6.4	20.7	25.2	36.4	4.5	16.9	21.2	32.4
	Australia	8.5	23.8	28.4	38.7	5.8	17.4	21.6	31.6
	Austria	4.3	8.4	9.7	13.2	4.5	8.0	9.1	11.9
	Belgium	5.9	15.1	18.1	25.5	3.3	9.6	11.7	17.4
	Brunei Darussalam	1.6	12.0	16.8	30.6	2.1	12.8	17.6	31.4
	Canada	8.7	18.9	21.1	26.1	6.7	16.2	18.1	23.3
	Chile	8.2	26.4	30.9	41.9	5.2	19.6	24.0	35.1
	Cyprus	3.1	12.4	16.1	25.8	2.4	10.2	13.1	21.3
	Denmark	5.3	14.7	17.3	23.1	4.1	10.6	12.4	16.8
	Finland	5.4	14.2	16.6	22.4	3.6	9.8	11.8	17.0
	France	3.6	9.7	12.3	19.1	3.3	10.7	13.8	22.0
	Germany	7.1	13.6	15.0	18.9	6.7	10.6	11.6	14.1
	Greece	4.5	12.9	16.9	26.8	4.1	13.0	16.7	26.0
	Greenland	16.9	23.6	25.6	29.9	11.1	17.3	19.0	23.4
	Iceland	7.4	18.2	20.7	26.2	5.8	11.7	12.8	15.6
	Ireland	6.1	16.1	18.2	24.2	5.0	14.7	17.1	23.7
	Israel	5.8	11.1	13.2	18.7	4.1	7.4	8.6	11.7
	Italy	3.3	8.0	10.5	17.0	2.1	6.1	8.1	13.4
	Japan	1.0	2.5	3.1	5.0	1.4	4.7	6.0	9.6
	Luxembourg	4.3	11.2	13.1	18.4	4.0	9.9	11.6	16.3
	Malta	3.7	12.7	16.0	24.3	2.0	8.2	10.8	17.8
	Monaco	9.0	17.7	20.5	27.0	8.6	15.2	17.3	22.3
	Netherlands	5.7	18.1	21.5	29.2	2.7	6.4	7.7	11.0
	New Zealand	14.4	28.8	32.3	40.0	11.3	24.1	27.0	33.7
	Norway	6.1	11.3	13.1	17.8	5.8	9.1	10.1	13.2
	Portugal	3.6	11.8	15.7	25.1	2.3	10.2	13.7	22.7
	Republic of Korea	0.3	2.1	3.1	6.2	0.6	3.4	4.8	8.9
	San Marino	7.3	15.9	19.0	26.9	6.8	13.8	16.5	23.6
	Singapore	1.2	9.2	13.3	26.1	1.2	9.9	14.5	28.7
	Spain	4.4	13.9	18.1	28.3	4.8	11.6	14.6	22.9
	Sweden	5.0	12.4	14.5	19.6	3.1	8.9	10.6	15.0
	Switzerland	4.6	10.1	11.4	14.8	3.7	6.4	7.1	9.0
	United Kingdom	8.5	19.1	21.5	27.5	6.7	14.7	16.8	21.9
	United States of America	12.0	33.3	36.3	42.8	10.0	27.1	29.6	35.6
	Uruguay	5.9	14.7	16.8	22.0	3.4	10.3	12.7	18.8

10.0 50.0

		5 to 9							
		Female				Male			
		1990	2021	2030	2050	1990	2021	2030	2050
Latin America and Caribbean	Antigua and Barbuda	6.2	17.2	21.6	33.6	3.8	12.4	16.1	27.4
	Bahamas	8.8	22.6	27.8	40.3	6.0	17.9	22.9	36.1
	Barbados	6.4	18.3	22.8	34.2	4.6	14.6	18.8	29.0
	Belize	6.2	15.3	18.1	25.7	2.9	9.7	12.2	20.0
	Bermuda	8.2	22.7	27.5	40.1	7.1	22.5	28.3	42.4
	Bolivia (Plurinational State of)	3.9	12.4	16.0	25.6	2.9	10.9	14.1	24.3
	Brazil	4.0	13.6	17.8	30.0	3.4	15.4	21.4	38.0
	Colombia	1.5	5.7	7.7	13.4	1.2	6.2	8.9	16.6
	Costa Rica	7.2	17.8	21.4	29.4	6.6	19.6	23.7	33.2
	Cuba	5.5	16.8	21.6	33.6	2.7	10.7	13.9	24.2
	Dominica	13.9	30.3	36.0	48.4	7.3	22.3	28.1	42.8
	Dominican Republic	3.7	12.4	15.9	25.5	1.4	6.0	8.2	15.0
	Ecuador	3.0	10.6	13.8	22.9	2.0	10.9	15.2	27.6
	El Salvador	2.9	9.5	12.5	20.6	1.7	7.4	10.4	19.1
	Grenada	3.7	11.7	15.1	25.3	1.6	7.4	10.5	20.5
	Guatemala	1.6	4.6	6.0	10.4	2.2	7.6	10.4	19.0
	Guyana	6.1	14.5	18.4	27.1	2.4	6.8	9.1	14.8
	Haiti	1.1	3.2	4.3	8.2	0.5	2.5	3.7	8.7
	Honduras	2.1	5.7	7.1	11.5	1.2	4.2	5.6	10.0
	Jamaica	9.6	23.1	27.7	38.7	2.8	9.5	12.4	20.7
	Mexico	4.9	14.2	18.4	30.1	3.8	15.8	21.2	36.2
	Nicaragua	2.5	7.4	9.7	15.9	2.4	8.3	11.0	19.1
	Panama	4.3	13.9	18.1	30.2	4.0	14.0	18.7	32.2
	Paraguay	2.8	10.4	13.1	20.4	3.0	12.7	16.7	26.1
	Peru	4.6	13.1	15.4	21.8	3.8	11.3	13.5	19.6
	Puerto Rico	12.1	27.1	30.7	38.7	11.3	27.2	31.0	39.4
	Saint Kitts and Nevis	6.4	18.9	23.8	36.8	4.0	14.5	19.4	32.9
	Saint Lucia	3.3	9.9	13.1	22.1	2.0	6.7	9.1	16.4
	Saint Vincent and the Grenadines	3.0	9.8	13.0	21.9	1.2	5.0	7.2	13.9
	Suriname	1.1	3.7	4.9	8.9	0.4	1.5	2.1	4.4
	Trinidad and Tobago	3.3	10.0	12.6	20.5	2.9	9.2	11.9	20.0
	United States Virgin Islands	20.8	35.0	38.5	47.6	12.5	25.4	28.7	37.7
	Venezuela (Bolivarian Republic of)	4.6	13.4	17.4	29.3	4.6	15.0	20.5	35.8

10.0 50.0

		10 to 14							
		Female				Male			
		1990	2021	2030	2050	1990	2021	2030	2050
Latin America and Caribbean	Antigua and Barbuda	5.0	15.0	19.4	31.1	3.2	11.8	15.7	27.6
	Bahamas	7.5	20.1	24.9	37.3	5.3	17.5	22.6	36.4
	Barbados	6.4	18.1	22.6	33.9	5.1	17.9	23.0	36.0
	Belize	7.2	17.6	21.0	29.3	3.5	11.9	14.8	23.8
	Bermuda	5.8	17.8	22.2	34.0	6.6	21.7	27.4	41.8
	Bolivia (Plurinational State of)	2.5	8.6	11.1	18.7	2.2	8.5	11.4	20.2
	Brazil	2.3	9.0	12.1	22.1	2.2	11.9	17.0	32.3
	Colombia	1.4	5.3	7.2	12.6	1.2	6.4	9.1	17.0
	Costa Rica	5.8	15.2	18.1	25.5	5.7	17.7	21.8	31.1
	Cuba	5.0	15.8	20.3	32.0	3.2	12.2	16.4	27.8
	Dominica	10.0	24.0	28.8	40.9	4.9	17.2	22.4	36.6
	Dominican Republic	3.0	10.7	14.0	23.2	1.6	6.9	9.4	17.2
	Ecuador	2.2	7.6	10.2	18.0	1.8	8.9	12.6	24.3
	El Salvador	3.8	11.7	15.1	24.3	2.2	9.4	12.8	22.9
	Grenada	3.3	10.6	13.9	23.8	1.5	7.2	10.4	20.8
	Guatemala	1.7	4.8	6.3	10.7	2.0	7.4	10.2	18.7
	Guyana	4.4	11.6	15.1	23.4	2.4	7.5	10.2	16.7
	Haiti	0.9	2.6	3.5	6.8	0.3	1.8	2.7	6.7
	Honduras	2.1	5.8	7.2	11.6	1.3	4.3	5.7	10.2
	Jamaica	7.6	19.5	23.8	34.5	2.2	8.1	10.7	18.8
	Mexico	4.0	14.4	18.7	30.4	4.3	18.2	24.4	40.5
	Nicaragua	2.1	6.2	8.2	13.8	2.0	7.2	9.6	16.9
	Panama	2.9	10.0	13.5	24.0	2.3	8.9	12.3	23.1
	Paraguay	2.2	8.4	10.9	17.3	2.4	10.8	14.1	22.9
	Peru	2.6	7.9	9.7	14.4	2.9	9.4	11.4	16.8
	Puerto Rico	8.8	21.9	25.1	32.6	9.3	24.2	27.9	36.1
	Saint Kitts and Nevis	5.4	16.7	21.5	34.2	3.5	14.3	19.3	33.5
	Saint Lucia	3.2	10.3	13.9	23.8	1.7	6.8	9.6	18.5
	Saint Vincent and the Grenadines	2.4	8.5	11.4	19.7	1.0	4.6	6.8	13.6
	Suriname	1.5	5.0	6.5	11.6	0.8	2.9	4.0	7.9
	Trinidad and Tobago	4.2	12.2	15.3	24.2	4.8	15.1	19.1	30.5
	United States Virgin Islands	16.2	29.5	33.4	42.4	10.2	22.1	25.1	33.9
	Venezuela (Bolivarian Republic of)	4.3	12.8	16.8	28.4	4.5	15.0	20.4	35.8

10.0 50.0

	15 to 19								
		Female				Male			
		1990	2021	2030	2050	1990	2021	2030	2050
Latin America and Caribbean	Antigua and Barbuda	4.4	13.6	17.6	28.7	2.0	7.8	10.8	20.6
	Bahamas	6.7	18.3	22.9	34.9	3.3	12.3	16.5	28.6
	Barbados	6.8	18.8	23.4	34.9	3.4	13.5	18.2	30.4
	Belize	8.2	19.3	22.8	31.4	2.6	9.3	11.9	19.7
	Bermuda	5.0	15.6	19.5	30.8	4.1	15.1	19.7	32.8
	Bolivia (Plurinational State of)	2.0	7.3	9.7	16.9	1.3	5.6	7.6	14.5
	Brazil	2.1	9.0	12.3	22.4	1.4	9.5	14.0	28.1
	Colombia	1.7	6.8	9.0	15.4	1.1	6.4	9.2	17.1
	Costa Rica	5.1	13.3	16.2	23.2	3.5	11.9	15.3	23.0
	Cuba	4.8	15.8	20.0	31.6	2.4	10.0	13.5	23.9
	Dominica	8.4	21.3	26.0	37.8	2.7	10.9	15.0	27.0
	Dominican Republic	2.8	9.9	13.0	21.9	1.2	5.8	8.1	15.1
	Ecuador	3.3	10.5	13.8	23.1	1.5	8.1	11.6	22.7
	El Salvador	4.5	13.3	17.2	27.0	2.0	8.8	12.2	22.0
	Grenada	2.8	9.4	12.4	21.6	0.9	4.7	7.0	15.1
	Guatemala	1.8	5.4	6.9	11.6	1.1	4.8	6.7	13.1
	Guyana	3.6	9.9	13.0	20.6	1.7	5.5	7.5	12.9
	Haiti	0.8	2.5	3.3	6.5	0.2	0.9	1.4	3.8
	Honduras	2.3	6.1	7.7	12.4	0.8	2.9	3.9	7.3
	Jamaica	6.7	18.1	22.3	32.8	1.4	5.7	8.0	14.7
	Mexico	4.7	18.0	23.0	35.8	3.4	17.1	23.3	39.2
	Nicaragua	3.0	8.5	11.0	17.8	1.8	6.6	9.0	16.1
	Panama	2.2	7.9	10.8	19.7	1.2	5.0	7.2	14.8
	Paraguay	1.8	7.3	9.5	15.5	1.4	7.0	9.4	16.2
	Peru	1.9	6.4	7.9	12.0	1.6	5.5	6.9	10.7
	Puerto Rico	8.1	19.7	22.7	30.0	5.9	17.0	20.3	27.8
	Saint Kitts and Nevis	4.6	15.0	19.4	31.6	2.1	9.6	13.3	25.3
	Saint Lucia	3.1	10.0	13.1	22.7	0.9	4.0	5.8	12.5
	Saint Vincent and the Grenadines	2.1	7.5	10.2	18.0	0.6	2.9	4.3	9.1
	Suriname	2.7	8.7	10.9	18.1	1.1	4.6	6.2	11.6
	Trinidad and Tobago	4.7	12.6	16.0	25.2	4.1	13.3	17.1	28.1
	United States Virgin Islands	13.8	26.2	29.6	38.4	6.8	16.1	18.8	26.6
	Venezuela (Bolivarian Republic of)	4.3	12.7	16.8	28.4	2.8	10.6	14.8	28.2

10.0 50.0

	20 to 24								
		Female				Male			
		1990	2021	2030	2050	1990	2021	2030	2050
Latin America and Caribbean	Antigua and Barbuda	8.0	22.2	27.4	40.5	3.6	12.7	17.1	29.5
	Bahamas	11.5	28.1	33.2	46.4	5.8	18.5	23.8	37.8
	Barbados	11.7	28.5	34.0	46.5	5.5	19.1	24.6	38.4
	Belize	14.9	30.7	34.8	44.6	5.5	16.5	20.6	30.9
	Bermuda	9.0	24.4	29.7	42.6	6.7	22.1	27.7	42.2
	Bolivia (Plurinational State of)	3.6	11.9	15.3	24.6	2.4	9.3	12.0	21.0
	Brazil	4.8	17.0	22.3	36.0	2.8	15.5	21.5	38.7
	Colombia	3.5	14.0	17.8	27.1	2.0	9.9	13.6	23.4
	Costa Rica	8.5	20.6	24.2	32.5	4.4	14.2	17.6	25.3
	Cuba	9.7	25.6	31.2	44.5	4.3	16.0	20.5	33.0
	Dominica	14.2	31.6	37.2	49.7	4.4	16.0	21.1	35.1
	Dominican Republic	5.8	18.5	23.1	34.7	2.8	10.1	13.6	23.5
	Ecuador	5.7	15.8	19.9	31.2	2.8	12.1	16.8	30.2
	El Salvador	8.4	21.7	26.8	38.4	5.4	18.8	24.2	37.6
	Grenada	6.4	18.2	23.0	35.3	1.8	8.3	11.9	23.1
	Guatemala	5.2	13.3	16.4	24.5	2.7	10.4	13.7	23.2
	Guyana	7.0	17.1	21.9	31.9	2.9	8.9	12.1	19.4
	Haiti	2.1	6.0	7.8	14.1	0.3	1.5	2.2	5.5
	Honduras	3.4	8.8	11.1	17.1	2.2	7.1	9.3	15.8
	Jamaica	11.7	28.0	32.9	44.5	2.8	10.1	13.4	23.0
	Mexico	9.0	25.4	30.9	44.6	5.5	22.7	29.7	46.5
	Nicaragua	9.1	21.0	25.4	35.7	5.1	15.7	19.8	30.7
	Panama	4.1	13.4	17.4	29.0	2.1	8.7	12.2	23.3
	Paraguay	4.2	14.1	17.7	26.4	2.2	10.7	14.1	22.8
	Peru	4.2	14.0	16.7	23.2	3.1	10.9	13.2	19.1
	Puerto Rico	12.0	29.4	33.1	41.2	10.7	23.9	27.8	36.0
	Saint Kitts and Nevis	8.6	24.2	29.9	43.9	3.9	15.2	20.6	35.2
	Saint Lucia	7.3	20.1	25.1	38.1	1.8	7.4	10.6	20.4
	Saint Vincent and the Grenadines	4.2	13.4	17.6	28.2	1.1	5.1	7.3	14.4
	Suriname	4.2	12.5	15.5	24.5	1.7	6.5	8.4	15.2
	Trinidad and Tobago	9.7	21.6	26.2	37.6	6.4	18.7	23.6	36.1
	United States Virgin Islands	23.2	40.1	44.1	53.2	10.2	21.3	24.6	33.4
	Venezuela (Bolivarian Republic of)	8.3	21.5	26.8	40.7	4.9	16.1	21.7	37.4

10.0 50.0

North Africa and Middle East	5 to 9 Female 1990	Female 2021	Female 2030	Female 2050	Male 1990	Male 2021	Male 2030	Male 2050
Afghanistan	2.9	4.9	5.6	8.4	0.7	3.6	5.0	12.7
Algeria	3.1	15.4	21.8	39.2	1.3	9.8	15.0	30.8
Bahrain	8.2	34.5	43.8	61.6	3.6	20.9	28.7	46.8
Egypt	6.7	30.1	40.0	61.0	3.9	23.1	33.1	55.9
Iran (Islamic Republic of)	3.7	22.7	31.3	51.2	1.9	17.1	24.8	45.0
Iraq	3.5	16.4	21.7	35.3	2.3	13.9	18.9	31.8
Jordan	2.5	15.2	20.7	35.3	2.3	11.9	15.8	26.6
Kuwait	8.3	36.0	43.7	59.4	12.4	42.6	49.5	63.1
Lebanon	6.0	22.2	27.3	40.7	4.0	20.1	25.3	39.3
Libya	4.1	26.6	36.2	57.4	3.3	23.1	32.1	53.2
Morocco	4.1	13.4	17.8	28.6	2.6	11.6	16.6	30.3
Oman	4.0	25.8	34.7	55.4	2.1	20.0	28.1	49.6
Palestine	2.3	11.1	14.4	23.8	1.8	10.0	13.0	22.0
Qatar	5.8	31.0	39.4	58.1	8.4	33.8	42.2	59.2
Saudi Arabia	7.3	35.7	44.0	60.3	7.3	36.9	45.1	61.4
Sudan	2.5	9.5	12.4	21.1	1.4	8.2	11.2	21.1
Syrian Arab Republic	5.7	31.8	40.9	60.8	3.4	28.4	38.3	59.8
Tunisia	3.2	18.0	25.9	46.1	2.1	16.6	24.1	43.8
Türkiye	2.4	10.4	13.8	22.1	1.7	12.3	16.6	27.5
United Arab Emirates	7.9	40.7	50.7	69.4	12.1	47.0	56.5	73.1
Yemen	4.0	9.1	11.1	18.4	0.9	9.1	14.2	32.0

10.0 50.0

North Africa and Middle East	10 to 14 Female 1990	Female 2021	Female 2030	Female 2050	Male 1990	Male 2021	Male 2030	Male 2050
Afghanistan	2.7	4.5	5.2	7.8	0.9	4.5	6.5	15.8
Algeria	2.6	13.4	19.2	35.7	1.4	10.5	16.2	32.9
Bahrain	8.3	35.1	44.3	61.9	4.3	24.1	32.9	51.6
Egypt	4.7	24.0	33.3	54.8	2.6	17.7	26.6	49.1
Iran (Islamic Republic of)	2.7	18.4	26.1	45.6	1.6	15.7	23.4	43.9
Iraq	2.6	13.0	17.8	30.6	2.7	16.2	22.1	36.3
Jordan	3.2	18.3	24.0	39.1	3.5	16.5	21.3	33.9
Kuwait	6.4	31.3	38.9	54.8	11.9	43.0	50.3	64.0
Lebanon	6.1	23.2	28.4	42.0	4.7	22.7	28.1	42.0
Libya	4.1	26.6	35.8	56.8	3.5	25.2	34.8	56.2
Morocco	2.2	7.8	10.8	19.0	1.3	7.0	10.6	21.9
Oman	3.5	23.8	32.6	53.3	1.9	18.9	27.0	48.8
Palestine	2.4	12.8	16.2	26.0	2.3	12.9	16.6	27.0
Qatar	4.5	26.3	34.5	53.4	9.2	36.2	44.3	61.3
Saudi Arabia	6.0	31.9	39.7	56.3	6.2	34.9	43.6	60.4
Sudan	2.4	9.9	13.1	22.4	2.0	11.6	15.9	28.6
Syrian Arab Republic	3.7	24.6	33.1	53.2	2.4	24.2	33.6	56.0
Tunisia	2.1	13.8	20.6	39.3	1.2	10.8	16.6	34.5
Türkiye	1.9	8.6	11.5	18.8	1.5	10.8	15.0	25.3
United Arab Emirates	6.0	35.3	45.5	65.5	11.3	46.2	55.5	72.7
Yemen	1.7	4.5	5.8	10.5	0.7	7.7	12.0	28.8

10.0 50.0

		15 to 19							
		Female				Male			
		1990	2021	2030	2050	1990	2021	2030	2050
North Africa and Middle East	Afghanistan	2.5	4.5	5.2	7.8	0.5	2.9	4.2	11.2
	Algeria	2.3	12.4	18.1	34.4	0.9	8.0	12.5	27.2
	Bahrain	9.9	35.6	44.7	62.4	3.1	18.6	26.0	44.3
	Egypt	4.3	20.3	29.1	50.7	1.3	9.9	16.2	35.8
	Iran (Islamic Republic of)	2.7	16.7	24.3	43.7	1.0	10.6	16.8	35.5
	Iraq	2.3	12.3	16.4	28.8	2.0	13.1	18.0	31.3
	Jordan	3.5	16.9	22.4	37.5	2.5	11.7	15.5	26.7
	Kuwait	5.9	28.2	35.9	52.3	10.2	32.6	39.8	54.9
	Lebanon	5.6	21.4	26.4	39.8	3.1	17.3	22.2	35.5
	Libya	3.7	25.2	34.8	56.0	2.2	18.6	27.0	48.3
	Morocco	1.8	6.0	8.5	15.5	0.7	3.7	5.9	13.6
	Oman	3.0	20.3	28.1	48.8	1.1	12.6	19.3	39.9
	Palestine	3.5	16.4	20.2	31.0	2.7	14.2	18.2	28.9
	Qatar	3.7	23.8	31.7	50.9	5.9	30.0	38.3	56.1
	Saudi Arabia	5.4	27.0	34.7	51.9	5.0	31.5	40.0	57.4
	Sudan	2.4	10.0	13.3	22.5	1.5	9.9	13.6	25.2
	Syrian Arab Republic	3.2	21.2	29.2	49.5	1.4	17.0	25.4	47.5
	Tunisia	1.6	12.2	18.3	36.5	0.6	6.3	10.4	24.9
	Türkiye	1.7	9.0	12.1	19.8	1.0	7.1	10.1	18.5
	United Arab Emirates	6.3	29.8	39.6	60.5	8.9	33.9	43.8	63.6
	Yemen	1.2	3.4	4.4	8.2	0.4	4.6	7.8	21.2

10.0 50.0

		20 to 24							
		Female				Male			
		1990	2021	2030	2050	1990	2021	2030	2050
North Africa and Middle East	Afghanistan	5.1	11.0	12.4	17.4	1.4	7.8	10.8	23.8
	Algeria	4.7	20.0	27.5	45.7	1.3	8.9	14.1	30.3
	Bahrain	12.1	40.6	50.2	67.0	5.6	29.0	38.1	56.7
	Egypt	9.5	32.7	42.9	64.0	2.8	16.8	25.5	47.8
	Iran (Islamic Republic of)	6.2	27.6	36.8	56.6	2.1	17.9	26.7	48.0
	Iraq	5.8	21.8	28.0	42.7	3.8	19.9	26.2	41.3
	Jordan	8.2	28.0	35.2	51.1	4.3	16.2	20.9	33.7
	Kuwait	10.8	39.1	46.8	61.8	15.5	42.7	49.8	63.8
	Lebanon	6.9	24.3	29.6	42.8	5.3	22.6	27.9	41.3
	Libya	6.6	34.3	44.8	64.7	3.5	25.0	34.7	56.1
	Morocco	4.3	12.8	17.1	27.8	1.0	6.5	9.9	21.0
	Oman	10.3	37.5	47.0	66.2	3.2	21.8	30.5	52.4
	Palestine	8.8	30.8	35.9	47.9	6.0	25.2	30.8	43.3
	Qatar	10.0	40.4	49.2	66.3	11.5	41.7	50.1	66.1
	Saudi Arabia	14.6	34.3	42.5	59.0	7.6	33.3	42.0	59.7
	Sudan	6.3	20.0	24.6	36.4	3.1	17.2	22.8	38.0
	Syrian Arab Republic	5.8	32.2	41.3	60.8	2.9	26.4	36.1	58.3
	Tunisia	3.3	20.0	28.0	48.0	1.7	13.6	20.4	39.4
	Türkiye	5.7	17.7	22.4	32.5	2.0	11.4	15.7	26.1
	United Arab Emirates	9.3	44.4	54.3	72.2	10.3	44.0	53.6	71.5
	Yemen	2.5	6.7	8.6	14.7	0.5	6.3	10.2	25.5

10.0 50.0

Sub-Saharan Africa	5 to 9							
	Female				Male			
	1990	2021	2030	2050	1990	2021	2030	2050
Angola	3.9	12.1	16.1	27.9	2.0	10.9	15.3	29.2
Benin	2.6	4.6	5.6	9.1	1.4	5.3	8.2	18.1
Botswana	3.4	11.4	15.1	25.5	1.4	9.4	14.0	28.2
Burkina Faso	4.2	6.3	7.1	9.4	3.3	7.5	9.5	15.6
Burundi	1.3	2.7	3.5	6.3	0.6	2.1	3.1	7.9
Cabo Verde	2.1	6.6	9.0	14.8	1.2	5.5	7.9	14.8
Cameroon	3.1	10.2	13.6	23.2	2.3	10.4	14.9	28.8
Central African Republic	4.0	10.4	14.5	27.1	2.1	11.1	18.6	40.9
Chad	2.2	3.6	4.1	6.0	1.3	3.6	4.7	9.2
Comoros	5.7	17.2	21.9	33.7	4.0	21.9	30.0	49.5
Congo	2.2	11.0	15.8	30.4	1.0	6.1	9.2	19.8
Côte d'Ivoire	0.6	1.5	1.8	3.0	0.4	2.1	3.3	8.1
Democratic Republic of the Congo	1.6	7.8	11.7	23.8	1.5	7.4	10.9	21.6
Djibouti	3.0	6.1	7.5	10.7	1.9	8.0	11.5	21.6
Equatorial Guinea	9.9	32.6	38.6	51.6	6.7	32.3	39.1	53.8
Eritrea	1.5	3.3	4.2	7.1	0.7	5.0	8.1	20.7
Eswatini	4.2	12.9	16.3	25.6	1.8	8.9	12.2	22.9
Ethiopia	1.8	2.5	2.8	3.6	0.6	2.4	3.4	7.2
Gabon	1.8	7.9	10.9	19.1	1.0	6.0	8.6	16.2
Gambia	0.7	1.2	1.6	2.6	0.4	1.3	1.7	3.1
Ghana	0.7	3.4	4.9	9.5	0.6	3.0	4.3	8.5
Guinea	2.2	3.5	4.1	6.2	1.0	3.3	4.7	9.8
Guinea-Bissau	2.7	4.9	6.0	9.2	1.5	5.4	7.7	15.8
Kenya	1.7	5.1	7.2	12.9	1.3	5.2	7.3	13.6
Lesotho	1.4	3.4	4.4	7.3	1.0	4.7	6.5	12.8
Liberia	2.2	5.6	7.2	11.9	2.1	6.4	8.6	14.6
Madagascar	1.5	4.0	5.2	9.1	1.0	3.4	4.7	8.9
Malawi	4.3	10.8	13.3	20.4	3.2	11.0	14.1	22.7
Mali	1.5	2.1	2.5	3.5	1.2	3.1	4.0	7.3
Mauritania	3.2	7.9	10.4	16.8	1.9	6.1	8.5	15.0
Mozambique	2.4	5.6	7.3	12.6	1.4	4.6	6.5	12.8
Namibia	3.3	10.2	12.5	19.0	2.1	9.7	12.6	21.4
Niger	1.6	2.2	2.5	3.3	1.1	2.2	2.7	4.7
Nigeria	1.5	4.2	5.6	9.8	1.1	4.5	6.3	12.9
Rwanda	2.3	5.6	7.2	12.1	1.6	4.9	6.7	11.9
Sao Tome and Principe	2.2	6.4	8.6	14.7	1.2	4.4	6.1	11.1
Senegal	2.0	3.9	4.7	7.0	0.9	2.9	4.2	8.5
Sierra Leone	4.8	7.6	9.1	13.2	2.2	5.2	6.9	12.4
Somalia	1.9	3.7	4.5	7.4	1.4	4.6	6.3	13.0
South Africa	5.1	15.6	19.7	31.3	3.1	11.6	14.8	23.8
South Sudan	5.0	6.4	6.9	8.7	2.3	7.1	10.8	24.9
Togo	1.7	4.3	5.8	10.1	0.9	4.0	6.1	13.3
Uganda	1.8	5.7	7.5	13.9	1.4	5.2	6.9	12.9
United Republic of Tanzania	1.9	6.4	9.2	17.3	1.6	6.2	8.7	16.5
Zambia	4.6	12.3	15.2	23.0	4.8	15.2	19.2	29.8
Zimbabwe	0.5	1.8	2.5	5.2	0.5	2.2	3.1	6.5

10.0 50.0

Sub-Saharan Africa	10 to 14							
	Female				Male			
	1990	2021	2030	2050	1990	2021	2030	2050
Angola	2.3	7.6	10.4	19.7	1.4	8.3	12.2	25.0
Benin	1.5	2.6	3.2	5.4	0.5	2.2	3.6	9.2
Botswana	2.3	8.8	11.8	20.9	0.7	5.2	8.1	18.7
Burkina Faso	1.9	3.0	3.5	4.9	1.6	3.8	5.0	9.1
Burundi	0.7	1.4	1.9	3.7	0.4	1.4	2.3	6.0
Cabo Verde	1.3	4.8	6.7	11.6	0.7	3.8	5.6	11.3
Cameroon	2.6	8.6	11.7	20.4	1.5	7.4	11.0	22.9
Central African Republic	2.6	7.5	11.0	21.9	1.6	9.4	16.5	38.7
Chad	1.1	1.8	2.1	3.2	0.8	2.3	3.1	6.4
Comoros	3.3	10.9	14.2	23.7	2.4	15.7	22.8	41.6
Congo	2.2	11.4	16.5	31.9	0.6	4.4	6.9	16.2
Côte d'Ivoire	0.4	0.9	1.2	2.1	0.4	2.1	3.3	8.1
Democratic Republic of the Congo	0.4	2.4	3.9	10.0	1.0	5.4	8.0	17.1
Djibouti	2.9	5.9	7.2	10.2	1.8	7.5	10.9	20.8
Equatorial Guinea	6.4	23.9	29.2	41.3	4.8	25.9	32.2	46.5
Eritrea	0.6	1.5	1.9	3.5	0.3	2.2	4.1	12.9
Eswatini	4.5	13.6	17.1	26.5	1.2	6.0	8.5	17.0
Ethiopia	1.3	1.8	2.0	2.6	0.4	1.5	2.2	4.9
Gabon	2.5	10.3	13.9	23.2	0.5	3.8	5.6	11.4
Gambia	0.9	2.0	2.5	4.3	0.3	1.1	1.6	3.0
Ghana	0.6	3.1	4.5	9.0	0.4	1.7	2.5	5.2
Guinea	1.6	2.9	3.5	5.4	0.6	2.3	3.4	7.7
Guinea-Bissau	1.5	2.9	3.5	5.7	0.9	3.7	5.6	12.5
Kenya	1.2	4.0	5.6	10.4	0.8	3.8	5.5	11.0
Lesotho	1.8	4.5	5.6	9.2	0.5	2.6	3.7	7.8
Liberia	1.4	3.9	5.1	8.6	1.6	5.3	7.2	12.5
Madagascar	0.3	0.9	1.3	2.5	0.7	2.5	3.5	7.0
Malawi	2.0	5.6	7.0	11.6	1.9	7.1	9.5	16.3
Mali	1.0	1.5	1.7	2.4	0.8	2.1	2.8	5.5
Mauritania	4.2	10.4	13.7	21.7	3.0	9.4	12.8	21.9
Mozambique	1.4	3.3	4.4	8.0	0.8	2.8	4.1	8.4
Namibia	2.4	7.5	9.3	14.9	1.4	6.6	8.9	16.1
Niger	1.1	1.5	1.7	2.2	0.7	1.5	1.9	3.4
Nigeria	1.1	3.8	5.0	8.9	0.7	3.5	5.2	11.1
Rwanda	1.1	2.7	3.6	6.4	0.6	1.9	2.6	5.2
Sao Tome and Principe	1.6	5.1	6.8	11.9	1.1	4.4	6.0	11.1
Senegal	1.6	3.1	3.8	5.8	0.5	1.8	2.8	6.0
Sierra Leone	2.2	3.9	4.7	7.2	1.5	4.8	6.4	11.6
Somalia	1.5	3.1	3.8	6.3	0.9	3.1	4.4	9.9
South Africa	4.0	12.9	16.5	27.2	2.0	8.1	10.6	18.2
South Sudan	1.9	2.4	2.7	3.5	1.1	3.9	6.4	17.5
Togo	1.3	3.5	4.7	8.4	0.6	2.8	4.5	10.3
Uganda	0.7	2.4	3.3	6.7	0.6	2.3	3.2	6.5
United Republic of Tanzania	1.5	5.1	7.4	14.5	0.8	3.7	5.4	11.3
Zambia	2.4	8.0	10.1	16.3	3.2	10.7	13.9	23.2
Zimbabwe	0.7	3.1	4.2	8.3	0.4	2.2	3.1	6.5

10.0 50.0

		15 to 19							
		Female				Male			
		1990	2021	2030	2050	1990	2021	2030	2050
Sub-Saharan Africa	Angola	0.9	3.3	4.7	10.1	0.3	2.4	3.9	10.1
	Benin	1.1	2.0	2.6	4.4	0.2	0.7	1.3	3.6
	Botswana	2.2	8.4	11.3	20.1	0.2	2.0	3.4	9.0
	Burkina Faso	1.0	1.4	1.6	2.4	0.6	1.4	1.9	3.9
	Burundi	0.4	0.8	1.1	2.2	0.1	0.5	0.8	2.3
	Cabo Verde	1.3	4.6	6.3	11.1	0.4	2.1	3.2	6.8
	Cameroon	2.8	9.0	12.2	21.1	0.8	4.3	6.7	15.4
	Central African Republic	1.5	4.2	6.5	14.4	0.5	3.8	7.4	22.7
	Chad	0.6	1.1	1.3	1.9	0.2	0.7	1.0	2.2
	Comoros	2.5	8.7	11.5	20.0	0.7	6.6	10.4	23.7
	Congo	2.3	11.8	16.7	32.2	0.3	2.2	3.7	9.7
	Côte d'Ivoire	1.0	2.2	2.8	4.6	0.6	3.0	4.8	11.3
	Democratic Republic of the Congo	0.1	0.8	1.4	4.0	0.3	1.6	2.7	6.7
	Djibouti	2.9	5.6	6.7	9.6	1.1	5.1	7.5	15.2
	Equatorial Guinea	2.8	13.0	16.8	27.2	1.5	11.2	15.4	27.2
	Eritrea	0.4	1.1	1.5	2.7	0.1	0.8	1.6	6.1
	Eswatini	5.0	13.8	17.4	27.1	0.6	3.2	4.7	10.2
	Ethiopia	1.3	1.6	1.8	2.3	0.2	0.7	1.1	2.5
	Gabon	3.3	13.1	17.3	28.0	0.2	1.7	2.7	6.1
	Gambia	1.3	2.7	3.4	5.5	0.3	1.0	1.4	2.7
	Ghana	0.7	3.8	5.4	10.5	0.2	0.8	1.2	2.6
	Guinea	1.5	2.6	3.1	4.9	0.3	1.0	1.6	4.0
	Guinea-Bissau	1.0	1.9	2.4	3.9	0.3	1.2	2.0	5.1
	Kenya	1.0	3.8	5.4	10.1	0.3	1.4	2.1	4.7
	Lesotho	2.7	7.0	8.6	13.5	0.2	1.0	1.4	3.3
	Liberia	1.3	3.4	4.5	7.7	1.0	3.6	4.9	8.9
	Madagascar	0.1	0.4	0.5	1.0	0.3	1.1	1.6	3.3
	Malawi	1.1	3.5	4.5	7.8	1.0	3.5	4.9	9.1
	Mali	0.8	1.2	1.4	2.0	0.3	0.7	0.9	2.0
	Mauritania	5.3	12.5	16.3	25.1	2.5	7.8	10.8	19.1
	Mozambique	0.9	2.1	2.8	5.2	0.4	1.3	1.9	4.2
	Namibia	2.3	5.9	7.5	12.4	0.9	3.8	5.2	10.1
	Niger	0.9	1.2	1.4	1.9	0.2	0.4	0.5	1.0
	Nigeria	1.0	3.4	4.6	8.2	0.3	1.8	2.7	6.3
	Rwanda	0.7	1.9	2.6	4.7	0.1	0.5	0.7	1.5
	Sao Tome and Principe	1.4	4.6	6.3	11.2	0.7	3.4	4.7	8.9
	Senegal	1.5	2.3	2.8	4.4	0.2	0.8	1.2	2.8
	Sierra Leone	1.2	2.1	2.6	4.1	0.5	1.7	2.3	4.7
	Somalia	1.3	2.8	3.5	5.8	0.3	1.0	1.5	3.9
	South Africa	5.3	18.2	22.8	35.1	1.4	5.5	7.4	13.3
	South Sudan	0.6	0.8	0.9	1.2	0.2	0.8	1.4	5.6
	Togo	1.3	3.4	4.7	8.5	0.3	1.5	2.5	6.2
	Uganda	0.5	1.9	2.6	5.4	0.2	1.0	1.4	3.0
	United Republic of Tanzania	1.2	4.6	6.5	12.6	0.3	1.4	2.2	5.2
	Zambia	1.8	6.4	8.2	13.5	1.4	4.9	6.5	12.1
	Zimbabwe	1.1	4.4	5.9	11.1	0.2	1.3	1.8	3.7

10.0 50.0

		20 to 24							
		Female				Male			
		1990	2021	2030	2050	1990	2021	2030	2050
Sub-Saharan Africa	Angola	1.0	3.7	5.4	11.3	0.2	2.0	3.2	8.6
	Benin	3.3	5.4	6.7	10.6	0.9	3.8	5.9	14.1
	Botswana	5.0	15.9	20.5	32.5	0.4	3.4	5.5	13.9
	Burkina Faso	2.1	3.3	3.8	5.2	0.9	2.8	3.7	7.0
	Burundi	0.6	1.3	1.7	3.3	0.2	0.6	0.9	2.6
	Cabo Verde	3.2	10.1	13.5	21.3	0.9	4.5	6.7	13.0
	Cameroon	4.3	11.4	15.0	25.0	1.3	6.9	10.3	21.7
	Central African Republic	1.5	4.4	6.7	14.7	0.3	2.4	5.0	17.6
	Chad	1.1	1.7	2.0	3.0	0.3	1.1	1.5	3.3
	Comoros	3.4	11.0	14.3	23.8	0.7	6.1	9.7	22.2
	Congo	2.9	14.6	20.5	37.2	0.5	3.6	5.6	13.8
	Côte d'Ivoire	3.8	7.9	9.6	14.2	0.8	3.7	5.6	12.5
	Democratic Republic of the Congo	0.3	2.2	3.6	9.3	0.3	2.1	3.3	8.3
	Djibouti	2.1	4.2	5.1	7.5	0.7	3.4	5.1	11.1
	Equatorial Guinea	3.2	14.6	18.6	29.4	1.3	10.2	13.8	24.7
	Eritrea	0.8	1.7	2.1	3.9	0.1	1.1	2.1	7.5
	Eswatini	9.5	23.7	28.6	40.2	1.3	6.2	8.9	17.6
	Ethiopia	1.1	1.2	1.3	1.7	0.2	1.1	1.6	3.7
	Gabon	6.4	21.1	26.6	38.8	0.5	3.6	5.4	10.9
	Gambia	3.2	6.6	8.1	12.6	0.8	2.7	3.6	6.6
	Ghana	1.7	8.5	11.7	20.7	0.5	2.1	3.1	6.5
	Guinea	3.6	6.5	7.7	11.2	0.5	1.9	3.0	6.9
	Guinea-Bissau	2.2	4.2	5.1	8.0	0.4	2.0	3.2	7.9
	Kenya	2.2	7.9	10.6	18.0	0.5	2.4	3.5	7.5
	Lesotho	6.8	15.5	18.1	25.8	0.4	1.8	2.6	5.7
	Liberia	2.8	6.1	7.7	12.6	2.8	8.6	11.3	18.6
	Madagascar	0.5	1.3	1.7	3.4	0.4	1.4	2.0	4.3
	Malawi	1.9	4.4	5.7	9.5	0.8	3.1	4.1	8.0
	Mali	2.1	3.7	4.2	5.7	0.3	0.8	1.2	2.5
	Mauritania	11.1	22.7	27.9	38.9	3.0	9.8	13.4	22.8
	Mozambique	2.2	4.8	6.4	11.1	0.9	2.9	4.1	8.5
	Namibia	4.5	11.1	13.6	20.9	1.4	5.7	7.8	14.4
	Niger	2.2	2.9	3.3	4.3	0.2	0.5	0.7	1.3
	Nigeria	2.4	6.9	9.0	15.0	0.6	3.4	4.9	10.5
	Rwanda	1.3	3.7	4.9	8.4	0.2	0.9	1.3	2.7
	Sao Tome and Principe	3.6	10.1	13.1	21.0	0.7	3.3	4.5	8.4
	Senegal	4.7	6.2	7.4	10.9	0.8	2.4	3.6	7.8
	Sierra Leone	2.7	5.5	6.7	9.9	0.8	2.7	3.7	7.1
	Somalia	3.1	6.5	7.9	12.3	0.3	1.3	1.8	4.7
	South Africa	10.0	27.5	33.1	46.5	2.8	7.9	10.4	18.1
	South Sudan	1.0	1.4	1.5	2.0	0.2	0.8	1.5	5.9
	Togo	3.0	6.9	9.0	15.1	0.6	2.8	4.5	10.5
	Uganda	1.1	3.9	5.3	10.2	0.2	1.1	1.6	3.4
	United Republic of Tanzania	2.6	9.1	12.6	22.5	0.6	2.7	4.1	9.0
	Zambia	3.1	8.5	10.7	17.2	1.7	6.1	8.2	15.0
	Zimbabwe	3.3	10.3	13.2	22.1	0.5	2.3	3.3	6.9

10.0 50.0

Figure S11: Change in the percentage of 5–24-year-olds with overweight or obesity 1990-2050, by super-region and by sex

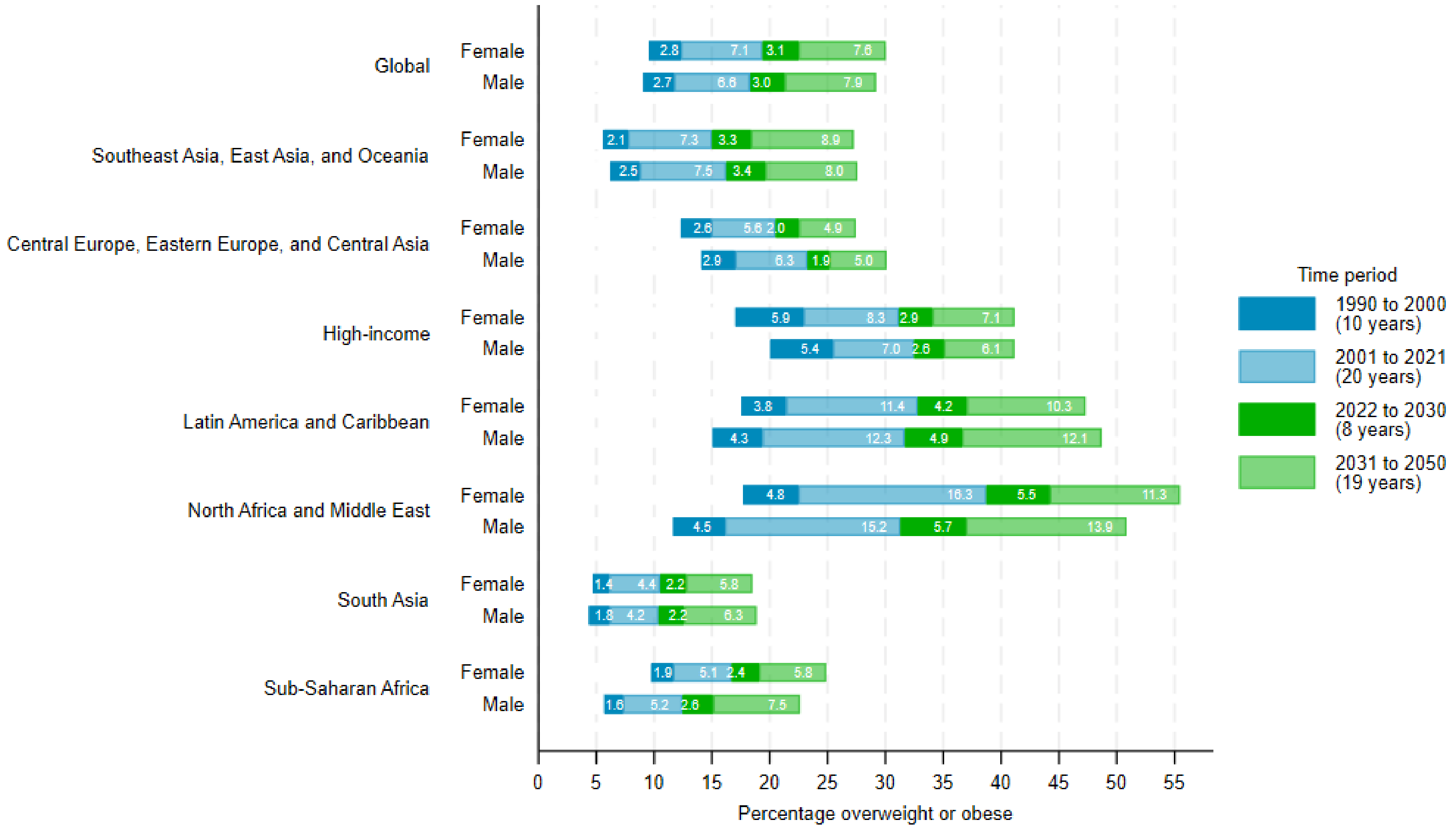

Figure S12: Change in the percentage of 5–24-year-olds with obesity 1990-2050, by super-region and by sex

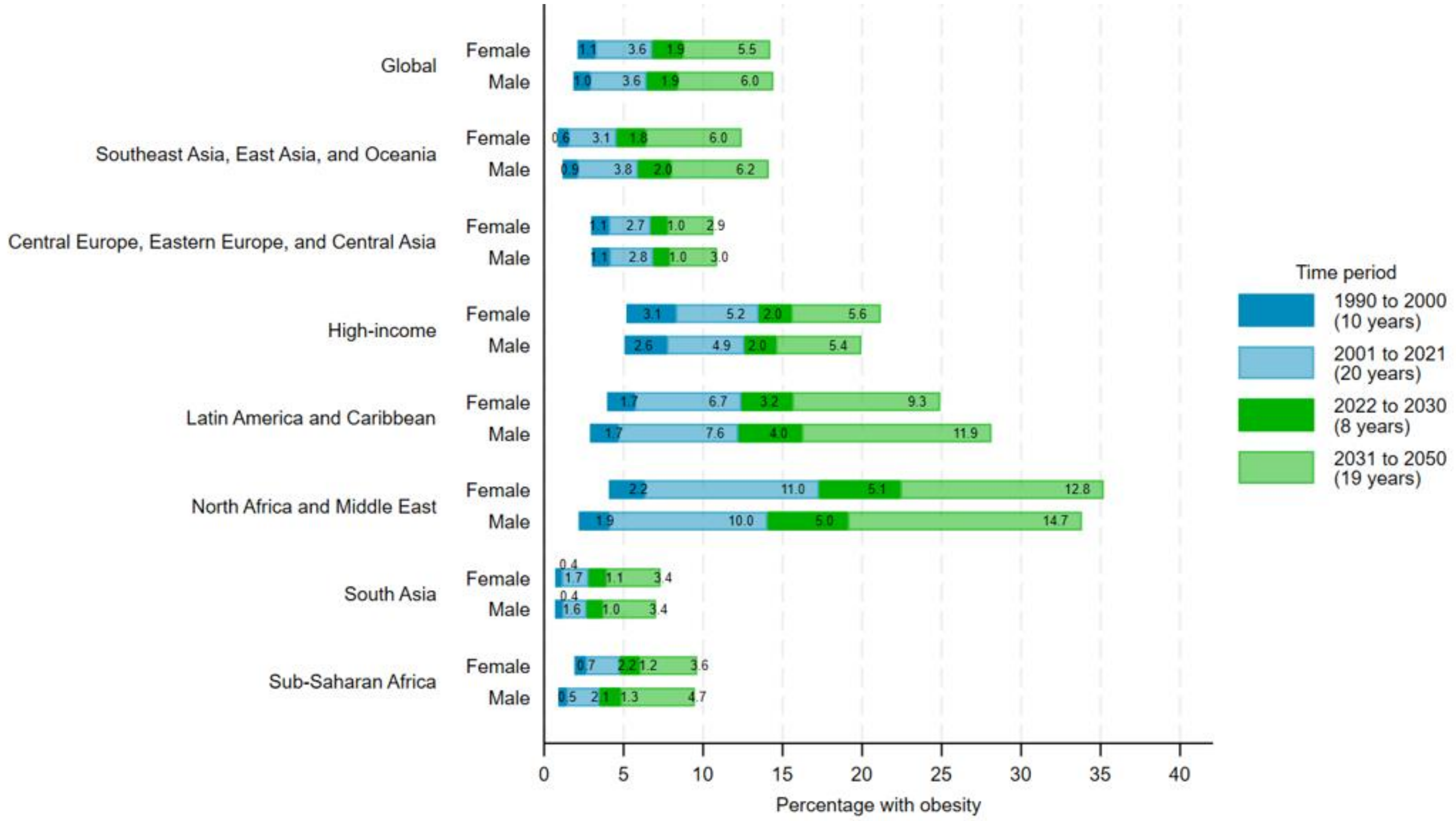

Figure S13: Overweight and obesity prevalence over time by age group and super-region

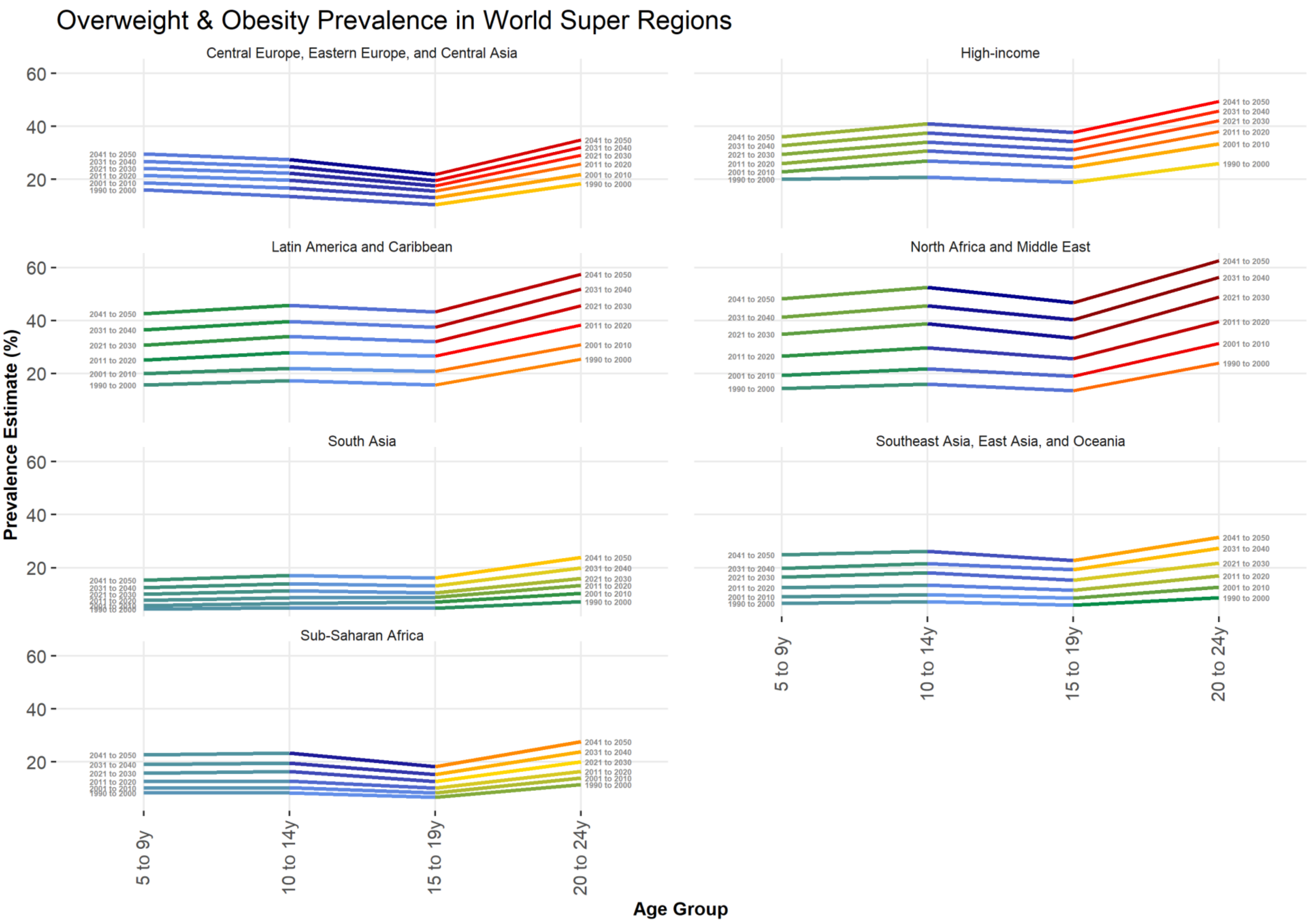

Figure S14: Obesity prevalence over time by age group and super-region

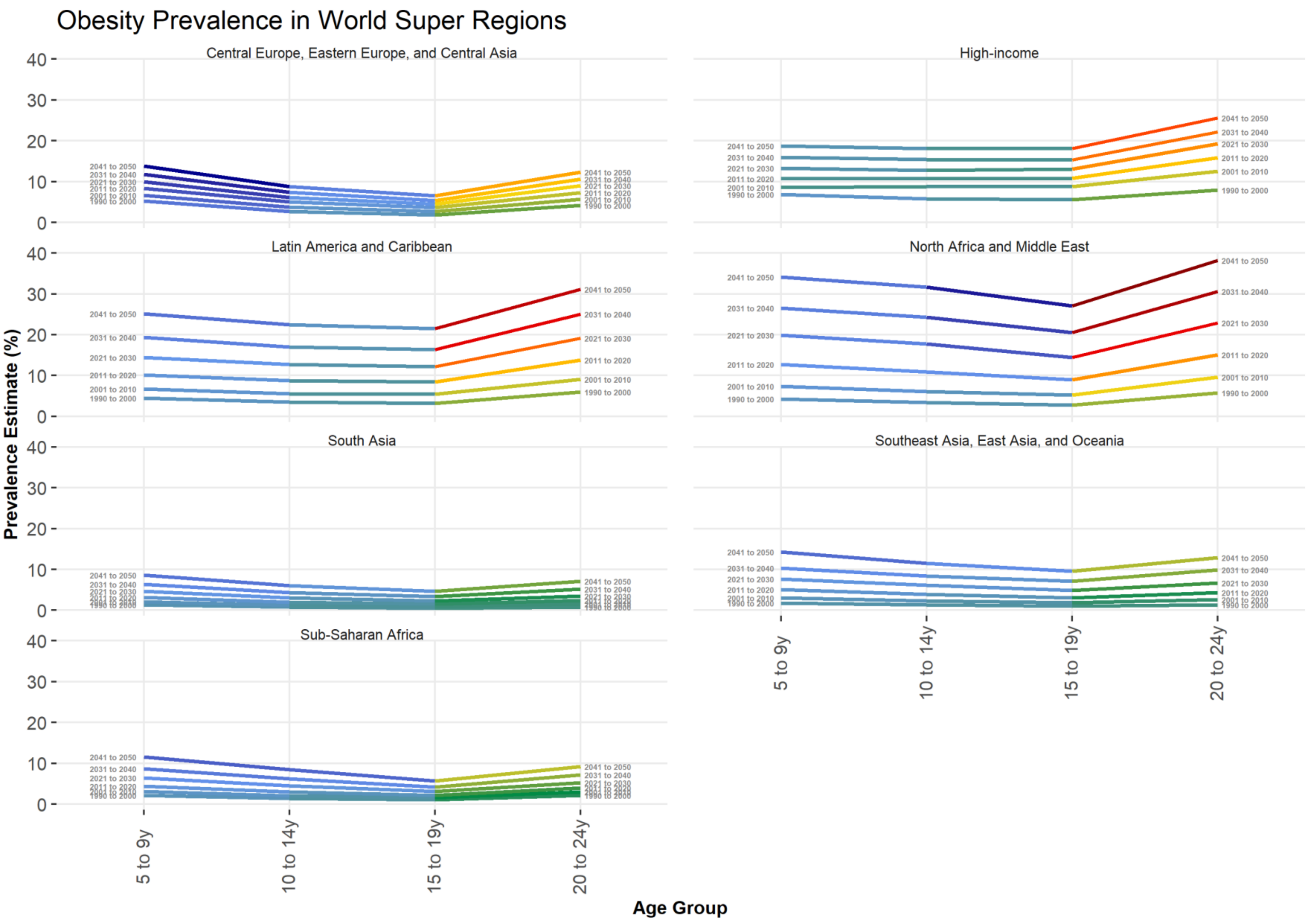

Figure S15: Mean counts of children and young adolescents (5-14 years) with obesity

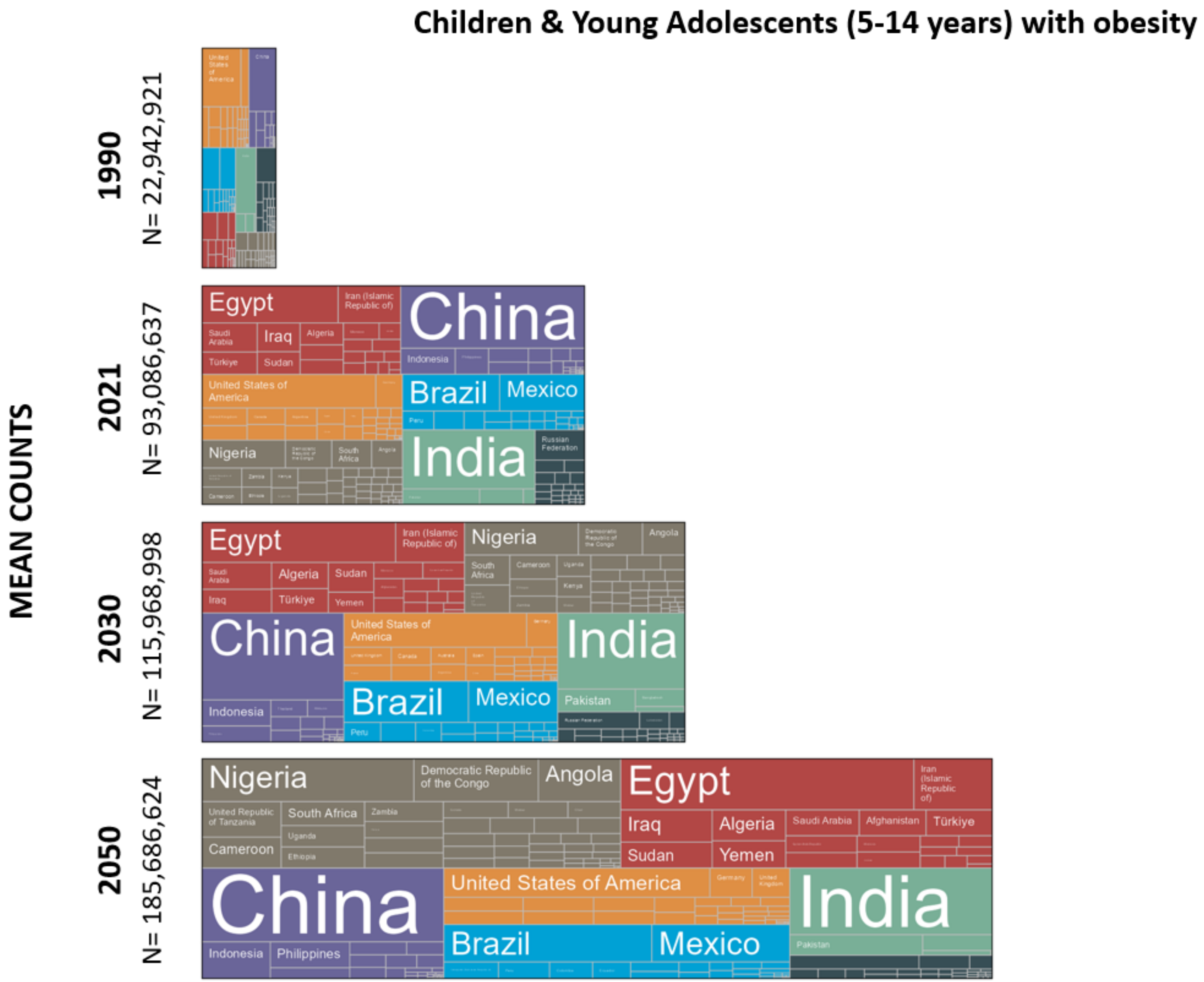

Figure S16: Mean counts of children and young adolescents (5-14 years) with overweight

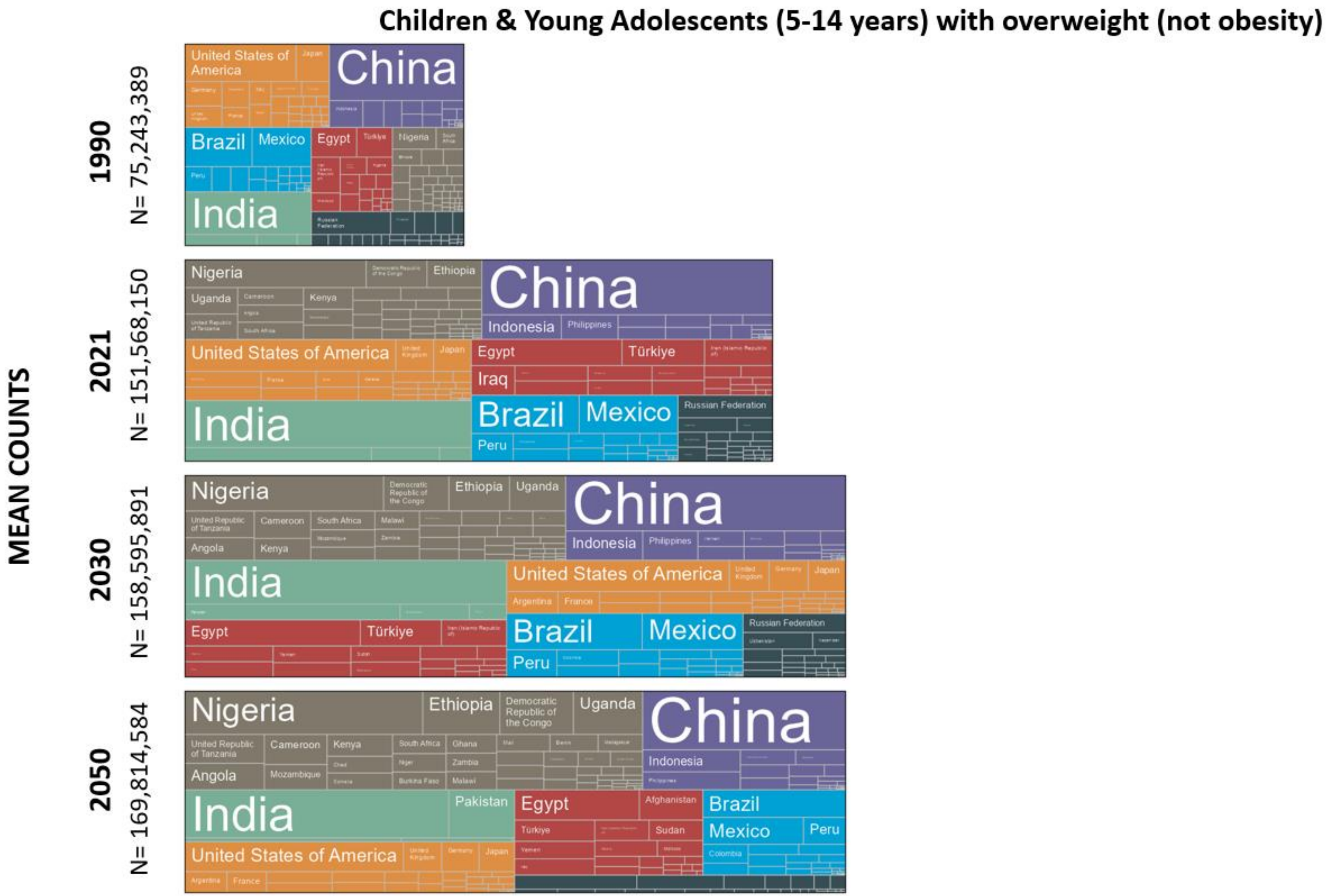

Figure S17: Mean counts of children and young adolescents (5-14 years) with overweight or obesity

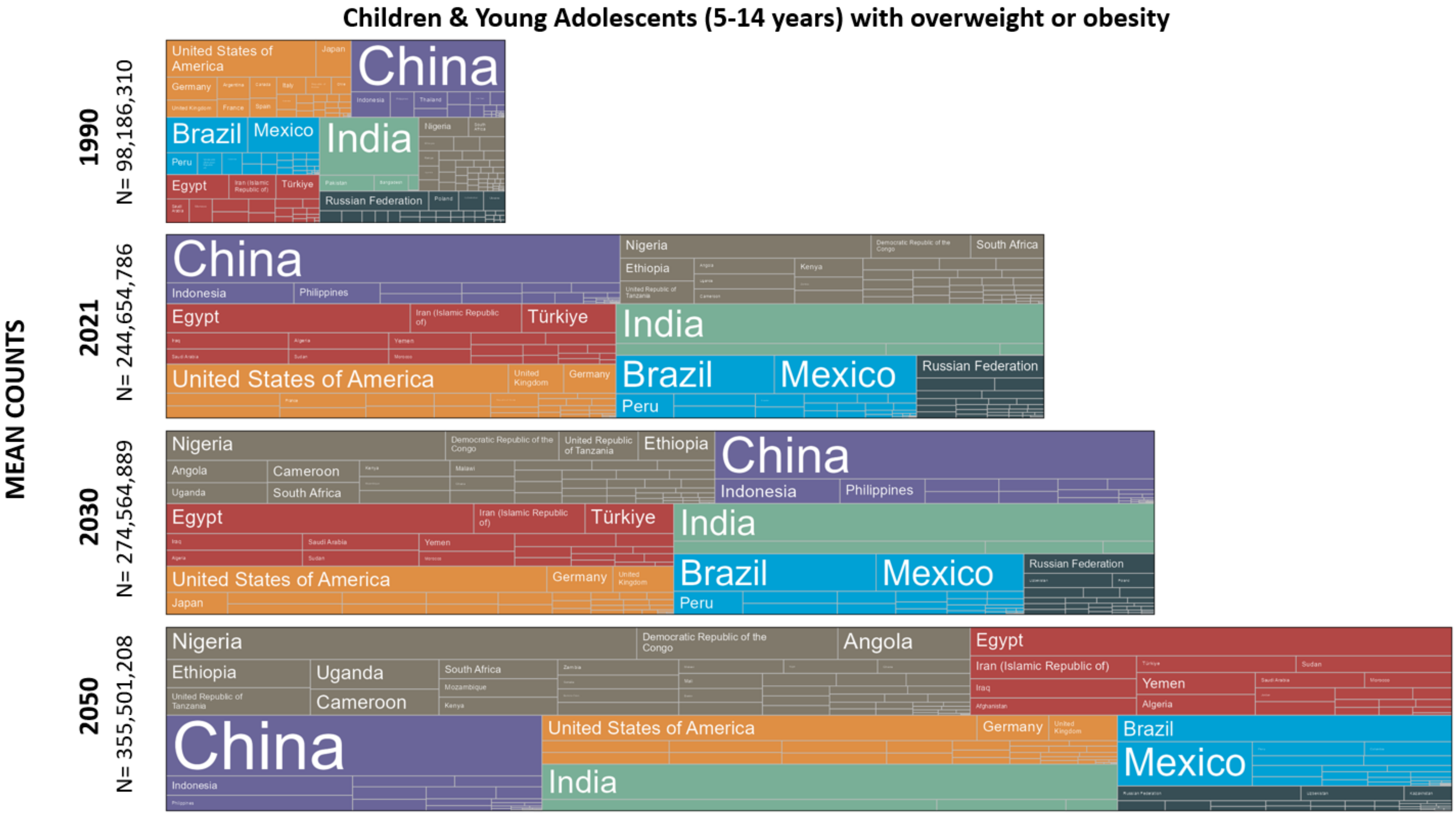

Figure S18: Mean counts of older adolescents (15-24 years) with obesity

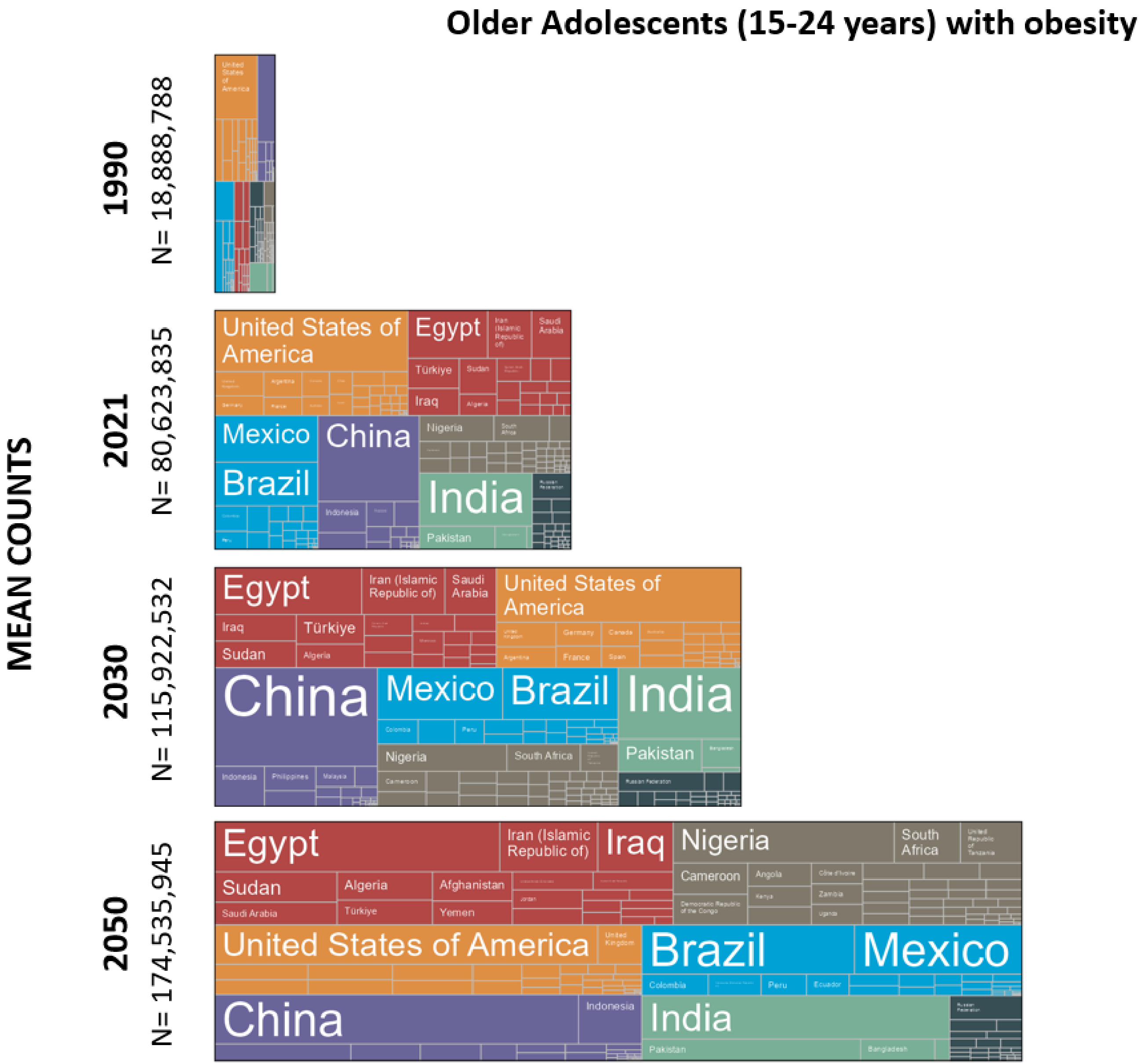

Figure S19: Mean counts of older adolescents (15-24 years) with overweight

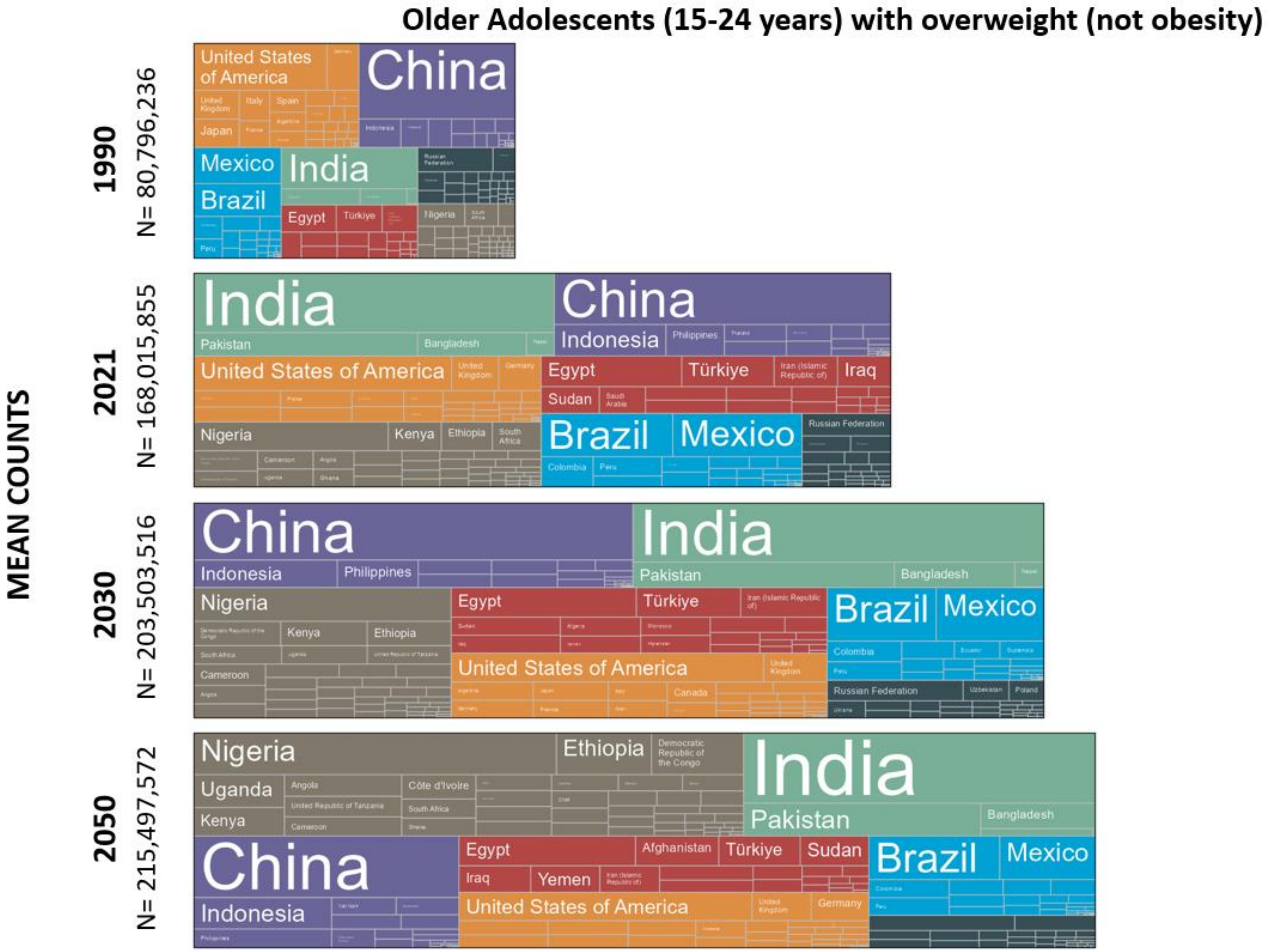

Figure S20: Mean counts of older adolescents (15-24 years) with overweight or obesity

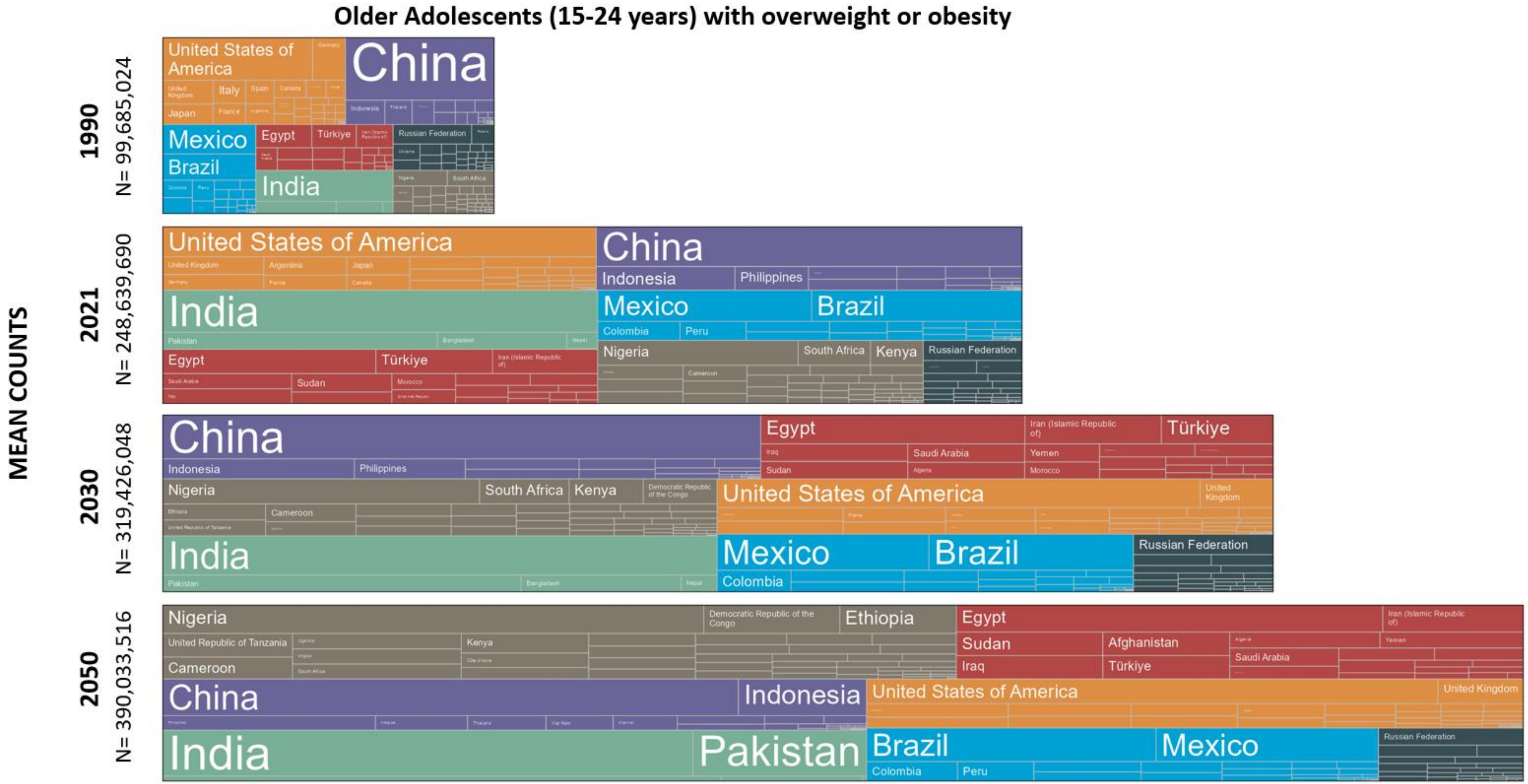

Supplement: Supplementary appendix 1 [file mmc1.pdf]
