# Supplementary material for: Global, regional, and national prevalence of child and adolescent overweight and obesity, 1990–2021, with forecasts to 2050: a forecasting study for the Global Burden of Disease Study 2021
Source: Lancet. 2025 Mar 8;405(10481):785–812. doi: 10.1016/S0140-6736(25)00397-6 (PMC11920006; doi:10.1016/S0140-6736(25)00397-6)
Supplement: Supplementary appendix 2 [file mmc2.pdf]

THE LANCET

Supplementary appendix 2

This appendix formed part of the original submission and has been peer reviewed. We post it as supplied by the authors.

Supplement to: GBD 2021 Adolescent BMI Collaborators. Global, regional, and national prevalence of child and adolescent overweight and obesity, 1990–2021, with forecasts to 2050: a forecasting study for the Global Burden of Disease Study 2021. *Lancet* 2025; published online March 3. https://doi.org/10.1016/S0140-6736(25)00397-6.

Appendix 2: Authorship appendix to "Global, regional, and national prevalence of child and adolescent overweight and obesity, 1990-2021, with forecasts to 2050: a forecasting study for the Global Burden of Disease Study 2021"

This appendix provides further authorship detail for "Global, regional, and national prevalence of child and adolescent overweight and obesity, 1990-2021, with forecasts to 2050: a forecasting study for the Global Burden of Disease Study 2021"

Table of Contents

GBD 2021 Child and Adolescent Obesity Collaborators……………………………………………………….. 2

Affiliations…….. 6

Authors' Contributions…………………………………………………………………………………………… 33

Providing data or critical feedback on data sources………………………………………………….. 33

Developing methods or computational machinery…………………………………………………….. 35

Providing critical feedback on methods or results…………………………………………………..... 35

Drafting the work or revising it critically for important intellectual content………………….. 38

Managing the estimation or publications process……………………………………………………... 42

GBD 2021 Child and Adolescent Obesity Collaborators

Jessica A Kerr*, George C Patton, Karly I Cini, Yohannes Habtegiorgis Abate, Nasir Abbas, Abdallah H A Abd Al Magied, Samar Abd ElHafeez, Sherief Abd-Elsalam, Arash Abdollahi, Meriem Abdoun, Deldar Morad Abdulah, Rizwan Suliankatchi Abdulkader, Auwal Abdullahi, Hansani Madushika Abeywickrama, Alemwork Abie, Olumide Abiodun, Shady Abohashem, Dariush Abtahi, Hasan Abualruz, Bilyaminu Abubakar, Eman Abu-Gharbieh, Hana J Abukhadijah, Niveen ME Abu-Rmeileh, Salahdein Aburuz, Ahmed Abu-Zaid, Lisa C. Adams, Mesafint Molla Adane, Isaac Yeboah Addo, Kamoru Ademola Adedokun, Nurudeen A Adegoke, Ridwan Olamilekan Adesola, Juliana Bunmi Adetunji, Temitayo Esther Adeyeoluwa, Usha Adiga, Qorinah Estiningtyas Sakilah Adnani, Abdelrahman Yousry Afify, Aanuoluwapo Adeyimika Afolabi, Muhammad Sohail Afzal, Saira Afzal, Suneth Buddhika Agampodi, Shahin Aghamiri, César Agostinis Sobrinho, Williams Agyemang-Duah, Bright Opoku Ahinkorah, Austin J Ahlstrom, Aqeel Ahmad, Danish Ahmad, Fuzail Ahmad, Muayyad M Ahmad, Noah Ahmad, Sajjad Ahmad, Ayman Ahmed, Haroon Ahmed, Luai A Ahmed, Mehrunnisha Sharif Ahmed, Meqdad Saleh Ahmed, Syed Anees Ahmed, Marjan Ajami, Mohammed Ahmed Akkaif, Ashley E Akrami, Hanadi Al Hamad, Syed Mahfuz Al Hasan, Zain Al Ta'ani, Yazan Al Thaher, Tariq A Alalwan, Ziyad Al-Aly, Khurshid Alam, Rasmieh Mustafa Al-amer, Amani Alansari, Fahmi Y. Al-Ashwal, Mohammed Albashtawy, Bezawit Abeje Alemayehu, Abdelazeem M Algammal, Khalid F Alhabib, Dari Alhuwail, Abid Ali, Endale Alemayehu Ali, Mohammad Daud Ali, Mohammed Usman Ali, Rafat Ali, Waad Ali, Sheikh Mohammad Alif, Yousef Alimohamadi, Samah W Al-Jabi, Mohamad Aljofan, Syed Mohamed Aljunid, Ahmad Alkhatib, Wael Almahmeed, Sabah Al-Marwani, Mahmoud A Alomari, Saleh A Alqahtani, Abdullah A Alqarni, Ahmad Alrawashdeh, Intima Alrimawi, Sahel Majed Alrousan, Najim Z Alshahrani, Zaid Altaany, Awais Altaf, Farrukh Jawad Alvi, Nelson Alvis-Guzman, Mohammad Al-Wardat, Yaser Mohammed Al-Worafi, Hany Aly, Safwat Aly, Karem H Alzoubi, Masoud Aman Mohammadi, Tewodros Getnet Amera, Sohrab Amiri, Hubert Amu, Dickson A Amugsi, Ganiyu Adeniyi Amusa, Roshan A Ananda, Robert Ancuceanu, Mohammed Tahir Ansari, Sumbul Ansari, Boluwatife Stephen Anuoluwa, Iyadunni Adesola Anuoluwa, Saeid Anvari, Sumadi Lukman Anwar, Anayochukwu Edward Anyasodor, Juan Pablo Arab, Jalal Arabloo, Mosab Arafat, Aleksandr Y Aravkin, Demelash Areda, Brhane Berhe Aregawi, Hidayat Arifin, Benedetta Armocida, Johan Ärnlöv, Mahwish Arooj, Amit Arora, Anton A Artamonov, Kurnia Dwi Artanti, Ashokan Arumugam, Mohammad Asghari-Jafarabadi, Tahira Ashraf, Bernard Kwadwo Yeboah Asiamah-Asare, Thomas Astell-Burt, Seyyed Shamsadin Athari, Prince Atorkey, Alok Atreya, Zaure Maratovna Aumoldaeva, Mamaru Ayenew Awoke, Adedapo Wasiu Awotidebe, Setognal Birara Aychiluhm, Amirali Azimi, Sadat Abdulla Aziz, Shahkaar Aziz, Ahmed Y. Azzam, Domenico Azzolino, Mina Babashahi, Giridhara Rathnaiah Babu, Alaa Aboelnour Badran, Nasser Bagheri, Ruhai Bai, Atif Amin Baig, Shankar M Bakkannavar, Senthilkumar Balakrishnan, Ovidiu Constantin Baltatu, Kiran Bam, Rajon Banik, Shirin Barati, Mainak Bardhan, Hiba Jawdat Barqawi, Simon Barquera, Amadou Barrow, Lingkan Barua, Mohammad-Mahdi Bastan, Saurav Basu, Reza Bayat, Mulat Tirfie Bayih, Nebiyou Simegnew Bayleyegn, Narasimha M Beeraka, Priyamadhaba Behera, Diana Fernanda Bejarano Ramirez, Umar Muhammad Bello, Luis Belo, Derrick A Bennett, Maria Bergami, Kidanemaryam Berhe, Abiye Assefa Berihun, Ajeet Singh Bhadoria, Neeraj Bhala, Ravi Bharadwaj, Nikha Bhardwaj, Pankaj Bhardwaj, Sonu Bhaskar, Ajay Nagesh Bhat, Priyadarshini Bhattacharjee, Gurjit Kaur Bhatti, Jasvinder Singh Bhatti, Cem Bilgin, Catherine Bisignano, Bijit Biswas, Bruno Bizzozero Peroni, Espen Bjertness, Tone Bjørge, Archith Boloor, Sri Harsha Boppana, Samuel Adolf Bosoka, Souad Bouaoud, Edward J Boyko, Dejana Braithwaite, Javier Brazo-Sayavera, Hermann Brenner, Dana Bryazka, Raffaele Bugiardini, Linh Phuong Bui, Yasser Bustanji, Nadeem Shafique Butt, Zahid A Butt, Mehtap Çakmak Barsbay, Daniela Calina, Luis Alberto Cámera, Luciana Aparecida Campos, Si Cao, Angelo

Capodici, Claudia Carletti, Andre F Carvalho, Márcia Carvalho, Monica Cattafesta, Maria Sofia Cattaruzza, Luca Cegolon, Francieli Cembranel, Ester Cerin, Achille Cernigliaro, Joshua Chadwick, Chiranjib Chakraborty, Eeshwar K Chandrasekar, Jung-Chen Chang, Vijay Kumar Chattu, Anis Ahmad Chaudhary, Akhilanand Chaurasia, An-Tian Chen, Haowei Chen, Nicholas WS Chew, Gerald Chi, Ritesh Chimoriya, Patrick R Ching, Abdulaal Chitheer, Dong-Woo Choi, Bryan Chong, Chean Lin Chong, Hitesh Chopra, Shivani Chopra, Hou In Chou, Sonali Gajanan Choudhari, Sheng-Chia Chung, Sunghyun Chung, Muhammad Chutiyami, Rebecca M Cogen, Alyssa Columbus, Nathalie Conrad, Michael H Criqui, Natalia Cruz-Martins, Alanna Gomes da Silva, Omid Dadras, Xiaochen Dai, Mayank Dalakoti, Emanuele D'Amico, Lalit Dandona, Rakhi Dandona, Lucio D'Anna, Pojsakorn Danpanichkul, Samuel Demissie Darcho, Reza Darvishi Cheshmeh Soltani, Nihar Ranjan Dash, Kairat Davletov, Azizallah Dehghan, Edgar Denova-Gutiérrez, Meseret Derbew Molla, Ismail Dergaa, Aragaw Tesfaw Desale, Vinoth Gnana Chellaiyan Devanbu, Devananda Devegowda, Arkadeep Dhali, Bibha Dhungel, Daniel Diaz, Monica Dinu, Thanh Chi Do, Camila Bruneli do Prado, Milad Dodangeh, Phidelia Theresa Doegah, Sushil Dohare, Klara Georgieva Dokova, Paul Narh Doku, Neda Dolatkhah, Mario D'Oria, Fariba - Dorostkar, Ojas Prakashbhai Doshi, Rajkumar Prakashbhai Doshi, Robert Kokou Dowou, Mi Du, Samuel C Dumith, Dorothea Dumuid, Bruce B Duncan, Sulagna Dutta, Alireza Ebrahimi, Kristina Edvardsson, Ashkan Eighaei Sedeh, Michael Ekholuenetale, Rabie Adel El Arab, Ibrahim Farahat El Bayoumy, Mohamed Ahmed Eladl, Said El-Ashker, Iffat Elbarazi, Islam Y Elgendy, Muhammed Elhadi, Ashraf A El-Metwally, Mohamed A Elmonem, Mohamed Hassan Elnaem, Randa Elsheikh, Chadi Eltaha, Theophilus I Emeto, Maysa Eslami, Natalia Fabin, Heidar Fadavian, Adeniyi Francis Fagbamigbe, Ildar Ravisovich Fakhradiyev, Seyed Nooreddin Faraji, Carla Sofia e Sá Farinha, MoezAlIslam Ezzat Mahmoud Faris, Pawan Sirwan Faris, Mohsen Farjoud Kouhanjani, Umar Farooque, Hossein Farrokhpour, Samuel Aanuoluwapo Fasusi, Patrick Fazeli, Timur Fazylov, Alireza Feizkhah, Ginenus Fekadu, Xiaoqi Feng, Rodrigo Fernandez-Jimenez, Nuno Ferreira, Nataliya A Foigt, Morenike Oluwatoyin Folayan, Artem Alekseevich Fomenkov, Roham Foroumadi, Celia Fortuna Rodrigues, Matteo Foschi, Kate Louise Francis, Richard Charles Franklin, Aleš Gába, Muktar A Gadanya, Abhay Motiramji Gaidhane, Yaseen Galali, Silvano Gallus, Balasankar Ganesan, Shivaprakash Gangachannaiah, Miglas Welay Gebregergis, Mesfin Gebrehiwot, Lemma Getacher, Molla Getie, Fataneh Ghadirian, Ramy Mohamed Ghazy, Artyom Urievich Gil, Tiffany K Gill, Richard F Gillum, Alem Abera Girmay, Mahaveer Golechha, Pouya Goleij, Alessandra C Goulart, Ayman Grada, Michal Grivna, Ashna Grover, Zhongyang Guan, Giovanni Guarducci, Mohammed Ibrahim Mohialdeen Gubari, Avirup Guha, Snigdha Gulati, Damitha Asanga Gunawardane, Zheng Guo, Bhawna Gupta, Rahul Gupta, Rajeev Gupta, Vipin Gupta, Roberth Steven Gutiérrez-Murillo, Jose Guzman-Esquivel, Najah R Hadi, Zahra Hadian, Nadia M Hamdy, Samer Hamidi, Asif Hanif, Nasrin Hanifi, Graeme J Hankey, Allie Haq, Josep Maria Haro, Faizul Hasan, Reza Hashempour, Mohammad Hashem Hashempur, Md Saquib Hasnain, Amr Hassan, Nageeb Hassan, Soheil Hassanipour, Afagh Hassanzade Rad, Rasmus J Havmoeller, Simon I Hay, Jeffrey J Hebert, Kamal Hezam, Yuta Hiraike, Mai Hoang, Ramesh Holla, Alamgir Hossain, Hassan Hosseinzadeh, Mihaela Hostiuc, Sorin Hostiuc, Zin Wai Htay, Mengsi Hu, Yifei Hu, Ayesha Humayun, Tsegaye Gebreyes Hundie, Mohamed Ibrahim Husseiny, Foziya Mohammed Hussien, Hong-Han Huynh, Bing-Fang Hwang, Ramzi Ibrahim, Anel Ibrayeva, Nayu Ikeda, Olayinka Stephen Ilesanmi, Irena M Ilic, Milena D Ilic, Leeberk Raja Inbaraj, Arit Inok, Khalid Iqbal, Md Sahidul Islam, Md. Fakrul Islam, Md. Rabiul Islam, Sheikh Mohammed Shariful Islam, Nahlah Elkudssiah Ismail, Hiroyasu Iso, Gaetano Isola, Mosimah Charles Ituka, Masao Iwagami, Chinwe Juliana Iwu-Jaja, Assefa N Iyasu, Louis Jacob, Shabbar Jaffar, Haitham Jahrami, Akhil Jain, Rajesh Jain, Ammar Abdulrahman Jairoun, Mihajlo Jakovljevic, Syed Sarmad Javaid, Sathish Kumar Jayapal, Shubha Jayaram, Felix K Jebasingh, Sun Ha Jee, Alelign Tasew Jema, Bijay

Mukesh Jeswani, Jost B Jonas, Nitin Joseph, Charity Ehimwenma Joshua, Jacek Jerzy Jozwiak, Mikk Jürisson, Billingsley Kaambwa, Ali Kabir, Vidya Kadashetti, Ashish Kumar Kakkar, Sanjay Kalra, Saddam Fuad Kanaan, Samuel Berchi Kankam, Arun R Kanmanthareddy, Kehinde Kazeem Kanmodi, Rami S Kantar, Debasish Kar, Mehrdad Karajizadeh, Paschalis Karakasis, Arman Karimi Behnagh, Sahand Karimzadhagh, Nicholas J Kassebaum, Joonas H Kauppila, Gbenga A Kayode, Shemsu Kedir, Dimitrios Kehagias, Ariz Keshwani, Emmanuelle Kesse-Guyot, Mohammad Keykhaei, Himanshu Khajuria, Pantea Khalili, Alireza Khalilian, Mohamed Khalis, Ajmal Khan, Maseer Khan, Md Abdullah Saeed Khan, Mohammad Jobair Khan, Moien AB Khan, Muhammad Shahzeb Khan, Nusrat Khan, Vishnu Khanal, Shaghayegh Khanmohammadi, Moawiah Mohammad Khatatbeh, Masoomeh Kheirkhah, Feriha Fatima Khidri, Manoj Khokhar, Atulya Aman Khosla, Sepehr Khosravi, Mahmood Khosrowjerdi, Helda Khusun, Gyu Ri Kim, Jihee Kim, Jinho Kim, Min Seo Kim, Yun Jin Kim, Ruth W Kimokoti, Adnan Kisa, Ladli Kishore, Shivakumar KM, Michail Kokkorakis, Farzad Kompani, Oleksii Korzh, Karel Kostev, Sindhura Lakshmi Koulmane Laxminarayana, Irene Akwo Kretchy, Chong-Han Kua, Barthelemy Kuate Defo, Mohammed Kuddus, Mukhtar Kulimbet, Vishnutheertha Kulkarni, G Anil Kumar, Vijay Kumar, Satyajit Kundu, Setor K Kunutsor, Om P Kurmi, Maria Dyah Kurniasari, Dian Kusuma, Ville Kytö, Carlo La Vecchia, Ben Lacey, Chandrakant Lahariya, Daphne Teck Ching Lai, Iván Landires, Bagher Larijani, Zohra S Lassi, Huyen Thi Thanh Le, Nhi Huu Hanh Le, Hye Ah Lee, Munjae Lee, Paul H Lee, Seung Won Lee, Wei-Chen Lee, An Li, Ming-Chieh Li, Wei Li, Yongze Li, Stephen S Lim, Jialing Lin, Queran Lin, Daniel Lindholm, Paulina A Lindstedt, Jue Liu, Justin Lo, José Francisco López-Gil, Stefan Lorkowski, Giancarlo Lucchetti, Alessandra Lugo, Angelina M Lutambi, Zheng Feei Ma, Javier A Magaña Gómez, Nastaran Maghbouli, Mehrdad Mahalleh, Nozad H. Mahmood, Azeem Majeed, Konstantinos Christos C. Makris, Elaheh Malakan Rad, Reza Malekzadeh, Kashish Malhotra, Ahmad Azam Malik, Iram Malik, Deborah Carvalho Malta, Abdullah A Mamun, Emmanuel Manu, Hamid Reza Marateb, Mirko Marino, Abdoljalal Marjani, Ramon Martinez-Piedra, Miquel Martorell, Winfried März, Sammer Marzouk, Soroush Masrouri, Yasith Mathangasinghe, Fernanda Penido Matozinhos, Thushara Matthias, Rita Mattiello, Andrea Maugeri, Mohsen Mazidi, Steven M McPhail, Enkeleint A Mechili, María Paz Medel Salas, Asim Mehmood, Kamran Mehrabani-Zeinabad, Tesfahun Mekene Meto, Hadush Negash Meles, Walter Mendoza, Ritesh G Menezes, Emiru Ayalew Mengistie, Sultan Ayoub Meo, Tomislav Mestrovic, Chamila Dinushi Kukulege Mettananda, Sachith Mettananda, Huanhuan Miao, Ted R Miller, Wai-kit Ming, Erkin M Mirrakhimov, Awoke Misganaw, Habtamu Mitiku, Madhukar Mittal, Jama Mohamed, Mona Gamal Mohamed, Nouh Saad Mohamed, Taj Mohammad, Sakineh Mohammad-Alizadeh-Charandabi, Abdollah Mohammadian-Hafshejani, Ibrahim Mohammadzadeh, Shafiu Mohammed, Ali H Mokdad, Lorenzo Monasta, Stefania Mondello, Mohammad Ali Moni, Sara Montazeri Namin, AmirAli Moodi Ghalibaf, Yousef Moradi, Shane Douglas Morrison, Rohith Motappa, Sumaira Mubarik, Francesk Mulita, Erin C Mullany, Yanjinlkham Munkhsaikhan, Efren Murillo-Zamora, Christopher J L Murray, Sani Musa, Ghulam Mustafa, Sathish Muthu, Julius C Mwita, Woojae Myung, Abdulrazaq Bidemi Nafiu, Gabriele Nagel, Ganesh R Naik, Hiten Naik, Gopal Nambi, Vinay Nangia, Shumaila Nargus, Gustavo G Nascimento, Mahmoud Nassar, Javaid Nauman, Zakira Naureen, Samidi Nirasha Kumari Navaratna, Nawsherwan , Biswa Prakash Nayak, Athare Nazri-Panjaki, Masoud Negahdary, Ionut Negoi, Ruxandra Irina Negoi, Soroush Nematollahi, Samata Nepal, Henok Biresaw Netsere, Marie Ng, Josephine W Ngunjiri, Dang Nguyen, Phat Tuan Nguyen, Phuong The Nguyen, Robina Khan Niazi, Luciano Nieddu, Mahdieh Niknam, Taxiarchis Konstantinos Nikolouzakis, Ali Nikoobar, Jan Rene Nkeck, Shuhei Nomura, Syed Toukir Ahmed Noor, Mamoona Noreen, Jean Jacques Noubiap, Mehran Nouri, Chisom Adaobi Nri-Ezedi, Fred Nugen, Virginia Nuñez-Samudio, Aqsha Nur, Felix Kwasi Nyande, Chimezie Igwegbe Nzoputam, Bogdan Oancea, Erin M

O'Connell, Ismail A Odetokun, Akinyemi O D Ofakunrin, James Odhiambo Oguta, In-Hwan Oh, Hassan Okati-Aliabad, Sylvester Reuben Okeke, Akinkunmi Paul Okekunle, Osaretin Christabel Okonji, Andrew T Olagunju, Oladotun Victor Olalusi, Tosin Abiola Olasehinde, Arão Belitardo Oliveira, Gláucia Maria Moraes Oliveira, Yinka Doris Oluwafemi, Hany A Omar, Ahmed Omar Bali, Nesredin Ahmed Omer, Sok King Ong, Michal Ordak, Alberto Ortiz, Augustus Osborne, Wael M S Osman, Adrian Otoiu, Abdu Oumer, Amel Ouyahia, Mayowa O Owolabi, Irene Amoakoh Owusu, Kolapo Oyebola, Tope Oyelade, Mahesh Padukudru P A, Alicia Padron-Monedero, Jagadish Rao Padubidri, Tamás Palicz, Sujogya Kumar Panda, Songhomitra Panda-Jonas, Anamika Pandey, Seithikurippu R Pandi-Perumal, Suman Pant, Shahina Pardhan, Utsav Parekh, Pragyan Paramita Parija, Romil R Parikh, Eun-Cheol Park, Roberto Passera, Jay Patel, Dimitrios Patoulias, Susan Paudel, Prince Peprah, Marcos Pereira, Norberto Perico, Simone Perna, Ionela-Roxana Petcu, Fanny Emily Petermann-Rocha, Hoang Nhat Pham, Tung Thanh Pham, Saeed Pirouzpanah, Roman V Polibin, Djordje S Popovic, Isabel Potani, Farzad Pourghazi, Akram Pourshams, Jalandhar Pradhan, Pranil Man Singh Pradhan, Manya Prasad, Akila Prashant, Elton Junio Sady Prates, Jagadeesh Puvvula, Ibrahim Qattea, Yanan Qiao, Venkatraman Radhakrishnan, Maja R Radojčić, Catalina Raggi, Fryad Majeed Rahman, Md. Mosfequr Rahman, Mohammad Hifz Ur Rahman, Mosiur Rahman, Muhammad Aziz Rahman, Mohammad Rahmanian, Vahid Rahmanian, Masoud Rahmati, Rajesh Kumar Rai, Ivano Raimondo, Jeffrey Pradeep Raj, Prashant Rajput, Mahmoud Mohammed Ramadan, Chitra Ramasamy, Shakthi Kumaran Ramasamy, Sheena Ramazanu, Kritika Rana, Chhabi Lal Ranabhat, Mithun Rao, Sowmya J Rao, Sina Rashedi, Mohammad-Mahdi Rashidi, Ashkan Rasouli-Saravani, Devarajan Rathish, Santosh Kumar Rauniyar, Ilari Rautalin, David Laith Rawaf, Salman Rawaf, Elrashdy M. Moustafa Mohamed Redwan, Sanika Rege, Aqeeb Ur Rehman, Ana Reis-Mendes, Giuseppe Remuzzi, Nazila Rezaei, Mohsen Rezaeian, Taeho Gregory Rhee, João Rocha Rocha-Gomes, Thales Philipe Rodrigues da Silva, Jefferson Antonio Buendia Rodriguez, Leonardo Roever, Peter Rohloff, Debby Syahru Romadlon, Moustaq Karim Khan Rony, Gholamreza Roshandel, Himanshu Sekhar Rout, Nitai Roy, Godfrey M Rwegerera, Aly M A Saad, Maha Mohamed Saber-Ayad, Leila Sabzmakan, Kabir P Sadarangani, Basema Ahmad Saddik, Masoumeh Sadeghi, Umar Saeed, Dominic Sagoe, Fatemeh Saheb Sharif-Askari, Amirhossein Sahebkar, Soumya Swaroop Sahoo, S. Mohammad Sajadi, Mirza Rizwan Sajid, Afeez Abolarinwa Salami, Luciane B Salaroli, Samreen Saleem, Mohamed A Saleh, Marwa Rashad Salem, Dauda Salihu, Sohrab Salimi, Abdallah M Samy, Milena M Santric-Milicevic, Tanmay Sarkar, Mohammad Sarmadi, Gargi Sachin Sarode, Sachin C Sarode, Michele Sassano, Jennifer Saulam, Monika Sawhney, Sonia Saxena, Ganesh Kumar Saya, Christophe Schinckus, Maria Inês Schmidt, Art Schuermans, Siddharthan Selvaraj, Ashenafi Kibret Sendekie, Pallav Sengupta, Yigit Can Senol, Subramanian Senthilkumaran, Sadaf G Sepanlou, Yashendra Sethi, Allen Seylani, Mahan Shafie, Sweni Shah, Samiah Shahid, Moyad Jamal Shahwan, Muhammad Aaqib Shamim, Mehran Shams-Beyranvand, Alfiya Shamsutdinova, Mohd Shanawaz, Mohammed Shannawaz, Medha Sharath, Amin Sharifan, Manoj Sharma, Ujjawal Sharma, Vishal Sharma, Fateme Sheida, Rekha Raghuveer Shenoy, Pavanchand H Shetty, Desalegn Shiferaw, Min-Jeong Shin, Mahsa Shirani Lapari, Rahman Shiri, Aminu Shittu, Sina Shool, Seyed Afshin Shorofi, Gambhir Shrestha, Rajan Shrestha, Kerem Shuval, Yafei Si, Nicole R S Sibuyi, Emmanuel Edwar Siddig, Mario Siervo, Diego Augusto Santos Silva, Luís Manuel Lopes Rodrigues Silva, Amit Singh, Baljinder Singh, Harmanjit Singh, Jasvinder A Singh, Kalpana Singh, Lucky Singh, Mitasha Singh, Poornima Suryanath Singh, Surjit Singh, Anna Aleksandrovna Skryabina, Amanda E Smith, Georgia Smith, Sameh S M Soliman, Soroush Soraneh, Michael Spartalis, Bahadar S Srichawla, Muhammad Haroon Stanikzai, Antonina V Starodubova, Kurt Straif, Pete Stubbs, Vetriselvan Subramaniyan, Muritala Odidi Suleiman Odidi, Aleksander Sulkowski, Anusha Sultan Meo, Zhong Sun, Sumam Sunny, Dev Ram

Sunuwar, Chandan Kumar Swain, Lukasz Szarpak, Sree Sudha T Y, Rafael Tabarés-Seisdedos, Seyyed Mohammad Tabatabaei, Ozra Tabatabaei Malazy, Seyed-Amir Tabatabaeizadeh, Shima Tabatabai, Celine Tabche, Mohammad Tabish, Jabeen Taiba, Mircea Tampa, Jacques Lukenze Tamuzi, Ker-Kan Tan, Manoj Tanwar, Saba Tariq, Nathan Y Tat, Mohamad-Hani Temsah, Reem Mohamad Hani Temsah, Masayuki Teramoto, Dufera Rikitu Terefa, Jay Tewari, Pugazhenthan Thangaraju, Rekha Thapar, Aravind Thavamani, Sathish Thirunavukkarasu, Joe Thomas, Sofonyas Abebaw Tiruneh, Tenaw Yimer Tiruye, Mariya Vladimirovna Titova, Krishna Tiwari, Sojit Tomo, Marcello Tonelli, Mathilde Touvier, Marcos Roberto Tovani-Palone, Khaled Trabelsi, Ngoc Ha Tran, Thang Huu Tran, Nguyen Tran Minh Duc, Domenico Trico, Thien Tan Tri Tai Truyen, Guesh Mebrahtom Tsegay, Munkhtuya Tumurkhuu, Sok Cin Tye, Aniefiok John Udoakang, Atta Ullah, Saeed Ullah, Shahid Ullah, Muhammad Umair, LAWAN UMAR, Umar Muhammad Umar, Brigid Unim, Dinesh Upadhya, Era Upadhyay, Jibrin Sammani Usman, Damla Ustunsoz, Masoud Vaezghasemi, Asokan Govindaraj Vaithinathan, Jef Van den Eynde, Joe Varghese, Tommi Juhani Vasankari, Siavash Vaziri, Balachandar Vellingiri, Narayanaswamy Venketasubramanian, Madhur Verma, Georgios-Ioannis Verras, Victor E Villalobos-Daniel, Sergey Konstantinovitch Vladimirov, Vasily Vlassov, Stein Emil Vollset, Rade Vukovic, Mohammad Wahiduzzaman, Cong Wang, Shu Wang, Xingxin Wang, Yanzhong Wang, Kosala Gayan Weerakoon, Fei-Long Wei, Anggi Lukman Wicaksana, Dakshitha Praneeth Wickramasinghe, Nuwan Darshana Wickramasinghe, Peter Willeit, Marcin W Wojewodzic, Qing Xia, Guangqin Xiao, Wanqing Xie, Suowen Xu, Xiaoyue Xu, Galal Yahya, Kazumasa Yamagishi, Yuichiro Yano, Haiqiang Yao, Amir Yarahmadi, Habib Yaribeygi, Pengpeng Ye, Subah Abderehim Yesuf, Dehui Yin, Dong Keon Yon, Naohiro Yonemoto, Chuanhua Yu, Chun-Wei Yuan, Deniz Yuce, Ismaeel Yunusa, Giulia Zamagni, Michael Zastrozhin, Mohammed G M Zeariya, Casper J P Zhang, Haijun Zhang, Jingya Zhang, Liqun Zhang, Xiaoyi Zhang, Zhiqiang Zhang, Hanqing Zhao, David X Zheng, Anthony Zhong, Claire Chenwen Zhong, Jiayan Zhou, Bin Zhu, Abzal Zhumagaliuly, Magdalena Zielińska, Osama A Zitoun, Ghazal Zoghi, Zhiyong Zou, Sa'ed H Zyoud, Emmanuela Gakidou**, Susan M Sawyer**, Peter S Azzopardi**

*Lead author
**Joint senior authors

Affiliations

Centre for Adolescent Health (J A Kerr PhD, K I Cini MClinEpi, K L Francis MBiostat, Prof S M Sawyer MD, P S Azzopardi PhD), Population Health Theme (Prof G C Patton MD), Murdoch Childrens Research Institute, Parkville, VIC, Australia; Department of Psychological Medicine (J A Kerr PhD), University of Otago, Christchurch, New Zealand; Department of Paediatrics (Prof G C Patton MD, P S Azzopardi PhD), Population Interventions Unit (B Dhungel DrPH), Department of Medicine (Prof F K Jebasingh DM), University of Melbourne, Melbourne, VIC, Australia; Department of Paediatrics (K I Cini MClinEpi), Centre for Adolescent Health (Prof S M Sawyer MD), University of Melbourne, Parkville, VIC, Australia; Department of Clinical Governance and Quality Improvement (Y H Abate MSc), Aleta Wondo General Hospital, Aleta Wondo, Ethiopia; Centre for Regenerative Medicine and Health (N Abbas PhD), Chinese Academy of Sciences, Hong Kong, China; Department of Neuroscience (N Abbas PhD), Department of Infectious Diseases and Public Health (G Fekadu PhD, Prof W Ming MD), City University of Hong Kong, Hong Kong, China; College of Pharmacy (A H A Abd Al Magied MSc), College of Medicine (S Dutta PhD),

Center for Medical and Bio-Allied Health Sciences Research (Prof M J Shahwan PhD), Ajman University, Ajman, United Arab Emirates (Prof N Hassan PhD); Department of Epidemiology (S Abd ElHafeez DrPH), Tropical Health Department (R M Ghazy PhD), Alexandria University, Alexandria, Egypt; Department of Tropical Medicine and Infectious Diseases (S Abd-Elsalam PhD), Tanta University, Tanta, Egypt; Minimally Invasive Surgery Research Center (A Abdollahi MD, A Kabir MD), Pars Advanced and Minimally Invasive Medical Manners Research Center (Y Alimohamadi PhD), Health Management and Economics Research Center (J Arabloo PhD), School of Medicine (M Bastan MD, M Dodangeh MD), Department of Medical Laboratory Sciences (F - Dorostkar PhD), Endocrine Research Center (A Karimi Behnagh MD), Department of Echocardiography (A Karimi Behnagh MD), Department of Obstetrics & Gynecology (P Khalili MD), Department of Midwifery and Reproductive Health (Prof M Kheirkhah PhD), Center for Technology and Innovation in Cardiovascular Informatics (S Shool MD), Iran University of Medical Sciences, Tehran, Iran; Department of Medicine (Prof M Abdoun PhD), University of Setif Algeria, Sétif, Algeria; Department of Health, Sétif, Algeria (Prof M Abdoun PhD); Community and Maternity Nursing Unit (D M Abdulah MPH), Department of Pathology and Microbiology (M S Ahmed PhD), University of Duhok, Duhok, Iraq; National Institute of Epidemiology (R Abdulkader PhD), Indian Council of Medical Research, Chennai, India; Department of Physiotherapy (A Abdullahi PhD, A W Awotidebe PhD, J S Usman PhD), Department of Community Medicine (Prof M A Gadanya MD), Bayero University Kano, Kano, Nigeria; Department of Physiotherapy (A Abdullahi PhD), Federal University Wukari, Wukari, Nigeria; School of Health Sciences (H M Abeywickrama PhD), Niigata University, Niigata, Japan; Midwifery Department (A Abie MSc), College of Medicine and Health Sciences (M Adane PhD, H B Netsere MSc), Department of Midwifery (B A Alemayehu MSc), Department of Public Health Nutrition (M T Bayih MSc), Department of Adult Health Nursing (E A Mengistie MSc), Bahir Dar University, Bahir Dar, Ethiopia; Department of Community Medicine (Prof O Abiodun MPH), Babcock University, Ilishan-Remo, Nigeria; Cardiovascular Research Center (S Abohashem MPH), Department of Orthopaedic Surgery (A Ebrahimi MD), Department of Medicine (D X Zheng MD), Massachusetts General Hospital, Boston, MA, USA (M Kim MD); Department of Radiology (S Abohashem MPH), Department of Pediatrics (S Aly MD), Division of Cardiovascular Medicine (G Chi MD), Division of Cardiology (I Y Elgendy MD), Department of Medicine (M Kokkorakis BSc), T.H. Chan School of Public Health (P M S Pradhan MD), Division of Global Health Equity (P Rohloff MD), Joslin Diabetes Center (S Tye PhD), Harvard Medical School (A Zhong MA), Harvard University, Boston, MA, USA; Department of Anesthesiology (Prof D Abtahi MD, S Salimi MD), Department of Biotechnology (S Aghamiri PhD), National Nutrition and Food Technology Research Institute (M Ajami PhD), Department of Food Science and Nutrition (M Aman Mohammadi PhD), Psychiatric Nursing and Management Department (F Ghadirian PhD), Food Technology Research (Z Hadian PhD), Department of Health Management, Policy and Economics (R Hashempour PhD), Research Institute for Gastroenterology and Liver (S Karimzadhagh MD), Research Institute for Endocrine Sciences (S Masrouri MD), Skull Base Research Center (I Mohammadzadeh MD), Research Center for Social Determinants of Health (M Niknam PhD), Social Determinants of Health Research Center (A Nikoobar BSc, M Rashidi MD), Student Research Committee (M Rahmanian MD), Department of Immunology (A Rasouli-Saravani PhD), Department of Medical Education (S Tabatabai PhD), Shahid Beheshti University of Medical Sciences, Tehran, Iran; Department of Nursing (H Abualruz PhD), Al Zaytoonah University of Jordan, Amman, Jordan; Department of Pharmacology and Toxicology (B Abubakar PhD), Department of Veterinary Public Health and Preventive Medicine (A Shittu MSc), Usmanu Danfodiyo University, Sokoto, Sokoto, Nigeria; Clinical Science Department (Prof M O Folayan PhD), Department of Biochemistry and Nutrition (K Oyebola PhD), Nigerian Institute of Medical

Research, Lagos, Nigeria (B Abubakar PhD); Department of Clinical Sciences (Prof E Abu-Gharbieh PhD, Prof M M Ramadan PhD), Department of Pharmacy Practice and Pharmacotherapeutics (Prof K H Alzoubi PhD, Prof H A Omar PhD), Department of Physiotherapy (A Arumugam PhD), Clinical Sciences Department (H J Barqawi MPhil, N R Dash MD, Prof M M Saber-Ayad PhD), Department of Basic Biomedical Sciences (Prof Y Bustanji PhD), Department of Basic Medical Sciences (M A Eladl PhD), College of Medicine (Prof B A Saddik PhD, Prof M A Saleh PhD), Sharjah Institute of Medical Sciences (F Saheb Sharif-Askari PhD), Department of Medicinal Chemistry (S S M Soliman PhD), University of Sharjah, Sharjah, United Arab Emirates; Department of Biopharmaceutics and Clinical Pharmacy (Prof E Abu-Gharbieh PhD), College of Pharmacy (Prof S Aburuz PhD), School of Nursing (Prof M M Ahmad PhD), University of Jordan, Amman, Jordan; Medical Research Center (H J Abukhadijah MPH), Department of Geriatric and Long Term Care (H Al Hamad MD), Rumailah Hospital (H Al Hamad MD), Department of Surgery (A Alansari MD), Research Department (K Singh PhD), Hamad Medical Corporation, Doha, Qatar; Institute of Community and Public Health (Prof N M Abu-Rmeileh PhD), Birzeit University, Ramallah, Palestine; Department of Pharmacology and Therapeutics (Prof S Aburuz PhD), Institute of Public Health (Prof L A Ahmed PhD, I Elbarazi DrPH, Prof M Grivna PhD), Family Medicine Department (M A Khan MSc), College of Medicine and Health Sciences (J Nauman PhD), United Arab Emirates University, Al Ain, United Arab Emirates; Department of Biochemistry and Molecular Medicine (A Abu-Zaid PhD), College of Medicine (Prof O Baltatu PhD), College of Pharmacy (R M H Temsah PharmD), Alfaisal University, Riyadh, Saudi Arabia; College of Graduate Health Sciences (A Abu-Zaid PhD), University of Tennessee, Memphis, TN, USA; Department of Diagnostic and Interventional Radiology (L C Adams PhD), Technical University of Munich, Munich, Germany; School of Medicine (I Y Addo PhD), School of Architecture, Design, and Planning (Prof T Astell-Burt PhD), Concord Clinical School (R Chimoriya PhD), University of Sydney, Sydney, NSW, Australia (S R Okeke PhD); Centre for Social Research in Health (I Y Addo PhD, S R Okeke PhD), School of Population Health (X Feng PhD, Prof B A Saddik PhD, X Xu PhD), International Centre for Future Health Systems (J Lin PhD), School of Risk and Actuarial Studies (Y Si PhD), The George Institute for Global Health (P Ye PhD), University of New South Wales, Sydney, NSW, Australia; Department of Immunology (K A Adedokun MSc), Department of Medicine (M Nassar PhD), University at Buffalo, Buffalo, NY, USA; Translational Research Team (N A Adegoke PhD), Melanoma Institute Australia, The University of Sydney, Sydney Australia, Sydney, NSW, Australia; Department of Veterinary Medicine and Surgery (R Adesola DVM), University of Missouri, Columbia, MO, USA; Department of Biochemistry (J B Adetunji PhD), Osun State University, Osogbo, Nigeria; Department of Pharmacology and Therapeutics (T E Adeyeoluwa PhD), Department of Environmental and Occupational Health (B S Anuoluwa MPH), Department of Microbiology (I A Anuoluwa PhD, Y Oluwafemi PhD), Department of Biosciences and Biotechnology (A J Udoakang PhD), University of Medical Sciences, Ondo, Ondo, Nigeria; Department of Veterinary Medicine (T E Adeyeoluwa PhD), Department of Epidemiology and Medical Statistics (A F Fagbamigbe PhD), College of Medicine (A P Okekunle PhD), Department of Medicine (O V Olalusi MD, Prof M O Owolabi DrM), University of Ibadan, Ibadan, Nigeria; Apollo Institute Of Medical Sciences & Research Chittoor (Prof U Adiga PhD), Apollo Hospital, Chittoor, India; Department of Public Health (Q Adnani PhD), Universitas Padjadjaran (Padjadjaran University), Bandung, Indonesia; Department of Internal Medicine (A Y Afify MD), Department of Neurology (Prof A Hassan MD), Cairo University, Cairo, Egypt; Technical Services Directorate (A A Afolabi MPH), MSI Nigeria Reproductive Choices, Abuja, Nigeria; Department of Life Sciences (M S Afzal PhD, Prof M Umair PhD), University of Management and Technology, Lahore, Pakistan; Department of Community Medicine (Prof S Afzal PhD), King Edward Memorial Hospital, Lahore, Pakistan; Department of Public Health (Prof S Afzal PhD), Public Health

Institute, Lahore, Pakistan; Department of New Initiatives (Prof S B Agampodi MD), International Vaccine Institute, Seoul, South Korea; Health Research and Innovation Sciences Center (C Agostinis Sobrinho PhD), Klaipeda University, Klaipeda, Lithuania; SPRINT Sport physical activity and health research & innovation center (C Agostinis Sobrinho PhD), Sport Physical Activity and Health Research & Innovation Center (SPRINT) (Prof L M R Silva PhD), Polytechnic Institute of Guarda, Guarda, Portugal; Department of Public Health Sciences (W Agyemang-Duah PhD), Queen's University, Kingston, ON, Canada; School of Public Health (B O Ahinkorah MPhil), School of Nursing and Midwifery (M Chutiyami PhD), Discipline of Physiotherapy (P Stubbs PhD), School of Biomedical Engineering (N Tran MD), University of Technology Sydney, Sydney, NSW, Australia; Institute for Health Metrics and Evaluation (A J Ahlstrom MSc, N Ahmad BS, A Y Aravkin PhD, C Bisignano MPH, D Bryazka BA, R M Cogen BA, X Dai PhD, Prof L Dandona MD, Prof R Dandona PhD, Prof S I Hay FMedSci, N J Kassebaum MD, Prof S S Lim PhD, P A Lindstedt MPH, J Lo BA, T Mestrovic PhD, Prof A H Mokdad PhD, E C Mullany BA, Prof C J L Murray DPhil, M Ng PhD, E M O'Connell BA, C Raggi MS, A E Smith MPA, G Smith MS, Prof S E Vollset DrPH, C Yuan PhD, Prof E Gakidou PhD), Department of Applied Mathematics (A J Ahlstrom MSc, A Y Aravkin PhD), Department of Health Metrics Sciences, School of Medicine (A Y Aravkin PhD, X Dai PhD, Prof R Dandona PhD, Prof S I Hay FMedSci, N J Kassebaum MD, Prof S S Lim PhD, A Misganaw PhD, Prof A H Mokdad PhD, Prof C J L Murray DPhil, Prof S E Vollset DrPH, Prof E Gakidou PhD), School of Medicine (E J Boyko MD), Department of Anesthesiology & Pain Medicine (N J Kassebaum MD), University of Washington, Seattle, WA, USA; College of Medicine (A Ahmad PhD, M Tabish MPharm), Shaqra University, Shaqra, Saudi Arabia; School of Medicine and Psychology (D Ahmad PhD), Australian National University, Canberra, ACT, Australia; Public Health Foundation of India, Gandhinagar, India (D Ahmad PhD); College of Applied Sciences (Prof F Ahmad PhD), Almaarefa University, Riyadh, Saudi Arabia; Department of Health and Biological Sciences (S Ahmad PhD), Abasyn University, Peshawar, Pakistan; Department of Natural Sciences (S Ahmad PhD), Gilbert and Rose-Marie Chagoury School of Medicine (Prof L Roever PhD), Lebanese American University, Beirut, Lebanon; Institute of Endemic Diseases (A Ahmed MSc), Unit of Basic Medical Sciences (E E Siddig MD), University of Khartoum, Khartoum, Sudan; Swiss Tropical and Public Health Institute (A Ahmed MSc), University of Basel, Basel, Switzerland; Department of Biosciences (H Ahmed PhD), COMSATS Institute of Information Technology, Islamabad, Pakistan; College of Nursing (M S Ahmed MSc), Majmaah University, Al Majmaah, Saudi Arabia; Brody School of Medicine (S Ahmed PhD), East Carolina University, Greenville, NC, USA; Department of Cardiology (M Akkaif PhD), Fudan University, Shanghai, China; Chicago College of Osteopathic Medicine (A E Akrami BS), Midwestern University, Downers Grove, IL, USA; Feinberg School of Medicine (A E Akrami BS, A Keshwani MPH), Medical Scientist Training Program (S Marzouk MA), Department of Preventive Medicine (M Teramoto MD), Northwestern University, Chicago, IL, USA; Division of Public Health Sciences (S Al Hasan PhD), Department of Research and Development (Z Al-Aly MD), Department of Surgery (C Wang PhD), Washington University in St. Louis, St. Louis, MO, USA; Jordan Medical Association, Amman, Jordan (Z Al Ta'ani MD); Faculty of Pharmacy (Y Al Thaher PhD), Philadelphia University, Amman, Jordan; School of Pharmacy (Y Al Thaher PhD), Cardiff University, Cardiff, UK; Department of Biology (T A Alalwan PhD), College of Health and Sport Sciences (A G Vaithinathan MSc), University of Bahrain, Zallaq, Bahrain; Clinical Epidemiology Center (Z Al-Aly MD), US Department of Veterans Affairs (VA), St. Louis, MO, USA; Murdoch Business School (K Alam PhD), Murdoch University, Perth, WA, Australia; School of Nursing (R M Al-amer PhD), Department of Basic Sciences (Z Altaany PhD), Department of Basic Medical Sciences (Prof M M Khatatbeh PhD), Yarmouk University, Irbid, Jordan; School of Nursing and Midwifery (R M Al-amer PhD), Department of Engineering (G R Naik PhD),

Translational Health Research Institute (K Rana PhD), Western Sydney University, Sydney, NSW, Australia; Department of Clinical Pharmacy (F Y Al-Ashwal PhD), Al-Ayen Iraqi University, Thi-Qar, Iraq; Department of Clinical Pharmacy and Pharmacy Practice (F Y Al-Ashwal PhD), University of Science and Technology, Sana'a, Yemen; Department of Community and Mental Health (Prof M Albashtawy PhD), Al al-Bayt University, Mafraq, Jordan; Department of Bacteriology, Immunology, and Mycology (Prof A M Algammal PhD), Suez Canal University, Ismailia, Egypt; Department of Cardiac Sciences (Prof K F Alhabib MD), Department of Physiology (Prof S A Meo PhD), University Diabetes Center (A Sultan Meo MPH), Pediatric Intensive Care Unit (Prof M Temsah MD), King Saud University, Riyadh, Saudi Arabia; Information Science Department (D Alhuwail PhD), Kuwait University, Sabah Alsalem University City, Kuwait; Health Informatics Unit and Geohealth Lab (D Alhuwail PhD), Dasman Diabetes Institute, Dasman, Kuwait; Department of Zoology (A Ali PhD), Abdul Wali Khan University Mardan, Mardan, Pakistan; Department of Public Health and Primary Care (E Ali MSc), Department of Cardiovascular Sciences (N Conrad PhD, A Schuermans BSc, J Van den Eynde BSc), Faculty of Medicine (A Schuermans BSc), Katholieke Universiteit Leuven, Leuven, Belgium; Department of Pharmacy (M Ali PhD), Department of Pharmacy, Mohammed Al-Mana College for Medical Sciences, Dammam, Saudi Arabia; Department of Medical Rehabilitation (Physiotherapy) (M U Ali PhD), University of Maiduguri, Maiduguri, Nigeria; Department of Rehabilitation Sciences (M U Ali PhD, M Khan MPH, J S Usman PhD), Hong Kong Polytechnic University, Hong Kong, China; Department of Biosciences (R Ali PhD), Centre for Interdisciplinary Research in Basic Sciences (T Mohammad PhD), Jamia Millia Islamia, New Delhi, India; Department of Geography (W Ali PhD), Sultan Qaboos University, Muscat, Oman; Institute of Health and Wellbeing (S M Alif PhD), Federation University Australia, Melbourne, VIC, Australia; School of Public Health and Preventive Medicine (S M Alif PhD, Prof M Asghari-Jafarabadi PhD), Department of Epidemiology and Preventive Medicine (M A Awoke MPH), Monash Centre for Health Research and Implementation (MCHRI) (S A Tiruneh MPH), Monash University, Melbourne, VIC, Australia; Department of Clinical and Community Pharmacy (Prof S W Al-Jabi PhD, Prof S H Zyoud PhD), An-Najah National University, Nablus, Palestine; Department of Biomedical Sciences (M Aljofan PhD), Nazarbayev University, Astana, Kazakhstan; Department of Public Health and Community Medicine (Prof S M Aljunid PhD), International Medical University, Kuala Lumpur, Malaysia; International Centre for Casemix and Clinical Coding (Prof S M Aljunid PhD), National University of Malaysia, Bandar Tun Razak, Malaysia; College of Life Sciences (Prof A Alkhatib PhD), Birmingham City University, Birmingham, UK; Department of Cardiology, Heart, Vascular, and Thoracic Institute (Prof W Almahmeed MD), Cleveland Clinic Abu Dhabi, Abu Dhabi, United Arab Emirates; College of Medicine and Health Sciences Academic Programs (Prof W Almahmeed MD), Department of Biology (W M S Osman PhD), Khalifa University, Abu Dhabi, United Arab Emirates; Independent Consultant, Amman, Jordan (S Al-Marwani MSc); Department of Physical Therapy and Rehabilitation Sciences (Prof M A Alomari PhD), Department of Rehabilitation Sciences and Physical Therapy (Prof M A Alomari PhD), Department of Allied Medical Sciences (A Alrawashdeh PhD), Department of Rehabilitation Sciences (M Al-Wardat PhD), Department of Clinical Pharmacy (Prof K H Alzoubi PhD), Jordan University of Science and Technology, Irbid, Jordan; Liver, Digestive, and Lifestyle Health Research Section (S A Alqahtani MD), King Faisal Specialist Hospital & Research Center, Riyadh, Saudi Arabia; Division of Gastroenterology and Hepatology (S A Alqahtani MD), Weill Cornell Medicine, New York, NY, USA; Faculty of Medical Rehabilitation Sciences (A A Alqarni PhD), Department of Family and Community Medicine (Prof N S Butt PhD), Rabigh Faculty of Medicine (Prof A Malik PhD), King Abdulaziz University, Jeddah, Saudi Arabia; Department of Nursing (I Alrimawi PhD), Georgetown University, Washington, DC, USA; Macro-Fiscal Policy Department (S M Alrousan PhD),

Ministry of Finance, Dubai, United Arab Emirates; Department of Family and Community Medicine (N Z Alshahrani MD), University of Jeddah, Jeddah, Saudi Arabia; Institute of Molecular Biology and Biotechnology (A Altaf PhD, S Shahid PhD), University Institute of Public Health (F J Alvi MPH, S Nargus PhD), University College of Medicine & Dentistry (Prof M Arooj PhD), Research Centre for Health Sciences (RCHS) (S Shahid PhD), The University of Lahore, Lahore, Pakistan; Research Group in Health Economics (Prof N Alvis-Guzman PhD), Universidad de Cartagena (University of Cartagena), Cartagena, Colombia; Research Group in Hospital Management and Health Policies (Prof N Alvis-Guzman PhD), Universidad de la Costa (University of the Coast), Barranquilla, Colombia; Department of Medical Sciences (Prof Y M Al-Worafi PhD), Azal University for Human Development, Sana'a, Yemen; Department of Clinical Sciences (Prof Y M Al-Worafi PhD), University of Science and Technology of Fujairah, Fujairah, United Arab Emirates; Department of Pediatrics (Prof H Aly MD), Bariatric and Metabolic Institute (R Foroumadi MD), Department of Thoracic Surgery (S Gulati MD), Cleveland Clinic, Cleveland, OH, USA; Department of Pediatric Cardiology (S Aly MD), Boston Children's Hospital, Boston, MA, USA; Department of Public Health (T G Amera MPH, A Oumer PhD), Dire Dawa University, Dire Dawa, Ethiopia; Spiritual Health Research Center (S Amiri PhD), Baqiyatallah University of Medical Sciences, Tehran, Iran; Department of Population and Behavioural Sciences (H Amu PhD, E Manu PhD), Department of Epidemiology and Biostatistics (S A Bosoka MPhil, R K Dowou MPhil), Institute of Health Research (P T Doegah PhD), Department of Nursing (F K Nyande PhD), University of Health and Allied Sciences, Ho, Ghana; Department of Maternal and Child Wellbeing (D A Amugsi PhD), African Population and Health Research Center, Nairobi, Kenya; Department of Medicine (G A Amusa MD), Department of Pediatrics (A O D Ofakunrin MD), University of Jos, Jos, Nigeria; Department of Internal Medicine (G A Amusa MD), Department of Pediatrics (A O D Ofakunrin MD), Jos University Teaching Hospital, Jos, Nigeria; Department of General Medicine (R A Ananda MD), Eastern Health, Box Hill, VIC, Australia; Faculty of Pharmacy (Prof R Ancuceanu PhD), Department of Internal Medicine (M Hostiuc PhD), Department of Legal Medicine and Bioethics (Prof S Hostiuc PhD), Department of General Surgery (I Negoi PhD), Department of Anatomy and Embryology (R I Negoi PhD), Department of Dermatology (M Tampa PhD), Carol Davila University of Medicine and Pharmacy, Bucharest, Romania; School of Pharmacy (M T Ansari PhD), University of Nottingham Malaysia, Semenyih, Malaysia; Department of Physiotherapy, School of Allied Health Sciences (S Ansari PhD), Galgotias University, Greater Noida, India; Regenerative Medicine, Organ Procurement and Transplantation Multi-disciplinary Center (S Anvari MD), Pediatric Diseases Research Center (R Bayat MSc, A Hassanzade Rad PhD), Department of Social Medicine and Epidemiology (A Feizkhah MD), Gastrointestinal and Liver Diseases Research Center (S Hassanipour PhD, F Sheida MD), Caspian Digestive Disease Research Center (S Hassanipour PhD), Clinical Research Development Unit of Poursina Hospital (S Karimzadhagh MD), Guilan University of Medical Sciences, Rasht, Iran; Department of Surgery (S Anwar PhD), Department of Medical Surgical Nursing (A L Wicaksana MS), Gadjah Mada University, Yogyakarta, Indonesia; Rural Health Research Institute (A E Anyasodor PhD, S B Aychiluhm MPH), Charles Sturt University, Orange, NSW, Australia; Division of Gastroenterology, Hepatology, and Nutrition (J Arab MD), Division of Infectious Diseases (P R Ching MD), Virginia Commonwealth University, Richmond, VA, USA; Gastroenterology Department (J Arab MD), Pontifical Catholic University of Chile, Santiago, Chile; College of Pharmacy (M Arafat PhD), Al Ain University, Abu Dhabi, United Arab Emirates; College of Art and Science (D Areda PhD), Ottawa University, Surprise, AZ, USA; School of Life Sciences (D Areda PhD), Arizona State University, Tempe, AZ, USA; College of Medicine and Health Sciences (B B Aregawi PhD), Department of Midwifery (M W Gebregergis MSc), Department of Medical Laboratory Sciences (H N Meles MSc), Adigrat University,

Adigrat, Ethiopia; Faculty of Nursing (H Arifin MKep), Division of Epidemiology (K D Artanti DrPH), Universitas Airlangga (Airlangga University), Surabaya, Indonesia; Department of Cardiovascular, Endocrine-Metabolic Diseases and Aging (B Armocida MD), Istituto Superiore di Sanità (ISS), Rome, Italy; Department of Neurobiology, Care Sciences and Society (Prof J Ärnlöv PhD), Department of Molecular Medicine and Surgery (Prof J H Kauppila MD), Karolinska Institute, Stockholm, Sweden; School of Health and Social Studies (Prof J Ärnlöv PhD), Dalarna University, Falun, Sweden; School of Health Sciences (A Arora PhD), Western Sydney University, Campbelltown, NSW, Australia; Disciple of Child and Adolescent Health (A Arora PhD), University of Sydney, Westmead, NSW, Australia; Institute for Biomedical Problems (A A Artamonov PhD), K.A. Timiryazev Institute of Plant Physiology (M V Titova PhD), Russian Academy of Sciences, Moscow, Russia; Department of Physiotherapy (A Arumugam PhD), Department of Forensic Medicine and Toxicology (S M Bakkannavar MD), Department of Pharmacology, Kasturba Medical College (S Gangachannaiah MD), Kasturba Medical College, Mangalore (R Holla MD), Kasturba Medical College Manipal (J P Raj DMPhil), Kasturba Medical College Mangalore (M Rao MD), Department of Pharmacology (R R Shenoy PhD), Kasturba Medical College (D Upadhya PhD), Manipal Academy of Higher Education, Manipal, India; Cabrini Research (Prof M Asghari-Jafarabadi PhD), Cabrini Health, Malvern, VIC, Australia; Pioneer Journal of Biostatistics and Medical Research (PJBMR), Pakistan, Pakistan (T Ashraf PhD); Deakin Health Economics/School of Health and Social Development (B K Y Asiamah-Asare PhD), Institute for Physical Activity and Nutrition (S Paudel PhD), Deakin University, Melbourne, VIC, Australia; Department of Immunology (S Athari PhD), Department of Critical Care and Emergency Nursing (N Hanifi PhD), Zanjan University of Medical Sciences, Zanjan, Iran; School of Medicine and Public Health (P Atorkey PhD), University of Newcastle, Newcastle, NSW, Australia; Australian College of Applied Professions (P Atorkey PhD), Australian College of Applied Professions, Sydney, NSW, Australia; Department of Forensic Medicine (A Atreya MD), Department of Community Medicine (S Nepal MD), Lumbini Medical College, Palpa, Nepal; кафедра (Z M Aumoldaeva MSc), Al Farabi Kazakh National University, Almaty, Kazakhstan; School of Nursing and Public Health (A W Awotidebe PhD), University of KwaZulu-Natal, Durban, South Africa; Institute of Public Health (S B Aychiluhm MPH), Department of Biochemistry (M Derbew Molla MSc), School of Nursing (H B Netsere MSc), Department of Clinical Pharmacy (A K Sendekie MSc), University of Gondar, Gondar, Ethiopia; Department of Medicine (A Azimi MD), Non-communicable Diseases Research Center (M Bastan MD, M Keykhaei MD, M Rashidi MD, N Rezaei MD), Iranian Research Center for HIV/AIDS (IRCHA) (O Dadras PhD), School of Medicine (H Farrokhpour MD, S Khanmohammadi MD), Department of Surgery (R Foroumadi MD), Students' Scientific Research Center (SSRC) (M Keykhaei MD), Children's Medical Center (Prof F Kompani MD), Endocrinology and Metabolism Research Institute (Prof B Larijani MD, O Tabatabaei Malazy PhD), Department of Physical Medicine and Rehabilitation (N Maghbouli MD), Department of Pediatric Cardiology (Prof E Malakan Rad MD), Digestive Diseases Research Institute (Prof R Malekzadeh MD, Prof A Pourshams MD, S G Sepanlou MD), Department of Cardiovascular Diseases (S Nematollahi MD), Department of Neurology (M Shafie MD), Sina Hospital (A Sharifan PharmD), Sina Trauma and Surgery Research Center (S Shool MD), Tehran University of Medical Sciences, Tehran, Iran (M Mahalleh MD); Department of Anesthesia (S A Aziz PhD), Cihan University-Sulaimaniya, Sulaymaniyah, Iraq; University of Sulaimani (S A Aziz PhD), Department of Basic Sciences, Sulaymaniyah, Iraq; Institute of Biotechnology and Genetic Engineering (S Aziz MS), The University of Agriculture, Peshawar, Pakistan; ASIDE Healthcare, Lewes, DE, USA (A Azzam MD); Faculty of Medicine (A Azzam MD), October 6 University, 6th of October City, Egypt; Geriatric Unit (D Azzolino PhD), Fondazione IRCCS Ca' Granda Ospedale Maggiore Policlinico, Milan, Italy; Nutrition Research Center (M Babashahi PhD),

Department of Pathology (S Faraji PhD), School of Medicine (M Farjoud Kouhanjani MD), Epilepsy Research Center (M Farjoud Kouhanjani MD), Research Center for Traditional Medicine and History of Medicine (Prof M Hashempur PhD), Trauma Research Center (M Karajizadeh PhD), Non-communicable Disease Research Center (Prof R Malekzadeh MD, S G Sepanlou MD), Health Policy Research Center (M Nouri PhD), Department of Medicine (A Yarahmadi PhD), Shiraz University of Medical Sciences, Shiraz, Iran; Department of Population Medicine (Prof G Babu PhD), Department of Rehabilitation Sciences (S F Kanaan PhD), Qatar University, Doha, Qatar; Department of Clinical Pathology (Prof A A Badran MD), Anatomy and Embryology (M A Eladl PhD), Department of Cardiology (Prof M M Ramadan PhD), Faculty of Pharmacy (Prof M A Saleh PhD), Mansoura University, Mansoura, Egypt; Microbiology Department (Prof A A Badran MD), Horus University Egypt, Damietta, Egypt; Health Research Institute (Prof N Bagheri PhD), University of Canberra, Canberra, ACT, Australia; School of Public Affairs (R Bai MD), Nanjing University of Information Science and Technology, Nanjing, China; International Medical School (A A Baig PhD), Management and Science University, Alam, Malaysia; Division of Biological Sciences (S Balakrishnan PhD), Tamil Nadu State Council for Science and Technology, Chennai, India; Center of Innovation, Technology and Education (CITE) (Prof O Baltatu PhD), Anhembi Morumbi University, São José dos Campos, Brazil; Department of Medicine (K Bam MPH), Department of Anatomy and Developmental Biology (Y Mathangasinghe PhD), Monash University, Clayton, VIC, Australia; Maternal and Child Health Division (R Banik MS), International Centre for Diarrhoeal Disease Research, Bangladesh (icddr,b), Dhaka, Bangladesh; Department of Anatomy (S Barati PhD), Saveh University of Medical Sciences, Saveh, Iran; Miller School of Medicine (M Bardhan MD), University of Miami, Miami, FL, USA; Center for Nutrition and Health Research (E Denova-Gutiérrez DSc), National Institute of Public Health, Cuernavaca, Mexico (S Barquera PhD); Department of Public and Environmental Health (A Barrow MPH), University of The Gambia, Banjul, The Gambia; Department of Epidemiology (A Barrow MPH, D Braithwaite PhD), University of Florida, Gainesville, FL, USA; Department of Non-communicable Diseases (L Barua MPH), Bangladesh University of Health Sciences, Dhaka, Bangladesh; Department of Academics (S Basu MD), Indian Institute of Public Health, Gurgaon, India; Department of Surgery (N S Bayleyegn MD), Department of Epidemiology (D Shiferaw MPH), Jimma University, Jimma, Ethiopia; Human Anatomy and Histology (Prof N Beeraka PhD), Department of Epidemiology and Evidence-Based Medicine (R V Polibin PhD), I.M. Sechenov First Moscow State Medical University, Moscow, Russia; Department of Community Medicine and Family Medicine (P Behera MD), All India Institute of Medical Sciences, Bhubaneswar, India; Transplant and Hepatobiliary Surgery Service (D F Bejarano Ramirez MSc), Hospital Universitorio Fundación Santa Fe de Bogotá, Bogota, Colombia; Subdirectorate of Clinical Studies and Clinical Epidemiology (D F Bejarano Ramirez MSc), Hospital Universitorio Fundación Santa Fe de Bogotá, Bogotá, Colombia; Department of Physiotherapy and Paramedicine (U M Bello PhD), Glasgow Caledonian University, Glasgow, UK; Department of Biological Sciences (Prof L Belo PhD), Research Unit on Applied Molecular Biosciences (UCIBIO) (Prof L Belo PhD), Associated Laboratory for Green Chemistry (LAQV) (M Carvalho PhD), Institute for Research and Innovation in Health (i3S) (Prof N Cruz-Martins PhD), Applied Molecular Biosciences Unit (Prof C Fortuna Rodrigues PhD), Faculty of Engineering (Prof C Fortuna Rodrigues PhD), Department of Chemical Sciences (A Reis-Mendes PhD), Faculty of Medicine (J R Rocha-Gomes MD), University of Porto, Porto, Portugal; Nuffield Department of Population Health (D A Bennett PhD, B Lacey DPhil), University of Oxford, Oxford, UK; Dipartimento di Scienze Mediche e Chirurgiche (M Bergami PhD), Department of Medical and Surgical Sciences (Prof R Bugiardini MD, M Sassano MD), University of Bologna, Bologna, Italy; Department of Nutrition and Dietetics (K Berhe MPH), Mekelle University, Mekelle, Ethiopia; School of Public Health (A A Berihun MA), Department of

Anesthesia and Critical Care Medicine (S Boppana MD), Department of Biostatistics (A Columbus MS), Department of Epidemiology (T G Hundie MD), Cardiovascular Pathology Department (M Shirani Lapari MD), Department of International Health (H Zhang MS), Johns Hopkins University, Baltimore, MD, USA; Department of Community and Family Medicine (A S Bhadoria MD), All India Institute of Medical Sciences, Rishikesh, India; Community Health Department (A S Bhadoria MD), University of South Wales, South Wales, UK; Institute of Applied Health Research (N Bhala PhD), University of Nottingham, Nottingham, UK; Institute of Applied Health Research (N Bhala PhD, K Malhotra MBBS), Department of Metabolism and Systems Science (S Tariq PhD), University of Birmingham, Birmingham, UK; Department of Medicine (R Bharadwaj PhD), Department of Neurology (B S Srichawla MD), University of Massachusetts Medical School, Worcester, MA, USA; Department of Anatomy (N Bhardwaj MD), Department of Community Medicine and Family Medicine (Prof P Bhardwaj MD), School of Public Health (Prof P Bhardwaj MD), Department of Biochemistry (M Khokhar PhD, S Tomo MD), Department of Pharmacology (M Shamim MBBS, S Singh MD, K Tiwari MBBS), All India Institute of Medical Sciences, Jodhpur, India; Global Health Neurology Lab (S Bhaskar MD), NSW Brain Clot Bank, Sydney, NSW, Australia; Division of Cerebrovascular Medicine and Neurology (S Bhaskar MD), National Cerebral and Cardiovascular Center, Suita, Japan; Department of General Medicine (A N Bhat MD), Department of Internal Medicine (A Boloor MD), Department of Community Medicine (N Joseph MD, R Motappa MD, R Thapar MD), Department of Forensic Medicine and Toxicology (Prof J Padubidri MD, P H Shetty MD), Manipal Academy of Higher Education, Mangalore, India; The Translational and Clinical Institute (P Bhattacharjee MD), Newcastle University, Newcastle upon Tyne, UK; Department of Clinical Medicine (P Bhattacharjee MD), Cambridge University Hospitals NHS Foundation Trust, Cambridge, UK; Department of Medical Lab Technology (Prof G K Bhatti PhD), University Centre for Research and Development (S Kalra DM), Chandigarh University, Mohali, India; Department of Human Genetics and Molecular Medicine (Prof J S Bhatti PhD, U Sharma PhD), Department of Microbiology (A Singh PhD), Department of Biochemistry (B Singh PhD), Department of Zoology (B Vellingiri PhD), Central University of Punjab, Bathinda, India; Department of Radiology (C Bilgin MD), Neurovascular Research Laboratory (C Bilgin MD), Mayo Clinic College of Medicine, Rochester, MN, USA; Department of Community Medicine and Family Medicine (B Biswas MD), Department of Pharmacology (S T Y MD), All India Institute of Medical Sciences, Deoghar, India; Health and Social Research Center (B Bizzozero Peroni PhD), Universidad de Castilla-La Mancha, Cuenca, Spain; Higher Institute of Physical Education (B Bizzozero Peroni PhD), Universidad de la República, Rivera, Uruguay; Department of Community Medicine and Global Health (Prof E Bjertness PhD), University of Oslo, Oslo, Norway; Department of Global Public Health and Primary Care (Prof T Bjørge PhD), Department of Psychosocial Science (Prof D Sagoe PhD), University of Bergen, Bergen, Norway; Department of Research (M W Wojewodzic PhD), Cancer Registry of Norway, Oslo, Norway (Prof T Bjørge PhD); Disease Surveillance Department (S A Bosoka MPhil), Ghana Health Service, Ho, Ghana; Department of Medicine (Prof S Bouaoud DrPH), Faculty of Medicine (Prof A Ouyahia PhD), University Ferhat Abbas of Setif, Sétif, Algeria; Department of Epidemiology and Preventive Medicine (Prof S Bouaoud DrPH), University Hospital Saadna Abdenour, Sétif, Algeria; General Medicine Service (E J Boyko MD), Department of Veterans Affairs, Seattle, WA, USA; Cancer Population Sciences Program (D Braithwaite PhD), University of Florida Health Cancer Center, Gainesville, FL, USA; Department of Sports and Computer Science (J Brazo-Sayavera PhD), Universidad Pablo de Olavid (Pablo de Olavide University), Seville, Spain; Division of Clinical Epidemiology and Aging Research (Prof H Brenner MD), German Cancer Research Center, Heidelberg, Germany; College of Health Sciences (L P Bui PhD, T T Pham PhD), VinUniversity, Hanoi, Viet Nam; Research Advancement

Consortium in Health, Hanoi, Viet Nam (L P Bui PhD, T T Pham PhD); School of Pharmacy (Prof Y Bustanji PhD), The University of Jordan, Amman, Jordan; School of Public Health Sciences (Z A Butt PhD, O A Zitoun MD), University of Waterloo, Waterloo, ON, Canada; Al Shifa School of Public Health (Z A Butt PhD), Al Shifa Trust Eye Hospital, Rawalpindi, Pakistan; Faculty of Health Sciences Healthcare Management Department (M Çakmak Barsbay PhD), Ankara University, Ankara, Turkiye; Department of Clinical Pharmacy (Prof D Calina PhD), University of Medicine and Pharmacy of Craiova, Romania, Craiova, Romania; Department of Internal and Geriatric Medicine (Prof L A Cámera MD), Hospital Italiano de Buenos Aires (Italian Hospital of Buenos Aires), Buenos Aires, Argentina; Board of Directors (Prof L A Cámera MD), Argentine Society of Medicine, Buenos Aires, Argentina; Institute of Biomedical Engineering (Prof L A Campos PhD), Anhembi Morumbi University, Sao Jose dos Campos, Brazil; Department of Biomedical Engineering (Prof L A Campos PhD), São José dos Campos Technology Park, São José dos Campos, Brazil; Department of Anesthesiology (S Cao MD), Third Xiangya Hospital of Central South University, Changsha, China; Unit of Hygiene and Public Health (A Capodici MD), Romagna Local Health Authority, Forlì-Cesena, Italy; Interdisciplinary Research Center for Health Science (A Capodici MD), Sant'Anna School of Advanced Studies, Pisa, Italy; Clinical Epidemiology and Public Health Research Unit (C Carletti MSc, L Monasta DSc, G Zamagni MSc), Burlo Garofolo Institute for Maternal and Child Health, Trieste, Italy; IMPInstitute for Mental and Physical Health and Clinical Translation (IMPACT) (A F Carvalho MD), Deakin University, Geelong, VIC, Australia; Faculty of Health Sciences (M Carvalho PhD), University Fernando Pessoa, Porto, Portugal; Graduate Program in Nutrition and Health (M Cattafesta PhD), Center for Health Sciences (C B do Prado MSc), Federal University of Espírito Santo, Vitória, Brazil; Department of Public Health and Infectious Diseases (M S Cattaruzza PhD), La Sapienza University, Rome, Italy; Department of Medical, Surgical, and Health Sciences (Prof L Cegolon PhD, Prof M D'Oria MD), University of Trieste, Trieste, Italy; Public Health Unit (Prof L Cegolon PhD), University Health Agency Giuliano-Isontina (ASUGI), Trieste, Italy; Department of Nutrition (Prof F Cembranel DSc), Department of Physical Education (Prof D A S Silva PhD), Federal University of Santa Catarina, Florianópolis, Brazil; Mary MacKillop Institute for Health Research (Prof E Cerin PhD), Australian Catholic University, Melbourne, VIC, Australia; School of Public Health (Prof E Cerin PhD, C J P Zhang PhD), Department of Medicine (H Chou MSc), University of Hong Kong, Hong Kong, China; Regional Epidemiological Observatory Department (A Cernigliaro MSc), Sicilian Regional Health Authority, Palermo, Italy; Non-communicable Diseases Division (J Chadwick MD), National Institute of Epidemiology, Chennai, India; Department of Biotechnology (Prof C Chakraborty PhD), Adamas University, Kolkata, India; Institute for Skeletal Aging & Orthopedic Surgery (Prof C Chakraborty PhD), Hallym University, Chuncheon, South Korea; Department of Anesthesiology and Perioperative Medicine (E K Chandrasekar MD), School of Medicine (Prof S Xu PhD), University of Rochester, Rochester, NY, USA; College of Medicine (J Chang PhD), National Taiwan University, Taipei, Taiwan; Department of Nursing (J Chang PhD), National Taiwan University Hospital, Taipei, Taiwan; Temerty Faculty of Medicine (V Chattu MD), University of Toronto, Toronto, ON, Canada; Department of Community Medicine (V Chattu MD), Datta Meghe Institute of Medical Sciences, Sawangi, India; Department of Biology (A A Chaudhary PhD), Al-Imam Mohammad Ibn Saud Islamic University, Riyadh, Saudi Arabia; Department of Oral Medicine and Radiology (Prof A Chaurasia MD), Internal Medicine Department (J Tewari MBBS), King George's Medical University, Lucknow, India; Fuwai Hospital (A Chen PhD), Chinese Academy of Medical Sciences, Beijing, China; Clinical Research Center (H Chen PhD), Zhujiang Hospital of Southern Medical University, Guangzhou, China; Yong Loo Lin School of Medicine (N W Chew MD, M Ng PhD, Prof N Venketasubramanian MSc), Department of Medicine (B Chong MBBS), Cardiovascular Metabolic

Translational Research Program (M Dalakoti MPH), Saw Swee Hock School of Public Health (S Ramazanu PhD), Department of Surgery (K Tan PhD), National University of Singapore, Singapore, Singapore; Concord Institute of Academic Surgery (R Chimoriya PhD), Sydney Local Health District, Sydney, NSW, Australia; Iraq Field Epidemiology Training Program (I-FETP) (A Chitheer MD), Ministry of Health, Baghdad, Iraq; Cancer Big Data Center (D Choi PhD), National Cancer Center, Goyang, South Korea; RIPAS Hospital (C Chong MD), University of Brunei Darussalam, Bandar Seri Begawan, Brunei; Centre for Research Impact & Outcome (H Chopra PhD), Chitkara University, Rajpura, India; Department of Biosciences (S Chopra MPH), Center for Global Health Research (Prof A Sahebkar PhD), Saveetha Dental College and Hospitals (S Selvaraj PhD, M Tovani-Palone PhD), Centre for Global Health Research (M Tabish MPharm), Saveetha University, Chennai, India; Department of Community Medicine (Prof S G Choudhari MD), Jawaharlal Nehru Medical College, Wardha, India; Department of Health Informatics (S Chung PhD), Institute of Epidemiology and Health Care (J Kim MSc), Division of Medicine (T Oyelade PhD), University College London, London, UK; Health Data Research UK, London, UK (S Chung PhD); Department of Health Behavior (S Chung MPH), Department of Biomedical Engineering (M Negahdary PhD), Texas A&M University, College Station, TX, USA; Department of Family Medicine and Public Health (Prof M H Criqui MD), University of California San Diego, La Jolla, CA, USA; Department of Diagnostic and Therapeutic Technologies (Prof N Cruz-Martins PhD), Cooperativa de Ensino Superior Politécnico e Universitário (Polytechnic and University Higher Education Cooperative), Vila Nova de Famalicão, Portugal; School of Nursing (A da Silva PhD), Department of Maternal-Child Nursing and Public Health (Prof D C Malta PhD, Prof F P Matozinhos PhD, E J S Prates BS), Vaccination Research Observatory (T Rodrigues da Silva PhD), Federal University of Minas Gerais, Belo Horizonte, Brazil; Research Center for Child Psychiatry (O Dadras PhD), University of Turku, Turku, Finland; Department of Public Health and Primary Care (M Dalakoti MPH, Prof P Willeit PhD), University of Cambridge, Cambridge, UK; Department of Medical and Surgical Sciences and Advanced Technologies "GF Ingrassia" (Prof E D'Amico MD, A Maugeri PhD), Department of General Surgery and Medical-Surgical Specialties (Prof G Isola PhD), University of Catania, Catania, Italy; Public Health Foundation of India, Gurugram, India (Prof L Dandona MD, Prof R Dandona PhD, G Kumar PhD, A Pandey PhD); Department of Brain Sciences (L D'Anna PhD), WHO Collaborating Centre for Public Health Education and Training (Q Lin MPH, D L Rawaf MD), Department of Primary Care and Public Health (Prof A Majeed MD, Prof S Rawaf MD, C Tabche MSc), School of Public Health (Prof S Saxena MD), Imperial College London, London, UK; Department of Internal Medicine (P Danpanichkul MD), Texas Tech University, Lubbock, TX, USA; Department of Public Health (S D Darcho MPH), Department of Medical Laboratory Science (H Mitiku MSc), Department of Nursing (N Omer MSc), School of Public Health (A Oumer PhD), Haramaya University, Harar, Ethiopia (H Mitiku MSc); Department of Environmental Health (R Darvishi Cheshmeh Soltani PhD), Arak University of Medical Sciences, Arak, Iran; Population Health Research Center (Prof K Davletov PhD), Director of the Scientific and Technological Park (I R Fakhradiyev PhD), Laboratory of Experimental Medicine (T Fazylov MD), Science and Technology Park (A Ibrayeva PhD), Research and Publication Activity Division (M Kulimbet MSc), Science Department (A Shamsutdinova MD), Atchabarov Scientific-Research Institute of Fundamental and Applied Medicine (A Zhumagaliuly MD), Kazakh National Medical University, Almaty, Kazakhstan; Department of Epidemiology and Community Medicine (A Dehghan PhD), Non-Communicable Diseases Research Center (NCDRC), Fasa, Iran; Department of Biological Sciences (I Dergaa PhD), University of Manouba, Manouba, Tunisia; Department of Social Sciences (I Dergaa PhD), University of Jendouba, El Kef, Tunisia; Department of Public Health (A T Desale MPH, S A Tiruneh MPH), Debre Tabor University, Debre Tabor, Ethiopia; Chettinad Hospital & Research Institute (Prof V Devanbu

MD), Chettinad Academy of Research and Education, Chennai, India; JSS Medical College Department of Biochemistry (D Devegowda PhD), Department of Biochemistry (Prof A Prashant PhD), Jagadguru Sri Shivarathreeswara University, Mysuru, India; Sheffield Teaching Hospitals NHS Foundation Trust, Sheffield, UK (A Dhali MBBS); Faculty of Science (Prof D Diaz PhD), National Autonomous University of Mexico, Mexico City, Mexico; Department of Experimental and Clinical Medicine (M Dinu PhD), University of Florence, Florence, Italy; Department of Medicine (T C Do MD), Pham Ngoc Thach University of Medicine, Ho Chi Minh, Viet Nam; Department of Public Health (S Dohare MD, A Mehmood PhD), Epidemiology Program (M Khan MD), College of Nursing and Health Sciences (M Shanawaz MD), College of Public Health and Tropical Medicine (J Varghese PhD), Jazan University, Jazan, Saudi Arabia; Department of Social Medicine and Health Care Organisation (Prof K G Dokova PhD), Medical University of Varna, Varna, Bulgaria; Department of Psychology (P N Doku PhD), American University in Dubai, Dubai, United Arab Emirates; University of Cape Coast, Cape Coast, Ghana (P N Doku PhD); Faculty of Medicine (N Dolatkhah PhD), Social Determinants of Health Research Center (Prof S Mohammad-Alizadeh-Charandabi PhD), Midwifery Department (Prof S Mohammad-Alizadeh-Charandabi PhD), Molecular Medicine Research Center (S Pirouzpanah PhD), Tabriz University of Medical Sciences, Tabriz, Iran; Cardio-Thoraco-Vascular Department (Prof M D'Oria MD), Azienda Sanitaria Universitaria Giuliano Isontina, Trieste, Italy; Independent Consultant, South Plainfield, NJ, USA (O P Doshi MS); Department of Cardiology (R P Doshi MD), Hackettstown Medical Center, Hackettstown, NJ, USA; Newton Medical Center, Sparta, NJ, USA (R P Doshi MD); School of Stomatology (M Du PhD), Department of Periodontology (M Du PhD), Shandong Provincial Hospital Affiliated to Shandong First Medical University (M Hu MD), Cheeloo College of Medicine (M Hu MD), Department of Epidemiology (Y Qiao MD), Shandong University, Jinan, China; Postgraduate Program in Health Sciences (S C Dumith PhD), Federal University of Rio Grande, Rio Grande, Brazil; Allied Health and Human Performance (D Dumuid PhD), Department of Allied Health and Human Performance (T Y Tiruye PhD), University of South Australia, Adelaide, SA, Australia; Postgraduate Program in Epidemiology (Prof B B Duncan MD, Prof M I Schmidt MD), Department of Social Medicine (R Mattiello PhD), Federal University of Rio Grande do Sul, Porto Alegre, Brazil; School of Nursing and Midwifery (K Edvardsson PhD), La Trobe University, Bundoora, VIC, Australia; Isenberg School of Management (A Eighaei Sedeh MD), University of Massachusetts Amherst, Amherst, MA, USA; Faculty of Science and Health (M Ekholuenetale PhD), University of Portsmouth, Hampshire, UK; Almoosa College of Health Sciences, Al Ahsa, Saudi Arabia (R A El Arab PhD); Department of Public Health and Community Medicine (Prof I F El Bayoumy DrPH), Tanta University, Tanta city, Egypt; School of Public Health (Prof I F El Bayoumy DrPH), Texila American University, Guyana, Guyana; Deanship of Preparatory Year and Supporting Studies (Prof S El-Ashker PhD), Division of Forensic Medicine (Prof R G Menezes MD), Imam Abdulrahman Bin Faisal University, Dammam, Saudi Arabia; Division of Cardiovascular Medicine (I Y Elgendy MD), University of Kentucky, Lexington, KY, USA; Faculty of Medicine (M Elhadi MD), University of Tripoli, Tripoli, Libya; Houston Methodist Hospital, Houston, TX, USA (M Elhadi MD); College of Public Health and Health Informatics (Prof A A El-Metwally PhD), King Saud bin Abdulaziz University for Health Sciences, Riyadh, Saudi Arabia; Medical Genomics Research Department (Prof M Umair PhD), King Abdullah International Medical Research Center, Riyadh, Saudi Arabia (Prof A A El-Metwally PhD); Egypt Center for Research and Regenerative Medicine (ECRRM), Cairo, Egypt (M A Elmonem PhD); School of Pharmacy and Pharmaceutical Sciences (M Elnaem PhD), Ulster University, Coleraine, UK; Deanery of Biomedical Sciences (R Elsheikh MD), Global Health Governance Programme (J Patel BSc), College of Medicine and Veterinary Medicine (G Verras MSc), University of Edinburgh, Edinburgh, UK; Department of Pediatrics (C Eltaha MD), University of Texas,

Dallas, TX, USA; Department of Public Health and Tropical Medicine (T I Emeto PhD), College of Medicine, Dentistry and Public Health (Prof R C Franklin PhD), James Cook University, Townsville, QLD, Australia; Gastroenterology and hepatology department (M Eslami MD), Department of Radiology (F Nugen PhD), Department of Cardiovascular Medicine (H Pham MD), Department of Physiology and Biomedical Engineering (F Pourghazi MD), Mayo Clinic, Rochester, MN, USA; Department of Biomedical Sciences (N Fabin MD), Humanitas University, Milan, Italy; IRCCS Humanitas Research Hospital, Milan, Italy (N Fabin MD); Department of Electrical and Computer Engineering (H Fadavian MSc), Tarbiat Modares University, Tehran, Iran; Research Centre for Healthcare and Community (A F Fagbamigbe PhD), Faculty of Health and Life Sciences (O P Kurmi PhD), Centre for Healthcare and Communities (S Pant MPH), Coventry University, Coventry, UK; Department of Medicine (I R Fakhradiyev PhD), Department of Health Policy and Management (Prof J Kim PhD), Korea University, Seoul, South Korea (Prof M Shin PhD); Environmental Statistics Unit (C S e Farinha PhD), National Institute of Statistics, Lisbon, Portugal; Ecological Economics and Environmental Management (C S e Farinha PhD), NOVA University of Lisbon, Lisbon, Portugal; Department of Clinical Nutrition and Dietetics (Prof M E M Faris PhD), Applied Science Private University, Amman, Jordan; Department of Biology (P S Faris PhD), Department of Food Technology (Y Galali ResM), Salahaddin University-Erbil, Erbil, Iraq; Department of Biology (P S Faris PhD), Department of Nutrition and Dietetics (Y Galali ResM), Cihan University-Erbil, Erbil, Iraq; Department of Family Medicine (U Farooque MD), Luton & Dunstable University Hospital, Luton, UK; Endocrinology and Metabolism Research Institute (H Farrokhpour MD), Department of Epidemiology (S Khanmohammadi MD, S Rashedi MD), Department of Epidemiology and Biostatistics (S Khosravi MD), Non-Communicable Diseases Research Center (NCDRC), Tehran, Iran; Department of Crop Science and Biotechnology (S A Fasusi PhD), Dankook University,, Cheonan, South Korea; Department of Biology and Medicine (P Fazeli MSc), School of Public Health (A Haq BS), Brown University, Providence, RI, USA; Department of Pharmacy (G Fekadu PhD), Department of Public Health (D R Terefa MSc), Wollega University, Nekemte, Ethiopia; National Institute of Environmental Health (X Feng PhD), National Center for Chronic and Noncommunicable Disease Control and Prevention (P Ye PhD), Chinese Center for Disease Control and Prevention, Beijing, China; Cardiovascular Health and Imaging Laboratory (R Fernandez-Jimenez PhD), Centro Nacional de Investigaciones Cardiovasculares (CNIC), Madrid, Spain; Department of Cardiology (R Fernandez-Jimenez PhD), Hospital Clinico San Carlos, IdISSC, Madrid, Spain; Department of Social Sciences (Prof N Ferreira PhD), University of Nicosia, Nicosia, Cyprus; Institute of Gerontology (N A Foigt PhD), National Academy of Medical Sciences of Ukraine, Kyiv, Ukraine; Department of Child Dental Health (Prof M O Folayan PhD), Obafemi Awolowo University, Ile-Ife, Nigeria; Department of Cell Biology and Biotechnology (A A Fomenkov PhD), K.A. Timiryazev Institute of Plant Physiology, Moscow, Russia; Department of Neuroscience (M Foschi MD), Multiple Sclerosis Research Center, Ravenna, Italy; Department of Biotechnological and Applied Clincial Sciences (M Foschi MD), University of L'Aquila, L'Aquila, Italy; Faculty of Physical Culture (A Gába PhD), Palacký University Olomouc, Olomouc, Czech Republic; Department of Community Medicine (Prof M A Gadanya MD), Aminu Kano Teaching Hospital, Kano, Nigeria; Department of Community Medicine (Prof A M Gaidhane MD), Datta Meghe Institute of Medical Sciences, Wardha, India; Department of Medical Epidemiology (S Gallus PhD), Department of Environmental Health Sciences (A Lugo PhD), Mario Negri Institute for Pharmacological Research, Milan, Italy; School of Public Health (B Ganesan PhD), Institute of Health &Managment, Australia @ Powered by Arizona State University,USA, Melbourne, VIC, Australia; Department of Environmental Health (M Gebrehiwot DSc), Department of Public Health (F M Hussien MPH, F M Hussien MPH), Wollo University, Dessie, Ethiopia; Department of Public Health (L Getacher

PhD), Debre Berhan University, Debre Berhan, Ethiopia; Department of Medical Laboratory Science (M Getie MSc), Addis Ababa University, Addis Ababa, Ethiopia; Family and Community Medicine Department (R M Ghazy PhD), King Khalid University, Abha, Saudi Arabia; Country Office (A U Gil PhD), World Health Organization (WHO), Astana, Kazakhstan; Adelaide Medical School (T K Gill PhD), Robinson Research Institute (Z S Lassi PhD), University of Adelaide, Adelaide, SA, Australia; Division of General Internal Medicine (R F Gillum MD), Department of Community and Family Medicine (R F Gillum MD), Howard University, Washington, DC, USA; Department of Nursing (A A Girmay MSc, A N Iyasu MSc, G M Tsegay MSc), Aksum University, Aksum, Ethiopia; Department of Health Systems and Policy Research (Prof M Golechha PhD), Indian Institute of Public Health, Gandhinagar, India; Department of Genetics (P Goleij MSc), Sana Institute of Higher Education, Sari, Iran; Universal Scientific Education and Research Network (USERN) (P Goleij MSc), Department of Infectious Disease (Prof S Vaziri MD), Kermanshah University of Medical Sciences, Kermanshah, Iran; Department of Epidemiology (Prof A C Goulart PhD), Universidade de São Paulo (University of São Paulo), São Paulo, Brazil; Department of Dermatology (A Grada MD), Harrington Heart and Vascular Institute (A Guha MD), Department of Neonatology (I Qattea MD), Department of Pediatrics (A Thavamani MD), Division of Pediatric Gastroenterology (A Thavamani MD), Case Western Reserve University, Cleveland, OH, USA; Department of Public Health and Preventive Medicine (Prof M Grivna PhD), Charles University, Prague, Czech Republic; Department of Endocrinology (A Grover MD), National Institutes of Health, Bethesda, MD, USA; School of Population Health (Z Guan MPH, Prof M Siervo PhD), School of Public Health (T R Miller PhD), School of Pharmacy (A K Sendekie MSc), Curtin University, Perth, WA, Australia; Post Graduate School of Public Health (G Guarducci MD), University of Siena, Siena, Italy; Department of Clinical Science (M I M Gubari PhD), University Of Sulaimani, Sulaimani, Iraq; Division of Cardiovascular Medicine (A Guha MD), Ohio State University, Columbus, OH, USA; Department of Community Medicine (D A Gunawardane MD, S N K Navaratna MD), University of Peradeniya, Kandy, Sri Lanka; Divison of Epidemology (Z Guo PhD), Vanderbilt University Medical Center, Nashville, TN, USA; Department of Public Health (B Gupta PhD), Torrens University Australia, Melbourne, VIC, Australia; Department of Cardiology (R Gupta MBBS), Lehigh Valley Health Network, Allentown, PA, USA; Department of Preventive Cardiology & Medicine (Prof R Gupta MD), Eternal Heart Care Centre & Research Institute, Jaipur, India; Department of Medicine (Prof R Gupta MD), Mahatma Gandhi University Medical Sciences, Jaipur, India; Department of Anthropology (V Gupta PhD), Deemed University, Delhi, India; Department of Biomedical Gerontology (R S Gutiérrez-Murillo PhD), Pontifical Catholic University of Rio Grande do Sul, Porto Alegre, Brazil; Research Unit (J Guzman-Esquivel PhD), Mexican Institute of Social Security, Colima, Mexico; Department of Clinical Pharmacology and Medicine (Prof N R Hadi PhD), University of Kufa, Najaf, Iraq; Food and Nutrition Research Institute, Tehran, Iran (Z Hadian PhD); Biochemistry Department (Prof N M Hamdy PhD), Department of Entomology (A M Samy PhD), Medical Ain Shams Research Institute (MASRI) (A M Samy PhD), Ain Shams University, Cairo, Egypt; School of Health and Environmental Studies (Prof S Hamidi DrPH), Hamdan Bin Mohammed Smart University, Dubai, United Arab Emirates; Sakarya University, Turkey, Sakarya, Turkiye (A Hanif PhD); Centre for Neuromuscular and Neurological Disorders (Perron Institute) (Prof G J Hankey MD), The University of Western Australia, Perth, WA, Australia; Stroke Research Centre (Prof G J Hankey MD), Perron Institute for Neurological and Translational Science, Perth, WA, Australia; Research Unit (J M Haro MD), Parc Sanitari Sant Joan de Deu, Barcelona, Spain; Department of Mental Health (J M Haro MD), Carlos III Health Institute (Prof R Tabarés-Seisdedos PhD), Biomedical Research Networking Center for Mental Health Network (CiberSAM), Madrid, Spain; Faculty of Nursing (F Hasan PhD, D S Romadlon PhD), Chulalongkorn University, Bangkok, Thailand; Department

of Pharmacy (Prof M S Hasnain PhD), Marwadi University, Rajkot, India; Skaane University Hospital (R J Havmoeller PhD), Skaane County Council, Malmö, Sweden; Faculty of Kinesiology (Prof J J Hebert PhD), University of New Brunswick, Fredericton, NB, Canada; School of Allied Health (Prof J J Hebert PhD), Murdoch University, Murdoch, WA, Australia; Department of Microbiology (K Hezam PhD), Taiz University, Taiz, Yemen; School of Medicine (K Hezam PhD), Nankai University, Tianjin, China; Graduate School of Medicine (Y Hiraike PhD), Department of Global Health Policy (Prof S Nomura PhD, S K Rauniyar PhD), University of Tokyo, Tokyo, Japan; School of Medicine (M Hoang MD), Faculty of Medicine (T T Truyen MD), Tan Tao University, Long An, Viet Nam; Department of Physics (A Hossain PhD), Department of Population Science and Human Resource Development (Prof M Rahman PhD, Prof M Rahman DrPH), University of Rajshahi, Rajshahi, Bangladesh; School of Health and Society (H Hosseinzadeh PhD), University of Wollongong, Wollongong, NSW, Australia; Department of Clinical Legal Medicine (Prof S Hostiuc PhD), National Institute of Legal Medicine Mina Minovici, Bucharest, Romania; Health and Environmental Risk Division (Z Htay PhD), National Institute for Environmental Studies, Japan, Tsukuba, Japan; Maternal Care and Child Health Department (Y Hu PhD), Department of Neurosurgery (S Wang MD), Capital Medical University, Beijing, China; Department of Public Health and Community Medicine (Prof A Humayun PhD), Shaikh Zayed Postgraduate Medical Institute, Lahore, Pakistan; Artur Riggs Diabetes & Metabolism Research Institute (Prof M I Husseiny PhD), Cancer Prevention and Research Institute, Duarte, CA, USA; International Master Program for Translational Science (H Huynh BS), Nursing School (M Kurniasari PhD), School of Nursing (A L Wicaksana MS), Taipei Medical University, Taipei, Taiwan; Department of Occupational Safety and Health (Prof B Hwang PhD), China Medical University, Taiwan, Taichung, Taiwan; Department of Occupational Therapy (Prof B Hwang PhD), Asia University, Taiwan, Taichung, Taiwan; Department of Cardiovascular Medicine (R Ibrahim MD), Mayo Clinic, Phoenix, AZ, USA; Center for Nutritional Epidemiology and Policy Research (N Ikeda PhD), National Institutes of Biomedical Innovation, Health and Nutrition, Osaka, Japan; West Africa RCC (O S Ilesanmi PhD), Africa Centre for Disease Control and Prevention, Abuja, Nigeria; Department of Community Medicine (O S Ilesanmi PhD), Department of Neurology (O V Olalusi MD), Department of Medicine (Prof M O Owolabi DrM), Department of Oral and Maxillofacial Surgery (A A Salami BDS), University College Hospital, Ibadan, Ibadan, Nigeria; Faculty of Medicine (I M Ilic PhD, Prof M M Santric-Milicevic PhD), School of Public Health and Health Management (Prof M M Santric-Milicevic PhD), School of Medicine (R Vukovic PhD), University of Belgrade, Belgrade, Serbia; Faculty of Medical Sciences (Prof M D Ilic PhD), University of Kragujevac, Kragujevac, Serbia; Department of Health Research (L R Inbaraj MD), ICMR National Institute for Research in Tuberculosis, Chennai, India; Faculty of Health and Life Sciences (A Inok PhD), University of Exeter, Exeter, UK; Department of Epidemiological Methods and Etiological Research (K Iqbal DrPH), Leibniz Institute for Prevention Research and Epidemiology, Bremen, Germany; Department of Human Nutrition (K Iqbal DrPH), Khyber Medical University, Peshawar, Pakistan; Research and Publication Department (M Islam MSc), World Health Organization (WHO), Dhaka, Bangladesh; Department of Statistics (M Islam BSc), Shahjalal University of Science and Technology, Sylhet, Sylhet, Bangladesh; School of Pharmacy (M Islam PhD), BRAC University, Dhaka, Bangladesh; Institute for Physical Activity and Nutrition (Prof S Islam PhD), Deakin University, Burwood, VIC, Australia; Department of Clinical Pharmacy & Pharmacy Practice (Prof N Ismail PhD), Asian Institute of Medicine, Science and Technology, Bedong, Malaysia; Malaysian Academy of Pharmacy, Puchong, Malaysia (Prof N Ismail PhD); Public Health Department of Social Medicine (Prof H Iso MD), Osaka University, Suita, Japan; Department of Medicine (M C Ituka MD), University of Yaoundé, Yaounde, Cameroon; Department of Health Services Research (M Iwagami PhD), Department of Public

Health Medicine (Prof K Yamagishi MD), University of Tsukuba, Tsukuba, Japan; Department of Non-Communicable Disease Epidemiology (M Iwagami PhD), Department of Non-communicable Disease Epidemiology (A Nur MPH), London School of Hygiene & Tropical Medicine, London, UK; Department of Global Health (C J Iwu-Jaja PhD), South African Medical Research Council, Cape Town, South Africa; Department of Global Health (C J Iwu-Jaja PhD), Department of Epidemiology (J L Tamuzi MSc), Stellenbosch University, Cape Town, South Africa; Department of Physical and Medicine (L Jacob MD), Université Paris Cité, Paris, France; Research and Development Unit (L Jacob MD), Biomedical Research Networking Center for Mental Health Network (CiberSAM), Barcelona, Spain; UCL Institute for Global Health (Prof S Jaffar PhD), University of London, London, UK; College of Medicine and Medical Sciences (H Jahrami PhD), Arabian Gulf University, Manama, Bahrain; Ministry of Health, Manama, Bahrain (H Jahrami PhD); Department of Internal Medicine (A Jain MD), University of Iowa Hospitals and Clinics, Iowa City, IA, USA; Non-Communicable Disease Department (R Jain MD), Jain Hospital MSS, Kanpur, India; Department of Medicine (R Jain MD), Mahavir Sikshan Sansthan, Kanpur, India; Department of Health and Safety (A A Jairoun PhD), Dubai Municipality, Dubai, United Arab Emirates; The World Academy of Sciences UNESCO, Trieste, Italy (Prof M Jakovljevic PhD); Shaanxi University of Technology, Hanzhong, China (Prof M Jakovljevic PhD); Department of Medicine (S Javaid MD), University of Mississippi Medical Center, Jackson, MS, USA; Department of Medicine (S Javaid MD), Jinnah Sindh Medical University, Karachi, Pakistan; Centre of Studies and Research (S Jayapal PhD), Ministry of Health, Muscat, Oman; Department of Biochemistry (Prof S Jayaram MD), Government Medical College, Mysuru, India; Department of Endocrinology, Diabetes and Metabolism (Prof F K Jebasingh DM), Christian Medical College and Hospital (CMC), Vellore, India; Department of Epidemiology and Health Promotion (Prof S Jee PhD), Institute of Health Services Research (G Kim PhD, Prof E Park PhD), Department of Preventive Medicine (Prof E Park PhD), Yonsei University, Seoul, South Korea; Department of Public Health (A Jema MPH), Madda Walabu University, Goba, Ethiopia; Department of Internal Medicine (B M Jeswani MBBS), GCS Medical College, Hospital & Research Centre, Ahmedabad, India; Rothschild Foundation Hospital (Prof J B Jonas MD), Institut Français de Myopie, Paris, France; Singapore Eye Research Institute (Prof J B Jonas MD), Singapore Eye Research Institute, Singapore, Singapore; Department of Economics (C E Joshua BSc), National Open University, Benin City, Nigeria; Department of Family Medicine and Public Health (J J Jozwiak PhD), University of Opole, Opole, Poland; Institute of Family Medicine and Public Health (M Jürisson PhD), University of Tartu, Tartu, Estonia; Health Economics Unit (B Kaambwa PhD), College of Medicine and Public Health (B Kaambwa PhD, G R Naik PhD, S Ullah PhD), Department of Nursing and Health Sciences (S Shorofi PhD), Flinders University, Adelaide, SA, Australia; Department of Oral and Maxillofacial Pathology (V Kadashetti MDS), Department of Public Health Dentistry (Prof S KM MD), Krishna Vishwa Vidyapeeth (Deemed to be University), Karad, India; Department of Pharmacology (A K Kakkar MD), Post Graduate Institute of Medical Education and Research, Chandigarh, India; Department of Endocrinology (S Kalra DM), Bharti Hospital Karnal, Karnal, India; T. H. Chan School of Public Health (S B Kankam MD), Harvard University, Boston, USA; Cardiology Division (A R Kanmanthareddy MD), Creighton University, Omaha, NE, USA; College of Public Health (A R Kanmanthareddy MD), Department of Environmental, Agricultural and Occupational Health (J Taiba PhD), University of Nebraska Medical Center, Omaha, NE, USA; Faculty of Dentistry (K K Kanmodi MPH, A A Salami BDS), University of Puthisastra, Phnom Penh, Cambodia; Office of the Executive Director (K K Kanmodi MPH), Cephas Health Research Initiative Inc, Ibadan, Nigeria; The Hansjörg Wyss Department of Plastic and Reconstructive Surgery (R S Kantar MD), NYU Langone Health, New York, NY, USA; Cleft Lip and Palate Surgery Division (R S Kantar MD), Global Smile Foundation, Norwood, MA, USA; Community

and Primary Care Research Group (D Kar MD), Plymouth University, Plymouth, UK; 2nd Cardiology Department (P Karakasis MSc), Second Department of Cardiology (Prof D Patoulias PhD), Aristotle University of Thessaloniki, Thessaloniki, Greece; Surgery Research Unit (Prof J H Kauppila MD), University of Oulu, Oulu, Finland; International Research Center of Excellence (G A Kayode PhD), Institute of Human Virology Nigeria, Abuja, Nigeria; Julius Centre for Health Sciences and Primary Care (G A Kayode PhD), Utrecht University, Utrecht, Netherlands; Department of Public Health (S Kedir MSc), Werabe University, Werabe, Ethiopia; Department of Surgery (D Kehagias PhD), University of Patras, Patras, Greece; Department of Human Nutrition (E Kesse-Guyot PhD), National Research Institute for Agriculture, Food and Environment, Jouy-en-Josas, France; Department of Health, Medicine and Human Biology (M Touvier PhD), Sorbonne Paris Nord University, Bobigny, France (E Kesse-Guyot PhD); Amity Institute of Forensic Sciences (H Khajuria PhD, B P Nayak PhD), Amity Institute of Public Health (M Shannawaz PhD), Amity institute of Public health and hospital administration (P S Singh PhD), Amity University, Noida, India; Department of Biostatistics (Prof A Khalilian PhD), Department of Medical-Surgical Nursing (S Shorofi PhD), Mazandaran University of Medical Sciences, Sari, Iran; Department of Public Health (Prof M Khalis PhD), Mohammed VI Center for Research and Innovation, Rabat, Morocco; Higher Institute of Nursing Professions and Health Techniques, Rabat, Morocco (Prof M Khalis PhD); Natural and Medical Sciences Research Center (A Khan PhD, A Ullah MS), Dept of Biological Sciences and Chemistry (Z Naureen PhD), University of Nizwa, Nizwa, Oman; Department of Community Medicine (M Khan MPH), National Institute of Preventive and Social Medicine, Dhaka, Bangladesh; Primary Care Department (M A Khan MSc), NHS North West London, London, UK; Department of Internal Medicine (M S Khan MD), John H. Stroger, Jr. Hospital of Cook County, Chicago, IL, USA; Department of Internal Medicine (M S Khan MD), Dow University of Health Sciences, Karachi, Pakistan; Institute of Population Health Sciences (N Khan PhD), Newcastle University, Newcastle Upon Tyne, UK; Department of Health (V Khanal PhD), Nepal Development Society, Chitwan, Nepal; Department of Preventable Non Communicable Disease (V Khanal PhD), Menzies School of Health Research, Alice Springs, NT, Australia; Department of Biochemistry (F Khidri PhD), Liaquat University Of Medical and Health Sciences, Jamshoro, Pakistan; Department of Internal Medicine (A A Khosla MD), Corewell Health East William Beaumont University Hospital, Royal Oak, MI, USA; Department of Medical Oncology (A A Khosla MD), Miami Cancer Institute, Miami, FL, USA; Department of Clinical Research (S Khosravi MD), Icahn School of Medicine at Mount Sinai, New York, NY, USA; Research Department (M Khosrowjerdi PhD), Inland Norway University of Applied Sciences, Elverum, Norway; Faculty of Health Sciences (H Khusun PhD), University of Muhammadiyah Prof. Dr. Hamka, Jakarta, Indonesia; Program Division (H Khusun PhD), SEAMEO Regional Center for Food and Nutrition, Jakarta, Indonesia; Broad Institute of MIT and Harvard, Cambridge, MA, USA (M Kim MD); School of Traditional Chinese Medicine (Y Kim PhD), Xiamen University Malaysia, Sepang, Malaysia; Millennium Prevention, Inc., Westwood, MA, USA (R W Kimokoti MD); School of Health Sciences (Prof A Kisa PhD), Kristiania University College, Oslo, Norway; Department of International Health and Sustainable Development (Prof A Kisa PhD), Tulane University, New Orleans, LA, USA; School of Pharmacy (Prof L Kishore PhD), Maharaja Agrasen University, Himachal Pradesh, India; Department of General Practice and Family Medicine (Prof O Korzh DSc), Kharkiv National Medical University, Kharkiv, Ukraine; Department of Epidemiology (Prof K Kostev PhD), IQVIA, Frankfurt am Main, Germany; University Hospital Marburg, Marburg, Germany (Prof K Kostev PhD); Kasturba Medical College, Manipal (S Koulmane Laxminarayana MD), Manipal Academy of Higher Education, Udupi, India; School of Pharmacy (Prof I A Kretchy PhD), University of Ghana, Legon, Ghana; School of Applied Science (C Kua PhD), Republic Polytechnic, Singapore, Singapore; Department of

Demography (Prof B Kuate Defo PhD), Department of Social and Preventive Medicine (Prof B Kuate Defo PhD), University of Montreal, Montreal, QC, Canada; Department of Biochemistry (Prof M Kuddus PhD), Department of Public Health (M G M Zeariya PhD), University of Hail, Hail, Saudi Arabia; Center of Medicine and Public Health (M Kulimbet MSc), Asfendiyarov Kazakh National Medical University, Almaty, Kazakhstan; Department of Medicine (V Kulkarni MS), Digital Health and Informatics Directorate (Prof S M McPhail PhD), Queensland Health, Brisbane, QLD, Australia; Centre for Digital Transformation (V Kumar PhD), Indian Institute of Management, Ahmedabad, Ahmedabad, India; Centre for Studies in Economics and Planning (V Kumar PhD), Central University of Gujarat, Gandhinagar, India; Public Health, School of Medicine and Dentistry (S Kundu MPH), Griffith University, Gold Coast, QLD, Australia; Section of Cardiology (Prof S K Kunutsor PhD), University of Manitoba, Winnipeg, MB, Canada; Translational Health Sciences (Prof S K Kunutsor PhD), University of Bristol, Bristol, UK; Department of Medicine (O P Kurmi PhD), Department of Psychiatry and Behavioural Neurosciences (Prof A T Olagunju PhD), McMaster University, Hamilton, ON, Canada; Faculty of Medicine and Health Science (M Kurniasari PhD), Universitas Kristen Satya Wacana, Salatiga, Indonesia; Department of Public Health and Epidemiology (D Kusuma DSc), Khalifa University of Science and Technology, Abu Dhabi, United Arab Emirates; Faculty of Public Health (D Kusuma DSc), University of Indonesia, Depok, Indonesia; Clinical Research Center (V Kytö MD), Turku University Hospital, Turku, Finland; Heart Center (V Kytö MD), University of Turku and Turku University Hospital, Turku, Finland; Department of Clinical Sciences and Community Health (Prof C La Vecchia MD), University of Milan, Milan, Italy; National Institute for Health Research (NIHR) Oxford Biomedical Research Centre, Oxford, UK (B Lacey DPhil); Integrated Department of Epidemiology, Health Policy, Preventive Medicine and Pediatrics (Prof C Lahariya MD), Foundation for People-centric Health Systems, New Delhi, India; Centre for Health: The Specialty Practice, New Delhi, India (Prof C Lahariya MD); School of Digital Science (D Lai PhD), Institute of Applied Data Analytics (D Lai PhD), Universiti Brunei Darussalam (University of Brunei Darussalam), Bandar Seri Begawan, Brunei; Unidad de Genética y Salud Pública (Prof I Landires MD), Instituto de Ciencias Médicas, Las Tablas, Panama; Ministry of Health (Prof I Landires MD), Hospital Joaquín Pablo Franco Sayas, Las Tablas, Panama; Department of Pediatrics (Z S Lassi PhD), Aga Khan University, Karachi, Pakistan; Faculty of Medicines (H T Le MD), Faculty of Medicine (N Le MD), Department of Internal Medicine (T H Tran MD), University of Medicine and Pharmacy at Ho Chi Minh City, Ho Chi Minh, Viet Nam; Department of Cardiovascular Research (N Le MD), Methodist Hospital, Merrillville, IN, USA; Clinical Trial Center (H Lee PhD), Ewha Womans University, Seoul, South Korea; Department of Medical Science (M Lee PhD), Ajou University School of Medicine, Suwon, South Korea; Southampton Clinical Trials Unit (P H Lee PhD), Department of Surgery (G Verras MSc), University of Southampton, Southampton, UK; Department of Precision Medicine (Prof S Lee MD), Sungkyunkwan University, Suwon-si, South Korea; Department of Family Medicine (W Lee PhD), University of Texas Medical Branch, Galveston, TX, USA; Center for Dentistry and Oral Hygiene (A Li PhD), University of Groningen, Groningen, Netherlands; Stomatological Hospital (A Li PhD), Southern Medical University, Guangzhou, China; Department of Health Promotion and Health Education (M Li PhD), National Taiwan Normal University, Taipei, Taiwan; Department of Psychiatry (W Li PhD, T Rhee PhD), Yale University, New Haven, CT, USA; Department of Endocrinology and Metabolism (Y Li PhD), The First Hospital of China Medical University, Shenyang, China; Breast Tumor Center (Q Lin MPH), Sun Yat-sen Memorial Hospital, Guangzhou, China; Department of Medical Sciences (D Lindholm MD), Uppsala University, Uppsala, Sweden; Department of Medicine (D Lindholm MD), Norrtälje Hospital (Tiohundra), Norrtälje, Sweden; Department of Epidemiology and Biostatistics (Prof J Liu PhD), School of Public Health (H Zhang MS), Institute of Child and Adolescent Health (Prof Z

Zou MD), Peking University, Beijing, China; One Health Research Group (J López-Gil PhD), Universidad de Las Américas (University of the Americas), Quito, Ecuador; Institute of Nutritional Sciences (Prof S Lorkowski PhD), Friedrich Schiller University Jena, Jena, Germany; Competence Cluster for Nutrition and Cardiovascular Health (nutriCARD), Jena, Germany (Prof S Lorkowski PhD); School of Medicine (Prof G Lucchetti PhD), Federal University of Juiz de Fora, Juiz de Fora, Brazil; Dodoma Medical Research Centre (A M Lutambi PhD), National Institute for Medical Research, Dodoma, Tanzania; Centre for Public Health and Wellbeing (Z Ma PhD), University of the West of England, Bristol, UK; Department of Human Nutrition Research (J A Magaña Gómez PhD), Autonomous University of Sinaloa, Culiacán, Mexico; Cihan University Sulaimaniya Research Center (CUSRC) (N H Mahmood PhD), Cihan University Sulaimaniya, Sulaymaniyah, Iraq; Cyprus International Institute for Environmental and Public Health (Prof K C C Makris PhD), Cyprus University of Technology, Limassol, Cyprus; Rama Medical College Hospital and Research Centre, Uttar Pradesh, India (K Malhotra MBBS); Department of Electrical Engineering (I Malik PhD), Department of Health and Rehabilitation Sciences (Prof G Nambi PhD), Prince Sattam bin Abdulaziz University, Al Kharj, Saudi Arabia; Poche Centre for Indigenous Health (Prof A A Mamun PhD), The University of Queensland, Brisbane, QLD, Australia (M Moni PhD); Department of Biomedical Engineering (H Marateb PhD), University of Isfahan, Isfahan, Iran; Biomedical Engineering Research Center (CREB) (H Marateb PhD), Universitat Politècnica de Catalunya (Barcelona Tech - UPC), Barcelona, Spain; Department of Food, Environmental and Nutritional Sciences (DeFENS) (M Marino PhD), Department of Food, Environmental and Nutritional Sciences (Prof S Perna PhD), University of Milan, Milano, Italy; Department of Biochemistry (A Marjani PhD), Golestan Research Center of Gastroenterology and Hepatology (G Roshandel PhD), Golestan University of Medical Sciences, Gorgan, Iran; Department of Non-communicable Diseases and Mental Health (R Martinez-Piedra BSc), Pan American Health Organization, Washington, DC, USA; Department of Nutrition and Dietetics (M Martorell PhD), Centre for Healthy Living (M Martorell PhD), University of Concepción, Concepción, Chile; Clinical Institute of Medical and Chemical Laboratory Diagnostics (Prof W März MD), Medical University of Graz, Graz, Austria; Medical Clinic V (Prof W März MD), Heidelberg University, Mannheim, Germany; Department of Anatomy, Genetics and Biomedical Informatics (Y Mathangasinghe PhD), Postgraduate Institute of Medicine (S N K Navaratna MD), Department of Surgery (D P Wickramasinghe MD), University of Colombo, Colombo, Sri Lanka; Faculty of Medical Sciences (Prof T Matthias MD), University of Sri Jayewardenepura, Sri Lanka, Nugegoda, Sri Lanka; Nuffield Department of Population Health (M Mazidi PhD), University of Oxford, London, UK; Australian Centre for Health Services Innovation (Prof S M McPhail PhD), Queensland University of Technology, Kelvin Grove, QLD, Australia; Department of Healthcare (Prof E A Mechili PhD), University of Vlora, Vlora City, Albania; Clinic of Social and Family Medicine (Prof E A Mechili PhD), Laboratory of Toxicology (T K Nikolouzakis PhD), University of Crete, Heraklion, Greece; National Child Health Program (M P Medel Salas MD), Ministry of Health, Santiago, Chile; Department of Epidemiology and Biostatistics (K Mehrabani-Zeinabad PhD), Cardiac Rehabilitation Research Center (Prof M Sadeghi MD), Isfahan University of Medical Sciences, Isfahan, Iran; Department of Public Health (T Mekene Meto MPH), Arba Minch University, Arba Minch, Ethiopia; Universidad Nacional Mayor de San Marcos, Lima, Peru (W Mendoza MD); University Centre Varazdin (T Mestrovic PhD), University North, Varazdin, Croatia; Department of Pharmacology (Prof C D K Mettananda PhD), Department of Paediatrics (Prof S Mettananda DPhil), University of Kelaniya, Ragama, Sri Lanka; Clinical Medicine Department (Prof C D K Mettananda PhD), University Paediatrics Unit (Prof S Mettananda DPhil), Colombo North Teaching Hospital, Ragama, Sri Lanka; Department of Cardiology (H Miao MD), Peking Union Medical College, Beijing, China; Pacific Institute for Research & Evaluation,

Beltsville, MD, USA (T R Miller PhD); Internal Medicine Programme (Prof E M Mirrakhimov PhD), Kyrgyz State Medical Academy, Bishkek, Kyrgyzstan; Department of Atherosclerosis and Coronary Heart Disease (Prof E M Mirrakhimov PhD), National Center of Cardiology and Internal Disease, Bishkek, Kyrgyzstan; National Data Management Center for Health (A Misganaw PhD), Ethiopian Public Health Institute, Addis Ababa, Ethiopia; Department of Endocrinology & Metabolism (Prof M Mittal MD), All India Institute of Medical Sciences, Bhopal, India; College of Applied and Natural Science (J Mohamed MSc), University of Hargeisa, Hargeisa, Somalia; RAK College of Nursing (M Mohamed PhD), RAK Medical and Health Sciences University, Ras Alkhima, United Arab Emirates; Nursing College (M Mohamed PhD), Sohag University, Sohag, Egypt; Molecular Biology Unit (N S Mohamed MSc), Bio-Statistical and Molecular Biology Department (N S Mohamed MSc), Sirius Training and Research Centre, Khartoum, Sudan; Department of Biophysics (T Mohammad PhD), Department of Laboratory Medicine (A Singh PhD), All India Institute of Medical Sciences, New Delhi, India; Modeling in Health Research Center (A Mohammadian-Hafshejani PhD), Shahrekord University of Medical Sciences, Shahrekord, Iran; Health Systems and Policy Research Unit (Prof S Mohammed PhD), Department of Paediatrics (S Musa MSc), Department of Community medicine (U M Umar MPH), Ahmadu Bello University, Zaria, Nigeria; Heidelberg Institute of Global Health (HIGH) (Prof S Mohammed PhD), Department of Ophthalmology (S Panda-Jonas MD), Heidelberg University, Heidelberg, Germany; Department of Biomedical and Dental Sciences and Morphofunctional Imaging (Prof S Mondello MD), Messina University, Messina, Italy; AI & Cyber Futures Institute (M Moni PhD), Charles Sturt University, Bathurst, NSW, Australia; Shahid Rajai Centre for Educational Research and Clinical Cardiology (S Montazeri Namin MD), Islamic Azad University, Tehran, Iran; Faculty of Medicine (A Moodi Ghalibaf MD), Birjand University of Medical Sciences, Birjand, Iran; Department of Epidemiology and Biostatistics (Y Moradi PhD), Kurdistan University of Medical Sciences, Sanandaj, Iran; Division of Plastic and Reconstructive Surgery (S D Morrison MD), University of Washington Medical Center, Seattle, WA, USA; Unit of Pharmacotherapy, Epidemiology and Economics (Prof S Mubarik PhD), University of Groningen (Rijksuniversiteit Groningen), Groningen, Netherlands; Department of Epidemiology and Biostatistics (Prof S Mubarik PhD, Prof C Yu PhD), Wuhan University, Wuhan, China; Department of Surgery (F Mulita PhD), General University Hospital of Patras, Patras, Greece; Faculty of Medicine (F Mulita PhD), University of Thessaly, Larissa, Greece; Department of Community and Global Health (Y Munkhsaikhan MD), The University of Tokyo, Tokyo, Japan; Clinical Epidemiology Research Unit (E Murillo-Zamora PhD), Mexican Institute of Social Security, Villa de Alvarez, Mexico; Postgraduate in Medical Sciences (E Murillo-Zamora PhD), Universidad de Colima, Colima, Mexico; College of Medicine (Prof G Mustafa MD), Shaqra University, Riyadh, Shaqra, Saudi Arabia; Department of Pediatrics & Pediatric Pulmonology (Prof G Mustafa MD), Institute of Mother & Child Care, Multan, Pakistan; Department of Research Methods (S Muthu PhD), Orthopaedic Research Group, Coimbatore, India; Department of Biotechnology (S Muthu PhD), Karpagam Academy of Higher Education (Deemed to be University), Coimbatore, India; Department of Internal Medicine (J C Mwita MD, G M Rwegerera MD), University of Botswana, Gaborone, Botswana; Department of Psychiatry (W Myung PhD), Department of Food and Nutrition (A P Okekunle PhD), Seoul National University, Seoul, South Korea; Department of Neuropsychiatry (W Myung PhD), Seoul National University Bundang Hospital, Seongnam, South Korea; Faculty of Basic Medical Sciences (A B Nafiu PhD), Federal University Dutse, Dutse, Nigeria; Institute of Epidemiology and Medical Biometry (Prof G Nagel PhD), Ulm University, Ulm, Germany; Department of Medicine (H Naik MS), University of British Columbia, Vancouver, BC, Canada; Suraj Eye Institute, Nagpur, India (V Nangia MD); National Dental Research Institute Singapore (G G Nascimento PhD), Duke-NUS Medical School, Singapore, Singapore;

Department of Internal Medicine (M Nassar PhD), Mount Sinai Health System, Queens, NY, USA; Department of Circulation and Medical Imaging (J Nauman PhD), Norwegian University of Science and Technology, Trondheim, Norway; School of Medicine (N PhD), Xiamen University, Xiamen, China; Department of Health Promotion (A Nazri-Panjaki MSc), Health Promotion Research Center (H Okati-Aliabad PhD), Zahedan University of Medical Sciences, Zahedan, Iran; Department of General Surgery (I Negoi PhD), Emergency University Hospital Bucharest, Bucharest, Romania; Department of Cardiology (R I Negoi PhD), Cardio-Aid, Bucharest, Romania; Department of Biological Sciences (J W Ngunjiri PhD), University of Embu, Embu, Kenya; Harvard T.H. Chan School of Public Health (D Nguyen BS), Harvard University, Cambridge, MA, USA; Department of Medical Engineering (D Nguyen BS), University of South Florida, Tampa, FL, USA; Department of Surgery (P T Nguyen MD), Danang Family Hospital, Danang, Viet Nam; Hitotsubashi Institute for Advanced Study (HIAS) (P T Nguyen DrPH), Hitotsubashi University, Tokyo, Japan; Institute for Cancer Control (P T Nguyen DrPH), National Cancer Center, Chuo-ku, Japan; International Islamic University Islamabad, Islamabad, Pakistan (R K Niazi PhD); Department of Humanities and Social Science (L Nieddu PhD), UNINT, University for International Studies in Rome, Rome, Italy; Department of General Surgery (T K Nikolouzakis PhD), University Hospital of Heraklion, Heraklion, Greece; Department of Internal Medicine and Specialties (J Nkeck MD), University of Yaoundé I, Yaounde, Cameroon; Global Research Institute (Prof S Nomura PhD), Keio University, Tokyo, Japan; Maternal and Child Health Division (S Noor MS), International Centre for Diarrhoeal Disease Research, Bangladesh, Dhaka, Bangladesh; Department of Statistics (S Noor MS), Shahjalal University of Science and Technology, Sylhet, Bangladesh; Department of Microbiology and Molecular Genetics (M Noreen PhD), The Women University Multan, Multan, Pakistan; Division of Cardiology (J Noubiap MD), Department of Neurosurgery (Y Senol MD), Department of Bioengineering and Therapeutical Sciences (Prof M Zastrozhin PhD), University of California San Francisco, San Francisco, CA, USA; Health Research Institute (M Nouri PhD), School of Medicine (S Soraneh MD), Babol University of Medical Sciences, Babol, Iran; Department of Paediatrics (C A Nri-Ezedi PhD), Nnamdi Azikiwe University, Awka, Nigeria; School of Information (F Nugen PhD), Health Initiative of the Americas (Prof V E Villalobos-Daniel PhD), University of California Berkeley, Berkeley, CA, USA; Unit of Microbiology and Public Health (V Nuñez-Samudio PhD), Instituto de Ciencias Medicas, Las Tablas, Panama; Department of Public Health (V Nuñez-Samudio PhD), Ministry of Health, Herrera, Panama; Center for Health System and Strategy (A Nur MPH), Ministry of Health, Jakarta, Indonesia; Center of Excellence in Reproductive Health Innovation (CERHI) (C I Nzoputam MPH), University of Benin, Benin City, Nigeria; Department of Applied Economics and Quantitative Analysis (Prof B Oancea PhD), University of Bucharest, Bucharest, Romania; Bioinformatics Department (Prof B Oancea PhD), National Institute of Research and Development for Biological Sciences, Bucharest, Romania; Department of Veterinary Public Health and Preventive Medicine (I A Odetokun PhD), University of Ilorin, Ilorin, Nigeria; Sheffield Centre for Health and Related Research (J O Oguta MSc), University of Sheffield, Sheffield, UK; Department of Preventive Medicine (Prof I Oh MD), Department of Pediatrics (Prof D Yon MD), Kyung Hee University, Seoul, South Korea; School of Pharmacy (O C Okonji MSc), University of the Western Cape, Cape Town, South Africa; Department of Psychiatry (Prof A T Olagunju PhD), University of Lagos, Lagos, Nigeria; Federal Institute of Industrial Research Oshodi, Lagos Nigeria, Lagos, Nigeria (T A Olasehinde PhD); Department of Biochemistry, Genetics and Microbiology (T A Olasehinde PhD), University of KwaZulu-Natal, Westville, South Africa; Center for Clinical and Epidemiological Research (A B Oliveira PhD), University of São Paulo, São Paulo, Brazil; Associação Brasileira de Cefaleia em Salvas e Enxaqueca (ABRACES), São Paulo, Brazil (A B Oliveira PhD); Cardiology Department (Prof G M M Oliveira PhD), Federal University of Rio de

Janeiro, Rio de Janeiro, Brazil; Department of Pharmacology and Toxicology (Prof H A Omar PhD), Beni-Suef University, Beni-Suef, Egypt; Diplomacy and Public Relations Department (A Omar Bali PhD), University of Human Development, Sulaymaniyah, Iraq; Department of Public Health (S Ong FAMS), Ministry of Health, Bandar Seri Begawan, Brunei; Institute of Health Sciences (S Ong FAMS), Universiti Brunei Darussalam, Bandar Seri Begawan, Brunei; Department of Pharmacotherapy and Pharmaceutical Care (M Ordak PhD), Department of Biochemistry and Pharmacogenomics (M Zielińska MPharm), Medical University of Warsaw, Warsaw, Poland; Department of Medicine (Prof A Ortiz MD), Universidad Autónoma de Madrid (Autonomous University of Madrid), Madrid, Spain; Department of Nephrology and Hypertension (Prof A Ortiz MD), The Institute for Health Research Foundation Jiménez Díaz University Hospital, Madrid, Spain; Department of Biological Sciences (A Osborne MSc), Njala University, Sierra Leone, Freetown, Sierra Leone; Department of Statistics and Econometrics (A Otoiu PhD, I Petcu PhD), Bucharest University of Economic Studies, Bucharest, Romania; Division of Infectious Diseases (Prof A Ouyahia PhD), University Hospital of Setif, Sétif, Algeria; West African Center for Cell Biology of Infectious Pathogens (I A Owusu PhD), University of Ghana, Legon-Accra, Ghana; Department of Life and Medical Science (T Oyelade PhD), University of Hertfordshire, Hatfield, UK; Department of Respiratory Medicine (Prof M P P A DNB), Jagadguru Sri Shivarathreeswara University, Mysore, India; National School of Public Health (A Padron-Monedero PhD), Institute of Health Carlos III, Madrid, Spain; Health Services Management Training Centre (T Palicz MD), Semmelweis University, Budapest, Hungary; Hungarian Health Management Association, Budapest, Hungary (T Palicz MD); Centre for Biotechnology (S K Panda PhD), Siksha 'O' Anusandhan (Deemed to be University), Bhubaneswar, India; Centre for Research and Development (Prof S R Pandi-Perumal MSc), Chandigarh University, Punjab, India; Division of Research and Development (Prof S R Pandi-Perumal MSc), Lovely Professional University, Phagwara, India; Clinical Research Department (S Pant MPH), Nepal Health Research Council, Kathmandu, Nepal; Vision and Eye Research Institute (Prof S Pardhan PhD), Anglia Ruskin University, Cambridge, UK; Department of Forensic Medicine and Toxicology (U Parekh MD), All India Institute of Medical Sciences, Rajkot, India; Department of Community Medicine (P P Parija MD), All India Institute of Medical Sciences, Jammu, India; Division of Health Policy and Management (R R Parikh MD), University of Minnesota, Minneapolis, MN, USA; Department of Medical Sciences (R Passera PhD), University of Torino, Torino, Italy; Department of Imaging (R Passera PhD), AOU Città della Salute e della Scienza di Torino, Torino, Italy; School of Dentistry (J Patel BSc), University of Leeds, Leeds, UK; Australian Institute of Health Innovation (P Peprah MSc), Macquarie University, Sydney, NSW, Australia; Institute of Collective Health (Prof M Pereira PhD), Federal University of Bahia, Salvador, Brazil; Mario Negri Institute for Pharmacological Research, Bergamo, Italy (N Perico MD, Prof G Remuzzi MD); Facultad de Medicina (Faculty of Medicine) (F E Petermann-Rocha PhD), Universidad Diego Portales (Diego Portales University), Santiago, Chile; School of Cardiovascular and Metabolic Health (F E Petermann-Rocha PhD), University of Glasgow, Glasgow, UK; Department of Internal Medicine (H Pham MD), University of Arizona, Tucson, AZ, USA; Department of Internal Medicine (D S Popovic PhD), University of Novi Sad, Novi Sad, Serbia; Clinic for Endocrinology, Diabetes and Metabolic Disorders (D S Popovic PhD), Clinical Center of Vojvodina, Novi Sad, Serbia; Global Health Department (I Potani PhD), Ripple Global Health, Lilongwe, Malawi; Department of Humanities and Social Sciences (Prof J Pradhan PhD), National Institute of Technology Rourkela, Rourkela, India; Department of Community Medicine and Public Health (P M S Pradhan MD), Tribhuvan University, Kathmandu, Nepal; Department of Clinical Research and Epidemiology (M Prasad MD), Institute of Liver and Biliary Sciences, New Delhi, India; Department of Biostatistics, Epidemiology, and Informatics (J Puvvula PhD), University of Pennsylvania, Philadelphia,

PA, USA; Department of Medical Oncology (Prof V Radhakrishnan MD), Cancer Institute (W.I.A), Chennai, India; Division of Psychology and Mental Health (M R Radojčić PhD), University of Manchester, Manchester, UK; College of Science (F M Rahman PhD), University of Sulaimani, Sulaymaniyah, Iraq; College of Health Sciences (F M Rahman PhD), Cihan University - Sulaimaniya, Sulaymaniyah, Iraq; College of Medicine and Health Sciences (M Rahman PhD), National University of Science and Technology, Sohar, Oman; Institute of Health and Wellbeing (Prof M Rahman PhD), Federation University Australia, Berwick, VIC, Australia; School of Nursing and Midwifery (Prof M Rahman PhD), La Trobe University, Melbourne, VIC, Australia; Department of Public Health (V Rahmanian PhD), Torbat Jam Faculty of Medical Sciences, Torbat Jam, Iran; Health Service Research and Quality of Life Center (CEReSS) (Prof M Rahmati PhD), Aix-Marseille University, Marseille, France; Society for Health and Demographic Surveillance, Suri, India (R Rai PhD); Institute of Nutrition (R Rai PhD), Mahidol University, Salaya, Thailand; Department of Medical, Surgical and Experimental Sciences (I Raimondo MD), University of Sassari, Sassari, Italy; Gynecology and Breast Care Center (I Raimondo MD), Mater Olbia Hospital (Qatar Foundation Endowment and Policlinico Universitario Agostino Gemelli IRCCS Foundation), Olbia, Italy; Centre for Chronic Disease Control, New Delhi, India (P Rajput PhD); Department of Anatomy (C Ramasamy MD), Govt. Siddhartha Medical College, Vijayawada, India; Department of Radiology (S Ramasamy MD), Stanford University, Stanford, CA, USA; School of Nursing & Health Sciences (S Ramazanu PhD), Hong Kong Metropolitan University, Hong Kong, China; Department of Research (C L Ranabhat PhD), Eastern Scientific LLC, Richmond, KY, USA; Planetary Health Research Centre (PHRC), Kathmandu, Nepal (C L Ranabhat PhD); Department of Oral Pathology, Microbiology and Forensic Odontology (S Rao MDS), Sharavathi Dental College and Hospital, Shimogga, India; Brigham and Women's Hospital (S Rashedi MD), Harvard Medical School, Boston, MA, USA; Department of Family Medicine (Prof D Rathish PhD), Department of Parasitology (Prof K G Weerakoon PhD), Department of Community Medicine (N D Wickramasinghe MD), Rajarata University of Sri Lanka, Anuradhapura, Sri Lanka; Department of Neurosurgery (I Rautalin PhD), Helsinki University Hospital, Helsinki, Finland; The National Institute for Stroke and Applied Neurosciences (I Rautalin PhD), Auckland University of Technology, Auckland, New Zealand; Inovus Medical, St Helens, UK (D L Rawaf MD); Academic Public Health England (Prof S Rawaf MD), Public Health England, London, UK; Department of Biological Sciences (Prof E M M Redwan PhD), King Abdulaziz University, Jeddah, Egypt; Department of Protein Research (Prof E M M Redwan PhD), Research and Academic Institution, Alexandria, Egypt; Institute for Health, Health Care Policy and Aging Research (S Rege PhD), Rutgers University, New Brunswick, NJ, USA; Department of Internal Medicine (A Rehman MD), Department of Radiology (M Tanwar MD), University of Alabama at Birmingham, Birmingham, AL, USA; Department of Internal Medicine (A Rehman MD), King Edward Medical University, Lahore, Pakistan; Department of Epidemiology and Biostatistics (Prof M Rezaeian PhD), Rafsanjan University of Medical Sciences, Rafsanjan, Iran; Department of Public Health Sciences (T Rhee PhD), University of Connecticut, Farmington, CT, USA; Department of Nursing in Women's Health (T Rodrigues da Silva PhD), Federal University of São Paulo, São Paulo, Brazil; Department of Pharmacology and Toxicology (Prof J A B Rodriguez PhD), University of Antioquia, Medellin, Colombia; Warwick Medical School (Prof J A B Rodriguez PhD), University of Warwick, Coventry, UK; Department of Clinical Research (Prof L Roever PhD), University of Sao Paulo, Ribeirão Preto, Brazil; Center for Indigenous Health Research (P Rohloff MD), Wuqu' Kawoq Maya Health Alliance, Tecpan, Guatemala; Department of Public Health (M Rony MPH), Bangladesh Open University, Gazipur, Bangladesh; Department of Analytical and Applied Economics (Prof H Rout PhD, C Swain MPhil), RUSA Centre of Excellence in Public Policy and Governance (Prof H Rout PhD), Utkal University,

Bhubaneswar, India; Department of Biochemistry and Food Analysis (N Roy PhD), Patuakhali Science and Technology University, Patuakhali, Bangladesh; Cardiovascular Department (Prof A M A Saad MD), Department of Microbiology and Immunology (G Yahya PhD), Zagazig University, Zagazig, Egypt; Department of Medical Pharmacology (Prof M M Saber-Ayad PhD), Public Health and Community Medicine Department (M R Salem MD), Cairo University, Giza, Egypt; Non-communicable Diseases Research Center (L Sabzmakan PhD), School of Medicine (M Shams-Beyranvand MSc), Alborz University of Medical Sciences, Karaj, Iran; Faculty of Health and Dentistry (Prof K P Sadarangani PhD), Diego Portales University, Santiago de Chile, Chile; Autonomous University of Chile, Santiago de Chile, Chile (Prof K P Sadarangani PhD); Operational Research Center in Healthcare (Prof U Saeed PhD), Near East University (NEU), Nicosia Cyprus, Turkiye; International Center of Medical Sciences Research (ICMSR), Islamabad, Pakistan (Prof U Saeed PhD); Biotechnology Research Center (Prof A Sahebkar PhD), Department of Medical Informatics (S Tabatabaei PhD), Clinial Research Development Unit (S Tabatabaei PhD), Department of Medicine (A Yarahmadi PhD), Mashhad University of Medical Sciences, Mashhad, Iran; Department of Community Medicine and Family Medicine (S S Sahoo MD, M Verma MD), All India Institute of Medical Sciences, Bathinda, India; Department of Nutrition and Dietetics (Prof S Sajadi PhD), Cihan University, Erbil, Erbil, Iraq; Department of Statistics (M R Sajid PhD), University of Gujrat, Gujrat, Pakistan; Department of Integrated Health Education (Prof L B Salaroli PhD), Federal University of Espirito Santo, Vitória, Brazil; Faculty of Allied Health Sciences (S Saleem PhD), Health Services Academy, Islamabad, Pakistan; College of Nursing (D Salihu PhD), Jouf University, Jouf, Saudi Arabia; Department of Food Processing Technology (T Sarkar PhD), West Bengal State Council of Technical Education, Malda, India; Department of Environmental Health Engineering (M Sarmadi MSc), Health Sciences Research Center (M Sarmadi MSc), Torbat Heydariyeh University of Medical Sciences, Torbat Heydariyeh, Iran; Department of Oral Pathology and Microbiology (Prof G S Sarode PhD, Prof S C Sarode PhD), Dr. D. Y. Patil Vidyapeeth, Pune (Deemed to be University), Pune, India; Department of Medical Informatics (J Saulam MSc), Kagawa University, Miki-cho, Japan; Food Processing and Nutrition (J Saulam MSc), Karnataka State Akkamahadevi Women's University, Vijayapura, India; Department of Public Health Sciences (M Sawhney PhD), University of North Carolina at Charlotte, Charlotte, NC, USA; Department of Preventive and Social Medicine (G Saya MD), Jawaharlal Institute of Postgraduate Medical Education and Research, Puducherry, India; Faculty of Business and Computing (Prof C Schinckus PhD), University of the Fraser Valley, Abbotsford, BC, Canada; Department of Finance (Prof C Schinckus PhD), International School of Management, Paris, France; Dr. D. Y. Patil Dental College and Hospital (S Selvaraj PhD), Dr. D. Y. Patil Vidyapeeth, Pune, India; Department of Biomedical Sciences (P Sengupta PhD), Gulf Medical University, Ajman, United Arab Emirates; Emergency Department (S Senthilkumaran PhD), Manian Medical Centre, Erode, India; Department of Medicine and Surgery (Y Sethi MBBS), Government Doon Medical College, Dehradun, India; National Heart, Lung, and Blood Institute (A Seylani BS), National Institutes of Health, Rockville, MD, USA; Rita A Patel Institute of Physiotherapy (S Shah PhD), The Charutar Vidya Mandal(CVM) University, Anand, India; Department of Medicine (M Sharath MBBS), Bangalore Medical College and Research Institute, Bangalore, India; Department for Evidence-based Medicine and Evaluation (A Sharifan PharmD), University for Continuing Education Krems, Krems, Austria; Department of Social and Behavioral Health (Prof M Sharma PhD), University of Nevada Las Vegas, Las Vegas, NV, USA; Institute of Forensic Science & Criminology (V Sharma PhD), Panjab University, Chandigarh, India; Department of Public Health (D Shiferaw MPH), Dambi Dollo University, Dembi Dollo, Ethiopia; Finnish Institute of Occupational Health, Helsinki, Finland (R Shiri PhD); Department of Community Medicine and Public Health (G Shrestha MD), Institute of Medicine,

Kathmandu, Nepal; Department of Public Health (R Shrestha MPH), Nepal Development Society, Pokhara, Nepal; Research Unit for Global Health (R Shrestha MPH), Aarhus University, Aarhus, Denmark; Kenneth H. Cooper Institute (Prof K Shuval PhD), Texas Tech University Health Sciences Center, Dallas, TX, USA; Advanced Materials Division (N R S Sibuyi PhD), Mintek, Randburg, South Africa; Department of Biotechnology (N R S Sibuyi PhD), University of the Western Cape, Bellville, South Africa; Department of Medical Microbiology and Infectious Diseases (E E Siddig MD), Erasmus University, Rotterdam, Netherlands; CICS-UBI Health Sciences Research Center (Prof L M R Silva PhD), University of Beira Interior, Covilhã, Portugal; Department of Pharmacology (H Singh DM), Government Medical College and Hospital, Chandigarh, India; School of Medicine (Prof J A Singh MD), Baylor College of Medicine, Houston, TX, USA; Department of Medicine Service (Prof J A Singh MD), US Department of Veterans Affairs (VA), Houston, TX, USA; Indian Council of Medical Research, New Delhi, India (L Singh PhD); Department of Community Medicine (M Singh MD), Department of Health, DELHI, India; Department of Infectious Diseases and Epidemiology (A A Skryabina MD), Department of Internal Disease (A V Starodubova DSc), Pirogov Russian National Research Medical University, Moscow, Russia; Student Research Committee (S Soraneh MD), Urmia University of Medical Sciences, Urmia, Iran; 3rd Department of Cardiology (M Spartalis PhD), University of Athens, Athens, Greece; Department of Public Health (M Stanikzai MPH), Kandahar University, Kandahar, Afghanistan; Nutrition and Dietetics Department (A V Starodubova DSc), Federal Research Institute of Nutrition, Biotechnology and Food Safety, Moscow, Russia; Global Observatory on Pollution and Health (Prof K Straif PhD), Boston College, Chestnut Hill, MA, USA; ISGlobal Instituto de Salud Global de Barcelona, Barcelona, Spain (Prof K Straif PhD); Department of Medical Sciences (Prof V Subramaniyan PhD), Sunway University, Subang Jaya, Malaysia; Department of Human Anatomy (M O Suleiman Odidi PhD), Federal University, Dutse, Dutse, Nigeria; GKT School of Medical Education (A Sulkowski BSc), School of Life Course and Population Sciences (Prof Y Wang PhD), King's College London, London, UK; School of Medicine, Medical Sciences and Nutrition (A Sultan Meo MPH), University of Aberdeen, Aberdeen, UK; Department of Biomedical Sciences (Z Sun PhD), Universiti Putra Malaysia, Selangor, Malaysia; Ashok & Rita Patel Institute of Physiotherapy (S Sunny PhD), Charotar University of Science and Technology, Anand, India; School of Public Health (D R Sunuwar MSc), University of Michigan, Ann Arbor, MI, USA; Department of Public Health (D R Sunuwar MSc), Asian College for Advance Studies, Purbanchal University, Lalitpur, Nepal; Department of Clinical Research and Development (Prof L Szarpak PhD), LUXMED Group, Warsaw, Poland; Collegium Medicul (Prof L Szarpak PhD), John Paul II Catholic University of Lublin, Lublin, Poland; Department of Medicine (Prof R Tabarés-Seisdedos PhD), University of Valencia, Valencia, Spain; Department of Basic Medical Sciences (S Tabatabaeizadeh PhD), Department of Internal Medicine (S Tabatabaeizadeh PhD), Islamic Azad University, Mashhad, Iran; Sri Ramachandra Medical College and Research Institute, Chennai, India (J Taiba PhD); Department of Dermato-Venereology (M Tampa PhD), Dr. Victor Babes Clinical Hospital of Infectious Diseases and Tropical Diseases, Bucharest, Romania; Department of Medicine (J L Tamuzi MSc), Northlands Medical Group, Omuthiya, Namibia; Department of Pharmacology and Therapeutics (S Tariq PhD), The University of Faisalabad, Faisalabad, Pakistan; Taking Our Best Shot, Houston, TX, USA (N Y Tat MS); Department of Research and Innovation (N Y Tat MS), Enventure Medical Innovation, Houston, TX, USA; Outpatient Department (D R Terefa MSc), Wollega University, Bedele Town, Ethiopia; Department of Pharmacology (P Thangaraju MD), All India Institute of Medical Sciences, Raipur, India; Department of Family and Preventive Medicine (S Thirunavukkarasu PhD), Emory University, Atlanta, GA, USA; Department of Global Health (Prof J Thomas PhD), Sustainable Policy Solutions Foundation, South Yatta, VIC, Australia; Director (Prof J

Thomas PhD), Australian Institute of Community and Health Management, South Yarra, VIC, Australia; Public Health Department (T Y Tiruye PhD), Debre Markos University, Debre Markos, Ethiopia; Laboratory of Public Health Indicators Analysis and Health Digitalization (M V Titova PhD), Moscow Institute of Physics and Technology, Moscow, Russia; Department of Medicine (Prof M Tonelli MD), University of Calgary, Calgary, AB, Canada; Nutritional Epidemiology Research Team (EREN) (M Touvier PhD), National Institute for Health and Medical Research (INSERM), Paris, France; High Institute of Sport and Physical Education of Sfax (K Trabelsi PhD), University of Sfax, Sfax, Tunisia; Department of Business Analytics (T H Tran MD), University of Massachusetts Dartmouth, Dartmouth, MA, USA; Molecular Neuroscience Research Center (N Tran Minh Duc MD), Shiga University of Medical Science, Shiga, Japan; Department of Clinical and Experimental Medicine (D Trico MD), University of Pisa, Pisa, Italy; Department of Internal Medicine (M Tumurkhuu PhD), Wake Forest University, Winston-Salem, NC, USA; International Center for Chemical and Biological Sciences (S Ullah MSc), University of Karachi, Karachi, Pakistan; Federal University of Health Sciences Azare (L Umar PhD), Federal Teaching Hospital, Azare, Nigeria; Federal Teaching Hospital Azare (L Umar PhD), Federal Medical Centre, Azare, Nigeria; Department of Cardiovascular, Endocrine-metabolic Diseases and Aging (B Unim PhD), National Institute of Health, Rome, Italy; Amity Institute of Biotechnology (E Upadhyay PhD), Amity University Rajasthan, Jaipur, India; College of Sciences (D Ustunsoz BS), Louisiana State University and A&M College, Baton Rouge, LA, USA; Department of Epidemiology and Global Health (M Vaezghasemi PhD), Umeå University, Umeå, Sweden; UKK Institute, Tampere, Finland (Prof T J Vasankari PhD); Faculty of Medicine and Health Technology (Prof T J Vasankari PhD), Tampere University, Tampere, Finland; Department of Human Genetics & Molecular Biology (B Vellingiri PhD), Bharathiar University, Coimbatore, India; Raffles Neuroscience Centre (Prof N Venketasubramanian MSc), Raffles Hospital, Singapore, Singapore; Center for Disease and Control Programs (Prof V E Villalobos-Daniel PhD), Ministry of Health, Mexico City, Mexico; Department of Molecular Epidemiology (S K Vladimirov PhD), Research Institute for Systems Biology and Medicine, Moscow, Russia; Department of Information Technologies and Management (S K Vladimirov PhD), Moscow Institute of Physics and Technology, Dolgoprudny, Russia; Department of Health Care Administration and Economics (Prof V Vlassov MD), National Research University Higher School of Economics, Moscow, Russia; GBD Collaborating Unit (Prof S E Vollset DrPH), Norwegian Institute of Public Health, Bergen, Norway; Department of Pediatric Endocrinology (R Vukovic PhD), Mother and Child Healthcare Institute of Serbia "Dr Vukan Cupic", Belgrade, Serbia; Faculty of Public Health (M Wahiduzzaman PhD), Bangladesh University of Health Sciences (BUHS), Dhaka, Bangladesh; Department of Cardiology (M Wahiduzzaman PhD), National Healthcare Network-Diabetic Association of Bangladesh, Dhaka, Bangladesh; Brigham and Women's Hospital, Boston, MA, USA (C Wang PhD); Department of Neurosurgery (S Wang MD), Beijing Tiantan Hospital, Beijing, China; Shandong University of Traditional Chinese Medicine (X Wang MD), Shandong University of Traditional Chinese Medicine, Jinan, China; Department of Orthopaedics (F Wei PhD), General Hospital of Central Theater Command (Wuhan General Hospital of Guangzhou Command, previously), Wuhan, China; Fourth Military Medical University, Xi'an, China (F Wei PhD); Institute of Clinical Epidemiology, Public Health, Health Economics, Medical Statistics and Informatics (Prof P Willeit PhD), Medical University Innsbruck, Innsbruck, Austria; Department of Chemical Toxicology (M W Wojewodzic PhD), Norwegian Institute of Public Health, Oslo, Norway; Australian Centre for Health Services Innovation (Q Xia PhD), Queensland University of Technology, Brisbane, QLD, Australia; Tongji Medical College (G Xiao MD), Huazhong University of Science and Technology, Wuhan, China; Department of Intelligent Medical Engineering (Prof W Xie DrPH), Anhui Medical University, Anhui, China; Department of Endocrinology (Prof S Xu PhD), University

of Science and Technology of China, Hefei, China; Cardiovascular Program (X Xu PhD), The George Institute for Global Health, Sydney, NSW, Australia; Department of Cells and Tissues (G Yahya PhD), Molecular Biology Institute of Barcelona, Barcelona, Spain; Department of Public Health (Prof K Yamagishi MD, Prof N Yonemoto PhD), Faculty of Medicine (Y Yano MD), Juntendo University, Tokyo, Japan; School of Traditional Chinese Medicine (Prof H Yao PhD), Beijing University of Chinese Medicine, Beijing, China; Pritzker School of Medicine (Prof H Yao PhD), University of Chicago, Chicago, IL, USA; Research Center of Physiology (H Yaribeygi PhD), Semnan University of Medical Sciences, Semnan, Iran; Department of Family Medicine (S A Yesuf MSc), St. Paul's Hospital Millennium Medical College, Addis Ababa, Ethiopia; Independent Consultant, Addis Ababa, Ethiopia (S A Yesuf MSc); Department of Epidemiology (D Yin DrPH), Xuzhou Medical University, Xuzhou, China; Department of Biostatistics (Prof N Yonemoto PhD), University of Toyama, Toyama, Japan; Cancer Institute (D Yuce MD), Hacettepe University, Ankara, Turkiye; Department of Clinical Pharmacy and Outcomes Sciences (I Yunusa PhD), University of South Carolina, Columbia, SC, USA; Department of Administration (Prof M Zastrozhin PhD), PGxAI, San Francisco, CA, USA; Department of Zoology and Entomology (M G M Zeariya PhD), Al-Azhar University, Cairo, Egypt; School of Public Policy and Administration (J Zhang BA), Xi 'an Jiaotong University, Xi'an, China; Medical Oncology Department of Gastrointestinal Cancer (L Zhang MS), Cancer Hospital of Dalian University of Technology, Shenyang, China; School of Biomedical Engineering (L Zhang MS), Dalian University of Technology, Dalian, China; Department of Internal Medicine (X Zhang MD), Graduate Medical Education (X Zhang MD), Albert Einstein College of Medicine, Bronx, NY, USA; Tianjin Medical University General Hospital (Z Zhang MD), Tianjin Centers for Disease Control and Prevention, Tianjin, China; College of Traditional Chinese Medicine (H Zhao MD), Hebei University, Baoding, China; Jockey Club School of Public Health and Primary Care (C Zhong PhD), The Chinese University of Hong Kong, Hong Kong, China; School of Medicine (J Zhou PhD), Stanford University, Palo Alto, CA, USA; School of Public Health and Emergency Management (B Zhu PhD), Southern University of Science and Technology, Shenzhen, China; College of Medicine (O A Zitoun MD), Sulaiman Alrajhi University, Al Bukairiyah, Saudi Arabia; Endocrinology and Metabolism Research Center (G Zoghi MD), Hormozgan University of Medical Sciences, Bandar Abbas, Iran; Clinical Research Centre (Prof S H Zyoud PhD), An-Najah National University Hospital, Nablus, Palestine.

Authors’ Contributions

Providing data or critical feedback on data sources

Yohannes Habtegiorgis Abate, Abdallah H A Abd Al Magied, Samar Abd ElHafeez, Rizwan Suliankatchi Abdulkader, Auwal Abdullahi, Shady Abohashem, Hana J Abukhadijah, Niveen ME Abu-Rmeileh, Ahmed Abu-Zaid, Mesafint Molla Adane, Kamoru Ademola Adedokun, Usha Adiga, Qorinah Estiningtyas Sakilah Adnani, Abdelrahman Yousry Afify, Saira Afzal, Muhammad Sohail Afzal, Bright Opoku Ahinkorah, Danish Ahmad, Noah Ahmad, Sajjad Ahmad, Ayman Ahmed, Haroon Ahmed, Mehrunnisha Sharif Ahmed, Meqdad Saleh Ahmed, Hanadi Al Hamad, Zain Al Ta'ani, Rasmieh Mustafa Al-amer, Mohammed Albashtawy, Abdelazeem M Algammal, Khalid F Alhabib, Dari Alhuwail, Abid Ali, Waad Ali, Sheikh Mohammad Alif, Samah W Al-Jabi, Mohamad Aljofan, Syed Mohamed Aljunid, Wael Almahmeed, Intima Alrimawi, Awais Altaf, Nelson Alvis-Guzman, Mohammad Al-Wardat, Hubert Amu, Sumbul Ansari, Saeid Anvari, Sumadi Lukman Anwar, Juan Pablo Arab, Jalal Arabloo, Hidayat Arifin, Benedetta Armocida, Johan Ärnlöv, Amit Arora, Tahira Ashraf, Seyyed Shamsadin Athari, Prince Atorkey, Alok Atreya, Zaure Maratovna Aumoldaeva, Adedapo Wasiu Awotidebe, Setognal Birara Aychiluhm, Ahmed Y. Azzam, Domenico Azzolino, Mina Babashahi, Giridhara Rathnaiah Babu, Atif Amin Baig, Senthilkumar Balakrishnan, Ovidiu Constantin Baltatu, Rajon Banik, Mainak Bardhan, Hiba Jawdat Barqawi, Amadou Barrow, Mohammad-Mahdi Bastan, Mulat Tirfie Bayih, Nebiyou Simegnew Bayleyegn, Narasimha M Beeraka, Diana Fernanda Bejarano Ramirez, Ravi Bharadwaj, Sonu Bhaskar, Ajay Nagesh Bhat, Priyadarshini Bhattacharjee, Jasvinder Singh Bhatti, Gurjit Kaur Bhatti, Cem Bilgin, Bijit Biswas, Espen Bjertness, Archith Boloor, Sri Harsha Boppana, Souad Bouaoud, Dejana Braithwaite, Dana Bryazka, Luciana Aparecida Campos, Claudia Carletti, Monica Cattafesta, Francieli Cembranel, Joshua Chadwick, Vijay Kumar Chattu, Akhilanand Chaurasia, Nicholas WS Chew, Ritesh Chimoriya, Abdulaal Chitheer, Bryan Chong, Chean Lin Chong, Hitesh Chopra, Shivani Chopra, Hou In Chou, Sunghyun Chung, Alyssa Columbus, Nathalie Conrad, Michael H Criqui, Natalia Cruz-Martins, Xiaochen Dai, Rakhi Dandona, Lalit Dandona, Lucio D'Anna, Samuel Demissie Darcho, Reza Darvishi Cheshmeh Soltani, Meseret Derbew Molla, Ismail Dergaa, Vinoth Gnana Chellaiyan Devanbu, Devananda Devegowda, Thanh Chi Do, Klara Georgieva Dokova, Paul Narh Doku, Fariba - Dorostkar, Ojas Prakashbhai Doshi, Rajkumar Prakashbhai Doshi, Robert Kokou Dowou, Bruce B Duncan, Michael Ekholuenetale, Rabie Adel El Arab, Ibrahim Farahat El Bayoumy, Ashraf A El-Metwally, Mohamed A Elmonem, Chadi Eltaha, Heidar Fadavian, Adeniyi Francis Fagbamigbe, Ildar Ravisovich Fakhradiyev, Carla Sofia e Sá Farinha, Hossein Farrokhpour, Timur Fazylov, Alireza Feizkhah, Morenike Oluwatoyin Folayan, Muktar A Gadanya, Emmanuela Gakidou, Yaseen Galali, Silvano Gallus, Balasankar Ganesan, Lemma Getacher, Molla Getie, Alem Abera Girmay, Mahaveer Golechha, Pouya Goleij, Ayman Grada, Guha, Rajeev Gupta, Najah R Hadi, Zahra Hadian, Nadia M Hamdy, Nasrin Hanifi, Allie Haq, Josep Maria Haro, Faizul Hasan, Soheil Hassanipour, Rasmus J Havmoeller, Jeffrey J Hebert, Mai Hoang, Mohamed Ibrahim Husseiny, Hong-Han Huynh, Ramzi Ibrahim, Nayu Ikeda, Olayinka Stephen Ilesanmi, Sheikh Mohammed Shariful Islam, Md Sahidul Islam, Nahlah Elkudssiah Ismail, Gaetano Isola, Haitham Jahrami, Rajesh Jain, Mihajlo Jakovljevic, Syed Sarmad Javaid, Sathish Kumar Jayapal, Shubha Jayaram, Bijay Mukesh Jeswani, Jost B Jonas, Charity Ehimwenma Joshua, Jacek Jerzy Jozwiak, Mikk Jürisson, Billingsley Kaambwa, Vidya Kadashetti, Sanjay Kalra, Samuel Berchi Kankam, Nicholas J Kassebaum, Gbenga A Kayode, Shemsu Kedir, Dimitrios Kehagias, Jessica A Kerr, Ariz Keshwani, Himanshu Khajuria, Maseer Khan, Moien AB Khan, Muhammad Shahzeb Khan, Masoomeh Kheirkhah, Feriha Fatima Khidri, Atulya Aman Khosla, Min Seo Kim, Jihee Kim, Yun Jin Kim, Gyu Ri Kim, Adnan Kisa, Ladli Kishore, Shivakumar KM, Michail Kokkorakis, Oleksii Korzh, Sindhura Lakshmi Koulmane Laxminarayana, Irene Akwo Kretchy, Chong-Han Kua, Barthelemy Kuate Defo, Vijay Kumar, G Anil

Kumar, Satyajit Kundu, Dian Kusuma, Ville Kytö, Chandrakant Lahariya, Nhi Huu Hanh Le, Munjae Lee, Seung Won Lee, Wei-Chen Lee, Yongze Li, Stephen S Lim, Queran Lin, Jue Liu, Stefan Lorkowski, Alessandra Lugo, Zheng Feei Ma, Nozad H. Mahmood, Kashish Malhotra, Deborah Carvalho Malta, Winfried März, Sammer Marzouk, Thushara Matthias, Rita Mattiello, Andrea Maugeri, Steven M McPhail, Enkeleint A Mechili, Asim Mehmood, Tesfahun Mekene Meto, Walter Mendoza, Ritesh G Menezes, Sachith Mettananda, Ted R Miller, Erkin M Mirrakhimov, Awoke Misganaw, Mona Gamal Mohamed, Nouh Saad Mohamed, Abdollah Mohammadian-Hafshejani, Shafiu Mohammed, Ali H Mokdad, Lorenzo Monasta, Sara Montazeri Namin, Yousef Moradi, Rohith Motappa, Sumaira Mubarik, Francesk Mulita, Yanjinlkham Munkhsaikhan, Efren Murillo-Zamora, Christopher J L Murray, Julius C Mwita, Gabriele Nagel, Ganesh R Naik, Gopal Nambi, Shumaila Nargus, Biswa Prakash Nayak, Masoud Negahdary, Ruxandra Irina Negoi, Ionut Negoi, Henok Biresaw Netsere, Josephine W Ngunjiri, Dang Nguyen, Phat Tuan Nguyen, Robina Khan Niazi, Luciano Nieddu, Taxiarchis Konstantinos Nikolouzakis, Shuhei Nomura, Syed Toukir Ahmed Noor, Mamoona Noreen, Jean Jacques Noubiap, Mehran Nouri, Fred Nugen, Aqsha Nur, Bogdan Oancea, Ismail A Odetokun, Akinkunmi Paul Okekunle, Andrew T Olagunju, Oladotun Victor Olalusi, Tosin Abiola Olasehinde, Yinka Doris Oluwafemi, Hany A Omar, Ahmed Omar Bali, Sok King Ong, Abdu Oumer, Amel Ouyahia, Mayowa O Owolabi, Mahesh Padukudru P A, Jagadish Rao Padubidri, Sujogya Kumar Panda, Songhomitra Panda-Jonas, Anamika Pandey, Seithikurippu R Pandi-Perumal, Shahina Pardhan, Romil R Parikh, Eun-Cheol Park, Jay Patel, Prince Peprah, Simone Perna, Hoang Nhat Pham, Saeed Pirouzpanah, Jalandhar Pradhan, Elton Junio Sady Prates, Jagadeesh Puvvula, Venkatraman Radhakrishnan, Masoud Rahmati, Jeffrey Pradeep Raj, Mahmoud Mohammed Ramadan, Chitra Ramasamy, Shakthi Kumaran Ramasamy, Sheena Ramazanu, Kritika Rana, Chhabi Lal Ranabhat, Mithun Rao, Sowmya J Rao, Sina Rashedi, Santosh Kumar Rauniyar, Ilari Rautalin, Salman Rawaf, Jefferson Antonio Buendia Rodriguez, Leonardo Roever, Peter Rohloff, Debby Syahru Romadlon, Moustaq Karim Khan Rony, Godfrey M Rwegerera, Aly M A Saad, Kabir P Sadarangani, Basema Ahmad Saddik, Umar Saeed, S. Mohammad Sajadi, Mirza Rizwan Sajid, Afeez Abolarinwa Salami, Samreen Saleem, Marwa Rashad Salem, Abdallah M Samy, Milena M Santric-Milicevic, Monika Sawhney, Sonia Saxena, Maria Inês Schmidt, Siddharthan Selvaraj, Yigit Can Senol, Subramanian Senthilkumaran, Yashendra Sethi, Allen Seylani, Samiah Shahid, Muhammad Aaqib Shamim, Mehran Shams-Beyranvand, Mohammed Shannawaz, Medha Sharath, Amin Sharifan, Ujjawal Sharma, Vishal Sharma, Aminu Shittu, Sina Shool, Gambhir Shrestha, Luís Manuel Lopes Rodrigues Silva, Jasvinder A Singh, Baljinder Singh, Harmanjit Singh, Kalpana Singh, Anna Aleksandrovna Skryabina, Georgia Smith, Michael Spartalis, Muhammad Haroon Stanikzai, Vetriselvan Subramaniyan, Anusha Sultan Meo, Chandan Kumar Swain, Lukasz Szarpak, Sree Sudha T Y, Rafael Tabarés-Seisdedos, Seyyed Mohammad Tabatabaei, Shima Tabatabai, Celine Tabche, Jabeen Taiba, Mircea Tampa, Ker-Kan Tan, Manoj Tanwar, Dufera Rikitu Terefa, Jay Tewari, Pugazhenthan Thangaraju, Joe Thomas, Mariya Vladimirovna Titova, Krishna Tiwari, Marcello Tonelli, Mathilde Touvier, Marcos Roberto Tovani-Palone, Nguyen Tran Minh Duc, Domenico Trico, Guesh Mebrahtom Tsegay, Munkhtuya Tumurkhuu, Shahid Ullah, Muhammad Umair, Era Upadhyay, Jibrin Sammani Usman, Jef Van den Eynde, Joe Varghese, Tommi Juhani Vasankari, Balachandar Vellingiri, Narayanaswamy Venketasubramanian, Georgios-Ioannis Verras, Sergey Konstantinovitch Vladimirov, Vasily Vlassov, Stein Emil Vollset, Shu Wang, Xingxin Wang, Anggi Lukman Wicaksana, Dakshitha Praneeth Wickramasinghe, Peter Willeit, Suowen Xu, Galal Yahya, Pengpeng Ye, Subah Abderehim Yesuf, Naohiro Yonemoto, Chuanhua Yu, Ismaeel Yunusa, Michael Zastrozhin, Xiaoyi Zhang, Jingya Zhang, Anthony Zhong, Abzal Zhumagaliuly, Magdalena Zielińska

Developing methods or computational machinery

Austin J Ahlstrom, Noah Ahmad, Aleksandr Y Aravkin, Dana Bryazka, Rebecca M Cogen, Xiaochen Dai, Emmanuela Gakidou, Nicholas J Kassebaum, Stephen S Lim, Justin Lo, Ali H Mokdad, Christopher J L Murray, Catalina Raggi, Stein Emil Vollset, Chun-Wei Yuan

Providing critical feedback on methods or results

Yohannes Habtegiorgis Abate, Nasir Abbas, Samar Abd ElHafeez, Sherief Abd-Elsalam, Arash Abdollahi, Meriem Abdoun, Deldar Morad Abdulah, Rizwan Suliankatchi Abdulkader, Auwal Abdullahi, Alemwork Abie, Shady Abohashem, Dariush Abtahi, Bilyaminu Abubakar, Eman Abu-Gharbieh, Hana J Abukhadijah, Salahdein Aburuz, Ahmed Abu-Zaid, Lisa C. Adams, Mesafint Molla Adane, Isaac Yeboah Addo, Kamoru Ademola Adedokun, Nurudeen A Adegoke, Ridwan Olamilekan Adesola, Temitayo Esther Adeyeoluwa, Usha Adiga, Qorinah Estiningtyas Sakilah Adnani, Abdelrahman Yousry Afify, Aanuoluwapo Adeyimika Afolabi, Saira Afzal, Muhammad Sohail Afzal, Suneth Buddhika Agampodi, Shahin Aghamiri, César Agostinis Sobrinho, Williams Agyemang-Duah, Bright Opoku Ahinkorah, Aqeel Ahmad, Danish Ahmad, Fuzail Ahmad, Muayyad M Ahmad, Sajjad Ahmad, Ayman Ahmed, Haroon Ahmed, Luai A Ahmed, Mehrunnisha Sharif Ahmed, Syed Anees Ahmed, Ashley E Akrami, Hanadi Al Hamad, Syed Mahfuz Al Hasan, Zain Al Ta'ani, Yazan Al Thaher, Ziyad Al-Aly, Khurshid Alam, Rasmieh Mustafa Al-amer, Amani Alansari, Fahmi Y. Al-Ashwal, Mohammed Albashtawy, Bezawit Abeje Alemayehu, Abdelazeem M Algammal, Khalid F Alhabib, Dari Alhuwail, Abid Ali, Endale Alemayehu Ali, Mohammad Daud Ali, Mohammed Usman Ali, Rafat Ali, Waad Ali, Sheikh Mohammad Alif, Yousef Alimohamadi, Samah W Al-Jabi, Syed Mohamed Aljunid, Ahmad Alkhatib, Wael Almahmeed, Sabah Al-Marwani, Mahmoud A Alomari, Saleh A Alqahtani, Intima Alrimawi, Najim Z Alshahrani, Zaid Altaany, Awais Altaf, Farrukh Jawad Alvi, Nelson Alvis-Guzman, Mohammad Al-Wardat, Yaser Mohammed Al-Worafi, Safwat Aly, Hany Aly, Karem H Alzoubi, Masoud Aman Mohammadi, Tewodros Getnet Amera, Sohrab Amiri, Hubert Amu, Dickson A Amugsi, Ganiyu Adeniyi Amusa, Roshan A Ananda, Robert Ancuceanu, Mohammed Tahir Ansari, Sumbul Ansari, Boluwatife Stephen Anuoluwa, Iyadunni Adesola Anuoluwa, Saeid Anvari, Sumadi Lukman Anwar, Anayochukwu Edward Anyasodor, Juan Pablo Arab, Jalal Arabloo, Mosab Arafat, Demelash Areda, Brhane Berhe Aregawi, Hidayat Arifin, Benedetta Armocida, Mahwish Arooj, Amit Arora, Anton A Artamonov, Kurnia Dwi Artanti, Ashokan Arumugam, Mohammad Asghari-Jafarabadi, Tahira Ashraf, Bernard Kwadwo Yeboah Asiamah-Asare, Thomas Astell-Burt, Seyyed Shamsadin Athari, Prince Atorkey, Alok Atreya, Mamaru Ayenew Awoke, Setognal Birara Aychiluhm, Amirali Azimi, Sadat Abdulla Aziz, Shahkaar Aziz, Ahmed Y. Azzam, Domenico Azzolino, Peter S Azzopardi, Mina Babashahi, Giridhara Rathnaiah Babu, Alaa Aboelnour Badran, Nasser Bagheri, Ruhai Bai, Atif Amin Baig, Senthilkumar Balakrishnan, Ovidiu Constantin Baltatu, Rajon Banik, Shirin Barati, Mainak Bardhan, Hiba Jawdat Barqawi, Simon Barquera, Amadou Barrow, Lingkan Barua, Mohammad-Mahdi Bastan, Saurav Basu, Reza Bayat, Mulat Tirfie Bayih, Nebiyou Simegnew Bayleyegn, Narasimha M Beeraka, Priyamadhaba Behera, Umar Muhammad Bello, Derrick A Bennett, Kidanemaryam Berhe, Abiye Assefa Berihun, Ajeet Singh Bhadoria, Neeraj Bhala, Ravi Bharadwaj, Pankaj Bhardwaj, Nikha Bhardwaj, Sonu Bhaskar, Ajay Nagesh Bhat, Priyadarshini Bhattacharjee, Jasvinder Singh Bhatti, Gurjit Kaur Bhatti, Cem Bilgin, Bijit Biswas, Bruno Bizzozero Peroni, Espen Bjertness, Archith Boloor, Sri Harsha Boppana, Samuel Adolf Bosoka, Souad Bouaoud, Edward J Boyko, Dejana Braithwaite, Javier Brazo-Sayavera, Hermann Brenner, Dana Bryazka, Raffaele Bugiardini, Linh Phuong Bui, Yasser Bustanji, Nadeem Shafique Butt, Zahid A Butt, Mehtap Çakmak Barsbay, Luis Alberto Cámera, Luciana Aparecida Campos, Si Cao, Andre F Carvalho, Monica Cattafesta, Luca Cegolon, Francieli Cembranel, Ester Cerin, Achille Cernigliaro, Joshua Chadwick, Chiranjib Chakraborty, Eeshwar K Chandrasekar, Jung-Chen Chang, Vijay Kumar Chattu,

Akhilanand Chaurasia, Haowei Chen, An-Tian Chen, Nicholas WS Chew, Gerald Chi, Ritesh Chimoriya, Dong-Woo Choi, Bryan Chong, Hitesh Chopra, Shivani Chopra, Hou In Chou, Sonali Gajanan Choudhari, Sunghyun Chung, Sheng-Chia Chung, Muhammad Chutiyami, Karly I Cini, Alyssa Columbus, Nathalie Conrad, Michael H Criqui, Natalia Cruz-Martins, Alanna Gomes da Silva, Omid Dadras, Xiaochen Dai, Mayank Dalakoti, Lucio D'Anna, Pojsakorn Danpanichkul, Samuel Demissie Darcho, Reza Darvishi Cheshmeh Soltani, Nihar Ranjan Dash, Kairat Davletov, Azizallah Dehghan, Meseret Derbew Molla, Ismail Dergaa, Aragaw Tesfaw Desale, Vinoth Gnana Chellaiyan Devanbu, Devananda Devegowda, Arkadeep Dhali, Bibha Dhungel, Daniel Diaz, Thanh Chi Do, Camila Bruneli do Prado, Milad Dodangeh, Paul Narh Doku, Neda Dolatkhah, Mario D'Oria, Fariba - Dorostkar, Ojas Prakashbhai Doshi, Rajkumar Prakashbhai Doshi, Robert Kokou Dowou, Mi Du, Dorothea Dumuid, Sulagna Dutta, Alireza Ebrahimi, Kristina Edvardsson, Ashkan Eighaei Sedeh, Michael Ekholuenetale, Rabie Adel El Arab, Ibrahim Farahat El Bayoumy, Mohamed Ahmed Eladl, Said El-Ashker, Islam Y Elgendy, Muhammed Elhadi, Ashraf A El-Metwally, Mohamed Hassan Elnaem, Randa Elsheikh, Chadi Eltaha, Theophilus I Emeto, Natalia Fabin, Heidar Fadavian, Adeniyi Francis Fagbamigbe, Ildar Ravisovich Fakhradiyev, Seyed Nooreddin Faraji, Carla Sofia e Sá Farinha, MoezAlIslam Ezzat Mahmoud Faris, Pawan Sirwan Faris, Umar Farooque, Hossein Farrokhpour, Samuel Aanuoluwapo Fasusi, Patrick Fazeli, Alireza Feizkhah, Ginenus Fekadu, Xiaoqi Feng, Rodrigo Fernandez-Jimenez, Nataliya A Foigt, Artem Alekseevich Fomenkov, Roham Foroumadi, Celia Fortuna Rodrigues, Matteo Foschi, Kate Louise Francis, Richard Charles Franklin, Aleš Gába, Muktar A Gadanya, Abhay Motiramji Gaidhane, Emmanuela Gakidou, Yaseen Galali, Balasankar Ganesan, Miglas Welay Gebregergis, Mesfin Gebrehiwot, Lemma Getacher, Molla Getie, Ramy Mohamed Ghazy, Tiffany K Gill, Alem Abera Girmay, Mahaveer Golechha, Alessandra C Goulart, Ayman Grada, Michal Grivna, Ashna Grover, Zhongyang Guan, Mohammed Ibrahim Mohialdeen Gubari, Avirup Guha, Damitha Asanga Gunawardane, Zheng Guo, Vipin Gupta, Bhawna Gupta, Rahul Gupta, Roberth Steven Gutiérrez-Murillo, Jose Guzman-Esquivel, Najah R Hadi, Zahra Hadian, Nadia M Hamdy, Samer Hamidi, Asif Hanif, Nasrin Hanifi, Allie Haq, Faizul Hasan, Md Saquib Hasnain, Soheil Hassanipour, Afagh Hassanzade Rad, Rasmus J Havmoeller, Simon I Hay, Kamal Hezam, Yuta Hiraike, Mai Hoang, Ramesh Holla, Alamgir Hossain, Hassan Hosseinzadeh, Mihaela Hostiuc, Zin Wai Htay, Yifei Hu, Mengsi Hu, Ayesha Humayun, Tsegaye Gebreyes Hundie, Mohamed Ibrahim Husseiny, Foziya Mohammed Hussien, Hong-Han Huynh, Bing-Fang Hwang, Ramzi Ibrahim, Olayinka Stephen Ilesanmi, Irena M Ilic, Milena D Ilic, Leeberk Raja Inbaraj, Arit Inok, Khalid Iqbal, Md. Rabiul Islam, Sheikh Mohammed Shariful Islam, Md. Fakrul Islam, Md Sahidul Islam, Nahlah Elkudssiah Ismail, Gaetano Isola, Mosimah Charles Ituka, Masao Iwagami, Chinwe Juliana Iwu-Jaja, Assefa N Iyasu, Louis Jacob, Shabbar Jaffar, Haitham Jahrami, Akhil Jain, Ammar Abdulrahman Jairoun, Mihajlo Jakovljevic, Syed Sarmad Javaid, Sathish Kumar Jayapal, Shubha Jayaram, Sun Ha Jee, Alelign Tasew Jema, Bijay Mukesh Jeswani, Jost B Jonas, Nitin Joseph, Charity Ehimwenma Joshua, Jacek Jerzy Jozwiak, Mikk Jürisson, Billingsley Kaambwa, Ali Kabir, Vidya Kadashetti, Ashish Kumar Kakkar, Sanjay Kalra, Saddam Fuad Kanaan, Samuel Berchi Kankam, Arun R Kanmanthareddy, Kehinde Kazeem Kanmodi, Rami S Kantar, Debasish Kar, Mehrdad Karajizadeh, Paschalis Karakasis, Arman Karimi Behnagh, Sahand Karimzadhagh, Nicholas J Kassebaum, Joonas H Kauppila, Gbenga A Kayode, Shemsu Kedir, Dimitrios Kehagias, Jessica A Kerr, Ariz Keshwani, Mohammad Keykhaei, Himanshu Khajuria, Pantea Khalili, Alireza Khalilian, Mohamed Khalis, Ajmal Khan, Maseer Khan, Md Abdullah Saeed Khan, Mohammad Jobair Khan, Moien AB Khan, Muhammad Shahzeb Khan, Nusrat Khan, Vishnu Khanal, Shaghayegh Khanmohammadi, Moawiah Mohammad Khatatbeh, Masoomeh Kheirkhah, Feriha Fatima Khidri, Manoj Khokhar, Atulya Aman Khosla, Helda Khusun, Jinho Kim, Min Seo Kim, Jihee Kim, Yun Jin Kim, Gyu Ri Kim, Ruth W Kimokoti, Adnan Kisa, Ladli Kishore, Shivakumar KM, Michail Kokkorakis, Farzad Kompani, Oleksii Korzh, Sindhura Lakshmi

Koulmane Laxminarayana, Irene Akwo Kretchy, Chong-Han Kua, Barthelemy Kuate Defo, Mohammed Kuddus, Vijay Kumar, Satyajit Kundu, Setor K Kunutsor, Om P Kurmi, Maria Dyah Kurniasari, Dian Kusuma, Ville Kytö, Carlo La Vecchia, Chandrakant Lahariya, Daphne Teck Ching Lai, Iván Landires, Bagher Larijani, Nhi Huu Hanh Le, Huyen Thi Thanh Le, Hye Ah Lee, Munjae Lee, Seung Won Lee, Wei-Chen Lee, An Li, Ming-Chieh Li, Wei Li, Yongze Li, Stephen S Lim, Jialing Lin, Queran Lin, Daniel Lindholm, Jue Liu, Justin Lo, José Francisco López-Gil, Stefan Lorkowski, Giancarlo Lucchetti, Zheng Feei Ma, Nastaran Maghbouli, Mehrdad Mahalleh, Nozad H. Mahmood, Azeem Majeed, Konstantinos Christos C. Makris, Elaheh Malakan Rad, Reza Malekzadeh, Kashish Malhotra, Ahmad Azam Malik, Iram Malik, Deborah Carvalho Malta, Abdullah A Mamun, Emmanuel Manu, Hamid Reza Marateb, Mirko Marino, Ramon Martinez-Piedra, Miquel Martorell, Winfried März, Sammer Marzouk, Soroush Masrouri, Yasith Mathangasinghe, Fernanda Penido Matozinhos, Thushara Matthias, Rita Mattiello, Andrea Maugeri, Mohsen Mazidi, Steven M McPhail, Enkeleint A Mechili, Asim Mehmood, Kamran Mehrabani-Zeinabad, Tesfahun Mekene Meto, Hadush Negash Meles, Walter Mendoza, Ritesh G Menezes, Emiru Ayalew Mengistie, Sultan Ayoub Meo, Tomislav Mestrovic, Sachith Mettananda, Chamila Dinushi Kukulege Mettananda, Huanhuan Miao, Ted R Miller, Wai-kit Ming, Erkin M Mirrakhimov, Habtamu Mitiku, Madhukar Mittal, Jama Mohamed, Mona Gamal Mohamed, Nouh Saad Mohamed, Sakineh Mohammad-Alizadeh-Charandabi, Abdollah Mohammadian-Hafshejani, Ibrahim Mohammadzadeh, Shafiu Mohammed, Ali H Mokdad, Stefania Mondello, Mohammad Ali Moni, Sara Montazeri Namin, AmirAli Moodi Ghalibaf, Yousef Moradi, Shane Douglas Morrison, Rohith Motappa, Sumaira Mubarik, Francesk Mulita, Yanjinlkham Munkhsaikhan, Efren Murillo-Zamora, Christopher J L Murray, Ghulam Mustafa, Sathish Muthu, Julius C Mwita, Gabriele Nagel, Ganesh R Naik, Hiten Naik, Gopal Nambi, Vinay Nangia, Shumaila Nargus, Mahmoud Nassar, Javaid Nauman, Zakira Naureen, Nawsherwan, Biswa Prakash Nayak, Athare Nazri-Panjaki, Masoud Negahdary, Ruxandra Irina Negoi, Ionut Negoi, Soroush Nematollahi, Henok Biresaw Netsere, Josephine W Ngunjiri, Dang Nguyen, Phuong The Nguyen, Phat Tuan Nguyen, Robina Khan Niazi, Luciano Nieddu, Taxiarchis Konstantinos Nikolouzakis, Ali Nikoobar, Shuhei Nomura, Syed Toukir Ahmed Noor, Mamoona Noreen, Jean Jacques Noubiap, Mehran Nouri, Chisom Adaobi Nri-Ezedi, Fred Nugen, Virginia Nuñez-Samudio, Aqsha Nur, Felix Kwasi Nyande, Chimezie Igwegbe Nzoputam, Bogdan Oancea, Ismail A Odetokun, Akinyemi O D Ofakunrin, James Odhiambo Oguta, Hassan Okati-Aliabad, Akinkunmi Paul Okekunle, Osaretin Christabel Okonji, Andrew T Olagunju, Oladotun Victor Olalusi, Tosin Abiola Olasehinde, Arão Belitardo Oliveira, Yinka Doris Oluwafemi, Hany A Omar, Ahmed Omar Bali, Nesredin Ahmed Omer, Michal Ordak, Wael M S Osman, Adrian Otoiu, Abdu Oumer, Amel Ouyahia, Mayowa O Owolabi, Kolapo Oyebola, Mahesh Padukudru P A, Jagadish Rao Padubidri, Tamás Palicz, Sujogya Kumar Panda, Songhomitra Panda-Jonas, Suman Pant, Shahina Pardhan, Utsav Parekh, Pragyan Paramita Parija, Romil R Parikh, Eun-Cheol Park, Roberto Passera, Jay Patel, Dimitrios Patoulias, George C Patton, Susan Paudel, Prince Peprah, Marcos Pereira, Ionela-Roxana Petcu, Fanny Emily Petermann-Rocha, Tung Thanh Pham, Hoang Nhat Pham, Saeed Pirouzpanah, Roman V Polibin, Farzad Pourghazi, Akram Pourshams, Jalandhar Pradhan, Pranil Man Singh Pradhan, Manya Prasad, Akila Prashant, Elton Junio Sady Prates, Jagadeesh Puvvula, Ibrahim Qattea, Yanan Qiao, Venkatraman Radhakrishnan, Maja R Radojčić, Muhammad Aziz Rahman, Fryad Majeed Rahman, Md. Mosfequr Rahman, Mohammad Hifz Ur Rahman, Mosiur Rahman, Vahid Rahmanian, Mohammad Rahmanian, Masoud Rahmati, Rajesh Kumar Rai, Jeffrey Pradeep Raj, Prashant Rajput, Mahmoud Mohammed Ramadan, Chitra Ramasamy, Shakthi Kumaran Ramasamy, Kritika Rana, Mithun Rao, Sowmya J Rao, Sina Rashedi, Mohammad-Mahdi Rashidi, Ashkan Rasouli-Saravani, Devarajan Rathish, Santosh Kumar Rauniyar, Ilari Rautalin, David Laith Rawaf, Salman Rawaf, Elrashdy M. Moustafa Mohamed Redwan, Sanika Rege, Aqeeb Ur Rehman, Nazila Rezaei, Mohsen Rezaeian, Taeho Gregory Rhee, João Rocha Rocha-Gomes, Thales Philipe Rodrigues da Silva,

Jefferson Antonio Buendia Rodriguez, Leonardo Roever, Peter Rohloff, Debby Syahru Romadlon, Moustaq Karim Khan Rony, Gholamreza Roshandel, Himanshu Sekhar Rout, Nitai Roy, Godfrey M Rwegerera, Aly M A Saad, Maha Mohamed Saber-Ayad, Kabir P Sadarangani, Basema Ahmad Saddik, Umar Saeed, Fatemeh Saheb Sharif-Askari, Soumya Swaroop Sahoo, S. Mohammad Sajadi, Mirza Rizwan Sajid, Afeez Abolarinwa Salami, Samreen Saleem, Mohamed A Saleh, Marwa Rashad Salem, Sohrab Salimi, Abdallah M Samy, Milena M Santric-Milicevic, Tanmay Sarkar, Mohammad Sarmadi, Sachin C Sarode, Gargi Sachin Sarode, Michele Sassano, Jennifer Saulam, Monika Sawhney, Susan M Sawyer, Sonia Saxena, Ganesh Kumar Saya, Christophe Schinckus, Art Schuermans, Siddharthan Selvaraj, Ashenafi Kibret Sendekie, Pallav Sengupta, Yigit Can Senol, Subramanian Senthilkumaran, Sadaf G Sepanlou, Yashendra Sethi, Mahan Shafie, Sweni Shah, Samiah Shahid, Muhammad Aaqib Shamim, Mehran Shams-Beyranvand, Alfiya Shamsutdinova, Mohd Shanawaz, Mohammed Shannawaz, Medha Sharath, Amin Sharifan, Vishal Sharma, Fateme Sheida, Desalegn Shiferaw, Mahsa Shirani Lapari, Rahman Shiri, Aminu Shittu, Sina Shool, Seyed Afshin Shorofi, Gambhir Shrestha, Rajan Shrestha, Kerem Shuval, Yafei Si, Nicole R S Sibuyi, Emmanuel Edwar Siddig, Diego Augusto Santos Silva, Luís Manuel Lopes Rodrigues Silva, Jasvinder A Singh, Mitasha Singh, Lucky Singh, Amit Singh, Baljinder Singh, Harmanjit Singh, Kalpana Singh, Anna Aleksandrovna Skryabina, Amanda E Smith, Sameh S M Soliman, Soroush Soraneh, Michael Spartalis, Bahadar S Srichawla, Muhammad Haroon Stanikzai, Antonina V Starodubova, Kurt Straif, Pete Stubbs, Vetriselvan Subramaniyan, Muritala Odidi Suleiman Odidi, Aleksander Sulkowski, Anusha Sultan Meo, Zhong Sun, Sumam Sunny, Chandan Kumar Swain, Lukasz Szarpak, Sree Sudha T Y, Rafael Tabarés-Seisdedos, Seyyed Mohammad Tabatabaei, Ozra Tabatabaei Malazy, Shima Tabatabai, Celine Tabche, Mohammad Tabish, Jabeen Taiba, Mircea Tampa, Jacques Lukenze Tamuzi, Ker-Kan Tan, Manoj Tanwar, Mohamad-Hani Temsah, Masayuki Teramoto, Dufera Rikitu Terefa, Jay Tewari, Pugazhenthan Thangaraju, Rekha Thapar, Aravind Thavamani, Sathish Thirunavukkarasu, Joe Thomas, Sofonyas Abebaw Tiruneh, Tenaw Yimer Tiruye, Mariya Vladimirovna Titova, Krishna Tiwari, Sojit Tomo, Marcello Tonelli, Mathilde Touvier, Marcos Roberto Tovani-Palone, Khaled Trabelsi, Ngoc Ha Tran, Nguyen Tran Minh Duc, Domenico Trico, Guesh Mebrahtom Tsegay, Munkhtuya Tumurkhuu, Sok Cin Tye, Aniefiok John Udoakang, Shahid Ullah, Saeed Ullah, Atta Ullah, Muhammad Umair, Umar Muhammad Umar, LAWAN UMAR, Dinesh Upadhya, Era Upadhyay, Jibrin Sammani Usman, Masoud Vaezghasemi, Jef Van den Eynde, Joe Varghese, Siavash Vaziri, Balachandar Vellingiri, Narayanaswamy Venketasubramanian, Georgios-Ioannis Verras, Victor E Villalobos-Daniel, Vasily Vlassov, Stein Emil Vollset, Rade Vukovic, Mohammad Wahiduzzaman, Yanzhong Wang, Shu Wang, Cong Wang, Xingxin Wang, Kosala Gayan Weerakoon, Fei-Long Wei, Anggi Lukman Wicaksana, Nuwan Darshana Wickramasinghe, Dakshitha Praneeth Wickramasinghe, Peter Willeit, Qing Xia, Guangqin Xiao, Wanqing Xie, Suowen Xu, Xiaoyue Xu, Galal Yahya, Yuichiro Yano, Haiqiang Yao, Amir Yarahmadi, Habib Yaribeygi, Pengpeng Ye, Subah Abderehim Yesuf, Dehui Yin, Dong Keon Yon, Naohiro Yonemoto, Deniz Yuce, Ismaeel Yunusa, Giulia Zamagni, Michael Zastrozhin, Mohammed G M Zeariya, Xiaoyi Zhang, Liqun Zhang, Jingya Zhang, Haijun Zhang, Zhiqiang Zhang, Casper J P Zhang, Hanqing Zhao, David X Zheng, Anthony Zhong, Claire Chenwen Zhong, Jiayan Zhou, Bin Zhu, Magdalena Zielińska, Zhiyong Zou, Sa'ed H Zyoud

Drafting the work or revising it critically for important intellectual content

Yohannes Habtegiorgis Abate, Abdallah H A Abd Al Magied, Samar Abd ElHafeez, Sherief Abd-Elsalam, Arash Abdollahi, Auwal Abdullahi, Hansani Madushika Abeywickrama, Olumide Abiodun, Shady Abohashem, Hasan Abualruz, Eman Abu-Gharbieh, Hana J Abukhadijah, Niveen ME Abu-Rmeileh, Salahdein Aburuz, Ahmed Abu-Zaid, Lisa C. Adams, Mesafint Molla Adane, Isaac Yeboah Addo, Kamoru Ademola

Adedokun, Nurudeen A Adegoke, Juliana Bunmi Adetunji, Usha Adiga, Qorinah Estiningtyas Sakilah Adnani, Abdelrahman Yousry Afify, Aanuoluwapo Adeyimika Afolabi, Saira Afzal, Muhammad Sohail Afzal, Suneth Buddhika Agampodi, César Agostinis Sobrinho, Bright Opoku Ahinkorah, Danish Ahmad, Fuzail Ahmad, Muayyad M Ahmad, Ayman Ahmed, Haroon Ahmed, Luai A Ahmed, Mehrunnisha Sharif Ahmed, Syed Anees Ahmed, Marjan Ajami, Mohammed Ahmed Akkaif, Zain Al Ta'ani, Yazan Al Thaher, Tariq A Alalwan, Khurshid Alam, Rasmieh Mustafa Al-amer, Amani Alansari, Fahmi Y. Al-Ashwal, Mohammed Albashtawy, Abdelazeem M Algammal, Dari Alhuwail, Abid Ali, Mohammed Usman Ali, Waad Ali, Samah W Al-Jabi, Ahmad Alkhatib, Wael Almahmeed, Mahmoud A Alomari, Saleh A Alqahtani, Abdullah A Alqarni, Ahmad Alrawashdeh, Intima Alrimawi, Sahel Majed Alrousan, Najim Z Alshahrani, Awais Altaf, Nelson Alvis-Guzman, Mohammad Al-Wardat, Yaser Mohammed Al-Worafi, Safwat Aly, Hany Aly, Karem H Alzoubi, Sohrab Amiri, Hubert Amu, Dickson A Amugsi, Ganiyu Adeniyi Amusa, Roshan A Ananda, Robert Ancuceanu, Sumbul Ansari, Boluwatife Stephen Anuoluwa, Iyadunni Adesola Anuoluwa, Saeid Anvari, Anayochukwu Edward Anyasodor, Juan Pablo Arab, Jalal Arabloo, Hidayat Arifin, Benedetta Armocida, Johan Ärnlöv, Mahwish Arooj, Amit Arora, Kurnia Dwi Artanti, Ashokan Arumugam, Seyyed Shamsadin Athari, Prince Atorkey, Alok Atreya, Adedapo Wasiu Awotidebe, Sadat Abdulla Aziz, Ahmed Y. Azzam, Domenico Azzolino, Peter S Azzopardi, Mina Babashahi, Giridhara Rathnaiah Babu, Ruhai Bai, Atif Amin Baig, Shankar M Bakkannavar, Senthilkumar Balakrishnan, Ovidiu Constantin Baltatu, Kiran Bam, Mainak Bardhan, Hiba Jawdat Barqawi, Simon Barquera, Amadou Barrow, Lingkan Barua, Mohammad-Mahdi Bastan, Reza Bayat, Mulat Tirfie Bayih, Priyamadhaba Behera, Umar Muhammad Bello, Luis Belo, Maria Bergami, Kidanemaryam Berhe, Ajeet Singh Bhadoria, Neeraj Bhala, Ravi Bharadwaj, Sonu Bhaskar, Ajay Nagesh Bhat, Priyadarshini Bhattacharjee, Jasvinder Singh Bhatti, Gurjit Kaur Bhatti, Catherine Bisignano, Bijit Biswas, Bruno Bizzozero Peroni, Espen Bjertness, Tone Bjørge, Sri Harsha Boppana, Samuel Adolf Bosoka, Souad Bouaoud, Edward J Boyko, Dejana Braithwaite, Javier Brazo-Sayavera, Hermann Brenner, Dana Bryazka, Raffaele Bugiardini, Yasser Bustanji, Mehtap Çakmak Barsbay, Daniela Calina, Luciana Aparecida Campos, Si Cao, Angelo Capodici, Andre F Carvalho, Márcia Carvalho, Monica Cattafesta, Maria Sofia Cattaruzza, Luca Cegolon, Francieli Cembranel, Ester Cerin, Joshua Chadwick, Eeshwar K Chandrasekar, Jung-Chen Chang, Vijay Kumar Chattu, Anis Ahmad Chaudhary, Akhilanand Chaurasia, Haowei Chen, An-Tian Chen, Nicholas WS Chew, Ritesh Chimoriya, Patrick R Ching, Bryan Chong, Hitesh Chopra, Shivani Chopra, Sunghyun Chung, Karly I Cini, Rebecca M Cogen, Alyssa Columbus, Nathalie Conrad, Michael H Criqui, Natalia Cruz-Martins, Alanna Gomes da Silva, Xiaochen Dai, Mayank Dalakoti, Emanuele D'Amico, Lucio D'Anna, Pojsakorn Danpanichkul, Samuel Demissie Darcho, Nihar Ranjan Dash, Edgar Denova-Gutiérrez, Meseret Derbew Molla, Ismail Dergaa, Devananda Devegowda, Arkadeep Dhali, Bibha Dhungel, Daniel Diaz, Monica Dinu, Thanh Chi Do, Camila Bruneli do Prado, Milad Dodangeh, Phidelia Theresa Doegah, Sushil Dohare, Klara Georgieva Dokova, Paul Narh Doku, Neda Dolatkhah, Mario D'Oria, Ojas Prakashbhai Doshi, Rajkumar Prakashbhai Doshi, Mi Du, Samuel C Dumith, Dorothea Dumuid, Bruce B Duncan, Ashkan Eighaei Sedeh, Michael Ekholuenetale, Rabie Adel El Arab, Ibrahim Farahat El Bayoumy, Said El-Ashker, Iffat Elbarazi, Islam Y Elgendy, Muhammed Elhadi, Ashraf A El-Metwally, Mohamed A Elmonem, Mohamed Hassan Elnaem, Randa Elsheikh, Chadi Eltaha, Theophilus I Emeto, Maysa Eslami, Natalia Fabin, Heidar Fadavian, Adeniyi Francis Fagbamigbe, Carla Sofia e Sá Farinha, MoezAlIslam Ezzat Mahmoud Faris, Mohsen Farjoud Kouhanjani, Samuel Aanuoluwapo Fasusi, Rodrigo Fernandez-Jimenez, Nuno Ferreira, Nataliya A Foigt, Morenike Oluwatoyin Folayan, Roham Foroumadi, Matteo Foschi, Kate Louise Francis, Aleš Gába, Muktar A Gadanya, Emmanuela Gakidou, Yaseen Galali, Silvano Gallus, Balasankar Ganesan, Shivaprakash Gangachannaiah, Miglas Welay Gebregergis, Lemma Getacher, Molla Getie, Fataneh Ghadirian, Ramy Mohamed Ghazy, Artyom Urievich Gil, Tiffany K Gill, Richard F Gillum, Alem Abera Girmay, Alessandra C Goulart, Ayman Grada, Michal Grivna,

Ashna Grover, Giovanni Guarducci, Mohammed Ibrahim Mohialdeen Gubari, Avirup Guha, Snigdha Gulati, Damitha Asanga Gunawardane, Zheng Guo, Rajeev Gupta, Bhawna Gupta, Rahul Gupta, Roberth Steven Gutiérrez-Murillo, Najah R Hadi, Zahra Hadian, Nadia M Hamdy, Nasrin Hanifi, Graeme J Hankey, Allie Haq, Josep Maria Haro, Faizul Hasan, Reza Hashempour, Mohammad Hashem Hashempur, Md Saquib Hasnain, Amr Hassan, Nageeb Hassan, Afagh Hassanzade Rad, Rasmus J Havmoeller, Simon I Hay, Jeffrey J Hebert, Kamal Hezam, Yuta Hiraike, Mai Hoang, Ramesh Holla, Zin Wai Htay, Yifei Hu, Mohamed Ibrahim Husseiny, Foziya Mohammed Hussien, Hong-Han Huynh, Ramzi Ibrahim, Anel Ibrayeva, Olayinka Stephen Ilesanmi, Irena M Ilic, Milena D Ilic, Arit Inok, Md. Rabiul Islam, Sheikh Mohammed Shariful Islam, Md Sahidul Islam, Nahlah Elkudssiah Ismail, Hiroyasu Iso, Gaetano Isola, Mosimah Charles Ituka, Chinwe Juliana Iwu-Jaja, Assefa N Iyasu, Louis Jacob, Shabbar Jaffar, Haitham Jahrami, Akhil Jain, Mihajlo Jakovljevic, Syed Sarmad Javaid, Sathish Kumar Jayapal, Shubha Jayaram, Felix K Jebasingh, Alelign Tasew Jema, Bijay Mukesh Jeswani, Jost B Jonas, Nitin Joseph, Charity Ehimwenma Joshua, Jacek Jerzy Jozwiak, Mikk Jürisson, Ali Kabir, Vidya Kadashetti, Ashish Kumar Kakkar, Sanjay Kalra, Saddam Fuad Kanaan, Samuel Berchi Kankam, Arun R Kanmanthareddy, Kehinde Kazeem Kanmodi, Rami S Kantar, Debasish Kar, Paschalis Karakasis, Sahand Karimzadhagh, Joonas H Kauppila, Gbenga A Kayode, Shemsu Kedir, Dimitrios Kehagias, Jessica A Kerr, Ariz Keshwani, Emmanuelle Kesse-Guyot, Himanshu Khajuria, Pantea Khalili, Ajmal Khan, Maseer Khan, Md Abdullah Saeed Khan, Mohammad Jobair Khan, Moien AB Khan, Muhammad Shahzeb Khan, Nusrat Khan, Vishnu Khanal, Shaghayegh Khanmohammadi, Moawiah Mohammad Khatatbeh, Feriha Fatima Khidri, Atulya Aman Khosla, Sepehr Khosravi, Mahmood Khosrowjerdi, Jinho Kim, Min Seo Kim, Adnan Kisa, Ladli Kishore, Shivakumar KM, Michail Kokkorakis, Oleksii Korzh, Karel Kostev, Sindhura Lakshmi Koulmane Laxminarayana, Chong-Han Kua, Barthelemy Kuate Defo, Mohammed Kuddus, Mukhtar Kulimbet, Vishnutheertha Kulkarni, Setor K Kunutsor, Om P Kurmi, Maria Dyah Kurniasari, Dian Kusuma, Carlo La Vecchia, Ben Lacey, Chandrakant Lahariya, Daphne Teck Ching Lai, Iván Landires, Zohra S Lassi, Nhi Huu Hanh Le, Paul H Lee, An Li, Wei Li, Yongze Li, Stephen S Lim, Queran Lin, Daniel Lindholm, Jue Liu, José Francisco López-Gil, Stefan Lorkowski, Giancarlo Lucchetti, Alessandra Lugo, Angelina M Lutambi, Zheng Feei Ma, Javier A Magaña Gómez, Nastaran Maghbouli, Elaheh Malakan Rad, Reza Malekzadeh, Kashish Malhotra, Ahmad Azam Malik, Deborah Carvalho Malta, Abdullah A Mamun, Emmanuel Manu, Hamid Reza Marateb, Mirko Marino, Abdoljalal Marjani, Ramon Martinez-Piedra, Miquel Martorell, Sammer Marzouk, Yasith Mathangasinghe, Fernanda Penido Matozinhos, Thushara Matthias, Rita Mattiello, Andrea Maugeri, Steven M McPhail, Enkeleint A Mechili, María Paz Medel Salas, Asim Mehmood, Hadush Negash Meles, Walter Mendoza, Ritesh G Menezes, Emiru Ayalew Mengistie, Sultan Ayoub Meo, Tomislav Mestrovic, Sachith Mettananda, Chamila Dinushi Kukulege Mettananda, Ted R Miller, Wai-kit Ming, Awoke Misganaw, Madhukar Mittal, Mona Gamal Mohamed, Nouh Saad Mohamed, Taj Mohammad, Sakineh Mohammad-Alizadeh-Charandabi, Abdollah Mohammadian-Hafshejani, Shafiu Mohammed, Ali H Mokdad, Lorenzo Monasta, Stefania Mondello, Mohammad Ali Moni, Sara Montazeri Namin, AmirAli Moodi Ghalibaf, Shane Douglas Morrison, Francesk Mulita, Yanjinlkham Munkhsaikhan, Efren Murillo-Zamora, Christopher J L Murray, Sani Musa, Ghulam Mustafa, Sathish Muthu, Woojae Myung, Abdulrazaq Bidemi Nafiu, Gopal Nambi, Shumaila Nargus, Gustavo G Nascimento, Mahmoud Nassar, Javaid Nauman, Samidi Nirasha Kumari Navaratna, Nawsherwan, Biswa Prakash Nayak, Masoud Negahdary, Ruxandra Irina Negoi, Ionut Negoi, Soroush Nematollahi, Samata Nepal, Marie Ng, Josephine W Ngunjiri, Dang Nguyen, Phat Tuan Nguyen, Robina Khan Niazi, Luciano Nieddu, Mahdieh Niknam, Taxiarchis Konstantinos Nikolouzakis, Jan Rene Nkeck, Mamoona Noreen, Jean Jacques Noubiap, Mehran Nouri, Chisom Adaobi Nri-Ezedi, Fred Nugen, Virginia Nuñez-Samudio, Aqsha Nur, Chimezie Igwegbe Nzoputam, Bogdan Oancea, Ismail A Odetokun, Akinyemi O D Ofakunrin, James Odhiambo Oguta, In-Hwan Oh, Sylvester Reuben Okeke,

Osaretin Christabel Okonji, Andrew T Olagunju, Oladotun Victor Olalusi, Gláucia Maria Moraes Oliveira, Arão Belitardo Oliveira, Hany A Omar, Nesredin Ahmed Omer, Michal Ordak, Alberto Ortiz, Augustus Osborne, Wael M S Osman, Adrian Otoiu, Amel Ouyahia, Mayowa O Owolabi, Irene Amoakoh Owusu, Kolapo Oyebola, Tope Oyelade, Mahesh Padukudru P A, Alicia Padron-Monedero, Jagadish Rao Padubidri, Tamás Palicz, Sujogya Kumar Panda, Songhomitra Panda-Jonas, Seithikurippu R Pandi-Perumal, Shahina Pardhan, Romil R Parikh, Roberto Passera, Jay Patel, Dimitrios Patoulias, George C Patton, Susan Paudel, Marcos Pereira, Norberto Perico, Simone Perna, Ionela-Roxana Petcu, Fanny Emily Petermann-Rocha, Tung Thanh Pham, Hoang Nhat Pham, Saeed Pirouzpanah, Djordje S Popovic, Isabel Potani, Jalandhar Pradhan, Pranil Man Singh Pradhan, Manya Prasad, Akila Prashant, Elton Junio Sady Prates, Jagadeesh Puvvula, Ibrahim Qattea, Yanan Qiao, Venkatraman Radhakrishnan, Maja R Radojčić, Md. Mosfequr Rahman, Mohammad Hifz Ur Rahman, Mohammad Rahmanian, Masoud Rahmati, Ivano Raimondo, Jeffrey Pradeep Raj, Prashant Rajput, Mahmoud Mohammed Ramadan, Chitra Ramasamy, Shakthi Kumaran Ramasamy, Kritika Rana, Mithun Rao, Sowmya J Rao, Sina Rashedi, Ashkan Rasouli-Saravani, Devarajan Rathish, Ilari Rautalin, Salman Rawaf, Elrashdy M. Moustafa Mohamed Redwan, Sanika Rege, Aqeeb Ur Rehman, Ana Reis-Mendes, Giuseppe Remuzzi, João Rocha Rocha-Gomes, Thales Philipe Rodrigues da Silva, Jefferson Antonio Buendia Rodriguez, Leonardo Roever, Peter Rohloff, Debby Syahru Romadlon, Gholamreza Roshandel, Nitai Roy, Godfrey M Rwegerera, Aly M A Saad, Maha Mohamed Saber-Ayad, Leila Sabzmakan, Kabir P Sadarangani, Basema Ahmad Saddik, Masoumeh Sadeghi, Umar Saeed, Dominic Sagoe, Fatemeh Saheb Sharif-Askari, Amirhossein Sahebkar, Soumya Swaroop Sahoo, Afeez Abolarinwa Salami, Luciane B Salaroli, Samreen Saleem, Marwa Rashad Salem, Dauda Salihu, Abdallah M Samy, Milena M Santric-Milicevic, Tanmay Sarkar, Mohammad Sarmadi, Sachin C Sarode, Gargi Sachin Sarode, Michele Sassano, Susan M Sawyer, Sonia Saxena, Ganesh Kumar Saya, Maria Inês Schmidt, Art Schuermans, Siddharthan Selvaraj, Ashenafi Kibret Sendekie, Sadaf G Sepanlou, Yashendra Sethi, Allen Seylani, Mahan Shafie, Sweni Shah, Samiah Shahid, Moyad Jamal Shahwan, Muhammad Aaqib Shamim, Mehran Shams-Beyranvand, Alfiya Shamsutdinova, Mohd Shanawaz, Mohammed Shannawaz, Medha Sharath, Amin Sharifan, Manoj Sharma, Ujjawal Sharma, Vishal Sharma, Rekha Raghuveer Shenoy, Pavanchand H Shetty, Min-Jeong Shin, Mahsa Shirani Lapari, Aminu Shittu, Seyed Afshin Shorofi, Gambhir Shrestha, Kerem Shuval, Emmanuel Edwar Siddig, Mario Siervo, Diego Augusto Santos Silva, Luís Manuel Lopes Rodrigues Silva, Surjit Singh, Jasvinder A Singh, Lucky Singh, Harmanjit Singh, Kalpana Singh, Poornima Suryanath Singh, Anna Aleksandrovna Skryabina, Amanda E Smith, Sameh S M Soliman, Soroush Soraneh, Michael Spartalis, Bahadar S Srichawla, Muhammad Haroon Stanikzai, Antonina V Starodubova, Pete Stubbs, Muritala Odidi Suleiman Odidi, Aleksander Sulkowski, Anusha Sultan Meo, Dev Ram Sunuwar, Chandan Kumar Swain, Lukasz Szarpak, Sree Sudha T Y, Seyed-Amir Tabatabaeizadeh, Shima Tabatabai, Celine Tabche, Mohammad Tabish, Mircea Tampa, Jacques Lukenze Tamuzi, Ker-Kan Tan, Manoj Tanwar, Saba Tariq, Nathan Y Tat, Reem Mohamad Hani Temsah, Mohamad-Hani Temsah, Dufera Rikitu Terefa, Jay Tewari, Pugazhenthan Thangaraju, Sathish Thirunavukkarasu, Tenaw Yimer Tiruye, Krishna Tiwari, Sojit Tomo, Marcello Tonelli, Mathilde Touvier, Marcos Roberto Tovani-Palone, Khaled Trabelsi, Thang Huu Tran, Ngoc Ha Tran, Nguyen Tran Minh Duc, Domenico Trico, Thien Tan Tri Tai Truyen, Sok Cin Tye, Aniefiok John Udoakang, Shahid Ullah, Muhammad Umair, Umar Muhammad Umar, LAWAN UMAR, Brigid Unim, Dinesh Upadhya, Era Upadhyay, Jibrin Sammani Usman, Damla Ustunsoz, Masoud Vaezghasemi, Asokan Govindaraj Vaithinathan, Jef Van den Eynde, Joe Varghese, Tommi Juhani Vasankari, Balachandar Vellingiri, Narayanaswamy Venketasubramanian, Madhur Verma, Georgios-Ioannis Verras, Victor E Villalobos-Daniel, Vasily Vlassov, Stein Emil Vollset, Rade Vukovic, Mohammad Wahiduzzaman, Yanzhong Wang, Shu Wang, Cong Wang, Xingxin Wang, Kosala Gayan Weerakoon, Fei-Long Wei, Nuwan Darshana Wickramasinghe,

Dakshitha Praneeth Wickramasinghe, Peter Willeit, Marcin W Wojewodzic, Qing Xia, Galal Yahya, Kazumasa Yamagishi, Haiqiang Yao, Amir Yarahmadi, Dong Keon Yon, Naohiro Yonemoto, Deniz Yuce, Michael Zastrozhin, Mohammed G M Zeariya, Xiaoyi Zhang, Haijun Zhang, Zhiqiang Zhang, Casper J P Zhang, David X Zheng, Anthony Zhong, Claire Chenwen Zhong, Bin Zhu, Magdalena Zielińska, Osama A Zitoun, Ghazal Zoghi, Sa'ed H Zyoud

Managing the estimation or publications process

Xiaochen Dai, Simon I Hay, Nicholas J Kassebaum, Jessica A Kerr, Paulina A Lindstedt, Ali H Mokdad, Erin C Mullany, Christopher J L Murray, Erin M O'Connell, George C Patton, Susan M Sawyer, Amanda E Smith
